# CD8^+^XCR1^neg^ Dendritic Cells Express High Levels of Toll-Like Receptor 5 and a Unique Complement of Endocytic Receptors

**DOI:** 10.3389/fimmu.2018.02990

**Published:** 2019-01-16

**Authors:** Ben Wylie, James Read, Anthony C. Buzzai, Teagan Wagner, Niamh Troy, Genevieve Syn, Shane R. Stone, Bree Foley, Anthony Bosco, Mark N. Cruickshank, Jason Waithman

**Affiliations:** Telethon Kids Institute, University of Western Australia, Perth, WA, Australia

**Keywords:** dendritic cells, TLR5, XCR1, immunosurveillance, transcriptomics, microarray

## Abstract

Conventional dendritic cells (cDC) resident in the lymphoid organs of mice have been classically divided into CD8^+^ and CD8^neg^ subsets. It is well-established that CD8^+^ dendritic cells (DCs) and their migratory counterparts in the periphery comprise the cross-presenting cDC1 subset. In contrast, CD8^neg^ DCs are grouped together in the heterogeneous cDC2 subset. CD8^neg^ DCs are relatively poor cross-presenters and drive more prominent CD4^+^ T cell responses against exogenous antigens. The discovery of the X-C motif chemokine receptor 1 (XCR1) as a specific marker of cross-presenting DCs, has led to the identification of a divergent subset of CD8^+^ DCs that lacks the ability to cross-present. Here, we report that these poorly characterized CD8^+^XCR1^neg^ DCs have a gene expression profile that is consistent with both plasmacytoid DCs (pDCs) and cDC2. Our data demonstrate that CD8^+^XCR1^neg^ DCs possess a unique pattern of endocytic receptors and a restricted toll-like receptor (TLR) profile that is particularly enriched for TLR5, giving them a unique position within the DC immunosurveillance network.

## Introduction

Dendritic cells (DCs) play a key role in the immunosurveillance of pathogens and tumors, being specialized in the acquisition and presentation of foreign antigens and the regulation of T cell immunity (1). Numerous phenotypically distinct subsets of conventional DCs (cDC) exist in the periphery and secondary lymphoid organs, where they perform unique roles suggestive of a division of labor (2, 3). Classically, CD8^+^ and CD103^+^ DCs comprise the cross-presenting cDC1 family (4, 5), which can also be identified by expression of the X-C motif chemokine receptor 1 (XCR1) (6, 7). The primary roles of these DCs are the cross-presentation of viral (8–10) and tumor antigens (11), and the maintenance of peripheral tolerance (12–14). CD8^neg^ DCs comprise the cDC2 family, which is more heterogeneous and less well characterized. cDC2s universally express signal regulatory protein alpha (SIRPα) on their surface, which is inversely correlated to XCR1 expression (15). Individual cDC2 subsets carry out specialized roles in the generation of CD4^+^ T cell immunity (16–18). Plasmacytoid DCs (pDCs) are the third distinct lineage of DCs and are the primary source of type I interferon (IFN) following viral infection (19). pDCs can be distinguished from cDC subsets by their expression of bone marrow stromal antigen 2 (BST2) (20) and sialic acid binding Ig-like lectin H (Siglec H) (21). Despite the identification of these subset-specific surface markers, the separation of DCs based on surface antigen expression remains complex and difficult across species (22).

The discovery of DC lineage-specific transcription factors has facilitated the consistent organization of DC subsets based on their ontogeny (11, 23–26). cDC1 and cDC2 arise from distinct precursor populations (27) under the control of the master transcription factors interferon regulatory factor 8 (IRF8) and interferon regulatory factor 4 (IRF4), respectively. IRF8 also plays a role in determining the phenotype of mature pDCs and regulates gene expression in these cells but is not critical for their development (28). The generation of pDCs instead relies on the transcription factors E2-2 (20) and SpiB (29), highlighting their unique ontogeny compared to cDCs. Transcriptional profiling of individual DC subsets (30–33) has allowed for detailed interrogation of their unique gene expression patterns and the definition of subset-specific gene signatures (31, 32). In particular, data from the Immunological Genome Project (ImmGen) database (34) has been used to infer transcriptional programs that control DC lineage development, homeostasis and function (31). Recently, the combination of DC phenotyping and ontogeny has refined the definition of DC subsets and provided a simplified and unified DC nomenclature (35). However, the precise role of some DC subsets in the generation of immunity remains to be elucidated.

DCs possess a varied array of pattern recognition receptors that facilitate active sensing of their environment and the uptake of antigen from their surroundings. The expression of specific Toll-like receptors (TLR) and C-type lectin receptors (CLRs) can be used to differentiate unique subsets of DCs (36) and infer their function. TLR3 and TLR7 bind specifically to viral RNAs, inducing strong anti-viral immunity (37), and are expressed exclusively on cDC1 and pDCs, respectively (38). cDC2s exhibit greater expression of TLR5, which recognizes bacterial flagellin (39). Signaling via TLR5 drives specific gene expression programs directing Th1 and Th17 type immunity (40, 41). The expression of CLRs has also be used to differentiate between related subsets of DCs. Inverse expression of the dendritic cell inhibitory receptor 2 (DCIR2) and dendritic cell-associated lectin 2 (DCAL2) distinguishes two unique subsets of CD11b^+^ DCs that respond to unique TLR ligands and produce distinct sets of cytokines (36). Therefore, determining the TLR and CLR expression profiles of a given DC subset may provide useful information about their specific DC lineage and role in immunosurveillance.

To date, the role of CD8^+^XCR1^neg^ DCs in the generation of immunity remains unresolved. We have reported previously that four unique subsets of XCR1^+^ DCs are present in the skin-draining lymph nodes (sdLNs) of mice (42), where they exist alongside CD8^+^XCR1^neg^ DCs. Here, we use transcriptome profiling to analyze the gene expression of CD8^+^XCR1^neg^ DCs and infer their position within the complex network of DC immunosurveillance. We find that these DCs have a transcriptional profile that shares features with pDCs and cDC2 subsets but is markedly different to that of CD8^+^XCR1^+^ DCs. Our data shows that CD8^+^XCR1^neg^ DCs display a high surface expression of the flagellin receptor, TLR5, and express a unique repertoire of pattern recognition and endocytic receptors, linking them to both pDC and cDC2 lineages.

## Methods

### Mice

C57BL/6 mice were purchased from the Animal Resource Center, Murdoch, Western Australia. Mice were housed under specific pathogen-free conditions at the Telethon Kids Institute Bioresources Center and provided with standard food pellets and water *ad libitum* on a 12 h light/dark cycle. All animal experiments were performed in accordance with protocols approved by the Telethon Kids Institute Animal Ethics Committee (Ethics Application ID: 243, 290) and conformed to the National Health and Medical Research Council of Australia code of practice for the care and use of animals for scientific purposes.

### Sample Preparation and Sorting

DCs were isolated from lymphoid organs as described previously (9). Briefly, single cell suspensions were prepared from pooled skin-draining lymph nodes and enriched for cDCs using antibody depletion and magnetic bead enrichment. Cells were incubated with anti-mouse monoclonal antibodies CD11c Brilliant Violet (BV) 421, MHC II APC-Cy7, CD8α PE-CF594, XCR1 PE, CD103 FITC, and CD326 PE-Cy7 purchased from BD Biosciences (Franklin Lakes, NJ, USA) or BioLegend (San Diego,CA, USA) to identify DC subsets. DCs were sorted from Propidium Iodide negative events using a FACSAriaIII cell sorter (BD Biosciences). During sorting, DCs were collected in buffer containing FCS (Sigma-Aldrich, St. Louis, MO, USA) and EDTA (Sigma-Aldrich) and kept on ice at all times before being recovered by centrifugation and stored in Trizol reagent (Invitrogen, Carlsbad, CA, USA).

### DC Phenotyping by Flow Cytometry

Single cell suspensions were incubated with anti-mouse monoclonal antibodies CD8α PE-CF594 (C53-6.7), XCR1 PE (ZET), CD11c BV421 (N418), MHC II APC-Cy7 (M5/114), CD103 BV510 (N290), CD326 PE-Cy7 (G8.8), CD4 PE-Cy7 (RM4-5), CD11b PERCP-Cy5-5 (M1/70), CD206 FITC (C068C2), CD301b APC (URA-1), Siglec-H APC (551), PDCA-1 BV650 (927), CD274 FITC (10F.9G2), TLR3 PE (11F8), TLR5 APC (ACT5), and TLR7 PE (A94B10), purchased from BD Biosciences or BioLegend. For intracellular cytokine staining of TLR3 and TLR7 cells were first fixed with 1% paraformaldehyde (Sigma-Aldrich) and then incubated with relevant antibodies to intracellular targets in a buffer containing 0.2% saponin to permeabilize cells (Sigma-Aldrich). Post intracellular staining, cells were washed and kept in serum free buffer until analysis. Multi-parameter analysis was performed on a LSRFortessa (BD Biosciences). All data were analyzed with FlowJo (v10, Tree Star, Ashland, OR, USA).

### RNA Isolation and Transcriptome Profiling

Total RNA was extracted from up to 50,000 DCs per subset using Trizol (Invitrogen) phase separation and purified using the RNeasy MinElute kit (Qiagen, Hilden, Germany). RNA integrity was measured using a BioAnalyzer (Agilent, Santa Clara, CA, USA) and the mean RNA integrity score +/− *SD* was 9.5 +/− 0.5 for all samples. RNA samples were shipped on dry ice to the Ramiciotti Center for Genomics (New South Wales, Australia) where sample amplification was performed with the Ovation Pico WTA v2 protocol (NuGen, San Carlos, CA, USA). Hybridisation to GeneChip™ Mouse Gene 2.0 ST microarrays (Affymetrix, Santa Clara, CA, USA) was performed according to standard procedures.

### RNA Extraction, cDNA Synthesis and RT-PCR

RNA was isolated from sorted splenic or sdLN DCs using phenol chloroform extraction and the RNEasy Mini Kit (Qiagen) according to the manufacturer's instructions. cDNA was synthesized using the SuperscriptIII First Strand Synthesis System (Invitrogen), with 1 μg of RNA per reaction according to the manufacturer's instructions. RT-PCRs were performed in 25 μL reactions consisting of 12.5 μL GoTaq Green Mastermix (Promega, Madison, WI, USA), 1 μL each of forward and reverse primers (20 μM stock), 1 μL of cDNA template, and 9.5 μL of RNase/DNase-free water. The touchdown protocol and conditions were as follows: 93°C for 3 min, followed by 15 cycles where the annealing temperature reduced from 63°C by 0.5°C per cycle, followed by 25 cycles of 93°C for 30 s, 55°C for 30 s, and 72°C for 45 s and a final extension at 72°C for 3 min. PCR products were run on a 1% agarose gel at 100 V for 25 min with the EasyLadder I DNA ladder (Meridian Biosciene, Cincinatti, OH, USA) in the first lane and visualized with ethidium bromide. *GAPDH* was used as a reference gene. For TLR 1-9 primers see Supplementary Methods. ImageJ software was used to calculate the intensity of bands via densitometry. Data are presented as relative expression normalized to *GAPDH* controls.

### Microarray Data Analysis

The microarray data were analyzed in R (v3.2.4). Raw expression data were background corrected, normalized and summarized into probe set level data using the robust multi-array average (RMA) method (43). Probe sets were re-mapped to a current genome annotation using a brain array chip description file (CDF, v019) (44). Non-informative probe sets were identified with the Proportion of Variation Accounted for by the first principal Component (PVAC) algorithm and filtered out of the analysis (45). Principal component analysis (PCA) was performed on PVAC-filtered expression data using the *PCA* function from the *FactoMineR* package. Differential expression analysis was performed using linear modeling and empirical Bayesian methods, carried out using *limma* (46). Differentially expressed genes (DEGs) were called with an adjusted *p*-value < 0.01 [Benjamini and Hochberg correction (47) for multiple testing] and Log2 fold change (FC) >1. *SWAMP* (v1.3.1) was used to determine the most likely identity of the principal components. Volcano plots were generated by plotting Log2FC and –log10 adjusted *p*-value using R based graphics and heatmaps were drawn with *gplots*. Gene set enrichment was performed using the Mouse Gene Atlas, KEGG Pathways and GO Biological Processes databases via the Enrichr online software platform (48). Ingenuity Pathway Analysis (IPA) (Qiagen) was used to identify canonical pathways associated with genes differentially expressed between CD8^+^XCR1^neg^ and CD8^+^XCR1^+^ DCs with a FC >5. A network was constructed in IPA using the “connect” and “build” functions with the same data set and filtering as for pathway analysis. Expression profiles for DC_4+_SLN, DC_8+_SLN, DC_8-4-11b+_SLN, and DC-PDC_8+_SLN subsets from the Immgen database were used as reference gene sets and data were analyzed using the Immgen MyGeneSet online tool (http://rstats.immgen.org/MyGeneSet/).

### *Ex vivo* Proliferation Assay

DCs were sorted from sdLNs or spleens and resuspended in RPMI media (Life Technologies, Carlsbad, CA, USA) supplemented with 10% FCS (Sigma-Aldrich), 2-mercaptoethanol (50 μM) (Life Technologies), L-glutamine (2 mM) (Life Technologies), penicillin (100 U/mL) (Life Technologies) and streptomycin (100 mg/mL) (Life Technologies), plus TLR agonists LPS (100 ng/mL) (Sigma-Aldrich) or flagellin (1 μg/mL) (Sigma-Aldrich), or left unstimulated and cultured at 37°C for 24 h. The supernatant was then removed and DCs were pulsed in media containing 1 nM of gD-peptide^8^ for a further 1 h. gD-specific CD4^+^ T cells (gDT-II) were isolated from the spleen and lymph nodes of gDT-II mice^6^ and purified by antibody depletion and magnetic bead enrichment. For *ex vivo* presentation assays: purified gDT-II cells were labeled with 2.5 μM CFSE (Sigma-Aldrich) and cultured with peptide-pulsed DCs at a 10:1 ratio for 4 days. Cells were then incubated with anti-mouse monoclonal antibodies CD45.1 BV421, CD4 APC, and Va3.2 PE (BD Biosciences) to identify the CD45.1^+^CD4^+^ Va3.2^+^ gDT-II population and proliferation of gDT-II cells was measured by CFSE dilution.

### Interferon Bioassay

Murine IFN standards and test samples were titrated in an IFN bioassay as described previously (49). Briefly, DCs were isolated and sorted into subsets as described previously (Supplementary Figures 1A,B). After 24 h incubation in the presence of the TLR agonists LPS (100 ng/ml) (Sigma), CpG (100 ng/mL) (Sigma) or flagellin (1 μg/ml) (Sigma), culture supernatants were collected and acid treated. Supernatants from TLR-stimulated DCs were evaluated for acid-stable IFN titres using the 50% protection from encephalomyocarditis virus (EMCV)-induced cytopathic effect (CPE) on L929 cell monolayers method (PBL Assay Science, Piscataway, NJ, USA). Cells were stained with crystal violet to assess CPE and resuspended in 200 μL of 100% methanol to read absorbance at 595 nM. IFN levels are expressed as biological activity (% viablilty) relative to uninfected control wells.

### Luminex Assay

DCs were isolated from spleens of naïve mice as described previously (9) and sorted for pDC, CD4^+^, CD8^+^XCR1^+^ and CD8^+^XCR1^neg^ subsets as above (see Supplementary Figure 1B). Two independent sorting experiments were conducted. A total of 25,000 DCs were sorted per well for each subset. DCs were stimulated for 36 h with TLR ligands flagellin (1 μg/ml), Poly I:C (10 μg/ml) (Sigma) or CpG (2 μM) or left unstimulated in serum-containing media. Culture supernatants were collected after stimulation, centrifuged to remove cell debris and stored at −80°C. For analysis, culture supernatants were thawed, diluted 1:2 in serum-containing media and run in duplicate using the Bio-plex Pro Mouse 23-plex Luminex assay (Bio-Rad) according to manufacturer's instructions on a Bio-Plex 200 instrument (Bio-Rad).

### Statistical Analysis

Statistical analyses were performed using the student's *T*-test function in the Prism software package (GraphPad, La Jolla, CA, USA). Paired samples were compared using the paired Student's *T*-test. For significance values; ^*^p < 0.05, ^**^p < 0.01, ^***^p < 0.001.

## Results

### CD8^+^ DCs in the Skin-Draining Lymph Nodes Are Divided by XCR1 Expression Into Two Subsets With Unique Gene Expression Profiles

**Figure 1:**
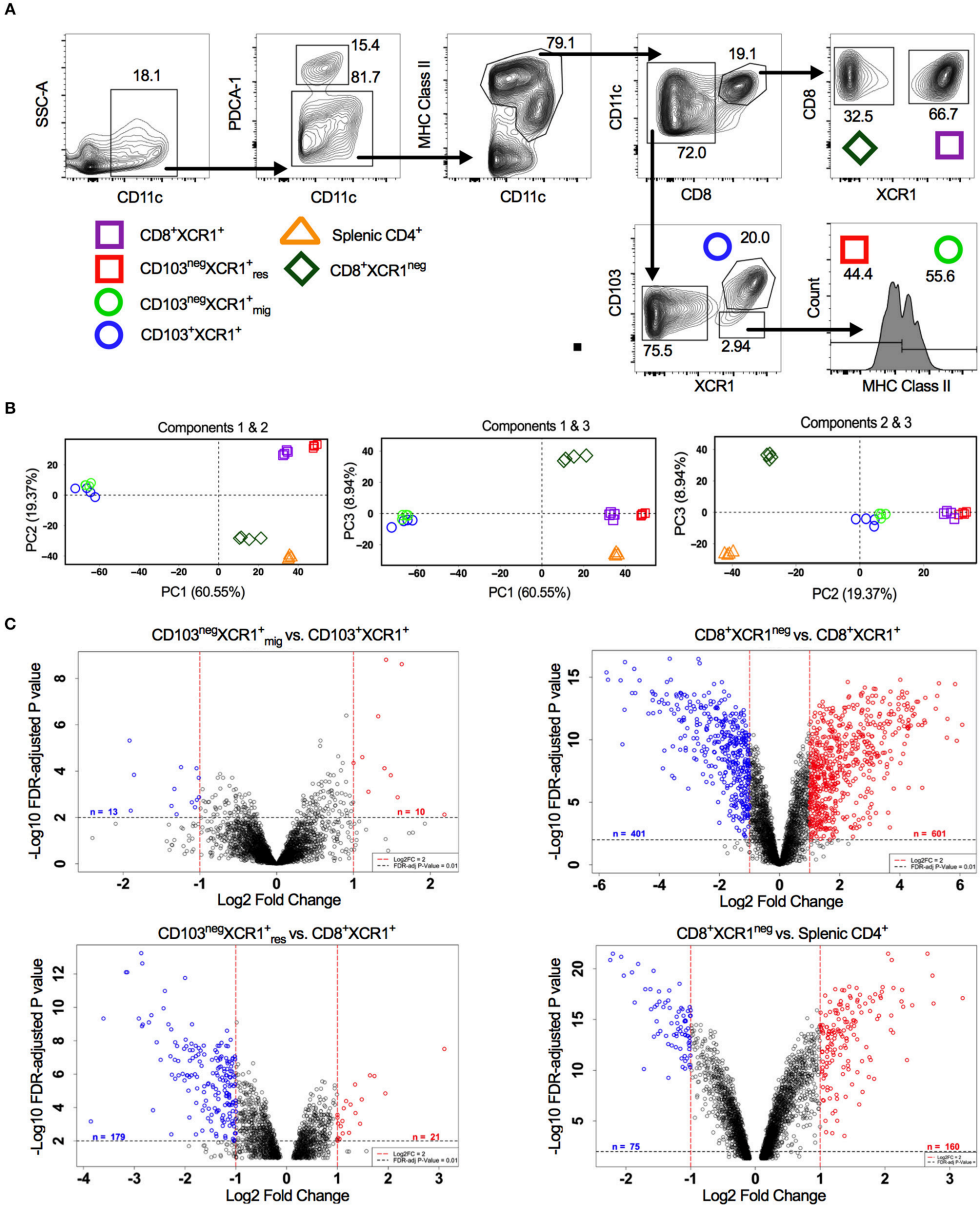
Analysis of DC subset relatedness in the skin-draining lymph nodes reveals differences in gene expression profiles of CD8^+^XCR1^neg^ and CD8^+^ XCR1^+^ DCs. **(A)** Gating strategy used to identify and sort dendritic cell subsets for microarray analysis. **(B)** RNA was extracted from sorted DC subsets and analyzed with the Affymetrix Mouse Gene 2.0 ST array. Principle component analysis of the first three components. CD8^+^XCR1^neg^ subset (diamond), XCR1^+^ resident subsets (squares), XCR1^+^ migratory subsets (circles) and splenic CD4^+^ subset (triangle). **(C)** Volcano plots displaying differential gene expression between related migratory and resident DC subsets. Red dashes represent log2FC>1 and black dashes represent adjusted *P*-value <0.01. Numbers of differentially expressed genes are indicated on each plot. Red labeled genes are up-regulated and blue labeled genes are down-regulated relative to the population indicated first in the title.

XCR1^+^ DCs display conserved surface antigen and transcription factor expression and share overlapping functions. In the sdLNs of mice multiple subsets of XCR1^+^ DCs exist. These include; resident CD8^+^XCR1^+^ and CD8^neg^CD103^neg^XCR1^+^ DCs, as well as migratory CD103^+^XCR1^+^ and CD103^neg^XCR1^+^ DCs (42). Alongside these XCR1^+^ populations a minor subset of DCs that is CD8^+^XCR1^neg^ can also be identified, which may be confused with the previously mentioned CD8^+^XCR1^+^ DCs. To determine whether further specialization exists within these subsets we performed microarray analysis on DCs sorted from the sdLNs (Figure 1A). PCA of these populations showed a high degree of relatedness between XCR1^+^ DC subsets (squares and circles), with a distinct separation of populations based on their migratory or resident origin. Conversely, CD8^+^XCR1^neg^ DCs (diamonds) appeared distinct from XCR1^+^ subsets. CD8^+^XCR1^neg^ DCs clustered together with splenic CD4^+^ DCs (triangles), a cDC2 subset, on the first two principal components (Figure 1B). To interrogate the differences between closely clustered subsets in more detail we analyzed the differential gene expression between related migratory and resident XCR1^+^ subsets. There were only 23 DEGs between the two migratory XCR1^+^ subsets (Supplementary Table 7) and 200 DEGs between the two resident XCR1^+^ subsets (Supplementary Table 2), in line with their common ontogeny and function. In contrast, when we compared the gene expression patterns of CD8^+^XCR1^+^ DCs and CD8^+^XCR1^neg^ DCs over 1,000 genes were differentially expressed (Supplementary Table 3; Figure 1C). These data are consistent with the conserved biological role of XCR1^+^ DC subsets. However, the highly divergent transcriptional profiles of CD8^+^XCR1^+^ and CD8^+^XCR1^neg^ DCs suggests that shared expression of the CD8 marker is not a reliable indicator of relatedness between these subsets. As CD8^+^XCR1^neg^ DCs lack expression of XCR1 they could be considered a cDC2 subset. We compared the gene expression of CD8^+^XCR1^neg^ DCs to that of CD4^+^ splenic DCs and identified 235 DEGs between these populations (Figure 1C; Supplementary Table 7), suggesting that their gene expression pattern is more similar to a cDC2 subset than conventional CD8^+^XCR1^+^ DCs. We set out to further elucidate the differences between CD8^+^XCR1^+^ and CD8^+^XCR1^neg^ DCs to determine whether they possess unique functions in the generation of immunity.

### CD8^+^XCR1^neg^ DC Exhibit a Unique Gene Signature in Common With Both cDC2 and pDC Subsets

**Figure 2:**
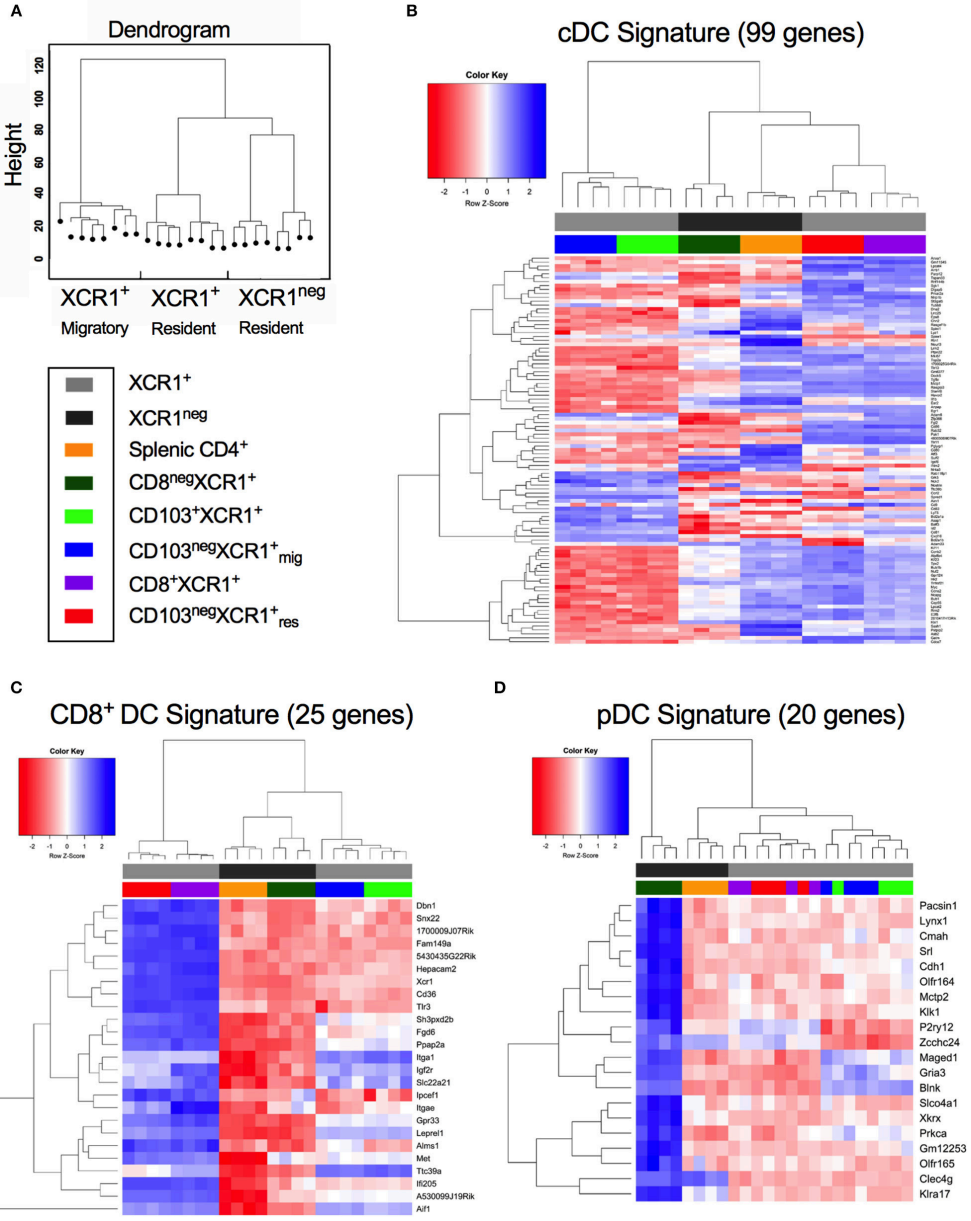
CD8^+^XCR1^neg^ DCs display up-regulated expression of a gene set associated with pDCs but not conventional or CD8^+^ DCs. Gene expression data from microarray analysis of sorted DC subsets were compared to previously reported gene signatures for conventional DCs (cDC), CD8-specific (CD8^+^ DC) DCs and pDCs (pDC) (27). **(A)** Dendrogram displaying hierarchical clustering of individual sorted DC populations divided into XCR1^+^ migratory, XCR1^+^ resident and XCR1^neg^ resident. **(B)** Differential expression of a panel of 99 cDC signature genes from microarray data of sorted DC subsets. **(C)** Differential expression of a panel of 25 CD8^+^ DC signature genes from microarray data of sorted DC subsets. **(D)** Differential expression of a panel of 20 pDC signature genes from microarray data of sorted DC subsets. Red; upregulated, Blue; down-regulated. Populations have been clustered hierarchically, dark gray bars represent XCR1^neg^ populations while light gray bars represent XCR1^+^ populations. Heatmaps are colored coded according to the legend.

cDC1, cDC2, and pDCs can be defined by the expression of subset-specific genes (31). Hierarchical clustering of DC subsets based on gene expression placed CD8^+^XCR1^neg^ DCs together with splenic CD4^+^ DCs on a common branch separated from to XCR1^+^ resident DCs. Migratory XCR1^+^ DCs clustered together on a different branch (Figure 2A). To investigate these relationships in more depth we utilized gene expression profiles established previously (31) (Supplementary Table 4) to determine the relatedness of our DC subsets to cDC, CD8^+^ DC and pDC gene signatures. Most of the 99 genes comprising the cDC signature were strongly expressed in the two resident XCR1^+^ DC subsets and splenic CD4^+^ DCs also had upregulated expression of many genes in this profile. However, expression of cDC-related genes was lower in CD8^+^XCR1^neg^ and migratory XCR1^+^ DCs (Figure 2B). Next, the expression of 25 genes reported to constitute a CD8^+^ DC signature were analyzed. Resident XCR1^+^ DCs displayed strong expression of all genes in this profile and migratory XCR1^+^ DCs had a more restricted pattern of upregulated genes. Of particular interest, CD8^+^XCR1^neg^ DCs exhibited lower levels of expression of all genes associated with the CD8^+^ gene signature (Figure 2C) and instead showed upregulation of 16 genes associated with a CD8^neg^ DC gene signature, which were also strongly expressed in CD4^+^ DCs (Supplementary Figure 2). Strikingly, CD8^+^XCR1^neg^ DCs displayed increased expression of 20 genes associated with the pDC signature, which were expressed at low levels by both the XCR1^+^ DCs and splenic CD4^+^ DCs (Figure 2D). These data are in line with previous reports demonstrating a pDC-like origin for a C-X3-C motif chemokine receptor 1 (CX_3_CR1)^+^ subset of CD8^+^ DCs (50), which likely overlaps with the CD8^+^XCR1^neg^ population in this study (51). To date little is known about the function of these DCs. However, their gene expression profile suggests they are more closely related to pDCs and cDC2 than XCR1^+^ DCs.

### CD8^+^XCR1^neg^ DCs Show Enrichment of NF-kB Signaling and Toll-Like Receptor Signaling

**Figure 3:**
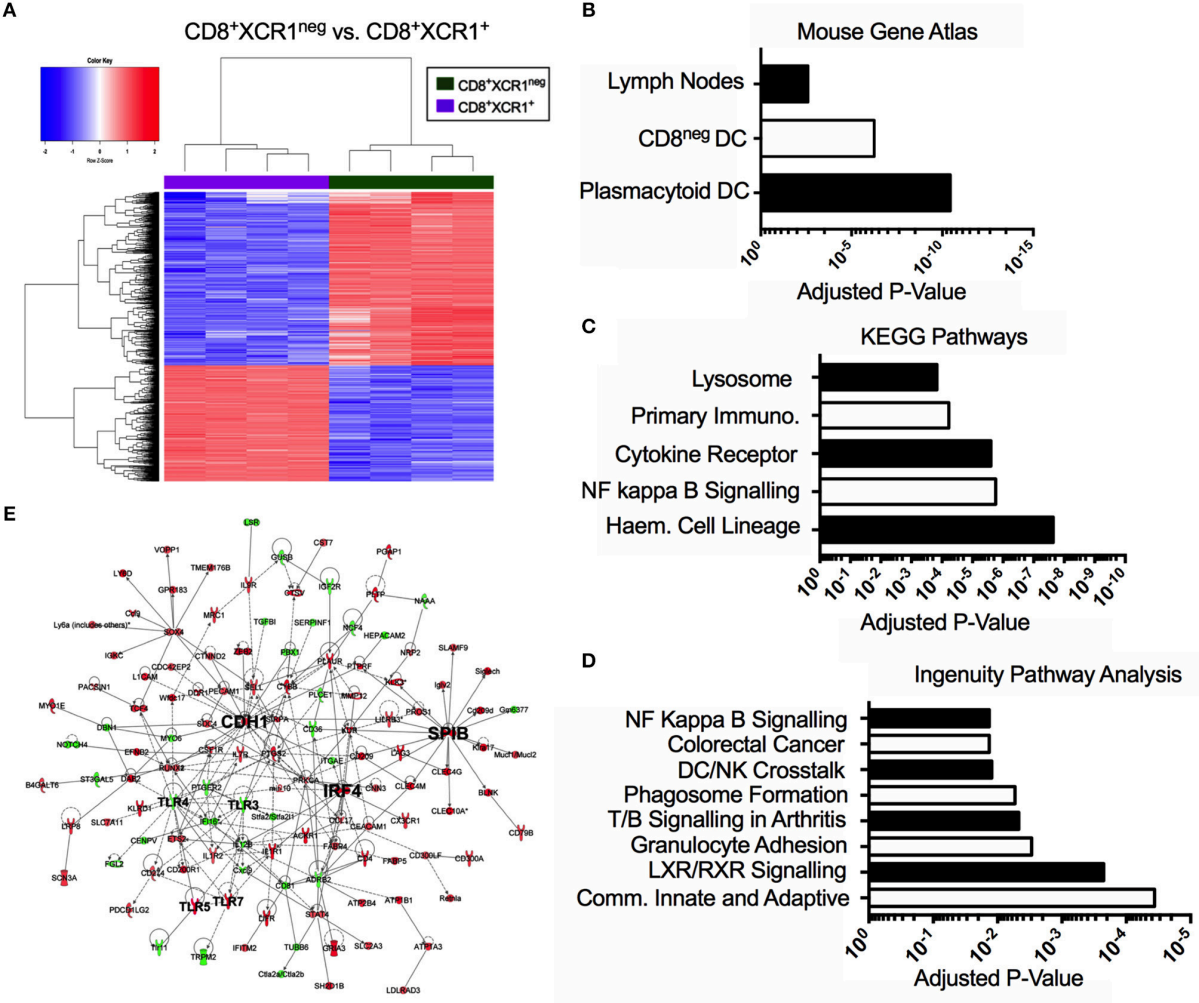
The CD8^+^XCR1^neg^ DCs transcriptome is related to both pDCs and CD8^neg^ DCs and is enriched for TLR and NF-kappa B signaling pathways. Bioinformatics tools were used to interrogate the differential gene expression observed between CD8^+^XCR1^neg^ and CD8^+^XCR1^+^ DCs. **(A)** Heatmap shows differential gene expression between CD8^+^XCR1^neg^ and CD8^+^XCR1^+^ DCs. Red; up-regulated (*n* = 601), Blue; down-regulated (*n* = 401) in CD8^+^XCR1^neg^ population. Genes upregulated in CD8^+^XCR1^neg^ DCs with a FC >2 and adj. *p*-value < 0.01 were analyzed using the Enrichr online platform and queried against: **(B)** The Mouse Genome Atlas database. Significant results (*p* < 0.05) are shown. **(C)** The KEGG pathways database. The top 5 enriched pathways are shown. **(D)** Highly differentially expressed genes (FC >5) were analyzed using IPA to identify enriched pathways and over-represented gene sets. The top 8 pathways are shown. **(E)** IPA network analysis was conducted in IPA to construct a network of differentially expressed genes (FC >5). Hub genes and important signaling molecule, such as TLRs, are labeled in bold. Red; upregulated in CD8^+^XCR1^neg^, Green; up-regulated in CD8^+^XCR1^+^. Full data tables for each analysis are provided in the Supplementary Data. Immuno, Immunodeficency; Haem, Haemopoietic.

Our microarray analysis identified 601 genes up-regulated in CD8^+^XCR1^neg^ DCs when compared to CD8^+^XCR1^+^ DCs (Figure 3A). To determine the relatedness of CD8^+^XCR1^neg^ DCs to specific immune cell subtypes we queried these 601 genes against the Mouse Gene Atlas database. The two strongest associations reported were with pDCs (adjusted *p*-value = 3.654e-11) and CD8^neg^ DCs (adjusted *p*-value = 5.725e-07), while there was no significant association with CD8^+^ DCs (Figure 3B, Supplementary Table 5). Using these same genes to query the GO Biological Processes database for enriched GO terms, we found that genes up-regulated in CD8^+^XCR1^neg^ DCs were involved in many key DC processes including; T cell activation, cytokine production and regulation of defense responses (Supplementary Table 5). Querying the KEGG Pathways database we noted enrichment in the NF-κB signaling pathway (adjusted *p*-value = 1.748e-06) (Figure 3C, Supplementary Table 5), which is a primary signaling pathway downstream of TLR activation in DCs. These data were consistent with IPA analysis, which also identified enrichment of the NF-kB signaling pathway (adjusted *p*-value = 1.35e-02), which was predicted by IPA to be activated in CD8^+^XCR1^neg^ DCs. Genes involved in the TLR signaling pathway (adjusted *p*-value = 4.17e-02) and response to pathogen-associated molecular patterns (adjusted *p*-value = 3.31e-02) were also found to be over-represented amongst the DEGs (Figure 3D, Supplementary Table 6). Network analysis using IPA identified *E-cadherin* (*Cdh1*), the cDC2-specific transcription factor, *Irf4*, and pDC-specific transcription factor, *Spi-B*, as important hub genes within the network of differentially expressed genes. Increased expression of *Spi-B* in CD8^+^XCR1^neg^ DCs appeared to regulate the expression of many pDC-specific genes, including *SiglecH* and *Klra17*. Hub genes *Cdh1* and *Irf4* were positioned in the network upstream of *Tlr3, Tlr4, Tlr5*, and *Tlr7* which in turn interacted with the inflammatory cytokine *Il-12b* and immuno-regulatory ligands *Cd274* (PD-L1) and *PDCD1LG2* (PD-L2) (Figure 3E). These data provide further evidence of the unusual transcriptional profile of CD8^+^XCR1^neg^ DCs and indicate that differences in gene expression between CD8^+^XCR1^+^ and CD8^+^XCR1^neg^ DCs are highly relevant to their function.

### CD8^+^XCR1^neg^ DCs Express a Restricted Pattern of TLRs and a Unique Range of Endocytic Receptors Related to Both pDC and cDC2 Lineages

**Figure 4:**
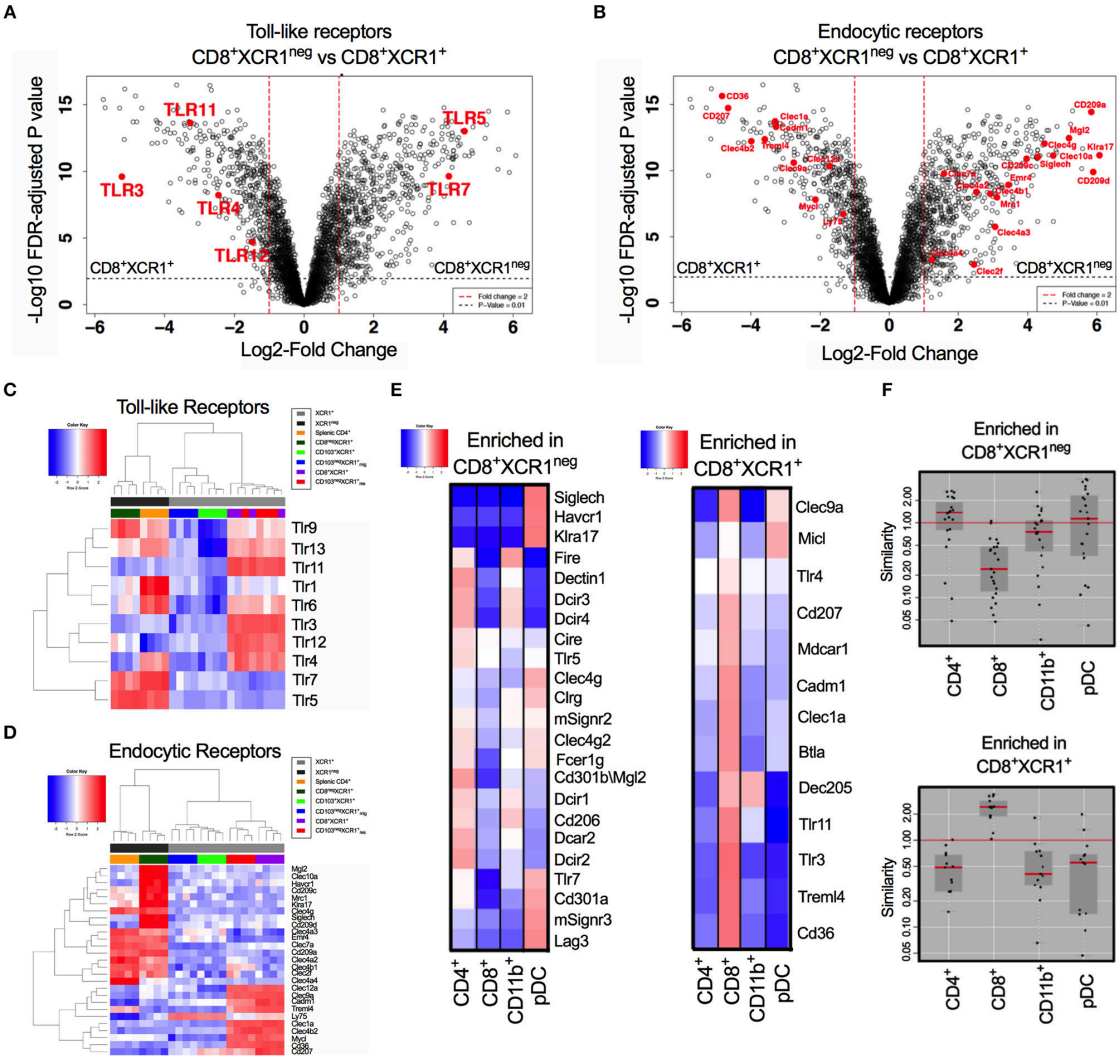
Differential expression of TLRs and endocytic receptors between CD8^+^XCR1^neg^ and CD8^+^XCR1^+^ DCs. The expression of TLRs and endocytic receptors was compared between CD8^+^XCR1^neg^ and CD8^+^XCR1^+^ DCs. Volcano plots show differential between CD8^+^XCR1^neg^ and CD8^+^XCR1^+^ DCs. Red dashed line represents log2FC>1 and black dashed line represents adjusted *p*-value < 0.01. TLRs **(A)** and endocytic receptors **(B)** are highlighted in red. Heatmaps of gene expression comparing expression of TLRs 1-13 **(C)** and endocytic receptors **(D)** across the DC subsets in the microarray. **(E)** TLRs and CLRs enriched in either CD8^+^XCR1^neg^ or CD8^+^XCR1^+^ DCs were compared to expression profiles of known DC subsets from the Immgen database using online tools. Red; up-regulated, Blue; down-regulated. **(F)** W-Plots summarize the relatedness of TLR and CLR profiles for CD8^+^XCR1^neg^ and CD8^+^XCR1^+^ DCs obtained from microarray data to specific Immgen populations.

CD8^+^XCR1^+^ and CD8^+^XCR1^neg^ DCs have distinct gene expression profiles. Our pathway analysis indicated that many DEGs were involved in TLR signaling and responses to pathogens. Therefore, we specifically compared the expression of genes encoding TLRs and endocytic receptors between these two DC subsets. We observed that TLR genes were among the most differentially expressed between CD8^+^XCR1^+^ and CD8^+^XCR1^neg^ DCs (Figure 4A). Endocytic receptors, including CLRs, were also highly differentially expressed (Figure 4B). Our microarray data demonstrate that resident XCR1^+^ DCs share a common TLR pattern with high expression of *Tlr3, Tlr4, Tlr11*, and *Tlr12*, but low expression of *Tlr5* and *Tlr7*. CD8^+^XCR1^neg^ DCs do not express *Tlr3* and instead share expression of *Tlr5, Tlr7*, and *Tlr9* in common with splenic CD4^+^ DCs, a major cDC2 subset. However, their lower expression of *Tlr1, Tlr4*, and *Tlr6* gives CD8^+^XCR1^neg^ DCs a more restricted TLR expression pattern compared to CD4^+^ DCs (Figure 4C). Examining the expression of endocytic receptors we observed that CD8^+^XCR1^neg^ DCs expressed many CLRs distinct from those expressed by CD8^+^XCR1^+^ DCs and exhibited up-regulated expression of several CLRs expressed by splenic CD4^+^ DCs or pDCs (52), but not seen in other DC subsets (Figure 4D). To investigate this further, we used expression data from the Immgen database (30) to interrogate the TLR and CLR expression of CD8^+^XCR1^+^ and CD8^+^XCR1^neg^ DCs compared to a panel of sdLN DC subsets, including pDCs and CD11b^+^ DCs. Receptors up-regulated in the CD8^+^XCR1^+^ subset, including *Tlr3, Treml4, Cd207*, and *CD36* were highly associated with the CD8^+^ Immgen subset, with high expression of CLRs *Micl* and *Clec9a* also shared with pDCs (Figure 4E). TLRs and CLRs up-regulated in CD8^+^XCR1^neg^ DCs showed a more varied pattern of association. Expression of *SiglecH, Tlr7, Cd301a, Havcr1*, and *Klra17* were restricted to the Immgen pDC subset. Other CLRs were shared with CD4^+^ and CD11b^+^ subsets but were absent from pDCs, including *Dectin1, Dcir1 Dcir2, Cd206*, and *Emr4*. Furthermore, several genes including *Tlr5, Cd209a*, and *CD301b* showed high expression only in the Immgen CD4^+^ subset (Figure 4E). In summary, the TLR and CLR expression pattern of CD8^+^XCR1^neg^ DCs is positively correlated to both CD4^+^ DCs and pDCs, but not the classical CD8^+^ DC population (Figure 4F). These data suggest a unique role for CD8^+^XCR1^neg^ DCs in the sensing of pathogens during immunosurveillance and suggests they play a specific role in the division of labor within the DC network.

### RT-PCR and Flow Cytometry Confirms TLR5 Expression on CD8^+^XCR1^neg^ DCs

**Figure 5:**
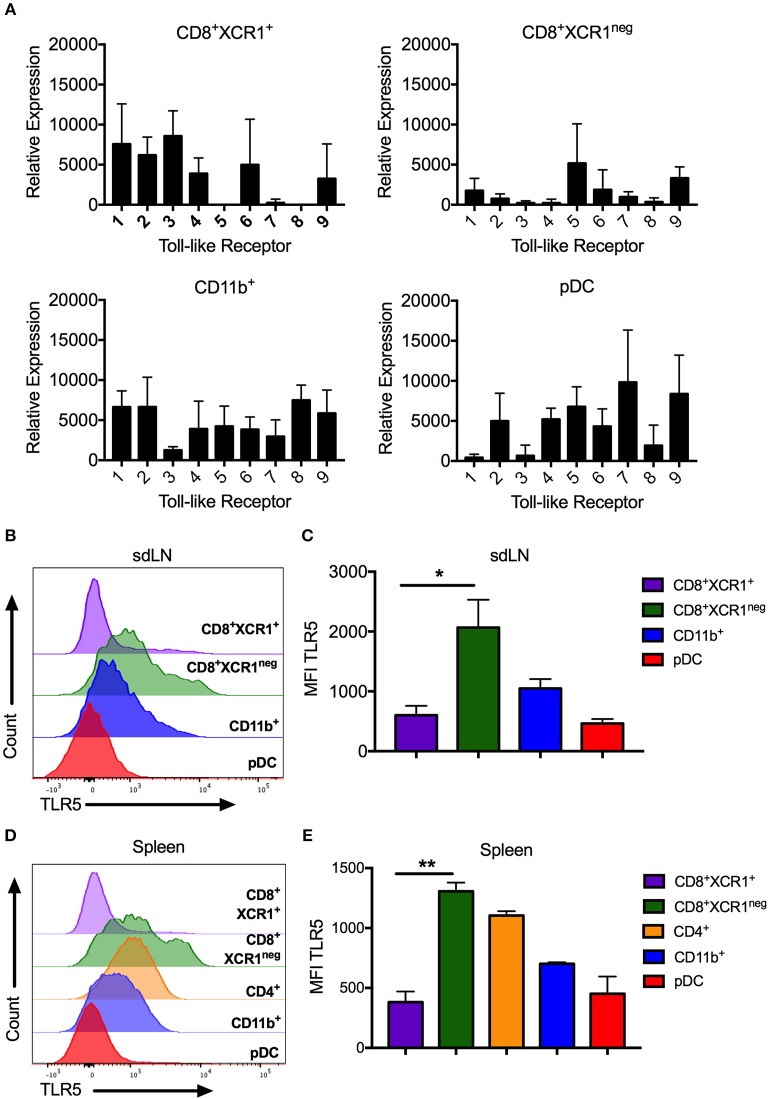
CD8^+^XCR1^neg^ DCs can be differentiated from CD8^+^XCR1^+^ DCs by TLR5 mRNA and cell surface expression. DC subsets were isolated from the sdLN and spleen and their TLR expression was analyzed by RT-PCR or flow cytometry **(A)** RT-PCR analysis of DC subsets sorted from the sdLNs (Supplementary Figure 1A) and converted to cDNA for use as template in RT-PCR reactions with primers specific to TLRs 1-9. Data are presented as relative expression normalized to GAPDH, *n* = 3 and error bars represent SEM. DC subsets were isolated from sdLN **(B,C)** or spleen **(D,E)** and analyzed by flow cytometry for TLR5 expression. Representative histograms of 4 independent experiments are shown for sdLN **(B)** and spleen **(D)**. MFI of TLR5 expression on DC subsets from the sdLN **(C)** or spleen **(E)** presented as the mean ± the SEM. ^*^p < 0.05, ^**^p < 0.01, *n* = 4.

High levels of *Tlr5* expression by CD8^+^XCR1^neg^ DCs clearly demarcates them from classical CD8^+^XCR1^+^ DCs. We confirmed the TLR expression patterns observed in our microarray data by both RT-PCR and flow cytometry, after sorting DC subsets from the sdLN and spleen (Supplementary Figures 1A,B). CD8^+^XCR1^neg^ DCs from sdLNs had high levels of *Tlr5* mRNA and expressed low levels of other TLRs. *Tlr5* expression was also detected in CD4^+^ DCs and pDCs, which expressed mRNA for most TLRs to moderate levels (Figure 5A). In comparison, CD8^+^XCR1^+^ DCs in the sdLN and spleen consistently expressed *Tlr1-4, Tlr6*, and *Tlr9* but lacked expression of *Tlr5, Tlr7*, and *Tlr8*. Importantly, expression of *Tlr3* was highly selective for CD8^+^XCR1^+^ DCs as has been reported previously (38) (Figure 5A, Supplementary Figure 3A). As expression of *Tlr3, Tlr5* and *Tlr7* are reported to be restricted to individual subsets of DCs (38), their expression was further validated by flow cytometry. These data confirm that CD8^+^XCR1^neg^ DCs possess the highest surface expression of TLR5 among resident sdLN (Figure 5B) and splenic DC subsets (Figure 5D) and this expression was significantly higher than was seen on CD8^+^XCR1^+^ DCs (Figures 5C,E). Expression of TLR3 was largely restricted to CD8^+^XCR1^+^ DCs, while TLR7 expression was restricted to pDCs in the sdLN, with some expression also observed on CD4^+^ and CD8^+^XCR1^neg^ DCs in the spleen (Supplementary Figures 3B,C) after intracellular cytokine staining. Therefore, CD8^+^XCR1^neg^ DCs, expressing high levels of TLR5, are uniquely positioned to detect and respond to flagellated bacterial pathogens unlike CD8^+^XCR1^+^ DCs which uniquely express TLR3.

### CD8+XCR1^neg^ DCs Do Not Produce Type I IFN After Stimulation With the TLR5 Ligand Flagellin

**Figure 6:**
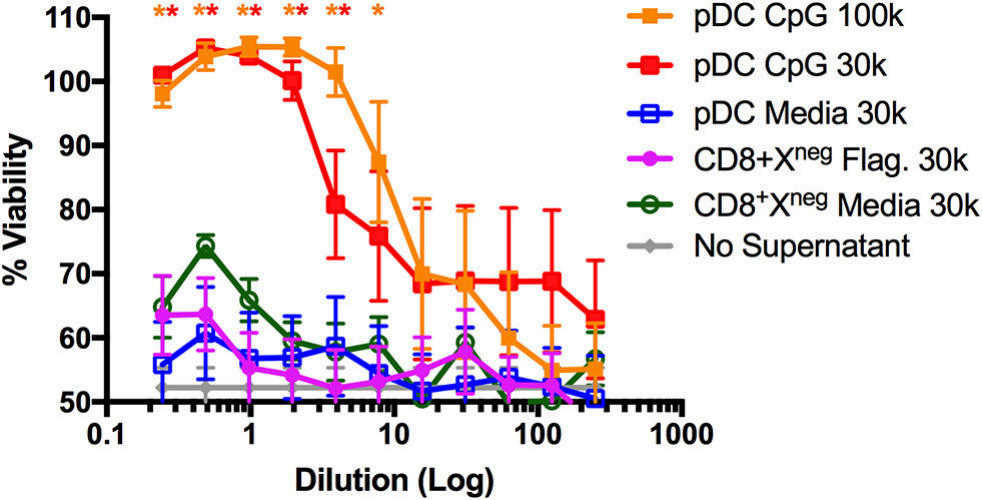
CD8^+^XCR1^neg^ DCs do not produce Type I IFNs after stimulation with the TLR5 agonist flagellin. DC subsets sorted from the spleens of naïve C57BL/6 mice (Supplementary Figure 1B) were cultured overnight in the presence of TLR agonists Flagellin (Flag.) (100ng/uL), CpG (1 μg/mL) or in media alone at a concentration of 30,000–100,000 DCs/well. After 24 h supernatants were collected, acid treated, serially diluted onto L929 cells and incubated overnight prior to addition of EMCV. After 2 days cells were stained with crystal violet, washed and absorbance was measured at 595 nM. Bioactive IFN production was measured as protection from viral killing compared to no virus control wells. Data are presented as the mean ± SEM of three independent experiments, *n* = 6. ^**^ represents *p* < 0.01 and ^*^ represent *p* < 0.05 vs. unstimulated pDC control well. 100 k = 100,000 DC/well, 30 k = 30,000 DC/well.

The pDC-like gene signature of CD8^+^XCR1^neg^ DCs prompted us to examine their ability to make type I IFN. It was reported previously that this subset does not produce type I IFN in response to viral challenge (50), however, stimulation with bacterial flagellin, the TLR5 agonist, has not been investigated. To determine the capacity of CD8^+^XCR1^neg^ DCs to produce type I IFN after TLR stimulation, we sorted DC subsets from the spleens of naïve C57BL/6 mice (Supplementary Figure 1B) and stimulated them for 24 h with either the TLR4 agonist LPS, TLR5 agonist flagellin or TLR9 agonist CpG. Culture supernatants were then assayed for active type I IFN levels using a viral protection bioassay. We found that CpG-stimulated pDCs produced adequate amounts of type I IFN to prevent viral killing of L929 cells. However, similar protection was not observed with supernatants from flagellin- or CpG-stimulated CD8^+^XCR1^neg^ DCs even at the highest concentrations (Figure 6). Cell death in these wells was similar to media only controls (no supernatant) which displayed no anti-viral effect. This type I IFN bioassay is a sensitive method of measuring bioactive type I IFN production by DCs (53), therefore this result strongly suggests that CD8^+^XCR1^neg^ DCs do not produce type I IFN after activation via TLR5. These data indicate that CD8^+^XCR1^neg^ DCs can be separated from pDCs on a functional level despite similarities in their transcriptional profile. We sought to gain further insight into the functional role of CD8^+^XCR1^neg^ DCs by assessing their cytokine production after TLR stimulation using a multiplex Luminex assay. Interestingly, we detected lower levels of cytokine production after stimulating CD8^+^XCR1^neg^ with flagellin (Supplementary Figure 4A) as compared to CD4^+^ DCs, and observed a reduced response to CpG stimulation compared to other DC subsets (Supplementary Figure 4B). These data suggest that the capability of CD8^+^XCR1^neg^ DCs to drive CD4^+^ T cell proliferation differs to that of cDC2 DC subsets as it is not dependant on the production of inflammatory cytokines.

### CD8^+^XCR1^neg^ DCs Resemble cDC2 in Antigen Presentation Ability and CLR Expression

**Figure 7:**
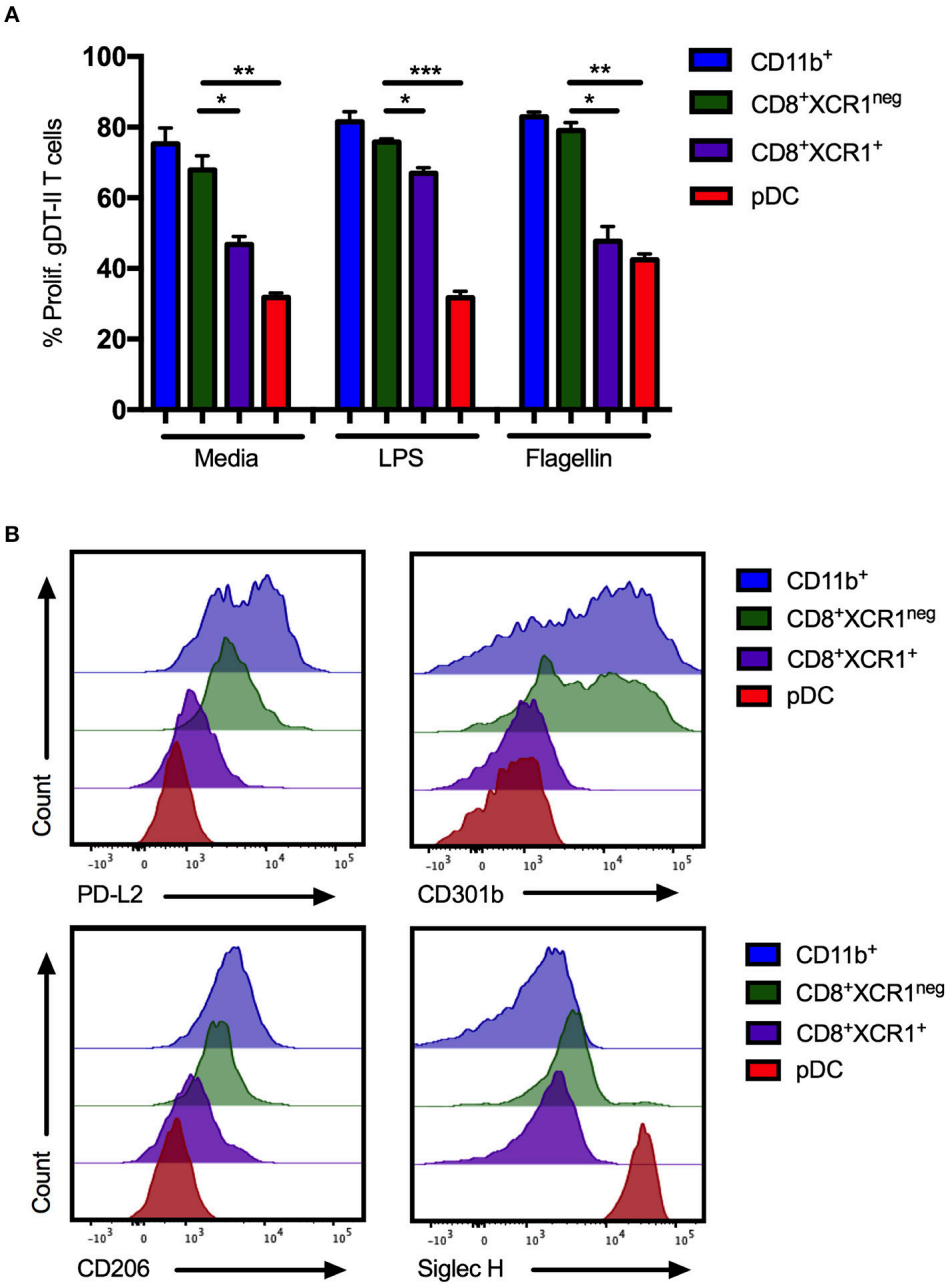
CD8^+^XCR1^neg^ DCs resemble cDC2 DCs in MHC class II presentation ability and CLR expression in the sdLN. sdLN DCs were sorted as in Supplementary Figure 1A. **(A)** DC subsets were activated for 24 h *in vitro* with TLR agonists LPS (100 ng/mL) or Flagellin (1 μg/mL) or left unstimulated. Activated DCs were pulsed with 1 nM gD peptide for 1 h and cultured for 4 days with naïve CFSE-labeled gDT-II at a 1:10 ratio. Proliferation of gDT-II cells was measured by CFSE dilution. Data are presented as the percentage of CFSE low (proliferated) gDT-II cells and error bars show mean ± SEM from two independent experiments, *n* = 4, ^*^p > 0.05, ^**^p > 0.01, ^***^p > 0.001. **(B)** Expression of PD-L2 and CLRs, CD206, CD301b, and Siglec-H on DC subsets isolated from sdLNs of naïve C57Bl/6 mice. Representative plots of 3 independent experiments are shown.

pDCs are reported to be poor presenters of antigen to CD4^+^ T cells due to continous recycling of major histocompatability complex (MHC) class II molecules (54). To determine if CD8^+^XCR1^neg^ DCs share this characteristic, DC subsets were sorted from the sdLNs and spleens of naïve mice (Supplementary Figures 1A,B), stimulated with the TLR ligands LPS or flagellin and pulsed with gD-peptide before co-culture with gD-specific transgenic CD4 T cells (gDT-II) (6). CD11b^+^ DCs, CD4^+^ DCs and CD8^+^XCR1^neg^ DCs were able to efficiently present antigen and drove proliferation of gDT-IIs to similar levels. gDT-IIs proliferated significantly less when co-cultured with CD8^+^XCR1^+^ DCs or pDCs (Figure 7A; Supplementary Figure 5A). CD8^+^XCR1^neg^ DCs express moderate levels of MHC class II (42, 51) and the results of our functional experiments indicate they are capable of presenting antigen efficiently to CD4^+^ T cells, further separating them functionally from pDCs. We next examined the surface expression of several CLRs, which were upregulated on CD8^+^XCR1^neg^ DCs in our microarray data, on sorted DC subsets. CD8^+^XCR1^neg^ DCs shared high expression of CD301b with CD11b^+^ DCs in the sdLN and expressed increased levels of PD-L2 and CD206 compared to CD8^+^XCR1^+^ DCs and pDCs (Figure 7B). Interestingly, although our microarray data suggested that CD8^+^XCR1^neg^ DCs had up-regulated expression of the pDC-specific gene *Siglech*, we were unable to detect Siglec H expression on these DCs by flow cytometry. pDCs were the only population in the sdLN or spleen to stain brightly for Siglec H (Figure 7B; Supplementary Figure 5B). Expression of PD-L2, CD301b, and CD206 were consistently low across all DC subsets isolated from the spleen (Supplementary Figure 5B), suggesting there may be differences in CLR expression based on tissue of origin. Our phenotyping data further strengthens the link between CD8^+^XCR1^neg^ DCs and cDC2 subsets in the sdLN. Together these findings further differentiate CD8^+^XCR1^neg^ DCs from CD8^+^XCR1^+^ DCs and pDCs both functionally and phenotypically, and suggest they occupy a similar role to cDC2 subsets.

## Discussion

The classical T cell markers CD4 and CD8 have historically been used to classify murine DC subsets with differing functions and ontogeny (55). CD8^+^ DCs were intially described as cross-presenting cDC1 with their development controlled by the transcription factor IRF8. The more heterogeneous cDC2 subset was first described in the spleen as being CD4^+^, reliant on the transcription factor IRF4 and better equipped to present antigen on MHC class II (56). However, the use of CD4 and CD8 to describe DC subsets has been described as unfortunate, due to their lack of specificity and functional role in DC biology and furthermore, because they are not conserved across species (57). CD4^+^ DCs are the major population of cDC2 in the spleen, however they are less well represented among cDC2 subsets in the lymph nodes, which contain a mix of resident and migratory DCs (55). Similarly, migratory cDC1 are commonly identified by their expression of CD103 rather than CD8, which they express to a lesser degree than their LN-resident counterparts (4). More recent work has shown that not all CD8^+^ or CD103^+^ DCs possess the ability to cross-present antigen (52) instead, expression of the receptor XCR1 uniformly identifies cross-presenting DCs in both mice and humans (35). Alongside the two classical cross-presenting populations that are CD8^+^XCR1^+^ and CD103^+^XCR1^+^ we have previously identified populations of LN-resident CD8^−^XCR1^+^ DCs and migratory CD103^−^XCR1^+^ DCs that are able to cross-present tumor antigen (38). These data call into question the usefulness of traditional DC surface markers to robustly identify unique subsets of DCs.

A focus of our current study was to identify whether differences exist in the transcriptomes of these four XCR1^+^ subsets (42), to determine if they possess unique functions or occupy specific niches within the DC immunosurveillance network. Examining our microarray data we observed few gross differences in the expression patterns between XCR1^+^ subsets, apart from those related to their migratory or resident origin. We did, however, observe a striking difference in the transcriptional profiles of CD8^+^XCR1^+^ and CD8^+^XCR1^neg^ DCs. Despite their expression of CD8 and CD24, both classical cDC1 markers, CD8^+^XCR1^neg^ DCs do not cross-present antigen and co-express high levels of the cDC2-specific surface markers SIRPa and CX_3_CR1 (51). Further supporting their position within the cDC2 lineage, these DCs have increased levels of IRF4 compared to CD8^+^XCR1^+^ DCs (42). Curiously, it has been reported previously that a CX_3_CR1^+^ subset of CD8^+^ DCs, which likely overlaps with the CD8^+^XCR1^neg^ population, possess a mixed ontogeny. These DCs display upregulation of key cDC2-specific transcription factors and are reliant on expression of the pDC-specific factor E2-2 for their generation (50). The results from our study further corroborate the findings of Bar-On and colleagues, demonstrating an enrichment of pDC-specific and CD8^neg^ DC-specific genes (31) within the transcriptome of CD8^+^XCR1^neg^ DCs. Despite this population being well-represented across the secondary lymphoid compartment, to date only a handful of studies have included them in their analyses (42, 50, 51). Thus, their role within the complex network of DC surveillance remains to be elucidated.

Considering CD8^+^XCR1^neg^ DCs display a hybrid cDC2/pDC ontogeny we delved deeper into their expression of key sensory receptors, such as TLR and CLR, which modulate the way DCs interact with their environment. We discovered that CD8^+^XCR1^neg^ DCs expressed many pDC- and cDC2-specific endocytic receptors including a unique array of CLRs and TLRs not expressed on cDC1. The expression of specific TLRs can be used to classify DC subsets in mice (34) and humans (58). Cross-presenting murine cDC1 and human CD141^+^ DCs detect viral RNA via expression of TLR3 (11, 59), while pDCs universally express high levels of TLR7 and respond to viral stimulus by producing copious quantities of type I IFN (60). Our transcriptomic and phenotypic data confirm that CD8^+^XCR1^neg^ DCs lacked the TLR profile to efficiently respond to viral challenge. Instead they display increased expression of TLR5, the receptor for the bacterial flagellin protein (61), which is expressed mainly by CD11b^+^ and CD103^+^CD11b^+^ cDC2 (62). We also identified that CD8^+^XCR1^neg^ DCs express a unique profile of endocytic receptors, with pDC-specific CLRs such as Klra17 (Ly49Q) (63) and Siglec H (21) and cDC2-related CLRs including Emr4, Mgl2, mannose receptor and DC-SIGN all up-regulated compared to CD8^+^XCR1^+^ DCs. These data suggest that CD8^+^XCR1^neg^ DCs possess the sensory machinery to respond to a broad range of stimuli giving them a unique position in the DC surveillance network that differs to that of CD8^+^XCR1^+^ DCs.

The unique pDC/cDC2 phenotype of CD8^+^XCR1^neg^ DCs is also reflected in their expression of key DC lineage transcription factors. Our data identifies that they have increased levels of IRF4, E2-2 and SpiB compared to CD8^+^XCR1^+^ DCs. These results mirror the report by Bar-On and colleagues who reported that CX_3_CR1^+^CD8^+^ DCs have an expression profile more closely related to CD8^neg^ DCs than CD8^+^ DCs and are dependent on E2-2 for their generation (50). More recently, it was reported that E2-2^hi^ DC progenitors could differentiate into both pDCs or cDC dependent on the specific cytokines present in their microenvironment (64). These findings indicate that the function of CD8^+^XCR1^neg^ DCs may be fine-tuned by the cytokine milieu of their niche. Even though these DCs possess a transcriptome enriched for pDC signature genes (31) they fail to make type I IFN after exposure to viral (50) or bacterial compounds, suggesting they lack bonafide pDC functionality. Instead, CD8^+^XCR1^neg^ DCs are more efficient at driving CD4^+^ T cell proliferation than either CD8^+^XCR1^+^ DCs or pDCs supporting a functional link to the cDC2 lineage. With their adundant expression of TLR5 CD8^+^XCR1^neg^ DCs may instead be poised to influence the phenotype of the CD4^+^ T cell response during bacterial infection similar to CD11b^+^CD103^+^ DCs, which drive Th17 responses after TLR5 ligation via production of IL-6 (18) and IL-23 (65).

CD8^+^XCR1^neg^ DCs display a unique transcriptional profile, providing a clear distinction from the CD8^+^XCR1^+^ subset. Their core ontogeny and functional responses show no correlation to those relating to classical cDC1, which are still often defined by their CD8 expression. Our findings draw into question whether CD8 is a reliable marker for identifying cross-presenting DC subsets as CD8^+^XCR1^neg^ DCs, which do not cross-present antigen, exist throughout the secondary lymphoid compartment. Importantly, although these CD8^+^XCR1^neg^ DCs display a pDC-like transcriptional profile they share many sensory mechanisms with cDC2 subsets, in particular expression of TLR5. Functionally these DCs fail to make type I IFN after TLR stimulation, the classical test of pDC function, and are capable of efficiently presenting antigen to CD4^+^ T cells. This divergent population makes up a significant percentage of DCs in the secondary lymphoid organs and therefore warrants additional study to identify their role in the generation of immunity.

## Datasets Are in a Publicly Accessible Repository

The datasets generated for this study can be found in the Gene Expression Omnibus (GEO) https://www.ncbi.nlm.nih.gov/geo/.

## Data Has Been Obtained From a Third Party

The data used for comparison in Figures 2B–D and Supplementary Figure 2. were published by Miller et al. (31). The data used for comparison in Figures 4E,F was obtained from The Immunological Genome Project. This data is publicy available at https://www.immgen.org/.

## Author Contributions

BW and JW designed the experiments. BW, JR, ACB, TW, NT, and GS performed experiments or analyzed data. MC and AB performed microarray QC and analysis. JW, BW, MC, and BF were involved in data discussion and drafting the manuscript. BW wrote the manuscript. JW, MC, BF, and SS edited the manuscript.

### Conflict of Interest Statement

The authors declare that the research was conducted in the absence of any commercial or financial relationships that could be construed as a potential conflict of interest.

## Supplementary Material

Supplementary Figure 1 Gating strategy used to identify and sort DC subsets. Gating strategy to identify DC subsets for sorting from the sdLNs **(A)** and spleen **(B)** for use in RT-PCR, flow cytometry and *ex-vivo* proliferation experiments.

Supplementary Figure 2 Comparison of DC gene expression to a CD8^neg^ DC gene signature. Gene expression data from microarray analysis of sorted DC subsets were compared to a panel of 16 CD8^neg^ DC signature genes (27). Red; upregulated, Blue; down-regulated. Populations have been clustered hierarchically. Heatmaps are colored coded according to the legend.

Supplementary Figure 3 TLR validation on sdLN and splenic DC subsets. **(A)** RT-PCR analysis of sorted splenic DC subsets (Supplementary Figure 1B) was converted to cDNA and used as template in RT-PCR reactions with primers specific to TLRs 1-9. Data are presented as relative expression normalized to GAPDH control reactions, *n* = 3 and error bars represent SEM. **(B)** DC subsets from the sdLN (Supplementary Figure 1A) were analyzed by intracellular cytokine staining for expression of TLR3 and TLR7. Representative histograms of 3 independent experiments are shown. **(C)** DC subsets from the spleen (Supplementary Figure 1B) were analyzed by intracellular cytokine staining for expression of TLR3 and TLR7. Representative histograms of 3 independent experiments are shown.

Supplementary Figure 4 Cytokine expression by CD8^+^XCR1^neg^ DCs following TLR stimulation. Splenic DCs were sorted as in Supplementary Figure 1B. **(A)** CD4^+^ DCs and CD8^+^XCR1^neg^ DCs were stimulated with flagellin (1 μg/mL) or left unstimulated. After 36 h culture supernatants were collected and used in Luminex assays at a 1:2 dilution to assay for inflammatory cytokines IL-12p40, CCL5, MIP-1a, and MIP-1b as indicator of an inflammatory response. **(B)** CD8^+^XCR1^+^ DCs, CD8^+^XCR1^neg^ DCs, CD4^+^ DCs, and pDCs were stimulated with CpG (2 μM) or left unstimulated. After 36 h culture supernatants were collected and production of cytokines IL-6, IL-12p40, and IL-12p70 was measured using a Luminex assay. Data are presented in pg/mL and are from two independent experiments run in duplicate on a single assay plate. Error bars represent the mean ± SEM.

Supplementary Figure 5 Splenic CD8^+^XCR1^neg^ DCs resemble cDC2 DCs in MHC class II presentation ability and CLR expression. Splenic DCs were sorted as in Supplementary Figure 1B. **(A)** DC subsets were activated for 24 h *in vitro* with TLR agonists LPS (100 ng/mL) or flagellin (1 μg/mL) or left unstimulated. Activated DCs were pulsed with 1 nM gD peptide for 1 h and cultured for 4 days with naïve CFSE-labeled gDT-IIs at a 1:10 ratio. Proliferation of gDT-II cells was measured by CFSE dilution. Data are presented as the percentage of CFSE low (proliferated) gDT-II cells and error bars show mean ± SEM from two independent experiments, *n* = 4. ^*^*p* > 0.05, ^**^*p* > 0.01. **(B)**. Expression of PD-L2 and CLRs, CD206, CD301b and Siglec H on DC subsets isolated from spleens of naïve C57Bl/6 mice. Representative plots of 3 independent experiments are shown.

Supplementary Table 1 Differentially expressed genes between CD103^−^XCR1^+^MHCIIhi and CD103^+^XCR1^+^DC in the sdLNs. Differentially expressed genes between CD103^−^XCR1^+^MHCIIhi and CD103^+^XCR1^+^ DC subsets. The data are derived from TopTable analysis with an FDR < 0.05.

Supplementary Table 2 Differentially expressed genes between CD103^−^XCR1^+^MHCIIlow and CD8^+^XCR1^+^ DC in the sdLNs. Differentially expressed genes between CD103^−^XCR1^+^MHCIIlow and CD8^+^XCR1^+^ DC subsets. The data are derived from TopTable analysis with an FDR < 0.05.

Supplementary Table 3 Differentially expressed genes between CD8^+^XCR1^neg^ and CD8^+^XCR1^+^ DC in the sdLNs. Differentially expressed genes between CD8^+^XCR1^+^ and CD8^+^XCR1^neg^ DC subsets. The data are derived from TopTable analysis with an FDR < 0.05.

Supplementary Table 4 Genes used in cDC, CD8^+^ DC and pDC gene signatures. Gene sets with specific expression in cDC, CD8^+^DC or pDC subsets were compiled using data from Miller et al. (31). Subset specific signature genes were used to create heatmaps of differential gene expression across DC subsets in the microarray.

Supplementary Table 5 Gene set enrichment analysis of genes upregulated in CD8^+^XCR1^neg^ vs. CD8^+^XCR1^+^ DC. Gene set erichment analysis was carried out on genes upregulated with a FC >2 and adj. *P*–value < 0.05 (*n* = 601) using Enrichr to query.

Supplementary Table 6 Ingenuity Pathway Analysis of differentially expressed genes between CD8^+^XCR1^neg^ and CD8^+^XCR1^+^DC. Pathway and network analysis was carried out using Ingenuity pathway analysis with genes up– or down–regulated with a FC >5 and adj. *P* < 0.05.

Supplementary Table 7 Differentially expressed genes between CD8^+^XCR1^neg^ and Splenic CD4^+^ DCs. Differentially expressed genes between CD8^+^XCR1^neg^ and Splenic CD4^+^DC subsets. The data are derived from Top Table analysis with FDR < 0.05.
