# Supplementary material for: CD8+XCR1neg Dendritic Cells Express High Levels of Toll-Like Receptor 5 and a Unique Complement of Endocytic Receptors
Source: Front Immunol. 2019 Jan 16;9:2990. doi: 10.3389/fimmu.2018.02990 (PMC6343586; doi:10.3389/fimmu.2018.02990)
Supplement: Supplementary file 1 [file Data_Sheet_1.DOCX]

**Supplementary Methods.**

**Primers used for RT-PCR analysis of TLR expression:**

TLR1 forward 5’-GGATGTGTCCGTCAGCACTA-3’

TLR1 reverse 5’-TGTAACTTTGGGGGAAGCTG-3’

TLR2 forward 5’-CAGACGTAGTGAGCGAGCTG-3’

TLR2 reverse 5’-GGCATCGGATGAAAAGTGTT-3’

TLR3 forward 5’-GAGGGCTGGAGGATCTCTTT-3’

TLR3 reverse 5’-TGCCTCAATAGCTTGCTGAA-3’

TLR4 forward 5’-GCTTTCACCTCTGCCTTCAC-3’

TLR4 reverse 5’-CGAGGCTTTTCCATCCAATA-3’

TLR5 forward 5’-GCTTTGTTTTCTTCGCTTCG-3’

TLR5 reverse 5’-ACACCAGCTTCTGGATGGTC-3’

TLR6 forward 5’-GCAACATGAGCCAAGACAGA-3’

TLR6 reverse 5’-GTTTTGCAACCGATTGTGTG-3’

TLR7 forward 5’-ATTCAGAGGCTCCTGGATGA-3’

TLR7 reverse 5’-AGGGATGTCCTAGGTGGTGA-3’

TLR8 forward 5’-TCCTGGGGATCAAAAATCAA-3’

TLR8 reverse 5’-AAGGTGGTAGCGCAGTTCAT-3’

TLR9 forward 5’-ACCCTGGTGTGGAACATCAT-3’

TLR9 reverse 5’-GTTGGACAGGTGGACGAAGT-3’
