# Supplementary figures and images for: CD8+XCR1neg Dendritic Cells Express High Levels of Toll-Like Receptor 5 and a Unique Complement of Endocytic Receptors
Source: Front Immunol. 2019 Jan 16;9:2990. doi: 10.3389/fimmu.2018.02990 (PMC6343586; doi:10.3389/fimmu.2018.02990)

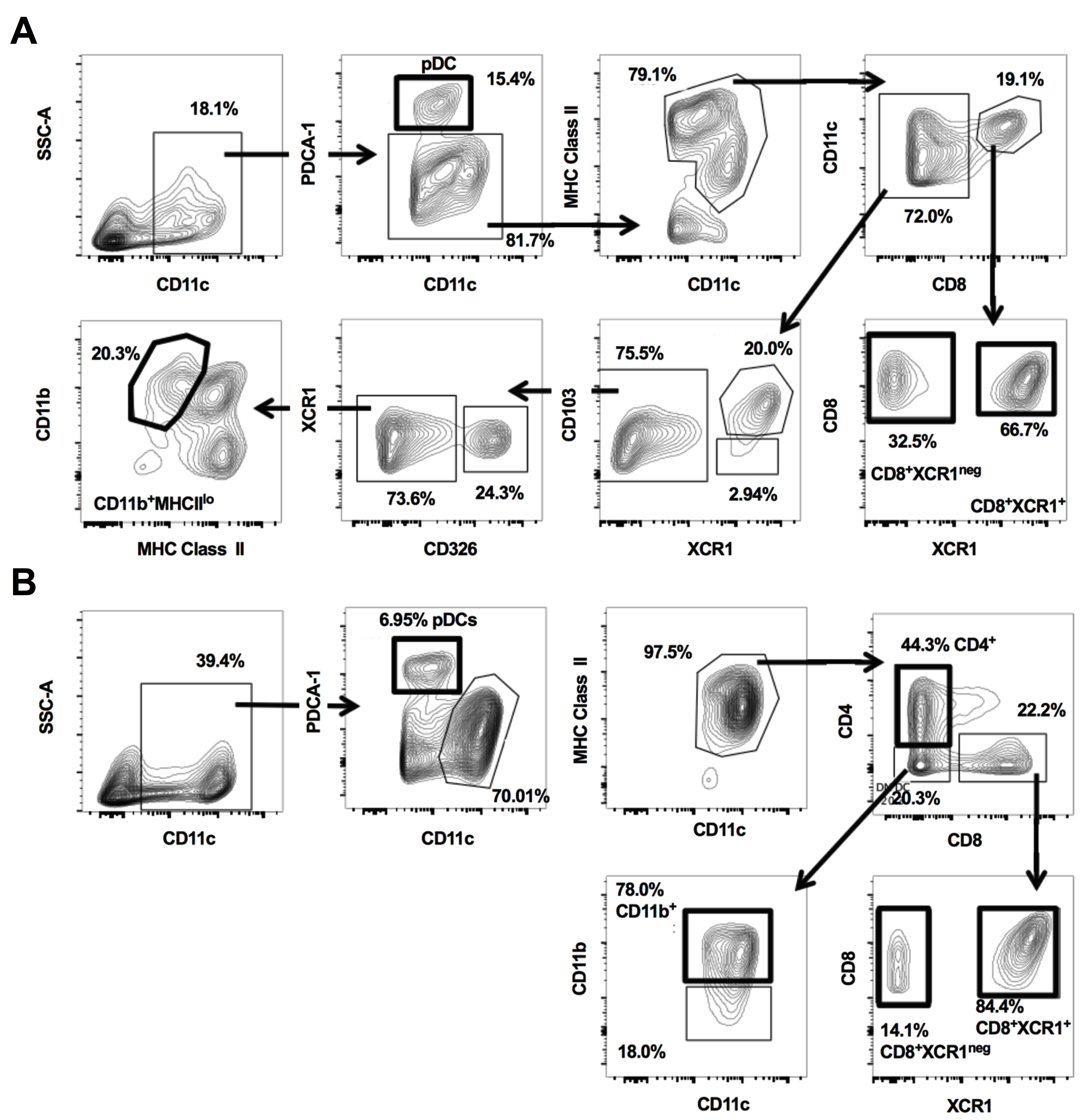

Supplement: Supplementary Figure 1 — Gating strategy used to identify and sort DC subsets. Gating strategy to identify DC subsets for sorting from the sdLNs (A) and spleen (B) for use in RT-PCR, flow cytometry and ex-vivo proliferation experiments. [file Image_1.TIFF]

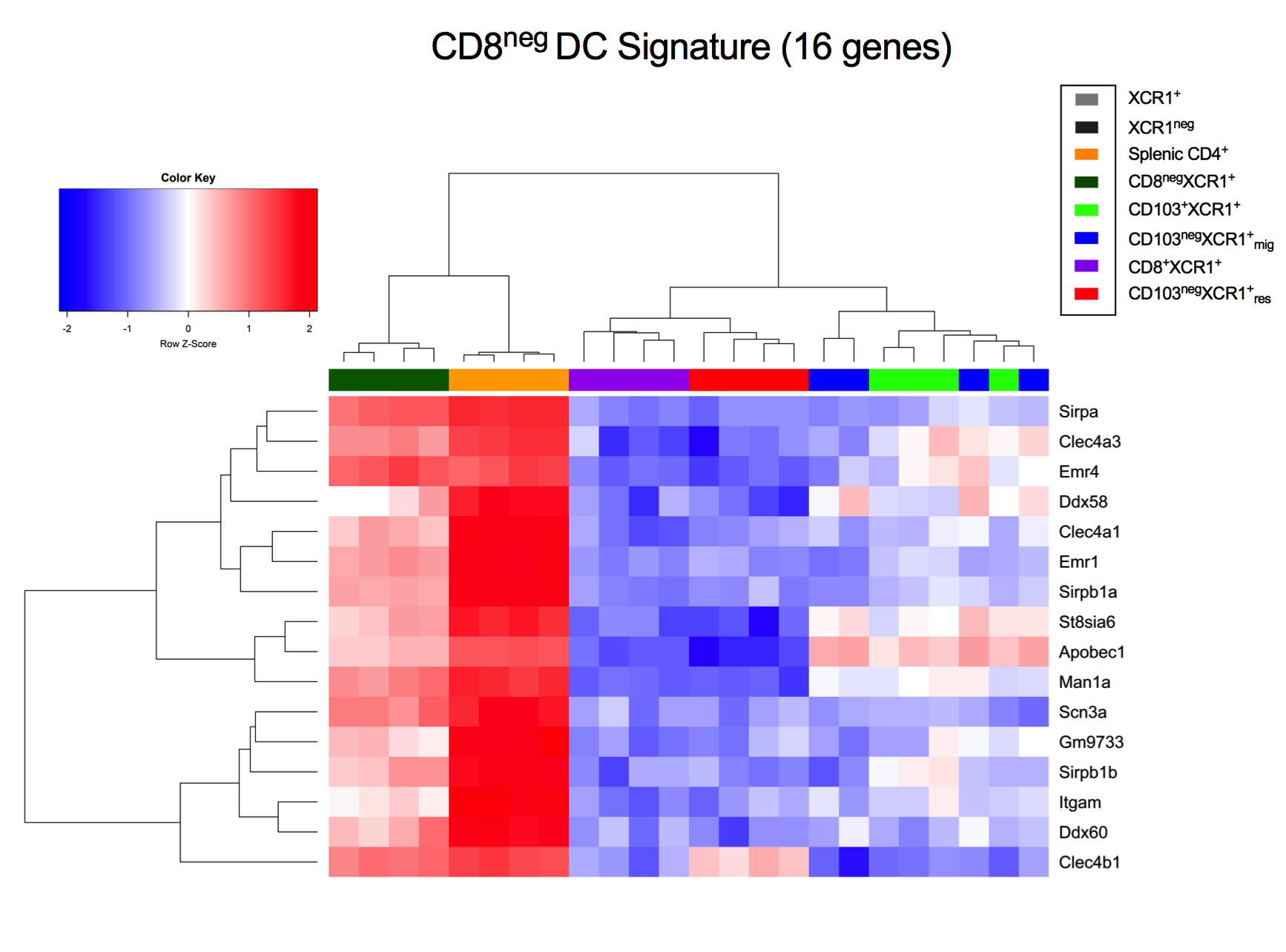

Supplement: Supplementary Figure 2 — Comparison of DC gene expression to a CD8neg DC gene signature. Gene expression data from microarray analysis of sorted DC subsets were compared to a panel of 16 CD8neg DC signature genes (27). Red; upregulated, Blue; down-regulated. Populations have been clustered hierarchically. Heatmaps are colored coded according to the legend. [file Image_2.TIFF]

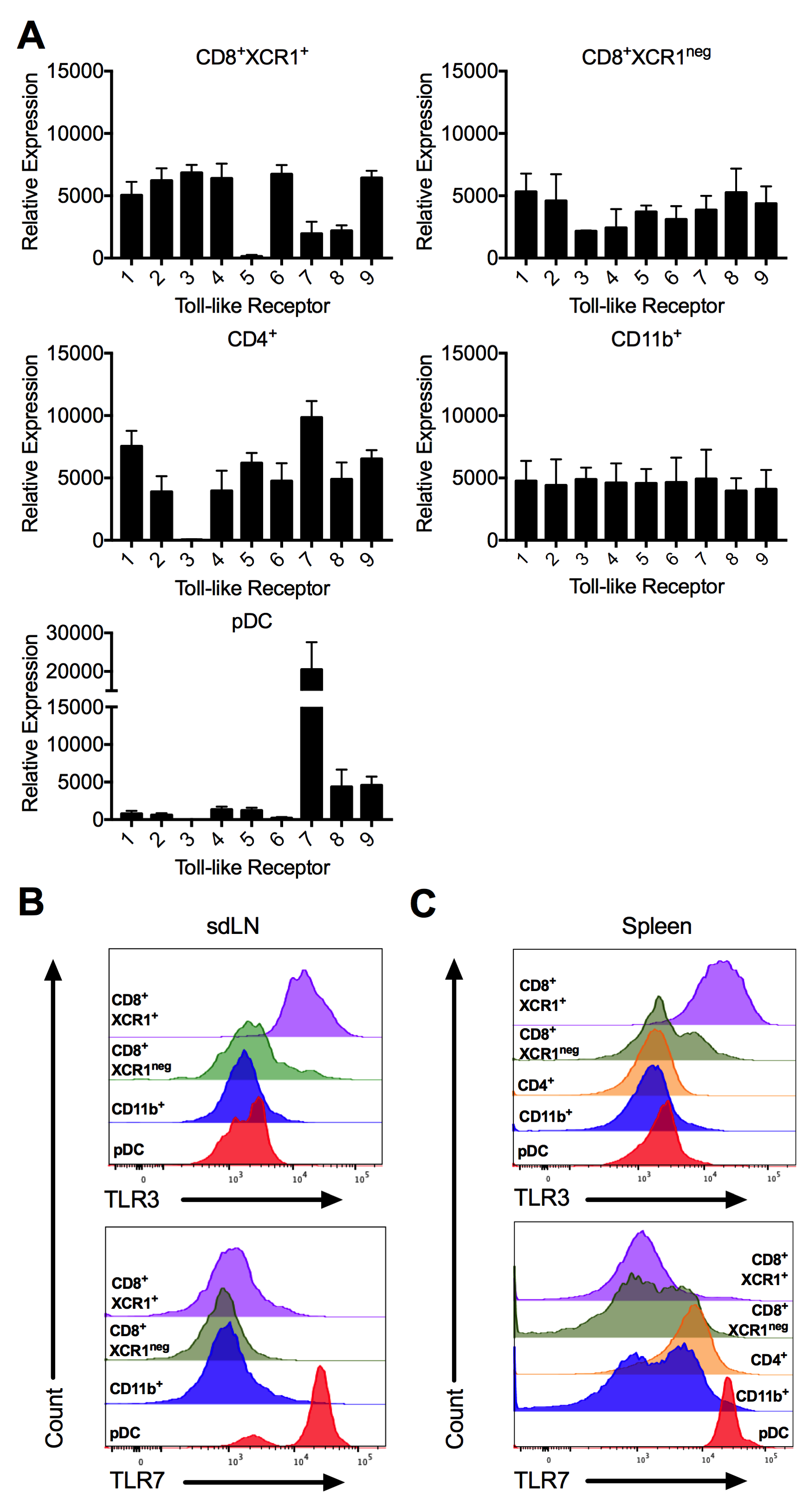

Supplement: Supplementary Figure 3 — TLR validation on sdLN and splenic DC subsets. (A) RT-PCR analysis of sorted splenic DC subsets (Supplementary Figure 1B) was converted to cDNA and used as template in RT-PCR reactions with primers specific to TLRs 1-9. Data are presented as relative expression normalized to GAPDH control reactions, n = 3 and error bars represent SEM. (B) DC subsets from the sdLN (Supplementary Figure 1A) were analyzed by intracellular cytokine staining for expression of TLR3 and TLR7. Representative histograms of 3 independent experiments are shown. (C) DC subsets from the spleen (Supplementary Figure 1B) were analyzed by intracellular cytokine staining for expression of TLR3 and TLR7. Representative histograms of 3 independent experiments are shown. [file Image_3.TIFF]

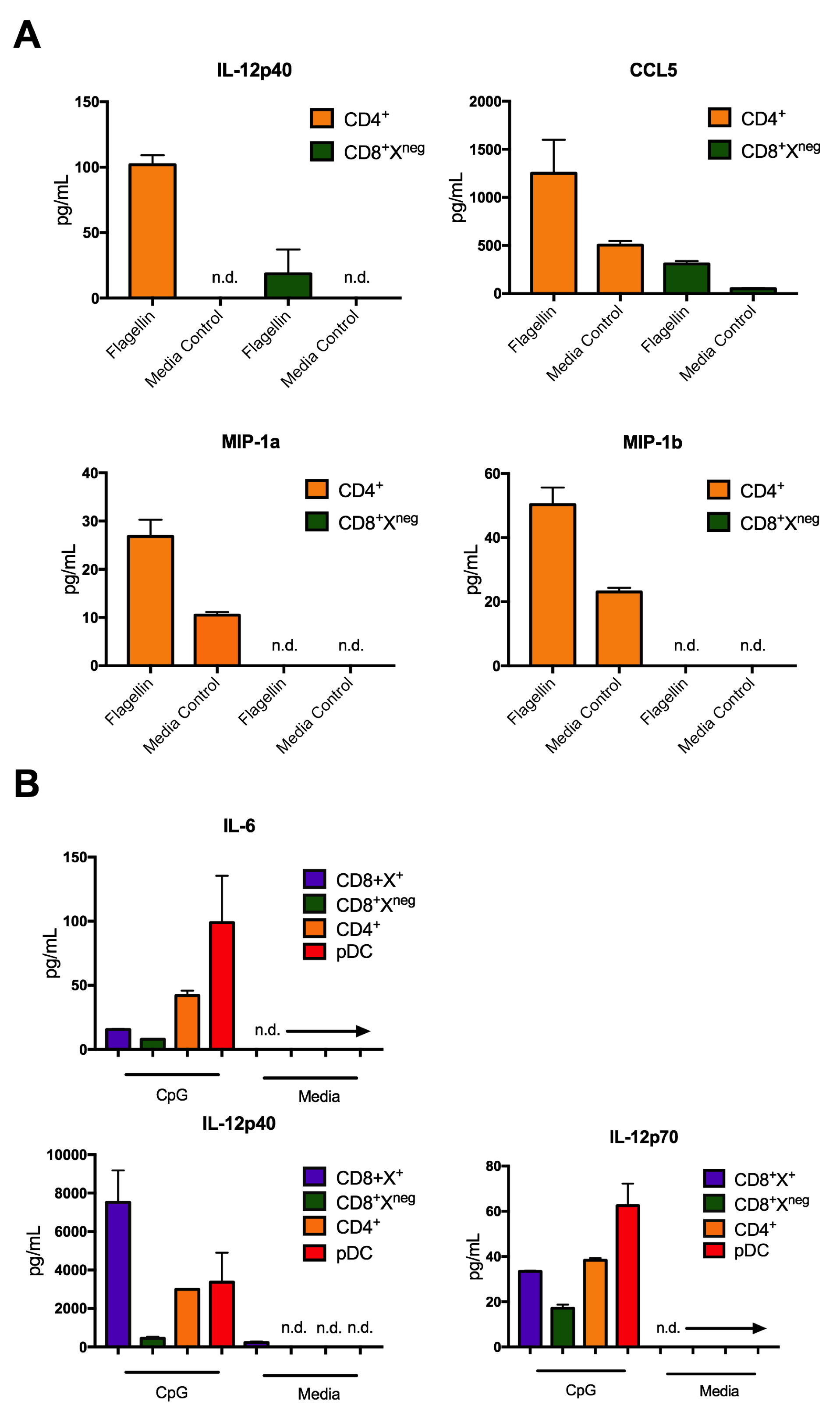

Supplement: Supplementary Figure 4 — Cytokine expression by CD8+XCR1neg DCs following TLR stimulation. Splenic DCs were sorted as in Supplementary Figure 1B. (A) CD4+ DCs and CD8+XCR1neg DCs were stimulated with flagellin (1 μg/mL) or left unstimulated. After 36 h culture supernatants were collected and used in Luminex assays at a 1:2 dilution to assay for inflammatory cytokines IL-12p40, CCL5, MIP-1a, and MIP-1b as indicator of an inflammatory response. (B) CD8+XCR1+ DCs, CD8+XCR1neg DCs, CD4+ DCs, and pDCs were stimulated with CpG (2 μM) or left unstimulated. After 36 h culture supernatants were collected and production of cytokines IL-6, IL-12p40, and IL-12p70 was measured using a Luminex assay. Data are presented in pg/mL and are from two independent experiments run in duplicate on a single assay plate. Error bars represent the mean ± SEM. [file Image_4.TIFF]

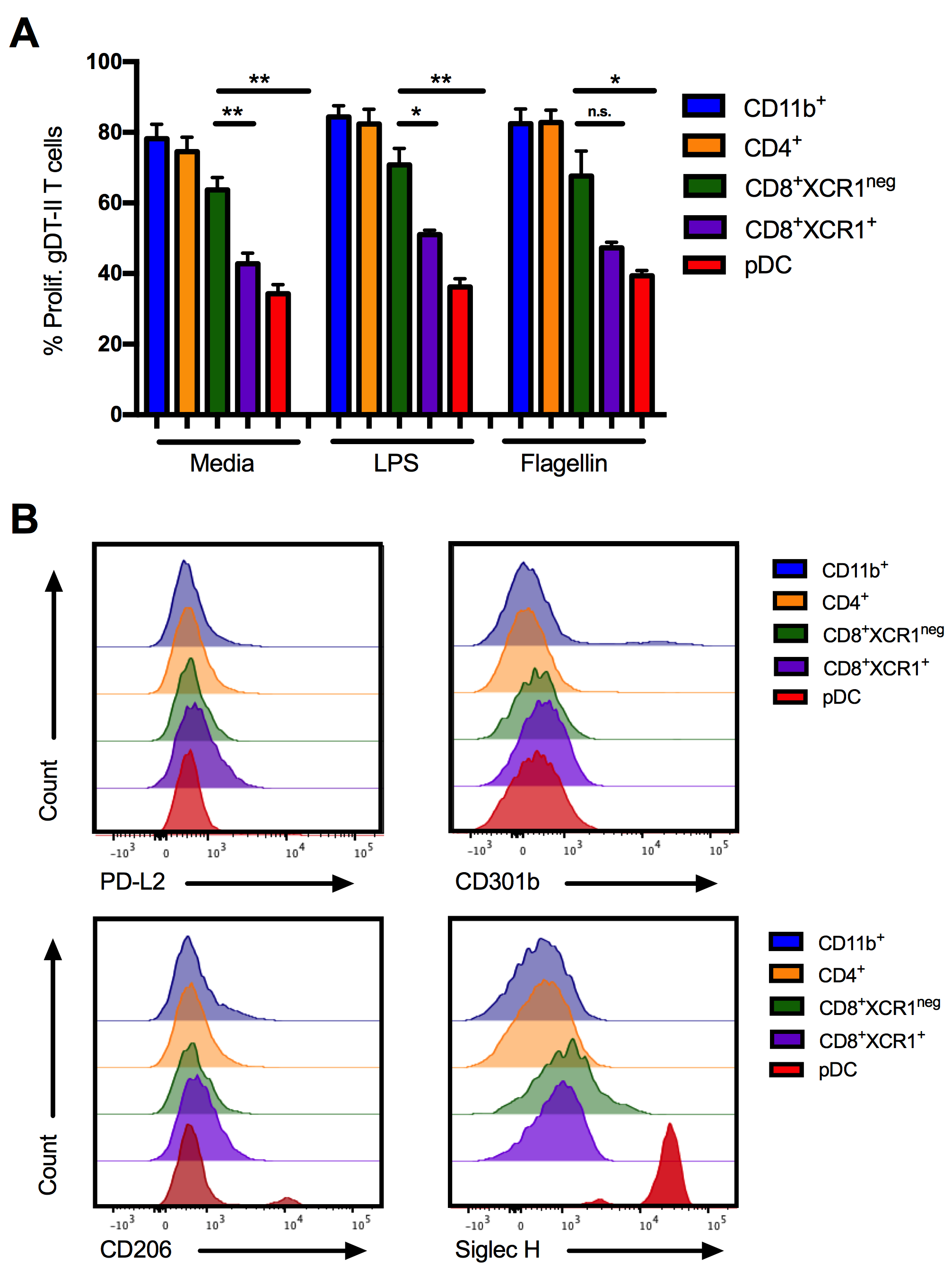

Supplement: Supplementary Figure 5 — Splenic CD8+XCR1neg DCs resemble cDC2 DCs in MHC class II presentation ability and CLR expression. Splenic DCs were sorted as in Supplementary Figure 1B. (A) DC subsets were activated for 24 h in vitro with TLR agonists LPS (100 ng/mL) or flagellin (1 μg/mL) or left unstimulated. Activated DCs were pulsed with 1 nM gD peptide for 1 h and cultured for 4 days with naïve CFSE-labeled gDT-IIs at a 1:10 ratio. Proliferation of gDT-II cells was measured by CFSE dilution. Data are presented as the percentage of CFSE low (proliferated) gDT-II cells and error bars show mean ± SEM from two independent experiments, n = 4. *p > 0.05, **p > 0.01. (B). Expression of PD-L2 and CLRs, CD206, CD301b and Siglec H on DC subsets isolated from spleens of naïve C57Bl/6 mice. Representative plots of 3 independent experiments are shown. [file Image_5.TIFF]
