# Supplementary material for: CD8+XCR1neg Dendritic Cells Express High Levels of Toll-Like Receptor 5 and a Unique Complement of Endocytic Receptors
Source: Front Immunol. 2019 Jan 16;9:2990. doi: 10.3389/fimmu.2018.02990 (PMC6343586; doi:10.3389/fimmu.2018.02990)
Supplement: Supplementary Table 1 — Differentially expressed genes between CD103−XCR1+MHCIIhi and CD103+XCR1+DC in the sdLNs. Differentially expressed genes between CD103−XCR1+MHCIIhi and CD103+XCR1+ DC subsets. The data are derived from TopTable analysis with an FDR < 0.05. [file Table_1.PDF]

Table S1. Differentially expressed genes between CD103-XCR1+MHCIIhi and CD103+XCR1+ DC in the sdLNs

Differentially expressed genes between CD103-XCR1+MHCIIhi and CD103+XCR1+ DC subsets
The data are derived from TopTable analysis with an FDR<0.05.

Gene ID	Log2FC	AveExpr	t	P.Value	adj.P.Value	B
Mir3074-2	2.182923369	5.278872336	4.278963857	3.45E-04	7.52E-03	0.106576681
Tlr13	1.627619812	8.703241941	15.09247529	1.28E-12	2.42E-09	18.30272826
Aldh1a2	1.574173751	5.543874307	5.421763578	2.37E-05	1.35E-03	2.710077514
Spib	1.485030834	6.878346384	6.937438104	8.24E-07	1.51E-04	5.973258932
Dpp4	1.423753707	10.14600062	16.01447987	4.14E-13	1.56E-09	19.25459032
Cd8a	1.403132902	8.739158364	7.463815598	2.75E-07	7.52E-05	7.03366211
Clec12a	1.321233555	9.380610779	10.97373786	4.55E-10	4.29E-07	13.08827076
Gria3	1.194532578	6.160128071	5.843232792	9.05E-06	7.75E-04	3.64563935
Dusp5	1.114835278	7.654534895	8.228190874	5.97E-08	2.50E-05	8.501074597
Pld4	1.000677785	9.764413769	7.833305129	1.30E-07	4.46E-05	7.753751494
Ehf	0.995841886	7.074262335	5.04688227	5.64E-05	2.22E-03	1.863885317
Tlr1	0.972693115	8.018519773	4.968571519	6.78E-05	2.53E-03	1.685791024
Oasl2	0.970955051	6.184009877	4.497765361	2.05E-04	5.34E-03	0.608779451
Trim30a	0.951636051	8.528502272	5.553240758	1.75E-05	1.15E-03	3.003907428
5430437J10Rik	0.918176477	7.554636855	4.368714586	2.79E-04	6.50E-03	0.31257041
Asprv1	0.917186623	7.089679277	4.203444206	4.13E-04	8.44E-03	-0.066667264
Fosb	0.90647102	10.43459645	11.19749124	3.18E-10	3.99E-07	13.41850775
Arid5b	0.903562825	6.304026727	4.54353979	1.84E-04	4.96E-03	0.713805366
Gpr183	0.903250862	8.736456522	6.482842442	2.19E-06	2.90E-04	5.025603709
Gm19723	0.896835614	6.886787169	5.627608276	1.48E-05	1.03E-03	3.169339382
Pde1b	0.871940512	7.49661626	4.994651324	6.38E-05	2.44E-03	1.745145243
Bcl2l11	0.860511658	7.351244766	7.837578179	1.29E-07	4.46E-05	7.761961317
Mnda	0.851432139	9.177238962	4.291877161	3.35E-04	7.42E-03	0.13621006
Apba1	0.846625282	5.694321765	5.753467054	1.11E-05	8.89E-04	3.447978276
Ldlr	0.837147482	7.415915726	7.322360014	3.68E-07	9.25E-05	6.752663157
B3gnt5	0.833253434	5.861956962	5.086069118	5.15E-05	2.19E-03	1.952851136
Pura	0.816093467	7.323970896	4.48434392	2.12E-04	5.39E-03	0.577979201
Themis2	0.801743015	9.674419917	8.332359741	4.88E-08	2.30E-05	8.694392128
Mndal	0.790592866	8.763007765	6.331797374	3.05E-06	3.63E-04	4.704456329
Tlr11	0.788538746	9.432931584	5.72533716	1.18E-05	9.28E-04	3.385851141
Btla	0.765040061	8.4863276	4.867866255	8.58E-05	3.00E-03	1.456223528
Scd2	0.761225157	7.327463785	5.25868811	3.45E-05	1.76E-03	2.34339537
Ptafr	0.760421106	8.032854137	4.846709988	9.02E-05	3.12E-03	1.407925556
Rnase6	0.742960174	10.16806111	6.927335583	8.42E-07	1.51E-04	5.952515114
Dock9	0.739152211	5.981866566	5.42245537	2.36E-05	1.35E-03	2.711627916
Sik1	0.732527306	8.41514791	4.557252059	1.78E-04	4.87E-03	0.745260021
Icos	0.720534968	8.472618963	5.705429156	1.24E-05	9.32E-04	3.341830055
Tnf	0.71772471	8.951195836	5.502492963	1.97E-05	1.21E-03	2.890695035
I830077J02Rik	0.717182504	8.557607012	5.932331941	7.41E-06	6.81E-04	3.840914653
Phf11a	0.706764133	8.857535434	5.942336377	7.24E-06	6.81E-04	3.86278261
Pirb	0.698488914	9.8244488	6.928316381	8.40E-07	1.51E-04	5.954529657
Cd180	0.691136025	9.615059718	5.862297556	8.67E-06	7.60E-04	3.687500812
Gm6377	0.688908739	8.684248536	5.080608128	5.22E-05	2.19E-03	1.940459457
Chd3	0.685740675	8.084725642	6.49303222	2.14E-06	2.90E-04	5.047158622
Ifi27l2a	0.675942155	8.018114552	4.337390749	3.00E-04	6.90E-03	0.240669441
Loxl3	0.674402765	6.642279417	4.616887597	1.55E-04	4.39E-03	0.882010386
Hmgcs1	0.67106338	8.270747815	5.800119672	9.98E-06	8.17E-04	3.550819708
Evl	0.668232657	8.696234512	7.090175614	5.97E-07	1.32E-04	6.285094916
Fgd2	0.656970656	9.020210552	4.827449743	9.44E-05	3.18E-03	1.363936518
Tmem173	0.636565171	8.723526935	6.54284099	1.92E-06	2.79E-04	5.152318468
Dhrs3	0.634816118	7.197166405	4.670885023	1.36E-04	4.03E-03	1.005750293
Alms1	0.615590806	7.382319374	4.51198557	1.99E-04	5.24E-03	0.641410137
Ighm	0.612696298	9.791587563	5.395547707	2.51E-05	1.37E-03	2.651291207
Pyhin1	0.610758849	9.294258255	5.382866786	2.59E-05	1.37E-03	2.622832785
St3gal4	0.605810442	7.270926763	5.896388881	8.03E-06	7.20E-04	3.762251527
Mpeg1	0.599952063	9.402039646	4.574826828	1.71E-04	4.74E-03	0.785569293
Clec2i	0.5974585	8.423080457	4.12352243	4.99E-04	9.69E-03	-0.249852546
Serpina3g	0.588345508	9.750266444	4.165393196	4.52E-04	9.04E-03	-0.153906854
Sdc3	0.585211381	9.558665753	6.224912352	3.86E-06	4.30E-04	4.475380574
4930506M07Rik	0.584979357	9.555455155	6.473874965	2.23E-06	2.90E-04	5.006622763
Cited2	0.572191886	8.808943968	5.824293407	9.45E-06	7.91E-04	3.604011718
Vav1	0.570098832	8.620204871	9.335601419	7.48E-09	4.80E-06	10.4758954
Rnd3	0.566076747	9.183053008	4.669868469	1.37E-04	4.03E-03	1.003421596
Lcp1	0.564893104	11.18046409	8.945105784	1.53E-08	8.23E-06	9.799586588

Dhx58	0.557588939	7.111695806	5.31892803	3.00E-05	1.57E-03	2.479120659
Snora23	0.552850323	8.952696054	4.228771405	3.89E-04	8.15E-03	-0.008578962
Pglyrp1	0.547194718	8.99847216	6.326589996	3.08E-06	3.63E-04	4.693330487
Cyb5r1	0.541419025	7.097028257	4.461611314	2.24E-04	5.62E-03	0.52580653
Tnfaip8l2	0.539798327	8.073018546	4.37833626	2.73E-04	6.46E-03	0.334656777
Slc8b1	0.532140116	8.684286706	5.65759967	1.38E-05	9.99E-04	3.235892334
Phf11b	0.532137923	10.6584096	4.90983157	7.78E-05	2.79E-03	1.551957013
Cd274	0.531427031	8.431430832	4.187521418	4.29E-04	8.68E-03	-0.103178326
LOC73899	0.526622966	8.472098309	4.112888881	5.12E-04	9.84E-03	-0.274209199
Cd44	0.513126516	8.384105976	4.673599249	1.36E-04	4.03E-03	1.011967812
Fes	0.507214606	7.66689926	5.138826721	4.56E-05	2.04E-03	2.072454531
Limd2	0.506988642	8.929954048	4.949690281	7.08E-05	2.62E-03	1.642793825
Atp7a	0.502937979	8.017220413	4.275751092	3.48E-04	7.53E-03	0.099204411
Fnbp1	0.502064205	10.23608777	5.658125156	1.38E-05	9.99E-04	3.237057571
Camk2d	0.500863629	7.126427314	4.325441698	3.09E-04	7.06E-03	0.213243017
Lmo4	0.497085192	8.462543328	4.375920824	2.74E-04	6.46E-03	0.329112174
Il2rg	0.476906698	10.20092691	5.066107353	5.40E-05	2.19E-03	1.907545232
Unc93b1	0.475189663	10.15589925	4.88643221	8.22E-05	2.89E-03	1.498588868
Adrbk2	0.470259643	9.214031684	6.372762904	2.78E-06	3.50E-04	4.79185653
Lyst	0.469163006	9.716795656	5.187523742	4.07E-05	1.93E-03	2.182668291
Plekho2	0.464773935	9.555258859	4.43841385	2.36E-04	5.81E-03	0.472562222
Klf4	0.463285372	8.068468362	5.389891613	2.55E-05	1.37E-03	2.63859967
Atp1a1	0.450828763	9.934634783	4.549686871	1.82E-04	4.93E-03	0.727906641
Ero1lb	0.450755394	8.924627302	5.4230817	2.36E-05	1.35E-03	2.713031568
Il10ra	0.435142241	9.539337693	4.99308632	6.40E-05	2.44E-03	1.741584695
5031439G07Rik	0.422174055	7.97473599	4.54079715	1.86E-04	4.96E-03	0.707513577
Etnk1	0.416883703	9.066965994	4.784749093	1.04E-04	3.45E-03	1.266348349
Lmnb1	0.412886912	8.33251024	4.228205276	3.89E-04	8.15E-03	-0.009877547
Flna	0.405641328	9.697425211	4.482492121	2.13E-04	5.39E-03	0.573729419
Rgs2	0.392024311	10.7464812	5.065518515	5.40E-05	2.19E-03	1.906208365
4632427E13Rik	0.386470742	9.105084465	4.184345444	4.32E-04	8.70E-03	-0.110460047
Erlin1	0.366968479	8.906676362	5.252881655	3.50E-05	1.76E-03	2.330296764
AW549877	0.364142995	8.216257921	4.21429261	4.02E-04	8.28E-03	-0.041788125
Kynu	0.360874418	9.500901259	5.220966073	3.76E-05	1.82E-03	2.258249835
Gns	0.355284772	9.523041536	4.970824528	6.74E-05	2.53E-03	1.690920235
Fos	0.355037241	10.34011871	4.27280547	3.50E-04	7.54E-03	0.092445285
Atp6v0a2	0.352245187	8.727068389	4.807744239	9.89E-05	3.30E-03	1.318912004
Rab43	0.347382163	9.169934332	5.540800481	1.80E-05	1.15E-03	2.976178495
Prkcd	0.341106248	10.48809687	5.233115514	3.66E-05	1.81E-03	2.285686055
Plekho1	0.313697552	9.786064377	4.380408535	2.71E-04	6.46E-03	0.339413666
Nup210	0.271062385	9.562463156	4.493413445	2.08E-04	5.36E-03	0.598792686
Timm8b	-0.255325715	9.250817445	-4.319318246	3.14E-04	7.12E-03	0.199188422
Hspa9	-0.275008885	8.686977268	-4.280451429	3.44E-04	7.52E-03	0.109990235
Rtcb	-0.282433395	10.39745546	-4.163631776	4.54E-04	9.04E-03	-0.157944254
Psmd1	-0.288521819	9.012356677	-4.713252719	1.23E-04	3.78E-03	1.102773849
Fth1	-0.294873445	11.66780373	-5.113849256	4.83E-05	2.13E-03	2.015854932
Psmc6	-0.295218152	9.389233189	-4.293516262	3.33E-04	7.42E-03	0.139971617
Eif3g	-0.297717725	10.57812097	-4.256795497	3.64E-04	7.77E-03	0.055710666
Nsfl1c	-0.308793887	9.625860581	-4.157319466	4.60E-04	9.13E-03	-0.172412085
Cmtm6	-0.327558087	10.03588072	-4.689401229	1.31E-04	3.97E-03	1.048160753
Tma7	-0.328484431	10.09237539	-4.127337684	4.94E-04	9.65E-03	-0.241112501
Ccdc90b	-0.328917652	7.933726226	-4.918286816	7.63E-05	2.76E-03	1.571233872
Ccng1	-0.343824135	10.01916553	-5.18000246	4.14E-05	1.93E-03	2.165657619
Eprs	-0.348419539	8.440869468	-4.114541733	5.10E-04	9.84E-03	-0.270423542
Med10	-0.358873978	9.464074613	-4.140256372	4.79E-04	9.43E-03	-0.211514304
Gtpbp4	-0.360547502	9.94326197	-5.705452782	1.24E-05	9.32E-04	3.341882324
Snhg12	-0.361706263	10.12606681	-4.485979166	2.11E-04	5.39E-03	0.58173197
Cpd	-0.363347552	7.138861168	-4.367041652	2.80E-04	6.50E-03	0.308730244
2810428I15Rik	-0.381879478	8.847852183	-4.639945842	1.47E-04	4.19E-03	0.934860978
Rin3	-0.383138715	9.071171021	-4.201925774	4.14E-04	8.44E-03	-0.070149323
Prps2	-0.403552951	7.390724665	-4.255351121	3.65E-04	7.77E-03	0.052396783
Ptpn12	-0.404894439	8.38912839	-4.73571244	1.17E-04	3.68E-03	1.154180947
Dad1	-0.408797997	9.404591649	-4.718468957	1.22E-04	3.78E-03	1.114714781
Psmd10	-0.4097791	8.944532348	-5.608909028	1.54E-05	1.06E-03	3.127796483
Kat2a	-0.409855595	7.175666915	-4.401362366	2.58E-04	6.24E-03	0.387513076
Bccip	-0.416446995	8.906753681	-4.929432088	7.43E-05	2.72E-03	1.596637448
Crot	-0.422206724	7.472947051	-4.225187273	3.92E-04	8.16E-03	-0.01680011
Psmd11	-0.424649566	9.163201297	-4.767370454	1.09E-04	3.50E-03	1.226607667
Mrpl16	-0.4339018	7.787653503	-4.768103388	1.09E-04	3.50E-03	1.228283971
Ndufaf2	-0.441009656	7.725851644	-5.047100184	5.64E-05	2.22E-03	1.864380337

Dstn	-0.45468545	9.509330842	-5.385545282	2.57E-05	1.37E-03	2.628845073
Aco1	-0.455699187	8.073504308	-4.560620356	1.77E-04	4.87E-03	0.752986008
Sssca1	-0.461192431	7.475136068	-4.43690339	2.37E-04	5.81E-03	0.469095185
Rnf130	-0.483283834	9.432565763	-5.645519713	1.42E-05	1.01E-03	3.209097556
Tet3	-0.490284736	8.021395469	-4.679484275	1.34E-04	4.03E-03	1.025447891
Snx11	-0.499464202	7.075745421	-4.65910036	1.40E-04	4.04E-03	0.978752229
Fli1	-0.500498224	8.345223609	-4.441436729	2.35E-04	5.81E-03	0.479500746
Arhgef12	-0.502097145	5.942036877	-4.769191387	1.08E-04	3.50E-03	1.230772306
Arhgap25	-0.51418892	8.000823728	-4.229045318	3.88E-04	8.15E-03	-0.007950658
H2-Ke6	-0.525593579	8.553088376	-4.829765673	9.39E-05	3.18E-03	1.369226899
Zc2hc1a	-0.531243658	7.335114838	-5.402128717	2.48E-05	1.37E-03	2.666054424
Olfm1	-0.531624351	7.388906806	-6.022177035	6.05E-06	6.01E-04	4.036869612
Map2k3	-0.532077311	7.959959013	-4.598290525	1.62E-04	4.52E-03	0.839374553
Ndufa12	-0.533023318	10.32803259	-5.543954736	1.79E-05	1.15E-03	2.983210695
Rcn2	-0.533331772	6.831274168	-5.153202234	4.41E-05	2.00E-03	2.105008781
Zfp385a	-0.535822404	8.666631898	-5.519869472	1.89E-05	1.19E-03	2.929488924
Gstp1	-0.554879313	9.459110516	-4.449625673	2.30E-04	5.75E-03	0.498296823
Sash1	-0.55884967	6.453687552	-5.972476144	6.77E-06	6.54E-04	3.928590609
Commd3	-0.579114501	8.881836971	-6.747105481	1.24E-06	1.94E-04	5.58000581
Hps3	-0.590396378	8.345855002	-4.220093427	3.97E-04	8.22E-03	-0.028483698
Galk1	-0.598920347	5.981854271	-4.365108857	2.81E-04	6.50E-03	0.304293584
Sigmar1	-0.613417087	7.008433043	-4.389022892	2.66E-04	6.38E-03	0.359187883
1500011K16Rik	-0.613811599	7.720054498	-6.222326856	3.88E-06	4.30E-04	4.469820987
Nans	-0.625752509	8.399407472	-5.006117321	6.21E-05	2.41E-03	1.771226835
Aff3	-0.652926816	7.048278479	-5.225499971	3.72E-05	1.82E-03	2.268489814
Fam162a	-0.657287256	7.857495524	-5.161987861	4.32E-05	1.98E-03	2.124896694
Slc6a12	-0.66675466	6.258640874	-4.901369939	7.93E-05	2.82E-03	1.532661599
Pdgfa	-0.674229004	6.39406221	-4.406149307	2.55E-04	6.21E-03	0.398501424
Stk10	-0.676453774	8.138296859	-5.077808743	5.25E-05	2.19E-03	1.934106484
Dhrs11	-0.697618461	6.790519072	-6.77373808	1.17E-06	1.94E-04	5.635340604
Ppil3	-0.733253805	7.533747424	-6.086537362	5.24E-06	5.65E-04	4.176636759
Hirip3	-0.736046395	6.42377956	-7.100909109	5.84E-07	1.32E-04	6.306882473
Dkc1	-0.736229386	6.678650642	-4.71711801	1.22E-04	3.78E-03	1.111622312
Sesn2	-0.766622797	7.213054822	-6.747829343	1.23E-06	1.94E-04	5.581511102
LOC102632310	-0.771840987	6.818439964	-4.285000726	3.40E-04	7.50E-03	0.120429771
Psd3	-0.786878145	5.251119094	-4.606321929	1.59E-04	4.47E-03	0.857788524
Cks1b	-0.793002232	8.850983575	-4.658246806	1.41E-04	4.04E-03	0.97679661
Sult1a1	-0.796181287	6.841788588	-5.589697876	1.61E-05	1.08E-03	3.085078354
Zfp189	-0.800992018	6.913289242	-4.748845251	1.14E-04	3.62E-03	1.184230958
Olfr98	-0.820842165	7.521583878	-4.296052605	3.31E-04	7.42E-03	0.145792314
Mrps5	-0.820919976	6.988145611	-4.139608542	4.80E-04	9.43E-03	-0.212998695
Ephx1	-0.836866314	6.46295913	-5.44160785	2.26E-05	1.35E-03	2.754533378
Prc1	-0.881618007	6.137332334	-5.112006715	4.85E-05	2.13E-03	2.011677874
Cst6	-0.891253196	3.923886683	-5.074676155	5.29E-05	2.19E-03	1.926996685
Dapk1	-0.92614704	6.611717922	-4.503344636	2.03E-04	5.31E-03	0.621582386
Sept3	-0.928272448	7.385939	-5.070409065	5.34E-05	2.19E-03	1.917310892
Smyd5	-0.951783717	6.031133565	-5.184531488	4.10E-05	1.93E-03	2.175901323
Ikzf4	-0.963001183	5.968379243	-6.026736073	5.99E-06	6.01E-04	4.046786945
Mboat1	-0.990004102	5.965190479	-4.660003229	1.40E-04	4.04E-03	0.980820813
Hrsp12	-1.011584263	6.419549095	-5.453843661	2.20E-05	1.34E-03	2.781925877
Entpd1	-1.014963448	7.963696257	-6.729516667	1.28E-06	1.94E-04	5.543406786
Itgae	-1.039380248	5.754123003	-5.257808183	3.46E-05	1.76E-03	2.34141055
Mtss1	-1.043831724	6.961106159	-7.456115816	2.79E-07	7.52E-05	7.018442227
Sprr4	-1.064189879	5.221341317	-4.746097265	1.14E-04	3.62E-03	1.177943691
Myo6	-1.105824092	5.736837342	-5.053336477	5.56E-05	2.22E-03	1.878545547
Cd207	-1.245980276	6.744875284	-7.583328258	2.15E-07	6.77E-05	7.268779916
2210409E12Rik	-1.303864742	5.310097792	-4.305146623	3.24E-04	7.32E-03	0.166662964
Ska2	-1.332833175	6.698848109	-6.05516401	5.62E-06	5.89E-04	4.1085691
Cib3	-1.370260421	4.915802268	-4.83350767	9.30E-05	3.18E-03	1.377774358
BC033916	-1.857176593	5.963136809	-7.020245718	6.92E-07	1.45E-04	6.142739297
BE692007	-1.90022999	5.353110861	-4.526616534	1.92E-04	5.09E-03	0.674980211
Sprr1a	-1.916120143	5.528731847	-9.32393102	7.64E-09	4.80E-06	10.4559937
