# Supplementary material for: CD8+XCR1neg Dendritic Cells Express High Levels of Toll-Like Receptor 5 and a Unique Complement of Endocytic Receptors
Source: Front Immunol. 2019 Jan 16;9:2990. doi: 10.3389/fimmu.2018.02990 (PMC6343586; doi:10.3389/fimmu.2018.02990)
Supplement: Supplementary Table 2 — Differentially expressed genes between CD103−XCR1+MHCIIlow and CD8+XCR1+ DC in the sdLNs. Differentially expressed genes between CD103−XCR1+MHCIIlow and CD8+XCR1+ DC subsets. The data are derived from TopTable analysis with an FDR < 0.05. [file Table_2.PDF]

Table S2. Differentially expressed genes between CD103-XCR1+MHCIIlow and CD8+XCR1+ DC in the sdLNs

Differentially expressed genes between CD103-XCR1+MHCIIlow and CD8+XCR1+ DC subsets
The data are derived from TopTable analysis with an FDR<0.05.

Gene ID	Log2FC	AveExpr	t	P.Value	adj.P.Value	B
Serpinb10	3.105630645	5.677656661	11.26569836	2.85E-10	3.07E-08	13.71156273
Clec4b1	1.94472513	4.455076531	7.092279279	5.94E-07	1.41E-05	5.992395505
Gpr141	1.729937043	6.446440822	8.524797416	3.37E-08	1.30E-06	8.897859584
Sell	1.64166647	7.831860346	8.596261998	2.94E-08	1.18E-06	9.035523704
Fdx1l	1.57179957	4.351755823	2.693844879	1.37E-02	4.15E-02	-3.988741964
Emb	1.479689605	7.983373404	6.631201892	1.59E-06	3.25E-05	4.998685442
Cdca7	1.446674349	5.397573053	4.761962245	1.10E-04	9.35E-04	0.73002057
Bex6	1.356102899	5.087320473	5.581247067	1.64E-05	2.14E-04	2.640116929
Ccr2	1.347645905	8.945593659	7.812241432	1.36E-07	4.16E-06	7.487466264
Ckap4	1.258814607	5.940182469	6.216284487	3.93E-06	6.63E-05	4.081537045
Hist2h2bb	1.229066126	5.166653987	4.105342432	5.21E-04	3.25E-03	-0.8159256
Hist1h2ab	1.227873094	6.91739219	2.65937354	1.48E-02	4.38E-02	-4.060048061
A530064D06Rik	1.195720271	4.824906537	2.669764154	1.45E-02	4.33E-02	-4.038601018
Klrb1b	1.17192328	6.37398408	6.497041806	2.12E-06	4.08E-05	4.704435041
Chil5	1.125648479	6.259129028	5.942471734	7.24E-06	1.10E-04	3.465237896
Hist2h2ab	1.105491936	7.317596392	4.055422028	5.86E-04	3.54E-03	-0.93301747
Esco2	1.099818386	5.266789131	4.971451276	6.73E-05	6.38E-04	1.222151036
Clec4a2	1.065866648	4.46171855	4.591492717	1.65E-04	1.32E-03	0.32858362
Pdlim1	1.042412193	6.176726668	3.646591786	1.54E-03	7.53E-03	-1.884418902
LOC102640273	1.01414036	4.717692076	3.720095783	1.30E-03	6.60E-03	-1.714629907
Cdca5	1.013804616	6.465459127	4.778809294	1.06E-04	9.06E-04	0.769656193
Rad51	1.003944726	6.443806365	3.50057261	2.17E-03	9.77E-03	-2.219511652
Racgap1	1.002783833	6.844890032	5.227155802	3.71E-05	3.98E-04	1.819918464
Psat1	1.001626539	5.968781552	5.435763175	2.29E-05	2.73E-04	2.304346886
Gm14548	0.996139567	7.357601006	3.592834253	1.75E-03	8.26E-03	-2.00814618
Cd300a	0.994289843	6.707110784	3.624137274	1.63E-03	7.85E-03	-1.936148081
Dhcr24	0.992056325	7.184347266	7.005123486	7.14E-07	1.64E-05	5.806685573
Mcm5	0.950113149	7.500254275	6.209318697	3.99E-06	6.69E-05	4.065963556
Uhrf1	0.947318858	8.288915739	7.732542903	1.59E-07	4.68E-06	7.325422844
S100a10	0.946978674	10.22979102	7.488672061	2.61E-07	7.09E-06	6.824216666
Gm1966	0.926488204	7.856155264	6.555751367	1.87E-06	3.67E-05	4.833477877
Ifi203	0.918465745	8.372503605	6.357378835	2.88E-06	5.27E-05	4.395756463
Cd226	0.91793557	6.123845494	3.265681788	3.76E-03	1.50E-02	-2.75099856
Lyl1	0.917908417	5.370303578	5.129827476	4.65E-05	4.75E-04	1.5928495
Cxx1b	0.884042973	7.029144456	4.762763172	1.10E-04	9.35E-04	0.73190508
Spc24	0.877065081	6.634818845	5.139381967	4.55E-05	4.66E-04	1.615167054
Ifitm3	0.860885798	9.440855115	5.430725743	2.32E-05	2.75E-04	2.292688645
Krt80	0.85845704	6.615562215	4.438450164	2.36E-04	1.76E-03	-0.032097039
Ctnnd2	0.856403436	6.170108896	5.945257494	7.19E-06	1.10E-04	3.471549666
Arhgap19	0.85231443	5.605952042	4.072491321	5.63E-04	3.46E-03	-0.89299622
Wbp5	0.851944107	7.829932972	5.204834452	3.91E-05	4.16E-04	1.767897014
Ect2	0.850865102	6.642866292	5.174753737	4.19E-05	4.37E-04	1.697739451
Hist1h2bf	0.847385172	8.767130309	4.067529232	5.70E-04	3.49E-03	-0.904632277
E2f8	0.842517248	6.283492015	4.976220262	6.66E-05	6.32E-04	1.233332756
Shcbp1	0.841237143	5.881510491	3.106755855	5.42E-03	1.98E-02	-3.104054491
Dtl	0.834478507	6.323683569	4.868361223	8.57E-05	7.57E-04	0.980185974
Dck	0.831331113	6.87177999	4.919488362	7.60E-05	6.97E-04	1.100245773
Gins2	0.828843488	7.687115237	5.716956473	1.20E-05	1.67E-04	2.951612738
Cdca3	0.828418526	6.821949373	3.820388343	1.02E-03	5.50E-03	-1.481944284
Sh2d1b1	0.825318239	4.516968073	2.374164303	2.74E-02	7.02E-02	-4.631690814
Ifi27l2a	0.825095049	8.018114552	5.294476174	3.17E-05	3.50E-04	1.976604177
Abcb1b	0.823994198	5.72263566	2.578668974	1.77E-02	5.00E-02	-4.225217901
Mmp12	0.820834444	6.593020593	2.657330134	1.49E-02	4.40E-02	-4.064261036
Cd22	0.820370218	6.406835851	5.047030409	5.64E-05	5.55E-04	1.3992243
Fam111a	0.819410247	8.15542721	5.462959637	2.15E-05	2.65E-04	2.36725217
Ms4a6c	0.811558078	7.748411	3.582405982	1.79E-03	8.44E-03	-2.032100742
Il31ra	0.810760262	5.148853816	4.684760511	1.32E-04	1.10E-03	0.548292465
Gins1	0.80941557	6.392274297	5.42915471	2.33E-05	2.75E-04	2.289052345
2810417H13Rik	0.807667172	6.605113048	3.080528447	5.75E-03	2.08E-02	-3.161729914
Kifc1	0.802216932	5.876097953	2.11962277	4.63E-02	1.05E-01	-5.110140451
Rad51ap1	0.80028853	5.652995835	3.695190888	1.38E-03	6.88E-03	-1.772233825
Nuf2	0.800160083	6.831419082	3.70030097	1.36E-03	6.83E-03	-1.760420559

Milr1	0.791612849	6.356383792	2.797673073	1.09E-02	3.45E-02	-3.771383041
1110001J03Rik	0.791265888	8.123282875	4.684472837	1.32E-04	1.10E-03	0.547615035
Rgs18	0.790816258	7.008813238	3.639027672	1.57E-03	7.64E-03	-1.901852165
E2f7	0.788674489	5.74246035	5.500830945	1.97E-05	2.48E-04	2.454745184
Cd209a	0.788019897	7.100332225	3.62092912	1.64E-03	7.89E-03	-1.943533227
Plac8	0.787580652	9.747400092	3.689347478	1.39E-03	6.94E-03	-1.785738427
Asf1b	0.787431162	6.814742429	3.975926165	7.08E-04	4.09E-03	-1.119161299
Prim1	0.769552117	6.635813257	3.271766101	3.70E-03	1.48E-02	-2.737368231
Rrm1	0.76827715	7.725356115	6.717096157	1.32E-06	2.78E-05	5.185885892
Cdkn3	0.767670679	6.479366691	3.097432449	5.53E-03	2.02E-02	-3.124577363
Nusap1	0.766539945	7.094866574	3.647228655	1.54E-03	7.53E-03	-1.882950742
Tyms	0.763642232	7.393578347	4.593284529	1.64E-04	1.32E-03	0.332805732
Ncapd2	0.761687502	7.284973666	4.78672766	1.04E-04	8.94E-04	0.788282564
Rsph3a	0.760583406	6.627604681	2.114466698	4.68E-02	1.06E-01	-5.119483728
Mcm3	0.760163684	7.943897165	6.060912803	5.55E-06	8.83E-05	3.732851443
Prr11	0.758210101	7.336073471	4.328151849	3.07E-04	2.17E-03	-0.291962056
Clspn	0.756835904	6.121162963	3.983228044	6.96E-04	4.04E-03	-1.102081625
Fignl1	0.7562429	5.726990431	3.272723052	3.70E-03	1.48E-02	-2.73522371
Ccna2	0.755126521	7.486485905	3.002911947	6.87E-03	2.39E-02	-3.331345879
Mcm7	0.750739672	6.914199869	4.552161039	1.81E-04	1.43E-03	0.235897268
Cdc45	0.74988716	6.657804356	8.000894562	9.33E-08	3.08E-06	7.867588985
Hmga1-rs1	0.749860941	9.655638175	4.045572715	6.00E-04	3.60E-03	-0.956102536
Chaf1a	0.747717508	6.586308494	4.330452046	3.05E-04	2.16E-03	-0.286544769
Rnd3	0.744909475	9.183053008	6.14515485	4.60E-06	7.48E-05	3.922248575
Dctpp1	0.744615133	8.693015794	4.004577935	6.61E-04	3.90E-03	-1.052120738
Pbk	0.743380014	6.318359666	3.423510804	2.60E-03	1.12E-02	-2.395003546
Shmt2	0.740235864	6.958254088	3.81478907	1.04E-03	5.56E-03	-1.494963156
Snora17	0.739773703	6.176337473	2.85354876	9.62E-03	3.12E-02	-3.652887706
Tcf19	0.738261014	6.22572444	4.296802065	3.31E-04	2.31E-03	-0.365783347
Nrp1	0.737444925	6.127841616	5.448046066	2.23E-05	2.72E-04	2.332764724
Anln	0.737024054	6.283065888	5.416452356	2.40E-05	2.81E-04	2.259644214
Cnn3	0.734429978	6.723065266	2.487659433	2.15E-02	5.83E-02	-4.408335981
Kif11	0.733601999	7.209686623	6.592720609	1.72E-06	3.42E-05	4.914515508
Ccnb2	0.731259387	7.145030868	2.722153651	1.29E-02	3.95E-02	-3.9298572
Hist1h2an	0.728801495	8.371550245	3.493786039	2.21E-03	9.88E-03	-2.235006729
Kif4	0.727557495	6.112230354	3.863443946	9.24E-04	5.07E-03	-1.381733403
Zdhhc15	0.725922867	5.807321038	3.473211816	2.32E-03	1.03E-02	-2.281935395
Stil	0.725554216	6.086438878	5.44572129	2.24E-05	2.72E-04	2.327387049
Kntc1	0.722691244	5.508194284	5.033013378	5.83E-05	5.66E-04	1.366406485
Ckap2l	0.721051575	5.816784486	3.92186938	8.05E-04	4.54E-03	-1.245478669
BC147527	0.720600555	6.31984269	2.358336097	2.83E-02	7.21E-02	-4.662375175
Wdhd1	0.719966669	7.084076548	5.069227123	5.36E-05	5.30E-04	1.451171038
5430402E10Rik	0.719925491	1.758841863	2.120210072	4.63E-02	1.05E-01	-5.10907528
Cks1b	0.718497311	8.850983575	4.220590649	3.96E-04	2.64E-03	-0.545130736
Mki67	0.717981755	8.862648692	4.714195576	1.23E-04	1.04E-03	0.617598142
Dhfr	0.712874174	5.5654861	3.902828601	8.42E-04	4.69E-03	-1.289916408
Lbr	0.709200572	6.872942257	3.823540385	1.02E-03	5.47E-03	-1.47461409
Mcm6	0.704835432	8.373850819	4.766954969	1.09E-04	9.30E-04	0.741767702
Cenph	0.704268782	6.515609191	4.110012951	5.15E-04	3.22E-03	-0.804963536
Lig1	0.70316322	8.180028593	8.077760319	8.02E-08	2.70E-06	8.02107861
Sec61b	0.702360237	9.228497948	3.900202965	8.47E-04	4.71E-03	-1.296041763
Stambpl1	0.69732547	6.595963499	3.061091493	6.01E-03	2.14E-02	-3.204357523
Syce2	0.69142832	7.435500125	5.43904777	2.27E-05	2.73E-04	2.311947377
Birc5	0.691142037	6.453424096	3.689428157	1.39E-03	6.94E-03	-1.785552
Hmmr	0.686590753	5.981850155	5.436341968	2.29E-05	2.73E-04	2.305686265
Foxm1	0.683768146	6.582058808	4.673114076	1.36E-04	1.12E-03	0.520865438
Aurkb	0.681838562	6.940124183	2.663545555	1.47E-02	4.36E-02	-4.051441565
Ube2c	0.6790965	8.272575505	3.226879547	4.11E-03	1.60E-02	-2.837735662
Atad2	0.678392507	7.506059793	7.45531751	2.80E-07	7.43E-06	6.7550385
Lgals1	0.67799832	9.844921301	5.159411565	4.34E-05	4.50E-04	1.661934161
Kif22	0.677149828	6.124604266	4.067493586	5.70E-04	3.49E-03	-0.904715861
Cbx5	0.675491614	8.048774449	5.728271313	1.17E-05	1.63E-04	2.977504873
Casc5	0.675344292	7.128899501	4.695412867	1.29E-04	1.08E-03	0.573375974
Nrm	0.673217696	8.795855381	5.71043683	1.22E-05	1.69E-04	2.936687973
Mcm2	0.673031389	7.157555036	4.885479467	8.24E-05	7.43E-04	1.02039604
Cenpa	0.670599436	6.942435553	5.01916904	6.02E-05	5.83E-04	1.333982687
Exosc8	0.666886299	8.512593988	6.613890702	1.65E-06	3.34E-05	4.960843816
Pola1	0.665840816	6.805403006	3.837928618	9.81E-04	5.34E-03	-1.441141261

Mef2c	0.665672078	7.304929711	3.539874405	1.98E-03	9.11E-03	-2.129633068
Plk1	0.664267785	6.298864528	3.351591801	3.08E-03	1.29E-02	-2.557827611
Ncaph	0.664173788	6.332242332	3.413360012	2.66E-03	1.15E-02	-2.418042816
Hist1h2af	0.663346785	7.634328566	3.10563582	5.43E-03	1.99E-02	-3.106521104
Cysltr1	0.660787605	5.337569228	2.666295064	1.46E-02	4.35E-02	-4.045765997
Cotl1	0.659515966	8.606640306	3.393514913	2.79E-03	1.19E-02	-2.463031171
Ndc80	0.655218054	7.265193978	2.906664691	8.54E-03	2.87E-02	-3.539315248
Ccnf	0.654582801	5.685526494	4.137499023	4.83E-04	3.06E-03	-0.740429114
Aspm	0.653551118	5.80728042	3.922723115	8.03E-04	4.54E-03	-1.243485499
H2afx	0.6528641	10.35689016	4.625076058	1.52E-04	1.24E-03	0.407710737
Cdca8	0.651388612	7.372109183	4.131924417	4.89E-04	3.09E-03	-0.753520737
Kif18b	0.647174566	6.065445181	2.853700213	9.62E-03	3.12E-02	-3.652565136
Diap3	0.647001275	6.309115058	2.826669626	1.02E-02	3.29E-02	-3.710018244
Fcer1g	0.645984785	10.27034666	6.343140074	2.97E-06	5.36E-05	4.364153699
Dlgap5	0.645644391	6.945147684	6.824067398	1.05E-06	2.27E-05	5.417703201
Ms4a4c	0.644761657	6.976595002	2.603500784	1.67E-02	4.80E-02	-4.174667974
Gpr183	0.642769664	8.736456522	4.613308032	1.56E-04	1.26E-03	0.379985262
Hpgd	0.642650758	7.028232134	2.853597312	9.62E-03	3.12E-02	-3.6527843
Klri2	0.640696115	5.958130431	2.331328034	3.00E-02	7.55E-02	-4.714460245
Sept11	0.638934722	8.52495266	4.948879709	7.10E-05	6.63E-04	1.169213319
Gm4956	0.636125166	5.336935621	2.418195315	2.49E-02	6.51E-02	-4.545724398
Srm	0.629120401	7.430320503	3.135431034	5.07E-03	1.89E-02	-3.040796816
Mis18bp1	0.628715227	5.871107781	5.438738342	2.28E-05	2.73E-04	2.311231405
Cdc7	0.628635683	6.252031741	3.128156277	5.16E-03	1.92E-02	-3.056864401
Serpinb2	0.626679482	4.017203926	2.341823361	2.93E-02	7.41E-02	-4.694261107
Haus1	0.625840687	7.063965573	3.408403931	2.70E-03	1.16E-02	-2.429284878
Dkc1	0.623632607	6.678650642	3.995695713	6.75E-04	3.96E-03	-1.072909918
Slc7a5	0.622842275	8.415955372	5.107792999	4.90E-05	4.93E-04	1.541359819
Bub1	0.622036666	5.929172823	4.011119871	6.51E-04	3.86E-03	-1.036805622
Top2a	0.612446671	8.384721952	5.139845675	4.54E-05	4.66E-04	1.616250047
Galk1	0.607932272	5.981854271	4.430790439	2.41E-04	1.78E-03	-0.050148224
Cox6b2	0.606223858	6.884386771	3.015216331	6.68E-03	2.35E-02	-3.304566537
B930041F14Rik	0.60079899	5.652051417	3.318727244	3.32E-03	1.36E-02	-2.631902218
Hist1h1c	0.60074811	8.548923635	2.752970613	1.20E-02	3.74E-02	-3.865428937
Hells	0.600213612	7.467238717	5.195991448	3.99E-05	4.22E-04	1.747278555
Lsm2	0.592626432	8.462852179	3.559970041	1.89E-03	8.81E-03	-2.083584013
Ybx3	0.591577258	9.667879521	7.408020812	3.08E-07	8.07E-06	6.656685877
Ticam2	0.591329692	6.536630763	3.720487209	1.30E-03	6.60E-03	-1.713723963
Tkt	0.591151043	8.508079739	4.953401376	7.02E-05	6.58E-04	1.179820031
Smc2	0.58942957	8.104656369	5.342254974	2.84E-05	3.20E-04	2.087608422
Ncapg	0.586704333	6.221170747	3.39650179	2.77E-03	1.18E-02	-2.456264622
Hirip3	0.578635845	6.42377956	5.582311894	1.64E-05	2.14E-04	2.642567675
Spag5	0.577699831	6.289914974	3.198574922	4.39E-03	1.68E-02	-2.900794831
Tbxas1	0.576772539	5.933410115	3.608584036	1.69E-03	8.05E-03	-1.971938249
Tipin	0.576693263	6.755392379	2.818123967	1.04E-02	3.35E-02	-3.728132249
Incenp	0.576290662	6.942759663	5.976101524	6.71E-06	1.04E-04	3.541378209
Tpx2	0.574736528	7.015767367	3.541141934	1.98E-03	9.09E-03	-2.126730353
Lmnb1	0.573656618	8.33251024	5.874581807	8.43E-06	1.26E-04	3.311163866
Tacc3	0.571989368	7.704170081	4.15424275	4.64E-04	2.98E-03	-0.701098628
Cep55	0.570643689	6.104527312	2.797394527	1.09E-02	3.45E-02	-3.77197116
Topbp1	0.570574248	8.513326381	5.383779905	2.58E-05	2.99E-04	2.183942356
Snora68	0.568341074	6.266093098	2.451565917	2.32E-02	6.18E-02	-4.479987874
Dpysl2	0.567533983	7.879435438	4.19200259	4.24E-04	2.77E-03	-0.612356192
Fbxo5	0.563265696	7.738206919	3.794226697	1.09E-03	5.79E-03	-1.54274536
Ccnb1	0.561616874	6.983603521	2.597659625	1.69E-02	4.85E-02	-4.186580773
Scd2	0.56006077	7.327463785	3.86900628	9.12E-04	5.02E-03	-1.368774432
Ppat	0.560025823	8.311824528	5.102871521	4.95E-05	4.98E-04	1.529855451
Rhof	0.557019744	7.134903993	3.333683638	3.21E-03	1.33E-02	-2.598218078
LOC102634079	0.555150849	6.308990362	2.094442085	4.88E-02	1.09E-01	-5.155631246
Hspd1	0.555114399	10.00018943	4.802148257	1.00E-04	8.72E-04	0.824550748
Nasp	0.554042952	8.464926462	5.370467564	2.66E-05	3.08E-04	2.153073536
Mcm4	0.553747427	8.473777556	5.637260577	1.44E-05	1.92E-04	2.768894147
Ckap2	0.553293086	5.61373894	3.293006996	3.53E-03	1.42E-02	-2.689721941
Sptssa	0.551074164	8.63194741	3.762103502	1.17E-03	6.15E-03	-1.617303147
Vrk1	0.546841659	8.933395091	5.669255174	1.34E-05	1.82E-04	2.842320521
Ran	0.546503395	9.152434151	5.325249829	2.96E-05	3.29E-04	2.048119983
Kif15	0.546040326	6.397428172	2.829514225	1.02E-02	3.27E-02	-3.703983293
Cenpf	0.545471657	5.955520099	3.923052406	8.02E-04	4.54E-03	-1.242716704

Pa2g4	0.54547045	10.35086929	4.496017938	2.06E-04	1.59E-03	0.103578686
Gpatch4	0.545360795	6.599658701	3.455976988	2.41E-03	1.05E-02	-2.321192413
Pecam1	0.543729527	6.861937052	4.30380701	3.25E-04	2.28E-03	-0.349290383
Tfdp1	0.543596033	8.605353186	5.469025714	2.12E-05	2.62E-04	2.381274594
Idh3a	0.54277828	5.973003123	2.250532777	3.55E-02	8.62E-02	-4.868165268
Ets2	0.541403241	6.114770552	2.192579787	4.00E-02	9.44E-02	-4.976396405
Kif20b	0.540902874	6.961430944	4.225513984	3.92E-04	2.62E-03	-0.53355035
Sdf2l1	0.540436652	8.648768234	4.1568018	4.61E-04	2.96E-03	-0.695086359
2700094K13Rik	0.53939467	9.542577716	5.453971345	2.20E-05	2.70E-04	2.346469068
Spn	0.538721806	6.44509541	2.453833979	2.31E-02	6.16E-02	-4.475502109
Kif23	0.537446128	7.012581323	3.597945882	1.73E-03	8.20E-03	-1.996398665
Gstk1	0.534939355	6.017326542	2.608031095	1.66E-02	4.78E-02	-4.165419348
Dyrk3	0.534264173	6.053546435	3.109118748	5.39E-03	1.98E-02	-3.098849731
Dtd1	0.531502752	6.241813837	3.555180159	1.91E-03	8.90E-03	-2.09456556
Klf4	0.531263753	8.068468362	6.180756444	4.25E-06	7.03E-05	4.002047927
Sae1	0.530761576	8.079304943	5.961944087	6.93E-06	1.07E-04	3.50933954
Nme1	0.530457612	8.797112597	3.726815401	1.28E-03	6.54E-03	-1.699075037
Nt5c3b	0.530275954	6.25784932	2.478323203	2.19E-02	5.92E-02	-4.426924187
LOC102631525	0.526053708	7.015069863	2.30464526	3.17E-02	7.88E-02	-4.765575325
Prmt1	0.525139837	8.313782018	4.200316244	4.16E-04	2.73E-03	-0.592809672
D430020J02Rik	0.524793639	6.267967899	2.736710225	1.25E-02	3.85E-02	-3.899466018
Lamtor4	0.523919189	8.438136959	3.297679515	3.49E-03	1.42E-02	-2.679228136
Lgals9	0.523064988	7.785499644	2.298263689	3.21E-02	7.97E-02	-4.777749085
Chek1	0.520337567	5.901866106	4.188649453	4.27E-04	2.78E-03	-0.62023909
Prc1	0.519988497	6.137332334	3.015120681	6.68E-03	2.35E-02	-3.304774871
Tfrc	0.519302822	7.153072695	2.684085656	1.40E-02	4.22E-02	-4.008974253
Atp5o	0.517456555	9.748730843	4.468348662	2.20E-04	1.66E-03	0.03836629
Utp20	0.516768161	6.347473314	5.003257555	6.25E-05	5.98E-04	1.296705212
Gins3	0.516308062	7.043052593	2.507882034	2.06E-02	5.62E-02	-4.367945407
Ncf2	0.516186818	8.233993957	3.63465485	1.59E-03	7.70E-03	-1.911926861
Krtcap2	0.515521632	9.686981593	5.177465376	4.16E-05	4.37E-04	1.704066255
Nop58	0.515510408	8.659630321	4.120083545	5.03E-04	3.16E-03	-0.78132324
Dnajc9	0.51394903	8.619504314	6.93284293	8.32E-07	1.84E-05	5.651911606
Bub1b	0.512855917	6.893234691	3.444852525	2.48E-03	1.07E-02	-2.346504389
Gen1	0.512753225	6.162161115	2.727028542	1.28E-02	3.91E-02	-3.919687826
Hn1l	0.509726903	7.756053008	4.876772535	8.41E-05	7.51E-04	0.999945268
Rin2	0.508125062	5.804000711	2.779845768	1.13E-02	3.55E-02	-3.808970531
Cdc20	0.505504251	6.669799448	3.538614776	1.99E-03	9.12E-03	-2.132517449
Lifr	0.500800416	5.738064486	2.307775104	3.15E-02	7.84E-02	-4.759597445
Cdk4	0.499477256	8.464676801	4.345782289	2.94E-04	2.09E-03	-0.250437154
Hist1h2bn	0.499075982	10.43283997	3.220940903	4.17E-03	1.62E-02	-2.850981276
Trim59	0.496122415	7.628116091	2.167202915	4.21E-02	9.82E-02	-5.02323963
Set	0.490166327	7.539909817	4.046752288	5.98E-04	3.59E-03	-0.953338138
Prmt5	0.488977859	6.821657915	4.641379815	1.46E-04	1.20E-03	0.446119011
Acss1	0.487893598	7.290714487	2.632600048	1.57E-02	4.58E-02	-4.115122393
Mrpl28	0.487467583	7.413976397	3.171346913	4.67E-03	1.77E-02	-2.961280974
Atic	0.487389386	8.426220159	2.663574309	1.47E-02	4.36E-02	-4.051382224
Eif4e3	0.484172774	7.801185562	3.455745359	2.41E-03	1.05E-02	-2.321719666
Kif14	0.482650303	5.953497357	2.759417715	1.19E-02	3.69E-02	-3.851907883
Cfp	0.481959516	7.736438303	3.509524934	2.13E-03	9.60E-03	-2.199060317
Snrpd3	0.481769567	9.813793417	4.142807103	4.77E-04	3.02E-03	-0.727962025
Ipo9	0.480560136	7.331686966	3.283856922	3.60E-03	1.44E-02	-2.710258488
Magohb	0.480018876	7.119054119	3.471727815	2.32E-03	1.03E-02	-2.285317592
Nop56	0.47955896	8.628420096	7.042513186	6.60E-07	1.53E-05	5.886477925
Cenpe	0.479381054	6.877350117	4.130703304	4.90E-04	3.09E-03	-0.756388244
Raph1	0.478613744	7.024343745	5.033158516	5.83E-05	5.66E-04	1.366746349
Hyou1	0.478486081	7.762261065	2.87009042	9.27E-03	3.05E-02	-3.617613466
Nifk	0.477271853	7.641157929	3.166784316	4.72E-03	1.78E-02	-2.971399546
Pdia4	0.476020293	8.242289352	4.110945714	5.14E-04	3.22E-03	-0.802774134
Pole4	0.475893927	7.197415939	3.326152165	3.26E-03	1.35E-02	-2.615185745
G6pdx	0.475715939	8.026289978	2.650671116	1.51E-02	4.44E-02	-4.077979259
Rcc2	0.475004098	7.189731297	3.731023805	1.26E-03	6.49E-03	-1.689330542
Uqcr11	0.473534712	9.758105032	3.712690313	1.32E-03	6.67E-03	-1.73176623
Iqgap2	0.473421836	8.002293327	2.128793319	4.55E-02	1.04E-01	-5.093486624
Sfxn1	0.473385125	6.838366897	3.605334945	1.70E-03	8.09E-03	-1.979410608
Rpn1	0.473071167	7.897951793	4.209382974	4.07E-04	2.69E-03	-0.571489516
Apex1	0.472753309	7.938799624	3.466659106	2.35E-03	1.03E-02	-2.296866919
Rrm2	0.471632318	6.449233412	2.272116487	3.39E-02	8.33E-02	-4.827419724

Mndal	0.469006383	8.763007765	3.756235994	1.19E-03	6.19E-03	-1.630909473
Gm15987	0.467836098	9.173930624	3.975315647	7.09E-04	4.09E-03	-1.120589172
Knstrn	0.467732058	6.958623656	4.142792312	4.77E-04	3.02E-03	-0.727996767
Mrpl9	0.467529508	7.668590327	3.467723489	2.35E-03	1.03E-02	-2.294442026
Mrpl14	0.467088374	8.028208314	3.206204191	4.31E-03	1.66E-02	-2.88381575
Ndufaf2	0.46678699	7.725851644	5.342106841	2.84E-05	3.20E-04	2.087264532
Prps2	0.466323649	7.390724665	4.917250284	7.64E-05	6.99E-04	1.094992488
Anxa2	0.463861285	10.57800886	6.619804237	1.63E-06	3.31E-05	4.973774831
B4galt5	0.46290773	8.211726051	3.982502216	6.97E-04	4.04E-03	-1.103779569
Fkbp2	0.46242439	9.680905063	3.963782904	7.29E-04	4.17E-03	-1.147556669
Klf10	0.462423239	6.604197981	2.409969499	2.54E-02	6.61E-02	-4.561851717
Atp13a3	0.461203765	7.985150653	4.147677715	4.71E-04	3.01E-03	-0.716521248
Vdac1	0.460018307	9.240820141	5.041583661	5.71E-05	5.58E-04	1.386473191
Ctps	0.457815494	6.459737716	3.625189342	1.62E-03	7.85E-03	-1.933725922
Vav3	0.455615094	7.245556563	2.775665799	1.15E-02	3.58E-02	-3.817768016
Zfp36l2	0.455437453	8.750356119	4.491856992	2.08E-04	1.60E-03	0.093771884
Ttc27	0.454949148	6.09660865	3.175774578	4.62E-03	1.75E-02	-2.951456918
Tagln2	0.451485502	10.8969888	6.158900143	4.46E-06	7.28E-05	3.953075218
Prmt7	0.451191991	6.513351759	2.964946322	7.48E-03	2.57E-02	-3.413704756
Nmral1	0.451007134	6.393974288	2.463920553	2.26E-02	6.05E-02	-4.455525592
Kdelr2	0.450770262	10.16988095	6.108161299	5.00E-06	8.05E-05	3.839176253
Nucks1	0.44986481	8.753800926	5.792402015	1.02E-05	1.45E-04	3.124019435
Fam103a1	0.447285454	7.201749249	2.204983991	3.89E-02	9.27E-02	-4.953376443
Snrpd1	0.445703914	8.995198001	3.830421302	9.99E-04	5.40E-03	-1.458608837
Cry1	0.445353175	6.304864079	2.081893903	5.00E-02	1.12E-01	-5.178169187
Lta4h	0.442905744	8.071077853	4.532597254	1.89E-04	1.49E-03	0.189790427
Fanca	0.44243621	5.926878605	3.425862258	2.59E-03	1.12E-02	-2.38966381
Uck2	0.440771761	6.607527817	2.53270992	1.95E-02	5.40E-02	-4.318120576
Sssca1	0.440536513	7.475136068	4.238183054	3.80E-04	2.57E-03	-0.503747008
Smyd5	0.440068416	6.031133565	2.397129222	2.61E-02	6.74E-02	-4.586964655
Polr1e	0.438095297	6.046992013	3.370776043	2.94E-03	1.24E-02	-2.514489749
Snrnp25	0.437082335	9.09904644	3.068212466	5.92E-03	2.12E-02	-3.188751894
Ehd4	0.436670285	6.895175965	3.394912169	2.78E-03	1.19E-02	-2.459865997
Bzw2	0.436502418	7.594191642	2.685192627	1.40E-02	4.21E-02	-4.006681105
Klrd1	0.435254327	8.063558152	2.212680365	3.83E-02	9.14E-02	-4.939053142
Nup205	0.433863043	7.367519451	4.400329105	2.59E-04	1.88E-03	-0.121929295
Ubap2	0.43370218	8.716053896	4.667169132	1.38E-04	1.14E-03	0.506864217
Tomm5	0.433425736	7.026994105	2.142011221	4.43E-02	1.02E-01	-5.069402237
Anp32e	0.431637116	7.902049104	3.980589518	7.00E-04	4.05E-03	-1.108253794
Kars	0.431019694	9.035525158	5.833183665	9.26E-06	1.35E-04	3.216976017
Mthfd1	0.430585559	6.274285845	2.514237118	2.03E-02	5.57E-02	-4.355216588
B3gnt5	0.429683824	5.861956962	2.622733419	1.60E-02	4.66E-02	-4.135349269
Sdhc	0.428901964	8.447859503	3.52646383	2.04E-03	9.30E-03	-2.160328846
Naa10	0.428207427	7.4975416	2.109415756	4.73E-02	1.07E-01	-5.128622337
Dnajc25	0.428096798	6.764892148	2.648955865	1.52E-02	4.45E-02	-4.081510123
0610010K14Rik	0.427434664	7.984032003	2.858984065	9.51E-03	3.10E-02	-3.641306822
Padi2	0.426531133	6.734578723	2.83730201	9.98E-03	3.22E-02	-3.687447595
Rgs14	0.425558942	6.240981566	2.47566391	2.21E-02	5.94E-02	-4.432211897
Ube2s	0.42475329	10.14046106	3.19620089	4.41E-03	1.69E-02	-2.906075547
Emp3	0.424613412	9.850826433	3.859357987	9.33E-04	5.11E-03	-1.39125094
Mbnl3	0.424520975	7.314900856	3.578959395	1.81E-03	8.47E-03	-2.040014362
Lpcat4	0.423922199	8.087878002	4.360897359	2.84E-04	2.03E-03	-0.214831998
Nol11	0.42349914	7.123120373	2.525530531	1.98E-02	5.46E-02	-4.332554742
Aldh9a1	0.422969514	7.939045981	4.145190064	4.74E-04	3.02E-03	-0.72236473
Atf5	0.421912673	6.745089214	3.164507099	4.74E-03	1.79E-02	-2.976447924
Dis3	0.42013595	6.867589886	2.855843058	9.57E-03	3.12E-02	-3.64800045
Lbh	0.418351065	10.97657183	5.112435661	4.84E-05	4.89E-04	1.552211115
Pdia6	0.417655541	9.012691465	2.72802148	1.27E-02	3.91E-02	-3.917615445
Etfb	0.417614198	9.073510347	4.210707871	4.06E-04	2.68E-03	-0.568373796
Eef1e1	0.416577411	7.426649589	2.283681975	3.31E-02	8.17E-02	-4.805490908
Rpa2	0.415769291	7.274297115	2.545922828	1.90E-02	5.27E-02	-4.291500185
Athl1	0.415694095	6.309804941	2.648389956	1.52E-02	4.45E-02	-4.082674806
Smc3	0.414741259	9.351918591	5.827306923	9.38E-06	1.37E-04	3.20359115
Fasn	0.412079684	7.242576655	3.6456139	1.55E-03	7.54E-03	-1.886673097
Gspt1	0.412045836	8.182397338	2.765125882	1.17E-02	3.65E-02	-3.839924489
Isyna1	0.411697443	6.910294819	3.446408183	2.47E-03	1.07E-02	-2.342966023
Ruvbl2	0.408419927	7.877198794	3.293185036	3.52E-03	1.42E-02	-2.689322171
Dbf4	0.408158221	7.068987923	4.010524294	6.52E-04	3.86E-03	-1.038200028

Nccrp1	0.407477139	6.655291153	2.611434403	1.64E-02	4.75E-02	-4.158466202
Mrpl12	0.404789278	8.740856678	3.112552606	5.35E-03	1.97E-02	-3.091283436
Tmem50b	0.404508993	8.526738975	3.528184029	2.04E-03	9.27E-03	-2.156393008
Mycbp	0.403847053	7.872536434	2.993796193	7.01E-03	2.43E-02	-3.351158114
Zcchc24	0.403263095	6.169388032	2.433548092	2.41E-02	6.34E-02	-4.515542699
Lsm7	0.402798857	9.223519015	2.194454877	3.98E-02	9.42E-02	-4.97292173
Ndufaf4	0.402582286	6.9113349	2.272386697	3.39E-02	8.33E-02	-4.826908147
Tubgcp2	0.401627751	7.832134398	3.82687493	1.01E-03	5.43E-03	-1.466858406
Ostc	0.401422084	8.612321925	4.184809266	4.31E-04	2.80E-03	-0.629266436
Ercc6l	0.398298966	6.825063585	2.274195356	3.38E-02	8.31E-02	-4.823482962
Vdac3	0.397873378	7.494490208	2.256588842	3.50E-02	8.54E-02	-4.856756264
Nup62	0.397615435	10.83653911	5.346357218	2.82E-05	3.19E-04	2.097131156
Ak6	0.397579301	8.207344207	2.294698126	3.24E-02	8.02E-02	-4.78454224
Mthfd1l	0.397344367	6.283367817	2.123831083	4.60E-02	1.05E-01	-5.102503808
Nucb2	0.397283231	8.607871551	2.752172729	1.21E-02	3.75E-02	-3.867101273
Runx2	0.396163039	8.177669998	3.311971855	3.37E-03	1.38E-02	-2.647101615
Sqle	0.393371473	7.309741972	2.543384934	1.91E-02	5.30E-02	-4.296618935
Spc25	0.393280954	6.421271441	2.554167525	1.86E-02	5.21E-02	-4.274852979
Spcs3	0.392618767	9.383002655	5.363447486	2.71E-05	3.09E-04	2.136789729
Mybbp1a	0.392383635	7.535336552	3.135590456	5.07E-03	1.89E-02	-3.040444557
Heatr5a	0.391841281	7.427991262	4.674832368	1.35E-04	1.12E-03	0.524912141
L1cam	0.3909768	6.522586459	2.434546798	2.41E-02	6.33E-02	-4.51357571
Spdl1	0.389761246	7.63771356	4.225488166	3.92E-04	2.62E-03	-0.533611081
Copz1	0.38926556	8.832379727	3.729893715	1.27E-03	6.50E-03	-1.69194745
C1qbp	0.388654164	8.622356557	3.986576374	6.90E-04	4.03E-03	-1.094248329
Hsd17b12	0.388197784	7.968276626	3.288484886	3.56E-03	1.44E-02	-2.699873617
Cope	0.387340673	9.429892984	3.719654297	1.30E-03	6.60E-03	-1.715651692
H1f0	0.386994176	9.316212284	3.11178037	5.36E-03	1.97E-02	-3.092985271
Twf2	0.386891343	8.735840362	3.115356319	5.31E-03	1.96E-02	-3.085103421
Ptpro	0.384313036	6.646402938	2.50931451	2.05E-02	5.62E-02	-4.365077739
Lman1	0.383600573	8.068961671	2.109857417	4.73E-02	1.07E-01	-5.127823807
Cdca7l	0.381541618	6.795128755	2.491510891	2.13E-02	5.79E-02	-4.400656914
Dna2	0.381376147	7.774422339	2.249033623	3.56E-02	8.64E-02	-4.870986666
Arhgdib	0.379937565	10.57902611	5.886490394	8.21E-06	1.23E-04	3.338225055
Mar2	0.378385197	8.710773797	4.521160741	1.94E-04	1.53E-03	0.162836686
Ppp2r4	0.378253132	7.941475074	2.79688514	1.09E-02	3.45E-02	-3.773046608
Cluh	0.377729197	6.697306366	2.131278408	4.53E-02	1.04E-01	-5.088965774
Hist1h2bl	0.377608925	10.89743868	3.792331885	1.09E-03	5.80E-03	-1.547146264
Whsc1	0.376334997	8.342371218	4.795659675	1.02E-04	8.81E-04	0.809290982
Iars	0.376288715	8.07428721	4.499257636	2.05E-04	1.58E-03	0.111214229
Lyrm4	0.376015724	7.306610585	2.623099402	1.60E-02	4.66E-02	-4.134599665
Fkbp3	0.37557873	8.858164502	3.890518526	8.67E-04	4.80E-03	-1.318629479
Nolc1	0.374791055	8.129528095	3.070953072	5.88E-03	2.11E-02	-3.182742283
Zfp414	0.37455072	8.581325737	3.493727612	2.21E-03	9.88E-03	-2.235140099
Ept1	0.373863053	7.671363457	3.28581944	3.58E-03	1.44E-02	-2.705855265
Ndufc1	0.373197892	9.677172367	3.404569313	2.72E-03	1.16E-02	-2.437980015
Hpse	0.373134207	7.745905087	3.425860705	2.59E-03	1.12E-02	-2.389667337
Snrpa1	0.371671019	8.645691761	2.953220541	7.69E-03	2.63E-02	-3.439057551
Thrap3	0.371233217	9.223363338	2.309738897	3.14E-02	7.82E-02	-4.755844246
Flna	0.370924055	9.697425211	4.09885295	5.29E-04	3.29E-03	-0.831154992
Prdx1	0.370864113	8.674054576	2.151523855	4.34E-02	1.00E-01	-5.052010781
Anxa1	0.370625885	8.531892019	3.469819402	2.34E-03	1.03E-02	-2.289666522
Mri1	0.370589145	6.586380692	2.346411377	2.90E-02	7.37E-02	-4.685414626
Syne2	0.370495932	6.554340539	2.927336081	8.15E-03	2.75E-02	-3.494879859
Noc2l	0.370110846	8.100415156	3.772720243	1.15E-03	6.03E-03	-1.592674052
Naa50	0.369941467	9.873600921	5.671494813	1.34E-05	1.82E-04	2.847456797
Prmt3	0.369843773	6.812243127	3.871427681	9.07E-04	5.00E-03	-1.363132234
Cd3eap	0.369675769	6.299352577	3.116251519	5.30E-03	1.96E-02	-3.083129782
Kti12	0.368723279	7.035665745	2.815838872	1.05E-02	3.36E-02	-3.732971818
Gatc	0.365737456	7.404293477	2.342224689	2.93E-02	7.41E-02	-4.693487676
Uchl3	0.363947202	8.201967752	2.676946584	1.42E-02	4.28E-02	-4.023752314
Clns1a	0.363555191	8.521679665	2.87559195	9.16E-03	3.03E-02	-3.605862495
Sigmar1	0.363274322	7.008433043	2.599241773	1.69E-02	4.84E-02	-4.183355377
Ipo5	0.363120452	8.195369606	3.116271401	5.30E-03	1.96E-02	-3.083085946
Tuba4a	0.363058122	9.091270957	3.694873976	1.38E-03	6.88E-03	-1.772966341
Frrs1	0.36301682	6.398355441	2.616576195	1.63E-02	4.71E-02	-4.147952648
Rbm38	0.362159271	8.380431812	3.061457327	6.01E-03	2.14E-02	-3.203556124
Dbi	0.36002393	9.875933835	3.750374801	1.21E-03	6.24E-03	-1.644497321

Dpagt1	0.359583493	8.444745631	4.411885948	2.52E-04	1.84E-03	-0.094697097
2700029M09Rik	0.358432702	8.698459774	4.407069034	2.55E-04	1.85E-03	-0.106047709
Ttc37	0.357725067	6.787021524	3.072752979	5.86E-03	2.10E-02	-3.178794368
Srsf1	0.356770342	8.999450196	3.089117895	5.64E-03	2.05E-02	-3.142860766
Prss30	0.355463887	6.017274107	2.375229668	2.73E-02	7.01E-02	-4.629621315
Acaa2	0.354575927	7.408279496	3.247554762	3.92E-03	1.55E-02	-2.791560133
Ltv1	0.353823094	7.799079234	2.675269197	1.43E-02	4.28E-02	-4.027221806
Hdac9	0.353719615	5.968537578	2.789565397	1.11E-02	3.49E-02	-3.788490808
Rfc1	0.352307949	7.965233286	3.018392697	6.63E-03	2.33E-02	-3.297646638
Dtymk	0.352217131	7.80674548	2.572369921	1.79E-02	5.05E-02	-4.238001702
Tcf4	0.35084356	8.23638794	2.79880799	1.09E-02	3.44E-02	-3.768986516
Cxcr4	0.350546899	8.758485541	3.545357042	1.96E-03	9.04E-03	-2.11707573
Casp1	0.350103115	8.469603928	4.130573575	4.91E-04	3.09E-03	-0.75669288
Sf3b5	0.350076904	9.730834822	4.872463515	8.49E-05	7.54E-04	0.989823154
Oaz1	0.349263813	9.50504057	2.445672498	2.35E-02	6.23E-02	-4.491633257
Serp1	0.348797427	8.719306807	3.536795835	2.00E-03	9.14E-03	-2.136682145
Txndc5	0.34862913	7.398265358	2.24036081	3.62E-02	8.75E-02	-4.887286528
Tmpo	0.348595669	8.733536691	4.652328707	1.43E-04	1.17E-03	0.471909915
Ict1	0.346426099	8.004206762	2.553459307	1.87E-02	5.22E-02	-4.276284063
Tram1	0.345940068	9.937451896	4.362787424	2.83E-04	2.03E-03	-0.210379469
Mif	0.34510199	10.93337379	2.952310949	7.70E-03	2.63E-02	-3.441022536
Trmt112	0.344947533	8.077911018	2.255358782	3.51E-02	8.55E-02	-4.859075065
Ldlr	0.344613527	7.415915726	3.014264947	6.69E-03	2.35E-02	-3.306638638
Manf	0.343682142	7.494804591	3.689003484	1.40E-03	6.94E-03	-1.786533294
Ccnd1	0.343138575	9.549727089	3.788378189	1.10E-03	5.83E-03	-1.556327932
Hspa9	0.342217454	8.686977268	5.326537682	2.95E-05	3.29E-04	2.051111329
Prelid1	0.341626341	9.648274455	3.218916989	4.19E-03	1.62E-02	-2.855493624
Map2k3	0.340310152	7.959959013	2.941010482	7.90E-03	2.68E-02	-3.465414508
Ndufb6	0.340040849	9.80983814	3.672128971	1.45E-03	7.17E-03	-1.825507024
Slc35c1	0.339893628	7.324697813	2.580372557	1.76E-02	4.99E-02	-4.221757778
Rell1	0.339647378	7.604468128	2.468125266	2.24E-02	6.02E-02	-4.447185006
Snx30	0.338103216	7.282775689	2.599816681	1.69E-02	4.83E-02	-4.182183115
Tbrg4	0.335304299	7.34630806	2.560269989	1.84E-02	5.16E-02	-4.262513354
Ftsj3	0.333587249	7.27137769	2.22542001	3.73E-02	8.94E-02	-4.91527686
Anapc5	0.333199882	9.669393313	4.092899825	5.36E-04	3.31E-03	-0.845123678
Gars	0.332962154	8.946203026	4.41750127	2.48E-04	1.82E-03	-0.081464809
Me2	0.332841625	8.91666079	4.053940384	5.88E-04	3.54E-03	-0.936490562
Rbfa	0.332566897	7.665539823	2.804312087	1.07E-02	3.42E-02	-3.757357766
Fh1	0.332221343	8.201755828	2.935493851	8.00E-03	2.71E-02	-3.477308364
Larp7	0.332048591	7.61286599	3.207726257	4.29E-03	1.66E-02	-2.880426763
Mrpl33	0.331994293	9.323604793	3.90891215	8.30E-04	4.63E-03	-1.275721815
Pcna	0.329874502	10.98498825	4.149743697	4.69E-04	3.00E-03	-0.711668044
Noa1	0.329780916	7.191941466	2.47747227	2.20E-02	5.93E-02	-4.428616505
Fkbp4	0.329701509	8.024314456	2.12846708	4.55E-02	1.04E-01	-5.094079866
Ak2	0.329064282	8.734626125	3.525034117	2.05E-03	9.32E-03	-2.163599694
Hcfc1	0.327712423	8.900663113	3.921156976	8.06E-04	4.54E-03	-1.247141836
I830077J02Rik	0.327435882	8.557607012	2.708457517	1.33E-02	4.04E-02	-3.958382643
Alg8	0.327060661	8.296483449	3.00641224	6.81E-03	2.38E-02	-3.323732118
Hprt	0.326951445	8.745739891	3.328452334	3.25E-03	1.34E-02	-2.610004892
Cct6a	0.32671889	8.910795952	3.833091428	9.93E-04	5.38E-03	-1.452396775
Prep	0.326586303	7.648154436	2.719049461	1.30E-02	3.97E-02	-3.936328328
Anp32b	0.32604625	9.530335772	2.633165438	1.57E-02	4.58E-02	-4.11396219
Ptpn12	0.325404363	8.38912839	3.805983348	1.06E-03	5.66E-03	-1.515430932
Mrps12	0.325213318	8.937340406	2.960336597	7.56E-03	2.59E-02	-3.42367642
Tmem147	0.324625267	7.728351434	3.096205311	5.55E-03	2.02E-02	-3.12727691
Pitpna	0.323747855	9.622120839	3.545920395	1.95E-03	9.04E-03	-2.115785174
Klf3	0.322292867	9.146957941	2.573203722	1.79E-02	5.05E-02	-4.236310435
Exosc1	0.322022929	7.806353045	4.199354558	4.17E-04	2.73E-03	-0.595070864
2810428I15Rik	0.320636075	8.847852183	3.895820817	8.56E-04	4.75E-03	-1.306263572
Il17ra	0.320180025	8.688023485	2.805725985	1.07E-02	3.41E-02	-3.754368923
Bak1	0.319712553	10.2180028	3.458293661	2.40E-03	1.05E-02	-2.315918503
Dcps	0.319367704	7.928724614	3.282015758	3.62E-03	1.45E-02	-2.714388698
Gramd4	0.319260559	7.314102902	2.919569669	8.29E-03	2.79E-02	-3.511589796
Txn2	0.319209109	8.461302864	2.508641737	2.06E-02	5.62E-02	-4.366424668
Plp2	0.317179105	10.66565248	2.447523551	2.34E-02	6.21E-02	-4.487977231
Psip1	0.315921776	8.807663079	4.194834351	4.21E-04	2.75E-03	-0.605698647
Immt	0.315375489	8.491685294	3.226712635	4.11E-03	1.60E-02	-2.83810805
Cct3	0.313377762	8.072976821	3.542747519	1.97E-03	9.08E-03	-2.123053112

Tomm6	0.312464892	10.08375717	3.451094372	2.44E-03	1.06E-02	-2.332304677
Ndufs6	0.312174913	10.93200461	3.184942031	4.53E-03	1.72E-02	-2.931101559
Zfp367	0.309995748	7.734106861	2.425403294	2.46E-02	6.43E-02	-4.531567545
Smim4	0.309401695	7.683069322	2.185359696	4.05E-02	9.53E-02	-4.98975854
Pcyt1a	0.306760632	7.701833456	2.500403584	2.09E-02	5.69E-02	-4.382902385
Emg1	0.306729946	8.994635374	4.170589694	4.46E-04	2.89E-03	-0.66268786
Gnl3	0.306376655	8.009938235	2.522259734	2.00E-02	5.49E-02	-4.339123558
Nrros	0.306237629	8.923187298	4.788036056	1.04E-04	8.93E-04	0.791360117
Slc25a12	0.306113638	8.382688905	3.966178796	7.24E-04	4.16E-03	-1.141955069
Nceh1	0.304946836	6.857937413	2.170207399	4.18E-02	9.77E-02	-5.017711419
Eif2s1	0.303032228	8.71498893	3.343987626	3.13E-03	1.30E-02	-2.574985945
Ssrp1	0.302842317	7.813491905	2.428030534	2.44E-02	6.40E-02	-4.526401707
Smc6	0.302810926	9.43683015	3.460283098	2.39E-03	1.05E-02	-2.311388812
Arl6ip5	0.302649665	8.633088863	3.129284605	5.14E-03	1.91E-02	-3.054373148
Sass6	0.30258786	7.387408582	2.314855192	3.10E-02	7.76E-02	-4.746057204
Atrnl1	0.302315897	7.620135965	2.112294871	4.70E-02	1.06E-01	-5.123414909
Idh3g	0.301421483	9.883362579	4.022025349	6.35E-04	3.78E-03	-1.011268757
Phpt1	0.300672363	8.603423087	3.147943154	4.93E-03	1.85E-02	-3.013131138
Lrpprc	0.300535099	7.295646962	3.070853442	5.88E-03	2.11E-02	-3.182960788
Gng10	0.300111077	9.579819918	2.449309227	2.33E-02	6.20E-02	-4.484448898
Uqcrq	0.30011006	9.983952704	2.684912734	1.40E-02	4.21E-02	-4.007260961
Srebf2	0.297838644	9.669078356	4.340732724	2.98E-04	2.12E-03	-0.262331002
Cpsf2	0.297549321	8.289097167	3.247745719	3.92E-03	1.55E-02	-2.791133216
Pigt	0.295959185	8.614754473	2.903941208	8.59E-03	2.88E-02	-3.545159964
Wdr3	0.295063861	7.507355817	3.365924315	2.98E-03	1.25E-02	-2.525456656
Heatr1	0.294540061	7.506136066	3.362624812	3.00E-03	1.26E-02	-2.53291232
Slc35b1	0.294199696	8.988351259	2.364452291	2.80E-02	7.12E-02	-4.650532282
E2f4	0.293987422	8.31906059	3.080985495	5.75E-03	2.08E-02	-3.160726361
Stt3b	0.293468477	7.959908335	2.435978569	2.40E-02	6.32E-02	-4.51075501
Ndufb7	0.292177645	10.66961468	3.785961951	1.11E-03	5.86E-03	-1.561938351
Ndufaf7	0.291076809	7.349962194	2.405013685	2.57E-02	6.66E-02	-4.571553162
Smdt1	0.290566473	10.4821889	3.593505035	1.75E-03	8.26E-03	-2.006604806
Nsfl1c	0.290015389	9.625860581	3.904502886	8.38E-04	4.67E-03	-1.286010153
Noc4l	0.289444524	6.679838326	2.587435558	1.73E-02	4.94E-02	-4.207399831
Rpn2	0.289057975	8.796089204	3.107505194	5.41E-03	1.98E-02	-3.10240407
Etfdh	0.28695587	8.40496869	2.466169895	2.25E-02	6.04E-02	-4.451064693
Arhgap11a	0.28683143	8.113835898	2.463991551	2.26E-02	6.05E-02	-4.455384824
Hmgcs1	0.285788348	8.270747815	2.470119316	2.23E-02	6.00E-02	-4.443226862
Ssh2	0.284312601	8.003402261	3.080114202	5.76E-03	2.08E-02	-3.162639435
Lsm4	0.284044677	9.095897235	2.658158199	1.48E-02	4.39E-02	-4.06255397
Alkbh6	0.283537271	7.731434324	2.524816997	1.98E-02	5.46E-02	-4.333988128
Rbl1	0.283392328	8.340940102	3.656624828	1.51E-03	7.40E-03	-1.861283741
Cops6	0.283190262	9.62590851	2.367098069	2.78E-02	7.10E-02	-4.645403788
Rexo2	0.281972597	8.982996381	2.741916791	1.23E-02	3.82E-02	-3.888577495
Ints7	0.281405535	7.460114385	2.942672159	7.87E-03	2.68E-02	-3.461830159
Ndufa9	0.280790794	8.730402779	3.956841619	7.41E-04	4.23E-03	-1.163782959
Tspo	0.279408486	9.315990539	2.368852967	2.77E-02	7.08E-02	-4.642000344
Prkd3	0.278998244	7.348369237	2.817680301	1.04E-02	3.35E-02	-3.729072018
Ube2l3	0.278501959	9.562109471	2.171004953	4.18E-02	9.76E-02	-5.016243125
Eif3b	0.278290758	8.430748391	3.322777342	3.29E-03	1.35E-02	-2.6227852
Tiam1	0.278065758	7.418402708	2.540700792	1.92E-02	5.32E-02	-4.30202977
Cse1l	0.277114179	8.834863432	3.62210318	1.63E-03	7.88E-03	-1.940830713
Ahsa1	0.275906575	9.544638363	2.083100711	4.99E-02	1.12E-01	-5.176005448
Psmc5	0.275584148	10.19100178	3.234444646	4.04E-03	1.58E-02	-2.820850946
Nat10	0.275350813	6.550623892	2.315312972	3.10E-02	7.76E-02	-4.74518089
Coro7	0.274623219	8.811223326	2.318827518	3.08E-02	7.73E-02	-4.738449743
Plekhg3	0.274158741	6.186037572	2.11838975	4.65E-02	1.05E-01	-5.112376123
Arl6ip4	0.273761365	9.221707545	3.320841761	3.30E-03	1.36E-02	-2.627142724
Cebpg	0.273693404	8.705414741	2.947793657	7.78E-03	2.65E-02	-3.450777601
Rsl1d1	0.273399719	9.146633681	3.750606508	1.21E-03	6.24E-03	-1.643960234
Nars	0.27334719	9.679719553	3.966090111	7.25E-04	4.16E-03	-1.142162422
Cldn25	0.273145905	8.85698214	2.190096357	4.02E-02	9.47E-02	-4.980995545
Mrpl16	0.272487171	7.787653503	2.994334211	7.00E-03	2.43E-02	-3.349989433
Vps36	0.272421694	8.234588843	3.513492209	2.11E-03	9.53E-03	-2.189993069
Depdc5	0.27198895	7.623876108	2.451798234	2.32E-02	6.18E-02	-4.479528502
Snrpb	0.271874873	9.35665934	2.129231227	4.55E-02	1.04E-01	-5.09269023
Dhx9	0.271119004	9.51333576	4.095694799	5.33E-04	3.30E-03	-0.838565661
Smc4	0.270807669	9.806815031	3.210839073	4.26E-03	1.65E-02	-2.873494217

Ppil3	0.269885381	7.533747424	2.240244021	3.62E-02	8.75E-02	-4.887505765
Ppa1	0.268974172	7.430259083	2.662322929	1.47E-02	4.36E-02	-4.053964412
Parp1	0.268417713	7.974468963	2.39832406	2.60E-02	6.73E-02	-4.58463097
Pik3cg	0.267238297	8.49155148	2.246469314	3.58E-02	8.68E-02	-4.875810047
Rad21	0.266872309	10.09108726	3.7136359	1.32E-03	6.67E-03	-1.729578496
Tcerg1	0.26636513	8.936153765	3.609937294	1.68E-03	8.05E-03	-1.968825547
Ythdf1	0.265852388	8.93689419	3.074862351	5.83E-03	2.10E-02	-3.17416659
Ndufb8	0.265130565	10.15583138	2.591139485	1.72E-02	4.91E-02	-4.199862394
Park7	0.264429907	9.845097238	3.43951999	2.51E-03	1.08E-02	-2.358630116
Ctsc	0.264299379	8.773481176	2.491826986	2.13E-02	5.79E-02	-4.400026399
Timm8b	0.264267374	9.250817445	4.470583346	2.19E-04	1.65E-03	0.043633046
Abce1	0.263785434	8.300199417	2.505086298	2.07E-02	5.65E-02	-4.373539683
Eif3l	0.261039202	9.860297056	3.516132054	2.10E-03	9.48E-03	-2.183958287
Coro1c	0.260900357	9.102477004	2.616595635	1.63E-02	4.71E-02	-4.147912879
Abcb7	0.260695869	7.258104094	2.268336824	3.42E-02	8.38E-02	-4.834571772
Hnrnpab	0.25725721	10.99523167	3.946090537	7.60E-04	4.33E-03	-1.188907934
Mfsd5	0.257224851	8.610843334	2.716963408	1.30E-02	3.98E-02	-3.940675061
Cep128	0.257162307	8.110778769	2.186953395	4.04E-02	9.50E-02	-4.986811454
Tmem173	0.25694095	8.723526935	2.640929568	1.54E-02	4.52E-02	-4.098017465
Thyn1	0.256388451	7.329347585	2.384603582	2.68E-02	6.90E-02	-4.611389569
Nudc	0.25623189	8.840562761	2.228162382	3.71E-02	8.90E-02	-4.910147802
Hn1	0.255062753	9.344102646	2.608424812	1.66E-02	4.78E-02	-4.164615194
Coro1a	0.252210775	10.25338072	2.671446398	1.44E-02	4.31E-02	-4.035124932
Ywhae	0.250356088	11.21763741	4.077692693	5.56E-04	3.42E-03	-0.880797518
Rars	0.250332308	8.947238163	3.093796044	5.58E-03	2.03E-02	-3.132575871
Gmps	0.249590161	9.116218908	4.253075677	3.67E-04	2.50E-03	-0.468706028
Nol9	0.249557085	7.573180472	2.234782954	3.66E-02	8.81E-02	-4.897749493
Nelfa	0.248997923	8.747676154	3.014349802	6.69E-03	2.35E-02	-3.306453834
Cct5	0.248671105	10.7254308	4.020884657	6.36E-04	3.78E-03	-1.013940233
Wars	0.248380021	8.463867807	2.729794135	1.27E-02	3.90E-02	-3.913914825
Taf12	0.246426336	8.797012875	2.346542956	2.90E-02	7.37E-02	-4.685160771
Utp18	0.246274765	7.967755028	2.743621993	1.23E-02	3.81E-02	-3.885009316
Tfg	0.246103911	8.811251717	2.799818797	1.09E-02	3.44E-02	-3.766851703
Pitrm1	0.245565198	7.567220741	2.534461185	1.94E-02	5.38E-02	-4.314596406
Ube2d1	0.245526896	9.52977387	3.207260272	4.30E-03	1.66E-02	-2.881464368
Clic1	0.245195048	10.45592137	2.412882567	2.52E-02	6.57E-02	-4.556143932
Canx	0.24517686	10.65018817	3.609052772	1.68E-03	8.05E-03	-1.970860112
Eif4ebp2	0.244320835	9.611668832	2.86849909	9.30E-03	3.06E-02	-3.621010679
Uhrf1bp1l	0.242074242	7.864060128	2.528168459	1.97E-02	5.43E-02	-4.32725368
Dek	0.241479887	10.45619108	3.328059136	3.25E-03	1.34E-02	-2.610890598
Sec31a	0.241358293	8.937880557	3.059450424	6.04E-03	2.15E-02	-3.207952027
Slc7a6	0.240327892	7.448704038	2.161198986	4.26E-02	9.90E-02	-5.034272388
Ptpn11	0.239602711	7.607147497	2.116284748	4.67E-02	1.05E-01	-5.116190919
Eprs	0.237946135	8.440869468	2.809943744	1.06E-02	3.39E-02	-3.745449022
Atf6	0.237491524	8.572314359	2.651304949	1.51E-02	4.44E-02	-4.076674226
Ift20	0.237379823	8.572077564	2.270805866	3.40E-02	8.34E-02	-4.829900546
Smarcc1	0.234518464	8.736918209	3.193791239	4.43E-03	1.70E-02	-2.91143416
Tma7	0.233897262	10.09237539	2.938869836	7.94E-03	2.69E-02	-3.470030817
Alcam	0.233526236	9.274051067	3.441221756	2.50E-03	1.08E-02	-2.354760986
Cd164	0.233197173	10.1362609	2.16319756	4.24E-02	9.86E-02	-5.030601961
BC037034	0.232134646	7.008524925	2.146754806	4.39E-02	1.01E-01	-5.060735879
Ddi2	0.231367872	8.796848232	2.164466208	4.23E-02	9.85E-02	-5.028270956
Atp2a2	0.231302456	9.080327385	3.013209097	6.71E-03	2.35E-02	-3.308937971
Psmc6	0.231036163	9.389233189	3.360083101	3.02E-03	1.27E-02	-2.538654224
Spin1	0.231028803	8.764168481	2.386615742	2.67E-02	6.88E-02	-4.607470734
Msmo1	0.230298981	7.744015209	2.459680848	2.28E-02	6.10E-02	-4.463927779
Ssr3	0.229237664	9.385962918	2.426560752	2.45E-02	6.41E-02	-4.529292062
Exosc3	0.229167693	9.461928811	2.653226398	1.50E-02	4.43E-02	-4.072717115
Sec13	0.227369836	9.409369633	3.407769978	2.70E-03	1.16E-02	-2.430722574
Bccip	0.22604136	8.906753681	2.6756239	1.43E-02	4.28E-02	-4.02648823
Kat2a	0.22580397	7.175666915	2.424866484	2.46E-02	6.43E-02	-4.532622673
Srsf7	0.22573893	10.59160666	2.296123971	3.23E-02	8.00E-02	-4.781826449
Dpy19l1	0.225355664	9.071452102	2.380161041	2.70E-02	6.95E-02	-4.620035137
Sp100	0.220785814	9.233526579	2.320802849	3.06E-02	7.71E-02	-4.734663922
Exoc6	0.219439084	8.708559095	2.652746741	1.50E-02	4.43E-02	-4.073705071
Trappc1	0.21868089	9.005984639	2.120178167	4.63E-02	1.05E-01	-5.109133151
Vamp3	0.217692757	8.919361864	2.448915897	2.34E-02	6.20E-02	-4.485226203
Herpud1	0.217138615	10.36268195	3.241928351	3.97E-03	1.56E-02	-2.804135386

Gtf3c6	0.21517111	9.184720912	2.865977082	9.36E-03	3.07E-02	-3.626393083
Crcp	0.214996209	8.317228019	2.584128252	1.75E-02	4.97E-02	-4.214125533
Ndufa12	0.2148107	10.32803259	2.234237712	3.67E-02	8.81E-02	-4.898771411
Rnf144b	0.214653689	8.56536867	2.81000273	1.06E-02	3.39E-02	-3.745324233
5031439G07Rik	0.21435574	7.97473599	2.305556018	3.16E-02	7.87E-02	-4.763836304
Psmd2	0.213985064	9.26655604	2.198849148	3.94E-02	9.36E-02	-4.964771633
Pet100	0.212475464	8.335572868	2.151556786	4.34E-02	1.00E-01	-5.051950491
Necap2	0.212032308	9.496200585	2.199518666	3.94E-02	9.35E-02	-4.963528987
Ssr2	0.211058132	9.606735776	3.183827209	4.54E-03	1.73E-02	-2.933577958
Psmb7	0.211040685	10.36739486	2.123743772	4.60E-02	1.05E-01	-5.102662344
Esyt1	0.210677989	8.290483069	2.407923569	2.55E-02	6.63E-02	-4.565858159
Magt1	0.210220167	9.137929854	2.745291741	1.22E-02	3.80E-02	-3.881514336
Psmb4	0.209580757	10.6951349	3.308552769	3.40E-03	1.39E-02	-2.654790901
Vmp1	0.208789574	7.727168419	2.316895703	3.09E-02	7.74E-02	-4.742150343
Rsu1	0.208159895	10.5514756	2.441220756	2.38E-02	6.26E-02	-4.500419713
Ncl	0.207784338	10.46013762	3.036439016	6.36E-03	2.25E-02	-3.258279008
Ipo7	0.205498899	8.769932159	2.429695936	2.43E-02	6.38E-02	-4.523125492
Eif5a	0.203904757	6.773670585	2.10867574	4.74E-02	1.07E-01	-5.129960061
Ptbp1	0.202373998	10.03914115	2.318015588	3.08E-02	7.73E-02	-4.740005302
AI662270	0.202049672	9.907195292	2.134951503	4.49E-02	1.03E-01	-5.082277567
Sdcbp	0.20176103	9.312618541	2.871994539	9.23E-03	3.04E-02	-3.613547451
Trp53	0.201364167	9.398256355	2.462085752	2.27E-02	6.07E-02	-4.459162739
Hint1	0.20046875	10.34433811	2.141464007	4.43E-02	1.02E-01	-5.070401197
Psmd14	0.198541382	9.610959104	2.924512328	8.20E-03	2.77E-02	-3.50095745
Mapk3	0.19530461	9.212088447	2.736365796	1.25E-02	3.85E-02	-3.900185986
Rnf130	0.194762816	9.432565763	2.275137789	3.37E-02	8.30E-02	-4.821697566
Cct8	0.194583389	9.589623708	2.644910395	1.53E-02	4.48E-02	-4.089833337
Aatf	0.193961076	8.245625066	2.370055094	2.76E-02	7.07E-02	-4.639668116
Nup153	0.193187133	9.173346848	2.863629616	9.41E-03	3.08E-02	-3.631401169
Strap	0.191830713	8.470731268	2.130575736	4.53E-02	1.04E-01	-5.09024441
Psma2	0.191149577	9.536831707	2.106654631	4.76E-02	1.07E-01	-5.133612069
Hnrnpd	0.190921933	10.52192222	2.56574271	1.82E-02	5.11E-02	-4.251434233
Snx2	0.189315461	9.013788488	2.244207503	3.59E-02	8.71E-02	-4.880061677
Psmd1	0.188813899	9.012356677	3.084437865	5.70E-03	2.06E-02	-3.153144145
Actb	0.188295725	13.26639215	2.091219331	4.91E-02	1.10E-01	-5.161428055
P4hb	0.186207094	9.255836332	2.154971668	4.31E-02	1.00E-01	-5.045695309
Mdh1	0.185323758	9.74781267	2.25453953	3.52E-02	8.56E-02	-4.860619023
Nup210	0.184907242	9.562463156	3.065215743	5.96E-03	2.13E-02	-3.195320852
Atp5k	0.184515472	11.68008413	2.382619624	2.69E-02	6.92E-02	-4.615251648
Dad1	0.184048261	9.404591649	2.124340166	4.59E-02	1.05E-01	-5.101579339
Sgk3	0.183485158	8.801436768	2.196370389	3.96E-02	9.39E-02	-4.969370251
Slk	0.182108159	8.967968012	3.267153194	3.74E-03	1.49E-02	-2.74770298
Efr3a	0.18080687	8.622091223	2.675734004	1.43E-02	4.28E-02	-4.026260508
Emc3	0.17985032	8.90399068	2.639411308	1.55E-02	4.52E-02	-4.101137241
Crip1	0.176624508	12.09455551	2.264100794	3.45E-02	8.43E-02	-4.84257891
Srp72	0.17300497	9.516169769	2.316793932	3.09E-02	7.74E-02	-4.742345246
Bag1	0.172064008	10.25254322	2.616050044	1.63E-02	4.71E-02	-4.149028958
Ptbp3	0.170012602	10.38102999	3.336428554	3.19E-03	1.32E-02	-2.59203124
Pgls	0.169633373	9.883998196	2.093072935	4.89E-02	1.10E-01	-5.15809466
Mtpn	0.166923188	10.54419486	2.59127525	1.72E-02	4.91E-02	-4.19958601
Mdh2	0.16551996	10.42819064	2.441944332	2.37E-02	6.25E-02	-4.498992179
Atp5a1	0.165176808	10.95232479	2.716511282	1.31E-02	3.98E-02	-3.941616956
Phka2	0.16110006	8.569577961	2.224112274	3.74E-02	8.96E-02	-4.917721361
Fubp1	0.159031828	9.131630988	2.369805678	2.76E-02	7.07E-02	-4.640152059
Fam49b	0.157343567	9.838358785	2.232395325	3.68E-02	8.84E-02	-4.902223373
Atp5g3	0.156955098	10.21215774	2.147143796	4.38E-02	1.01E-01	-5.060024672
Gltp	0.156348375	10.48633967	2.153831382	4.32E-02	1.00E-01	-5.047784714
S100a6	0.15229055	11.84529172	2.396093998	2.61E-02	6.75E-02	-4.588986063
Akr1a1	0.151487943	11.07702221	2.5561746	1.85E-02	5.19E-02	-4.270796203
Stk17b	0.151348874	10.30237153	2.312249012	3.12E-02	7.80E-02	-4.751044192
Clint1	0.15082664	10.23952167	2.565914956	1.82E-02	5.11E-02	-4.251085335
Eif4g1	0.147900938	9.280453241	2.1394601	4.45E-02	1.02E-01	-5.074058018
Psma7	0.14609856	11.25333926	2.384178632	2.68E-02	6.90E-02	-4.612216952
Stt3a	0.144018125	9.738726591	2.198147546	3.95E-02	9.37E-02	-4.966073578
Etf1	0.136778326	9.315167535	2.17651619	4.13E-02	9.67E-02	-5.006087738
Dnajc3	0.132697878	10.05251622	2.294361386	3.24E-02	8.02E-02	-4.785183479
Atp5b	0.129232975	10.94094313	2.097063151	4.85E-02	1.09E-01	-5.150912442
Sf3b1	-0.115407075	11.08099994	-2.21461151	3.82E-02	9.11E-02	-4.935454381

Supt20	-0.129608564	9.797387864	-2.422537473	2.47E-02	6.45E-02	-4.537198967
Dync1h1	-0.134589052	9.555243113	-2.238897284	3.63E-02	8.76E-02	-4.890033348
Nckap1l	-0.142247357	10.57264742	-2.471460408	2.23E-02	5.99E-02	-4.440563853
Samhd1	-0.142699805	11.46015402	-2.261836957	3.46E-02	8.46E-02	-4.846854443
Rab21	-0.1443026	10.29103406	-2.149958514	4.36E-02	1.01E-01	-5.054875962
Cat	-0.14664251	10.46107331	-2.187732932	4.04E-02	9.50E-02	-4.98536944
Ctss	-0.146645417	11.37669948	-2.850627973	9.68E-03	3.14E-02	-3.659107048
Npc2	-0.148787522	11.23210372	-2.29845401	3.21E-02	7.97E-02	-4.777386308
Eif3g	-0.149781013	10.57812097	-2.141582734	4.43E-02	1.02E-01	-5.07018447
Mapre2	-0.152853036	8.412625988	-2.152144588	4.34E-02	1.00E-01	-5.050874234
Map4k4	-0.157655687	8.980275759	-2.267389153	3.42E-02	8.39E-02	-4.836363882
Fam129a	-0.158206552	9.094217316	-2.345080665	2.91E-02	7.38E-02	-4.687981495
Nup98	-0.158300611	9.509503916	-2.450943607	2.33E-02	6.18E-02	-4.48121828
Nfatc1	-0.161404097	8.262931748	-2.119252771	4.64E-02	1.05E-01	-5.110811408
Camta2	-0.163790955	8.880773249	-2.448768183	2.34E-02	6.20E-02	-4.4855181
Ptprc	-0.163926082	11.0398556	-2.152674559	4.33E-02	1.00E-01	-5.049903709
Pcmtd1	-0.164375717	8.975362427	-2.178393972	4.11E-02	9.64E-02	-5.002623937
Mef2a	-0.165647627	9.717840895	-2.500787177	2.09E-02	5.69E-02	-4.382135771
Itpripl2	-0.16784432	9.173486037	-2.345601676	2.91E-02	7.38E-02	-4.686976593
H2-D1	-0.168166547	12.72114069	-2.166128176	4.22E-02	9.83E-02	-5.02521597
Sri	-0.169017042	10.83247243	-2.60750575	1.66E-02	4.78E-02	-4.166492253
Itch	-0.169267107	9.243875922	-2.167116897	4.21E-02	9.82E-02	-5.023397831
Usp3	-0.169688065	8.932321997	-2.495265046	2.12E-02	5.75E-02	-4.393165744
Mtf1	-0.170341611	8.110609612	-2.40382498	2.57E-02	6.66E-02	-4.573878497
St3gal1	-0.170582184	9.559838168	-2.771457551	1.16E-02	3.61E-02	-3.826618965
Smarca2	-0.170784007	8.972771849	-2.483511381	2.17E-02	5.87E-02	-4.416599282
Il10rb	-0.170859667	8.656377767	-2.097489445	4.85E-02	1.09E-01	-5.150144608
Inpp5d	-0.173442687	9.803473566	-2.191216547	4.01E-02	9.45E-02	-4.978921431
Esyt2	-0.176126439	9.266199166	-2.090964491	4.91E-02	1.10E-01	-5.161886191
Zfr	-0.177902769	9.659222493	-2.23643954	3.65E-02	8.79E-02	-4.894643716
Rab43	-0.18063821	9.169934332	-2.881208034	9.04E-03	3.01E-02	-3.593857015
Ifi30	-0.181383073	11.59780226	-2.949076163	7.76E-03	2.65E-02	-3.448008647
Bcl6	-0.18168915	10.3768874	-2.581609164	1.76E-02	4.99E-02	-4.219245394
Mtmr4	-0.182141281	9.287022832	-2.904178573	8.59E-03	2.88E-02	-3.54465066
Apobec3	-0.182398972	10.4048234	-2.892063014	8.82E-03	2.94E-02	-3.570624406
Ifngr1	-0.18295225	9.772967925	-2.488684623	2.15E-02	5.82E-02	-4.406292569
Vti1b	-0.18302858	9.753453867	-2.241574028	3.61E-02	8.74E-02	-4.885008676
Skap2	-0.183570193	9.940936205	-3.012812016	6.71E-03	2.35E-02	-3.309802617
Aftph	-0.183990575	9.902087221	-2.255526603	3.51E-02	8.55E-02	-4.858758748
Sema4a	-0.184321938	9.022452447	-2.235895314	3.65E-02	8.80E-02	-4.895664188
Otud5	-0.185869819	8.958872794	-2.61915438	1.62E-02	4.69E-02	-4.142677088
Myo1g	-0.186155188	10.208374	-2.191821809	4.00E-02	9.44E-02	-4.977800471
Mir3064	-0.186895442	12.24381025	-2.728369937	1.27E-02	3.91E-02	-3.91688809
Arhgap30	-0.187917435	10.07460151	-3.020350618	6.60E-03	2.33E-02	-3.293379804
Kdm6a	-0.188841323	9.730908967	-2.532174987	1.95E-02	5.40E-02	-4.319196797
Dapp1	-0.188993015	9.459491782	-2.986647278	7.12E-03	2.46E-02	-3.366679178
Pias1	-0.189083743	9.815255319	-2.605407503	1.67E-02	4.79E-02	-4.170776391
Man2a1	-0.189824818	8.488955669	-2.301551013	3.19E-02	7.93E-02	-4.771480526
Spop	-0.1903746	10.86286836	-2.75650747	1.19E-02	3.72E-02	-3.858013128
Eya3	-0.192199665	9.194532742	-2.608299523	1.66E-02	4.78E-02	-4.164871099
Lyst	-0.192498209	9.716795656	-2.128447927	4.55E-02	1.04E-01	-5.094114692
Kdm7a	-0.19327116	9.319035151	-3.245074721	3.94E-03	1.55E-02	-2.797103981
Heca	-0.194507943	8.539183651	-2.277180644	3.36E-02	8.27E-02	-4.817825957
Kif2a	-0.194968014	9.878273478	-2.605210595	1.67E-02	4.79E-02	-4.171178344
Flt3	-0.195801805	10.87706901	-2.630720979	1.58E-02	4.60E-02	-4.118977435
Polr2g	-0.195883567	10.38476024	-2.157584505	4.29E-02	9.96E-02	-5.040905045
Itgb1	-0.196329578	10.05490668	-2.580447058	1.76E-02	4.99E-02	-4.221606432
Wnk1	-0.196477629	10.63201125	-2.390417063	2.65E-02	6.83E-02	-4.600062283
Adrbk2	-0.196930427	9.214031684	-2.668719163	1.45E-02	4.33E-02	-4.040759793
2700060E02Rik	-0.198464515	10.32676512	-2.28842576	3.28E-02	8.10E-02	-4.796477295
App	-0.198853301	9.084715326	-2.793090033	1.10E-02	3.47E-02	-3.781056305
Sh3glb1	-0.199076276	10.1358786	-2.954235304	7.67E-03	2.63E-02	-3.436865079
Jarid2	-0.199434057	8.235004869	-2.403490385	2.57E-02	6.66E-02	-4.574532915
P2rx4	-0.200763178	8.693934776	-2.18323213	4.07E-02	9.56E-02	-4.993690775
Ypel5	-0.201255602	8.941647997	-2.365036322	2.79E-02	7.12E-02	-4.649400494
Tbc1d1	-0.201430413	8.531929633	-2.568106586	1.81E-02	5.09E-02	-4.246644986
Myo9a	-0.202335515	8.440630997	-2.188641434	4.03E-02	9.49E-02	-4.983688459
Kansl3	-0.204014266	8.04951119	-2.942187937	7.88E-03	2.68E-02	-3.462874744

Dip2b	-0.204635878	8.799551663	-2.423691067	2.47E-02	6.44E-02	-4.534932563
Itga4	-0.204796542	10.21937184	-2.736891458	1.25E-02	3.85E-02	-3.899087166
Foxn2	-0.205270146	9.011833285	-2.906309328	8.55E-03	2.87E-02	-3.540078002
Uvrag	-0.205396195	10.56590961	-2.502646754	2.08E-02	5.68E-02	-4.378418511
Trpv2	-0.205738925	8.86267612	-2.961406237	7.54E-03	2.59E-02	-3.421363148
Trappc11	-0.205988653	8.115889079	-2.404515708	2.57E-02	6.66E-02	-4.57252738
Mgea5	-0.206695605	9.508741585	-2.568653122	1.81E-02	5.09E-02	-4.245537375
Lrrc8c	-0.208600289	9.874346184	-2.146271554	4.39E-02	1.01E-01	-5.06161932
Marcks	-0.208709837	8.350355368	-2.566934414	1.81E-02	5.10E-02	-4.249020105
Vgll4	-0.210522902	8.185339691	-2.163580787	4.24E-02	9.86E-02	-5.029897914
Irf5	-0.211985164	9.022943291	-2.480943097	2.18E-02	5.89E-02	-4.42171183
Arid1b	-0.212703049	9.019683648	-2.682327807	1.41E-02	4.23E-02	-4.012614796
Gpbp1	-0.21367662	9.698651061	-2.924733257	8.20E-03	2.77E-02	-3.500482029
Themis2	-0.214677743	9.674419917	-2.231104168	3.69E-02	8.86E-02	-4.904641499
Stat6	-0.215463899	8.903458899	-2.606199571	1.66E-02	4.79E-02	-4.169159374
Man2b1	-0.216921763	10.38462222	-3.234200864	4.04E-03	1.58E-02	-2.821395248
Arf4	-0.218897945	9.83413132	-3.387520816	2.83E-03	1.20E-02	-2.476605364
Dhx57	-0.219688552	8.625237736	-2.19958219	3.94E-02	9.35E-02	-4.963411071
Usp47	-0.221041668	9.226235512	-3.545295156	1.96E-03	9.04E-03	-2.117217499
Traf6	-0.221701647	8.470601645	-3.00024567	6.91E-03	2.41E-02	-3.337143202
Rtn1	-0.221749292	7.328068351	-2.41783586	2.50E-02	6.51E-02	-4.546429773
Atmin	-0.221895348	8.517875431	-2.475795271	2.21E-02	5.94E-02	-4.431950771
2810474O19Rik	-0.222559783	9.376350358	-2.982627391	7.19E-03	2.48E-02	-3.3754004
Evl	-0.223280281	8.696234512	-2.369079678	2.77E-02	7.08E-02	-4.641560557
Wrn	-0.223361107	8.562542319	-2.710969174	1.32E-02	4.02E-02	-3.953156591
Usp6nl	-0.223386126	8.452063577	-2.263088713	3.46E-02	8.44E-02	-4.844490665
Os9	-0.22519235	9.088481992	-2.63433632	1.56E-02	4.57E-02	-4.111559101
Kmt2a	-0.225358075	9.195752649	-2.816594893	1.05E-02	3.36E-02	-3.731370844
Fyn	-0.228730408	8.513394063	-2.268394833	3.42E-02	8.38E-02	-4.834462059
Rspry1	-0.229204036	7.898636647	-2.375049525	2.73E-02	7.01E-02	-4.629971285
Kdm1b	-0.22977495	7.899149508	-2.77280179	1.15E-02	3.60E-02	-3.823792372
Pik3cb	-0.230064573	9.726493434	-3.292927306	3.53E-03	1.42E-02	-2.689900874
H2-Eb1	-0.230949597	11.71711714	-3.28803702	3.57E-03	1.44E-02	-2.700878796
Jak2	-0.231132398	10.864644	-3.461249716	2.38E-03	1.05E-02	-2.309187704
Lamp1	-0.231367369	10.05206597	-2.525686765	1.98E-02	5.46E-02	-4.332240861
1700021K19Rik	-0.231448197	7.968984152	-2.464876967	2.26E-02	6.05E-02	-4.453629101
Aim2	-0.232415364	8.790212108	-2.144516012	4.41E-02	1.01E-01	-5.064827588
Hbp1	-0.233624249	8.964966265	-2.717179604	1.30E-02	3.98E-02	-3.940224645
Gdi1	-0.233766312	9.975047132	-2.509865503	2.05E-02	5.62E-02	-4.363974478
Rabgap1	-0.234369817	8.381419137	-2.799688573	1.09E-02	3.44E-02	-3.767126754
Fbxw11	-0.235810369	8.57744409	-2.863640151	9.41E-03	3.08E-02	-3.631378697
Aebp2	-0.235950927	9.918539978	-2.265711037	3.44E-02	8.40E-02	-4.839536213
Ccni	-0.237974117	9.916304015	-2.192500319	4.00E-02	9.44E-02	-4.976543625
Zbtb20	-0.23819622	7.557193019	-2.572671538	1.79E-02	5.05E-02	-4.237389939
Arl4c	-0.238408046	8.834541836	-2.809067848	1.06E-02	3.39E-02	-3.747301893
Glce	-0.240540041	8.171378452	-2.234570783	3.66E-02	8.81E-02	-4.898147171
Atp6v1d	-0.240877951	9.889857474	-3.920037377	8.08E-04	4.55E-03	-1.249755548
Sidt2	-0.24090244	8.444553943	-2.321769514	3.06E-02	7.70E-02	-4.732810575
Nub1	-0.241027393	8.86451565	-3.295722691	3.50E-03	1.42E-02	-2.683623433
Plekhm2	-0.241124167	7.419033372	-2.138493521	4.46E-02	1.02E-01	-5.075821101
Plekha5	-0.241865007	7.540841404	-2.340273228	2.94E-02	7.42E-02	-4.697247771
Sdf2	-0.242738414	8.922772906	-3.035481259	6.38E-03	2.25E-02	-3.260370569
Tspan33	-0.242803674	8.268463452	-2.403634504	2.57E-02	6.66E-02	-4.574251046
Snapc3	-0.243464914	8.713171147	-4.005845507	6.59E-04	3.89E-03	-1.049153496
N4bp1	-0.244093927	8.261647606	-2.79534474	1.10E-02	3.46E-02	-3.776298258
Cep63	-0.244296427	8.581992374	-3.8758406	8.97E-04	4.96E-03	-1.352848158
Lrch1	-0.245703024	9.643352394	-2.798988519	1.09E-02	3.44E-02	-3.768605266
Hps5	-0.245962352	8.54779371	-2.581600531	1.76E-02	4.99E-02	-4.219262935
Pgap2	-0.246639166	8.369613482	-2.327509202	3.02E-02	7.61E-02	-4.72179685
Mta3	-0.247591396	7.926485239	-3.985985745	6.91E-04	4.03E-03	-1.095630143
Cstb	-0.249573647	9.342205419	-2.547978622	1.89E-02	5.26E-02	-4.287351854
Ankib1	-0.250096829	8.592629792	-3.059249643	6.04E-03	2.15E-02	-3.208391758
Adam17	-0.251477097	8.865466685	-3.23313529	4.05E-03	1.58E-02	-2.823774241
Taf9b	-0.251804628	7.502412357	-2.301033806	3.19E-02	7.93E-02	-4.772467132
Stat3	-0.252118816	10.09440044	-3.661427563	1.49E-03	7.35E-03	-1.850204469
Fbxl5	-0.252145113	8.614672768	-2.864494159	9.39E-03	3.08E-02	-3.629556956
Myo9b	-0.252390381	9.257832393	-2.6290246	1.58E-02	4.61E-02	-4.122456508
Eif2ak4	-0.252912261	7.262947738	-2.240771218	3.62E-02	8.75E-02	-4.886516059

Pold4	-0.253005423	9.388089589	-2.486596543	2.16E-02	5.84E-02	-4.410454059
Taz	-0.253136973	8.720949005	-2.791813711	1.10E-02	3.48E-02	-3.783748932
Tsc22d1	-0.253235739	7.729004776	-2.66271563	1.47E-02	4.36E-02	-4.053154148
Pus10	-0.25388693	8.177980789	-2.510404557	2.05E-02	5.61E-02	-4.362894997
Akap13	-0.254806488	9.638277845	-3.379012064	2.89E-03	1.22E-02	-2.495862688
Pdlim2	-0.255024578	7.51362227	-2.442387322	2.37E-02	6.25E-02	-4.498118097
Rala	-0.255599842	9.548555386	-3.268106196	3.74E-03	1.49E-02	-2.745568251
Vps39	-0.256565168	8.885415411	-2.573801112	1.79E-02	5.05E-02	-4.23509853
Sipa1l1	-0.256714916	7.79716476	-2.871448748	9.24E-03	3.04E-02	-3.614713038
Zbtb38	-0.257722854	6.478478237	-2.434288267	2.41E-02	6.33E-02	-4.514084939
Agap1	-0.258080303	7.730620302	-2.280149496	3.33E-02	8.23E-02	-4.812195715
4930523C07Rik	-0.258425025	9.101364274	-2.849914749	9.70E-03	3.14E-02	-3.660625327
Atp7a	-0.258763502	8.017220413	-2.199890188	3.94E-02	9.35E-02	-4.962839329
Trim5	-0.258934104	8.616767148	-2.111834373	4.71E-02	1.06E-01	-5.124248114
Pik3r1	-0.259770412	9.733766561	-3.759645617	1.18E-03	6.16E-03	-1.62300327
Btg2	-0.260276713	10.48640989	-4.299089492	3.29E-04	2.30E-03	-0.360397793
Sepsecs	-0.261265743	8.368442376	-2.442086586	2.37E-02	6.25E-02	-4.498711502
Gmpr2	-0.26148856	8.833886938	-2.872216753	9.23E-03	3.04E-02	-3.613072867
Mvp	-0.261918188	9.193361843	-3.115708354	5.31E-03	1.96E-02	-3.084327316
Fbrs	-0.26204293	9.848601574	-2.646610017	1.52E-02	4.47E-02	-4.086337266
Peak1	-0.26244262	9.454094552	-2.876980926	9.13E-03	3.02E-02	-3.602894208
Gns	-0.262549552	9.523041536	-3.673356862	1.45E-03	7.16E-03	-1.822672274
3110043O21Rik	-0.262604704	8.813981224	-2.874032251	9.19E-03	3.03E-02	-3.609194897
Gramd1b	-0.262784884	8.16803985	-2.896880247	8.73E-03	2.91E-02	-3.560302486
Sirt2	-0.265132667	8.828188085	-3.243848237	3.95E-03	1.55E-02	-2.799845146
Ptpn22	-0.265276126	8.721069116	-2.13105181	4.53E-02	1.04E-01	-5.08937814
Foxj2	-0.265448789	9.117275917	-2.498472022	2.10E-02	5.71E-02	-4.386761671
Gm10865	-0.265475592	6.550821236	-3.47332851	2.32E-03	1.03E-02	-2.281669422
Rcsd1	-0.26609048	8.986713566	-3.254091623	3.86E-03	1.53E-02	-2.776941281
Nedd4	-0.266251952	9.783911036	-2.889916322	8.87E-03	2.95E-02	-3.575221822
Sat1	-0.266954462	11.63745037	-3.295387694	3.51E-03	1.42E-02	-2.684375803
Cdk12	-0.268565044	9.35971897	-2.607696584	1.66E-02	4.78E-02	-4.166102528
Baz2b	-0.268612618	9.542936428	-2.987334164	7.11E-03	2.46E-02	-3.365188504
Nedd4l	-0.269322479	7.9162906	-3.54227929	1.97E-03	9.08E-03	-2.124125529
Malt1	-0.269420735	9.825356768	-3.181616568	4.56E-03	1.73E-02	-2.938487681
Snx11	-0.27020912	7.075745421	-2.520563844	2.00E-02	5.50E-02	-4.342527691
Smim24	-0.270974754	7.752642103	-2.850903829	9.68E-03	3.14E-02	-3.658519776
Mbp	-0.272811105	7.195870688	-3.324026089	3.28E-03	1.35E-02	-2.619973528
Evi2a	-0.273714168	9.687468554	-2.697609765	1.36E-02	4.12E-02	-3.98092746
Cmc1	-0.273882935	8.080299865	-2.826713785	1.02E-02	3.29E-02	-3.709924577
1500012F01Rik	-0.273924784	9.987463355	-3.657190599	1.50E-03	7.40E-03	-1.859978739
Bsdc1	-0.274877798	8.808291864	-3.148120444	4.93E-03	1.85E-02	-3.012738855
Btg1	-0.275657914	10.30754286	-2.308918319	3.14E-02	7.83E-02	-4.757412761
Tmem168	-0.275817937	8.538883319	-4.284403998	3.41E-04	2.36E-03	-0.394971198
Dnase1l1	-0.276378252	8.219977996	-2.667223369	1.46E-02	4.34E-02	-4.043849139
Cd86	-0.276587473	10.07374991	-3.086408691	5.68E-03	2.06E-02	-3.148814362
Il18bp	-0.276720347	7.578878356	-3.591274738	1.76E-03	8.27E-03	-2.011729506
Tapbpl	-0.278678942	9.474355929	-3.359487345	3.02E-03	1.27E-02	-2.539999898
Hivep1	-0.279436208	9.831987263	-2.311852798	3.12E-02	7.80E-02	-4.751802069
H2-T22	-0.280067791	9.143150084	-2.186865088	4.04E-02	9.50E-02	-4.986974788
Tiparp	-0.280333497	8.440486327	-2.40859443	2.55E-02	6.62E-02	-4.564544655
Bcor	-0.280670091	8.238829559	-2.812311906	1.06E-02	3.38E-02	-3.740438128
Cd44	-0.280747441	8.384105976	-2.557071184	1.85E-02	5.19E-02	-4.268983462
Asl	-0.280970038	8.374243822	-2.31127296	3.13E-02	7.80E-02	-4.752911043
Zmym5	-0.281541645	8.621768466	-3.790593948	1.10E-03	5.82E-03	-1.551182478
Idnk	-0.282296952	9.334638783	-3.098304227	5.52E-03	2.02E-02	-3.122659327
Ctsz	-0.283027501	10.31717075	-3.5346916	2.01E-03	9.16E-03	-2.141499422
Ncoa2	-0.283260366	9.6950241	-2.857873264	9.53E-03	3.11E-02	-3.643674349
Dnajc1	-0.28346812	8.245227644	-3.181531298	4.56E-03	1.73E-02	-2.938677038
Jak1	-0.284257835	10.09292527	-3.846541536	9.62E-04	5.25E-03	-1.421094526
2410006H16Rik	-0.284544242	11.29789839	-4.214452835	4.02E-04	2.67E-03	-0.55956653
Abhd16a	-0.284629792	7.781922627	-2.278321067	3.35E-02	8.25E-02	-4.815663733
Kdm2b	-0.285009458	7.73830249	-3.122397803	5.23E-03	1.94E-02	-3.069573699
Atp6ap2	-0.285957588	8.144918206	-3.245078354	3.94E-03	1.55E-02	-2.797095862
Card11	-0.286073036	7.6039283	-2.123302112	4.60E-02	1.05E-01	-5.10346424
Aff1	-0.28619047	8.930080265	-3.601855377	1.71E-03	8.14E-03	-1.987411381
Gusb	-0.2876856	9.733924042	-3.991359618	6.82E-04	3.99E-03	-1.083056713
Mtmr1	-0.28827589	8.654608545	-4.051315056	5.92E-04	3.56E-03	-0.942644216

D730005E14Rik	-0.288404545	7.485929021	-2.443738128	2.36E-02	6.25E-02	-4.495452224
Snhg12	-0.28963134	10.12606681	-3.59208643	1.75E-03	8.27E-03	-2.009864511
Arhgef2	-0.2912857	8.722576086	-3.739644241	1.24E-03	6.38E-03	-1.66936364
Parp3	-0.291891948	7.370260213	-3.23131262	4.07E-03	1.59E-02	-2.827842933
Ppfibp2	-0.292671954	7.483509246	-2.253845729	3.52E-02	8.57E-02	-4.861926295
Pnpla2	-0.29328571	8.020311527	-3.151698054	4.89E-03	1.84E-02	-3.004821187
Mthfd2	-0.293829627	9.437257988	-2.787337253	1.12E-02	3.50E-02	-3.793188452
Dennd3	-0.294063646	8.145258638	-2.193130451	3.99E-02	9.44E-02	-4.975376176
Zfp652	-0.294358199	8.087079624	-2.430187094	2.43E-02	6.38E-02	-4.522159039
Arid4a	-0.29541168	9.500167141	-3.8167037	1.03E-03	5.54E-03	-1.490511802
B4galt6	-0.295955394	7.616353071	-2.507747268	2.06E-02	5.62E-02	-4.368215151
Klf6	-0.297409588	9.597114818	-3.326006053	3.27E-03	1.35E-02	-2.615514808
Pstpip1	-0.297558905	8.561788219	-3.605171789	1.70E-03	8.09E-03	-1.9797858
Hcst	-0.298209061	8.750234596	-2.861397031	9.45E-03	3.09E-02	-3.636162543
Stx16	-0.298731158	8.670860697	-2.803640693	1.08E-02	3.42E-02	-3.758776795
Glcci1	-0.299117722	7.947170583	-2.528112964	1.97E-02	5.43E-02	-4.32736523
Mllt6	-0.299161994	7.819269665	-2.862046623	9.44E-03	3.09E-02	-3.634777339
Stat1	-0.29917864	10.24231808	-3.472015063	2.32E-03	1.03E-02	-2.284662952
Trafd1	-0.300173882	9.98069212	-3.546772069	1.95E-03	9.04E-03	-2.113834029
Vrk2	-0.302510508	8.359128097	-2.552733071	1.87E-02	5.22E-02	-4.277751344
Krit1	-0.302666175	8.286597696	-3.864950912	9.21E-04	5.06E-03	-1.378222794
Dusp11	-0.303657665	9.632591157	-3.682657084	1.42E-03	7.03E-03	-1.801195272
Tbc1d9	-0.30425725	9.653421178	-3.704197486	1.35E-03	6.79E-03	-1.751410617
Traf2	-0.305343415	7.593529067	-3.146835616	4.94E-03	1.85E-02	-3.01558157
Ndrg1	-0.305708992	7.647247118	-2.65427376	1.50E-02	4.42E-02	-4.070559546
Zfand6	-0.306287402	9.453075983	-3.042892046	6.27E-03	2.22E-02	-3.244180318
Il2rg	-0.306297759	10.20092691	-3.253754528	3.86E-03	1.53E-02	-2.77769538
Cpd	-0.307275585	7.138861168	-3.693117713	1.38E-03	6.90E-03	-1.777025585
Tspan13	-0.309148416	10.03267696	-3.536592471	2.00E-03	9.14E-03	-2.137147743
Dnajb9	-0.309339675	9.582822841	-3.1225387	5.22E-03	1.94E-02	-3.069262828
Gtpbp2	-0.309438041	8.02549402	-4.313983208	3.18E-04	2.24E-03	-0.325328661
Pex13	-0.309606053	7.690457477	-2.501830604	2.09E-02	5.68E-02	-4.38005016
Stoml1	-0.309614038	7.596365082	-3.259862599	3.81E-03	1.51E-02	-2.764027508
Acvr2a	-0.309897662	7.846643838	-3.844299069	9.67E-04	5.27E-03	-1.426314595
Bmp2k	-0.30992619	10.63906269	-3.829371859	1.00E-03	5.41E-03	-1.46105018
Arhgap25	-0.310042188	8.000823728	-2.550001394	1.88E-02	5.25E-02	-4.283268466
Use1	-0.3101026	11.05664512	-3.197212836	4.40E-03	1.69E-02	-2.903824767
Ptk2b	-0.310735811	8.310747496	-2.974955344	7.32E-03	2.52E-02	-3.39203222
Atf3	-0.310825557	9.381658997	-2.482213196	2.18E-02	5.88E-02	-4.419183866
Map4	-0.310946592	8.686825366	-3.629206782	1.61E-03	7.78E-03	-1.924475245
Arap1	-0.312149663	7.212967377	-3.118611505	5.27E-03	1.95E-02	-3.077925778
Myb	-0.313346317	8.313497545	-3.462788642	2.37E-03	1.04E-02	-2.30568305
Lrrk1	-0.314237228	9.569106877	-4.058380825	5.82E-04	3.53E-03	-0.926081415
Clic4	-0.314786255	10.61398822	-3.563133333	1.88E-03	8.77E-03	-2.076329788
Atp6v0c-ps2	-0.31576878	9.775275537	-2.650077236	1.51E-02	4.44E-02	-4.079201895
Kynu	-0.315921804	9.500901259	-4.570612207	1.73E-04	1.38E-03	0.2793797
Gyg	-0.316392143	9.883210275	-3.031531748	6.43E-03	2.27E-02	-3.268992921
Trps1	-0.316419555	8.058831658	-3.581258311	1.80E-03	8.45E-03	-2.034736073
Aff3	-0.316703234	7.048278479	-2.534637419	1.94E-02	5.38E-02	-4.31424169
Flot1	-0.317075404	7.623460251	-2.136549142	4.48E-02	1.03E-01	-5.079366198
Agpat3	-0.317337795	7.987993737	-2.928863102	8.12E-03	2.75E-02	-3.491592236
S100pbp	-0.31842281	8.08926657	-3.924796668	7.99E-04	4.53E-03	-1.238644235
Tgoln1	-0.318432105	9.000143253	-3.886793719	8.74E-04	4.84E-03	-1.327314939
Cd36	-0.318576045	7.516305538	-2.157650919	4.29E-02	9.96E-02	-5.040783236
Peli1	-0.318803923	9.674905003	-3.439438564	2.51E-03	1.08E-02	-2.358815233
Dock9	-0.31909815	5.981866566	-2.34091903	2.94E-02	7.41E-02	-4.696003632
Ccnl1	-0.319795688	10.23467425	-4.390227252	2.65E-04	1.92E-03	-0.145731712
Clip1	-0.319914356	8.90853155	-3.32138457	3.30E-03	1.36E-02	-2.625920789
Zfp366	-0.320010886	9.313859423	-3.288012995	3.57E-03	1.44E-02	-2.700932715
Slc25a38	-0.3201759	7.544225489	-4.201712012	4.14E-04	2.73E-03	-0.589527769
Tle3	-0.321194831	9.386325261	-3.352724246	3.07E-03	1.29E-02	-2.555271356
Snora31	-0.32184384	10.00919895	-3.262744091	3.78E-03	1.50E-02	-2.757576867
Tap2	-0.322635443	9.052288971	-4.351703385	2.90E-04	2.07E-03	-0.236489869
Lipa	-0.322644936	8.20974465	-3.019005167	6.62E-03	2.33E-02	-3.296312016
Aplp2	-0.322915062	8.942225291	-4.221390003	3.95E-04	2.64E-03	-0.5432506
Sh3pxd2b	-0.323276414	6.489092709	-2.217656166	3.79E-02	9.06E-02	-4.929776642
Cep350	-0.323653677	9.30341734	-4.722877805	1.21E-04	1.02E-03	0.638036828
Lysmd1	-0.3237179	8.534767984	-2.118495408	4.64E-02	1.05E-01	-5.11218458

L3mbtl3	-0.324020548	6.502710149	-2.314153098	3.11E-02	7.77E-02	-4.747401001
Sik2	-0.324744609	8.169961277	-3.263766033	3.77E-03	1.50E-02	-2.755288669
Creg1	-0.325665235	9.204069351	-4.199727608	4.16E-04	2.73E-03	-0.594193722
Fam13b	-0.327304242	8.972103211	-4.527696631	1.91E-04	1.51E-03	0.178240625
Kif16b	-0.328083523	7.455063103	-2.68989086	1.38E-02	4.17E-02	-3.996943444
Fam53b	-0.329341425	7.776168672	-3.148087073	4.93E-03	1.85E-02	-3.012812695
Tnfrsf18	-0.330531025	8.740497798	-2.772041677	1.15E-02	3.61E-02	-3.825390772
Limd2	-0.332469046	8.929954048	-3.245869177	3.93E-03	1.55E-02	-2.795328213
Uba7	-0.33259696	9.028587374	-4.491328112	2.09E-04	1.60E-03	0.092525386
Dennd4a	-0.333619647	9.615690285	-4.042424151	6.05E-04	3.62E-03	-0.963480956
Kdm6b	-0.334041167	8.228598405	-2.622229998	1.61E-02	4.66E-02	-4.136380287
Rgs10	-0.334813706	7.703669139	-3.371790482	2.94E-03	1.24E-02	-2.512196131
Cdc42ep3	-0.335494849	9.148667672	-3.190095439	4.47E-03	1.71E-02	-2.91965031
Lix1l	-0.336108863	7.033023825	-2.362190798	2.81E-02	7.15E-02	-4.654913293
Rasip1	-0.336313402	6.153926783	-2.659891379	1.48E-02	4.38E-02	-4.058980161
D930015E06Rik	-0.336321398	10.40792802	-4.199714152	4.16E-04	2.73E-03	-0.594225362
Hgd	-0.336817895	5.382994489	-2.165835955	4.22E-02	9.83E-02	-5.02575323
Ubash3b	-0.336922212	7.660277122	-2.897055274	8.73E-03	2.91E-02	-3.559927317
Gramd1a	-0.337061173	8.32618794	-3.74774834	1.21E-03	6.27E-03	-1.650584923
St14	-0.337113771	6.100646941	-2.236657091	3.65E-02	8.79E-02	-4.894235745
Ncoa7	-0.337976316	8.252287709	-3.390044819	2.81E-03	1.20E-02	-2.470890337
Nup93	-0.338526329	8.17604901	-4.780580627	1.05E-04	9.05E-04	0.773823068
Ier5	-0.338630803	9.458613193	-3.764092408	1.17E-03	6.13E-03	-1.612690154
Dstn	-0.341116453	9.509330842	-4.040371432	6.08E-04	3.63E-03	-0.968291014
Rabgef1	-0.341283384	8.215492019	-2.865710447	9.36E-03	3.07E-02	-3.626962009
Gramd2	-0.341513438	4.99572626	-2.343210626	2.92E-02	7.40E-02	-4.691587275
Vwa5a	-0.343321287	9.159014234	-4.689012985	1.31E-04	1.10E-03	0.558306222
Eml6	-0.34413973	7.435132559	-3.284822449	3.59E-03	1.44E-02	-2.708092277
Ifi27	-0.344345493	8.284228093	-2.888783775	8.89E-03	2.96E-02	-3.577646738
Mif4gd	-0.344364264	7.642641722	-2.99650844	6.97E-03	2.42E-02	-3.345265746
Zbtb37	-0.345308759	7.221477456	-2.655041539	1.49E-02	4.42E-02	-4.068977656
Cep70	-0.346881635	7.049002245	-2.397695132	2.61E-02	6.74E-02	-4.585859435
AW549877	-0.346961076	8.216257921	-4.015443165	6.45E-04	3.83E-03	-1.026682909
H2-K1	-0.347033213	11.6412621	-4.79074956	1.03E-04	8.89E-04	0.797742531
Rhbdf2	-0.347402004	6.90086526	-2.911224147	8.45E-03	2.84E-02	-3.529525369
Fmnl2	-0.347483672	9.424976127	-4.671236775	1.36E-04	1.13E-03	0.51644419
Sdhaf1	-0.347979333	8.451404962	-3.34599031	3.12E-03	1.30E-02	-2.570468115
Crispld2	-0.34859788	6.203753672	-2.123302192	4.60E-02	1.05E-01	-5.103464095
Ogt	-0.351026092	9.621394033	-3.315217568	3.35E-03	1.37E-02	-2.639800028
Ttyh3	-0.354028629	9.764569633	-5.317355674	3.01E-05	3.34E-04	2.029781214
Cdk17	-0.355550892	8.28047828	-2.841971914	9.87E-03	3.19E-02	-3.677522577
Gm3650	-0.356282483	8.347979439	-2.529593322	1.96E-02	5.42E-02	-4.324389132
Nfkb2	-0.356380595	9.151319051	-4.440479387	2.35E-04	1.75E-03	-0.027314829
Il10ra	-0.356877725	9.539337693	-4.095031725	5.34E-04	3.30E-03	-0.840121511
Sgpl1	-0.357586941	9.122644762	-4.873541819	8.47E-05	7.54E-04	0.992356216
Naip5	-0.358495477	8.110632216	-2.291890841	3.25E-02	8.04E-02	-4.789886329
Nfkbie	-0.359196929	9.770662543	-4.166172699	4.51E-04	2.91E-03	-0.673067733
A530032D15Rik	-0.360211684	9.332967056	-3.135057746	5.08E-03	1.89E-02	-3.041621607
Thbs4	-0.36115946	5.005515343	-2.176959288	4.12E-02	9.66E-02	-5.005270557
Gsap	-0.361688746	9.214472884	-3.595847999	1.74E-03	8.23E-03	-2.001220459
Kdm5b	-0.362144564	7.352220996	-3.617764906	1.65E-03	7.94E-03	-1.950815836
Fbxw17	-0.362320909	7.49126675	-3.453516035	2.43E-03	1.06E-02	-2.32679377
Atp8a1	-0.364365644	8.247189182	-4.162039557	4.55E-04	2.94E-03	-0.682779779
Dnmbp	-0.365245847	6.875853083	-2.341751696	2.93E-02	7.41E-02	-4.69439921
Gtpbp1	-0.367840396	9.612021093	-3.469542394	2.34E-03	1.03E-02	-2.290297724
Dennd1c	-0.369201414	8.407239784	-3.054467339	6.11E-03	2.17E-02	-3.218862299
Msi2	-0.371467883	8.694893584	-3.954046504	7.46E-04	4.25E-03	-1.170315922
Tbc1d8	-0.373521098	10.30723254	-3.973752813	7.12E-04	4.09E-03	-1.124244189
Dram2	-0.373945805	7.549955651	-2.639400628	1.55E-02	4.52E-02	-4.101159184
Gsdmd	-0.374376026	9.18949689	-4.912290836	7.73E-05	7.06E-04	1.083350748
Scai	-0.375481827	7.448338072	-4.493761373	2.07E-04	1.60E-03	0.098260259
Fuca1	-0.376153697	10.70535348	-4.957980442	6.95E-05	6.55E-04	1.190560415
Katna1	-0.37818511	9.570122243	-2.814401377	1.05E-02	3.37E-02	-3.736015381
Neat1	-0.378859818	10.1294555	-3.609048798	1.68E-03	8.05E-03	-1.970869253
Mcemp1	-0.379416645	8.716568471	-4.214113561	4.02E-04	2.67E-03	-0.560364443
Tpp1	-0.380268151	7.566488397	-3.286565165	3.58E-03	1.44E-02	-2.704181899
Epb4.1l2	-0.380463587	9.68981099	-4.068423827	5.68E-04	3.49E-03	-0.902534564
Zfp36l1	-0.380784353	11.69348317	-5.250904139	3.51E-05	3.80E-04	1.875228257

H2-Ob	-0.38195752	7.88379445	-2.147608953	4.38E-02	1.01E-01	-5.059174096
Mylip	-0.382248027	8.612815351	-3.871653726	9.06E-04	5.00E-03	-1.362605492
Herc6	-0.383075933	8.480791638	-3.700265769	1.36E-03	6.83E-03	-1.760501944
Rnf11	-0.3847152	7.894148149	-3.373213402	2.93E-03	1.24E-02	-2.508978618
Mex3b	-0.38570312	7.265464399	-2.536602216	1.94E-02	5.37E-02	-4.310286162
Nampt	-0.386091998	8.165847889	-4.480720342	2.14E-04	1.63E-03	0.067524342
Ddhd1	-0.387877136	10.16701978	-4.454236538	2.28E-04	1.70E-03	0.005106941
B630005N14Rik	-0.38895922	9.858335367	-4.455890597	2.27E-04	1.70E-03	0.009005169
Polg2	-0.389144836	7.408760038	-2.70599184	1.34E-02	4.06E-02	-3.963510804
Dusp6	-0.390216736	7.301248296	-2.192583867	4.00E-02	9.44E-02	-4.976388847
Prr14	-0.391111984	9.106605547	-4.618924007	1.54E-04	1.25E-03	0.393216742
Gfpt1	-0.391698433	9.684916274	-4.510149661	1.99E-04	1.56E-03	0.136885255
Nbeal1	-0.39214882	7.41568612	-4.102065675	5.25E-04	3.27E-03	-0.823615712
Mir5107	-0.39486893	10.20298079	-3.520537763	2.07E-03	9.40E-03	-2.173884205
Krtcap3	-0.395427104	7.046032349	-3.048956058	6.18E-03	2.20E-02	-3.230921316
Suco	-0.395755503	8.796939137	-3.794668319	1.09E-03	5.79E-03	-1.541719592
Ace2	-0.396018079	5.596206375	-2.811999489	1.06E-02	3.38E-02	-3.741099292
Slc25a35	-0.396997904	7.576866351	-3.158897812	4.81E-03	1.81E-02	-2.988877895
Itpr2	-0.397243211	7.687540401	-3.701370939	1.36E-03	6.83E-03	-1.757946645
Jup	-0.397578847	7.878903307	-2.685221607	1.40E-02	4.21E-02	-4.006621065
Nod1	-0.397633136	7.350378131	-2.972121744	7.36E-03	2.53E-02	-3.398170752
Tnfsf9	-0.398082043	7.050427487	-2.703713173	1.34E-02	4.07E-02	-3.968248053
Pias3	-0.399773086	8.031485068	-3.580702569	1.80E-03	8.45E-03	-2.03601212
Cbx7	-0.400167142	7.649549545	-3.088796191	5.64E-03	2.05E-02	-3.143567824
Tap1	-0.400359516	10.17986499	-6.414203194	2.54E-06	4.75E-05	4.521634279
Cd200	-0.400777154	7.847900744	-2.895693893	8.75E-03	2.92E-02	-3.562845161
Usp32	-0.40190692	8.434299231	-5.980945275	6.64E-06	1.03E-04	3.552334786
St8sia6	-0.40270823	7.887947463	-2.245438192	3.58E-02	8.69E-02	-4.877748615
Mpp5	-0.402828506	7.734066744	-3.918085501	8.12E-04	4.56E-03	-1.25431197
Slc8b1	-0.403261693	8.684286706	-4.287391891	3.38E-04	2.35E-03	-0.387937393
Clec2i	-0.403382535	8.423080457	-2.78405434	1.12E-02	3.52E-02	-3.800106816
Ppfibp1	-0.405718542	8.098501127	-4.5661471	1.75E-04	1.39E-03	0.268857371
Kcnk6	-0.40643	8.720180906	-3.776652452	1.13E-03	5.98E-03	-1.583548857
Nck2	-0.408863946	8.409463978	-2.708888894	1.33E-02	4.04E-02	-3.957485233
LOC102636131	-0.409101217	5.690353138	-3.010173037	6.75E-03	2.36E-02	-3.315547899
Sfi1	-0.409945	7.4496034	-3.910415869	8.27E-04	4.63E-03	-1.272212743
Birc3	-0.410816325	10.86469779	-5.252229149	3.50E-05	3.80E-04	1.878313047
Rnf19a	-0.411029242	8.763980654	-2.378840751	2.71E-02	6.96E-02	-4.622602775
Nuak2	-0.411290324	8.188889694	-3.365805766	2.98E-03	1.25E-02	-2.525724571
Gpcpd1	-0.411510319	9.404185707	-4.24730145	3.72E-04	2.53E-03	-0.482293107
Spg11	-0.411704445	7.919803895	-4.094241993	5.35E-04	3.30E-03	-0.841974522
Zfp189	-0.413425908	6.913289242	-2.45108018	2.33E-02	6.18E-02	-4.480948269
A030001D20Rik	-0.414707449	6.185188271	-2.099858094	4.82E-02	1.09E-01	-5.145876399
Lima1	-0.417149256	7.392467967	-2.921226589	8.26E-03	2.79E-02	-3.508026353
Flnb	-0.417173584	8.919780202	-5.423028107	2.36E-05	2.78E-04	2.27486984
Fut8	-0.41740465	8.170669602	-5.78619005	1.03E-05	1.47E-04	3.109845312
Rel	-0.417851315	10.67593003	-3.759746885	1.18E-03	6.16E-03	-1.622768429
Fbxo11	-0.418542195	9.53830609	-6.033197597	5.91E-06	9.29E-05	3.67036737
Gm11545	-0.418778369	8.392483298	-2.097347109	4.85E-02	1.09E-01	-5.150400992
Nxf1	-0.419322254	10.45560388	-6.270021737	3.49E-06	6.12E-05	4.201487776
Cyp27a1	-0.419361321	9.232841609	-4.869853645	8.54E-05	7.56E-04	0.983692076
Ggt5	-0.419907894	4.793938291	-2.200460456	3.93E-02	9.35E-02	-4.9617806
Zfp263	-0.42260322	7.414723533	-3.613031077	1.67E-03	8.01E-03	-1.961708412
Car13	-0.422745178	5.76917305	-2.403807605	2.57E-02	6.66E-02	-4.573912483
Lpin1	-0.422817854	6.722122094	-2.365859486	2.79E-02	7.11E-02	-4.647805025
Pi4k2a	-0.4233725	8.386356246	-4.882954933	8.28E-05	7.45E-04	1.01446675
Adam19	-0.42456021	8.950181306	-5.332622909	2.91E-05	3.25E-04	2.065244036
Cpeb4	-0.425178404	8.502568545	-5.196335574	3.99E-05	4.22E-04	1.748081019
Tas1r3	-0.426546603	5.861095658	-2.734139192	1.26E-02	3.87E-02	-3.904839297
Zufsp	-0.427100449	8.950817547	-5.936549489	7.34E-06	1.11E-04	3.451816957
Dirc2	-0.427184076	7.017556715	-3.071413397	5.87E-03	2.11E-02	-3.181732687
Atp6v0a1	-0.427921402	8.312829673	-3.713209378	1.32E-03	6.67E-03	-1.730565322
Map3k14	-0.429847413	8.625553068	-4.277644811	3.46E-04	2.38E-03	-0.410882118
Scpep1	-0.430776999	10.12671836	-4.965695325	6.82E-05	6.45E-04	1.208653742
Mir1931	-0.431096128	6.203462568	-2.156823479	4.30E-02	9.97E-02	-5.042300655
Nfkbia	-0.432601985	11.18519394	-6.642313951	1.55E-06	3.21E-05	5.022956071
H2-K2	-0.432641349	7.851857606	-3.032695114	6.42E-03	2.27E-02	-3.266453567
Camk2d	-0.432864881	7.126427314	-3.738206766	1.24E-03	6.40E-03	-1.672693757

4632428N05Rik	-0.433019728	8.291762172	-5.452661056	2.20E-05	2.70E-04	2.343438804
Cst7	-0.433398222	6.569860835	-2.197740143	3.95E-02	9.37E-02	-4.966829466
Pcgf5	-0.43375959	8.410066276	-3.265217097	3.76E-03	1.50E-02	-2.752039253
Rassf2	-0.433955483	8.984512911	-3.765361873	1.17E-03	6.12E-03	-1.609745576
Galnt7	-0.434090733	9.069237934	-5.354174342	2.77E-05	3.14E-04	2.115273854
Bcl3	-0.434298614	9.160843434	-3.518849357	2.08E-03	9.43E-03	-2.177745272
Acp2	-0.435984825	8.104161769	-2.90213626	8.63E-03	2.89E-02	-3.549032209
Dok1	-0.436106739	7.398253906	-3.094288729	5.57E-03	2.03E-02	-3.131492378
Trim35	-0.436318823	8.131212981	-6.316330678	3.15E-06	5.58E-05	4.304584753
Stxbp3a	-0.439243658	9.524176453	-4.066227048	5.71E-04	3.49E-03	-0.907685648
Nr4a1	-0.442972821	9.232516331	-2.846595792	9.77E-03	3.16E-02	-3.667688413
Ankrd12	-0.442994551	8.381190505	-3.755846246	1.19E-03	6.19E-03	-1.631813136
Ell2	-0.443302406	9.00578349	-5.361222373	2.72E-05	3.10E-04	2.131627549
LOC102632708	-0.444470345	5.887268374	-2.216063563	3.81E-02	9.09E-02	-4.932747158
Tnfrsf11a	-0.444676939	7.473995635	-3.101977541	5.48E-03	2.00E-02	-3.11457538
Tmem64	-0.445529147	7.521046326	-2.996893879	6.96E-03	2.42E-02	-3.34442821
Tnip1	-0.446135725	8.514109516	-5.384040338	2.58E-05	2.99E-04	2.184546112
Lilrb4	-0.44626106	8.476135627	-2.455536527	2.30E-02	6.14E-02	-4.472133332
Pnpla7	-0.446737388	6.349281468	-4.367060844	2.80E-04	2.01E-03	-0.200312122
Lcp2	-0.448493127	9.742275422	-2.829359852	1.02E-02	3.27E-02	-3.704310871
Parp4	-0.448670034	8.761551488	-5.101720063	4.97E-05	4.98E-04	1.527163614
Ppef2	-0.448929906	6.291472617	-2.243913399	3.59E-02	8.71E-02	-4.880614328
H2-Q4	-0.449162929	8.183913814	-4.555656379	1.79E-04	1.42E-03	0.244134654
Ppm1h	-0.449166237	8.583667396	-4.007489542	6.57E-04	3.88E-03	-1.045304829
Dpp7	-0.449341708	7.18402913	-2.17418093	4.15E-02	9.71E-02	-5.010392814
Gpr157	-0.44990589	8.377502606	-4.86623558	8.62E-05	7.59E-04	0.975192116
Htra2	-0.450220394	10.20388944	-5.150013465	4.44E-05	4.58E-04	1.639993664
Ric1	-0.450244899	8.669532826	-4.159646877	4.58E-04	2.95E-03	-0.688401749
Stx11	-0.450334543	6.705081303	-4.21209339	4.04E-04	2.68E-03	-0.565115446
Mir674	-0.450498072	6.774024287	-2.355493442	2.85E-02	7.24E-02	-4.667873481
Abi3	-0.451081306	7.532042125	-3.472451457	2.32E-03	1.03E-02	-2.28366838
Slc22a18	-0.451271023	6.684383083	-2.458990026	2.29E-02	6.10E-02	-4.465296096
Avpi1	-0.451503392	8.515324833	-3.489956926	2.23E-03	9.95E-03	-2.243746027
Pde1b	-0.45185766	7.49661626	-2.588331921	1.73E-02	4.93E-02	-4.205576247
LOC73899	-0.451859372	8.472098309	-3.528990397	2.03E-03	9.26E-03	-2.154547868
Plekhb2	-0.452010932	8.491843308	-4.509119275	2.00E-04	1.56E-03	0.134456782
Plk3	-0.453262553	6.219663783	-2.434903339	2.41E-02	6.33E-02	-4.512873382
Hsd17b11	-0.453558025	9.553483754	-4.363750146	2.82E-04	2.03E-03	-0.20811151
Havcr2	-0.453899756	7.995881211	-3.34431001	3.13E-03	1.30E-02	-2.574258735
Cdc14a	-0.454310477	8.86376701	-6.056181927	5.61E-06	8.89E-05	3.722191678
Suox	-0.454518824	7.699592444	-3.761589584	1.18E-03	6.15E-03	-1.618495039
Pirb	-0.457181485	9.8244488	-4.534786319	1.88E-04	1.49E-03	0.19494959
Ppp4r2	-0.458558153	9.99914904	-3.218875221	4.19E-03	1.62E-02	-2.855586735
Rabgap1l	-0.459017933	8.209510415	-4.47745868	2.16E-04	1.63E-03	0.059837093
Gm4651	-0.459788247	6.078918018	-2.218405174	3.79E-02	9.06E-02	-4.928379143
Trio	-0.463250385	8.673700567	-5.741457728	1.14E-05	1.60E-04	3.007664038
Tbc1d10a	-0.463402135	6.491908451	-2.558477685	1.85E-02	5.18E-02	-4.266139097
Srgn	-0.463406793	11.24575118	-6.095917929	5.14E-06	8.20E-05	3.811648615
Bcl2a1a	-0.463827282	10.52265101	-3.351332193	3.08E-03	1.29E-02	-2.558413587
Ltc4s	-0.464880904	6.708749541	-2.580956769	1.76E-02	4.99E-02	-4.220570924
Rnf115	-0.465095515	10.36184594	-5.118930803	4.77E-05	4.83E-04	1.567390049
Cdc42ep2	-0.465128043	6.821108363	-2.720117537	1.30E-02	3.96E-02	-3.934102161
Nfe2l1	-0.465313889	9.02767145	-5.443730261	2.25E-05	2.73E-04	2.322781042
Ssh1	-0.465566807	7.864040393	-4.530562676	1.90E-04	1.50E-03	0.184995338
Slc12a2	-0.46622038	6.947475412	-3.211216325	4.26E-03	1.65E-02	-2.872653889
Arhgap22	-0.467293787	6.822421678	-3.831885079	9.96E-04	5.39E-03	-1.455203438
Gpr132	-0.46794573	9.477460002	-4.376329369	2.74E-04	1.98E-03	-0.178476281
Cdk14	-0.468830625	8.095736195	-3.294917905	3.51E-03	1.42E-02	-2.685430862
Mical2	-0.469218832	5.712521307	-2.603163612	1.67E-02	4.80E-02	-4.175355988
Ptpn21	-0.470787398	7.046424588	-3.532204593	2.02E-03	9.21E-03	-2.147192103
4930562F07Rik	-0.472036245	8.659146986	-3.295703098	3.50E-03	1.42E-02	-2.683667439
Smurf1	-0.474109211	7.884457162	-4.704834432	1.26E-04	1.06E-03	0.595559206
Sort1	-0.474614537	8.232147634	-5.127130412	4.68E-05	4.77E-04	1.586548637
Lpp	-0.476329195	7.518762087	-3.673690707	1.45E-03	7.16E-03	-1.821901516
Smpdl3a	-0.479412712	7.934927589	-4.665785224	1.38E-04	1.14E-03	0.503604809
Mamld1	-0.479421753	5.965600832	-3.654102398	1.51E-03	7.41E-03	-1.867101448
Nfat5	-0.480847329	8.502267883	-4.500447548	2.04E-04	1.58E-03	0.114018694
Slc33a1	-0.481322023	8.501209182	-4.309812568	3.21E-04	2.26E-03	-0.335149494

Ly96	-0.482313132	7.823256782	-4.905342802	7.86E-05	7.12E-04	1.067039249
Dusp5	-0.482703585	7.654534895	-3.562658368	1.88E-03	8.77E-03	-2.077419099
Ktn1	-0.483407915	8.698810043	-5.742710927	1.14E-05	1.60E-04	3.010529393
Icosl	-0.484791679	10.35968984	-7.182283279	4.92E-07	1.22E-05	6.183115889
Sult1a1	-0.48504558	6.841788588	-3.40532777	2.71E-03	1.16E-02	-2.436260399
Chd2	-0.485876719	8.829140919	-5.521145959	1.88E-05	2.39E-04	2.501627937
Zfc3h1	-0.48799827	9.842032054	-5.184268982	4.10E-05	4.33E-04	1.719938357
Rbm47	-0.488660916	7.592938741	-4.408943904	2.54E-04	1.85E-03	-0.101629781
Polr3c	-0.489010622	9.077878641	-6.242060068	3.71E-06	6.42E-05	4.139114685
Ephx1	-0.490678428	6.46295913	-3.19056884	4.47E-03	1.71E-02	-2.918598066
Gngt2	-0.491080563	7.195538512	-3.393463719	2.79E-03	1.19E-02	-2.463147135
Rras2	-0.492303297	8.744742223	-5.517482476	1.90E-05	2.40E-04	2.493176031
Klf5	-0.497596638	6.150367579	-2.443286474	2.36E-02	6.25E-02	-4.496343672
Tmem27	-0.498006526	4.681300129	-2.546445316	1.89E-02	5.27E-02	-4.290446035
A930002C04Rik	-0.498241075	5.246281794	-2.697187464	1.36E-02	4.12E-02	-3.981804257
B230398E01Rik	-0.498467798	9.002483335	-3.535681878	2.00E-03	9.15E-03	-2.139232441
Txndc17	-0.498636182	10.44803205	-5.877732829	8.37E-06	1.25E-04	3.318325716
Zrsr1	-0.502249373	6.209299522	-3.800295004	1.07E-03	5.72E-03	-1.528648546
Myo1h	-0.502354847	6.324608214	-2.875463178	9.16E-03	3.03E-02	-3.606137654
Pnpla8	-0.502383559	7.78327041	-3.440371257	2.50E-03	1.08E-02	-2.35669474
Zfp318	-0.502850733	8.914553586	-5.856269596	8.79E-06	1.29E-04	3.269522264
Cd300ld	-0.504587676	5.882217167	-2.769178331	1.16E-02	3.62E-02	-3.831410168
Glul	-0.504762705	8.690119744	-5.987889774	6.54E-06	1.02E-04	3.568038829
Itgb8	-0.505428192	6.234621951	-2.24267565	3.60E-02	8.72E-02	-4.8829397
Slc22a21	-0.506870978	6.600686177	-3.115835881	5.31E-03	1.96E-02	-3.084046159
Cblb	-0.507162892	10.33819136	-3.940556945	7.70E-04	4.38E-03	-1.201836274
Ffar4	-0.508468577	8.095909744	-4.499982866	2.04E-04	1.58E-03	0.112923501
Gm6377	-0.509379131	8.684248536	-3.756601717	1.19E-03	6.19E-03	-1.6300615
2010016I18Rik	-0.509673177	6.393384761	-2.942968551	7.87E-03	2.68E-02	-3.461190735
Dgka	-0.510784813	7.738730648	-3.065646047	5.95E-03	2.13E-02	-3.194377752
Khnyn	-0.512151933	7.609393393	-5.655416228	1.39E-05	1.87E-04	2.810572468
Arhgap31	-0.517258182	9.032510211	-4.476836134	2.16E-04	1.63E-03	0.058369848
Snora44	-0.52047166	7.759145923	-2.804750855	1.07E-02	3.42E-02	-3.756430325
Sh3bp4	-0.522382071	7.035105826	-4.292530772	3.34E-04	2.33E-03	-0.37583939
Sox4	-0.523432272	6.115738839	-2.783985331	1.12E-02	3.52E-02	-3.800252205
Swap70	-0.524782184	9.313710833	-4.87768923	8.39E-05	7.51E-04	1.002098537
BC051537	-0.524826764	7.018358814	-4.112089448	5.13E-04	3.21E-03	-0.800089474
Ahr	-0.524835345	7.982569263	-4.119475827	5.04E-04	3.16E-03	-0.78274998
Hspbap1	-0.526138959	6.230607685	-2.877535092	9.12E-03	3.02E-02	-3.601709767
Arg2	-0.526411587	5.833276439	-2.124499962	4.59E-02	1.05E-01	-5.101289129
Slc22a23	-0.526768244	7.456132298	-2.99132435	7.05E-03	2.44E-02	-3.356526391
Irf4	-0.52678632	7.426673453	-3.178786625	4.59E-03	1.74E-02	-2.944771167
Ppt2	-0.528057618	7.402863745	-2.549718878	1.88E-02	5.25E-02	-4.283838885
N4bp2l1	-0.528267761	9.087434618	-5.270605423	3.35E-05	3.66E-04	1.921082597
Ntng2	-0.528845346	8.471673925	-3.187924342	4.50E-03	1.71E-02	-2.924475401
4930455G09Rik	-0.52922346	6.326760136	-2.790389719	1.11E-02	3.49E-02	-3.786752446
Efna2	-0.532242809	6.619315659	-3.145974956	4.95E-03	1.85E-02	-3.017485577
Rgmb	-0.533759313	7.2767953	-4.649605521	1.43E-04	1.18E-03	0.465495444
Slc2a6	-0.535374292	7.823917221	-3.927665049	7.94E-04	4.50E-03	-1.231946654
1700029I15Rik	-0.535932713	6.875118788	-2.46854053	2.24E-02	6.02E-02	-4.446360858
Tbc1d4	-0.536146018	7.775308202	-3.789583162	1.10E-03	5.82E-03	-1.553529799
Pygl	-0.536831057	7.853292803	-3.037294714	6.35E-03	2.25E-02	-3.256410112
Crebrf	-0.539434433	8.18442127	-5.403571146	2.47E-05	2.89E-04	2.229808702
Ctsd	-0.540594174	8.140930245	-4.437885373	2.37E-04	1.76E-03	-0.033428061
Plat	-0.541010645	5.719035877	-2.206745191	3.88E-02	9.25E-02	-4.950101479
Gcnt4	-0.543448063	6.538850641	-3.310144234	3.39E-03	1.38E-02	-2.651212104
Fyb	-0.545158447	10.35410345	-6.281432777	3.40E-06	6.00E-05	4.226915567
Daam1	-0.546295618	7.58398934	-6.574290516	1.79E-06	3.54E-05	4.874137589
Gpr52	-0.546468132	6.361261711	-3.303197266	3.44E-03	1.40E-02	-2.66683024
Gbp10	-0.550119387	7.141067292	-3.077649175	5.79E-03	2.09E-02	-3.168050757
Icam1	-0.550405977	9.822749306	-4.711137957	1.24E-04	1.05E-03	0.610399818
Socs1	-0.550866363	6.977441914	-3.658105344	1.50E-03	7.39E-03	-1.857868708
Rab9	-0.553237532	9.038661827	-4.14518776	4.74E-04	3.02E-03	-0.722370143
Pacsin1	-0.554549746	5.628642607	-2.379797238	2.71E-02	6.95E-02	-4.620742724
Sesn2	-0.556146821	7.213054822	-4.895215552	8.05E-05	7.28E-04	1.043260427
Anxa4	-0.556953705	9.016721537	-6.445524513	2.37E-06	4.52E-05	4.590850098
Sp140	-0.557895362	7.380392817	-2.71635569	1.31E-02	3.98E-02	-3.941941076
Sqstm1	-0.559894417	9.503013141	-6.741136555	1.25E-06	2.65E-05	5.238111892

Apobec1	-0.560133516	7.821431361	-5.046158093	5.65E-05	5.55E-04	1.397182274
Caprin2	-0.560653459	6.906817971	-3.713845595	1.32E-03	6.67E-03	-1.729093327
H2-Oa	-0.562967675	9.86496398	-6.70565652	1.35E-06	2.83E-05	5.16100818
Rasa2	-0.564584135	8.180808887	-4.269867371	3.53E-04	2.42E-03	-0.429188389
Arhgef3	-0.567894435	7.881024686	-3.673891579	1.45E-03	7.16E-03	-1.82143775
Dtx1	-0.568743321	6.126468786	-3.075399936	5.82E-03	2.10E-02	-3.172986989
Ppp3cc	-0.570120701	6.679372703	-3.332467167	3.22E-03	1.33E-02	-2.600959434
Fcrla	-0.578044924	6.499836691	-3.654732258	1.51E-03	7.41E-03	-1.865648825
Cd80	-0.578342413	8.832254191	-4.370087686	2.78E-04	2.01E-03	-0.193181293
Tmem140	-0.57943983	6.407631326	-3.478724286	2.29E-03	1.02E-02	-2.269368654
Sh3bp5	-0.579632045	6.566644392	-3.616353585	1.66E-03	7.96E-03	-1.95406362
2310001H17Rik	-0.582011226	8.15331373	-4.280306237	3.44E-04	2.37E-03	-0.404617352
Nxpe4	-0.583178472	5.70430767	-2.594644853	1.71E-02	4.88E-02	-4.192724001
Bcl2l1	-0.583988509	6.717639828	-3.752349057	1.20E-03	6.23E-03	-1.639920886
4930412F12Rik	-0.584403148	6.185930048	-4.422448533	2.46E-04	1.80E-03	-0.069806523
Ttyh2	-0.587216335	6.335678113	-4.214545045	4.02E-04	2.67E-03	-0.559349668
Plxnc1	-0.587671253	11.14016603	-6.226076728	3.85E-06	6.56E-05	4.103420124
Pmepa1	-0.588944313	6.190990757	-4.487775875	2.10E-04	1.60E-03	0.08415324
Mocos	-0.591866753	5.191339313	-3.914836568	8.18E-04	4.59E-03	-1.261895517
Lphn3	-0.596220687	4.470689147	-2.470747843	2.23E-02	6.00E-02	-4.441978891
Clip2	-0.597738153	7.562019024	-5.550441205	1.76E-05	2.27E-04	2.569171935
Spred1	-0.598668663	8.33953904	-4.058398198	5.82E-04	3.53E-03	-0.926040687
H2-Q6	-0.598920035	10.73244139	-6.340781641	2.99E-06	5.36E-05	4.358916841
Gm9994	-0.599159183	6.48563562	-2.98420444	7.16E-03	2.48E-02	-3.371979511
Clec2d	-0.600152602	10.52621348	-7.050585637	6.49E-07	1.52E-05	5.903680877
Chd7	-0.60127755	7.977361565	-5.86670312	8.58E-06	1.27E-04	3.29325215
Gfpt2	-0.603022427	6.131373732	-2.17158628	4.17E-02	9.75E-02	-5.01517269
Iscu	-0.604184812	9.869137104	-5.581293343	1.64E-05	2.14E-04	2.640223438
Trim7	-0.605374296	7.669770897	-6.592272225	1.73E-06	3.42E-05	4.913533662
AW046200	-0.606142768	6.408441906	-3.358276599	3.03E-03	1.27E-02	-2.542734482
Rrad	-0.606835566	7.070788238	-4.062836508	5.76E-04	3.50E-03	-0.915635346
Peg13	-0.608424515	7.102329306	-4.23377874	3.84E-04	2.59E-03	-0.514108554
Hfe	-0.609183812	6.889314049	-2.908252944	8.51E-03	2.86E-02	-3.535905738
Tank	-0.61039885	9.255562733	-5.143656933	4.50E-05	4.64E-04	1.625150772
Cxcl1	-0.612149413	5.322661922	-2.341582932	2.93E-02	7.41E-02	-4.69472442
D130040H23Rik	-0.612157901	6.155933973	-2.579845967	1.76E-02	4.99E-02	-4.22282745
Cmah	-0.614786819	5.646292456	-2.219452653	3.78E-02	9.04E-02	-4.926424273
Rftn1	-0.615448393	8.501303902	-5.52262709	1.88E-05	2.39E-04	2.505044679
Prkca	-0.616653541	4.744676194	-2.535713604	1.94E-02	5.37E-02	-4.312075313
Ccr9	-0.617079018	6.708486551	-2.447455195	2.34E-02	6.21E-02	-4.488112269
Pla2g16	-0.617440817	8.650881302	-5.005405099	6.22E-05	5.96E-04	1.301737247
Il2ra	-0.618170747	6.1703802	-2.704332494	1.34E-02	4.07E-02	-3.966960698
Nr4a2	-0.621749245	7.629327767	-2.701395335	1.35E-02	4.09E-02	-3.973064801
Spsb1	-0.624148136	7.518544567	-2.877069439	9.13E-03	3.02E-02	-3.602705033
E130311K13Rik	-0.624467361	6.670934166	-3.997614258	6.72E-04	3.95E-03	-1.068419953
Trbj1-6	-0.625567346	8.669095088	-2.936994971	7.97E-03	2.70E-02	-3.474072848
Wdr91	-0.626362986	8.174263296	-8.15740076	6.86E-08	2.37E-06	8.179261295
BC021614	-0.626386124	6.386156855	-2.5573337	1.85E-02	5.19E-02	-4.268452637
LOC102636093	-0.626612054	6.91202084	-2.726006572	1.28E-02	3.92E-02	-3.921820433
Scn4b	-0.627338567	5.986040055	-3.481712868	2.27E-03	1.01E-02	-2.262553482
Abcg1	-0.627716459	9.144544143	-5.577209157	1.66E-05	2.15E-04	2.63082258
Serpinc1	-0.627784749	5.723618465	-3.72379421	1.29E-03	6.57E-03	-1.706069282
Tmem19	-0.628596993	8.52873234	-5.800507216	9.97E-06	1.44E-04	3.142507608
Pla2g4f	-0.633631169	6.05381645	-3.506025172	2.15E-03	9.67E-03	-2.207056965
Gm10277	-0.634411238	6.435102868	-3.257189117	3.83E-03	1.52E-02	-2.770010885
Chka	-0.637530227	8.123180258	-5.48498442	2.05E-05	2.55E-04	2.418150179
Cenpv	-0.63868707	6.677468049	-3.90965444	8.28E-04	4.63E-03	-1.273989633
Notch4	-0.639762617	6.469330801	-4.350829837	2.91E-04	2.07E-03	-0.238547573
Zfyve1	-0.640678202	7.44914547	-7.467792746	2.73E-07	7.29E-06	6.780930085
Dusp2	-0.642150711	8.301216617	-4.832347194	9.33E-05	8.18E-04	0.895553626
Cd2	-0.643148722	6.563491584	-2.273164752	3.38E-02	8.32E-02	-4.82543489
LOC102632070	-0.643909482	5.871555446	-3.048293444	6.19E-03	2.20E-02	-3.232370606
Il1r1	-0.644181317	5.033624163	-2.319689819	3.07E-02	7.72E-02	-4.73679733
LOC102632231	-0.644792231	7.362839127	-4.453389691	2.28E-04	1.70E-03	0.003111128
Stra6l	-0.644895991	7.104218902	-2.949475892	7.75E-03	2.65E-02	-3.447145525
Rnf180	-0.647487006	6.716873853	-4.302106652	3.27E-04	2.29E-03	-0.353293945
Sbf2	-0.647505623	8.228797713	-7.386263688	3.22E-07	8.38E-06	6.611340802
Gpr33	-0.648818835	7.530817407	-4.504385292	2.02E-04	1.57E-03	0.123299433

Fnip2	-0.649954586	7.759473744	-5.431796356	2.31E-05	2.75E-04	2.295166563
Cx3cl1	-0.650183903	6.049129719	-3.697685798	1.37E-03	6.86E-03	-1.766466598
Mmp14	-0.650730752	6.163449278	-2.663903984	1.47E-02	4.36E-02	-4.050701852
Rasal2	-0.652386641	7.649890943	-3.565071561	1.87E-03	8.74E-03	-2.071884208
Eml5	-0.652885528	7.86389125	-4.232225948	3.85E-04	2.59E-03	-0.517761481
Adcy4	-0.654406691	5.273531497	-2.959669713	7.57E-03	2.60E-02	-3.425118498
Galc	-0.654683836	6.986739478	-5.363486457	2.71E-05	3.09E-04	2.136880137
Gpr137b	-0.655454417	7.307606651	-4.095817884	5.33E-04	3.30E-03	-0.838276851
Gm1965	-0.657161589	7.120679071	-2.577049441	1.77E-02	5.02E-02	-4.228506233
Amn1	-0.65754266	7.730898699	-5.28293833	3.26E-05	3.57E-04	1.949773145
Arhgef9	-0.657740037	5.903035744	-4.056501569	5.85E-04	3.54E-03	-0.930486855
Irf1	-0.658247472	8.778343769	-7.163969719	5.11E-07	1.25E-05	6.144396554
Spic	-0.658730673	6.848823071	-2.318105388	3.08E-02	7.73E-02	-4.739833272
Il4ra	-0.658989821	8.748551574	-5.860795061	8.70E-06	1.29E-04	3.279816311
Scaper	-0.660204285	8.834122499	-9.69763088	3.92E-09	2.24E-07	11.07199171
Leprel1	-0.660916378	7.262982211	-3.235340341	4.03E-03	1.58E-02	-2.81885098
Asap2	-0.662051457	7.998992225	-6.347209174	2.95E-06	5.34E-05	4.373187499
Rab30	-0.666933245	9.814604645	-5.533547386	1.83E-05	2.34E-04	2.530230219
Galnt12	-0.667561379	7.791512267	-4.610550588	1.57E-04	1.27E-03	0.373488449
Mir155	-0.669530864	5.804127117	-2.149923998	4.36E-02	1.01E-01	-5.054939124
Ptafr	-0.671726118	8.032854137	-4.281393109	3.43E-04	2.37E-03	-0.402058894
Grk5	-0.674334896	8.194875821	-5.368245021	2.68E-05	3.08E-04	2.147918528
2510009E07Rik	-0.674385282	6.168242168	-3.806692131	1.06E-03	5.66E-03	-1.513783748
4930487H11Rik	-0.677673069	5.403209446	-4.258241686	3.62E-04	2.47E-03	-0.456549233
Lag3	-0.679346119	6.76458427	-3.330808504	3.23E-03	1.34E-02	-2.604696807
Izumo4	-0.681830296	7.431203413	-4.805201073	9.94E-05	8.68E-04	0.831729846
Ltb	-0.682744711	7.152595761	-4.608201723	1.58E-04	1.27E-03	0.367954209
Casp4	-0.683719233	7.747665533	-6.745647213	1.24E-06	2.64E-05	5.24790272
Mctp2	-0.68485294	5.066018869	-2.732477442	1.26E-02	3.88E-02	-3.908310989
Slc25a29	-0.686782195	6.235858642	-4.819948944	9.61E-05	8.40E-04	0.866407009
Nudt9	-0.68783357	8.026309762	-6.032675851	5.91E-06	9.29E-05	3.669190278
Pdgfa	-0.689547311	6.39406221	-4.506255866	2.01E-04	1.57E-03	0.127708122
Zmat1	-0.689827815	7.02098841	-3.495580274	2.20E-03	9.86E-03	-2.23091087
Grina	-0.69042514	8.795838638	-5.844524886	9.02E-06	1.32E-04	3.24279679
Gdap10	-0.69161246	9.220410884	-6.391253895	2.67E-06	4.97E-05	4.470843766
Ccser2	-0.69214864	9.951697815	-7.735405752	1.58E-07	4.68E-06	7.331258578
Hepacam2	-0.69270737	6.008295626	-3.502274926	2.16E-03	9.75E-03	-2.215623756
Tmcc3	-0.692930903	7.039926554	-5.017277612	6.05E-05	5.85E-04	1.329552131
Strip2	-0.693533596	8.744283296	-3.474284132	2.31E-03	1.03E-02	-2.279491242
Tagap	-0.69353785	8.707594605	-4.837595296	9.22E-05	8.10E-04	0.907889564
Pmaip1	-0.693910293	8.382504491	-5.095511306	5.04E-05	5.02E-04	1.512647661
Renbp	-0.694395091	6.636110869	-5.048941125	5.62E-05	5.54E-04	1.403697
Chst11	-0.695394732	6.981830444	-3.993619614	6.79E-04	3.97E-03	-1.077768312
Zfp945	-0.695461313	7.496625102	-5.297422654	3.15E-05	3.48E-04	1.983454608
Clnk	-0.695972675	6.504583493	-3.763750979	1.17E-03	6.13E-03	-1.613482082
Treml4	-0.696278584	8.190843141	-3.718899224	1.30E-03	6.60E-03	-1.717399197
Havcr1	-0.696446809	5.413989509	-2.547707825	1.89E-02	5.26E-02	-4.287898388
Mt1	-0.696840025	8.526046102	-4.518837308	1.95E-04	1.53E-03	0.157360734
Cxcl16	-0.696844118	10.71335507	-5.645436211	1.42E-05	1.89E-04	2.78766604
Atp11a	-0.697524216	8.515980279	-7.348725458	3.48E-07	9.00E-06	6.532955347
Rnf19b	-0.697707007	10.39380614	-7.577656727	2.18E-07	6.00E-06	7.008035199
Pianp	-0.698838629	6.269934137	-3.346583763	3.11E-03	1.30E-02	-2.569129202
Egr3	-0.699850299	6.959687874	-2.742099242	1.23E-02	3.82E-02	-3.888195762
Tnfrsf9	-0.700879416	6.853810243	-3.999922571	6.69E-04	3.93E-03	-1.063017477
Dapl1	-0.702031675	5.257824769	-2.118597909	4.64E-02	1.05E-01	-5.111998755
Sla2	-0.703330842	6.011888199	-2.239762892	3.63E-02	8.75E-02	-4.888408862
Spib	-0.715398085	6.878346384	-3.342038305	3.15E-03	1.31E-02	-2.579382636
Atp1b1	-0.716229212	6.698861839	-3.733661314	1.26E-03	6.46E-03	-1.68322238
Hpgds	-0.716308066	5.039068728	-3.606978067	1.69E-03	8.07E-03	-1.975631891
Tnfaip3	-0.716522289	9.737589431	-6.632898076	1.58E-06	3.25E-05	5.002391205
Cacnb1	-0.720942868	6.912137309	-6.215290659	3.94E-06	6.63E-05	4.079315477
Arhgap8	-0.722201301	5.097424559	-2.727327048	1.27E-02	3.91E-02	-3.919064846
Ppp1r15a	-0.723425305	10.27427317	-7.225980455	4.49E-07	1.13E-05	6.275321298
Cpq	-0.723677907	6.957047967	-5.474714015	2.09E-05	2.60E-04	2.394420943
Fam49a	-0.725940004	10.52776753	-6.961962158	7.82E-07	1.75E-05	5.714347013
Bmp1	-0.729646685	5.386705702	-3.805055358	1.06E-03	5.66E-03	-1.517587465
Olfr108	-0.732724649	5.283151633	-2.769185407	1.16E-02	3.62E-02	-3.831395295
Dscaml1	-0.734402379	5.86052372	-4.909894957	7.78E-05	7.08E-04	1.077726315

Entpd1	-0.734791587	7.963696257	-4.87189193	8.50E-05	7.54E-04	0.988480415
Tubb2b	-0.735648185	7.767366025	-4.238319019	3.80E-04	2.57E-03	-0.503427127
Ccr5	-0.736197945	6.231595598	-2.877730007	9.11E-03	3.02E-02	-3.601293147
AW112010	-0.736449198	10.15438181	-4.436519256	2.37E-04	1.76E-03	-0.036647528
Fgr	-0.736646414	8.274756724	-2.247851767	3.57E-02	8.66E-02	-4.87321011
H2-Q5	-0.736801242	9.356464689	-7.78832294	1.42E-07	4.33E-06	7.438925923
Adam11	-0.741466419	8.34307666	-4.948017632	7.11E-05	6.63E-04	1.167190994
Trp53inp1	-0.741603861	7.784251399	-5.674494642	1.33E-05	1.81E-04	2.854335708
Tec	-0.743205561	8.253682196	-5.901632579	7.93E-06	1.20E-04	3.372612894
Tmem39a	-0.743662756	9.613834003	-6.263966181	3.54E-06	6.17E-05	4.18798765
Cdcp1	-0.744511404	5.864575959	-3.83726443	9.83E-04	5.34E-03	-1.442686875
Rogdi	-0.745249963	11.30467892	-8.910264802	1.63E-08	7.78E-07	9.632310589
Ift43	-0.749049737	6.928648174	-5.484542615	2.05E-05	2.55E-04	2.417129597
Nabp1	-0.752238757	10.5218103	-6.602648105	1.69E-06	3.40E-05	4.936247702
Snx24	-0.753055353	4.709116672	-3.168131177	4.70E-03	1.78E-02	-2.9684131
Enpp5	-0.753449033	6.348916038	-2.874560903	9.18E-03	3.03E-02	-3.608065482
5430437J10Rik	-0.756696146	7.554636855	-3.600385733	1.72E-03	8.16E-03	-1.990790103
Emp2	-0.763945818	5.419228437	-2.738356178	1.24E-02	3.85E-02	-3.896024863
Foxh1	-0.763983467	5.517276132	-6.21970085	3.90E-06	6.62E-05	4.089172983
Cttnbp2nl	-0.764810043	8.293471147	-4.472135205	2.18E-04	1.65E-03	0.047290515
Pvr	-0.767562172	8.29720449	-7.730030344	1.60E-07	4.68E-06	7.320300234
4921511I17Rik	-0.768237206	7.372573061	-5.229410114	3.69E-05	3.97E-04	1.825170416
Myo1e	-0.768943349	5.99485464	-2.96622379	7.46E-03	2.57E-02	-3.410940267
Cd14	-0.77033498	6.280781495	-3.114373932	5.32E-03	1.96E-02	-3.087269048
Mir3097	-0.771643165	7.739232883	-4.366891625	2.80E-04	2.01E-03	-0.200710774
Pstpip2	-0.772043956	6.13053436	-3.004865303	6.84E-03	2.39E-02	-3.327097405
Gnb4	-0.773110558	8.434280596	-4.172602559	4.44E-04	2.88E-03	-0.657957379
Spint2	-0.779994834	8.820075567	-7.894803814	1.15E-07	3.65E-06	7.654420145
Ube2e2	-0.781126207	6.800375566	-5.09122047	5.09E-05	5.06E-04	1.502614455
Erc1	-0.781798599	6.552512306	-5.259488122	3.44E-05	3.75E-04	1.895210671
Ccng2	-0.783817337	9.114131972	-6.963336596	7.80E-07	1.75E-05	5.717291238
Il1b	-0.784615602	8.334010182	-3.636275806	1.58E-03	7.68E-03	-1.908192584
Rasgrp1	-0.789483468	6.51362526	-3.139879298	5.02E-03	1.88E-02	-3.030965608
Sdc4	-0.791268598	7.233748444	-4.413128581	2.51E-04	1.84E-03	-0.091768909
Olfr98	-0.792692532	7.521583878	-4.148725497	4.70E-04	3.01E-03	-0.714059924
LOC102635290	-0.793428859	6.459515137	-3.381135073	2.87E-03	1.22E-02	-2.491059089
Cacna1e	-0.793492758	6.591674235	-3.491379013	2.22E-03	9.93E-03	-2.240500636
Cdh2	-0.795170725	5.960212555	-3.632224488	1.59E-03	7.74E-03	-1.917525142
H2-Q7	-0.798472847	9.882887511	-6.542564642	1.92E-06	3.75E-05	4.804530907
Csf1	-0.798546768	6.307309958	-3.202452426	4.35E-03	1.67E-02	-2.892167044
Smagp	-0.801817227	6.77142385	-7.171656196	5.03E-07	1.24E-05	6.160653095
Nfkbid	-0.802706953	8.235729146	-4.923110363	7.54E-05	6.95E-04	1.108746976
Adam8	-0.803366667	9.635298195	-7.055787688	6.42E-07	1.51E-05	5.914762239
Hes1	-0.803638678	5.937299761	-4.424231169	2.44E-04	1.80E-03	-0.065605664
Itgb5	-0.804161594	6.119361564	-5.015621856	6.07E-05	5.85E-04	1.325673467
S1pr1	-0.805889001	6.288286262	-2.71227799	1.32E-02	4.01E-02	-3.950432408
Csf2rb2	-0.809197338	9.042320093	-7.039973259	6.63E-07	1.53E-05	5.881063382
Cd40	-0.815471618	8.786299272	-7.97526362	9.81E-08	3.19E-06	7.816228978
Fam129c	-0.819074543	6.144756143	-5.335825888	2.88E-05	3.24E-04	2.07268171
Fmn1	-0.819125403	7.043431058	-5.653399938	1.39E-05	1.87E-04	2.805945373
Cxcl10	-0.821606396	6.84682039	-3.957145966	7.40E-04	4.23E-03	-1.163071578
Tmem120b	-0.823623481	7.158514121	-5.814021416	9.67E-06	1.40E-04	3.17331911
Gabbr1	-0.823792124	6.837708429	-4.907907401	7.81E-05	7.10E-04	1.073060252
Nfkbiz	-0.826717357	8.903886475	-7.594546808	2.11E-07	5.88E-06	7.042804235
Oprd1	-0.82731036	6.834656496	-4.458059618	2.26E-04	1.69E-03	0.014117061
Nlrp1b	-0.828155134	6.982256958	-4.261045942	3.60E-04	2.46E-03	-0.449949838
Cd69	-0.828737824	6.796739945	-5.122560579	4.73E-05	4.81E-04	1.575871611
Gm19723	-0.831914519	6.886787169	-5.220230952	3.77E-05	4.03E-04	1.803783222
Stxbp1	-0.835223352	6.556577383	-5.368283075	2.68E-05	3.08E-04	2.148006796
Cd83	-0.838301754	9.309094156	-7.878406277	1.19E-07	3.74E-06	7.62133569
Csrnp1	-0.838708757	8.299377188	-7.162336456	5.13E-07	1.25E-05	6.140941263
Scn2b	-0.844533627	6.422007653	-6.258080861	3.58E-06	6.23E-05	4.174862916
Zdhhc14	-0.847043458	7.707144481	-6.317624941	3.14E-06	5.58E-05	4.307462501
Gm5797	-0.854182251	7.79616844	-4.684014996	1.32E-04	1.10E-03	0.546536884
Arc	-0.85428618	6.947228554	-5.590596017	1.61E-05	2.12E-04	2.66163044
Birc2	-0.857048126	9.322988779	-7.909089493	1.12E-07	3.57E-06	7.683213763
Calm4	-0.85770284	7.312268148	-4.053878631	5.88E-04	3.54E-03	-0.936635315
Dgkh	-0.858186726	6.178628513	-5.694325011	1.27E-05	1.74E-04	2.899787201

Nlrc5	-0.858877382	8.490616903	-8.725272309	2.31E-08	1.02E-06	9.282307441
Stat4	-0.859881288	7.914666835	-4.489917169	2.09E-04	1.60E-03	0.089199982
Bhlhe40	-0.862073534	8.641581431	-5.182642334	4.11E-05	4.33E-04	1.716143831
Cd81	-0.862894163	10.64802317	-5.497347568	1.99E-05	2.49E-04	2.446702722
Abcg3	-0.863023222	7.276252655	-4.99694402	6.34E-05	6.05E-04	1.281910249
Psca	-0.863209448	4.527116622	-3.237070872	4.01E-03	1.57E-02	-2.814986435
Fxyd2	-0.865695718	6.188534062	-4.425344549	2.44E-04	1.80E-03	-0.062981921
Tnnt2	-0.869648822	7.155603206	-3.552142124	1.93E-03	8.94E-03	-2.101528934
LOC102634822	-0.870745089	7.604991526	-4.236012804	3.82E-04	2.58E-03	-0.5088528
Trbj1-1	-0.872369143	6.039754236	-2.69208706	1.38E-02	4.16E-02	-3.992388764
Serpina3g	-0.875123767	9.750266444	-6.195737934	4.11E-06	6.83E-05	4.035584742
Ccl17	-0.875903705	7.23637869	-2.988500547	7.10E-03	2.46E-02	-3.362656925
Eno2	-0.87627127	6.883518439	-4.928265863	7.45E-05	6.90E-04	1.120846459
Rragd	-0.877189081	5.30797966	-6.428449409	2.47E-06	4.63E-05	4.553131283
Trbj2-7	-0.878376071	6.983072538	-2.098447321	4.84E-02	1.09E-01	-5.148418929
9330175E14Rik	-0.88257904	6.071710989	-2.901058918	8.65E-03	2.89E-02	-3.551343008
Ly75	-0.883976518	8.662952836	-5.600180031	1.57E-05	2.09E-04	2.683676648
Hap1	-0.892140218	6.347979654	-5.0422018	5.71E-05	5.58E-04	1.387920366
Sned1	-0.897204145	6.617780335	-4.306421969	3.23E-04	2.27E-03	-0.343133219
Pde8a	-0.899802655	6.828592452	-5.509932703	1.93E-05	2.44E-04	2.475754519
LOC102634900	-0.904247663	6.59448961	-5.53808329	1.81E-05	2.32E-04	2.540688336
Cdkn1a	-0.905915438	10.55864255	-7.297376441	3.87E-07	9.80E-06	6.42542337
Gal3st2	-0.908505235	7.93288929	-4.953716726	7.02E-05	6.58E-04	1.18055973
Tctex1d4	-0.913835166	5.892603968	-4.23113952	3.86E-04	2.60E-03	-0.520317245
Lef1	-0.915167755	5.742376523	-2.590135995	1.72E-02	4.92E-02	-4.20190502
Egr2	-0.915304204	6.81097633	-4.255595472	3.65E-04	2.49E-03	-0.462776481
Pilrb1	-0.915628204	4.839470505	-2.665704871	1.46E-02	4.35E-02	-4.046984518
Fndc7	-0.915672348	6.347289666	-6.769889567	1.18E-06	2.54E-05	5.300478413
Tnni3	-0.918318236	8.266889756	-7.561430993	2.25E-07	6.15E-06	6.974597266
Maml2	-0.923726842	7.171548022	-5.588118258	1.62E-05	2.13E-04	2.655929469
Ctla2b	-0.926982572	5.45870636	-3.148288039	4.92E-03	1.85E-02	-3.012368017
2610528A11Rik	-0.930668038	6.423871747	-2.62440517	1.60E-02	4.65E-02	-4.131924777
Serinc5	-0.932476691	7.377011638	-7.981936767	9.68E-08	3.17E-06	7.829609431
Tns1	-0.933972625	7.251446382	-6.595183884	1.72E-06	3.42E-05	4.919908996
Fnbp1l	-0.934531496	8.563502455	-6.45420489	2.33E-06	4.46E-05	4.610011346
Itk	-0.93933743	5.372851719	-2.669420228	1.45E-02	4.33E-02	-4.039311557
Nrp2	-0.945790458	6.163040967	-5.561331769	1.72E-05	2.22E-04	2.594262261
Cadm1	-0.949691647	8.560750181	-6.433767764	2.44E-06	4.62E-05	4.564883318
Cd96	-0.950304233	5.296380933	-3.552617099	1.92E-03	8.94E-03	-2.100440353
Scin	-0.951378332	5.666870296	-3.695285136	1.37E-03	6.88E-03	-1.772015974
Ttc39b	-0.953268857	7.850540114	-7.656364726	1.86E-07	5.27E-06	7.169728757
Bcl2l11	-0.953344327	7.351244766	-8.683102232	2.50E-08	1.08E-06	9.201885069
Poglut1	-0.956622665	9.874388144	-9.610746346	4.58E-09	2.57E-07	10.91702953
Timd4	-0.962209218	5.872775417	-3.07946778	5.77E-03	2.08E-02	-3.164058639
Trpm2	-0.967800581	6.134968766	-4.981359977	6.58E-05	6.26E-04	1.245382476
Homer2	-0.975586947	5.125106947	-4.920486839	7.59E-05	6.97E-04	1.102589357
Mtss1l	-0.977263692	6.853228425	-5.222975593	3.75E-05	4.01E-04	1.810178761
Lck	-0.980297686	6.697700307	-2.977746537	7.27E-03	2.51E-02	-3.385983295
Basp1	-0.982571887	9.214062707	-14.55592017	2.55E-12	8.01E-10	18.40989075
Cdkn2b	-0.987012826	6.895543904	-4.278618756	3.45E-04	2.38E-03	-0.40858956
Arhgef12	-0.988636947	5.942036877	-9.390610671	6.78E-09	3.60E-07	10.52011802
Ccnd2	-0.988785515	7.228677565	-4.48915103	2.10E-04	1.60E-03	0.087394293
Eno3	-0.989232249	9.223672038	-8.576155382	3.06E-08	1.19E-06	8.996861098
Cxcl2	-0.99020728	6.120702276	-2.178285765	4.11E-02	9.64E-02	-5.002823588
Gm6644	-0.990956887	5.266271756	-2.147100596	4.38E-02	1.01E-01	-5.060103659
Fam46c	-0.991445304	8.055999939	-8.07640341	8.04E-08	2.70E-06	8.018376032
Traj43	-0.993660028	5.964098492	-2.226728772	3.72E-02	8.92E-02	-4.912829562
Gpr126	-0.994586856	7.198298172	-5.171493058	4.22E-05	4.38E-04	1.690130997
LOC102633435	-0.994844091	6.533668971	-3.201102832	4.36E-03	1.68E-02	-2.895170403
LOC102639105	-0.9998685	10.94276412	-8.850532001	1.82E-08	8.52E-07	9.519793352
Phxr4	-1.000176195	7.23784469	-5.695019171	1.27E-05	1.74E-04	2.901377545
Il15ra	-1.000904104	8.848225173	-6.10608777	5.02E-06	8.05E-05	3.834515378
4932438H23Rik	-1.001229661	7.438397143	-6.351160532	2.92E-06	5.32E-05	4.381958001
Extl1	-1.001303697	6.200261255	-4.066822033	5.71E-04	3.49E-03	-0.906290536
Fas	-1.002772074	7.278762663	-5.099473053	4.99E-05	4.99E-04	1.521910409
Mfge8	-1.00532135	8.008199872	-6.536838931	1.95E-06	3.78E-05	4.791955317
Nlrp1c-ps	-1.012010806	6.660966512	-2.545912059	1.90E-02	5.27E-02	-4.29152191
Etv3	-1.012156912	10.13802526	-7.8337819	1.30E-07	4.02E-06	7.531113943

Mir29a	-1.013362644	4.240694836	-2.465498418	2.25E-02	6.04E-02	-4.452396599
Bcl2a1b	-1.014309099	11.29044787	-10.15923654	1.76E-09	1.27E-07	11.87950511
Tmem150c	-1.017285823	7.576876665	-4.063993539	5.74E-04	3.50E-03	-0.912922566
Zc3h12c	-1.017829275	9.266847298	-10.47456062	1.03E-09	8.63E-08	12.4161839
Vmn2r90	-1.018416716	4.203111535	-3.47615204	2.30E-03	1.02E-02	-2.27523322
Yes1	-1.019852457	5.503583187	-3.233441344	4.05E-03	1.58E-02	-2.823090973
Cflar	-1.025653902	9.028725244	-8.449593548	3.89E-08	1.45E-06	8.752250972
LOC102638508	-1.031245664	6.202001187	-4.579989032	1.69E-04	1.35E-03	0.301476261
Trbc2	-1.032948789	9.557385329	-3.560558844	1.89E-03	8.81E-03	-2.082233854
Traj39	-1.038551543	5.323567993	-2.292547004	3.25E-02	8.04E-02	-4.78863757
Gm3696	-1.040876019	6.567447241	-2.788136492	1.11E-02	3.50E-02	-3.791503592
Rhoc	-1.041229098	7.49019953	-3.500378056	2.17E-03	9.77E-03	-2.219955961
Asb2	-1.042520546	6.804679942	-7.684630146	1.75E-07	5.09E-06	7.227590183
A630001O12Rik	-1.047101087	4.608080994	-2.443582909	2.36E-02	6.25E-02	-4.495758596
Tspan3	-1.047491818	9.552115575	-8.597170369	2.94E-08	1.18E-06	9.037269113
Myo6	-1.049882543	5.736837342	-4.797697741	1.01E-04	8.79E-04	0.814084228
Rasgef1b	-1.054774751	7.355784036	-6.427638313	2.47E-06	4.63E-05	4.551338691
Slamf1	-1.055561218	6.195467169	-3.655652964	1.51E-03	7.41E-03	-1.863525337
Cldn1	-1.056138474	8.730410583	-6.36427758	2.84E-06	5.24E-05	4.411059395
Il15	-1.058133117	8.38113635	-5.731789196	1.16E-05	1.63E-04	2.985552423
Arl5c	-1.059015942	8.455719936	-10.26502754	1.47E-09	1.09E-07	12.06089174
Slc44a1	-1.066736178	9.094280242	-10.14270816	1.81E-09	1.27E-07	11.85104347
Gfra2	-1.070907891	6.940531456	-4.76450427	1.09E-04	9.33E-04	0.736001665
Traj23	-1.071238217	5.38865494	-3.446170557	2.47E-03	1.07E-02	-2.343506533
Frmd5	-1.071886043	6.163515456	-4.947532823	7.12E-05	6.63E-04	1.166053675
Idh1	-1.072810096	9.001287755	-9.704046211	3.88E-09	2.24E-07	11.083396
Pfkfb3	-1.077557237	9.784084317	-8.984583361	1.42E-08	6.87E-07	9.771644605
Trbc1	-1.077892409	8.658557022	-3.158806563	4.81E-03	1.81E-02	-2.989080035
Slc41a2	-1.08018519	7.295470705	-6.203803081	4.04E-06	6.74E-05	4.053628247
Snn	-1.090563941	7.548240817	-7.779218973	1.45E-07	4.37E-06	7.4204298
Gbp9	-1.092729567	9.836111383	-9.422757384	6.40E-09	3.49E-07	10.57846558
Perm1	-1.10133357	6.543290689	-12.45671202	4.63E-11	8.72E-09	15.53275402
Rbpms	-1.103586256	7.747178594	-9.017806598	1.34E-08	6.63E-07	9.833696923
Tnfrsf1b	-1.109247958	9.326362226	-8.391288767	4.35E-08	1.59E-06	8.638837627
E030011O05Rik	-1.115675773	6.394928518	-5.286285994	3.23E-05	3.55E-04	1.957559065
Trib1	-1.118700848	8.149807637	-7.657044044	1.86E-07	5.27E-06	7.171120646
Il27	-1.1198908	7.038506616	-4.634962022	1.49E-04	1.21E-03	0.431000513
Ttc39a	-1.120160962	7.940951292	-8.433591149	4.01E-08	1.48E-06	8.721169071
Vsig10	-1.1206719	6.573835531	-6.163877436	4.42E-06	7.24E-05	3.964232493
Fah	-1.123537486	5.988454985	-5.550629505	1.76E-05	2.27E-04	2.569605841
Gm10677	-1.127823368	6.472742237	-6.762347569	1.20E-06	2.56E-05	5.284129767
Esam	-1.13109667	5.533618529	-6.23992572	3.73E-06	6.42E-05	4.134349907
Procr	-1.133007201	7.392912417	-6.998232092	7.24E-07	1.65E-05	5.791958702
Arhgap28	-1.133292017	6.962414143	-6.854526986	9.83E-07	2.14E-05	5.48344201
Trbj2-6	-1.133317879	4.907188606	-2.515253508	2.03E-02	5.56E-02	-4.353179247
Mxd1	-1.134991772	9.559405354	-9.856577755	2.97E-09	1.78E-07	11.35302664
Heatr9	-1.143311894	6.760031674	-6.9796405	7.53E-07	1.71E-05	5.752197371
F11r	-1.144324322	7.638311332	-6.228466335	3.83E-06	6.56E-05	4.108758574
Adam23	-1.155833997	10.78064334	-11.56005865	1.80E-10	2.12E-08	14.17561529
Klri1	-1.156276654	6.456371583	-5.071657364	5.33E-05	5.28E-04	1.456856834
Gatsl2	-1.158669671	7.740080245	-8.013107724	9.10E-08	3.04E-06	7.892030603
Relb	-1.161912774	9.468017796	-9.723278047	3.75E-09	2.21E-07	11.1175527
Cd3d	-1.165888131	6.971837659	-3.205379914	4.32E-03	1.66E-02	-2.885650837
Ankrd33b	-1.166511572	6.361669232	-6.325387493	3.09E-06	5.52E-05	4.32471807
Cd274	-1.166941768	8.431430832	-9.195229747	9.65E-09	4.85E-07	10.16262838
Prkcb	-1.167690929	8.267696816	-6.87939974	9.32E-07	2.04E-05	5.537033773
Samsn1	-1.167868138	9.734249563	-9.886926985	2.82E-09	1.74E-07	11.40632898
Synpo2	-1.172267739	6.138728657	-7.126288085	5.53E-07	1.33E-05	6.064587672
Tmem123	-1.174094737	10.91072837	-13.40796435	1.19E-11	2.49E-09	16.8856409
Myof	-1.177085443	7.327679361	-8.595356864	2.95E-08	1.18E-06	9.033784405
Lat	-1.184002726	6.989621968	-3.225202753	4.12E-03	1.60E-02	-2.841476403
Bst1	-1.185660161	7.161537891	-7.110392419	5.72E-07	1.36E-05	6.030864296
Traf1	-1.1879407	10.06478964	-11.67957438	1.49E-10	2.01E-08	14.36135354
Traj22	-1.189954805	5.284663302	-2.800200191	1.08E-02	3.44E-02	-3.766046112
Tcf7l2	-1.190539431	8.288708339	-10.29884423	1.39E-09	1.08E-07	12.1185881
Spint1	-1.192278753	7.095951847	-6.883659719	9.24E-07	2.04E-05	5.546204405
Laptm4b	-1.192923713	7.818255141	-8.589806823	2.98E-08	1.18E-06	9.023117087
Sema7a	-1.197364376	7.618505621	-8.156569983	6.87E-08	2.37E-06	8.177615646

Ccl2	-1.203484733	6.444811623	-3.723115039	1.29E-03	6.57E-03	-1.707641457
Thy1	-1.206963519	6.632655824	-3.857145414	9.38E-04	5.13E-03	-1.3964041
Nipal1	-1.212240617	6.501934289	-6.175041663	4.31E-06	7.09E-05	3.989248294
Dgkk	-1.21373729	6.404650083	-7.667774092	1.82E-07	5.22E-06	7.193097662
Igf2r	-1.218215562	6.252836095	-7.331293059	3.61E-07	9.26E-06	6.496489286
Mical3	-1.225786332	8.373186707	-9.005980323	1.37E-08	6.69E-07	9.811625172
Glipr2	-1.22861199	7.568716101	-9.200241285	9.57E-09	4.85E-07	10.17185981
Naalad2	-1.22990461	4.553482999	-4.878010928	8.38E-05	7.51E-04	1.002854182
Apoe	-1.247479685	8.081830388	-5.398377143	2.50E-05	2.91E-04	2.217774573
Plekhn1	-1.248531068	8.385330343	-8.163992438	6.77E-08	2.37E-06	8.192315134
Stfa2l1	-1.257313684	3.659557381	-4.621453762	1.53E-04	1.25E-03	0.399176813
Tmem150cos	-1.261041497	6.161443142	-5.008781086	6.17E-05	5.93E-04	1.309647236
Zbtb10	-1.270531705	8.14872384	-8.387415218	4.39E-08	1.59E-06	8.63128662
LOC102632809	-1.270695517	6.950672693	-5.175692014	4.18E-05	4.37E-04	1.699928695
St6gal1	-1.281221827	8.589884546	-12.07173204	8.21E-11	1.34E-08	14.96021261
Cd38	-1.282233206	7.193817602	-10.46361062	1.05E-09	8.63E-08	12.39774618
Trac	-1.282917197	8.733904043	-3.397192883	2.77E-03	1.18E-02	-2.454698765
Traj19	-1.294047157	3.678687285	-2.265761708	3.44E-02	8.40E-02	-4.839440444
Bcl2l14	-1.296420379	6.679585554	-9.411889948	6.52E-09	3.51E-07	10.55875559
Igkj1	-1.299474139	7.545239457	-2.357078737	2.84E-02	7.22E-02	-4.664807649
Src	-1.306366438	6.673639372	-10.14063147	1.82E-09	1.27E-07	11.84746509
Rgs1	-1.307165662	10.50565477	-10.36142879	1.25E-09	1.00E-07	12.22500471
Exoc3l4	-1.310695684	6.417277582	-8.248099026	5.74E-08	2.04E-06	8.35835801
Tmem176b	-1.324659092	9.772513397	-11.65241293	1.56E-10	2.02E-08	14.31927628
Mmp23	-1.324758779	6.964847608	-9.353660494	7.24E-09	3.79E-07	10.45288796
Socs2	-1.339444964	6.369648136	-7.13872712	5.39E-07	1.30E-05	6.090954197
Nuak1	-1.340978289	6.866511441	-5.780735716	1.04E-05	1.48E-04	3.097396722
H2-Eb2	-1.343483101	8.237811341	-6.664022639	1.48E-06	3.08E-05	5.070326485
Gm13710	-1.34456804	5.496354147	-3.359509442	3.02E-03	1.27E-02	-2.539949988
Loxl3	-1.350332497	6.642279417	-9.244228635	8.83E-09	4.56E-07	10.25274601
2200002D01Rik	-1.352359041	8.398306857	-9.989943017	2.36E-09	1.53E-07	11.58640536
Dnase1l3	-1.355696748	10.43426646	-11.71066001	1.42E-10	1.99E-08	14.40941361
Ccrl2	-1.360367092	8.37656918	-8.622564952	2.80E-08	1.18E-06	9.086019306
Tcf7	-1.367844951	8.206866171	-5.75821167	1.10E-05	1.55E-04	3.045958005
Epsti1	-1.373028794	10.99479522	-10.90153884	5.12E-10	4.82E-08	13.12419294
Pik3r3	-1.378200882	6.016700141	-5.652689563	1.39E-05	1.87E-04	2.804315074
Adora2a	-1.37889237	8.418815621	-8.847994382	1.83E-08	8.52E-07	9.515002838
0610040B10Rik	-1.382596171	6.627295527	-3.853302034	9.46E-04	5.17E-03	-1.405354379
Cd3g	-1.388248025	6.372259831	-4.023117938	6.33E-04	3.77E-03	-1.008709855
Pdlim4	-1.390107891	7.972144293	-10.28116099	1.43E-09	1.08E-07	12.08843498
Mmp25	-1.390789444	8.128123082	-7.428036619	2.96E-07	7.80E-06	6.698345303
LOC102638993	-1.400721899	7.922518274	-6.943174752	8.14E-07	1.82E-05	5.674077101
Icos	-1.402288805	8.472618963	-11.10377677	3.69E-10	3.66E-08	13.45223015
Gbp2	-1.413304822	7.326664642	-5.94322128	7.23E-06	1.10E-04	3.466936245
Olfr164	-1.415369444	5.330024857	-2.292102448	3.25E-02	8.04E-02	-4.789483638
Gem	-1.448378191	6.753656349	-7.588120882	2.13E-07	5.91E-06	7.029580727
Cd207	-1.450319752	6.744875284	-8.826986245	1.91E-08	8.76E-07	9.475310911
Cd8a	-1.454813733	8.739158364	-7.73872626	1.57E-07	4.68E-06	7.338025823
Foxp4	-1.468911554	8.455609935	-10.06113665	2.08E-09	1.38E-07	11.71009088
Il23r	-1.475646623	5.434826588	-4.287679735	3.38E-04	2.35E-03	-0.387259768
Gm16524	-1.476467282	7.133819505	-5.439295393	2.27E-05	2.73E-04	2.312520337
Igkc	-1.482682126	7.666143451	-2.181176867	4.09E-02	9.60E-02	-4.997487116
2210409E12Rik	-1.491866796	5.310097792	-4.925898441	7.49E-05	6.92E-04	1.115290484
Arhgap42	-1.532547779	5.257296872	-5.91121568	7.77E-06	1.18E-04	3.394363634
Serpinb9	-1.540598812	8.023507824	-7.916244817	1.10E-07	3.55E-06	7.697625287
Spata31d1b	-1.547273259	6.080334146	-5.173485698	4.20E-05	4.38E-04	1.694780697
Itgae	-1.548985151	5.754123003	-7.835695183	1.30E-07	4.02E-06	7.53498779
Glycam1	-1.580102957	5.807860298	-2.854948726	9.59E-03	3.12E-02	-3.649905735
Ier3	-1.587248196	8.03625432	-6.525250847	2.00E-06	3.86E-05	4.766491513
Traj12	-1.591515363	6.188481269	-2.619705152	1.62E-02	4.69E-02	-4.141549746
Traj18	-1.613997737	6.048889584	-3.146865271	4.94E-03	1.85E-02	-3.015515963
Il7r	-1.616054168	8.214495016	-6.132871849	4.73E-06	7.65E-05	3.894683208
Gbp5	-1.620976995	8.207441373	-7.647928745	1.89E-07	5.32E-06	7.152438617
Stap2	-1.621149975	8.184655574	-10.68323662	7.32E-10	6.58E-08	12.76485636
Il21r	-1.626572836	8.484907492	-7.198351563	4.76E-07	1.19E-05	6.217051265
Tnfrsf4	-1.63103031	8.344897055	-11.77245192	1.29E-10	1.95E-08	14.50464299
Gm9257	-1.632617684	4.315911572	-3.12799261	5.16E-03	1.92E-02	-3.057225739
Prnp	-1.652435547	7.1176222	-5.782123683	1.04E-05	1.48E-04	3.100564803

Cd3e	-1.658216574	6.375508994	-4.270888097	3.52E-04	2.41E-03	-0.426785937
2610034B18Rik	-1.674552604	8.096127708	-9.484337277	5.73E-09	3.18E-07	10.6898658
Gca	-1.692222333	8.433500794	-11.04981275	4.03E-10	3.89E-08	13.36515062
Adm	-1.70965815	5.621088435	-9.878141458	2.86E-09	1.74E-07	11.39091073
Atxn1	-1.727933495	7.02812664	-11.37212919	2.41E-10	2.75E-08	13.88044067
Slc4a8	-1.73665521	7.911053065	-10.68177148	7.33E-10	6.58E-08	12.76242606
Clu	-1.760484106	7.811548096	-7.48441563	2.64E-07	7.10E-06	6.815397109
Insl6	-1.763551604	7.184780897	-8.451044591	3.88E-08	1.45E-06	8.755067666
Cpne8	-1.778326484	4.713023765	-6.35747034	2.88E-06	5.27E-05	4.395959479
Lad1	-1.783417733	7.675622289	-10.52586002	9.50E-10	8.32E-08	12.5023735
Catsperg1	-1.78417998	6.01083675	-3.98070304	7.00E-04	4.05E-03	-1.107988247
Tmem176a	-1.788997244	8.96562728	-11.71937342	1.40E-10	1.99E-08	14.42286658
Tm4sf5	-1.802821975	7.421446797	-8.731838835	2.28E-08	1.02E-06	9.294809089
Mpzl2	-1.848263317	6.129227521	-10.27990626	1.44E-09	1.08E-07	12.08629402
Actn1	-1.849897112	8.808033297	-11.13806883	3.50E-10	3.66E-08	13.50739606
Tnf	-1.861703043	8.951195836	-14.27289285	3.69E-12	1.04E-09	18.04447981
Serpinb6b	-1.872539134	8.883593062	-11.97015413	9.57E-11	1.50E-08	14.8066323
Papss2	-1.87327357	7.845593061	-8.585750667	3.00E-08	1.18E-06	9.015318442
Mab21l3	-1.90348289	7.347695515	-11.11573604	3.62E-10	3.66E-08	13.47148409
Slco5a1	-1.904317523	7.734937526	-9.968073298	2.45E-09	1.56E-07	11.54828574
Cd8b1	-1.912686924	6.375170188	-5.234178822	3.65E-05	3.94E-04	1.836279102
Ccl19	-1.913118171	6.66566471	-8.452991167	3.87E-08	1.45E-06	8.758845819
Traj37	-1.923388967	3.780071184	-3.093916336	5.58E-03	2.03E-02	-3.132311335
Olfr109	-1.931489368	6.683633351	-7.306749344	3.80E-07	9.68E-06	6.445078008
Art2a-ps	-1.950293401	8.487501198	-8.610805808	2.86E-08	1.18E-06	9.063455921
Nr4a3	-1.962737337	8.756167861	-8.624642827	2.79E-08	1.18E-06	9.090004397
Gbp4	-1.974144195	7.301715279	-11.57730589	1.75E-10	2.12E-08	14.20251356
Gbp8	-1.986534544	9.647635722	-12.50099513	4.34E-11	8.60E-09	15.59766085
Gclc	-1.998605386	8.926358168	-20.90509046	2.31E-15	1.74E-12	25.17121623
Sema6d	-2.008283465	8.109943708	-8.153327569	6.91E-08	2.37E-06	8.171191995
Gm11837	-2.016096398	5.469303034	-11.29738005	2.71E-10	3.01E-08	13.76196362
Serpinb1a	-2.045616871	7.963415846	-8.768987474	2.12E-08	9.64E-07	9.365426161
Zfp872	-2.125857936	6.597179897	-8.592433692	2.96E-08	1.18E-06	9.02816651
Akap2	-2.132865733	8.127523956	-9.937619579	2.58E-09	1.62E-07	11.49510567
Zmynd15	-2.156070955	8.678656716	-11.61561966	1.65E-10	2.07E-08	14.26215228
Mt2	-2.192515977	7.279654706	-8.291978569	5.28E-08	1.89E-06	8.444604034
Jag1	-2.192878828	7.21121173	-12.17053344	7.07E-11	1.23E-08	15.108579
Asprv1	-2.199842242	7.089679277	-10.08182402	2.01E-09	1.38E-07	11.74591478
Apol10b	-2.257382919	6.892952355	-8.691510754	2.46E-08	1.08E-06	9.217939871
Gzmc	-2.268875612	4.500343425	-3.98440496	6.94E-04	4.04E-03	-1.099328364
Nudt17	-2.270996237	8.359305642	-13.57623537	9.43E-12	2.09E-09	17.11622356
Adcy6	-2.285640913	8.255273089	-10.50364253	9.86E-10	8.44E-08	12.46508342
Vcam1	-2.325809042	7.210051655	-14.23680135	3.87E-12	1.04E-09	17.99740659
Ccl5	-2.397169052	11.56374644	-18.91354553	1.65E-14	1.04E-11	23.30813992
Cacnb3	-2.423317694	8.08297635	-16.5842445	2.12E-13	1.14E-10	20.84821411
Gm15698	-2.481728646	5.716071614	-10.06251968	2.08E-09	1.38E-07	11.71248748
Gm8221	-2.560261981	7.407882758	-12.15807984	7.21E-11	1.23E-08	15.08993275
Cpa3	-2.632520328	5.410089988	-5.794347887	1.01E-05	1.45E-04	3.128458622
Ccr7	-2.664035522	9.670087648	-15.67552598	6.24E-13	2.94E-10	19.79328973
Il12b	-2.713441422	7.579085673	-14.5583463	2.54E-12	8.01E-10	18.41299473
Cd63	-2.834003333	9.88766756	-14.16775993	4.24E-12	1.07E-09	17.90705388
Mreg	-2.842697204	9.196542901	-24.2180945	1.24E-16	2.34E-13	27.87007213
Apol7c	-2.848135358	10.10859492	-13.94814775	5.69E-12	1.34E-09	17.61697279
Gadd45b	-2.863605084	10.13053105	-26.91096005	1.51E-17	5.68E-14	29.76084255
Anxa3	-2.911391355	9.445309531	-15.18024545	1.15E-12	4.68E-10	19.19320111
Ccl22	-3.139844221	10.1259617	-21.99889213	8.42E-16	7.93E-13	26.11326567
H2-M2	-3.163042469	9.339898873	-22.28004265	6.54E-16	7.93E-13	26.34689463
Fscn1	-3.6072096	9.717929982	-15.12009122	1.24E-12	4.68E-10	19.11905551
Mcpt4	-3.854927438	4.669062579	-4.936288172	7.31E-05	6.79E-04	1.139671726
