# Supplementary material for: CD8+XCR1neg Dendritic Cells Express High Levels of Toll-Like Receptor 5 and a Unique Complement of Endocytic Receptors
Source: Front Immunol. 2019 Jan 16;9:2990. doi: 10.3389/fimmu.2018.02990 (PMC6343586; doi:10.3389/fimmu.2018.02990)
Supplement: Supplementary Table 3 — Differentially expressed genes between CD8+XCR1neg and CD8+XCR1+ DC in the sdLNs. Differentially expressed genes between CD8+XCR1+ and CD8+XCR1neg DC subsets. The data are derived from TopTable analysis with an FDR < 0.05. [file Table_3.PDF]

Table S3. Differentially expressed genes between CD8+XCR1neg and CD8+XCR1+ DC in the sdLNs

Differentially expressed genes between CD8+XCR1+ and CD8+XCR1neg DC subsets
The data are derived from TopTable analysis with an FDR<0.05.

Gene ID	Log2FC	AveExpr	t	P.Value	adj.P.Value	B
Klra17	6.072861774	4.579093567	16.10956462	3.70E-13	6.40E-12	20.165657
Cd209d	5.892082331	6.075770433	13.45013605	1.12E-11	1.22E-10	16.66457532
Cd209a	5.837200752	7.100332225	26.82177224	1.61E-17	3.57E-15	30.32138283
Dab2	5.583169399	5.182242888	17.28908521	9.48E-14	2.17E-12	21.55822959
Klk1	5.563107888	6.247160152	13.93721204	5.77E-12	6.84E-11	17.34738554
Cyp7b1	5.297062284	5.261113443	27.18800766	1.23E-17	3.08E-15	30.59185537
Cd209e	5.241002017	4.555078342	22.88598984	3.84E-16	3.15E-14	27.14845509
Mgl2	5.193731705	5.008933739	19.48861916	9.19E-15	3.33E-13	23.93665053
Lifr	4.846039418	5.738064486	22.33138944	6.25E-16	4.46E-14	26.6574716
Apod	4.836495725	6.283307869	16.51960258	2.28E-13	4.26E-12	20.65987597
Ly6d	4.812265051	6.744232264	16.94669697	1.40E-13	2.89E-12	21.16306913
Olfr164	4.744206276	5.330024857	7.682945867	1.76E-07	6.98E-07	6.720528423
Clec10a	4.738178041	4.969611722	16.03808592	4.03E-13	6.84E-12	20.07835697
Cybb	4.612488955	8.133423387	16.11166503	3.69E-13	6.40E-12	20.1682171
Tlr5	4.604552878	5.867738111	21.30581288	1.59E-15	9.24E-14	25.71691591
Spib	4.557095796	6.878346384	21.2888307	1.61E-15	9.24E-14	25.7009703
Clec4g	4.486544718	5.593282718	18.25218205	3.31E-14	8.84E-13	22.63246137
Gfra2	4.437923431	6.940531456	19.74446665	7.11E-15	2.76E-13	24.19671883
Dntt	4.43328987	4.035063744	17.89221526	4.87E-14	1.21E-12	22.23721922
Cd7	4.390096701	7.934175545	19.15271798	1.29E-14	4.20E-13	23.59023647
Cd33	4.363406183	6.718020597	15.94158267	4.52E-13	7.48E-12	19.95994355
Siglech	4.297254784	7.092461451	15.8060999	5.33E-13	8.61E-12	19.79262446
Olfr111	4.292505477	5.012727662	7.683807291	1.76E-07	6.97E-07	6.722315173
Gm12253	4.288628223	3.42687596	17.0108706	1.30E-13	2.76E-12	21.23768351
Mctp2	4.286754025	5.066018869	17.10361158	1.17E-13	2.58E-12	21.34506246
Il1r1	4.278992675	5.033624163	15.40860544	8.66E-13	1.30E-11	19.29432617
Gm4956	4.275711012	5.336935621	16.25388349	3.12E-13	5.62E-12	20.34087373
Pltp	4.267457419	6.553213191	20.10456673	4.98E-15	2.16E-13	24.55735714
Ighj1	4.264008449	8.666769349	15.56100573	7.18E-13	1.12E-11	19.48669502
Lag3	4.251130473	6.76458427	20.84313303	2.45E-15	1.26E-13	25.27798204
Ackr1	4.246436261	4.950229332	15.62158834	6.67E-13	1.05E-11	19.56270737
LOC102632602	4.244710397	5.74687902	14.2406344	3.85E-12	4.89E-11	17.76274314
Il1rl2	4.236886427	3.867068557	24.30119497	1.16E-16	1.74E-14	28.34926211
Gm5086	4.220744335	5.705982701	19.24227249	1.18E-14	3.97E-13	23.6831534
Tlr7	4.155706239	5.931645235	12.96908474	2.20E-11	2.17E-10	15.96998137
LOC102636133	4.141052371	4.043773346	15.84223322	5.10E-13	8.32E-12	19.83737221
Ddr1	4.140735342	5.913729409	20.39409755	3.76E-15	1.71E-13	24.84285229
Cadm3	4.12411663	6.286745477	22.43112842	5.72E-16	4.29E-14	26.74665692
2610528A11Rik	4.107939343	6.423871747	11.58404158	1.73E-10	1.35E-09	13.84887297
Wfdc17	4.090413088	7.848634158	16.72989606	1.79E-13	3.56E-12	20.90908828
Irf4	4.077915853	7.426673453	24.60736714	9.05E-17	1.42E-14	28.59973916
Cox6a2	4.070225503	6.771397706	23.42171225	2.42E-16	2.47E-14	27.61158207
Ptprf	4.030551974	4.872843688	17.70436267	5.98E-14	1.44E-12	22.02802595
Klk1b27	4.017698714	4.601251168	10.40294714	1.17E-09	7.45E-09	11.88338971
Lrrc16a	4.013610637	7.51026264	28.80795954	3.83E-18	1.61E-15	31.74392989
Efnb2	3.981732626	5.410776521	16.97358319	1.35E-13	2.85E-12	21.19436077
Cd209c	3.967997189	4.869990423	15.46062632	8.12E-13	1.23E-11	19.36017664
Kdr	3.960809643	4.942684479	22.25657499	6.68E-16	4.62E-14	26.59031403
Atp1b1	3.956853748	6.698861839	20.62684895	3.01E-15	1.46E-13	25.06954023
Gria3	3.951787172	6.160128071	19.33075147	1.08E-14	3.69E-13	23.7745531
Pi16	3.857176514	5.014210275	14.78582646	1.90E-12	2.64E-11	18.49059339
Blnk	3.805839475	6.621089958	18.21948845	3.43E-14	9.09E-13	22.59686417
Zeb2os	3.79787509	5.441255385	7.601323506	2.08E-07	8.10E-07	6.550783272
Cd200r1	3.765141989	5.269014038	14.43583368	2.98E-12	3.96E-11	18.0260243
BC022960	3.720304114	5.462656474	10.94291327	4.79E-10	3.40E-09	12.80099923
Klrd1	3.717095961	8.063558152	18.89641235	1.68E-14	5.28E-13	23.32202115
Cd4	3.697328616	7.432322213	18.10570878	3.87E-14	9.99E-13	22.47251472
Adam11	3.688978135	8.34307666	24.61760692	8.97E-17	1.42E-14	28.60806134
Cd200r4	3.677882861	4.470976845	11.96853668	9.59E-11	8.00E-10	14.45662445
Fabp5	3.676441714	6.53428505	16.66306486	1.93E-13	3.74E-12	20.83019608
Xkrx	3.670172991	4.122266501	20.62527237	3.01E-15	1.46E-13	25.06801306
Igkv1-110	3.668252012	3.458990896	4.12580938	4.96E-04	1.02E-03	-1.35091252
Ctsl	3.635052895	7.135945247	18.21125275	3.46E-14	9.11E-13	22.58788767
Zeb2	3.621060458	7.499516479	16.27488874	3.04E-13	5.53E-12	20.36626028
Ighj4	3.61144414	6.154665853	5.388770939	2.55E-05	6.81E-05	1.63530722
Igkj3	3.580048614	3.886220205	9.195066086	9.66E-09	4.99E-08	9.706443627
Sh2d1b1	3.571959451	4.516968073	10.27533165	1.45E-09	9.01E-09	11.66165284
Pgap1	3.570502965	6.09773425	16.79119949	1.67E-13	3.40E-12	20.98120533
Khdc1a	3.566906056	5.435765384	9.92599841	2.63E-09	1.52E-08	11.04487531
Plaur	3.501894873	6.106381584	14.83737337	1.78E-12	2.52E-11	18.55821757

Gm5431	3.495287113	5.519611975	11.74357418	1.35E-10	1.09E-09	14.10287271
Ceacam15	3.48321068	4.131645534	8.677520099	2.52E-08	1.18E-07	8.718082814
Ptgs2	3.481275147	7.591863572	13.97848607	5.46E-12	6.49E-11	17.40432918
Sirpa	3.473739137	7.524130947	16.34552194	2.80E-13	5.17E-12	20.451412
Rgl1	3.461054225	6.876479192	22.24587634	6.75E-16	4.62E-14	26.58069207
Emr4	3.440094273	6.868054266	11.717421	1.41E-10	1.13E-09	14.06141269
Gm5547	3.424799451	6.813177236	15.49447782	7.79E-13	1.20E-11	19.40292338
L1cam	3.413530682	6.522586459	21.25548166	1.66E-15	9.24E-14	25.66962063
Myo1e	3.393004704	5.99485464	13.08862517	1.86E-11	1.88E-10	16.14451614
Csf1r	3.343899606	7.587100798	16.25026728	3.13E-13	5.62E-12	20.33650029
Mir99a	3.341950093	3.855166105	10.54252191	9.24E-10	6.10E-09	12.1237504
Rin2	3.337887874	5.804000711	18.2608853	3.28E-14	8.82E-13	22.64192757
Atp2b4	3.33428611	6.118651759	18.33894671	3.02E-14	8.27E-13	22.72664473
Cdh1	3.309284076	5.209923455	17.73370608	5.79E-14	1.40E-12	22.06083741
Podn	3.305539512	4.375502883	15.60620081	6.79E-13	1.07E-11	19.54342538
Atp1a3	3.287273027	5.24782351	11.33057677	2.57E-10	1.92E-09	13.43985888
Pdlim1	3.247781835	6.176726668	11.36146972	2.45E-10	1.85E-09	13.49007352
Gfpt2	3.239025591	6.131373732	11.66428182	1.53E-10	1.22E-09	13.97695584
Ptgr1	3.23878191	4.878237233	11.20754012	3.13E-10	2.31E-09	13.23885877
Gpr183	3.206191104	8.736456522	23.01158254	3.44E-16	3.05E-14	27.25799493
Ighj3	3.203455393	5.260347345	5.358917206	2.73E-05	7.27E-05	1.56546603
Tcf4	3.186589317	8.23638794	25.42059384	4.72E-17	8.90E-15	29.2498934
Ccl9	3.181149425	6.568642601	16.04341613	4.00E-13	6.83E-12	20.08487891
Havcr1	3.165284967	5.413989509	11.57909142	1.74E-10	1.36E-09	13.84094944
Lrp8	3.134697083	6.979692763	25.565416	4.21E-17	8.36E-15	29.36343429
Cd8b1	3.121535448	6.375170188	8.542262996	3.26E-08	1.48E-07	8.454075608
Il7r	3.119167146	8.214495016	11.83713563	1.17E-10	9.58E-10	14.25062087
Gpm6b	3.115341335	5.113366433	16.56819459	2.16E-13	4.07E-12	20.7177135
Mrc1	3.11341586	4.27000146	10.21344082	1.61E-09	9.83E-09	11.55343083
Sirpb1a	3.104945718	7.286791986	12.39188533	5.09E-11	4.57E-10	15.10865964
Srl	3.10252827	5.222755018	21.00563758	2.10E-15	1.11E-13	25.43321776
Cx3cr1	3.084036714	7.523496785	22.93121529	3.69E-16	3.09E-14	27.18796897
Smim5	3.079417319	5.613966384	14.17335955	4.21E-12	5.27E-11	17.67129673
Igkv14-111	3.066065739	3.269126637	10.40508307	1.16E-09	7.44E-09	11.8870849
Pros1	3.064312178	4.653528898	14.11815141	4.53E-12	5.56E-11	17.59597888
Runx2os2	3.061156477	5.76179546	12.69838372	3.25E-11	3.07E-10	15.56991217
Retnla	3.058183561	4.546896247	6.14995103	4.55E-06	1.40E-05	3.389432217
Clec4a3	3.057670549	7.408177136	7.211299098	4.63E-07	1.71E-06	5.72754673
Cd300a	3.048008152	6.707110784	11.10983889	3.66E-10	2.65E-09	13.07808748
Cmah	3.043880208	5.646292456	10.98876521	4.44E-10	3.19E-09	12.87741548
Il9r	3.041590206	6.058439822	17.83764218	5.17E-14	1.27E-12	22.17665621
Dscam	3.015425878	6.025116048	16.27252523	3.05E-13	5.53E-12	20.36340525
Fabp4	3.011404685	4.93697196	5.827210891	9.38E-06	2.69E-05	2.652484083
Iglv2	3.004920033	3.519875654	4.967236065	6.80E-05	1.67E-04	0.643779183
Igkc	2.997559014	7.666143451	4.409715516	2.53E-04	5.52E-04	-0.67823365
Rasgrp2	2.99484718	5.783840734	11.73318744	1.37E-10	1.11E-09	14.08641526
Slco4a1	2.989251576	4.660285151	10.28211297	1.43E-09	8.92E-09	11.67348337
Iglv3	2.988176791	4.975499163	9.492429931	5.65E-09	3.08E-08	10.25892861
Capsl	2.978089608	5.325590319	13.13485714	1.74E-11	1.79E-10	16.21167136
Lair1	2.972885387	7.40769702	8.74700031	2.21E-08	1.06E-07	8.852774795
Cd79b	2.951538469	6.625056333	14.47776264	2.82E-12	3.79E-11	18.08218292
Rasgrp1	2.950322027	6.51362526	11.73381765	1.37E-10	1.11E-09	14.08741412
Dirc2	2.947110829	7.017556715	21.18945017	1.77E-15	9.66E-14	25.60740602
Sirpb1b	2.94649369	5.789849001	7.596128628	2.10E-07	8.16E-07	6.539949955
Slc15a3	2.935334811	7.45833855	22.32732686	6.27E-16	4.46E-14	26.65383053
LOC101055907	2.934788224	5.208557408	5.390713789	2.54E-05	6.78E-05	1.639850103
B930041F14Rik	2.934208522	5.652051417	16.20814602	3.29E-13	5.84E-12	20.28549478
Tmem176a	2.932747165	8.96562728	19.21185696	1.22E-14	4.02E-13	23.65164187
Pdcd1lg2	2.929694729	6.437592388	12.52782731	4.17E-11	3.82E-10	15.31434062
Stra6l	2.920312276	7.104218902	13.35624779	1.28E-11	1.37E-10	16.5306181
Clec4b1	2.919163342	4.455076531	10.64598866	7.78E-10	5.25E-09	12.30049122
Scn3a	2.911659154	5.489795404	11.46944302	2.07E-10	1.59E-09	13.66478341
Igkj5	2.904520273	7.116643771	7.291976877	3.92E-07	1.46E-06	5.899473264
Klri2	2.900305961	5.958130431	10.55346589	9.07E-10	6.01E-09	12.14250248
Nrp2	2.891581471	6.163040967	17.00275549	1.31E-13	2.77E-12	21.22826223
Lynx1	2.887778918	5.517728641	17.93764888	4.64E-14	1.18E-12	22.2875095
Klf12	2.883998499	4.250214825	12.87578236	2.52E-11	2.45E-10	15.83285217
BE692007	2.850411983	5.353110861	6.790084401	1.13E-06	3.85E-06	4.816283762
Tmod2	2.83553982	5.444923594	13.22392727	1.54E-11	1.60E-10	16.34051299
Slc2a3	2.83550492	6.392282222	10.79348788	6.10E-10	4.23E-09	12.55035397
Arap2	2.833760515	7.487372543	25.90742616	3.23E-17	6.76E-15	29.62896109
Abca9	2.822272251	5.235872773	11.86250095	1.13E-10	9.26E-10	14.29052317
Ceacam1	2.819894236	7.351868927	13.34650427	1.30E-11	1.38E-10	16.51667215
Mmp12	2.797188938	6.593020593	9.05548556	1.25E-08	6.28E-08	9.44326552
Pla2g4f	2.795570781	6.05381645	15.46852808	8.04E-13	1.23E-11	19.37016209
Mucl1	2.78260824	3.922006648	4.909033023	7.79E-05	1.89E-04	0.506123655

Dkk2	2.779058514	4.815850906	14.16098392	4.28E-12	5.34E-11	17.65443476
Slamf9	2.763215417	6.664319543	14.18740707	4.13E-12	5.19E-11	17.69042165
Sell	2.761444895	7.831860346	14.4597604	2.89E-12	3.86E-11	18.05808813
Clec4a1	2.75710341	6.687183147	10.12875131	1.86E-09	1.13E-08	11.40461341
Tmem176b	2.755536661	9.772513397	24.23918064	1.22E-16	1.74E-14	28.29813829
Atrnl1	2.752670906	7.620135965	19.23303635	1.19E-14	3.97E-13	23.67358943
Ctnnd2	2.733357436	6.170108896	18.97530194	1.55E-14	4.95E-13	23.40494022
Micu1	2.722639359	7.863483171	18.55652709	2.40E-14	7.11E-13	22.96101279
Chil5	2.721882814	6.259129028	14.3692387	3.25E-12	4.24E-11	17.93654315
B4galt6	2.714436411	7.616353071	23.00049481	3.48E-16	3.05E-14	27.24834846
Vopp1	2.704868204	7.323338617	23.6013833	2.08E-16	2.31E-14	27.7645298
Gm14548	2.698497542	7.357601006	9.732827332	3.69E-09	2.06E-08	10.69754203
Bhlhe40	2.694740367	8.641581431	16.20032973	3.32E-13	5.85E-12	20.27601686
Slc7a11	2.692651729	5.054615481	6.884772132	9.21E-07	3.19E-06	5.023105373
Ifitm2	2.660405974	8.903635541	14.01288477	5.22E-12	6.26E-11	17.45167987
Sdc4	2.654819256	7.233748444	14.80667724	1.85E-12	2.60E-11	18.51797181
Igkv10-96	2.653774447	3.01409125	4.202770317	4.13E-04	8.67E-04	-1.168852143
Igkj1	2.631730491	7.545239457	4.773620186	1.07E-04	2.54E-04	0.185379166
Emr1	2.620750244	6.304217259	14.90586449	1.63E-12	2.33E-11	18.64775877
Ets2	2.610658093	6.114770552	10.57266697	8.79E-10	5.86E-09	12.1753697
Ckap4	2.605954972	5.940182469	12.86873967	2.54E-11	2.47E-10	15.82246896
Zdhhc15	2.596664684	5.807321038	12.4238633	4.86E-11	4.41E-10	15.1572017
Pecam1	2.585180273	6.861937052	20.46259478	3.52E-15	1.64E-13	24.90982348
Fam65b	2.582848784	7.260962332	10.33638511	1.30E-09	8.21E-09	11.76797189
Traj40	2.565575829	3.340910179	4.072260452	5.63E-04	1.15E-03	-1.477406011
Ly6c2	2.556572269	8.737711394	10.8398635	5.66E-10	3.94E-09	12.6284098
Gm9733	2.548875392	5.384009065	6.262420439	3.55E-06	1.11E-05	3.643604554
Ldlrad3	2.545119856	5.741797787	12.25953223	6.19E-11	5.48E-10	14.90669918
Zhx2	2.541245837	6.617276123	13.87713131	6.26E-12	7.30E-11	17.26424285
Sox4	2.538166606	6.115738839	13.49977634	1.05E-11	1.14E-10	16.7350908
Ccl17	2.527207199	7.23637869	8.622591788	2.80E-08	1.29E-07	8.611156392
Clec4a2	2.516588621	4.46171855	10.84084801	5.65E-10	3.94E-09	12.63006424
Traj19	2.513265022	3.678687285	4.400503966	2.59E-04	5.63E-04	-0.700093722
Traj34	2.476008361	3.420676784	5.156709979	4.37E-05	1.11E-04	1.090781069
Cd274	2.4703724	8.431430832	19.46596	9.40E-15	3.36E-13	23.91346111
Cdc42ep2	2.465970276	6.821108363	14.42125259	3.04E-12	4.00E-11	18.00646225
Prkca	2.460007279	4.744676194	10.11568654	1.90E-09	1.15E-08	11.38158043
Pacsin1	2.452665841	5.628642607	10.52538106	9.50E-10	6.24E-09	12.09435275
Cd300lf	2.447843599	6.012179153	8.377849475	4.47E-08	1.98E-07	8.129935614
Clec2f	2.445502328	3.784587797	4.058570388	5.82E-04	1.19E-03	-1.509717297
Traj38	2.440125872	4.033074388	3.334615311	3.20E-03	5.67E-03	-3.190396703
Runx2	2.43940525	8.177669998	20.39372869	3.76E-15	1.71E-13	24.84249107
Stat4	2.439356921	7.914666835	12.73723556	3.07E-11	2.92E-10	15.62774815
Rem1	2.421476542	6.445426935	13.10951993	1.81E-11	1.83E-10	16.17489103
Sned1	2.41834584	6.617780335	11.60763435	1.67E-10	1.31E-09	13.88660202
Cst7	2.408813173	6.569860835	12.214968	6.62E-11	5.80E-10	14.83831427
Sema4b	2.401611565	6.39146451	13.44313731	1.13E-11	1.22E-10	16.6546162
Traj27	2.38632791	4.131222246	4.1230615	4.99E-04	1.03E-03	-1.357407505
Gm16525	2.366164965	4.05952705	4.161404635	4.56E-04	9.48E-04	-1.266742946
Il1r2	2.363706707	6.046481229	11.6439919	1.58E-10	1.25E-09	13.9446312
St8sia1	2.361599239	6.850724999	15.74032763	5.77E-13	9.29E-12	19.71093925
Cnn3	2.344232178	6.723065266	7.940377526	1.05E-07	4.34E-07	7.250110651
Snrnp25	2.32632046	9.09904644	16.33020797	2.85E-13	5.24E-12	20.43297826
Ms4a4c	2.316443413	6.976595002	9.353630409	7.24E-09	3.87E-08	10.00242603
Fcer1g	2.312904813	10.27034666	22.71118383	4.47E-16	3.44E-14	26.99498982
Setbp1	2.304712519	5.916199826	10.10382902	1.94E-09	1.17E-08	11.36065834
Man1a	2.302017323	8.470232544	19.95969619	5.75E-15	2.38E-13	24.41301968
Plk2	2.293577247	7.261903847	22.41470821	5.81E-16	4.29E-14	26.73200125
LOC102632682	2.289695144	5.901749515	2.693289571	1.37E-02	2.14E-02	-4.591549284
Stambpl1	2.288950015	6.595963499	10.04794134	2.13E-09	1.26E-08	11.26182214
Gngt2	2.288529579	7.195538512	15.81419154	5.27E-13	8.57E-12	19.80265305
Fcor	2.286081352	7.356809093	9.029123909	1.31E-08	6.58E-08	9.393282375
Ms4a6c	2.283547313	7.748411	10.08010859	2.02E-09	1.21E-08	11.31875457
Ace	2.27138249	6.226516113	7.075111402	6.16E-07	2.21E-06	5.435407006
Olfr165	2.271207653	2.996731939	8.282927852	5.37E-08	2.35E-07	7.941180854
Snx18	2.268402579	7.551678136	27.78614847	7.92E-18	2.41E-15	31.02548171
Traj43	2.243417455	5.964098492	5.027355492	5.91E-05	1.46E-04	0.785809914
Emb	2.240233019	7.983373404	10.03956329	2.16E-09	1.28E-08	11.24697372
Gatsl2	2.237525736	7.740080245	15.47424189	7.99E-13	1.22E-11	19.37737984
Eepd1	2.234939632	5.632225166	13.03388621	2.01E-11	2.00E-10	16.06475515
Anxa3	2.217433498	9.445309531	11.56188937	1.79E-10	1.39E-09	13.81339492
Ptpro	2.217233838	6.646402938	14.47709684	2.83E-12	3.79E-11	18.08129224
Cxcl1	2.21329032	5.322661922	8.466238349	3.77E-08	1.70E-07	8.304634268
Apobec1	2.194532228	7.821431361	19.77020879	6.93E-15	2.74E-13	24.22270745
Fxyd2	2.190876022	6.188534062	11.19952549	3.17E-10	2.33E-09	13.22570921
Tex2	2.182019979	7.303932274	12.70577696	3.22E-11	3.05E-10	15.58092882

Ptchd1	2.171985886	6.141948947	10.95849439	4.67E-10	3.33E-09	12.82699248
Ctsw	2.169411748	6.700542372	9.248419747	8.76E-09	4.57E-08	9.806389231
Ctsa	2.167497836	8.771278221	14.08547463	4.73E-12	5.74E-11	17.55128269
Usp11	2.16710102	7.320301731	18.79039094	1.88E-14	5.75E-13	23.21006966
A530064D06Rik	2.16243459	4.824906537	4.828211493	9.42E-05	2.26E-04	0.314757879
Hpse	2.152694041	7.745905087	19.76455064	6.97E-15	2.74E-13	24.2169979
Sema6d	2.15059843	8.109943708	8.731104837	2.28E-08	1.08E-07	8.822015654
Traj7	2.147799383	3.658119965	7.127973018	5.51E-07	2.00E-06	5.549087136
Clu	2.143274735	7.811548096	9.111788556	1.12E-08	5.72E-08	9.549722384
5430437J10Rik	2.119268245	7.554636855	10.08354965	2.01E-09	1.20E-08	11.32483758
Traj22	2.109460159	5.284663302	4.963979063	6.85E-05	1.68E-04	0.636079868
Gimap6	2.107368387	6.634162901	6.801152603	1.10E-06	3.77E-06	4.840517699
Abi3	2.105272402	7.532042125	16.20651561	3.30E-13	5.84E-12	20.28351811
Serinc5	2.095025312	7.377011638	17.93327354	4.66E-14	1.18E-12	22.28267158
Aldh1a2	2.088870391	5.543874307	7.194479896	4.80E-07	1.76E-06	5.691597676
Cd300c	2.088841954	7.270851524	9.571073314	4.91E-09	2.70E-08	10.40319966
Ticam2	2.086598808	6.536630763	13.1283179	1.76E-11	1.80E-10	16.2021843
Bst2	2.076367933	8.850016638	12.04309575	8.57E-11	7.26E-10	14.57274273
Samsn1	2.028409246	9.734249563	17.17208771	1.08E-13	2.41E-12	21.42400722
Lilrb4	2.025451073	8.476135627	11.14497666	3.46E-10	2.52E-09	13.13602707
Timd4	2.024219298	5.872775417	6.478339624	2.21E-06	7.20E-06	4.127498646
Syne2	2.022709342	6.554340539	15.98168704	4.31E-13	7.25E-12	20.00923026
Maged1	2.00931574	6.274701917	9.577783852	4.85E-09	2.67E-08	10.41547472
Lysmd2	2.006222643	5.659202189	9.756117184	3.54E-09	1.99E-08	10.73965862
St8sia6	2.001317656	7.887947463	11.15903466	3.38E-10	2.47E-09	13.15917018
Ptafr	1.991127773	8.032854137	12.6908877	3.29E-11	3.09E-10	15.55873717
S100a4	1.98998621	11.89356083	12.50850405	4.29E-11	3.92E-10	15.28521202
Slco5a1	1.988484501	7.734937526	10.40864195	1.16E-09	7.41E-09	11.89324062
Rilpl1	1.987003797	6.284855872	10.07491331	2.04E-09	1.21E-08	11.30956784
Ugcg	1.986541013	7.129474622	12.07364252	8.18E-11	7.02E-10	14.62015619
Clec2i	1.982332975	8.423080457	13.6816105	8.16E-12	9.13E-11	16.99157989
Marveld1	1.979448306	8.360815324	15.95550585	4.45E-13	7.42E-12	19.97706704
Slc44a2	1.972685662	8.683854448	17.43064648	8.10E-14	1.88E-12	21.71952694
Fam43a	1.965573012	6.87647787	11.86180862	1.13E-10	9.26E-10	14.28943492
Cercam	1.964278679	6.274143539	14.388139	3.17E-12	4.17E-11	17.96197447
Igkv4-91	1.952328282	5.479867776	3.026768308	6.50E-03	1.08E-02	-3.87740063
Crispld2	1.951537749	6.203753672	11.88677448	1.09E-10	8.98E-10	14.32864692
Tcf7	1.947836477	8.206866171	8.199799785	6.31E-08	2.72E-07	7.774902183
Cd3d	1.94231025	6.971837659	5.339999693	2.86E-05	7.56E-05	1.521175571
Papss2	1.938812599	7.845593061	8.886134855	1.71E-08	8.41E-08	9.120616925
Cd72	1.92825563	7.047166943	12.77267189	2.92E-11	2.79E-10	15.68037709
Acap1	1.9240058	6.965988276	8.20438336	6.25E-08	2.70E-07	7.784094345
Mtss1	1.920017177	6.961106159	13.71473018	7.80E-12	8.78E-11	17.03799357
Cyp4f16	1.914574722	5.766852292	9.230512118	9.05E-09	4.72E-08	9.772883562
Tnfrsf9	1.912337939	6.853810243	10.91372283	5.02E-10	3.56E-09	12.75223001
Ifitm1	1.910585156	5.029102203	9.320034445	7.70E-09	4.08E-08	9.93997852
H2-M2	1.908754673	9.339898873	13.44500933	1.13E-11	1.22E-10	16.65728048
Traj31	1.894394137	3.870988682	3.771562747	1.15E-03	2.20E-03	-2.183719909
Ccr9	1.887825267	6.708486551	7.487481542	2.62E-07	1.00E-06	6.312561893
Slc2a6	1.882616604	7.823917221	13.81143537	6.84E-12	7.86E-11	17.17298519
Elk3	1.870678963	8.592059914	17.82397715	5.25E-14	1.29E-12	22.16146451
Sla2	1.87016535	6.011888199	5.955557051	7.03E-06	2.07E-05	2.946852248
Man2a2	1.868584486	6.199859691	8.061874325	8.27E-08	3.48E-07	7.496998984
Ndnf	1.861257127	6.570422939	6.42233106	2.50E-06	8.02E-06	4.002503078
Gm14719	1.8593922	6.925941396	6.387518448	2.70E-06	8.63E-06	3.924624141
Tnip3	1.855703496	5.765577922	6.261310085	3.56E-06	1.12E-05	3.641102206
Aire	1.849818996	6.375042564	8.003785287	9.27E-08	3.86E-07	7.379202862
Gm14446	1.843779863	6.114111763	5.553044251	1.75E-05	4.81E-05	2.018372181
Gm614	1.843480954	6.875624682	8.697401629	2.43E-08	1.14E-07	8.756688512
Adm	1.843169514	5.621088435	10.64954956	7.74E-10	5.23E-09	12.30655226
Cd3g	1.839903552	6.372259831	5.332007575	2.91E-05	7.68E-05	1.502456309
Cxcl2	1.838280671	6.120702276	4.043901412	6.03E-04	1.23E-03	-1.544325731
Pcyt1a	1.837509686	7.701833456	14.97752752	1.49E-12	2.15E-11	18.74106784
Gsr	1.832304113	7.635480314	13.83772596	6.60E-12	7.61E-11	17.20954853
Lrp1	1.830061289	5.846875761	8.071983349	8.11E-08	3.42E-07	7.517452951
Ifitm6	1.813318014	5.901364061	7.561819728	2.25E-07	8.68E-07	6.468313119
Trac	1.807048741	8.733904043	4.785104712	1.04E-04	2.48E-04	0.212603971
LOC73899	1.806135806	8.472098309	14.10579553	4.61E-12	5.64E-11	17.57908841
Il2ra	1.804672353	6.1703802	7.894961243	1.15E-07	4.73E-07	7.157319749
Traj17	1.800953651	4.945568361	3.303501796	3.44E-03	6.03E-03	-3.260818061
Traj39	1.789838769	5.323567993	3.950973385	7.51E-04	1.49E-03	-1.763219095
LOC102638993	1.78468737	7.922518274	8.846435751	1.84E-08	9.00E-08	9.044448734
Il2rg	1.782021083	10.20092691	18.93013908	1.62E-14	5.14E-13	23.35751039
Gm16894	1.775539324	8.134267681	8.651838573	2.65E-08	1.24E-07	8.668138664
Il31ra	1.774802834	5.148853816	10.2552217	1.50E-09	9.27E-09	11.62653789
Pnkd	1.77235309	6.585197287	5.340155621	2.86E-05	7.56E-05	1.521540743

Adora2a	1.772091807	8.418815621	11.3710531	2.41E-10	1.83E-09	13.50563012
Traj18	1.771079219	6.048889584	3.453132281	2.43E-03	4.42E-03	-2.920411603
Cd3e	1.770847534	6.375508994	4.560979414	1.77E-04	3.99E-04	-0.319162737
Mcemp1	1.770412231	8.716568471	19.66365551	7.71E-15	2.90E-13	24.11492327
LOC102632070	1.770160733	5.871555446	8.380012269	4.45E-08	1.98E-07	8.134222581
Bmp1	1.767780994	5.386705702	9.218851651	9.25E-09	4.81E-08	9.751044744
Tpp1	1.765893903	7.566488397	15.26219163	1.04E-12	1.55E-11	19.1079408
Gm15987	1.763333692	9.173930624	14.98346972	1.47E-12	2.14E-11	18.7487876
Lef1	1.762522817	5.742376523	4.988346414	6.47E-05	1.60E-04	0.693671019
Gm5796	1.761430553	6.643001926	3.574770056	1.83E-03	3.40E-03	-2.640745961
Nipal1	1.758229245	6.501934289	8.95625727	1.50E-08	7.48E-08	9.254660894
Traj23	1.758012597	5.38865494	5.65552195	1.39E-05	3.86E-05	2.256193501
Npc1	1.748709932	7.596333075	14.87222138	1.70E-12	2.42E-11	18.60382016
Fscn1	1.746796808	9.717929982	7.321927473	3.68E-07	1.38E-06	5.963082319
Ank	1.742535754	7.387475783	12.39331618	5.08E-11	4.57E-10	15.11083375
Nxpe4	1.74084073	5.70430767	7.745250654	1.55E-07	6.22E-07	6.849506196
Npr2	1.734637278	5.347534335	9.438947753	6.21E-09	3.36E-08	10.1603775
Cox6b2	1.734086304	6.884386771	8.62494155	2.79E-08	1.29E-07	8.615738613
Traj37	1.730486263	3.780071184	2.783617776	1.12E-02	1.78E-02	-4.401737166
Lamp1	1.728325317	10.05206597	18.86700102	1.73E-14	5.40E-13	23.29102411
Spata31d1b	1.724930047	6.080334146	5.767501558	1.07E-05	3.04E-05	2.514980614
Ehf	1.72279321	7.074262335	8.731039163	2.28E-08	1.08E-07	8.821888502
Ptpn3	1.721937904	7.47262139	15.71569972	5.94E-13	9.49E-12	19.68027538
Tfrc	1.719459404	7.153072695	8.887254462	1.70E-08	8.41E-08	9.122762101
LOC102640356	1.718832562	6.566159049	8.014522064	9.08E-08	3.79E-07	7.401009166
Fam107b	1.718482497	8.392378174	16.78142255	1.69E-13	3.41E-12	20.96971975
Ctsd	1.717541133	8.140930245	14.09976473	4.64E-12	5.66E-11	17.57083978
Slc41a2	1.710414733	7.295470705	9.823386108	3.15E-09	1.79E-08	10.86093497
Ssh2	1.705797047	8.003402261	18.47983414	2.60E-14	7.57E-13	22.87869691
Izumo4	1.701590413	7.431203413	11.99196359	9.26E-11	7.76E-10	14.49316939
Hlx	1.700889101	8.202142955	12.41734879	4.90E-11	4.44E-10	15.14732074
Gaa	1.699833039	7.621946218	15.03040012	1.39E-12	2.02E-11	18.80966393
Trbj2-7	1.692847416	6.983072538	4.04422575	6.02E-04	1.23E-03	-1.543560675
Mmp25	1.688636353	8.128123082	9.018800598	1.33E-08	6.69E-08	9.373684621
4930487H11Rik	1.686403917	5.403209446	10.59672545	8.44E-10	5.66E-09	12.21649222
Scube3	1.686291045	5.630497436	7.374649925	3.30E-07	1.24E-06	6.074768659
Ms4a6b	1.682854399	8.795839479	7.841990231	1.28E-07	5.21E-07	7.048748069
Ceacam16	1.677340231	5.117770822	6.715853632	1.32E-06	4.47E-06	4.653359198
Tnfsf9	1.673761985	7.050427487	11.36793887	2.43E-10	1.84E-09	13.50057589
Traj21	1.671106402	5.431466581	3.074674729	5.83E-03	9.78E-03	-3.772034623
Il6st	1.668682475	9.836636816	16.17728383	3.41E-13	5.98E-12	20.248048
Gzmc	1.664876356	4.500343425	2.923713215	8.22E-03	1.34E-02	-4.101861328
Dennd3	1.661858064	8.145258638	12.39415882	5.07E-11	4.57E-10	15.11211401
Sulf2	1.661186408	9.139893671	19.46112387	9.44E-15	3.36E-13	23.90850847
Gramd4	1.660692745	7.314102902	15.18668069	1.14E-12	1.67E-11	19.01120539
Rell1	1.65762674	7.604468128	12.04552339	8.54E-11	7.25E-10	14.57651422
Itk	1.648129607	5.372851719	4.683674226	1.32E-04	3.07E-04	-0.027951559
Prdm1	1.64693036	5.676950836	9.73806169	3.65E-09	2.04E-08	10.70701343
Gm5150	1.642551754	5.079444192	4.021796207	6.35E-04	1.28E-03	-1.596451399
Mreg	1.64124325	9.196542901	13.98241925	5.43E-12	6.48E-11	17.40974825
Dapl1	1.635593324	5.257824769	4.93590919	7.32E-05	1.78E-04	0.569705753
Cd14	1.633646199	6.280781495	6.604639892	1.68E-06	5.60E-06	4.407985941
Plac8	1.631714002	9.747400092	7.643610749	1.91E-07	7.50E-07	6.638835608
LOC102634900	1.631242278	6.59448961	9.990576664	2.35E-09	1.38E-08	11.15998711
8430419L09Rik	1.621350895	6.707738718	9.723858661	3.75E-09	2.09E-08	10.68130575
Mgat4a	1.613027335	6.978483768	16.51151839	2.30E-13	4.28E-12	20.65023877
Gne	1.608987548	7.06922744	8.479460309	3.68E-08	1.66E-07	8.330679012
Sucnr1	1.595647746	4.458603885	9.837173864	3.07E-09	1.75E-08	10.88572455
LOC102633435	1.584929371	6.533668971	5.099816086	4.99E-05	1.26E-04	0.956758388
Mterf1b	1.582361392	5.696091824	2.714634457	1.31E-02	2.05E-02	-4.546960822
Defb25	1.581233238	5.96262295	5.276225767	3.31E-05	8.62E-05	1.371677721
Gcnt1	1.580603641	7.104824569	10.46436498	1.05E-09	6.82E-09	11.98943295
Cdcp1	1.579876911	5.864575959	8.142797328	7.05E-08	3.01E-07	7.660354214
Clec7a	1.57835626	7.301194461	13.23220847	1.52E-11	1.59E-10	16.35245602
Gpr155	1.575615603	5.683335985	10.63803119	7.88E-10	5.31E-09	12.28694154
Traj11	1.569629452	4.83081265	3.979788667	7.01E-04	1.41E-03	-1.695413059
Cdc7	1.567160321	6.252031741	7.798352097	1.40E-07	5.63E-07	6.959025924
LOC102636275	1.564847699	5.552270256	5.047617874	5.63E-05	1.40E-04	0.833640129
Fas	1.56061528	7.278762663	7.936315517	1.06E-07	4.37E-07	7.241822619
Acvr2a	1.558774999	7.846643838	19.33669726	1.07E-14	3.69E-13	23.78068093
Tec	1.556069938	8.253682196	12.35641056	5.36E-11	4.79E-10	15.05469429
L3mbtl3	1.555985138	6.502710149	11.11283791	3.64E-10	2.64E-09	13.08303788
Paqr3	1.554601919	6.513223303	12.98417715	2.16E-11	2.13E-10	15.99208848
Cd96	1.551733549	5.296380933	5.801000297	9.96E-06	2.84E-05	2.592166813
Gpr126	1.548619216	7.198298172	8.052261578	8.43E-08	3.54E-07	7.477536597
Ddx60	1.54634915	5.724973348	7.472089635	2.70E-07	1.03E-06	6.280222101

Cd28	1.541295452	6.399334558	4.069693932	5.67E-04	1.16E-03	-1.483464404
Trbj2-3	1.541206858	5.463456974	3.911648699	8.24E-04	1.62E-03	-1.855645617
Tmem63b	1.538183245	7.256600613	7.864769663	1.22E-07	5.00E-07	7.095483382
Tnfrsf1b	1.533968679	9.326362226	11.60423514	1.68E-10	1.31E-09	13.88116965
Nuak1	1.531785591	6.866511441	6.603274454	1.69E-06	5.61E-06	4.404963913
Lck	1.528667028	6.697700307	4.643470055	1.46E-04	3.34E-04	-0.123355758
Cd5	1.527998858	7.515526045	5.935224653	7.36E-06	2.15E-05	2.900330442
Ms4a4b	1.519555441	5.796419814	4.904139066	7.88E-05	1.90E-04	0.494542727
Bcl3	1.519043302	9.160843434	12.30785539	5.76E-11	5.12E-10	14.98063341
Vsig10	1.517572237	6.573835531	8.346893739	4.74E-08	2.10E-07	8.06850949
Cd2	1.516612081	6.563491584	5.360360688	2.73E-05	7.25E-05	1.568844497
Gm3696	1.512992959	6.567447241	4.052769788	5.90E-04	1.20E-03	-1.523404288
Trappc5	1.505990676	7.808930281	8.121743434	7.35E-08	3.13E-07	7.617937183
Eno2	1.50225116	6.883518439	8.448859799	3.90E-08	1.75E-07	8.270366963
Thy1	1.4976788	6.632655824	4.786196785	1.04E-04	2.47E-04	0.215192614
Ctsc	1.49474071	8.773481176	14.09248577	4.69E-12	5.70E-11	17.56088005
Scin	1.493615305	5.666870296	5.801408595	9.95E-06	2.84E-05	2.593106924
Slc4a8	1.493155932	7.911053065	9.184062762	9.85E-09	5.08E-08	9.685786714
Nmrk1	1.493064047	5.991636109	8.723439029	2.31E-08	1.10E-07	8.807169926
Nudt17	1.486469224	8.359305642	8.886256933	1.71E-08	8.41E-08	9.120850834
Panx1	1.481291943	6.901551805	9.359624403	7.17E-09	3.84E-08	10.01355267
Snn	1.481122137	7.548240817	10.56515166	8.90E-10	5.92E-09	12.16251047
Lat	1.472246377	6.989621968	4.010373426	6.52E-04	1.32E-03	-1.623373924
Smad7	1.472075586	7.741140771	9.084414788	1.18E-08	5.98E-08	9.498014904
Snord35b	1.47177392	6.814497495	3.38272791	2.86E-03	5.11E-03	-3.081116612
Bmp2k	1.470906243	10.63906269	18.17415615	3.60E-14	9.35E-13	22.54740737
Mical3	1.467882181	8.373186707	10.78468384	6.19E-10	4.28E-09	12.53550855
Tmem123	1.46781186	10.91072837	16.76216448	1.72E-13	3.46E-12	20.94707839
Gm1966	1.462140763	7.856155264	10.34598311	1.28E-09	8.09E-09	11.78464644
Igha	1.461978075	5.101757487	1.404847657	1.75E-01	2.17E-01	-6.857875611
Gm5797	1.454704308	7.79616844	7.977052661	9.78E-08	4.07E-07	7.324842744
Itgam	1.450978202	5.957914789	7.533927907	2.38E-07	9.16E-07	6.409960493
Pik3r3	1.450894336	6.016700141	5.950841694	7.10E-06	2.09E-05	2.936067015
Traj12	1.450672901	6.188481269	2.387872189	2.66E-02	3.91E-02	-5.209641763
Arl4c	1.446724319	8.834541836	17.0461813	1.25E-13	2.68E-12	21.27863014
Arl5c	1.44407185	8.455719936	13.99736938	5.32E-12	6.37E-11	17.43033461
BC021614	1.443506828	6.386156855	5.893375531	8.08E-06	2.34E-05	2.804443696
Lgals1	1.442894018	9.844921301	10.98009222	4.51E-10	3.23E-09	12.86297894
Sh3kbp1	1.435436847	9.028223686	20.06215781	5.20E-15	2.20E-13	24.51520783
Icosl	1.434495985	10.35968984	21.25233782	1.67E-15	9.24E-14	25.6666628
Enpp5	1.431092914	6.348916038	5.459909772	2.17E-05	5.85E-05	1.801459193
Ift57	1.430806033	8.629745409	12.79984675	2.81E-11	2.69E-10	15.72065739
Ncf2	1.429031343	8.233993957	10.0623176	2.08E-09	1.23E-08	11.28728172
Acp5	1.427456499	6.339838533	6.127760933	4.78E-06	1.47E-05	3.339117942
Bex6	1.419267406	5.087320473	5.841210173	9.09E-06	2.61E-05	2.68467239
Slc44a5	1.419250374	4.237277639	6.4793385	2.21E-06	7.20E-06	4.129724474
Cdk17	1.418457707	8.28047828	11.33794642	2.54E-10	1.90E-09	13.45184703
Ttc3	1.4109235	8.242462236	17.46948555	7.75E-14	1.82E-12	21.76357118
Mir1905	1.40214922	6.183371296	5.176340194	4.17E-05	1.07E-04	1.136979293
Trbj1-2	1.395788152	6.238764658	4.127941769	4.94E-04	1.02E-03	-1.345872053
Mmp23	1.39429397	6.964847608	9.844624271	3.03E-09	1.73E-08	10.89911036
Ly6a	1.393355924	8.838305379	4.160635375	4.57E-04	9.49E-04	-1.268562628
Trbc2	1.392473963	9.557385329	4.799836681	1.01E-04	2.40E-04	0.247521731
Tmcc3	1.39087838	7.039926554	10.07087854	2.05E-09	1.22E-08	11.30243101
Trbj1-4	1.390029388	4.570873134	2.534893297	1.94E-02	2.93E-02	-4.916963207
Gimap3	1.389439799	5.870577466	4.469218868	2.20E-04	4.87E-04	-0.537000155
Ddit4	1.383795924	6.294657343	4.60519267	1.59E-04	3.63E-04	-0.214207134
Hmga2-ps1	1.379291887	7.511288482	7.598090056	2.09E-07	8.14E-07	6.544040705
Fam3c	1.378800548	6.759240613	9.795887204	3.30E-09	1.87E-08	10.81142475
Neto2	1.378701447	5.522303513	9.109475485	1.13E-08	5.74E-08	9.545356813
Nkg7	1.37275912	7.765116745	4.674766693	1.35E-04	3.12E-04	-0.049086918
Cacnb3	1.367420353	8.08297635	9.358093458	7.19E-09	3.84E-08	10.01071121
Abcb1b	1.361678343	5.72263566	4.261337887	3.60E-04	7.62E-04	-1.030132342
Trbj2-1	1.361157936	7.724942096	3.136491439	5.06E-03	8.58E-03	-3.635177443
Trbc1	1.356252699	8.658557022	3.974552461	7.10E-04	1.42E-03	-1.70773935
Slc15a4	1.34875858	8.678473931	10.0298224	2.20E-09	1.29E-08	11.22969942
Ctsh	1.345327549	10.49736472	18.50526891	2.53E-14	7.45E-13	22.90603184
Rbpms	1.343674766	7.747178594	10.97965755	4.51E-10	3.23E-09	12.86225519
Cd300ld	1.340537145	5.882217167	7.356870951	3.43E-07	1.29E-06	6.037146729
Spic	1.338793272	6.848823071	4.711278859	1.24E-04	2.89E-04	0.037538397
Gm3591	1.337728028	5.551607712	2.487780859	2.15E-02	3.21E-02	-5.011782493
Fyn	1.337135165	8.513394063	13.26081007	1.46E-11	1.53E-10	16.39365802
Tspan3	1.336802126	9.552115575	10.97165194	4.57E-10	3.26E-09	12.84892179
Siglece	1.336181623	5.032308888	5.337957256	2.87E-05	7.59E-05	1.51639218
Tnfrsf18	1.330273253	8.740497798	11.15651065	3.39E-10	2.48E-09	13.15501659
Sdhaf1	1.326781654	8.451404962	12.75765008	2.98E-11	2.84E-10	15.65808141

Stk17b	1.326465732	10.30237153	20.26522558	4.26E-15	1.89E-13	24.71626152
Klf4	1.326393576	8.068468362	15.4313476	8.42E-13	1.27E-11	19.32313815
Satb1	1.319406004	6.890103683	8.703243537	2.40E-08	1.14E-07	8.768022487
Chka	1.318449212	8.123180258	11.34326355	2.52E-10	1.89E-09	13.46049277
Art2a-ps	1.316077427	8.487501198	5.810657588	9.74E-06	2.78E-05	2.614398512
Calm4	1.310711223	7.312268148	6.194994319	4.12E-06	1.28E-05	3.491396755
Zfp872	1.308970826	6.597179897	5.290685159	3.20E-05	8.35E-05	1.405598048
Nr4a3	1.293317428	8.756167861	5.683083858	1.30E-05	3.64E-05	2.319997297
Slc25a35	1.292414065	7.576866351	10.28369147	1.43E-09	8.91E-09	11.67623641
Pnpla8	1.291585627	7.78327041	8.844903434	1.84E-08	9.02E-08	9.041504702
Zcchc24	1.291102454	6.169388032	7.791340077	1.42E-07	5.70E-07	6.94458532
Cers6	1.290817653	8.866368711	13.16155452	1.68E-11	1.73E-10	16.25036393
Mef2c	1.290027194	7.304929711	6.860035737	9.71E-07	3.36E-06	4.969184023
P2ry12	1.288933251	6.385255103	5.497437631	1.99E-05	5.41E-05	1.888948388
I830077J02Rik	1.288580477	8.557607012	10.65877524	7.62E-10	5.17E-09	12.32224872
Gm11974	1.284679317	8.689864397	8.744447325	2.22E-08	1.06E-07	8.847836753
Adap1	1.284413849	7.794631984	9.967842265	2.45E-09	1.43E-08	11.11951969
Mfhas1	1.284205782	6.048125989	8.624546023	2.79E-08	1.29E-07	8.614967355
Oasl2	1.283424462	6.184009877	5.945220723	7.19E-06	2.11E-05	2.923207407
Ear2	1.281834865	8.213811162	3.969662856	7.18E-04	1.43E-03	-1.719247814
Spn	1.28044154	6.44509541	5.832307	9.28E-06	2.66E-05	2.664203714
Psd3	1.27956479	5.251119094	7.490470266	2.60E-07	9.97E-07	6.318837851
Foxp4	1.277280439	8.455609935	8.74858191	2.21E-08	1.06E-07	8.855833539
Rap2b	1.27514631	8.062853125	10.21938391	1.59E-09	9.75E-09	11.56384238
Aplp2	1.272157879	8.942225291	16.63061029	2.01E-13	3.84E-12	20.7917815
Tmem150cos	1.271663327	6.161443142	5.050970359	5.59E-05	1.39E-04	0.841551816
Ndst1	1.26860148	6.064562102	7.563128944	2.25E-07	8.66E-07	6.471049614
Irak3	1.267395897	5.777408181	4.576161495	1.71E-04	3.87E-04	-0.283121453
Mir340	1.26362072	7.054949722	4.490707382	2.09E-04	4.64E-04	-0.485988848
H2-Eb2	1.261773794	8.237811341	6.258723404	3.58E-06	1.12E-05	3.63527219
Trbj2-6	1.260231571	4.907188606	2.796922152	1.09E-02	1.74E-02	-4.373537347
Kdm1b	1.259612221	7.899149508	15.20032978	1.12E-12	1.65E-11	19.02872187
Gramd1b	1.259172288	8.16803985	13.88082631	6.23E-12	7.29E-11	17.26936484
Tctex1d2	1.254076304	9.086822178	11.87033088	1.11E-10	9.18E-10	14.30282729
Tmem173	1.250223078	8.723526935	12.85023306	2.61E-11	2.53E-10	15.79516244
Rcn3	1.248627308	6.545644014	6.753134838	1.22E-06	4.15E-06	4.735271314
Smim24	1.247154413	7.752642103	13.12121234	1.78E-11	1.81E-10	16.19187128
Stk38l	1.246470963	6.817065338	5.446761066	2.23E-05	6.02E-05	1.770778761
LOC102638758	1.245318381	6.401467103	6.022683932	6.05E-06	1.81E-05	3.100137091
Foxp1	1.24374405	8.186966706	9.730481216	3.70E-09	2.06E-08	10.69329573
Lpp	1.243015186	7.518762087	9.586759302	4.77E-09	2.63E-08	10.43188412
Ccr7	1.241535226	9.670087648	7.305352172	3.81E-07	1.42E-06	5.927894215
Malt1	1.24084174	9.825356768	14.65322496	2.25E-12	3.08E-11	18.31569972
Tmem120b	1.239715139	7.158514121	8.751244394	2.20E-08	1.06E-07	8.860981929
Prkcb	1.237345715	8.267696816	7.289767845	3.94E-07	1.46E-06	5.894777074
Nceh1	1.237087455	6.857937413	8.803948859	1.99E-08	9.67E-08	8.962706521
Clec4a4	1.233944467	4.597929583	4.428302852	2.42E-04	5.32E-04	-0.634120137
Prnp	1.2338187	7.1176222	4.317319571	3.15E-04	6.76E-04	-0.897427935
Rasal2	1.232784516	7.649890943	6.736748946	1.26E-06	4.28E-06	4.699290473
Trim43c	1.231937403	5.871536228	3.358677002	3.03E-03	5.39E-03	-3.135801428
Ltc4s	1.223620743	6.708749541	6.79337915	1.12E-06	3.83E-06	4.823499253
S1pr1	1.221165616	6.288286262	4.109921613	5.15E-04	1.06E-03	-1.388459719
Rtp4	1.220673674	6.53269222	5.000903773	6.28E-05	1.55E-04	0.7233392
Mfsd7b	1.220403493	7.533378222	10.77664364	6.28E-10	4.33E-09	12.52194356
Zbtb10	1.215287689	8.14872384	8.022721836	8.93E-08	3.73E-07	7.417652546
Olfr107	1.213219177	4.49294905	4.083306927	5.49E-04	1.13E-03	-1.451325683
S100a8	1.212830751	4.32728211	1.005663388	3.26E-01	3.80E-01	-7.327599794
Cdc42ep3	1.211175098	9.148667672	11.51661244	1.92E-10	1.48E-09	13.74072208
Athl1	1.205779694	6.309804941	7.682030774	1.76E-07	6.98E-07	6.718630249
Uap1	1.204808528	9.427953646	12.06232624	8.32E-11	7.11E-10	14.60260234
Aebp2	1.204443276	9.918539978	11.56562706	1.78E-10	1.38E-09	13.81938463
Idi2	1.203881106	6.25986961	5.730777049	1.17E-05	3.29E-05	2.430238492
Spire1	1.200191413	7.447518266	14.60555174	2.39E-12	3.24E-11	18.25249023
Slc25a37	1.197921315	7.71260046	7.029653008	6.78E-07	2.41E-06	5.33735947
Ddx58	1.1972258	6.903449636	5.704525048	1.24E-05	3.48E-05	2.369583989
Trbj1-1	1.193842681	6.039754236	3.684138142	1.41E-03	2.66E-03	-2.387376011
Zbtb18	1.191421003	7.506519081	11.26690295	2.85E-10	2.11E-09	13.33603992
Cd200	1.189975272	7.847900744	8.597805762	2.93E-08	1.35E-07	8.562777847
Rnf149	1.188648692	7.122473728	7.530803368	2.40E-07	9.21E-07	6.403417228
Tespa1	1.185916466	5.985631797	7.093679612	5.93E-07	2.14E-06	5.475379502
S100a6	1.181770165	11.84529172	18.59361858	2.30E-14	6.95E-13	23.00070958
Tspan14	1.181660003	7.184874862	6.379032664	2.75E-06	8.77E-06	3.905619114
Tnfaip3	1.177025502	9.737589431	10.89580926	5.17E-10	3.65E-09	12.7222547
Hspb6	1.174691013	7.692004913	5.772026642	1.06E-05	3.01E-05	2.525413404
Fgr	1.174649454	8.274756724	3.584403318	1.78E-03	3.33E-03	-2.618497375
Bend4	1.174541187	6.887051998	8.245192087	5.78E-08	2.51E-07	7.865812149

Oas2	1.173442138	5.753226051	4.728642974	1.19E-04	2.78E-04	0.078725803
Tbc1d4	1.169560833	7.775308202	8.266680883	5.54E-08	2.41E-07	7.908754217
Jup	1.168432815	7.878903307	7.891519033	1.16E-07	4.75E-07	7.150275752
Relb	1.167003359	9.468017796	9.765877784	3.48E-09	1.96E-08	10.75728971
Hcn3	1.16460704	6.063482685	5.951549228	7.09E-06	2.09E-05	2.937685474
Eif4e3	1.161300611	7.801185562	8.288692411	5.31E-08	2.33E-07	7.952677727
Ctnna1	1.160875358	9.744595207	17.7400437	5.75E-14	1.40E-12	22.0679175
Cd69	1.158866929	6.796739945	7.163141192	5.12E-07	1.87E-06	5.624516801
Dock4	1.158652077	6.860323132	9.458105565	6.00E-09	3.26E-08	10.19572031
Adcy6	1.158288481	8.255273089	5.322904438	2.97E-05	7.82E-05	1.481129228
Eno3	1.157061082	9.223672038	10.03114853	2.20E-09	1.29E-08	11.23205182
Ltb	1.154940966	7.152595761	7.795301621	1.40E-07	5.66E-07	6.952744553
Mefv	1.15423468	6.029786956	4.278540878	3.45E-04	7.36E-04	-0.989362925
Ankrd33b	1.153964668	6.361669232	6.257352135	3.59E-06	1.12E-05	3.632181232
Ccl3	1.153305007	7.065041317	2.96262661	7.52E-03	1.24E-02	-4.017470701
Sesn2	1.147584517	7.213054822	10.1010621	1.95E-09	1.17E-08	11.35577382
Tubgcp5	1.145402753	7.321933227	7.573592961	2.20E-07	8.50E-07	6.492913114
Ly86	1.142167476	9.650274161	15.57398977	7.07E-13	1.10E-11	19.50300782
Rftn1	1.139896257	8.501303902	10.22867557	1.57E-09	9.63E-09	11.5801119
Il17ra	1.135066065	8.688023485	9.946542908	2.54E-09	1.48E-08	11.08155047
Cd164	1.131127134	10.1362609	10.49262916	1.00E-09	6.52E-09	12.03808756
Dstyk	1.128662078	7.029671774	10.55680573	9.02E-10	5.99E-09	12.14822243
Ak2	1.125051022	8.734626125	12.05188	8.45E-11	7.19E-10	14.5863868
D130040H23Rik	1.124549878	6.155933973	4.73924369	1.16E-04	2.73E-04	0.103867334
Ptger4	1.123714942	9.358409981	10.29823819	1.39E-09	8.73E-09	11.70159345
Dusp5	1.114088222	7.654534895	8.222677127	6.04E-08	2.61E-07	7.820754061
LOC102636131	1.109850875	5.690353138	8.166299778	6.74E-08	2.89E-07	7.707635067
Tnfrsf11a	1.108400697	7.473995635	7.731981953	1.60E-07	6.35E-07	6.822081632
Cd63	1.10829585	9.88766756	5.540596705	1.80E-05	4.94E-05	1.989423122
Gadd45b	1.10599109	10.13053105	10.39364059	1.18E-09	7.56E-09	11.86728315
Stard5	1.105085479	6.892578624	4.816017978	9.70E-05	2.32E-04	0.285867461
Gpcpd1	1.104978879	9.404185707	11.4047648	2.29E-10	1.75E-09	13.56027661
Fbln5	1.104584261	5.082152778	3.714793413	1.31E-03	2.49E-03	-2.316070071
Phf21a	1.104114583	8.407863672	9.857718436	2.96E-09	1.70E-08	10.9226198
Clptm1	1.103732274	10.15138602	13.74538179	7.48E-12	8.52E-11	17.08086579
Stat5a	1.099612263	8.003215682	9.190535306	9.74E-09	5.03E-08	9.697939695
Gm15128	1.097647992	6.191734244	1.740589771	9.67E-02	1.27E-01	-6.364870737
Med10	1.09233051	9.464074613	12.60199577	3.74E-11	3.47E-10	15.42581593
Slc38a2	1.091850862	10.18494693	14.53446354	2.62E-12	3.54E-11	18.15790696
Mmp7	1.09082253	6.005220279	4.403109406	2.57E-04	5.60E-04	-0.693910841
Il2rb	1.088516802	6.930556936	4.237856019	3.80E-04	8.02E-04	-1.08576572
Synpo2	1.088106488	6.138728657	6.614666637	1.64E-06	5.50E-06	4.430170453
Gm19345	1.086754392	5.408309561	5.105319287	4.93E-05	1.24E-04	0.969730069
Aff3	1.085840144	7.048278479	8.690189319	2.46E-08	1.16E-07	8.742689679
Phc1	1.085462423	7.35645953	8.256563165	5.65E-08	2.46E-07	7.888543097
Shb	1.083447576	6.652843303	7.164838411	5.11E-07	1.87E-06	5.628152986
Rcsd1	1.08206995	8.986713566	13.23292274	1.52E-11	1.59E-10	16.35348583
Rundc3b	1.079551734	4.953777913	3.46736503	2.35E-03	4.28E-03	-2.887815379
Rac2	1.078034776	10.11202129	5.869528182	8.53E-06	2.46E-05	2.74972412
Slc26a10	1.075498825	6.700996605	8.700285598	2.42E-08	1.14E-07	8.762284297
Ccnd2	1.06810671	7.228677565	4.849274454	8.97E-05	2.16E-04	0.364651742
Pfkfb3	1.067413035	9.784084317	8.900001839	1.66E-08	8.23E-08	9.147174798
Ptpn4	1.067304642	7.289685198	7.611033517	2.04E-07	7.95E-07	6.571022804
B4galnt1	1.065226785	9.902589368	13.46093305	1.11E-11	1.20E-10	16.67993095
9530052E02Rik	1.063196154	5.305700469	3.604336261	1.70E-03	3.18E-03	-2.57241747
Rnd3	1.062348765	9.183053008	8.763880566	2.14E-08	1.03E-07	8.885403714
Pilrb1	1.061126671	4.839470505	3.089300354	5.64E-03	9.47E-03	-3.739744087
Stap2	1.057432729	8.184655574	6.968389244	7.72E-07	2.71E-06	5.204802166
Rhbdf2	1.057076254	6.90086526	8.858284871	1.80E-08	8.82E-08	9.067204171
Pcgf5	1.055354976	8.410066276	7.944407896	1.04E-07	4.33E-07	7.258331968
Mir3109	1.055203301	6.265670991	3.298837096	3.48E-03	6.10E-03	-3.271358669
Ephx1	1.053303552	6.46295913	6.84896115	9.94E-07	3.43E-06	4.945018148
LOC102639543	1.05169256	6.213710032	2.622961571	1.60E-02	2.46E-02	-4.737251046
Lad1	1.05145992	7.675622289	6.205792243	4.02E-06	1.25E-05	3.515806426
Ikzf4	1.042378072	5.968379243	6.523499283	2.00E-06	6.60E-06	4.228010043
Pisd-ps1	1.041829706	6.996663736	2.914823905	8.38E-03	1.36E-02	-4.121075066
Ccser2	1.04143284	9.951697815	11.63898201	1.59E-10	1.26E-09	13.9366432
Rap1a	1.041025577	8.372152589	7.022512432	6.88E-07	2.44E-06	5.321934075
Ccdc88a	1.040273058	10.29341758	16.69699695	1.86E-13	3.63E-12	20.87028747
Spin1	1.038710547	8.764168481	10.73027654	6.77E-10	4.62E-09	12.44357408
Arhgef3	1.038328964	7.881024686	6.717283712	1.32E-06	4.46E-06	4.656504474
Dgkd	1.03711605	7.694239482	7.313690329	3.75E-07	1.40E-06	5.945599984
Extl1	1.034971384	6.200261255	4.203564253	4.13E-04	8.66E-04	-1.166972574
Sidt2	1.0285106	8.444553943	9.912579372	2.69E-09	1.56E-08	11.02089282
Il21r	1.027844914	8.484907492	4.54869827	1.82E-04	4.10E-04	-0.34831803
Fmn1	1.02771241	7.043431058	7.093015622	5.93E-07	2.14E-06	5.473950873

Lgmn	1.025801367	8.240633634	7.872038921	1.20E-07	4.94E-07	7.110382837
Sort1	1.022958666	8.232147634	11.05074135	4.02E-10	2.90E-09	12.98033715
Mir1955	1.021580381	6.475308915	4.444778116	2.33E-04	5.13E-04	-0.595015711
Mrps6	1.020521206	8.864751589	8.261353111	5.60E-08	2.44E-07	7.898113161
Plcl1	1.020446551	5.21305933	6.286544938	3.37E-06	1.06E-05	3.697938012
2510009E07Rik	1.016371896	6.168242168	5.737098664	1.15E-05	3.25E-05	2.444834765
Ifitm3	1.011567241	9.440855115	6.381269466	2.73E-06	8.74E-06	3.910629545
Dclre1c	1.008287038	8.154477817	13.05639081	1.95E-11	1.96E-10	16.09757968
Hist1h2bc	1.007915706	8.712615473	4.37799016	2.73E-04	5.90E-04	-0.753515825
Pglyrp2	1.007171177	6.182148187	5.855903512	8.80E-06	2.53E-05	2.718435681
Gabarapl1	1.004845046	6.683747804	7.862510979	1.23E-07	5.01E-07	7.090852438
Cd244	1.001517115	7.857920437	6.296060605	3.30E-06	1.04E-05	3.719350996
Selm	1.001202748	7.298989355	4.920763725	7.58E-05	1.84E-04	0.533879051
Gpr114	0.99789832	7.747167133	4.19447784	4.22E-04	8.83E-04	-1.188482187
St3gal1	0.995398905	9.559838168	16.17229736	3.43E-13	5.99E-12	20.24199167
Fam129c	0.995144265	6.144756143	6.482824518	2.19E-06	7.16E-06	4.137491547
Trappc2	0.993930723	7.976006058	4.294449827	3.33E-04	7.11E-04	-0.951651919
Peg13	0.993334273	7.102329306	6.912209191	8.69E-07	3.02E-06	5.082823059
Nupr1	0.985936727	6.491913798	4.530154655	1.90E-04	4.27E-04	-0.39234095
H2-DMb2	0.984856715	10.85377278	7.779437476	1.45E-07	5.83E-07	6.920058094
Kynu	0.984803859	9.500901259	14.24769195	3.82E-12	4.86E-11	17.7723153
Atp11a	0.983135167	8.515980279	10.35776289	1.26E-09	7.96E-09	11.80509675
Slamf1	0.982643298	6.195467169	3.403121318	2.73E-03	4.91E-03	-3.03466092
Tubb3	0.98193405	4.862936316	3.494971516	2.20E-03	4.04E-03	-2.824490863
Gins3	0.980864005	7.043052593	4.764386612	1.09E-04	2.59E-04	0.163487863
Cxcr4	0.979269722	8.758485541	9.904126415	2.73E-09	1.58E-08	11.00577459
Ddx26b	0.978896781	6.855381665	4.603288276	1.60E-04	3.64E-04	-0.218727598
Slc44a1	0.976176016	9.094280242	9.281646808	8.25E-09	4.33E-08	9.868450883
Rnf130	0.974325851	9.432565763	11.38166727	2.37E-10	1.81E-09	13.52284862
Trf	0.972771563	8.606495664	5.896608215	8.02E-06	2.32E-05	2.811856925
Dscaml1	0.972743515	5.86052372	6.503340148	2.09E-06	6.87E-06	4.183172266
Tnfrsf4	0.972410918	8.344897055	7.018668333	6.94E-07	2.46E-06	5.313627168
Cdh2	0.969812979	5.960212555	4.429964967	2.41E-04	5.30E-04	-0.630175206
Mir1931	0.969276781	6.203462568	4.849403139	8.96E-05	2.16E-04	0.364956526
Fyb	0.967928105	10.35410345	11.15267563	3.42E-10	2.49E-09	13.14870423
Lamp2	0.966514101	8.787278516	14.62155928	2.34E-12	3.20E-11	18.27373413
Ifi27	0.965024617	8.284228093	8.09578611	7.74E-08	3.27E-07	7.565560527
Birc2	0.962688653	9.322988779	8.883971013	1.71E-08	8.43E-08	9.116470529
Kmo	0.962298668	9.089597823	4.549051307	1.82E-04	4.10E-04	-0.347479917
Dgka	0.962243421	7.738730648	5.775226008	1.06E-05	2.99E-05	2.532788507
Alkbh6	0.96016211	7.731434324	8.549964548	3.21E-08	1.47E-07	8.469172318
Rras2	0.959113511	8.744742223	10.74925159	6.56E-10	4.49E-09	12.4756748
Gm13157	0.958375062	8.059101914	5.765846221	1.08E-05	3.05E-05	2.511163669
Rxra	0.957528817	6.879840849	8.62869205	2.77E-08	1.28E-07	8.62305089
LOC102639885	0.956027453	10.9767459	10.83447405	5.71E-10	3.97E-09	12.619351
Gstk1	0.952071811	6.017326542	4.641709131	1.46E-04	3.35E-04	-0.127534943
Frmd4a	0.950778402	7.765393995	9.445692094	6.14E-09	3.33E-08	10.17282484
Ccl22	0.95010044	10.1259617	6.656749705	1.50E-06	5.04E-06	4.523146432
Gm13179	0.948703304	7.439981213	4.354518117	2.88E-04	6.22E-04	-0.809201717
Atf5	0.947916729	6.745089214	7.109739552	5.73E-07	2.07E-06	5.509916445
Ifi27l2a	0.947763808	8.018114552	6.081617994	5.30E-06	1.61E-05	3.234320015
Tmem150c	0.947131785	7.576876665	3.783732522	1.12E-03	2.14E-03	-2.155299349
Efna2	0.945859024	6.619315659	5.590773136	1.61E-05	4.43E-05	2.106036847
Osbpl8	0.942664612	9.147009753	11.66133546	1.54E-10	1.22E-09	13.97226451
Ric1	0.942418543	8.669532826	8.706657996	2.39E-08	1.13E-07	8.774644878
Thap2	0.941229532	7.301810575	5.52113648	1.88E-05	5.14E-05	1.944138683
4930562F07Rik	0.940163363	8.659146986	6.564113123	1.83E-06	6.08E-06	4.318194302
Milr1	0.938800413	6.356383792	3.317854982	3.33E-03	5.87E-03	-3.22835631
Suox	0.936626737	7.699592444	7.751505968	1.53E-07	6.15E-07	6.862426968
Cc2d2a	0.936600942	5.054512751	6.371264135	2.79E-06	8.90E-06	3.888213091
Lyz1	0.936255119	7.830666154	2.261190046	3.47E-02	5.00E-02	-5.453819944
Arid5b	0.933488807	6.304026727	4.694021735	1.29E-04	3.00E-04	-0.003401206
St5	0.932908493	6.418008934	6.988889887	7.39E-07	2.61E-06	5.249213222
Fcgr2b	0.932583154	6.398415851	4.456117746	2.27E-04	5.01E-04	-0.568099215
Zfp770	0.932429919	8.273622394	7.043536318	6.58E-07	2.36E-06	5.367332074
G6pdx	0.929007073	8.026289978	5.176392075	4.17E-05	1.07E-04	1.137101358
Kdm5b	0.922109017	7.352220996	9.211718113	9.37E-09	4.86E-08	9.737675915
Gcnt4	0.921848267	6.538850641	5.614981327	1.52E-05	4.21E-05	2.162220615
Lta4h	0.91951596	8.071077853	9.410118455	6.54E-09	3.51E-08	10.10710633
Runx1	0.918437249	8.486195312	8.266666258	5.54E-08	2.41E-07	7.908725012
Ccpg1	0.91833796	8.389878553	8.111234471	7.50E-08	3.18E-07	7.596742954
Jak2	0.917766125	10.864644	13.74371475	7.50E-12	8.52E-11	17.07853614
N28178	0.913614399	6.530428932	5.272804165	3.34E-05	8.67E-05	1.363648889
Tmem18	0.907712644	6.747887845	8.744453565	2.22E-08	1.06E-07	8.847848824
Socs2	0.907565953	6.369648136	4.836977894	9.23E-05	2.21E-04	0.335525451
Npl	0.907510112	5.363491215	4.396889414	2.61E-04	5.67E-04	-0.708671116

Fam49a	0.906764401	10.52776753	8.696117334	2.44E-08	1.15E-07	8.754196235
Nfkbiz	0.906072723	8.903886475	8.323536027	4.96E-08	2.19E-07	8.02207679
Mir1896	0.905513921	9.823093256	7.714326579	1.65E-07	6.57E-07	6.785554244
Crebl2	0.904330824	7.987635973	5.947727669	7.15E-06	2.10E-05	2.92894318
Macf1	0.904292884	8.743421785	9.413423201	6.51E-09	3.51E-08	10.11321814
Cyfip1	0.903653944	8.94079814	13.31394086	1.36E-11	1.44E-10	16.47000355
4930523C07Rik	0.900768597	9.101364274	9.933688548	2.60E-09	1.51E-08	11.05860935
Smc6	0.900056892	9.43683015	10.28513632	1.42E-09	8.91E-09	11.67875608
Rnf122	0.895772513	8.523823246	4.806647562	9.91E-05	2.36E-04	0.263662806
Fbrsl1	0.895050138	9.01373268	10.43474224	1.11E-09	7.11E-09	11.93834104
Klri1	0.892532975	6.456371583	3.91482559	8.18E-04	1.61E-03	-1.848183731
Mcu	0.892460832	7.001359463	6.185725939	4.21E-06	1.30E-05	3.470434358
Il13ra1	0.890129755	8.979215541	6.434374603	2.43E-06	7.83E-06	4.029412314
Spred1	0.88834228	8.33953904	6.022106934	6.06E-06	1.81E-05	3.098821531
Tns1	0.887912323	7.251446382	6.269932205	3.49E-06	1.10E-05	3.660529755
Tmem19	0.883431657	8.52873234	8.152046151	6.93E-08	2.96E-07	7.678969172
Inafm2	0.882973209	7.344556703	7.52233376	2.44E-07	9.35E-07	6.385674085
Rnf19a	0.878025521	8.763980654	5.081591958	5.21E-05	1.30E-04	0.913790181
Mir103-2	0.876890163	7.964302501	3.614341307	1.66E-03	3.11E-03	-2.549266582
LOC102632231	0.876208295	7.362839127	6.051712164	5.67E-06	1.71E-05	3.166275969
Usp18	0.876202049	8.865904292	8.039847069	8.64E-08	3.62E-07	7.452383531
Nucb2	0.876145781	8.607871551	6.069484788	5.45E-06	1.65E-05	3.206725218
Scpep1	0.873667301	10.12671836	10.07102431	2.05E-09	1.22E-08	11.30268888
Serpinc1	0.873443089	5.723618465	5.180951464	4.13E-05	1.06E-04	1.147828185
Dnajb9	0.871816072	9.582822841	8.800291864	2.00E-08	9.70E-08	8.955659782
Cdkn2b	0.871315852	6.895543904	3.777081966	1.13E-03	2.17E-03	-2.170832702
Cx3cl1	0.871293201	6.049129719	4.955164967	6.99E-05	1.71E-04	0.615241659
Pot1b	0.870198792	9.292337076	8.690787904	2.46E-08	1.16E-07	8.743851768
1700021K19Rik	0.869774495	7.968984152	9.262924244	8.54E-09	4.46E-08	9.833497902
Hspbap1	0.867767095	6.230607685	4.745952042	1.14E-04	2.69E-04	0.119776093
Pygl	0.861359583	7.853292803	4.873419432	8.47E-05	2.04E-04	0.421827901
Dync1li2	0.859860791	7.617509884	7.462297903	2.76E-07	1.05E-06	6.259632535
Usp17lc	0.858716031	6.91916187	3.210955966	4.26E-03	7.33E-03	-3.469055989
Dtx3	0.85635814	6.792925883	8.708861208	2.38E-08	1.13E-07	8.778917235
Dap	0.855457242	9.857154414	9.410804674	6.54E-09	3.51E-08	10.10837553
Nup62-il4i1	0.855268335	4.893257463	2.810001339	1.06E-02	1.69E-02	-4.34575606
Dkkl1	0.853911417	6.018116691	5.269998074	3.36E-05	8.72E-05	1.35706377
Gm16524	0.853296193	7.133819505	3.143537351	4.98E-03	8.46E-03	-3.61951662
Vps39	0.853176657	8.885415411	8.558866521	3.16E-08	1.44E-07	8.486612451
Gypc	0.850585642	7.021816212	6.091648983	5.18E-06	1.57E-05	3.257121679
Atp2a3	0.848881044	5.865098366	5.90201638	7.93E-06	2.30E-05	2.824256628
S100a9	0.844156705	4.89836198	1.04557649	3.08E-01	3.61E-01	-7.286827411
Fam53b	0.844119787	7.776168672	8.068716502	8.16E-08	3.44E-07	7.510844496
Mfge8	0.843432244	8.008199872	5.484197398	2.05E-05	5.56E-05	1.858094249
Gm19723	0.843208636	6.886787169	5.291101097	3.20E-05	8.35E-05	1.406573587
St3gal4	0.842755715	7.270926763	8.202591247	6.28E-08	2.70E-07	7.780500671
Txnip	0.841916946	9.282525143	7.131190566	5.48E-07	1.99E-06	5.555994883
Hmgn3	0.836843836	8.759306096	9.171144029	1.01E-08	5.19E-08	9.66151437
Clstn1	0.83442637	6.706904916	6.610927408	1.66E-06	5.53E-06	4.421898718
Spr	0.83338488	8.042858019	5.478855638	2.08E-05	5.62E-05	1.845642147
Casp1	0.827310549	8.469603928	9.760744609	3.51E-09	1.97E-08	10.74801883
A630001O12Rik	0.82726063	4.608080994	1.930548982	6.74E-02	9.20E-02	-6.051254972
Olfr109	0.823524255	6.683633351	3.115360307	5.31E-03	8.97E-03	-3.682070481
5430427O19Rik	0.821899389	6.668536759	4.029557389	6.23E-04	1.26E-03	-1.578153757
Sfxn3	0.820667847	7.425071617	4.278949951	3.45E-04	7.35E-04	-0.988393343
Pim2	0.820623287	6.69979141	4.984214597	6.53E-05	1.61E-04	0.68390754
Ptpn6	0.82043513	10.17497432	10.85201044	5.55E-10	3.88E-09	12.64881496
Necap2	0.817521668	9.496200585	8.480566885	3.67E-08	1.66E-07	8.332857716
Ripk2	0.81746408	6.834774958	6.437482815	2.42E-06	7.78E-06	4.036354297
Nrp1	0.816985094	6.127841616	6.035667582	5.87E-06	1.76E-05	3.129730565
Arhgap9	0.815214727	7.574111687	7.423820047	2.99E-07	1.13E-06	6.178600906
Gm15114	0.814978346	4.157550794	1.290264477	2.11E-01	2.57E-01	-7.006407448
4921511I17Rik	0.814939646	7.372573061	5.547314806	1.77E-05	4.87E-05	2.005048929
LOC102632310	0.811378148	6.818439964	4.504497706	2.02E-04	4.51E-04	-0.453251012
Gem	0.807520184	6.753656349	4.230635902	3.87E-04	8.14E-04	-1.10286762
Mylip	0.805913764	8.612815351	8.162812637	6.78E-08	2.91E-07	7.700624455
Cacnb1	0.805378166	6.912137309	6.943212305	8.14E-07	2.85E-06	5.150187287
Gm4793	0.800848835	7.367763296	5.846961764	8.97E-06	2.58E-05	2.697891312
Fhl3	0.800316105	7.019402723	5.475261763	2.09E-05	5.66E-05	1.83726322
Ppdpf	0.797845221	7.209965843	2.939477408	7.93E-03	1.30E-02	-4.067728694
Pot1a	0.79769823	6.960784123	5.639457356	1.44E-05	4.00E-05	2.218973442
Foxh1	0.797177091	5.517276132	6.489934979	2.16E-06	7.05E-06	4.153329617
LOC102640823	0.796770178	6.369809181	2.54203127	1.91E-02	2.89E-02	-4.902515442
Il1b	0.795853824	8.334010182	3.688358981	1.40E-03	2.64E-03	-2.377565162
Ypel5	0.792703795	8.941647997	9.315384277	7.76E-09	4.10E-08	9.931323723
Dusp22	0.79067654	8.71680643	7.448928654	2.84E-07	1.08E-06	6.231499947

Dse	0.790474514	7.34694106	5.904794729	7.88E-06	2.29E-05	2.830625603
Etv3	0.788938068	10.13802526	6.10613699	5.02E-06	1.53E-05	3.290035417
Ptk2	0.788677448	7.040505964	6.457841135	2.31E-06	7.47E-06	4.081794928
Ap3m2	0.787558461	7.337394272	7.388326318	3.21E-07	1.21E-06	6.103680912
Ybx3	0.787187183	9.667879521	9.857544314	2.97E-09	1.70E-08	10.92230731
Kif1b	0.78718671	6.705883306	7.812566745	1.36E-07	5.50E-07	6.988279668
Ehd4	0.786023273	6.895175965	6.110972198	4.97E-06	1.51E-05	3.301014917
Tspan13	0.782492975	10.03267696	8.951554077	1.51E-08	7.53E-08	9.245690169
Nfkb1	0.780154998	9.680437135	12.23814304	6.39E-11	5.64E-10	14.87390116
Csrnp1	0.777777995	8.299377188	6.642004919	1.55E-06	5.20E-06	4.49059485
1700025B11Rik	0.776778181	4.659059061	2.113983944	4.69E-02	6.60E-02	-5.727332695
Ifi30	0.77638875	11.59780226	12.62317108	3.63E-11	3.38E-10	15.45754706
Myb	0.774188419	8.313497545	8.555552488	3.18E-08	1.45E-07	8.480121037
Igsf6	0.772938254	9.005572965	7.312991112	3.75E-07	1.40E-06	5.944115572
Dhx57	0.771411411	8.625237736	7.723583165	1.62E-07	6.46E-07	6.804710442
Ikbkb	0.767895496	9.268071713	9.657211121	4.21E-09	2.34E-08	10.56034346
Avpi1	0.767327221	8.515324833	5.931160197	7.42E-06	2.16E-05	2.891025605
Trim24	0.766844513	8.774150411	9.067974581	1.22E-08	6.15E-08	9.466914433
Gba	0.764604475	9.804786213	10.50511828	9.83E-10	6.43E-09	12.05955739
Rsc1a1	0.764250181	7.047501388	2.784896625	1.12E-02	1.78E-02	-4.399029167
Rnf152	0.761038753	4.293565757	2.706740845	1.33E-02	2.08E-02	-4.563469699
0610040B10Rik	0.760858387	6.627295527	2.120515904	4.63E-02	6.52E-02	-5.715442589
5830416P10Rik	0.758673756	6.391629739	3.925888228	7.97E-04	1.57E-03	-1.822192961
Ctsb	0.757743797	10.26553072	8.894394376	1.68E-08	8.31E-08	9.136438436
Ttyh3	0.757118498	9.764569633	11.3715898	2.41E-10	1.83E-09	13.50650106
Wbp5	0.756586436	7.829932972	4.622259975	1.53E-04	3.49E-04	-0.173695945
Ccdc82	0.755500404	8.139431998	7.209767546	4.65E-07	1.71E-06	5.724274743
Mir5129	0.752479041	7.343505616	3.564757186	1.87E-03	3.47E-03	-2.663856461
H2-Q7	0.75178206	9.882887511	6.159987451	4.45E-06	1.37E-05	3.41217117
Car13	0.751375072	5.76917305	4.272458224	3.50E-04	7.45E-04	-1.003779327
Foxn3	0.749054171	7.666315883	7.116998916	5.64E-07	2.04E-06	5.525516786
Acp2	0.748972955	8.104161769	4.985544094	6.51E-05	1.61E-04	0.687049227
LOC102637244	0.748576494	5.052387715	1.346031276	1.93E-01	2.37E-01	-6.935440406
Stard7	0.746202413	9.097827599	8.09658375	7.72E-08	3.27E-07	7.567171332
Rassf2	0.743249738	8.984512911	6.449058341	2.36E-06	7.59E-06	4.062197392
Syk	0.742857133	7.183152812	6.711447906	1.33E-06	4.50E-06	4.64366778
Insr	0.742023822	7.534838404	9.013179414	1.35E-08	6.75E-08	9.363007648
Rgp1	0.739065278	8.063027561	7.640814257	1.92E-07	7.54E-07	6.63301994
Pet100	0.73896002	8.335572868	7.482814328	2.65E-07	1.01E-06	6.302758952
Insl6	0.738726031	7.184780897	3.540019249	1.98E-03	3.66E-03	-2.720887544
Atl3	0.738301459	8.54002135	8.735303214	2.26E-08	1.08E-07	8.83014307
6330416G13Rik	0.737672345	6.0908781	4.950278281	7.07E-05	1.72E-04	0.603687155
Frrs1	0.737513155	6.398355441	5.315895182	3.02E-05	7.93E-05	1.464703734
Etnk1	0.736630684	9.066965994	8.454619282	3.86E-08	1.74E-07	8.281727993
Zc2hc1a	0.735911847	7.335114838	7.483365614	2.64E-07	1.01E-06	6.303917016
Slc12a6	0.733431408	7.392005267	6.787470891	1.13E-06	3.87E-06	4.810559216
Ptk2b	0.733120569	8.310747496	7.018827177	6.94E-07	2.46E-06	5.31397046
Sash3	0.730642159	7.806577869	5.100168035	4.98E-05	1.26E-04	0.957588022
Zbed4	0.730477946	5.48811668	2.148675241	4.37E-02	6.19E-02	-5.663916185
Trp53inp2	0.724329662	6.979871888	4.50269882	2.03E-04	4.52E-04	-0.457521554
Mt2	0.723547568	7.279654706	2.736418338	1.25E-02	1.96E-02	-4.501284192
Gal3st2	0.720846885	7.93288929	3.930490588	7.88E-04	1.56E-03	-1.811377004
Slc25a29	0.719526861	6.235858642	5.049756321	5.61E-05	1.39E-04	0.838686816
Myo5a	0.71915578	8.94734547	9.536798812	5.22E-09	2.86E-08	10.34041729
Pigx	0.718222184	6.637496378	4.611300027	1.57E-04	3.58E-04	-0.199710309
Ankrd44	0.716462646	8.871671964	10.76570904	6.39E-10	4.38E-09	12.50348367
Gm3099	0.714305715	2.317110868	1.364018298	1.87E-01	2.31E-01	-6.91201308
Lrrc8c	0.712766933	9.874346184	7.333601522	3.60E-07	1.35E-06	5.987843841
Gucd1	0.711041946	7.264454341	6.082272355	5.29E-06	1.60E-05	3.235807788
Rgs1	0.710642027	10.50565477	5.633001976	1.46E-05	4.05E-05	2.204010457
Cdkn1a	0.710291793	10.55864255	5.721578835	1.19E-05	3.36E-05	2.408993603
Slc12a4	0.709702327	6.250071249	3.743729235	1.23E-03	2.34E-03	-2.248657452
Plxnd1	0.708643532	8.406928415	6.632932703	1.58E-06	5.30E-06	4.470553148
Ogfrl1	0.707794707	9.673878147	6.98397636	7.47E-07	2.63E-06	5.238573823
Map1lc3b	0.706313475	9.645468599	6.352840523	2.91E-06	9.26E-06	3.846905334
Cep63	0.705490216	8.581992374	11.19282691	3.20E-10	2.35E-09	13.21471358
Slc14a1	0.704233301	6.685360748	2.732201772	1.26E-02	1.98E-02	-4.510138875
Ldlr	0.703380281	7.415915726	6.152325308	4.53E-06	1.40E-05	3.394812491
Rel	0.703100055	10.67593003	6.326360946	3.08E-06	9.78E-06	3.787466133
Glipr2	0.70246867	7.568716101	5.260311073	3.44E-05	8.90E-05	1.334326986
Pram1	0.702151203	6.241623834	3.319648738	3.31E-03	5.85E-03	-3.224296474
Cd38	0.701640112	7.193817602	5.725704881	1.18E-05	3.33E-05	2.418524413
Nck1	0.700094985	7.421964569	7.624565149	1.98E-07	7.76E-07	6.599207316
Dusp6	0.699245647	7.301248296	3.928982493	7.91E-04	1.56E-03	-1.814921359
Stk4	0.698653264	10.33204959	11.6356089	1.60E-10	1.26E-09	13.93126349
Rgs18	0.698199583	7.008813238	3.212841892	4.24E-03	7.30E-03	-3.464831613

Swap70	0.695883365	9.313710833	6.46802216	2.26E-06	7.34E-06	4.104500939
Nlrc4	0.693173301	5.118041203	4.142588769	4.77E-04	9.88E-04	-1.311243522
Cdc42se2	0.691029606	9.24327325	8.57898214	3.04E-08	1.40E-07	8.525983451
Gca	0.690702958	8.433500794	4.510127427	2.00E-04	4.45E-04	-0.439886043
Il6ra	0.689555734	8.469521269	4.282935881	3.42E-04	7.29E-04	-0.978945664
Plin3	0.688139018	7.467296587	7.268782488	4.11E-07	1.52E-06	5.850132415
Pam	0.687953333	6.134043157	4.426332935	2.43E-04	5.34E-04	-0.638795571
Ube2d1	0.686899375	9.52977387	8.97280545	1.45E-08	7.26E-08	9.286201829
Galnt12	0.686849093	7.791512267	4.743762283	1.15E-04	2.70E-04	0.114583226
Nrip1	0.686313961	7.822943957	7.942912396	1.05E-07	4.33E-07	7.255281635
Kmt2e	0.684671714	9.816620887	10.25450982	1.50E-09	9.27E-09	11.62529399
Rab9	0.682229422	9.038661827	5.111672448	4.85E-05	1.23E-04	0.984703115
Cpeb4	0.681713613	8.502568545	8.331591311	4.89E-08	2.16E-07	8.038097972
Tank	0.680575988	9.255562733	5.735019648	1.16E-05	3.26E-05	2.440034842
Slc25a10	0.680084248	7.544453933	4.098080043	5.30E-04	1.09E-03	-1.41643549
Fthl17	0.677949196	2.795109414	0.803850107	4.31E-01	4.85E-01	-7.511364859
Gm6644	0.677068911	5.266271756	1.467001323	1.57E-01	1.98E-01	-6.772947807
LOC102637286	0.676898547	6.206674963	1.687081514	1.07E-01	1.39E-01	-6.448890609
Ighm	0.676625983	9.791587563	5.958527542	6.98E-06	2.06E-05	2.953645342
Wbscr27	0.673876718	5.642099939	3.608553338	1.69E-03	3.15E-03	-2.56266124
Itgb8	0.670331675	6.234621951	2.974382016	7.33E-03	1.21E-02	-3.991888451
Rab26	0.667876428	5.443616445	2.924081187	8.21E-03	1.34E-02	-4.101065457
Mapk3	0.664771829	9.212088447	9.313957794	7.78E-09	4.10E-08	9.92866824
Hivep1	0.664534101	9.831987263	5.497873849	1.99E-05	5.41E-05	1.889964677
Nfam1	0.66211007	7.63427417	3.877002406	8.95E-04	1.74E-03	-1.936964211
Prps2	0.660906595	7.390724665	6.969072132	7.71E-07	2.71E-06	5.206282388
Map3k3	0.659388921	8.453544492	7.306085359	3.81E-07	1.42E-06	5.929451475
Pirb	0.658018828	9.8244488	6.526893315	1.99E-06	6.55E-06	4.235554216
Acsf2	0.656696648	6.209571941	2.602245326	1.68E-02	2.56E-02	-4.779805345
E130311K13Rik	0.654854489	6.670934166	4.192141666	4.24E-04	8.87E-04	-1.194011869
Plxdc1	0.653562119	8.446097955	6.130991415	4.75E-06	1.46E-05	3.346446169
Siglecg	0.650569537	7.938280242	3.254381001	3.86E-03	6.70E-03	-3.371580537
Siah1a	0.650513213	8.668039544	5.314500405	3.03E-05	7.95E-05	1.461434799
Fth1	0.650140627	11.66780373	11.27507824	2.81E-10	2.09E-09	13.34939383
Ptrf	0.650047017	5.51229149	2.298358992	3.21E-02	4.66E-02	-5.382987829
Stoml1	0.649390498	7.596365082	6.837299146	1.02E-06	3.51E-06	4.919553742
Rpph1	0.648079037	8.555402965	2.503157561	2.08E-02	3.12E-02	-4.980939091
Trp53cor1	0.647257253	5.600240797	3.388215639	2.83E-03	5.06E-03	-3.068623456
Gzma	0.645225525	7.834905376	2.754397137	1.20E-02	1.89E-02	-4.463457996
Rasa2	0.644383789	8.180808887	4.873380495	8.47E-05	2.04E-04	0.421735715
Igkv4-55	0.643882746	3.021014785	0.805900938	4.29E-01	4.84E-01	-7.509689482
Nostrin	0.642557998	8.45801214	4.822727153	9.54E-05	2.28E-04	0.301764258
Atp6ap2	0.642526031	8.144918206	7.291456497	3.92E-07	1.46E-06	5.898367042
Mir1927	0.642122242	7.003013483	3.54626929	1.95E-03	3.61E-03	-2.706487628
Slc22a23	0.641982036	7.456132298	3.645581373	1.55E-03	2.90E-03	-2.476888561
Fam21	0.640263518	8.752665179	6.298013604	3.28E-06	1.04E-05	3.723744521
Tnnt2	0.64019423	7.155603206	2.614918614	1.63E-02	2.50E-02	-4.753792593
Dip2b	0.639549558	8.799551663	7.574774126	2.19E-07	8.49E-07	6.495380132
Cfp	0.639033764	7.736438303	4.653305629	1.42E-04	3.27E-04	-0.100013823
Cyb5r3	0.638485325	9.572005454	7.704793441	1.68E-07	6.69E-07	6.765813864
LOC102638331	0.635281484	8.560343293	3.039508155	6.32E-03	1.06E-02	-3.849441533
Rb1	0.634719105	9.24554471	7.635297066	1.94E-07	7.60E-07	6.621543184
Gm5779	0.633464292	4.959433166	1.392427519	1.79E-01	2.21E-01	-6.874484077
D330050G23Rik	0.632302986	5.737210234	2.675416837	1.43E-02	2.21E-02	-4.628754831
Donson	0.629880137	7.942985683	6.085915626	5.25E-06	1.59E-05	3.244090391
4632427E13Rik	0.6271943	9.105084465	6.790676057	1.13E-06	3.85E-06	4.817579587
LOC102631791	0.626325028	7.471903435	3.427906854	2.58E-03	4.66E-03	-2.978095496
Tyrobp	0.626125903	11.55008268	11.35182327	2.49E-10	1.87E-09	13.47440469
Mir1970	0.62598314	6.818910175	2.167266529	4.21E-02	5.97E-02	-5.629661615
Ipo9	0.624878477	7.331686966	4.270041064	3.52E-04	7.48E-04	-1.009507869
Ubl3	0.624475595	10.34155105	7.668314513	1.81E-07	7.16E-07	6.690165347
Adrbk2	0.622640603	9.214031684	8.437766233	3.98E-08	1.78E-07	8.248471768
H2-K2	0.622163199	7.851857606	4.361190391	2.84E-04	6.12E-04	-0.793373311
Mex3b	0.621183842	7.265464399	4.085256843	5.46E-04	1.12E-03	-1.446721235
Fcrl1	0.618917941	6.812729885	4.636166942	1.48E-04	3.39E-04	-0.140688428
Syngr2	0.618460897	11.82644779	9.213063993	9.35E-09	4.86E-08	9.740198695
Dock9	0.617548355	5.981866566	4.530363764	1.90E-04	4.27E-04	-0.39184452
Hcst	0.616763335	8.750234596	5.918011915	7.65E-06	2.23E-05	2.860913398
Elf1	0.614972922	9.826682739	9.051547787	1.26E-08	6.32E-08	9.43580492
Frmd5	0.614715123	6.163515456	2.837356887	9.98E-03	1.60E-02	-4.287464984
Ago1	0.613701909	7.640684123	4.523863899	1.93E-04	4.32E-04	-0.407275386
Rnf181	0.613375347	8.38733694	5.993478865	6.46E-06	1.92E-05	3.033505127
Chd3	0.612043118	8.084725642	5.795216525	1.01E-05	2.87E-05	2.57884786
Zmat1	0.610985866	7.02098841	3.096062664	5.55E-03	9.35E-03	-3.724795238
Chd9	0.606172692	8.120364412	7.306631915	3.80E-07	1.42E-06	5.930612292
Gramd1a	0.604848304	8.32618794	6.725245778	1.30E-06	4.39E-06	4.674011365

Jmjd1c	0.603624484	9.979501754	5.675947096	1.32E-05	3.69E-05	2.303482854
Rptor	0.602675713	7.955869598	9.11363834	1.12E-08	5.71E-08	9.553213082
Arl8b	0.602278177	8.837600109	8.43755478	3.98E-08	1.78E-07	8.248054268
Ndnl2	0.600507458	8.212723575	4.967752024	6.79E-05	1.67E-04	0.644998828
Prex1	0.599938154	9.393838827	8.801589271	2.00E-08	9.69E-08	8.95815998
Enox2	0.598570499	8.131282477	3.94352374	7.64E-04	1.52E-03	-1.780738261
Csnk1g3	0.598107351	8.296013865	6.611083331	1.66E-06	5.53E-06	4.422243679
Snx9	0.598022029	8.26912787	6.934229819	8.30E-07	2.89E-06	5.130682536
LOC100862024	0.597484865	9.103021944	4.561423832	1.77E-04	3.99E-04	-0.318107702
Zfp809	0.597482285	8.36910149	7.942328276	1.05E-07	4.33E-07	7.254090142
Zfp296	0.597452181	7.877308481	4.414077435	2.50E-04	5.48E-04	-0.66788188
Ikzf1	0.597332462	9.001966213	6.172408928	4.33E-06	1.34E-05	3.440298361
Gm5129	0.596544658	8.187087168	4.383330375	2.69E-04	5.83E-04	-0.740845033
Csf2rb2	0.596167914	9.042320093	5.186628749	4.08E-05	1.04E-04	1.161183297
D730005E14Rik	0.595966917	7.485929021	5.049806256	5.61E-05	1.39E-04	0.838804659
Ptger3	0.594318986	4.454484279	3.310925427	3.38E-03	5.96E-03	-3.244033825
Tpm1	0.592776805	7.778102818	6.696246614	1.38E-06	4.65E-06	4.610210541
Gm4651	0.590811784	6.078918018	2.850572905	9.69E-03	1.56E-02	-4.259214767
Lyst	0.590550739	9.716795656	6.529704894	1.98E-06	6.52E-06	4.241802675
Tlr9	0.589144928	9.377493913	5.576970815	1.66E-05	4.57E-05	2.07398077
Abcb1a	0.587638405	5.659006867	2.608951681	1.65E-02	2.53E-02	-4.766048031
Nfkbie	0.58629989	9.770662543	6.800243532	1.10E-06	3.78E-06	4.838527858
Ctsz	0.585220738	10.31717075	7.308741436	3.78E-07	1.41E-06	5.935092291
Dstn	0.584476084	9.509330842	6.922857149	8.50E-07	2.96E-06	5.105972957
Mink1	0.582499939	9.193692299	7.030337508	6.77E-07	2.41E-06	5.338837814
Spsb1	0.581270526	7.518544567	2.679421069	1.42E-02	2.20E-02	-4.620429625
Calhm2	0.577574631	8.047813332	5.403305762	2.47E-05	6.61E-05	1.669286468
Pias3	0.576544534	8.031485068	5.164015701	4.30E-05	1.09E-04	1.107977236
BC037034	0.576163179	7.008524925	5.328291553	2.94E-05	7.74E-05	1.493751025
Ghitm	0.57507783	9.570537292	8.230198542	5.95E-08	2.58E-07	7.835813744
Tgm2	0.575019779	6.136551958	3.184625973	4.53E-03	7.77E-03	-3.52794703
Arl6ip5	0.574931476	8.633088863	5.944576932	7.20E-06	2.11E-05	2.92173434
Fam13b	0.574854202	8.972103211	7.952128632	1.03E-07	4.26E-07	7.274075029
Arhgef6	0.574480163	8.83645183	5.421916708	2.37E-05	6.35E-05	1.71277128
Nupr1l	0.573878298	6.011958775	1.767064759	9.20E-02	1.22E-01	-6.322577519
Gyg	0.573496372	9.883210275	5.494992521	2.00E-05	5.43E-05	1.883251545
Gm15232	0.572560219	6.719535346	2.458923892	2.29E-02	3.40E-02	-5.069388248
Traf6	0.571913948	8.470601645	7.739601267	1.57E-07	6.28E-07	6.837832548
Slc9a1	0.570880525	8.715490911	6.835445628	1.02E-06	3.52E-06	4.915504939
Pgap2	0.570012069	8.369613482	5.379147037	2.61E-05	6.96E-05	1.612799871
Gm8995	0.569595368	8.100752162	4.245294526	3.74E-04	7.89E-04	-1.068144501
Zmiz2	0.568119432	10.18082655	8.931655923	1.57E-08	7.79E-08	9.207705626
Idh1	0.567592473	9.001287755	5.134127286	4.61E-05	1.17E-04	1.037606148
Galnt1	0.565290981	9.408780915	7.111055704	5.71E-07	2.07E-06	5.512745356
Hist3h2ba	0.565103619	6.657671302	2.132867149	4.51E-02	6.36E-02	-5.692895455
Ptpn12	0.564600255	8.38912839	6.60365813	1.68E-06	5.61E-06	4.405813099
Zc3h7b	0.562921415	7.564624537	4.658090375	1.41E-04	3.23E-04	-0.088659072
Gpr68	0.560752271	9.040171229	4.517088775	1.96E-04	4.38E-04	-0.423359697
Prdx5	0.56072307	9.172701971	3.988174174	6.88E-04	1.38E-03	-1.675668804
Ddhd1	0.559476356	10.16701978	6.424818058	2.48E-06	7.98E-06	4.008061252
E030011O05Rik	0.55874201	6.394928518	2.647426908	1.52E-02	2.34E-02	-4.686779401
Slfn8	0.558505402	7.442815307	2.020444535	5.65E-02	7.82E-02	-5.894967151
Katnbl1	0.558405745	7.695041159	5.03842089	5.76E-05	1.43E-04	0.811932805
Rnf13	0.558054668	8.863120403	6.455123251	2.33E-06	7.50E-06	4.075731342
Fah	0.557511013	5.988454985	2.754280223	1.20E-02	1.89E-02	-4.463704345
Spns3	0.557395273	7.253340514	3.374223377	2.92E-03	5.21E-03	-3.100466293
BC005537	0.557208393	10.4121897	9.394148056	6.73E-09	3.61E-08	10.07755137
Eef1a1	0.556237803	4.28235963	0.988544859	3.34E-01	3.89E-01	-7.344646318
Stxbp1	0.555591981	6.556577383	3.570990949	1.84E-03	3.43E-03	-2.649470227
Rab21	0.554986945	10.29103406	8.268727704	5.52E-08	2.41E-07	7.912841298
Olfr43	0.554378533	3.864142542	0.839878294	4.11E-01	4.67E-01	-7.481350749
Loxl3	0.554223173	6.642279417	3.794151248	1.09E-03	2.10E-03	-2.130955095
Plxnc1	0.553985578	11.14016603	5.869194209	8.54E-06	2.46E-05	2.748957387
Trps1	0.553421869	8.058831658	6.263666817	3.54E-06	1.11E-05	3.646413287
Kcnk6	0.548114802	8.720180906	5.093224193	5.07E-05	1.27E-04	0.941218341
1700029I15Rik	0.547213553	6.875118788	2.520500805	2.00E-02	3.01E-02	-4.946029728
Rab24	0.545292307	9.128452122	6.284365587	3.38E-06	1.07E-05	3.693032386
Olfr108	0.545052628	5.283151633	2.059916757	5.22E-02	7.28E-02	-5.824835886
Nup62	0.545005728	10.83653911	7.328174534	3.64E-07	1.36E-06	5.97633502
Ctss	0.542813238	11.37669948	10.551701	9.10E-10	6.02E-09	12.13947934
Gm9994	0.542430451	6.48563562	2.701658265	1.35E-02	2.10E-02	-4.57408744
Ggt5	0.541908271	4.793938291	2.839784007	9.92E-03	1.59E-02	-4.282281128
Trim12a	0.541061461	7.676931557	2.611422425	1.64E-02	2.52E-02	-4.760975093
Slc2a1	0.540935304	7.678969899	4.17772776	4.39E-04	9.14E-04	-1.228123978
Stap1	0.540260101	8.100793034	3.991782696	6.82E-04	1.37E-03	-1.667170662
D430020J02Rik	0.53849742	6.267967899	2.808173128	1.07E-02	1.70E-02	-4.349642812

Mrpl14	0.536344338	8.028208314	3.681593377	1.42E-03	2.68E-03	-2.393289911
Smad1	0.535039439	8.289304486	5.09846035	5.00E-05	1.26E-04	0.953562505
Rnf115	0.534718136	10.36184594	5.885210786	8.23E-06	2.38E-05	2.785715503
Slc8a1	0.533813175	6.144768764	2.209848651	3.86E-02	5.52E-02	-5.550509478
Snx11	0.532736585	7.075745421	4.9694717	6.76E-05	1.66E-04	0.649063791
Cflar	0.53260814	9.028725244	4.387759161	2.67E-04	5.78E-04	-0.730336418
Emc3	0.532091884	8.90399068	7.8087675	1.37E-07	5.53E-07	6.980463446
Prkcd	0.530861028	10.48809687	8.144257395	7.03E-08	3.00E-07	7.66329362
Ighv1-75	0.5289682	6.513786725	1.873332885	7.53E-02	1.01E-01	-6.148156268
Limd2	0.528053864	8.929954048	5.155348388	4.38E-05	1.11E-04	1.087575814
Rabgap1l	0.527494159	8.209510415	5.145405296	4.49E-05	1.14E-04	1.064165936
Lyrm4	0.524738564	7.306610585	3.660595356	1.49E-03	2.81E-03	-2.442055923
B630005N14Rik	0.521977411	9.858335367	5.979738017	6.66E-06	1.97E-05	3.002124067
Atp6v0a1	0.521452726	8.312829673	4.524810268	1.93E-04	4.32E-04	-0.405028679
Zfp36l1	0.52138441	11.69348317	7.189737547	4.85E-07	1.77E-06	5.681454816
Dnajc25	0.519970946	6.764892148	3.217450105	4.20E-03	7.24E-03	-3.454506
Abhd2	0.519297346	8.378873172	6.58212494	1.76E-06	5.85E-06	4.358126175
Jak1	0.518600456	10.09292527	7.017636643	6.95E-07	2.46E-06	5.311397413
Cib3	0.518103546	4.915802268	1.827577756	8.21E-02	1.10E-01	-6.224156842
Sgk3	0.51778365	8.801436768	6.198019971	4.09E-06	1.27E-05	3.498237828
Slc27a3	0.516905301	5.757569312	2.249700015	3.55E-02	5.11E-02	-5.475575899
Mxd1	0.515363969	9.559405354	4.475561111	2.17E-04	4.81E-04	-0.52194467
Impact	0.514777579	8.535549517	4.603396421	1.60E-04	3.64E-04	-0.218470894
Uhrf1bp1l	0.514737537	7.864060128	5.375802055	2.63E-05	7.01E-05	1.604975362
Pcbp4	0.514623045	5.101903567	2.210590374	3.85E-02	5.51E-02	-5.549122299
Gtf2a1	0.512587653	10.38547307	6.010310519	6.22E-06	1.86E-05	3.071917885
Nfe2l1	0.512210633	9.02767145	5.992377594	6.47E-06	1.92E-05	3.030990797
Akap2	0.509411522	8.127523956	2.373491139	2.74E-02	4.02E-02	-5.237747991
Mir142	0.508993852	8.312883842	2.652397832	1.50E-02	2.32E-02	-4.676496209
Tuba1a	0.508894603	9.858660891	4.169402336	4.47E-04	9.32E-04	-1.247822709
Gga2	0.507886193	6.16009029	3.516826471	2.09E-03	3.85E-03	-2.774268784
Snord89	0.506227733	7.016434335	1.54772983	1.37E-01	1.74E-01	-6.658218524
Ly9	0.504397211	7.990207099	4.57020966	1.73E-04	3.92E-04	-0.297250598
Sh3bp5	0.504255757	6.566644392	3.146077121	4.95E-03	8.42E-03	-3.613868492
Fam110a	0.502739025	7.721265124	4.053950592	5.88E-04	1.20E-03	-1.52061826
Mycbp2	0.502446848	9.347973155	7.581066973	2.16E-07	8.39E-07	6.508520446
Adpgk	0.502434161	6.87857665	3.389967886	2.81E-03	5.04E-03	-3.064633147
Nod1	0.502265728	7.350378131	3.754201434	1.20E-03	2.28E-03	-2.224235553
Rfwd2	0.50179103	10.60466722	5.193801882	4.01E-05	1.03E-04	1.178054365
Ccnd1	0.501780715	9.549727089	5.539846747	1.80E-05	4.95E-05	1.987678539
Rdm1	0.501616067	6.744513989	3.952939624	7.48E-04	1.49E-03	-1.758594379
Hgd	0.499127699	5.382994489	3.209534683	4.28E-03	7.35E-03	-3.472239045
Sdf4	0.497360465	8.28547558	6.113818417	4.93E-06	1.51E-05	3.307476748
Hfe	0.495838491	6.889314049	2.367140628	2.78E-02	4.07E-02	-5.250128589
Pepd	0.494482716	7.951314573	3.709116695	1.33E-03	2.52E-03	-2.329283336
Htra2	0.493538129	10.20388944	5.645519489	1.42E-05	3.95E-05	2.233021522
Myo1h	0.492826892	6.324608214	2.820925466	1.04E-02	1.65E-02	-4.322508097
AW011738	0.491169307	5.450191371	1.635070642	1.17E-01	1.51E-01	-6.528648791
Adam23	0.490616953	10.78064334	4.906899057	7.83E-05	1.89E-04	0.50107401
Renbp	0.490447588	6.636110869	3.566040467	1.86E-03	3.46E-03	-2.660895398
2900052N01Rik	0.490147063	5.16344675	1.583090715	1.29E-01	1.65E-01	-6.606430741
Gbp10	0.488579644	7.141067292	2.733364378	1.26E-02	1.98E-02	-4.507698069
Smurf1	0.48847041	7.884457162	4.847348147	9.01E-05	2.16E-04	0.360089319
Ssh1	0.488183597	7.864040393	4.750653078	1.13E-04	2.67E-04	0.130923903
Cebpg	0.487535406	8.705414741	5.250962411	3.51E-05	9.07E-05	1.312378386
Mob3b	0.487227235	7.439309083	3.093756718	5.58E-03	9.39E-03	-3.729894132
Shisa5	0.486241264	7.918526875	4.311323826	3.20E-04	6.85E-04	-0.911645198
Pus10	0.482219523	8.177980789	4.768130797	1.09E-04	2.57E-04	0.172364981
Prr14	0.481848046	9.106605547	5.690491721	1.28E-05	3.58E-05	2.33713413
Tmem168	0.481830195	8.538883319	7.484484995	2.64E-07	1.01E-06	6.306268325
Degs1	0.481803465	9.218194156	4.700770967	1.27E-04	2.95E-04	0.012610881
Net1	0.481010117	9.997706294	4.537939433	1.87E-04	4.20E-04	-0.373859678
Arhgdib	0.480668002	10.57902611	7.447138267	2.85E-07	1.08E-06	6.227730692
Elovl5	0.47932527	9.66784009	5.083451003	5.18E-05	1.30E-04	0.918174197
Zbtb20	0.47857894	7.557193019	5.16895867	4.25E-05	1.08E-04	1.119610155
Ccl4	0.477798476	7.587501416	1.823731115	8.27E-02	1.10E-01	-6.230484723
S100a10	0.477702987	10.22979102	3.777657415	1.13E-03	2.17E-03	-2.169488851
Zfc3h1	0.477600909	9.842032054	5.073812208	5.30E-05	1.32E-04	0.895441912
Ech1	0.476960856	8.470227631	3.216689345	4.21E-03	7.25E-03	-3.456210969
Rexo2	0.476573368	8.982996381	4.634225223	1.49E-04	3.40E-04	-0.14529687
Igsf8	0.476029972	6.589246261	3.353439335	3.06E-03	5.45E-03	-3.147695365
Tead2	0.474767741	5.294625757	3.04174768	6.29E-03	1.05E-02	-3.844522009
Myo1b	0.473757575	4.808375465	2.601471521	1.68E-02	2.56E-02	-4.781391567
Klra13-ps	0.472025508	6.703620488	1.18316049	2.50E-01	3.00E-01	-7.135475447
Tbc1d1	0.471399714	8.531929633	6.010039354	6.22E-06	1.86E-05	3.071299277
Cln5	0.471094058	8.422977047	7.144960684	5.32E-07	1.94E-06	5.585542765

Cd83	0.470974299	9.309094156	4.426242527	2.43E-04	5.34E-04	-0.639010146
Fam32a	0.470433354	9.656072671	4.634610347	1.49E-04	3.40E-04	-0.14438282
Crebrf	0.469851085	8.18442127	4.706547477	1.25E-04	2.92E-04	0.026314565
Pik3cg	0.469722479	8.49155148	3.948599988	7.55E-04	1.50E-03	-1.768801052
Mir155	0.46971852	5.804127117	1.508308536	1.47E-01	1.85E-01	-6.714858078
Casp3	0.467397734	8.329072314	3.884430188	8.79E-04	1.72E-03	-1.919539629
Pde6d	0.46727615	8.537532495	3.655818158	1.51E-03	2.84E-03	-2.453142381
Runx3	0.467067976	9.058005508	2.918637014	8.31E-03	1.35E-02	-4.112836216
Foxn2	0.465969233	9.011833285	6.597407141	1.71E-06	5.67E-06	4.391975617
Actn1	0.465734227	8.808033297	2.804145077	1.07E-02	1.71E-02	-4.358202414
Arhgef12	0.465377914	5.942036877	4.420412181	2.47E-04	5.40E-04	-0.652847702
Arhgap39	0.465352127	7.282672981	2.666470074	1.46E-02	2.26E-02	-4.64733426
Agrn	0.464761448	6.351269059	2.605265255	1.67E-02	2.54E-02	-4.773612516
Hpgd	0.462986157	7.028232134	2.055822757	5.27E-02	7.33E-02	-5.832151524
Sbf2	0.462348563	8.228797713	5.274129342	3.33E-05	8.65E-05	1.366758528
Csgalnact2	0.461606463	7.744400232	3.450706041	2.44E-03	4.44E-03	-2.9259647
Irf2bp2	0.46157691	9.383922919	4.355741433	2.88E-04	6.20E-04	-0.806299756
Zc3h12c	0.461575842	9.266847298	4.750113064	1.13E-04	2.67E-04	0.129643366
Suv39h2	0.46149192	7.452299547	3.397900604	2.76E-03	4.96E-03	-3.046561065
2200002D01Rik	0.461354521	8.398306857	3.408048631	2.70E-03	4.86E-03	-3.023424877
Ccng2	0.461105214	9.114131972	4.096401879	5.32E-04	1.09E-03	-1.420399512
5430402E10Rik	0.461103616	1.758841863	1.357969045	1.89E-01	2.33E-01	-6.919920986
Rgag4	0.458257108	6.729223033	2.566801177	1.81E-02	2.75E-02	-4.852215712
H2-T10	0.454455603	6.970751562	2.676314704	1.43E-02	2.21E-02	-4.626888599
Oprd1	0.452003571	6.834656496	2.435674643	2.40E-02	3.56E-02	-5.115532383
Tango2	0.451811845	7.257445856	3.124544079	5.20E-03	8.81E-03	-3.661704227
Rnf166	0.449370969	7.394403935	3.055333433	6.09E-03	1.02E-02	-3.814649079
Uvrag	0.449165992	10.56590961	5.472856073	2.10E-05	5.68E-05	1.831653899
Slc43a2	0.448701172	6.186619644	1.763622896	9.26E-02	1.22E-01	-6.328102496
Abcb7	0.448581639	7.258104094	3.903146811	8.41E-04	1.65E-03	-1.875610489
Igkv4-70	0.448173325	1.575726063	0.946799	3.55E-01	4.09E-01	-7.385097221
Ell2	0.446343846	9.00578349	5.398005015	2.50E-05	6.68E-05	1.65689633
Tgoln1	0.446139987	9.000143253	5.445600709	2.24E-05	6.03E-05	1.768070595
Slc26a2	0.445666773	7.379477969	2.894555994	8.77E-03	1.42E-02	-4.164791741
Card11	0.443486746	7.6039283	3.291664105	3.54E-03	6.19E-03	-3.28755819
Olfr1175-ps	0.440296518	5.205853475	0.892101387	3.83E-01	4.38E-01	-7.435669944
LOC102639613	0.440161836	8.055301996	2.119770495	4.63E-02	6.53E-02	-5.716800645
Gpr52	0.439659676	6.361261711	2.657579745	1.49E-02	2.30E-02	-4.665766486
Wdr53	0.438871451	7.743575901	3.697609175	1.37E-03	2.59E-03	-2.356056205
Ptbp3	0.437070612	10.38102999	8.577333976	3.05E-08	1.40E-07	8.522759593
Cd47	0.436710187	10.94816477	8.229284435	5.96E-08	2.58E-07	7.833983881
Cblb	0.42959463	10.33819136	3.337866651	3.18E-03	5.64E-03	-3.183026289
Trappc11	0.428090015	8.115889079	4.997115863	6.34E-05	1.57E-04	0.714390607
Snx30	0.42651673	7.282775689	3.27966508	3.64E-03	6.35E-03	-3.314632372
Abcg1	0.426342938	9.144544143	3.788021967	1.10E-03	2.12E-03	-2.145278128
Fdft1	0.426244903	8.624388933	4.41771228	2.48E-04	5.43E-04	-0.659255411
Caprin2	0.425603377	6.906817971	2.81925528	1.04E-02	1.66E-02	-4.326065061
Tmed3	0.42537165	7.613231381	3.564959373	1.87E-03	3.47E-03	-2.663389949
Serhl	0.425193527	8.219425882	4.652439741	1.43E-04	3.27E-04	-0.102068709
Mir1190	0.425132973	7.932418002	3.3846181	2.85E-03	5.10E-03	-3.076814125
Sptan1	0.425045805	8.969610801	7.084674666	6.04E-07	2.17E-06	5.455999767
Mef2d	0.424493586	8.627504269	4.429920976	2.41E-04	5.30E-04	-0.630279617
LOC102631525	0.424062649	7.015069863	1.857821662	7.75E-02	1.04E-01	-6.174071164
Trp53inp1	0.423188258	7.784251399	3.238089268	4.00E-03	6.94E-03	-3.408200264
Calm5	0.422332281	6.071889688	1.314848836	2.03E-01	2.48E-01	-6.975434782
Traf3	0.422105059	8.499352135	5.21072905	3.85E-05	9.92E-05	1.217854011
P2rx4	0.422007599	8.693934776	4.58919089	1.65E-04	3.76E-04	-0.252191491
Gm10257	0.421963269	8.128439099	1.732012143	9.82E-02	1.29E-01	-6.378471605
Clec2d	0.421652105	10.52621348	4.953563921	7.02E-05	1.71E-04	0.611456122
Xist	0.421200978	11.16675979	3.831777347	9.96E-04	1.93E-03	-2.042941989
Ifi203	0.420993819	8.372503605	2.914008721	8.40E-03	1.37E-02	-4.122835826
Dennd4a	0.420446494	9.615690285	5.094493315	5.05E-05	1.27E-04	0.944210414
Zmym2	0.4189026	9.279056517	5.77774145	1.05E-05	2.98E-05	2.538586352
Tram1	0.416621326	9.937451896	5.2541768	3.48E-05	9.01E-05	1.319925719
Galk1	0.415563323	5.981854271	3.028748568	6.47E-03	1.08E-02	-3.873057664
Ly96	0.414773351	7.823256782	4.218432667	3.98E-04	8.37E-04	-1.131768199
Hist1h2bg	0.413982712	8.452634929	1.32522855	2.00E-01	2.44E-01	-6.962209499
Mob3a	0.413640923	9.829493819	4.293913758	3.33E-04	7.12E-04	-0.952922754
Ptdss2	0.413500273	6.587134826	3.169579325	4.69E-03	8.02E-03	-3.56152734
Nt5c3b	0.413378357	6.25784932	1.931984971	6.72E-02	9.18E-02	-6.048796827
Bmyc	0.413302098	9.304658059	2.662029656	1.47E-02	2.28E-02	-4.656544284
2810474O19Rik	0.412993347	9.376350358	5.534716353	1.83E-05	5.00E-05	1.975742737
Cyb561a3	0.41266553	10.2147701	4.272715888	3.50E-04	7.45E-04	-1.003168667
Gbp2	0.411837306	7.326664642	1.731855862	9.82E-02	1.29E-01	-6.378718943
Zfp46	0.410368184	6.952123732	4.092011543	5.38E-04	1.10E-03	-1.430769232
Hs6st1	0.410207808	6.677672939	2.864169571	9.40E-03	1.51E-02	-4.230091552

Dok1	0.40973296	7.398253906	2.907160031	8.53E-03	1.38E-02	-4.137620576
AU040320	0.409515874	7.521858658	4.802392626	1.00E-04	2.38E-04	0.253579211
Tex9	0.40749092	6.374412765	1.415806145	1.72E-01	2.14E-01	-6.8431207
Acer3	0.407264306	6.967284246	2.515535252	2.03E-02	3.04E-02	-4.956037705
Nedd4l	0.407230425	7.9162906	5.356121429	2.75E-05	7.31E-05	1.558922085
Trappc3	0.407170168	9.710688339	4.027168214	6.27E-04	1.27E-03	-1.583786882
Myo1c	0.407079395	7.491244337	4.687288544	1.31E-04	3.04E-04	-0.019376063
Cd48	0.404977724	9.552175874	4.97988738	6.60E-05	1.62E-04	0.673681497
Speer7-ps1	0.404869136	3.848460126	0.854232091	4.03E-01	4.59E-01	-7.469050626
LOC102631934	0.404055363	7.906667009	1.682963292	1.07E-01	1.40E-01	-6.455275152
Vkorc1	0.40399785	7.18523198	2.126058734	4.57E-02	6.45E-02	-5.705334544
Alcam	0.403406496	9.274051067	5.944562093	7.20E-06	2.11E-05	2.921700386
Pgls	0.40219954	9.883998196	4.962661282	6.87E-05	1.68E-04	0.632964597
Gtf2i	0.400205793	7.204460377	3.201253011	4.36E-03	7.49E-03	-3.490777045
Gm10432	0.398354834	5.427322899	2.172534492	4.16E-02	5.92E-02	-5.619921601
BC147527	0.398192022	6.31984269	1.303177764	2.07E-01	2.53E-01	-6.990200398
Rela	0.397963226	9.717072179	4.717032542	1.22E-04	2.86E-04	0.051186737
Apoe	0.396884352	8.081830388	1.717488021	1.01E-01	1.32E-01	-6.401386935
Racgap1	0.396753468	6.844890032	2.068134849	5.14E-02	7.18E-02	-5.810121955
Klf3	0.395717841	9.146957941	3.159432689	4.80E-03	8.20E-03	-3.584141217
Dpp7	0.395192303	7.18402913	1.91217408	6.98E-02	9.49E-02	-6.082597127
Prkag2	0.395064334	7.268781153	2.792913356	1.10E-02	1.75E-02	-4.382040726
Dock8	0.393655614	10.02759937	6.014548748	6.16E-06	1.84E-05	3.081585552
Rassf3	0.393559868	7.992389334	3.117750148	5.28E-03	8.93E-03	-3.67677275
Gbp8	0.391654935	9.647635722	2.464631916	2.26E-02	3.37E-02	-5.058022544
Poglut1	0.391006905	9.874388144	3.928265884	7.93E-04	1.57E-03	-1.816605481
Golph3l	0.390941951	9.021638138	4.379389686	2.72E-04	5.89E-04	-0.750195201
Gpr18	0.390582588	8.93181356	3.384171388	2.85E-03	5.10E-03	-3.077831002
Mknk2	0.390391413	8.580602412	4.570211714	1.73E-04	3.92E-04	-0.297245722
Cytip	0.388771899	9.433059202	3.922049082	8.04E-04	1.59E-03	-1.831213918
Mir467e	0.385117425	8.287390071	2.071142165	5.11E-02	7.14E-02	-5.804727952
Serpinb1a	0.384978864	7.963415846	1.650296734	1.14E-01	1.48E-01	-6.505497498
Ppp4r2	0.384628867	9.99914904	2.699924365	1.35E-02	2.11E-02	-4.577707462
Itgb1	0.383914633	10.05490668	5.045960944	5.66E-05	1.40E-04	0.82972965
Pitrm1	0.383247819	7.567220741	3.955473862	7.43E-04	1.48E-03	-1.752633236
Nptn	0.383061175	9.279358368	3.896716113	8.54E-04	1.67E-03	-1.890707339
Atp6v0c-ps2	0.382877326	9.775275537	3.213283109	4.24E-03	7.30E-03	-3.46384319
Stat3	0.380430098	10.09440044	5.524844473	1.87E-05	5.11E-05	1.952769718
Sbds	0.379977983	9.46117572	4.161222851	4.56E-04	9.48E-04	-1.267172959
Sepsecs	0.379775623	8.368442376	3.549814633	1.94E-03	3.59E-03	-2.69831654
Rhof	0.379619833	7.134903993	2.271970497	3.39E-02	4.91E-02	-5.433346935
Fh1	0.379381962	8.201755828	3.352203107	3.07E-03	5.47E-03	-3.150501857
Ppp1r15a	0.378853254	10.27427317	3.784200242	1.11E-03	2.14E-03	-2.154206737
Tagln2	0.378140053	10.8969888	5.158364578	4.35E-05	1.11E-04	1.094675934
2310014F06Rik	0.377370979	3.928415841	0.798134126	4.34E-01	4.88E-01	-7.516013224
Picalm	0.375570728	9.587280378	6.29517358	3.30E-06	1.04E-05	3.717355374
Elmo2	0.375008809	8.409033323	2.868407483	9.31E-03	1.50E-02	-4.221002015
Hecw2	0.374702354	5.536957892	1.963547384	6.32E-02	8.68E-02	-5.994449786
Map3k5	0.374623528	7.983284828	3.30597055	3.42E-03	6.01E-03	-3.255237682
Rogdi	0.374054302	11.30467892	4.472221474	2.18E-04	4.84E-04	-0.529872476
Mrpl24	0.373351075	10.14407526	6.310871909	3.19E-06	1.01E-05	3.752660005
Dpp4	0.372660078	10.14600062	4.191706248	4.24E-04	8.87E-04	-1.19504247
Marcks	0.371817594	8.350355368	4.573006196	1.72E-04	3.90E-04	-0.290611838
Cmtm3	0.370850549	8.747457542	3.729745651	1.27E-03	2.41E-03	-2.281247991
Flt3l	0.370058952	8.331057735	2.355826404	2.85E-02	4.16E-02	-5.272139067
Ube2e2	0.369069608	6.800375566	2.405520039	2.56E-02	3.78E-02	-5.175019846
Lactb	0.36792584	8.647504152	3.289213802	3.56E-03	6.21E-03	-3.293089466
Cyth1	0.366913807	8.428855772	3.308071539	3.40E-03	5.99E-03	-3.250487598
Setd2	0.366511652	9.651018978	6.004523359	6.30E-06	1.88E-05	3.058713948
Cbx7	0.365704987	7.649549545	2.82279091	1.03E-02	1.65E-02	-4.318534199
Eif1	0.364490955	12.01633072	5.166733838	4.27E-05	1.09E-04	1.114374357
Arg2	0.363263531	5.833276439	1.466064533	1.58E-01	1.98E-01	-6.77425008
Smarce1	0.362924686	9.745781375	2.371775596	2.75E-02	4.04E-02	-5.241094384
Lyn	0.362755967	10.11607452	4.034879428	6.16E-04	1.25E-03	-1.565604217
Sord	0.361982093	5.496681871	0.776041882	4.47E-01	5.02E-01	-7.533685554
Mndal	0.360280292	8.763007765	2.885457108	8.96E-03	1.45E-02	-4.184375764
Zdhhc3	0.360090778	8.07570634	4.255574451	3.65E-04	7.71E-04	-1.043788911
Hmgb1	0.360063273	6.580863385	1.091453267	2.88E-01	3.39E-01	-7.238204937
Hbp1	0.359675328	8.964966265	4.183223571	4.33E-04	9.04E-04	-1.21511863
Gnai3	0.359558097	9.070254675	5.625303449	1.48E-05	4.12E-05	2.186161116
Gdi1	0.358112708	9.975047132	3.844928406	9.65E-04	1.87E-03	-2.012145267
Xcl1	0.358066991	8.215646694	0.736462367	4.70E-01	5.24E-01	-7.564174162
Kansl1	0.354909437	9.188588773	4.011454426	6.51E-04	1.31E-03	-1.620826494
Polr3c	0.354417425	9.077878641	4.524022087	1.93E-04	4.32E-04	-0.406899842
Gstp1	0.354337419	9.459110516	2.841462714	9.89E-03	1.59E-02	-4.278694603
Depdc5	0.352615957	7.623876108	3.178596712	4.59E-03	7.87E-03	-3.541409332

Nfkbia	0.35135173	11.18519394	5.394770664	2.52E-05	6.73E-05	1.649335208
Slc38a9	0.35121597	8.196513986	2.945984617	7.81E-03	1.28E-02	-4.053617403
Atp13a3	0.351020596	7.985150653	3.156783211	4.83E-03	8.25E-03	-3.590041997
Mtmr4	0.350116421	9.287022832	5.58248304	1.64E-05	4.52E-05	2.086784965
Wrn	0.348127778	8.562542319	4.225282039	3.92E-04	8.24E-04	-1.115547739
Slc12a2	0.348120963	6.947475412	2.397775317	2.60E-02	3.84E-02	-5.190231289
Rbm38	0.347797429	8.380431812	2.94005172	7.92E-03	1.30E-02	-4.066483776
N6amt2	0.346161209	7.558025266	1.694577151	1.05E-01	1.37E-01	-6.43723971
Dhrs3	0.345587971	7.197166405	2.542786223	1.91E-02	2.89E-02	-4.900986121
H2-Q5	0.344321736	9.356464689	3.639636743	1.57E-03	2.94E-03	-2.490671721
Traf2	0.344286961	7.593529067	3.548183503	1.94E-03	3.60E-03	-2.702076112
Tmem231	0.344148863	6.565367204	2.083016038	4.99E-02	6.99E-02	-5.783380603
Etfdh	0.344129882	8.40496869	2.957537532	7.61E-03	1.25E-02	-4.028532993
Iqcj	0.343882318	3.108108309	0.532390001	6.00E-01	6.49E-01	-7.697052208
Myo1g	0.342104029	10.208374	4.027989127	6.26E-04	1.27E-03	-1.581851402
Pigyl	0.341403799	8.10939723	2.567899081	1.81E-02	2.74E-02	-4.849980388
H2-Q4	0.341029285	8.183913814	3.458905753	2.40E-03	4.36E-03	-2.907193308
Uggt1	0.34088313	8.718364477	5.10842796	4.89E-05	1.24E-04	0.97705683
Evi2a	0.340596148	9.687468554	3.356769957	3.04E-03	5.41E-03	-3.140132663
Slc38a1	0.338637245	8.215991186	1.587187458	1.28E-01	1.64E-01	-6.600371513
B4galt5	0.338524649	8.211726051	2.912405814	8.43E-03	1.37E-02	-4.126297432
Mllt6	0.338268931	7.819269665	3.236177968	4.02E-03	6.96E-03	-3.412492489
Rgmb	0.33792728	7.2767953	2.94370236	7.85E-03	1.29E-02	-4.058568068
LOC102633400	0.337680964	6.302574915	1.820727884	8.32E-02	1.11E-01	-6.23541847
Fbxo11	0.336205457	9.53830609	4.846330849	9.03E-05	2.17E-04	0.357679818
Cyp51	0.335522503	7.296512703	2.234221403	3.67E-02	5.27E-02	-5.504777827
2310001H17Rik	0.334937269	8.15331373	2.463241287	2.27E-02	3.37E-02	-5.060792859
Phlpp1	0.333848039	8.109536035	3.415508585	2.65E-03	4.78E-03	-3.00640492
Tmem127	0.333565493	8.145187911	3.708440631	1.33E-03	2.52E-03	-2.330856692
Scn4b	0.332818363	5.986040055	1.847133334	7.91E-02	1.06E-01	-6.191838939
Gm10865	0.332561005	6.550821236	4.351035115	2.91E-04	6.26E-04	-0.817463943
Kdelr1	0.332523847	9.118197367	2.83621998	1.00E-02	1.60E-02	-4.28989253
Lphn3	0.332507925	4.470689147	1.377918037	1.83E-01	2.26E-01	-6.893731652
Tpd52	0.331616909	7.324192737	2.018037762	5.68E-02	7.85E-02	-5.899214071
Otulin	0.330471891	9.307573209	5.41689992	2.39E-05	6.42E-05	1.701052084
Nfat5	0.329791326	8.502267883	3.086652398	5.67E-03	9.53E-03	-3.745594433
Itga4	0.32863152	10.21937184	4.391816353	2.64E-04	5.73E-04	-0.720709223
Phpt1	0.328437717	8.603423087	3.4386375	2.51E-03	4.56E-03	-2.953571262
Rps6ka1	0.3277003	8.732453714	3.107456852	5.41E-03	9.13E-03	-3.699580233
Pdgfa	0.326982006	6.39406221	2.136857848	4.48E-02	6.32E-02	-5.685592586
Peak1	0.326221627	9.454094552	3.576147036	1.82E-03	3.39E-03	-2.637566596
H1f0	0.324744363	9.316212284	2.611236021	1.65E-02	2.52E-02	-4.761357902
Dennd5a	0.323733974	8.708794333	3.877918424	8.93E-04	1.74E-03	-1.934815633
Rras	0.321374037	8.651716488	2.380092978	2.70E-02	3.97E-02	-5.224857379
Hhex	0.320921913	7.354486823	1.158448581	2.60E-01	3.10E-01	-7.163874839
Appl1	0.320841187	8.877970544	4.242778754	3.76E-04	7.93E-04	-1.074104388
Tatdn3	0.320807509	7.049340352	1.782403057	8.94E-02	1.18E-01	-6.297859783
Tcf3	0.319108345	8.473646513	3.475453602	2.30E-03	4.21E-03	-2.869275051
Commd3	0.318416896	8.881836971	3.709788618	1.33E-03	2.52E-03	-2.327719561
LOC102640457	0.316772981	7.147407536	1.541502346	1.38E-01	1.76E-01	-6.667243263
Tmem50a	0.316550386	9.265576208	3.248803082	3.91E-03	6.78E-03	-3.384124961
Nrm	0.316369069	8.795855381	2.683538469	1.40E-02	2.18E-02	-4.611862868
Mir1962	0.315152293	4.291092035	1.536866638	1.40E-01	1.77E-01	-6.673942493
Bin2	0.314595786	7.695937308	1.827419165	8.21E-02	1.10E-01	-6.22441792
Phf14	0.314480343	7.966640981	2.692900378	1.38E-02	2.14E-02	-4.592360732
Tmem206	0.313306153	7.697016617	2.300272121	3.20E-02	4.64E-02	-5.379323449
Zyx	0.31323728	9.84032185	2.932668039	8.05E-03	1.32E-02	-4.082481585
Hsf2	0.312970341	7.690120968	2.595845651	1.70E-02	2.59E-02	-4.792916864
Emp3	0.312527294	9.850826433	2.840594941	9.91E-03	1.59E-02	-4.280548699
Colq	0.31250773	6.151374129	1.638830532	1.16E-01	1.51E-01	-6.522947213
Atp1a1	0.312198052	9.934634783	3.150649413	4.90E-03	8.35E-03	-3.603696271
Bag3	0.312034013	7.100645481	2.572233151	1.79E-02	2.72E-02	-4.841151453
Grina	0.311456825	8.795838638	2.636516344	1.56E-02	2.39E-02	-4.709316405
Grn	0.311056622	11.13126643	3.865377913	9.20E-04	1.79E-03	-1.964223315
Snord87	0.310805734	6.231727455	1.174330984	2.54E-01	3.04E-01	-7.145682693
Pts	0.310152727	8.813859412	3.092901032	5.59E-03	9.40E-03	-3.731785867
Cdk13	0.308596931	9.323093535	3.153386623	4.87E-03	8.31E-03	-3.597604187
Rhoc	0.308210789	7.49019953	1.036135356	3.12E-01	3.65E-01	-7.296601018
Pmaip1	0.307444775	8.382504491	2.257623702	3.49E-02	5.03E-02	-5.460579845
Uck2	0.30708756	6.607527817	1.764549769	9.24E-02	1.22E-01	-6.326615435
Anxa5	0.306731893	10.3067509	4.735820098	1.17E-04	2.74E-04	0.095747936
Arrdc1	0.306713204	9.097398201	3.657576192	1.50E-03	2.83E-03	-2.449062862
Zrsr1	0.305522618	6.209299522	2.31175217	3.12E-02	4.54E-02	-5.357296972
Smarca2	0.304880456	8.972771849	4.433518666	2.39E-04	5.26E-04	-0.621740607
B230398E01Rik	0.304668344	9.002483335	2.16104299	4.26E-02	6.04E-02	-5.641149248
Gpbp1	0.3032362	9.698651061	4.15059449	4.68E-04	9.70E-04	-1.292311692

Gm2382	0.30312308	10.00605864	1.780101914	8.98E-02	1.19E-01	-6.301578083
Msmo1	0.303103136	7.744015209	3.237256962	4.01E-03	6.95E-03	-3.410069484
Kctd12	0.301821355	10.05192945	2.017533485	5.68E-02	7.86E-02	-5.900103475
Naip5	0.300046058	8.110632216	1.918218936	6.90E-02	9.40E-02	-6.072309426
Gpc1	0.298557155	6.524127934	2.180722064	4.09E-02	5.83E-02	-5.604754082
Lamtor4	0.298097639	8.438136959	1.87630172	7.48E-02	1.01E-01	-6.143178749
Mid1	0.297828129	7.900943565	1.008404133	3.25E-01	3.78E-01	-7.324845929
Dram2	0.296704979	7.549955651	2.094216056	4.88E-02	6.85E-02	-5.763171895
Suds3	0.294546793	9.069120209	2.951241589	7.72E-03	1.27E-02	-4.042208067
Csf2rb	0.294354623	10.72379572	2.110736746	4.72E-02	6.64E-02	-5.733234779
Stk10	0.293206946	8.138296859	2.200961621	3.93E-02	5.62E-02	-5.567107746
Anxa2	0.2910268	10.57800886	4.153268457	4.65E-04	9.65E-04	-1.285987613
Hmg20b	0.290784178	8.10281802	2.882986691	9.01E-03	1.45E-02	-4.18968847
Ggct	0.290517196	6.79921403	1.119079317	2.76E-01	3.27E-01	-7.208028883
9530059O14Rik	0.290270018	5.069075082	1.270615784	2.18E-01	2.65E-01	-7.030804163
Bsdc1	0.288966129	8.808291864	3.309471276	3.39E-03	5.98E-03	-3.24732245
Ube2r2	0.288901013	8.35941618	3.405951398	2.71E-03	4.88E-03	-3.028207876
Traf1	0.288574597	10.06478964	2.837202618	9.98E-03	1.60E-02	-4.287794409
Rhog	0.287597978	9.883337307	3.832022601	9.95E-04	1.93E-03	-2.042367821
1110058L19Rik	0.286582137	6.584738351	1.393341166	1.78E-01	2.21E-01	-6.873266486
Tbc1d20	0.286540207	8.748389918	2.755510275	1.20E-02	1.89E-02	-4.461112262
BC033916	0.286536027	5.963136809	1.083124419	2.91E-01	3.43E-01	-7.247170861
Csnk1g1	0.285532728	7.984030339	3.308455782	3.40E-03	5.99E-03	-3.249618771
Tsc22d1	0.284459492	7.729004776	2.991026213	7.05E-03	1.17E-02	-3.955598304
Kcnn1	0.284423617	5.619274886	1.053692535	3.04E-01	3.57E-01	-7.278361859
Hmgcs1	0.282983159	8.270747815	2.445873577	2.35E-02	3.49E-02	-5.095319616
Ighv6-3	0.282284024	2.451755772	0.50870395	6.16E-01	6.64E-01	-7.709792394
Tnni3	0.28177198	8.266889756	2.320110061	3.07E-02	4.46E-02	-5.341220371
Plekha3	0.281658168	8.383996101	2.557395259	1.85E-02	2.80E-02	-4.871345873
Dapp1	0.281077736	9.459491782	4.441857562	2.34E-04	5.17E-04	-0.601947928
Os9	0.281005359	9.088481992	3.287245873	3.57E-03	6.24E-03	-3.297530913
Vrk1	0.280846564	8.933395091	2.911612182	8.44E-03	1.37E-02	-4.12801105
Rassf4	0.275956267	10.26089652	4.56151817	1.77E-04	3.99E-04	-0.317883747
LOC102634079	0.275830856	6.308990362	1.040639231	3.10E-01	3.63E-01	-7.291948491
Gnptg	0.275814386	8.106560968	2.19916287	3.94E-02	5.63E-02	-5.570462231
Nfkb2	0.275738418	9.151319051	3.435683025	2.53E-03	4.59E-03	-2.960325605
Dnase1l1	0.27563131	8.219977996	2.66001491	1.48E-02	2.29E-02	-4.660720671
LOC102633000	0.275419813	6.354907755	0.864428831	3.97E-01	4.53E-01	-7.460194854
Tmed4	0.275353495	8.477933646	1.916663416	6.92E-02	9.43E-02	-6.074958919
Use1	0.27507888	11.05664512	2.836112068	1.00E-02	1.60E-02	-4.290122925
Phip	0.274499714	9.450808787	4.134938301	4.86E-04	1.00E-03	-1.329332202
Pdk3	0.273959225	8.710002216	3.096538449	5.55E-03	9.34E-03	-3.723743011
Zfp36l2	0.273417569	8.750356119	2.696643881	1.36E-02	2.12E-02	-4.584553398
Sema7a	0.272770813	7.618505621	1.858142992	7.75E-02	1.04E-01	-6.173535867
Lbr	0.271390514	6.872942257	1.463158142	1.58E-01	1.99E-01	-6.778286089
Sh3glb1	0.270696335	10.1358786	4.017056616	6.42E-04	1.30E-03	-1.607623329
Chd2	0.269376505	8.829140919	3.060996632	6.02E-03	1.01E-02	-3.802181812
Lpcat3	0.265380953	7.290454107	2.045410735	5.38E-02	7.47E-02	-5.85071364
Fam69a	0.263264493	8.681197424	2.698722856	1.36E-02	2.11E-02	-4.580215313
Slc7a6	0.262006963	7.448704038	2.356152577	2.84E-02	4.16E-02	-5.271505384
Mtss1l	0.261400755	6.853228425	1.397053604	1.77E-01	2.20E-01	-6.86831223
Crlf3	0.261316067	8.558866861	2.451794718	2.32E-02	3.45E-02	-5.083563498
Map3k14	0.260935542	8.625553068	2.596711135	1.70E-02	2.59E-02	-4.791144634
Arhgap28	0.259346216	6.962414143	1.56861216	1.32E-01	1.69E-01	-6.627746608
LOC102639182	0.259252093	2.298826289	0.47109322	6.43E-01	6.89E-01	-7.728859362
Ighv5-16	0.259013371	2.585387162	0.336120755	7.40E-01	7.79E-01	-7.785429899
Arhgef2	0.257334763	8.722576086	3.303768309	3.44E-03	6.03E-03	-3.260215696
Gm5471	0.25731346	11.02602831	3.320332663	3.31E-03	5.85E-03	-3.222748363
Samd4b	0.257053557	8.055276218	2.170207771	4.18E-02	5.95E-02	-5.62422534
Lrrk1	0.256952599	9.569106877	3.31854856	3.32E-03	5.87E-03	-3.226786602
Ptpn21	0.255235035	7.046424588	1.914967062	6.95E-02	9.45E-02	-6.077846584
Ppp3cc	0.254252108	6.679372703	1.486153371	1.52E-01	1.92E-01	-6.74617623
Glcci1	0.254140679	7.947170583	2.147971505	4.38E-02	6.19E-02	-5.665209159
Sgpl1	0.253974556	9.122644762	3.461411695	2.38E-03	4.34E-03	-2.901454167
Mien1	0.25369532	9.561841625	3.136950715	5.05E-03	8.57E-03	-3.634156997
Gmps	0.252628248	9.116218908	4.304845406	3.24E-04	6.95E-04	-0.927005896
P2ry10	0.252536663	8.979875573	2.900764413	8.65E-03	1.40E-02	-4.151414123
Tmx3	0.252157029	9.148375192	3.858172551	9.36E-04	1.82E-03	-1.981113207
Ndufaf7	0.252087384	7.349962194	2.082864693	4.99E-02	6.99E-02	-5.783653198
Sav1	0.251335624	8.891017874	3.024165933	6.54E-03	1.09E-02	-3.883106318
Psmd10	0.250683602	8.944532348	3.431267046	2.56E-03	4.63E-03	-2.970418219
Tbc1d15	0.250480531	9.021130157	3.477025279	2.30E-03	4.20E-03	-2.865671207
Zfp715	0.250426037	5.639183837	0.966451055	3.45E-01	3.99E-01	-7.366253279
Gatad1	0.249620848	9.175970781	2.695952421	1.37E-02	2.13E-02	-4.585995875
Akap13	0.248469484	9.638277845	3.294976476	3.51E-03	6.14E-03	-3.280078865
5031439G07Rik	0.247057191	7.97473599	2.657284536	1.49E-02	2.30E-02	-4.666378025

Krit1	0.24648956	8.286597696	3.147593378	4.93E-03	8.40E-03	-3.610495768
Dync1h1	0.245831776	9.555243113	4.089426929	5.41E-04	1.11E-03	-1.436873415
Atxn1	0.245778487	7.02812664	1.617553403	1.21E-01	1.56E-01	-6.555079039
Gpr171	0.244533633	8.739253887	1.39231852	1.79E-01	2.21E-01	-6.874629293
Plekhg1	0.243487186	5.524182346	1.279706893	2.15E-01	2.61E-01	-7.019555903
Lgals3	0.242760703	9.583503698	1.768603341	9.17E-02	1.21E-01	-6.320105176
Dhcr24	0.242336638	7.184347266	1.711191223	1.02E-01	1.33E-01	-6.411276739
Rspry1	0.241602669	7.898636647	2.503526178	2.08E-02	3.12E-02	-4.980198453
Bcor	0.241543668	8.238829559	2.420265485	2.48E-02	3.67E-02	-5.145982338
Arf4	0.240655532	9.83413132	3.724226945	1.28E-03	2.44E-03	-2.2941036
Dnm1l	0.240635562	9.79228356	2.886410076	8.94E-03	1.45E-02	-4.182325861
Zfand6	0.240119066	9.453075983	2.385525461	2.67E-02	3.93E-02	-5.214234772
Herc6	0.239510831	8.480791638	2.313519731	3.11E-02	4.52E-02	-5.353899865
Ankib1	0.239095561	8.592629792	2.924679266	8.20E-03	1.34E-02	-4.099771806
Nae1	0.238667698	8.030288705	2.891011679	8.84E-03	1.43E-02	-4.172423457
Eya3	0.238051448	9.194532742	3.230544004	4.07E-03	7.05E-03	-3.425139942
Map4k4	0.237525013	8.980275759	3.416062238	2.65E-03	4.77E-03	-3.005141345
Arfgap3	0.23698776	6.716300029	1.561481398	1.34E-01	1.71E-01	-6.638188172
N4bp2l1	0.236515825	9.087434618	2.359753294	2.82E-02	4.14E-02	-5.264506627
Mitd1	0.23635877	7.480407271	1.576523692	1.30E-01	1.67E-01	-6.616118
Rsu1	0.235633337	10.5514756	2.76341893	1.18E-02	1.86E-02	-4.444433669
Dtx1	0.234068371	6.126468786	1.265691968	2.20E-01	2.67E-01	-7.036867697
LOC102634822	0.233326919	7.604991526	1.135092035	2.69E-01	3.20E-01	-7.190232465
Cyp4f37	0.233277168	5.153721191	0.874474734	3.92E-01	4.47E-01	-7.451374554
Etfb	0.23282531	9.073510347	2.347524028	2.90E-02	4.23E-02	-5.28825175
Cysltr1	0.232447744	5.337569228	0.937932653	3.59E-01	4.14E-01	-7.393482833
Rnf141	0.231877842	7.396223843	1.387573786	1.80E-01	2.23E-01	-6.880941414
Supt4a	0.230814357	11.12729739	2.255322152	3.51E-02	5.05E-02	-5.464938946
Flna	0.230082002	9.697425211	2.542494296	1.91E-02	2.89E-02	-4.90157751
A930013F10Rik	0.22989028	7.637995143	1.657257539	1.13E-01	1.46E-01	-6.494858795
Snord65	0.229036975	9.484607333	1.626145515	1.19E-01	1.54E-01	-6.542142619
Prps1	0.228933428	7.916576972	1.440301638	1.65E-01	2.06E-01	-6.809798906
Tmem64	0.228028067	7.521046326	1.533852326	1.40E-01	1.78E-01	-6.678289989
Snx5	0.227356197	9.708288176	2.710838078	1.32E-02	2.07E-02	-4.554903497
Ehmt1	0.226962166	8.528673404	2.702475867	1.35E-02	2.10E-02	-4.572380074
Nrros	0.226453661	8.923187298	3.540610921	1.98E-03	3.66E-03	-2.719524612
Tmem39a	0.226112843	9.613834003	1.904577299	7.09E-02	9.61E-02	-6.095493861
Zfp414	0.224975986	8.581325737	2.098527046	4.84E-02	6.80E-02	-5.755374632
Pura	0.224522114	7.323970896	1.233724343	2.31E-01	2.80E-01	-7.075743193
Ddi2	0.222494801	8.796848232	2.081457869	5.00E-02	7.01E-02	-5.786186487
Krtcap2	0.22247047	9.686981593	2.234306151	3.67E-02	5.27E-02	-5.504618275
Fcgrt	0.222202747	8.735862904	1.640513881	1.16E-01	1.50E-01	-6.520391279
Cd53	0.22190689	10.3504919	1.649665443	1.14E-01	1.48E-01	-6.506460648
Fcrla	0.221246324	6.499836691	1.398846427	1.77E-01	2.19E-01	-6.8659158
Lsp1	0.220436845	11.68298488	4.079747138	5.53E-04	1.13E-03	-1.459731026
Eif4a3	0.21960899	6.013763245	0.619594244	5.42E-01	5.93E-01	-7.645306026
Clic4	0.218453308	10.61398822	2.472719979	2.22E-02	3.31E-02	-5.041893223
8430408G22Rik	0.218437434	6.675134819	1.409085941	1.74E-01	2.16E-01	-6.852180233
Igkv4-59	0.218211905	2.7268373	0.413037166	6.84E-01	7.28E-01	-7.755473807
Fam129a	0.2176535	9.094217316	3.226257124	4.11E-03	7.10E-03	-3.434758558
Rabac1	0.216798257	9.083481699	2.232238771	3.68E-02	5.29E-02	-5.508509392
H2-Q6	0.216774932	10.73244139	2.295001724	3.23E-02	4.69E-02	-5.389413928
Fam111a	0.216743829	8.15542721	1.445018282	1.63E-01	2.05E-01	-6.80332907
Lnx2	0.215979071	7.189718792	1.912894206	6.97E-02	9.48E-02	-6.081372739
Kmt2a	0.215343493	9.195752649	2.691429558	1.38E-02	2.14E-02	-4.595426809
Sat1	0.215057875	11.63745037	2.654756426	1.50E-02	2.31E-02	-4.671613752
Lst1	0.214675704	9.537098751	1.697821803	1.05E-01	1.37E-01	-6.432184264
Eml4	0.213915951	9.028889358	1.902786349	7.11E-02	9.64E-02	-6.098529045
Vhl	0.213263777	8.161101895	1.404871104	1.75E-01	2.17E-01	-6.857844143
Itm2c	0.212875607	9.802697248	2.50000023	2.09E-02	3.14E-02	-4.987280526
Pip4k2a	0.212203015	10.93120768	3.543511086	1.96E-03	3.63E-03	-2.712843208
Marf1	0.211100453	9.205010362	2.952524265	7.70E-03	1.27E-02	-4.039422993
Spint1	0.210936955	7.095951847	1.21785129	2.37E-01	2.86E-01	-7.094727656
Ktn1	0.21015348	8.698810043	2.496547218	2.11E-02	3.16E-02	-4.994210933
C2cd2	0.208726669	6.296979245	1.118905324	2.76E-01	3.27E-01	-7.208221029
Ehd1	0.207106621	7.623871147	1.629301326	1.18E-01	1.53E-01	-6.537377885
Tubb2b	0.206932095	7.767366025	1.192206071	2.47E-01	2.97E-01	-7.12494925
Pcmtd1	0.206378893	8.975362427	2.735042283	1.25E-02	1.97E-02	-4.504174575
Ube4b	0.206235144	8.991661438	2.462971882	2.27E-02	3.37E-02	-5.061329453
Akap8l	0.205760356	8.469892613	2.137968842	4.47E-02	6.31E-02	-5.68355795
Dpysl2	0.205695305	7.879435438	1.519336772	1.44E-01	1.82E-01	-6.699130357
Rmrp	0.205178337	9.957613214	1.211327266	2.39E-01	2.88E-01	-7.102468813
Scel	0.204972976	3.388807078	1.276537006	2.16E-01	2.63E-01	-7.023485718
Pvr	0.2047133	8.29720449	2.061644095	5.21E-02	7.26E-02	-5.821746401
H2-K1	0.203444285	11.6412621	2.808522596	1.06E-02	1.70E-02	-4.348899934
Arl5a	0.202466494	9.694752634	1.918675379	6.90E-02	9.40E-02	-6.071531689

Lppr4	0.201420855	5.961044456	1.073754765	2.95E-01	3.47E-01	-7.257183922
Tnfrsf21	0.20141579	6.930871234	1.393537611	1.78E-01	2.21E-01	-6.873004603
Stat6	0.199770688	8.903458899	2.416378257	2.50E-02	3.70E-02	-5.153646892
6330407A03Rik	0.199565137	8.09860998	2.001083149	5.87E-02	8.10E-02	-5.929035347
Nub1	0.19932139	8.86451565	2.725449664	1.28E-02	2.01E-02	-4.524304824
Hexb	0.198917264	8.813483892	2.514453747	2.03E-02	3.05E-02	-4.958216071
Ndufaf4	0.197886215	6.9113349	1.116974139	2.77E-01	3.28E-01	-7.210351923
Man2a1	0.197784829	8.488955669	2.398063002	2.60E-02	3.84E-02	-5.189666737
Sri	0.196273168	10.83247243	3.027998865	6.49E-03	1.08E-02	-3.874701989
Iscu	0.194273664	9.869137104	1.794646751	8.74E-02	1.16E-01	-6.278016752
Ostf1	0.194077673	11.35565169	2.530102054	1.96E-02	2.96E-02	-4.92664908
G530011O06Rik	0.193976381	6.955013514	0.504222931	6.19E-01	6.66E-01	-7.712139063
Ubxn4	0.193873658	9.53107007	2.529114903	1.97E-02	2.96E-02	-4.928643488
Slc9a3r1	0.193566999	8.843901642	2.009380679	5.78E-02	7.98E-02	-5.914462043
Cd101	0.192687303	5.257482658	0.908663716	3.74E-01	4.29E-01	-7.420649436
Flt3	0.19193493	10.87706901	2.57876707	1.77E-02	2.68E-02	-4.827826773
Lrrc61	0.19126751	7.267981701	1.724293854	9.96E-02	1.31E-01	-6.390667042
Gm12100	0.191163527	2.018283733	0.813620538	4.25E-01	4.80E-01	-7.503347224
Plekhm2	0.19049662	7.419033372	1.68948552	1.06E-01	1.39E-01	-6.44515818
Tlr1	0.190490691	8.018519773	0.973037238	3.42E-01	3.96E-01	-7.359858807
Nuak2	0.190035126	8.188889694	1.555157717	1.35E-01	1.72E-01	-6.647416542
Acaa2	0.189474171	7.408279496	1.735390646	9.76E-02	1.28E-01	-6.373120551
Eps15	0.189156849	9.660893947	2.866585892	9.34E-03	1.50E-02	-4.224909698
Gpx1	0.188658102	11.64115289	3.307837199	3.41E-03	5.99E-03	-3.251017457
Dcps	0.188136982	7.928724614	1.933409462	6.70E-02	9.16E-02	-6.046357115
Ppif	0.187304564	7.622169975	1.117646749	2.77E-01	3.27E-01	-7.209610127
Ankle2	0.186227769	8.504328128	2.178130911	4.12E-02	5.86E-02	-5.609558066
Nxf1	0.183480515	10.45560388	2.743538664	1.23E-02	1.93E-02	-4.486317245
Acot9	0.182021396	7.06614501	1.07971748	2.93E-01	3.45E-01	-7.250820741
Birc3	0.181724132	10.86469779	2.32331757	3.05E-02	4.44E-02	-5.335041632
Fis1	0.18108192	10.51555921	1.794183545	8.75E-02	1.16E-01	-6.278769264
Dnajc5	0.17923353	8.918370937	2.0632926	5.19E-02	7.24E-02	-5.818796329
Fam60a	0.178996713	6.187891512	0.635781967	5.32E-01	5.83E-01	-7.634868395
Zfp524	0.17863973	7.831154726	1.808259354	8.52E-02	1.13E-01	-6.255838972
Dusp11	0.178639033	9.632591157	2.166473551	4.21E-02	5.98E-02	-5.631126478
Zfp945	0.176810361	7.496625102	1.346788375	1.93E-01	2.37E-01	-6.934459565
Taf9b	0.176035635	7.502412357	1.60864378	1.23E-01	1.58E-01	-6.56843741
Polb	0.175728005	9.055188819	1.822382702	8.29E-02	1.11E-01	-6.23270064
Ifih1	0.174619217	8.197264781	1.626592228	1.19E-01	1.53E-01	-6.541468592
Wipf1	0.174565177	8.131525949	1.939018953	6.63E-02	9.07E-02	-6.036737691
Gm2848	0.173394533	6.734693051	0.902055779	3.77E-01	4.33E-01	-7.426672841
Cep57	0.173105755	7.434842583	2.477026183	2.20E-02	3.28E-02	-5.03329405
Mir483	0.172885322	6.706860417	0.726709996	4.76E-01	5.30E-01	-7.571454256
Brk1	0.170491486	9.960923542	1.145108186	2.65E-01	3.16E-01	-7.178987203
Ccng1	0.168537106	10.01916553	2.53915457	1.92E-02	2.91E-02	-4.908340643
Kdm6b	0.168191481	8.228598405	1.320306565	2.01E-01	2.46E-01	-6.968491773
H2-T22	0.167308656	9.143150084	1.306403203	2.06E-01	2.52E-01	-6.98613089
Tmem68	0.165674271	8.040951717	2.084557557	4.97E-02	6.98E-02	-5.780603354
D17Wsu92e	0.165431543	8.837116183	1.645989125	1.15E-01	1.49E-01	-6.512063938
Rabgap1	0.164790626	8.381419137	1.968523247	6.26E-02	8.60E-02	-5.985826748
Nfkbid	0.164765931	8.235729146	1.010531752	3.24E-01	3.77E-01	-7.322703443
Sec61b	0.164676941	9.228497948	0.914450249	3.71E-01	4.26E-01	-7.41534146
Pex13	0.164627668	7.690457477	1.330305182	1.98E-01	2.43E-01	-6.955709202
Me2	0.164426872	8.91666079	2.002684423	5.85E-02	8.07E-02	-5.926226124
Mtf1	0.162331983	8.110609612	2.290794785	3.26E-02	4.72E-02	-5.397458512
Atp6v1g1	0.16228999	10.63373007	1.995561699	5.94E-02	8.18E-02	-5.938710368
Ccni	0.161452956	9.916304015	1.487496465	1.52E-01	1.92E-01	-6.744288275
Psenen	0.160534549	10.19863731	1.213416657	2.39E-01	2.88E-01	-7.099993543
Herpud1	0.160259575	10.36268195	2.392711493	2.63E-02	3.88E-02	-5.200162226
Scd2	0.159618193	7.327463785	1.102672825	2.83E-01	3.34E-01	-7.226030559
Srpk2	0.158993287	7.386154351	1.406889715	1.74E-01	2.17E-01	-6.855133274
S100pbp	0.158807605	8.08926657	1.957421193	6.40E-02	8.78E-02	-6.005045781
Aup1	0.158164691	10.31270206	2.021625249	5.64E-02	7.81E-02	-5.892882459
H2-D1	0.15807203	12.72114069	2.036102204	5.48E-02	7.60E-02	-5.867255623
LOC102631592	0.156970993	4.568339939	0.819325319	4.22E-01	4.77E-01	-7.498623872
Ezh1	0.156906402	8.469051689	1.666813287	1.11E-01	1.44E-01	-6.480198338
Padi2	0.156887256	6.734578723	1.04362025	3.09E-01	3.61E-01	-7.288859092
Ict1	0.156506506	8.004206762	1.15358801	2.62E-01	3.12E-01	-7.169398838
Ginm1	0.156292482	7.837860918	1.503110839	1.48E-01	1.87E-01	-6.722238862
Vmp1	0.155496139	7.727168419	1.725509234	9.94E-02	1.30E-01	-6.388749356
Med11	0.155106571	8.519640437	1.808141284	8.52E-02	1.13E-01	-6.256031857
Fut8	0.155057706	8.170669602	2.149457017	4.36E-02	6.18E-02	-5.662479515
A230045G11Rik	0.153824785	9.136361181	1.73288446	9.80E-02	1.29E-01	-6.377090728
Mir2137	0.152255807	7.355313432	0.846578674	4.07E-01	4.63E-01	-7.475633222
Ptpn2	0.152008219	8.672954971	1.676307164	1.09E-01	1.42E-01	-6.465569221
Hnrnph1	0.151902085	11.34317177	2.292127381	3.25E-02	4.72E-02	-5.394911245

1110001J03Rik	0.150348577	8.123282875	0.890097546	3.84E-01	4.39E-01	-7.437469911
Asprv1	0.149541226	7.089679277	0.685343838	5.01E-01	5.54E-01	-7.601306392
Cstb	0.149472869	9.342205419	1.526017189	1.42E-01	1.80E-01	-6.689558715
Camta2	0.146941022	8.880773249	2.196852079	3.96E-02	5.66E-02	-5.574769123
Taz	0.146790155	8.720949005	1.618928926	1.21E-01	1.55E-01	-6.553011594
Hnrnph3	0.146606469	9.920115551	1.940282582	6.61E-02	9.05E-02	-6.034568099
Smc3	0.146382258	9.351918591	2.056738579	5.26E-02	7.32E-02	-5.830515858
Tbl1xr1	0.145065601	7.870195122	1.783154209	8.93E-02	1.18E-01	-6.296645274
Mllt1	0.144520923	7.423920289	1.427931521	1.68E-01	2.10E-01	-6.826684833
Prkd3	0.14367209	7.348369237	1.450984111	1.62E-01	2.03E-01	-6.795121016
Parp3	0.143504573	7.370260213	1.588629426	1.27E-01	1.64E-01	-6.598235877
Kdm6a	0.142277282	9.730908967	1.907797346	7.04E-02	9.56E-02	-6.090031707
Ppil3	0.141816225	7.533747424	1.177177325	2.53E-01	3.03E-01	-7.142399511
Prr33	0.141152182	7.429084749	1.336576029	1.96E-01	2.40E-01	-6.947650924
Slc37a1	0.14106839	7.089819363	1.128485378	2.72E-01	3.23E-01	-7.197602129
Fam105a	0.140992462	8.736683624	0.978183091	3.39E-01	3.93E-01	-7.354835178
Tet2	0.140861043	8.745816868	1.38213124	1.82E-01	2.25E-01	-6.88815986
Srgn	0.140784095	11.24575118	1.851954487	7.84E-02	1.05E-01	-6.183833546
Psip1	0.140634946	8.807663079	1.867361943	7.61E-02	1.02E-01	-6.15815016
Neat1	0.13950208	10.1294555	1.328907921	1.98E-01	2.43E-01	-6.957500391
Trip10	0.139336038	6.932561915	0.916714251	3.70E-01	4.25E-01	-7.413256239
Cd37	0.139181919	9.087520565	1.068334416	2.98E-01	3.50E-01	-7.262940971
Hnrnpa3	0.138484635	8.217348395	1.181400614	2.51E-01	3.01E-01	-7.13751526
Scyl2	0.1372415	8.27569874	1.835688259	8.09E-02	1.08E-01	-6.210783271
Anxa4	0.137020775	9.016721537	1.585716649	1.28E-01	1.64E-01	-6.602548301
Mdm2	0.136194052	8.509881787	1.906345451	7.06E-02	9.58E-02	-6.092495351
Ddt	0.135575198	8.793858879	0.902358286	3.77E-01	4.33E-01	-7.426397981
C920009B18Rik	0.135298756	6.765265484	0.748963585	4.62E-01	5.17E-01	-7.55470749
Gfpt1	0.135227262	9.684916274	1.557052926	1.35E-01	1.72E-01	-6.644653899
Ppm1h	0.134968212	8.583667396	1.204194913	2.42E-01	2.91E-01	-7.110890524
Tmem234	0.13338259	10.09237042	1.935930422	6.67E-02	9.12E-02	-6.042036437
Gpr141	0.132686821	6.446440822	0.653855164	5.20E-01	5.73E-01	-7.622909427
Eif3c	0.132263922	10.22312128	2.554519172	1.86E-02	2.82E-02	-4.877188131
Cd74	0.13219575	12.57260271	2.510357396	2.05E-02	3.07E-02	-4.966462432
Slc6a6	0.131947391	10.29287552	1.966565347	6.28E-02	8.63E-02	-5.989221517
Lepre1	0.131866892	6.619966261	0.694758281	4.95E-01	5.48E-01	-7.594658984
Fam58b	0.129666165	8.779501144	0.674643232	5.07E-01	5.60E-01	-7.608756687
Arfrp1	0.129465274	8.5793341	1.268959677	2.19E-01	2.66E-01	-7.032845856
Mmp14	0.128280794	6.163449278	0.525144565	6.05E-01	6.53E-01	-7.701009293
Huwe1	0.126912158	9.96211981	1.810478083	8.48E-02	1.13E-01	-6.252212658
Mfng	0.124795948	7.005273864	0.762593295	4.54E-01	5.09E-01	-7.544214406
Arrdc3	0.123997559	8.247254313	0.978136496	3.39E-01	3.93E-01	-7.354880775
Dctn4	0.123890444	9.639681219	1.759912192	9.32E-02	1.23E-01	-6.334050124
Atf6	0.123858474	8.572314359	1.382729704	1.82E-01	2.24E-01	-6.887367269
Akt3	0.123106656	9.716809182	1.879924412	7.43E-02	1.00E-01	-6.137097424
2310045N01Rik	0.122374074	10.05715198	1.412417179	1.73E-01	2.15E-01	-6.847693814
Prnd	0.122098866	3.513295095	0.18644852	8.54E-01	8.76E-01	-7.826224484
Diap3	0.121874281	6.309115058	0.532453861	6.00E-01	6.49E-01	-7.697017096
Ptprc	0.121369828	11.0398556	1.593826534	1.26E-01	1.62E-01	-6.5905261
Stx6	0.121217673	7.876289015	1.157636536	2.60E-01	3.11E-01	-7.16479914
Flot1	0.11944844	7.623460251	0.804879402	4.30E-01	4.85E-01	-7.510524502
Rasgef1b	0.118952066	7.355784036	0.724875956	4.77E-01	5.31E-01	-7.572813061
Rap2c	0.118410926	10.32756104	1.625097258	1.19E-01	1.54E-01	-6.543723729
Suco	0.118360977	8.796939137	1.134894261	2.69E-01	3.20E-01	-7.19045363
Rnf19b	0.118175938	10.39380614	1.283485301	2.14E-01	2.60E-01	-7.014860832
Sbno2	0.118020673	9.043990924	1.425065375	1.69E-01	2.11E-01	-6.830580259
Plcl2	0.116465084	8.561360981	1.027976297	3.16E-01	3.68E-01	-7.304983043
Fubp1	0.115444614	9.131630988	1.720292747	1.00E-01	1.32E-01	-6.396973045
Ccr5	0.115351919	6.231595598	0.450900034	6.57E-01	7.03E-01	-7.738505302
Tdh	0.115276687	5.638546339	0.515763742	6.12E-01	6.60E-01	-7.706054175
Serp1	0.114496083	8.719306807	1.160986972	2.59E-01	3.09E-01	-7.160981874
LOC100502896	0.114444714	4.529357133	0.227233205	8.22E-01	8.51E-01	-7.817411456
Smap2	0.114243095	9.576306469	1.461993584	1.59E-01	1.99E-01	-6.779901448
Hipk1	0.113717486	9.412358854	2.054625252	5.28E-02	7.34E-02	-5.834289552
Srsf5	0.113197443	11.67269562	1.499013722	1.49E-01	1.88E-01	-6.728042402
Nadk	0.112212816	8.352258367	1.323687659	2.00E-01	2.45E-01	-6.964178365
Rtn1	0.1120263	7.328068351	1.221474949	2.36E-01	2.85E-01	-7.090412412
Sh2b2	0.111673839	6.485628486	0.651312286	5.22E-01	5.75E-01	-7.624611493
Snora52	0.111588904	8.259464978	0.682142268	5.03E-01	5.56E-01	-7.603547234
Scamp4	0.111479145	7.450321472	0.617341837	5.44E-01	5.95E-01	-7.646737803
Dhx9	0.111363627	9.51333576	1.682329233	1.08E-01	1.40E-01	-6.456257093
Slc30a5	0.111289473	7.303831987	0.716608573	4.82E-01	5.35E-01	-7.578897677
Fam117b	0.110665018	7.89527326	1.266698667	2.19E-01	2.66E-01	-7.035629614
Hmgn5	0.110491208	5.98068845	0.393982815	6.98E-01	7.41E-01	-7.763459799
Iffo2	0.109374355	7.344080261	1.130061634	2.71E-01	3.22E-01	-7.195847281
Irak4	0.108758447	8.816435277	1.155495838	2.61E-01	3.12E-01	-7.167233039

Gpr137b	0.108399623	7.307606651	0.67736993	5.06E-01	5.59E-01	-7.606868868
Xpa	0.108272314	8.084494867	0.987405581	3.35E-01	3.89E-01	-7.345771365
LOC102640635	0.107992029	3.58983004	0.201069996	8.43E-01	8.66E-01	-7.823263996
Glycam1	0.105303392	5.807860298	0.190263414	8.51E-01	8.73E-01	-7.825473525
Ccl5	0.105016028	11.56374644	0.828571275	4.17E-01	4.73E-01	-7.490902923
Mir3064	0.104974713	12.24381025	1.53246032	1.41E-01	1.79E-01	-6.680295366
Akirin1	0.104764993	8.24478555	0.530587731	6.01E-01	6.50E-01	-7.698041445
App	0.104425394	9.084715326	1.466757283	1.58E-01	1.98E-01	-6.773287122
Crybg3	0.104037416	7.146943958	1.327151711	1.99E-01	2.44E-01	-6.959749479
Apol7c	0.103984263	10.10859492	0.509241199	6.16E-01	6.64E-01	-7.709509682
Snord17	0.103275523	8.37538705	0.690978791	4.97E-01	5.50E-01	-7.597338033
Gm3086	0.102992712	7.652381086	0.730085124	4.74E-01	5.27E-01	-7.568945167
Clint1	0.1029285	10.23952167	1.751055235	9.48E-02	1.25E-01	-6.348208882
Ncoa2	0.101594896	9.6950241	1.025012222	3.17E-01	3.70E-01	-7.308013314
Bbs9	0.101158301	6.606649283	0.943518663	3.56E-01	4.11E-01	-7.388208131
Mpi	0.101139748	6.821104074	0.607012797	5.50E-01	6.01E-01	-7.653239223
Hsd17b4	0.100204676	8.317873347	0.842616389	4.09E-01	4.65E-01	-7.479019417
0610010K14Rik	0.099997726	7.984032003	0.668855221	5.11E-01	5.64E-01	-7.612739841
Lztfl1	0.099517076	6.991963645	0.534714634	5.99E-01	6.47E-01	-7.695771434
Tmem147	0.098659225	7.728351434	0.940990263	3.58E-01	4.12E-01	-7.390599184
Dnajc9	0.097086304	8.619504314	1.309632003	2.05E-01	2.50E-01	-6.982048611
Map2k1	0.097013343	8.594106169	0.876407082	3.91E-01	4.46E-01	-7.4496671
Stx4a	0.096640483	10.06102929	1.291244645	2.11E-01	2.57E-01	-7.00518208
Gspt1	0.09628749	8.182397338	0.646158771	5.25E-01	5.78E-01	-7.628041454
Dync1li1	0.095788198	9.0266832	1.418625292	1.71E-01	2.13E-01	-6.83930964
Mir1195	0.095079521	7.801985568	0.440062293	6.64E-01	7.10E-01	-7.743511467
Ino80d	0.094440701	9.534591535	1.554069283	1.35E-01	1.73E-01	-6.64900195
Exosc3	0.094252686	9.461928811	1.091225865	2.88E-01	3.39E-01	-7.238450546
Zfr	0.093267014	9.659222493	1.172472126	2.54E-01	3.04E-01	-7.147823086
Plekhg3	0.093101977	6.186037572	0.719387144	4.80E-01	5.34E-01	-7.576860113
Copz1	0.092394984	8.832379727	0.885317136	3.86E-01	4.41E-01	-7.441748812
Wnk1	0.091327668	10.63201125	1.111125044	2.79E-01	3.30E-01	-7.216785989
Ntng2	0.090969053	8.471673925	0.548369124	5.89E-01	6.38E-01	-7.688138898
1110065P20Rik	0.090287082	7.892623241	0.647970231	5.24E-01	5.77E-01	-7.626838804
Prm2	0.089405659	6.014910567	0.749365123	4.62E-01	5.17E-01	-7.554400918
S100a13	0.089208282	9.694482068	0.718605198	4.80E-01	5.34E-01	-7.577434283
Dctn2	0.088970126	8.807324526	0.983714397	3.37E-01	3.91E-01	-7.349408334
Cox16	0.088713728	8.499193384	0.638105522	5.30E-01	5.82E-01	-7.633348949
Gm3650	0.088572588	8.347979439	0.628862318	5.36E-01	5.87E-01	-7.639361795
Sec11c	0.088480957	10.45140109	1.433893287	1.67E-01	2.08E-01	-6.818561559
Zmym5	0.087889145	8.621768466	1.183313617	2.50E-01	3.00E-01	-7.135297838
Atp6v1a	0.087593609	8.576191383	1.220240963	2.36E-01	2.85E-01	-7.091883153
Rab23	0.08734217	6.864824453	0.646517816	5.25E-01	5.78E-01	-7.627803337
Apex1	0.087318948	7.938799624	0.640302293	5.29E-01	5.81E-01	-7.63190751
Itpr2	0.086823335	7.687540401	0.808988954	4.28E-01	4.82E-01	-7.507159242
Wdfy2	0.085761785	7.09754027	0.377550033	7.10E-01	7.51E-01	-7.770048647
Zcchc2	0.085201313	6.797453814	0.757942543	4.57E-01	5.12E-01	-7.547814991
Tma7	0.08448143	10.09237539	1.061491373	3.01E-01	3.53E-01	-7.270171832
Krcc1	0.084059944	9.406672555	1.043552509	3.09E-01	3.61E-01	-7.288929384
Dnmt3a	0.083833235	7.526398845	0.605161927	5.52E-01	6.02E-01	-7.654393039
Rab8b	0.083794491	10.29610929	0.981217906	3.38E-01	3.92E-01	-7.351861127
Herc4	0.083448225	8.999832874	1.056783756	3.03E-01	3.55E-01	-7.275122064
Dnpep	0.082927102	7.760857769	0.770942569	4.49E-01	5.04E-01	-7.537698239
Gatc	0.082758351	7.404293477	0.529993988	6.02E-01	6.50E-01	-7.698366627
Oaz2	0.082328923	8.457369975	0.483120366	6.34E-01	6.80E-01	-7.722917723
D930015E06Rik	0.081945337	10.40792802	1.023268197	3.18E-01	3.71E-01	-7.309792594
Zfyve1	0.081309788	7.44914547	0.947752933	3.54E-01	4.09E-01	-7.3841907
Mir674	0.080835136	6.774024287	0.422658042	6.77E-01	7.22E-01	-7.751300617
Gm19327	0.080751358	7.221612657	0.404329436	6.90E-01	7.34E-01	-7.759169415
Krt80	0.079923697	6.615562215	0.413226675	6.84E-01	7.28E-01	-7.755392517
Klf10	0.07954181	6.604197981	0.41454088	6.83E-01	7.27E-01	-7.754827779
Fxr1	0.079419491	9.666464583	1.140014962	2.67E-01	3.18E-01	-7.184716311
Rab12	0.079305157	7.327927656	0.746064243	4.64E-01	5.18E-01	-7.556916494
Arl6ip4	0.079043631	9.221707545	0.958832852	3.49E-01	4.03E-01	-7.373600242
Arl15	0.078033139	6.965870073	0.387056788	7.03E-01	7.45E-01	-7.766270568
1110012L19Rik	0.077458258	6.509551999	0.223073036	8.26E-01	8.54E-01	-7.818389645
Cox6b1	0.076955668	10.01576653	0.666660016	5.12E-01	5.65E-01	-7.614241928
Bcap31	0.076669696	9.746459529	0.815653224	4.24E-01	4.79E-01	-7.501667781
LOC102640273	0.07654009	4.717692076	0.280766328	7.82E-01	8.15E-01	-7.803221393
H2-T23	0.076053239	7.993352695	0.463164416	6.48E-01	6.94E-01	-7.732696163
Tmem106a	0.074317985	8.505505707	0.597584887	5.57E-01	6.07E-01	-7.659081029
Ccr2	0.074025005	8.945593659	0.429119557	6.72E-01	7.18E-01	-7.748444838
Tgs1	0.073878189	8.573119255	0.749251162	4.62E-01	5.17E-01	-7.554487943
H2-Ab1	0.073091385	12.02168764	0.969917564	3.43E-01	3.97E-01	-7.362892614
Gbp5	0.072452665	8.207441373	0.341838793	7.36E-01	7.75E-01	-7.783411968
Uap1l1	0.071798822	7.069188517	0.581593108	5.67E-01	6.16E-01	-7.668787892

Kansl3	0.07178744	8.04951119	1.03528123	3.13E-01	3.65E-01	-7.297481284
Stt3b	0.070776634	7.959908335	0.587491937	5.63E-01	6.13E-01	-7.665236991
Iqgap2	0.070691161	8.002293327	0.317870574	7.54E-01	7.90E-01	-7.791645175
Nabp1	0.06976651	10.5218103	0.612363709	5.47E-01	5.98E-01	-7.649884397
Ewsr1	0.069415588	9.050060143	0.818338556	4.23E-01	4.78E-01	-7.499443088
Reep5	0.068945227	9.660666667	0.688097086	4.99E-01	5.52E-01	-7.599371322
Cers2	0.068378002	8.70731225	0.789822654	4.39E-01	4.93E-01	-7.522716645
H2-Aa	0.067958372	12.15393585	1.072038085	2.96E-01	3.48E-01	-7.259010058
Clybl	0.06742219	5.839912425	0.331411816	7.44E-01	7.81E-01	-7.787066431
Arrb2	0.066437851	9.71203007	0.605263801	5.52E-01	6.02E-01	-7.65432962
Prdx6	0.066203736	7.166577851	0.41999746	6.79E-01	7.23E-01	-7.752464131
Calm2	0.065995605	11.71855629	1.054912939	3.04E-01	3.56E-01	-7.277083816
Sarnp	0.065896035	7.1880274	0.208626157	8.37E-01	8.61E-01	-7.821646846
Deb1	0.064547252	9.115016998	0.385284012	7.04E-01	7.46E-01	-7.766982112
Sft2d1	0.064304018	7.111629619	0.231531757	8.19E-01	8.48E-01	-7.816381839
Sgcb	0.064173069	6.129865845	0.235088551	8.16E-01	8.47E-01	-7.81551538
Serpinb10	0.064109766	5.677656661	0.232558657	8.18E-01	8.48E-01	-7.816133028
Stx7	0.063745476	8.92865665	0.854714334	4.03E-01	4.58E-01	-7.468634007
Basp1	0.063339101	9.214062707	0.938311897	3.59E-01	4.13E-01	-7.393125634
Olfr472	0.062945337	7.613361354	0.236634548	8.15E-01	8.46E-01	-7.815134669
Scamp2	0.061814993	9.84639656	0.93684419	3.60E-01	4.14E-01	-7.394507288
Tle3	0.061746602	9.386325261	0.644528835	5.26E-01	5.79E-01	-7.629120825
Gbp4	0.061568111	7.301715279	0.36106423	7.22E-01	7.63E-01	-7.776380517
Btg1	0.060642654	10.30754286	0.507944547	6.17E-01	6.64E-01	-7.710191511
Abr	0.059348622	8.422404304	0.718220235	4.81E-01	5.34E-01	-7.577716736
F630028O10Rik	0.059231894	6.378096678	0.201303628	8.42E-01	8.66E-01	-7.823214885
Uba7	0.059158831	9.028587374	0.798869966	4.33E-01	4.88E-01	-7.51541657
Zfp180	0.058658625	7.557829271	0.446310646	6.60E-01	7.06E-01	-7.740639813
Zbtb37	0.058540545	7.221477456	0.450111891	6.57E-01	7.03E-01	-7.738873388
Prkrir	0.058203249	8.96412226	0.536858784	5.97E-01	6.46E-01	-7.694585288
Gm14586	0.05772639	8.900308993	0.375916134	7.11E-01	7.52E-01	-7.770688645
Mcl1	0.05761815	10.76686569	0.76981542	4.50E-01	5.05E-01	-7.538581831
Nrbp1	0.05608707	10.30382564	0.936175472	3.60E-01	4.14E-01	-7.395136141
Pitpna	0.055553558	9.622120839	0.608462696	5.50E-01	6.00E-01	-7.652332995
Zfand3	0.05375914	9.095116067	0.557866037	5.83E-01	6.32E-01	-7.682720156
Apobec3	0.052651143	10.4048234	0.834820612	4.13E-01	4.69E-01	-7.485638438
Esyt1	0.052151826	8.290483069	0.596064221	5.58E-01	6.08E-01	-7.660015007
Asrgl1	0.052054961	7.115594574	0.318339997	7.53E-01	7.90E-01	-7.791489612
U2af1	0.051552305	9.449579774	0.421348139	6.78E-01	7.23E-01	-7.751874361
Xbp1	0.051228023	8.603376409	0.371733299	7.14E-01	7.55E-01	-7.772314583
Stx8	0.051135454	7.97793962	0.468562268	6.44E-01	6.91E-01	-7.730091034
Sc5d	0.050891101	7.739975008	0.397493321	6.95E-01	7.39E-01	-7.762016389
Spint2	0.050442815	8.820075567	0.510562515	6.15E-01	6.63E-01	-7.708813139
Cbx5	0.049745055	8.048774449	0.42184561	6.78E-01	7.22E-01	-7.751656672
Nedd9	0.049622255	8.27004941	0.633706671	5.33E-01	5.84E-01	-7.636220991
Aif1l	0.049262965	5.948663998	0.217384759	8.30E-01	8.57E-01	-7.819698029
Gtdc1	0.048338635	7.260635907	0.458029033	6.52E-01	6.98E-01	-7.735147175
Lyl1	0.047426195	5.370303578	0.265046264	7.94E-01	8.26E-01	-7.807696472
Tmem223	0.046614023	8.549819944	0.385459797	7.04E-01	7.46E-01	-7.766911701
Crot	0.046158942	7.472947051	0.461930523	6.49E-01	6.95E-01	-7.733287518
Trbj1-6	0.045771606	8.669095088	0.214894489	8.32E-01	8.59E-01	-7.820260239
Fbxl5	0.044006063	8.614672768	0.499930808	6.22E-01	6.69E-01	-7.714367814
Epsti1	0.043906272	10.99479522	0.348605897	7.31E-01	7.71E-01	-7.780980336
Scn2b	0.042494838	6.422007653	0.314891111	7.56E-01	7.92E-01	-7.79262725
Mrpl28	0.042393192	7.413976397	0.275799915	7.85E-01	8.19E-01	-7.804662849
Abracl	0.042327059	9.012256097	0.33536396	7.41E-01	7.79E-01	-7.785694453
Tpd52l2	0.041864138	8.35863488	0.489683196	6.30E-01	6.76E-01	-7.719613813
Mvd	0.041692321	6.639005543	0.365812541	7.18E-01	7.59E-01	-7.774585389
Mgea5	0.04155158	9.508741585	0.516370896	6.11E-01	6.59E-01	-7.705730337
Trim37	0.04090818	7.073019269	0.246861191	8.07E-01	8.40E-01	-7.812553798
Lcp2	0.039878352	9.742275422	0.251576227	8.04E-01	8.36E-01	-7.811327321
Zdhhc9	0.039654815	7.626780541	0.199533476	8.44E-01	8.67E-01	-7.823585569
Ankrd42	0.039486522	4.964971338	0.164036309	8.71E-01	8.91E-01	-7.830330076
Kxd1	0.03896392	9.223082549	0.351712179	7.29E-01	7.69E-01	-7.779848378
4930469K13Rik	0.038438098	5.976862406	0.183012956	8.57E-01	8.78E-01	-7.826887799
Itfg1	0.037724683	9.010074988	0.452963195	6.55E-01	7.01E-01	-7.737538755
Manbal	0.037259968	7.517942877	0.272412239	7.88E-01	8.21E-01	-7.805631441
Fbxo7	0.036899867	7.156550169	0.333745665	7.42E-01	7.80E-01	-7.786258186
Msantd4	0.03655274	7.534695055	0.330219195	7.45E-01	7.81E-01	-7.787477286
Adam17	0.035990113	8.865466685	0.462709743	6.48E-01	6.94E-01	-7.73291425
Ddx39b	0.03586157	9.646495447	0.332785648	7.43E-01	7.81E-01	-7.786591333
Gen1	0.035856166	6.162161115	0.190697556	8.51E-01	8.73E-01	-7.825387104
Gnl2	0.035554735	8.185067309	0.347105526	7.32E-01	7.72E-01	-7.781523531
Nat6	0.035132079	7.890550004	0.310882701	7.59E-01	7.95E-01	-7.793934028
Triobp	0.035054634	6.712151568	0.297997501	7.69E-01	8.04E-01	-7.79802234
Gm10051	0.03494019	11.40256086	0.35855365	7.24E-01	7.64E-01	-7.777320298

Sf3b1	0.03487482	11.08099994	0.669232608	5.11E-01	5.64E-01	-7.612481135
Cacnb2	0.034287775	5.886464418	0.122672589	9.04E-01	9.19E-01	-7.836531482
Apool	0.033672529	8.193196582	0.301236198	7.66E-01	8.02E-01	-7.797010878
H2-Ke6	0.033426473	8.553088376	0.307161349	7.62E-01	7.97E-01	-7.795132376
Daam1	0.032791044	7.58398934	0.394617573	6.97E-01	7.40E-01	-7.763199741
Tnip1	0.031639001	8.514109516	0.381824738	7.06E-01	7.48E-01	-7.768361298
Mdn1	0.031456043	6.951428273	0.201568368	8.42E-01	8.66E-01	-7.823159166
Mrpl9	0.030412911	7.668590327	0.225576275	8.24E-01	8.52E-01	-7.817803209
Psmd4	0.030368918	9.325038098	0.316190588	7.55E-01	7.91E-01	-7.79220005
Gna11	0.030284208	7.507706528	0.197264472	8.46E-01	8.68E-01	-7.824055947
Cd8a	0.029853885	8.739158364	0.158804552	8.75E-01	8.93E-01	-7.831213071
Ssr1	0.029828766	8.129016922	0.360792703	7.22E-01	7.63E-01	-7.77648247
Adamtsl4	0.029735079	5.892030618	0.209843358	8.36E-01	8.61E-01	-7.821380788
LOC102638448	0.028978855	11.93535434	0.476626175	6.39E-01	6.85E-01	-7.726144174
Amn1	0.028946045	7.730898699	0.232563117	8.18E-01	8.48E-01	-7.816131945
Ndufc2	0.028904902	10.34080047	0.292548272	7.73E-01	8.08E-01	-7.799699698
Cdk4	0.028189052	8.464676801	0.245263386	8.09E-01	8.40E-01	-7.812964187
Zranb1	0.028018031	8.689431545	0.331691859	7.43E-01	7.81E-01	-7.786969745
Gtpbp2	0.027748116	8.02549402	0.386846124	7.03E-01	7.45E-01	-7.766355292
Gm6115	0.027702938	9.02709995	0.162901004	8.72E-01	8.92E-01	-7.830524113
Nup98	0.027634519	9.509503916	0.42786094	6.73E-01	7.18E-01	-7.749004444
Parp1	0.027406546	7.974468963	0.244878695	8.09E-01	8.40E-01	-7.813062597
Peli1	0.026531527	9.674905003	0.286237249	7.78E-01	8.12E-01	-7.801603959
Tomm6	0.026306302	10.08375717	0.290546343	7.74E-01	8.09E-01	-7.800308212
Hist3h2a	0.026288933	8.255229841	0.177644186	8.61E-01	8.82E-01	-7.827899741
Lcp1	0.026239963	11.18046409	0.415510897	6.82E-01	7.27E-01	-7.754409815
Mrpl33	0.025691072	9.323604793	0.302487563	7.65E-01	8.01E-01	-7.796617168
Vdac1	0.024205828	9.240820141	0.26528446	7.93E-01	8.26E-01	-7.807630574
Il4ra	0.02354166	8.748551574	0.209370224	8.36E-01	8.61E-01	-7.82148439
Ptgs2os2	0.023012816	4.768199011	0.107538362	9.15E-01	9.30E-01	-7.838354022
Psma7	0.022903672	11.25333926	0.373764441	7.12E-01	7.53E-01	-7.771527286
LOC102631906	0.022798697	5.761202918	0.060132007	9.53E-01	9.62E-01	-7.84251317
Hcfc1	0.022646367	8.900663113	0.27096916	7.89E-01	8.22E-01	-7.806040431
Hist1h1c	0.022451575	8.548923635	0.102885927	9.19E-01	9.33E-01	-7.838866192
Sik2	0.021612402	8.169961277	0.217210145	8.30E-01	8.57E-01	-7.81973766
Rab22a	0.021549483	7.736631646	0.184899426	8.55E-01	8.77E-01	-7.826525094
Gm10375	0.021440227	4.043479659	0.035440789	9.72E-01	9.78E-01	-7.843748255
Bccip	0.021403521	8.906753681	0.253350852	8.03E-01	8.35E-01	-7.810859731
Ddit3	0.020458023	8.048832845	0.17078725	8.66E-01	8.87E-01	-7.829148501
Khnyn	0.020029451	7.609393393	0.221174375	8.27E-01	8.55E-01	-7.818830104
Mrps17	0.019580242	7.717197033	0.097897255	9.23E-01	9.36E-01	-7.83939024
Anapc5	0.017563245	9.669393313	0.215740179	8.31E-01	8.58E-01	-7.820070036
Vti1b	0.016710649	9.753453867	0.204657415	8.40E-01	8.64E-01	-7.822503633
Serpinb2	0.016674753	4.017203926	0.062311482	9.51E-01	9.60E-01	-7.842373507
Nsfl1c	0.015877701	9.625860581	0.213762896	8.33E-01	8.59E-01	-7.820513579
Taf12	0.015274887	8.797012875	0.145451902	8.86E-01	9.03E-01	-7.833337245
Lage3	0.015174618	9.563307155	0.085139339	9.33E-01	9.44E-01	-7.840612073
Cct5	0.015066608	10.7254308	0.243619342	8.10E-01	8.41E-01	-7.813383687
Ndufb10	0.014264822	10.47790021	0.144872385	8.86E-01	9.03E-01	-7.833425224
St8sia4	0.01372497	9.358675987	0.164743759	8.71E-01	8.91E-01	-7.830208484
Coro1a	0.013223443	10.25338072	0.140064269	8.90E-01	9.07E-01	-7.834141642
Snord14a	0.011886627	8.31333815	0.088215522	9.31E-01	9.42E-01	-7.840333036
Cotl1	0.011454803	8.606640306	0.058940263	9.54E-01	9.62E-01	-7.842587436
Ywhae	0.011396143	11.21763741	0.185615487	8.55E-01	8.76E-01	-7.826386449
Khk	0.011316784	7.033466061	0.04825878	9.62E-01	9.69E-01	-7.84318672
Rfc1	0.011165921	7.965233286	0.095663851	9.25E-01	9.37E-01	-7.839616423
Mical1	0.011141547	7.815553732	0.088275148	9.31E-01	9.42E-01	-7.84032753
Ythdf1	0.01018737	8.93689419	0.117827641	9.07E-01	9.22E-01	-7.837140978
Syncrip	0.010022735	10.37550024	0.114791708	9.10E-01	9.24E-01	-7.837510401
Dtymk	0.009516544	7.80674548	0.069502785	9.45E-01	9.55E-01	-7.84187743
Mt1	0.008525549	8.526046102	0.055286104	9.56E-01	9.64E-01	-7.842805888
Utp18	0.008000555	7.967755028	0.089130114	9.30E-01	9.42E-01	-7.840248166
Atm	0.007259244	6.9649563	0.059639386	9.53E-01	9.62E-01	-7.842544049
Usp3	0.006718112	8.932321997	0.098789914	9.22E-01	9.35E-01	-7.83929838
Oat	0.005788825	8.99249949	0.051584423	9.59E-01	9.67E-01	-7.843012935
Gm16039	0.005744205	7.039509195	0.036935419	9.71E-01	9.77E-01	-7.843691634
Npr1	0.005424214	5.169606469	0.022561888	9.82E-01	9.85E-01	-7.844139257
Eif4ebp2	0.004763901	9.611668832	0.055931564	9.56E-01	9.64E-01	-7.842768317
Lix1l	0.004500518	7.033023825	0.031629876	9.75E-01	9.80E-01	-7.84388204
Snora28	0.004074998	8.859041809	0.026375229	9.79E-01	9.83E-01	-7.844041578
Tep1	0.00399178	7.830993138	0.0316415	9.75E-01	9.80E-01	-7.843881655
H2-DMa	0.002943137	10.07050299	0.031001725	9.76E-01	9.80E-01	-7.843902633
Kpna4	0.002718417	9.061165381	0.038302121	9.70E-01	9.76E-01	-7.843637814
Ssbp4	0.002266793	6.861140855	0.013139561	9.90E-01	9.91E-01	-7.844315337
Rnf7	0.002133132	10.2088314	0.026232675	9.79E-01	9.83E-01	-7.844045504
Trim5	0.000773861	8.616767148	0.006311516	9.95E-01	9.96E-01	-7.844384857

Mdh2	0.000610772	10.42819064	0.009010831	9.93E-01	9.94E-01	-7.844363207
Rassf5	-0.000101037	8.662393684	-0.000854116	9.99E-01	9.99E-01	-7.844405326
Gabarap	-0.000327132	10.83932304	-0.005573819	9.96E-01	9.96E-01	-7.844389446
Sptssa	-0.000581482	8.63194741	-0.003969695	9.97E-01	9.97E-01	-7.844397459
Cope	-0.001440023	9.429892984	-0.013828617	9.89E-01	9.91E-01	-7.84430561
Pias1	-0.001707043	9.815255319	-0.023521546	9.81E-01	9.85E-01	-7.844116109
Uchl3	-0.002078769	8.201967752	-0.015290004	9.88E-01	9.90E-01	-7.844283336
Kif16b	-0.002992992	7.455063103	-0.024538942	9.81E-01	9.84E-01	-7.844090515
Sec1	-0.003265798	5.98696699	-0.02318617	9.82E-01	9.85E-01	-7.844124308
Mvp	-0.003695377	9.193361843	-0.043959214	9.65E-01	9.72E-01	-7.843394243
Inpp5j	-0.003728728	5.779583003	-0.029376871	9.77E-01	9.81E-01	-7.843953984
Fkbp3	-0.004328476	8.858164502	-0.044837515	9.65E-01	9.72E-01	-7.843353423
Tm9sf3	-0.005158704	9.350058861	-0.063467607	9.50E-01	9.60E-01	-7.842297404
Erdr1	-0.005539533	12.12042227	-0.034303602	9.73E-01	9.78E-01	-7.843789768
Kansl2	-0.005873296	6.794880941	-0.014653281	9.88E-01	9.90E-01	-7.844293315
Cops4	-0.006860761	8.248001318	-0.072027458	9.43E-01	9.53E-01	-7.841690439
Elovl7	-0.007354845	5.095507698	-0.038825182	9.69E-01	9.76E-01	-7.843616698
Manba	-0.007635612	6.975027848	-0.058395289	9.54E-01	9.62E-01	-7.842620902
Plekhb2	-0.007717452	8.491843308	-0.07698688	9.39E-01	9.50E-01	-7.841303704
Sirt1	-0.007748159	9.579620516	-0.078456561	9.38E-01	9.49E-01	-7.841184157
Fbrs	-0.010243469	9.848601574	-0.103458118	9.19E-01	9.32E-01	-7.838804422
Sbno1	-0.010323395	9.363457334	-0.114343402	9.10E-01	9.25E-01	-7.837564137
Slfn2	-0.011009152	9.681173407	-0.119724807	9.06E-01	9.21E-01	-7.836905235
Wdr3	-0.011231051	7.507355817	-0.128117574	8.99E-01	9.15E-01	-7.835817237
Hnrnpu	-0.011948253	10.63144227	-0.229178805	8.21E-01	8.50E-01	-7.816947811
Add3	-0.01196408	7.848449291	-0.101020461	9.21E-01	9.34E-01	-7.8390652
Pnp2	-0.01368215	5.203786928	-0.03754562	9.70E-01	9.76E-01	-7.843667846
Ctnnd1	-0.014355832	7.263918199	-0.095278098	9.25E-01	9.37E-01	-7.839654961
Baz2b	-0.014568837	9.542936428	-0.16202509	8.73E-01	8.92E-01	-7.830672899
Ints12	-0.014622754	8.692349326	-0.154384121	8.79E-01	8.97E-01	-7.831936882
LOC102639037	-0.014650835	3.418465429	-0.020531501	9.84E-01	9.86E-01	-7.844185056
Lbh	-0.014717302	10.97657183	-0.179851967	8.59E-01	8.80E-01	-7.827487239
Themis2	-0.015603401	9.674419917	-0.162163125	8.73E-01	8.92E-01	-7.830649505
A930002C04Rik	-0.016302953	5.246281794	-0.088254709	9.31E-01	9.42E-01	-7.840329418
Plaa	-0.016499104	9.253674364	-0.240328072	8.12E-01	8.43E-01	-7.814215068
Mfsd1	-0.016568726	9.362457941	-0.212465728	8.34E-01	8.60E-01	-7.82080235
Tnfaip8l2	-0.016960023	8.073018546	-0.137563757	8.92E-01	9.08E-01	-7.834504685
Dock10	-0.01784063	10.77414831	-0.258543814	7.99E-01	8.31E-01	-7.809472693
Zfp141	-0.018360459	7.572672253	-0.106441781	9.16E-01	9.30E-01	-7.838476779
Ier3	-0.019088436	8.03625432	-0.078473444	9.38E-01	9.49E-01	-7.84118277
Sec31a	-0.019410956	8.937880557	-0.246052692	8.08E-01	8.40E-01	-7.812761788
Spdl1	-0.019422023	7.63771356	-0.210558467	8.35E-01	8.61E-01	-7.82122376
Sugt1	-0.019946373	8.11675315	-0.17372612	8.64E-01	8.84E-01	-7.828619286
Dad1	-0.020293615	9.404591649	-0.234234987	8.17E-01	8.47E-01	-7.815724512
Cd68	-0.020799905	10.001137	-0.200423431	8.43E-01	8.67E-01	-7.823399613
Prkx	-0.020926463	8.38927261	-0.218369059	8.29E-01	8.57E-01	-7.819474033
Kctd9	-0.02094788	7.068143484	-0.16277442	8.72E-01	8.92E-01	-7.830545665
Haus1	-0.022072809	7.063965573	-0.120211182	9.05E-01	9.21E-01	-7.836844192
Arid1b	-0.022217908	9.019683648	-0.28018269	7.82E-01	8.16E-01	-7.803392112
Zfp935	-0.023286195	7.208469625	-0.118218545	9.07E-01	9.22E-01	-7.837092712
Clip1	-0.023676804	8.90853155	-0.245815081	8.08E-01	8.40E-01	-7.812822786
Lrrfip1	-0.023862795	8.595193726	-0.38642264	7.03E-01	7.45E-01	-7.766525468
Ipo7	-0.024212283	8.769932159	-0.28627153	7.78E-01	8.12E-01	-7.801593727
Thbs4	-0.024471709	5.005515343	-0.14750801	8.84E-01	9.02E-01	-7.833022272
Dnajc15	-0.025326947	9.526108933	-0.193518083	8.48E-01	8.71E-01	-7.824820866
Heatr9	-0.026750677	6.760031674	-0.163306366	8.72E-01	8.92E-01	-7.830454986
Tmem256	-0.027065778	8.360206486	-0.140022728	8.90E-01	9.07E-01	-7.834147727
2700029M09Rik	-0.027946957	8.698459774	-0.343618668	7.35E-01	7.74E-01	-7.78277697
Ccdc28b	-0.030625041	7.519328784	-0.388301385	7.02E-01	7.45E-01	-7.765769098
Ighv7-1	-0.031425086	5.317725269	-0.048295958	9.62E-01	9.69E-01	-7.843184841
Nop10	-0.032286387	9.228664565	-0.317230603	7.54E-01	7.91E-01	-7.791856892
Casp4	-0.034254006	7.747665533	-0.337953699	7.39E-01	7.78E-01	-7.784786707
Aftph	-0.034659607	9.902087221	-0.424889505	6.75E-01	7.20E-01	-7.750319196
Itpr1	-0.035346786	8.106807914	-0.333578138	7.42E-01	7.80E-01	-7.78631639
Tmem8	-0.036025867	9.059769091	-0.391219353	7.00E-01	7.43E-01	-7.764587176
Ubxn1	-0.036254329	10.04205814	-0.513127085	6.13E-01	6.61E-01	-7.707456183
Pabpc1	-0.036324298	12.41025517	-0.609246846	5.49E-01	6.00E-01	-7.65184201
Gm2a	-0.037206143	10.90741949	-0.496140165	6.25E-01	6.71E-01	-7.716320699
Tspan2	-0.037296135	6.426857442	-0.160462467	8.74E-01	8.93E-01	-7.830936344
Ccrl2	-0.038005222	8.37656918	-0.240892695	8.12E-01	8.43E-01	-7.814073242
Hspb11	-0.03821836	8.61421019	-0.341571291	7.36E-01	7.75E-01	-7.783507121
Aim1	-0.0385041	9.073155493	-0.515498641	6.12E-01	6.60E-01	-7.706195456
Ddost	-0.039153587	9.565829061	-0.404713968	6.90E-01	7.34E-01	-7.759007853
Supt6	-0.039755156	9.838257849	-0.57272008	5.73E-01	6.22E-01	-7.674063835
LOC102635469	-0.039858331	7.093872502	-0.150049232	8.82E-01	9.00E-01	-7.832626889
LOC102635953	-0.040228364	6.62897473	-0.132737245	8.96E-01	9.12E-01	-7.835186972

Hint2	-0.040665153	9.47131974	-0.331735527	7.43E-01	7.81E-01	-7.786954661
H2-Eb1	-0.041112792	11.71711714	-0.58532418	5.65E-01	6.14E-01	-7.666545938
Hpgds	-0.041292433	5.039068728	-0.207928554	8.37E-01	8.62E-01	-7.821798634
6530402F18Rik	-0.041393409	7.33185034	-0.208633529	8.37E-01	8.61E-01	-7.821645239
Gnb1	-0.041600687	10.4157008	-0.62559738	5.38E-01	5.89E-01	-7.64146551
Ttc37	-0.041747572	6.787021524	-0.358599347	7.24E-01	7.64E-01	-7.77730325
Psmc2	-0.042154776	8.756802746	-0.571945503	5.74E-01	6.22E-01	-7.674520674
Tmed10	-0.042777709	9.620965819	-0.285497054	7.78E-01	8.12E-01	-7.801824604
Plekhm3	-0.043224978	8.28831495	-0.395812574	6.96E-01	7.40E-01	-7.762709034
Snora47	-0.043289629	8.24086701	-0.198069509	8.45E-01	8.68E-01	-7.823889672
Rtn4	-0.043819012	7.966748875	-0.5066569	6.18E-01	6.65E-01	-7.710866928
Atp2b1	-0.044861143	9.525541273	-0.643938014	5.27E-01	5.79E-01	-7.62951143
Sqstm1	-0.046669995	9.503013141	-0.561907382	5.80E-01	6.29E-01	-7.680386867
Hnrnpd	-0.0475055	10.52192222	-0.638412197	5.30E-01	5.82E-01	-7.633148006
Ppp1r14a	-0.047882878	5.865865058	-0.232338317	8.19E-01	8.48E-01	-7.816186507
Foxj2	-0.04936175	9.117275917	-0.464605441	6.47E-01	6.93E-01	-7.732003583
Coq2	-0.049810281	7.404422024	-0.213063296	8.33E-01	8.59E-01	-7.820669539
Snora81	-0.049956447	10.62891577	-0.40854981	6.87E-01	7.31E-01	-7.757387941
Erich5	-0.050830568	5.646930566	-0.37564841	7.11E-01	7.52E-01	-7.770793252
Gltp	-0.051009995	10.48633967	-0.702705916	4.90E-01	5.43E-01	-7.588979921
Lman1	-0.051404794	8.068961671	-0.282733639	7.80E-01	8.14E-01	-7.802643339
Mir17	-0.051841316	6.110128731	-0.160090384	8.74E-01	8.93E-01	-7.830998699
Gm2614	-0.052119084	7.428677191	-0.229436677	8.21E-01	8.50E-01	-7.816886064
Hnrnpm	-0.05401546	10.78885268	-0.738311271	4.69E-01	5.23E-01	-7.562783581
Laptm5	-0.054199448	12.725684	-1.043640366	3.09E-01	3.61E-01	-7.288838218
Heatr1	-0.054784648	7.506136066	-0.625450457	5.39E-01	5.89E-01	-7.641559931
Mir505	-0.055251338	7.683116239	-0.199941972	8.43E-01	8.67E-01	-7.823500316
Csf1	-0.055530588	6.307309958	-0.222697122	8.26E-01	8.54E-01	-7.818477149
Abcd1	-0.055897332	8.96284987	-0.634684565	5.33E-01	5.84E-01	-7.635584169
Rad21	-0.05640353	10.09108726	-0.784877879	4.41E-01	4.96E-01	-7.526673398
Fdx1l	-0.056709041	4.351755823	-0.097191373	9.24E-01	9.36E-01	-7.83946229
Emg1	-0.056710205	8.994635374	-0.77108544	4.49E-01	5.04E-01	-7.537586153
Psmd2	-0.056812829	9.26655604	-0.58379234	5.66E-01	6.15E-01	-7.667468079
Psmd1	-0.057567679	9.012356677	-0.940417683	3.58E-01	4.12E-01	-7.391139843
Pik3r1	-0.057625675	9.733766561	-0.834013835	4.14E-01	4.70E-01	-7.486320151
Pigt	-0.057869612	8.614754473	-0.567814615	5.76E-01	6.25E-01	-7.67694692
Hrsp12	-0.058033376	6.419549095	-0.31288047	7.58E-01	7.93E-01	-7.79328481
Yif1b	-0.058123532	8.403902032	-0.595342315	5.58E-01	6.08E-01	-7.66045759
Aldoc	-0.05880474	5.907472239	-0.298533561	7.68E-01	8.03E-01	-7.797855674
Stx11	-0.059039765	6.705081303	-0.552213917	5.87E-01	6.36E-01	-7.68595602
Tspo	-0.059103339	9.315990539	-0.501083994	6.22E-01	6.68E-01	-7.713770831
Rp2h	-0.059353368	9.824543875	-0.713393988	4.84E-01	5.37E-01	-7.581245616
Uso1	-0.059420772	8.857169714	-0.593828033	5.59E-01	6.09E-01	-7.661384275
Nploc4	-0.059445632	8.331089262	-0.548574505	5.89E-01	6.38E-01	-7.688022667
Mbnl3	-0.059501458	7.314900856	-0.501631992	6.21E-01	6.68E-01	-7.713486673
Myd88	-0.059904975	7.644577263	-0.589139447	5.62E-01	6.12E-01	-7.664239053
Gdi2	-0.061492168	10.71312248	-0.93685766	3.60E-01	4.14E-01	-7.394494617
Rps5	-0.061769305	12.69296648	-1.205551845	2.42E-01	2.91E-01	-7.109291617
Gtpbp4	-0.061792535	9.94326197	-0.977830624	3.39E-01	3.93E-01	-7.355180044
Jag1	-0.062582323	7.21121173	-0.347333489	7.32E-01	7.72E-01	-7.781441149
Pabpc4	-0.063888125	9.212196579	-0.316644135	7.55E-01	7.91E-01	-7.792050537
Prrc2b	-0.065234336	8.649151081	-0.900767044	3.78E-01	4.33E-01	-7.427842843
Mrps16	-0.065880037	10.36034034	-0.797386443	4.34E-01	4.89E-01	-7.516618951
Dcaf7	-0.066594676	9.205852593	-0.634535776	5.33E-01	5.84E-01	-7.635681123
Haus8	-0.068023212	8.398956848	-0.500862493	6.22E-01	6.68E-01	-7.713885602
Fam49b	-0.06811421	9.838358785	-0.966406485	3.45E-01	3.99E-01	-7.366296417
F11r	-0.068835152	7.638311332	-0.374664259	7.12E-01	7.53E-01	-7.771177152
Gm10094	-0.068959777	10.60037925	-0.74549738	4.64E-01	5.18E-01	-7.557347438
Tmbim4	-0.06974047	10.22223503	-0.798037363	4.34E-01	4.88E-01	-7.516091645
Tdp1	-0.071042682	6.156678638	-0.283554097	7.80E-01	8.14E-01	-7.802401081
Gnb2l1	-0.071311677	11.8793544	-1.219072089	2.37E-01	2.85E-01	-7.093275104
Ttc27	-0.071391701	6.09660865	-0.498350094	6.24E-01	6.70E-01	-7.715183939
Tbk1	-0.071573867	9.01919196	-0.876529857	3.91E-01	4.46E-01	-7.449558496
Hspa9	-0.07206319	8.686977268	-1.121647341	2.75E-01	3.26E-01	-7.205189853
Ubap2	-0.072107288	8.716053896	-0.77596315	4.47E-01	5.02E-01	-7.533747699
1600010M07Rik	-0.072233101	7.735917806	-0.27899484	7.83E-01	8.16E-01	-7.80373848
Atp6v0e	-0.07257873	10.72179301	-0.847734624	4.06E-01	4.62E-01	-7.474642547
Sdhc	-0.073405917	8.447859503	-0.603548906	5.53E-01	6.03E-01	-7.655395809
Gm12159	-0.073470483	4.66302546	-0.270596895	7.89E-01	8.22E-01	-7.806145586
Mlf2	-0.074514505	8.867473113	-0.641797166	5.28E-01	5.80E-01	-7.630923909
Uqcrq	-0.07471623	9.983952704	-0.668443295	5.11E-01	5.64E-01	-7.613022065
Snord19	-0.07584504	5.567650199	-0.160048312	8.74E-01	8.93E-01	-7.831005741
Cbl	-0.076071482	9.583582249	-0.885718687	3.86E-01	4.41E-01	-7.441390209
Rpl4	-0.076750437	11.97248645	-1.472663006	1.56E-01	1.96E-01	-6.765062903
Hexa	-0.076969224	8.796534293	-1.042402425	3.09E-01	3.62E-01	-7.290122153
Chmp2a	-0.077665136	9.714701293	-1.041497521	3.10E-01	3.62E-01	-7.291059811

Mettl7a1	-0.077722412	5.920989144	-0.328547997	7.46E-01	7.83E-01	-7.788050544
Mir3097	-0.077966788	7.739232883	-0.441230518	6.64E-01	7.09E-01	-7.742977586
Tnfrsf10b	-0.079164113	5.558712991	-0.356421582	7.25E-01	7.65E-01	-7.778113306
Otud5	-0.08051307	8.958872794	-1.134536857	2.70E-01	3.20E-01	-7.190853222
Eef2k	-0.081236406	5.934377866	-0.435579201	6.68E-01	7.13E-01	-7.74554735
Wars	-0.082085945	8.463867807	-0.902156828	3.77E-01	4.33E-01	-7.426581036
Uqcc2	-0.082158103	8.623146973	-0.511759186	6.14E-01	6.62E-01	-7.708180786
Kcmf1	-0.082750038	9.93287145	-1.074646463	2.95E-01	3.47E-01	-7.256234339
Nlrc5	-0.082757607	8.490616903	-0.840728455	4.10E-01	4.66E-01	-7.480627643
Nol8	-0.083730952	7.636093619	-0.888587931	3.84E-01	4.40E-01	-7.438823459
Rabgef1	-0.083750568	8.215492019	-0.703242198	4.90E-01	5.43E-01	-7.588594497
H2-Oa	-0.084174177	9.86496398	-1.002620838	3.28E-01	3.81E-01	-7.330648952
Erbb2ip	-0.0842903	9.901118671	-1.011452556	3.24E-01	3.77E-01	-7.321774937
Alg3	-0.084830432	7.24022662	-0.568496727	5.76E-01	6.25E-01	-7.676547461
H2afy	-0.085254695	10.06786441	-1.102253182	2.83E-01	3.34E-01	-7.226487908
Gas5	-0.085570107	10.01203658	-1.248303701	2.26E-01	2.74E-01	-7.058119362
Pnpla2	-0.08587572	8.020311527	-0.92283507	3.67E-01	4.22E-01	-7.407594983
Yipf1	-0.08727825	7.944713194	-0.818774269	4.22E-01	4.78E-01	-7.499081471
Neurl3	-0.088141744	8.253296936	-0.44269139	6.63E-01	7.08E-01	-7.742308012
Hmga1-rs1	-0.088321978	9.655638175	-0.476505665	6.39E-01	6.85E-01	-7.726203642
Sdcbp	-0.088446712	9.312618541	-1.25900663	2.22E-01	2.70E-01	-7.045068232
Nlrp3	-0.089343303	5.979250927	-0.332306083	7.43E-01	7.81E-01	-7.786757397
Clns1a	-0.090530354	8.521679665	-0.716062821	4.82E-01	5.35E-01	-7.579297003
Josd1	-0.090618673	8.998543595	-0.776999906	4.46E-01	5.01E-01	-7.532928894
Fam98a	-0.090679774	7.141046949	-0.943905392	3.56E-01	4.11E-01	-7.387841892
Gm10012	-0.09141074	10.70033721	-0.766740702	4.52E-01	5.06E-01	-7.540985951
Dnaja2	-0.091435211	9.572760953	-1.133932644	2.70E-01	3.21E-01	-7.191528502
Mfsd5	-0.091555346	8.610843334	-0.967062572	3.45E-01	3.99E-01	-7.365661232
5031414D18Rik	-0.091782051	7.956665477	-0.573425328	5.73E-01	6.22E-01	-7.673647364
Phxr4	-0.092004403	7.23784469	-0.523874532	6.06E-01	6.54E-01	-7.701697483
Ctage5	-0.092204667	9.334316154	-1.105102837	2.82E-01	3.33E-01	-7.223379165
Slc25a4	-0.092552036	9.249083912	-0.730092863	4.74E-01	5.27E-01	-7.568939401
Ogt	-0.092615125	9.621394033	-0.874690794	3.92E-01	4.47E-01	-7.451183814
LOC102632661	-0.092961901	2.581011768	-0.21654014	8.31E-01	8.58E-01	-7.819889435
Ppip5k2	-0.093379656	7.948055121	-0.94965455	3.53E-01	4.08E-01	-7.382381101
Thrap3	-0.093425758	9.223363338	-0.581276401	5.67E-01	6.16E-01	-7.668977559
Arhgap30	-0.093771474	10.07460151	-1.507165784	1.47E-01	1.86E-01	-6.716482549
Lrch1	-0.094390135	9.643352394	-1.075269247	2.95E-01	3.47E-01	-7.255570708
Tmem179b	-0.094583367	9.398774252	-1.293744006	2.10E-01	2.56E-01	-7.002053895
Gm5424	-0.094625407	7.773352487	-0.833936764	4.14E-01	4.70E-01	-7.486385243
Rpl8	-0.09558367	11.34827519	-1.478897636	1.54E-01	1.94E-01	-6.756351639
Catsperg1	-0.09585913	6.01083675	-0.21387233	8.33E-01	8.59E-01	-7.820489137
Itgb5	-0.096100045	6.119361564	-0.599383867	5.55E-01	6.06E-01	-7.657973146
Gm561	-0.096671141	7.488479867	-0.552489571	5.87E-01	6.36E-01	-7.685798949
Sdf2	-0.097350507	8.922772906	-1.217383085	2.37E-01	2.86E-01	-7.09528441
Csrp1	-0.097579039	10.04090543	-0.890328245	3.84E-01	4.39E-01	-7.437262874
Ibtk	-0.097786052	7.614767306	-0.864117846	3.97E-01	4.53E-01	-7.460466387
Spcs3	-0.097875935	9.383002655	-1.337053864	1.96E-01	2.40E-01	-6.947035581
Ift20	-0.097976369	8.572077564	-0.937254526	3.59E-01	4.14E-01	-7.394121211
Arhgap25	-0.098372687	8.000823728	-0.80908502	4.28E-01	4.82E-01	-7.507080383
Dek	-0.098681625	10.45619108	-1.360023342	1.88E-01	2.32E-01	-6.917238791
Tbcb	-0.099202883	10.13971948	-1.444971538	1.63E-01	2.05E-01	-6.803393274
Ndufc1	-0.09945151	9.677172367	-0.907265466	3.75E-01	4.30E-01	-7.421927385
Aspscr1	-0.099758467	7.989442454	-1.082089716	2.92E-01	3.44E-01	-7.248280432
Nol11	-0.099762307	7.123120373	-0.594930963	5.58E-01	6.08E-01	-7.660709548
Fcho1	-0.099823261	7.121717319	-0.744299745	4.65E-01	5.19E-01	-7.55825689
Timm10b	-0.100169329	8.601342211	-0.717112623	4.81E-01	5.35E-01	-7.578528607
Nelfa	-0.100169751	8.747676154	-1.212647341	2.39E-01	2.88E-01	-7.100905371
Tgfb1	-0.100229715	9.447383532	-1.02960496	3.15E-01	3.68E-01	-7.303314647
Mir18	-0.100582284	5.60338481	-0.334763109	7.41E-01	7.80E-01	-7.785904075
Cd44	-0.101745523	8.384105976	-0.926706735	3.65E-01	4.19E-01	-7.403996103
Itpk1	-0.102300774	6.727091043	-0.704265667	4.89E-01	5.42E-01	-7.587858156
Klhl18	-0.102502967	7.236594628	-0.635155174	5.32E-01	5.84E-01	-7.635277362
Sipa1	-0.102538573	7.657427119	-1.299642663	2.08E-01	2.54E-01	-6.994650805
Pnn	-0.102569642	9.030712343	-1.166471784	2.57E-01	3.07E-01	-7.154711963
Ace2	-0.102841337	5.596206375	-0.730243904	4.73E-01	5.27E-01	-7.568826857
Eif3i	-0.103577557	10.12997966	-1.663015296	1.11E-01	1.45E-01	-6.486032931
Raph1	-0.10409597	7.024343745	-1.094685479	2.86E-01	3.38E-01	-7.234709014
Il18bp	-0.104315719	7.578878356	-1.353808674	1.90E-01	2.34E-01	-6.925342597
Sae1	-0.104373726	8.079304943	-1.172410265	2.54E-01	3.04E-01	-7.147894266
Ncl	-0.106604945	10.46013762	-1.557862437	1.34E-01	1.72E-01	-6.643473067
Heca	-0.107241823	8.539183651	-1.255522004	2.23E-01	2.71E-01	-7.049327875
Psmc6	-0.107400943	9.389233189	-1.561989642	1.33E-01	1.71E-01	-6.637445191
Nudt8	-0.107638606	7.557877291	-0.812399653	4.26E-01	4.80E-01	-7.504354053
Ttyh2	-0.10764735	6.335678113	-0.772602154	4.49E-01	5.04E-01	-7.53639504
Il10ra	-0.108370121	9.539337693	-1.243504573	2.28E-01	2.76E-01	-7.06394032

Arc	-0.10980992	6.947228554	-0.718615048	4.80E-01	5.34E-01	-7.577427054
Il10rb	-0.110140424	8.656377767	-1.352094278	1.91E-01	2.35E-01	-6.927572674
Uqcr11	-0.110793517	9.758105032	-0.868662856	3.95E-01	4.50E-01	-7.456488927
AI504432	-0.110803076	7.064546321	-0.637747064	5.31E-01	5.82E-01	-7.633583704
Snord49b	-0.1110005	8.573580917	-0.689152583	4.98E-01	5.52E-01	-7.59862752
Traf5	-0.111021601	7.728058392	-0.817427817	4.23E-01	4.78E-01	-7.500198368
Idnk	-0.112086693	9.334638783	-1.23018925	2.32E-01	2.81E-01	-7.079989604
Ppp3ca	-0.112365978	8.802794324	-1.478112791	1.54E-01	1.94E-01	-6.757449892
Trim59	-0.112576347	7.628116091	-0.491765296	6.28E-01	6.74E-01	-7.718556526
Ninj1	-0.112645371	7.141991122	-0.708702862	4.86E-01	5.39E-01	-7.584653989
Psmc5	-0.112903345	10.19100178	-1.325111124	2.00E-01	2.44E-01	-6.962359607
Mbnl1	-0.11298297	10.67613772	-1.660652831	1.12E-01	1.45E-01	-6.4896571
Csk	-0.113244389	8.554567902	-1.208451302	2.41E-01	2.89E-01	-7.105869874
LOC102636087	-0.113337732	8.950627442	-1.079541795	2.93E-01	3.45E-01	-7.251008675
Mef2a	-0.113478846	9.717840895	-1.713193518	1.02E-01	1.33E-01	-6.408134872
Idh3g	-0.113545966	9.883362579	-1.515103535	1.45E-01	1.84E-01	-6.705178347
Anp32b	-0.113547894	9.530335772	-0.917018336	3.70E-01	4.25E-01	-7.412975805
Calm1	-0.11449295	11.49740579	-1.654550592	1.13E-01	1.47E-01	-6.49900009
Crip1	-0.114891432	12.09455551	-1.472761551	1.56E-01	1.96E-01	-6.764925444
Hypk	-0.11557796	10.13367301	-1.119291162	2.76E-01	3.27E-01	-7.207794899
Snapc3	-0.115840479	8.713171147	-1.905979199	7.07E-02	9.59E-02	-6.093116617
Stxbp3a	-0.116079896	9.524176453	-1.074590847	2.95E-01	3.47E-01	-7.256293586
Timm8b	-0.116511256	9.250817445	-1.971008654	6.23E-02	8.57E-02	-5.98151402
Mrpl52	-0.117369722	10.20092411	-1.237780299	2.30E-01	2.78E-01	-7.070858171
Vim	-0.117692588	12.08088521	-1.662611153	1.12E-01	1.45E-01	-6.48665319
Sfmbt1	-0.11885585	7.972640786	-1.153735116	2.62E-01	3.12E-01	-7.169231954
Glod4	-0.119575491	8.287775406	-1.427228436	1.68E-01	2.10E-01	-6.827641002
Srsf1	-0.119968837	8.999450196	-1.038757538	3.11E-01	3.63E-01	-7.2938945
Myl6	-0.119969578	7.568858227	-0.588538307	5.63E-01	6.12E-01	-7.664603491
Ndufb6	-0.120381672	9.80983814	-1.300011529	2.08E-01	2.54E-01	-6.994186912
Lpin1	-0.120517071	6.722122094	-0.674348192	5.08E-01	5.61E-01	-7.60896052
Slc35c1	-0.121064082	7.324697813	-0.91908294	3.69E-01	4.24E-01	-7.411069512
Ythdf2	-0.121265128	8.653364993	-1.481171804	1.54E-01	1.93E-01	-6.753166682
Dpy19l1	-0.121341581	9.071452102	-1.281585291	2.14E-01	2.61E-01	-7.01722327
Nmt1	-0.121606879	9.512182525	-1.582028666	1.29E-01	1.65E-01	-6.607999547
Cd22	-0.122334652	6.406835851	-0.752619606	4.60E-01	5.15E-01	-7.551910408
Cfdp1	-0.122685355	9.206246495	-1.528770381	1.42E-01	1.80E-01	-6.685604227
Snord22	-0.122784662	8.552201335	-0.772367811	4.49E-01	5.04E-01	-7.53657922
Lrpprc	-0.1228275	7.295646962	-1.255045586	2.23E-01	2.71E-01	-7.049909465
Dnajc10	-0.122963919	9.1294144	-1.446289555	1.63E-01	2.04E-01	-6.801582308
Mfap3	-0.124302012	8.033133253	-1.150624273	2.63E-01	3.14E-01	-7.172757087
Nsmce1	-0.124666771	8.50857599	-1.377852791	1.83E-01	2.26E-01	-6.893817825
Jun	-0.127230541	9.482496013	-1.10161014	2.83E-01	3.34E-01	-7.227188429
Arhgap6	-0.128062663	6.041200694	-0.829033726	4.17E-01	4.73E-01	-7.490514618
Mif4gd	-0.131615331	7.642641722	-1.14525951	2.65E-01	3.16E-01	-7.178816643
Tmem55b	-0.13175423	8.659261819	-1.365171012	1.87E-01	2.31E-01	-6.910502864
Zmynd15	-0.131960658	8.678656716	-0.71092503	4.85E-01	5.38E-01	-7.583042125
Mpp5	-0.132578417	7.734066744	-1.289515432	2.11E-01	2.58E-01	-7.007343341
Jtb	-0.133688374	8.079071846	-1.045859265	3.08E-01	3.61E-01	-7.286533447
Canx	-0.133827833	10.65018817	-1.969972655	6.24E-02	8.58E-02	-5.983312158
Srsf7	-0.134019095	10.59160666	-1.363187363	1.88E-01	2.31E-01	-6.913101061
Asap1	-0.134408037	10.38024037	-1.522649576	1.43E-01	1.81E-01	-6.694387987
Actb	-0.134468655	13.26639215	-1.493413888	1.50E-01	1.90E-01	-6.73595394
Ccdc69	-0.134483965	5.983116015	-0.622167324	5.41E-01	5.91E-01	-7.643664261
Il15ra	-0.134728918	8.848225173	-0.821923493	4.21E-01	4.76E-01	-7.496462431
Itgax	-0.134866023	9.097775623	-1.131066877	2.71E-01	3.22E-01	-7.194727013
Trim34a	-0.134975383	8.614398784	-1.21969594	2.36E-01	2.85E-01	-7.092532335
Rbfa	-0.13603279	7.665539823	-1.147072666	2.64E-01	3.15E-01	-7.176771451
Chek2	-0.136059531	6.405861289	-0.757904255	4.57E-01	5.12E-01	-7.547844546
Tuba4a	-0.136172907	9.091270957	-1.385843483	1.81E-01	2.23E-01	-6.883238866
Slc33a1	-0.136185238	8.501209182	-1.219418235	2.36E-01	2.85E-01	-7.092863017
Rpl36a	-0.136879276	6.104537279	-0.520865158	6.08E-01	6.56E-01	-7.703321687
Nbeal1	-0.137123715	7.41568612	-1.434380156	1.66E-01	2.08E-01	-6.817896946
Casp6	-0.13727002	7.616400616	-0.737982917	4.69E-01	5.23E-01	-7.563030781
Ndufa2	-0.137506955	10.14272079	-1.438965114	1.65E-01	2.06E-01	-6.811629079
Sel1l	-0.138205432	9.282306354	-1.636630937	1.17E-01	1.51E-01	-6.52628395
Ndufs6	-0.138255173	10.93200461	-1.410538426	1.73E-01	2.16E-01	-6.850225139
Mir5125	-0.138434189	9.498614116	-0.710562778	4.85E-01	5.38E-01	-7.583305215
Creg1	-0.138491073	9.204069351	-1.785959073	8.88E-02	1.18E-01	-6.292106879
Ufd1l	-0.140600772	9.415744276	-1.497369704	1.49E-01	1.89E-01	-6.730367565
Sh3bp4	-0.141286255	7.035105826	-1.160980879	2.59E-01	3.09E-01	-7.160988825
Rplp1	-0.141327678	13.17483293	-2.355417579	2.85E-02	4.16E-02	-5.272933254
Cpsf2	-0.141603794	8.289097167	-1.545602968	1.37E-01	1.75E-01	-6.661303968
Smdt1	-0.14177556	10.4821889	-1.753372247	9.44E-02	1.25E-01	-6.344510005
Hdac2	-0.141871579	8.924002399	-2.082072727	5.00E-02	7.00E-02	-5.78507944
Gm12216	-0.142553007	6.310287734	-0.547101504	5.90E-01	6.39E-01	-7.688855341

Bola2	-0.14386481	10.38127783	-1.220953664	2.36E-01	2.85E-01	-7.091033869
Kdelr2	-0.14492555	10.16988095	-1.963813298	6.32E-02	8.68E-02	-5.993989343
Trim44	-0.145137647	8.629090791	-1.888586222	7.31E-02	9.90E-02	-6.122523475
Sod1	-0.145555085	10.95626094	-0.825101407	4.19E-01	4.74E-01	-7.493809986
Heatr3	-0.145791339	5.791643094	-0.634285752	5.33E-01	5.84E-01	-7.635843997
Cops2	-0.14628814	9.162666668	-2.260446412	3.47E-02	5.01E-02	-5.455230011
Nop56	-0.146505288	8.628420096	-2.151488158	4.34E-02	6.16E-02	-5.658745341
Prss30	-0.14658393	6.017274107	-0.979482056	3.39E-01	3.93E-01	-7.353563249
Acat1	-0.14816466	7.251654517	-0.885840988	3.86E-01	4.41E-01	-7.441280959
Fkbp15	-0.148177744	8.587625386	-2.146847474	4.39E-02	6.20E-02	-5.667273781
Tgfbr2	-0.148356804	6.889182062	-0.815844313	4.24E-01	4.79E-01	-7.501509698
St6gal1	-0.148896482	8.589884546	-1.402909621	1.76E-01	2.18E-01	-6.860475215
Thyn1	-0.149411106	7.329347585	-1.389634581	1.79E-01	2.22E-01	-6.878202045
Atp5k	-0.149636348	11.68008413	-1.932230903	6.72E-02	9.18E-02	-6.048375711
1810058I24Rik	-0.150436479	7.869946433	-1.575859089	1.30E-01	1.67E-01	-6.617096621
Prps1l3	-0.151114618	6.940503265	-0.539281188	5.95E-01	6.44E-01	-7.693239658
Cmtm6	-0.151147945	10.03588072	-2.163870737	4.24E-02	6.01E-02	-5.635932268
Eif3k	-0.15145889	10.47880132	-2.712536407	1.32E-02	2.06E-02	-4.551350951
Snord43	-0.152179166	6.895678452	-0.752942626	4.60E-01	5.15E-01	-7.551662658
Clcf1	-0.152306261	6.39142433	-0.720500669	4.79E-01	5.33E-01	-7.576041444
Snora23	-0.152574558	8.952696054	-1.167048114	2.57E-01	3.07E-01	-7.154051633
Dnajc1	-0.153034548	8.245227644	-1.717597744	1.01E-01	1.32E-01	-6.401214362
H2afz	-0.15309493	11.14183119	-1.344563326	1.93E-01	2.37E-01	-6.937340853
Poli	-0.153757741	7.122754953	-1.076411067	2.94E-01	3.46E-01	-7.254353106
Srsf10	-0.154731386	8.439143221	-1.176798245	2.53E-01	3.03E-01	-7.142837171
Mtmr1	-0.155819129	8.654608545	-2.189820253	4.02E-02	5.73E-02	-5.587857872
Atp5a1	-0.156276872	10.95232479	-2.570142223	1.80E-02	2.73E-02	-4.845411838
Lmnb1	-0.156295352	8.33251024	-1.600556502	1.25E-01	1.60E-01	-6.580513182
2610507B11Rik	-0.156432349	8.988008729	-2.377803597	2.72E-02	3.99E-02	-5.229329884
Mir15a	-0.156646551	8.154027754	-0.947472166	3.54E-01	4.09E-01	-7.3844576
Ltv1	-0.156771762	7.799079234	-1.185356957	2.49E-01	2.99E-01	-7.132925881
Tkt	-0.157721141	8.508079739	-1.321584606	2.01E-01	2.46E-01	-6.966862415
Gbp9	-0.157918883	9.836111383	-1.361756251	1.88E-01	2.32E-01	-6.914973584
Smim4	-0.158180434	7.683069322	-1.117256794	2.77E-01	3.28E-01	-7.210040242
Slirp	-0.158476207	9.649862897	-1.13784153	2.68E-01	3.19E-01	-7.187154242
Tmem170b	-0.158900214	6.901466507	-0.979869403	3.39E-01	3.93E-01	-7.353183666
Cox5a	-0.15942027	10.50995658	-2.171416701	4.17E-02	5.94E-02	-5.621989542
Idh3a	-0.159649251	5.973003123	-0.661956984	5.15E-01	5.68E-01	-7.617444091
Tmem156	-0.15986407	6.498661568	-0.826934214	4.18E-01	4.73E-01	-7.492275879
Magt1	-0.160074278	9.137929854	-2.090430236	4.91E-02	6.90E-02	-5.770010706
Ndufb8	-0.160784718	10.15583138	-1.571360247	1.31E-01	1.68E-01	-6.623712597
Ptp4a2	-0.161196257	10.32012911	-2.070190977	5.12E-02	7.15E-02	-5.806434584
Msi2	-0.161490737	8.694893584	-1.718969288	1.01E-01	1.32E-01	-6.399056488
Rsbn1l	-0.161721312	8.173656681	-2.020453453	5.65E-02	7.82E-02	-5.894951408
Scarna2	-0.161865861	7.022199427	-0.395902066	6.96E-01	7.40E-01	-7.762672226
Mbtd1	-0.162458299	8.499975782	-1.582438367	1.29E-01	1.65E-01	-6.607394455
LOC102634854	-0.162891267	8.15094599	-0.824582605	4.19E-01	4.75E-01	-7.494243659
Nphs1os	-0.164748268	4.450234987	-0.601789686	5.54E-01	6.04E-01	-7.656486521
Naip6	-0.165979557	7.555375722	-1.229693039	2.33E-01	2.81E-01	-7.080584819
Cd80	-0.166181472	8.832254191	-1.255705246	2.23E-01	2.71E-01	-7.049104129
Fkbp2	-0.166558587	9.680905063	-1.427697355	1.68E-01	2.10E-01	-6.827003333
Ndufa9	-0.166878741	8.730402779	-2.35161822	2.87E-02	4.19E-02	-5.280310121
Smc1a	-0.167020702	9.502584845	-2.279162102	3.34E-02	4.83E-02	-5.419657027
Lpar5	-0.167729429	7.521454346	-1.100969072	2.84E-01	3.35E-01	-7.227886437
Snord2	-0.170952487	10.22732222	-2.010857496	5.76E-02	7.96E-02	-5.911863994
LOC102638110	-0.171457056	2.791966099	-0.34551012	7.33E-01	7.73E-01	-7.782098592
Alg5	-0.171536905	8.902079487	-1.769921036	9.15E-02	1.21E-01	-6.317986516
E2f4	-0.171634246	8.31906059	-1.798725326	8.67E-02	1.15E-01	-6.2713847
Fem1c	-0.172260437	8.978487983	-1.891345077	7.27E-02	9.85E-02	-6.117871639
Glud1	-0.172790183	10.39313346	-2.318925833	3.08E-02	4.47E-02	-5.343500331
Micall1	-0.172965356	7.261187956	-1.561103048	1.34E-01	1.71E-01	-6.638741142
Olfr506	-0.173028994	2.22471143	-0.44803119	6.59E-01	7.04E-01	-7.739842102
AW549877	-0.173113467	8.216257921	-2.003473401	5.84E-02	8.07E-02	-5.92484141
Nucks1	-0.173936402	8.753800926	-2.239582961	3.63E-02	5.21E-02	-5.49467659
Myo9b	-0.174466152	9.257832393	-1.81732681	8.37E-02	1.12E-01	-6.2409987
Bcl2a1b	-0.174556197	11.29044787	-1.748340522	9.53E-02	1.26E-01	-6.352538039
Mif	-0.17635569	10.93337379	-1.508704237	1.47E-01	1.85E-01	-6.714295343
Strip2	-0.176356022	8.744283296	-0.883462507	3.87E-01	4.42E-01	-7.443403125
Wipf2	-0.176372295	7.972504004	-1.509664973	1.46E-01	1.85E-01	-6.712928565
Tifab	-0.176931645	8.001564787	-1.450944941	1.62E-01	2.03E-01	-6.795174996
Aim2	-0.177032705	8.790212108	-1.633495587	1.18E-01	1.52E-01	-6.531034242
Hprt	-0.177779385	8.745739891	-1.809841242	8.49E-02	1.13E-01	-6.253253847
Aimp1	-0.17792948	9.067290857	-1.922133046	6.85E-02	9.35E-02	-6.06563596
Atp5b	-0.17903676	10.94094313	-2.905229041	8.57E-03	1.39E-02	-4.141786516
Mri1	-0.179056083	6.586380692	-1.133706249	2.70E-01	3.21E-01	-7.191781443
Agap1	-0.179069839	7.730620302	-1.582088977	1.29E-01	1.65E-01	-6.607910481

Dennd1c	-0.179366715	8.407239784	-1.483931943	1.53E-01	1.93E-01	-6.749295819
Cse1l	-0.179919866	8.834863432	-2.351696043	2.87E-02	4.19E-02	-5.280159088
Atp5e	-0.180184942	11.82696374	-2.991633899	7.04E-03	1.17E-02	-3.954271822
Naa50	-0.180711576	9.873600921	-2.770451163	1.16E-02	1.83E-02	-4.429584964
Naa38	-0.182182054	9.453930121	-1.838072616	8.05E-02	1.08E-01	-6.206843559
Rpl36al	-0.182491235	9.20908672	-1.674549637	1.09E-01	1.42E-01	-6.468282162
Gpatch4	-0.182654686	6.599658701	-1.157491329	2.60E-01	3.11E-01	-7.16496436
Kdm2b	-0.182838824	7.73830249	-2.003075782	5.85E-02	8.07E-02	-5.925539307
Bcl2l1	-0.183269882	6.717639828	-1.177578938	2.52E-01	3.03E-01	-7.141935702
Srsf6	-0.18361089	8.820887935	-2.605574251	1.67E-02	2.54E-02	-4.772978668
Arpc2	-0.183936365	11.58376074	-3.396700634	2.77E-03	4.97E-03	-3.049295565
H13	-0.184270453	9.101509641	-2.440353393	2.38E-02	3.53E-02	-5.106265576
Snx17	-0.184339851	8.61848905	-2.559442692	1.84E-02	2.79E-02	-4.867184805
Eif3l	-0.184817587	9.860297056	-2.489446168	2.14E-02	3.20E-02	-5.008447048
Was	-0.184848564	7.520316934	-0.996615075	3.30E-01	3.84E-01	-7.336643154
Tcof1	-0.185570253	7.385726371	-1.877189073	7.47E-02	1.01E-01	-6.14168994
Twf2	-0.187118288	8.735840362	-1.506728315	1.47E-01	1.86E-01	-6.71710417
Polr1e	-0.189503056	6.046992013	-1.458067151	1.60E-01	2.00E-01	-6.785340104
Dtd1	-0.190397442	6.241813837	-1.273553533	2.17E-01	2.64E-01	-7.027176829
Plekha1	-0.1912445	9.010960055	-2.648783872	1.52E-02	2.33E-02	-4.683973233
Rpn2	-0.191454782	8.796089204	-2.058226311	5.24E-02	7.30E-02	-5.827857734
Tmem181c-ps	-0.191761825	6.267274548	-0.643785855	5.27E-01	5.79E-01	-7.62961197
Ssrp1	-0.191773963	7.813491905	-1.53754284	1.39E-01	1.77E-01	-6.672966287
Rhno1	-0.191865738	7.377953292	-1.014875831	3.22E-01	3.75E-01	-7.318316316
Ndufb5	-0.192296212	9.39358325	-2.354150477	2.86E-02	4.17E-02	-5.275394231
Pik3cd	-0.192671033	8.015576485	-0.822765669	4.20E-01	4.75E-01	-7.495760442
Eml6	-0.192902171	7.435132559	-1.841256113	8.00E-02	1.07E-01	-6.201577692
Psmb10	-0.193326126	10.34594769	-2.268406027	3.42E-02	4.93E-02	-5.440122641
3830406C13Rik	-0.193338313	7.781705907	-1.910001239	7.01E-02	9.52E-02	-6.086289533
Icam1	-0.193447957	9.822749306	-1.655795998	1.13E-01	1.47E-01	-6.497095416
Tmem128	-0.19360345	8.453703552	-1.529990433	1.41E-01	1.79E-01	-6.683850017
Lrrc42	-0.19400337	7.571615542	-1.227787683	2.33E-01	2.82E-01	-7.082868406
Cdk12	-0.194009511	9.35971897	-1.883781794	7.38E-02	9.97E-02	-6.130613018
Abhd16b	-0.194525068	5.309345571	-0.732886152	4.72E-01	5.26E-01	-7.566854485
Srsf9	-0.194945272	9.549030561	-1.885341596	7.35E-02	9.95E-02	-6.127988265
Gm21188	-0.195716937	6.920595	-0.766187667	4.52E-01	5.07E-01	-7.541417404
Nphs1	-0.195723205	5.009044016	-0.94797263	3.54E-01	4.09E-01	-7.383981804
2810008D09Rik	-0.195734767	7.884707161	-1.511097611	1.46E-01	1.85E-01	-6.710889149
9330175E14Rik	-0.19591079	6.071710989	-0.643963564	5.27E-01	5.79E-01	-7.629494545
Tapbp	-0.196055014	10.05171265	-2.928076372	8.14E-03	1.33E-02	-4.092421772
Timm9	-0.196290809	5.374326775	-0.987762175	3.35E-01	3.89E-01	-7.345419353
Park7	-0.196496521	9.845097238	-2.555889829	1.86E-02	2.81E-02	-4.874404311
Vcp	-0.19795844	9.59307091	-1.843847923	7.96E-02	1.07E-01	-6.197285716
Lmo4	-0.198039966	8.462543328	-1.743377642	9.62E-02	1.27E-01	-6.360439469
Selplg	-0.198055533	11.69175708	-2.675222304	1.43E-02	2.21E-02	-4.629159132
Psmb4	-0.198158222	10.6951349	-3.128230586	5.16E-03	8.74E-03	-3.653522888
Dbi	-0.19819315	9.875933835	-2.064581084	5.18E-02	7.23E-02	-5.816489451
Map2k6	-0.198453695	6.291518186	-0.871408943	3.94E-01	4.49E-01	-7.454076356
Lsg1	-0.198691182	8.054626699	-1.986644285	6.04E-02	8.32E-02	-5.954297809
Max	-0.198970769	9.810969631	-1.827540153	8.21E-02	1.10E-01	-6.224218746
2410006H16Rik	-0.199301925	11.29789839	-2.951908482	7.71E-03	1.27E-02	-4.040760104
Psma6	-0.19946976	10.58601345	-2.492075884	2.13E-02	3.19E-02	-5.003177554
Ndrg3	-0.199898881	8.633111794	-1.883905767	7.37E-02	9.97E-02	-6.130404459
Ndufb7	-0.200058603	10.66961468	-2.592307351	1.71E-02	2.61E-02	-4.800159039
Sik3	-0.200204986	7.287797889	-1.844869585	7.95E-02	1.07E-01	-6.19559268
Rapgef1	-0.202114654	8.279125804	-2.08439305	4.97E-02	6.98E-02	-5.780899799
Slc8a2	-0.202275578	3.63607074	-0.873547747	3.92E-01	4.48E-01	-7.452192413
Scarna3b	-0.202289418	8.377060878	-0.982025659	3.37E-01	3.92E-01	-7.351068134
Ssu72	-0.20294538	10.26194479	-2.408255052	2.55E-02	3.76E-02	-5.169641413
LOC102639286	-0.203119437	8.646948929	-1.40399506	1.75E-01	2.18E-01	-6.859019616
Apba1	-0.205518002	5.694321765	-1.39665219	1.77E-01	2.20E-01	-6.868848443
Ksr1	-0.205586039	5.740345123	-1.082704239	2.91E-01	3.43E-01	-7.247621559
Ube2l3	-0.206178301	9.562109471	-1.607220694	1.23E-01	1.59E-01	-6.570565766
Pmvk	-0.206548464	8.403665703	-1.56106379	1.34E-01	1.71E-01	-6.638798513
Ska2	-0.20750696	6.698848109	-0.942720138	3.57E-01	4.11E-01	-7.388963915
Sema4a	-0.208182319	9.022452447	-2.525330827	1.98E-02	2.98E-02	-4.936284916
Spg11	-0.208578372	7.919803895	-2.074231507	5.08E-02	7.10E-02	-5.799181482
Anp32e	-0.210369935	7.902049104	-1.940047158	6.62E-02	9.06E-02	-6.034972386
Rps27l	-0.210434433	8.634541595	-2.210539571	3.85E-02	5.51E-02	-5.54921732
Sec24d	-0.212712973	7.538310944	-1.872850486	7.53E-02	1.02E-01	-6.148964526
Morc3	-0.214176665	8.39523936	-2.400070285	2.59E-02	3.82E-02	-5.185726607
Stt3a	-0.214211455	9.738726591	-3.269507799	3.72E-03	6.49E-03	-3.3375267
Crcp	-0.215224865	8.317228019	-2.586876569	1.74E-02	2.64E-02	-4.811264972
Tm9sf2	-0.215232363	9.336732249	-2.696762137	1.36E-02	2.12E-02	-4.584306681
Gns	-0.215446099	9.523041536	-3.014327762	6.69E-03	1.11E-02	-3.904659345
Csrp2bp	-0.216138671	7.346657348	-1.788333972	8.84E-02	1.17E-01	-6.288260108

Kars	-0.216187487	9.035525158	-2.925762633	8.18E-03	1.33E-02	-4.097428197
Bcl2l11	-0.21631633	7.351244766	-1.970218682	6.24E-02	8.58E-02	-5.982885199
Rab42	-0.216627582	4.640975364	-0.748030202	4.63E-01	5.17E-01	-7.555419519
Metap1d	-0.217353948	6.729889315	-0.850270783	4.05E-01	4.61E-01	-7.472464585
Cops6	-0.217963494	9.62590851	-1.821888089	8.30E-02	1.11E-01	-6.233513165
Mir187	-0.217975665	6.313741944	-1.104389874	2.82E-01	3.33E-01	-7.224157619
Gadd45a	-0.218382963	6.844908055	-1.715823175	1.01E-01	1.32E-01	-6.404004405
Rsl1d1	-0.219747633	9.146633681	-3.014585774	6.69E-03	1.11E-02	-3.904094449
Parp14	-0.220697012	8.65691992	-2.433678268	2.41E-02	3.57E-02	-5.119483445
Heatr5a	-0.220826699	7.427991262	-2.634556011	1.56E-02	2.40E-02	-4.71336082
Noc3l	-0.22144554	6.362993053	-1.722956588	9.99E-02	1.31E-01	-6.392775878
Hnrnpab	-0.221676133	10.99523167	-3.40030932	2.75E-03	4.93E-03	-3.041071251
Cry1	-0.222182709	6.304864079	-1.038638217	3.11E-01	3.63E-01	-7.294017792
Mir3082	-0.222435098	8.530332818	-0.962202622	3.47E-01	4.01E-01	-7.370357004
Ddx10	-0.222493581	6.686066207	-1.347302946	1.92E-01	2.37E-01	-6.933792662
Eif4g1	-0.223202682	9.280453241	-3.228737009	4.09E-03	7.08E-03	-3.429194868
Scly	-0.223736855	7.215342432	-2.055735341	5.27E-02	7.33E-02	-5.832307625
Psmd14	-0.224465043	9.610959104	-3.306367572	3.42E-03	6.00E-03	-3.254340134
Rragc	-0.224758488	8.41384413	-2.171026259	4.18E-02	5.94E-02	-5.62271171
Tubgcp2	-0.224789591	7.832134398	-2.141887974	4.43E-02	6.26E-02	-5.676375215
Fam103a1	-0.225114841	7.201749249	-1.109749078	2.80E-01	3.31E-01	-7.218295214
LOC102632277	-0.225550731	4.932849546	-0.637818573	5.31E-01	5.82E-01	-7.633536882
Erp44	-0.225664201	9.34174214	-2.651748171	1.51E-02	2.32E-02	-4.67784068
N4bp1	-0.226505856	8.261647606	-2.593927513	1.71E-02	2.60E-02	-4.796843521
LOC102634949	-0.22672221	5.623895931	-0.686638994	5.00E-01	5.53E-01	-7.600397038
Clic1	-0.227222118	10.45592137	-2.236016966	3.65E-02	5.25E-02	-5.501396609
Hspa4	-0.227235366	9.650037234	-3.525972899	2.05E-03	3.77E-03	-2.75322737
Prelid1	-0.227328109	9.648274455	-2.141961038	4.43E-02	6.26E-02	-5.676241228
Adcy7	-0.227829866	7.651342018	-1.456475048	1.60E-01	2.01E-01	-6.787542003
Cluh	-0.228372007	6.697306366	-1.288553627	2.12E-01	2.58E-01	-7.008544391
Sec13	-0.228380527	9.409369633	-3.422917997	2.61E-03	4.71E-03	-2.989490118
Psma2	-0.228739172	9.536831707	-2.520928599	2.00E-02	3.01E-02	-4.94516703
St14	-0.228807424	6.100646941	-1.518074286	1.44E-01	1.83E-01	-6.700935471
Hist1h2bn	-0.229431853	10.43283997	-1.480709279	1.54E-01	1.94E-01	-6.753814765
Cat	-0.230089879	10.46107331	-3.432669047	2.55E-03	4.62E-03	-2.967214362
Tbc1d8	-0.230773138	10.30723254	-2.455110064	2.31E-02	3.43E-02	-5.076974251
Chst15	-0.231066629	7.941271286	-2.32554368	3.03E-02	4.42E-02	-5.330750462
Napsa	-0.231176025	9.74460215	-1.913075726	6.97E-02	9.48E-02	-6.08106406
Nckap1l	-0.231509243	10.57264742	-4.022330797	6.34E-04	1.28E-03	-1.595191191
Cep350	-0.231663995	9.30341734	-3.380529298	2.88E-03	5.14E-03	-3.08612027
Pcdhga6	-0.232175199	3.52908117	-0.746629634	4.64E-01	5.18E-01	-7.556486361
Ndufa4	-0.232342062	10.81530508	-3.483789928	2.26E-03	4.14E-03	-2.850155101
Pcdha5	-0.232393694	3.174678429	-0.81684028	4.23E-01	4.78E-01	-7.500685197
Ssr2	-0.232760557	9.606735776	-3.511209858	2.12E-03	3.89E-03	-2.787183104
Smarcc1	-0.232915723	8.736918209	-3.17196431	4.66E-03	7.98E-03	-3.556208284
Shmt2	-0.233608246	6.958254088	-1.203894906	2.42E-01	2.91E-01	-7.111243819
Fam96b	-0.233806225	9.343175687	-2.535139505	1.94E-02	2.93E-02	-4.916465219
Esam	-0.234286488	5.533618529	-1.292489243	2.10E-01	2.57E-01	-7.003624991
Pnpla7	-0.234360006	6.349281468	-2.290975491	3.26E-02	4.72E-02	-5.397113142
Ube2l6	-0.235127791	8.339166109	-2.19487127	3.98E-02	5.68E-02	-5.578458755
Rps20	-0.235715857	10.61235518	-1.807622211	8.53E-02	1.13E-01	-6.256879729
Mir378b	-0.23579413	7.092873005	-1.54417887	1.38E-01	1.75E-01	-6.663368033
Diap2	-0.235965401	7.889690124	-2.596756029	1.70E-02	2.59E-02	-4.791052698
Ndst2	-0.236165809	7.854364181	-1.856193873	7.78E-02	1.04E-01	-6.176781847
Pcna	-0.237005867	10.98498825	-2.981478099	7.21E-03	1.19E-02	-3.976426331
Actr3	-0.237224063	11.83035114	-3.996735815	6.74E-04	1.36E-03	-1.655504389
Anxa7	-0.237646134	8.622096383	-3.172080401	4.66E-03	7.98E-03	-3.555949341
Pfdn2	-0.238390956	8.299237971	-1.941345876	6.60E-02	9.04E-02	-6.032741718
Rasip1	-0.238530556	6.153926783	-1.88653014	7.34E-02	9.93E-02	-6.125987219
Sppl2a	-0.239184212	9.974177723	-3.53080918	2.02E-03	3.73E-03	-2.742096034
Vps51	-0.239240239	7.211910306	-2.326437738	3.03E-02	4.41E-02	-5.329026353
Mrto4	-0.239972799	7.105110534	-0.876167694	3.91E-01	4.46E-01	-7.449878817
Hsp90b1	-0.24055729	11.2856302	-3.419059305	2.63E-03	4.75E-03	-2.998300307
Set	-0.2407687	7.539909817	-1.987756469	6.03E-02	8.30E-02	-5.952356316
Mir29a	-0.241096917	4.240694836	-0.58658573	5.64E-01	6.13E-01	-7.665784751
Tmed5	-0.241191353	7.639673687	-2.47173515	2.23E-02	3.32E-02	-5.043858714
Fxyd5	-0.241594407	10.92075312	-3.090473073	5.62E-03	9.45E-03	-3.737152514
Txndc5	-0.241738204	7.398265358	-1.553458247	1.36E-01	1.73E-01	-6.649891599
Map4k1	-0.242643183	8.889682674	-2.874592406	9.18E-03	1.48E-02	-4.207726174
Gm5908	-0.244749622	9.116132479	-1.603202598	1.24E-01	1.60E-01	-6.576567292
Psmb8	-0.24508171	11.25174576	-3.383603313	2.86E-03	5.11E-03	-3.079124088
Pyhin1	-0.245108929	9.294258255	-2.160244938	4.27E-02	6.05E-02	-5.642620818
Pi4k2b	-0.245225154	6.39569669	-1.297877838	2.09E-01	2.55E-01	-6.996868741
Maml2	-0.245255724	7.171548022	-1.483683194	1.53E-01	1.93E-01	-6.749644907
Grcc10	-0.245520105	10.45373718	-3.269768406	3.72E-03	6.49E-03	-3.336939578
Atp5o	-0.245721247	9.748730843	-2.121855823	4.61E-02	6.50E-02	-5.713000629

Ube2q1	-0.245871772	9.378778177	-3.344478286	3.13E-03	5.56E-03	-3.168031902
Plek	-0.246025108	10.00054358	-2.06771152	5.14E-02	7.18E-02	-5.810880836
Fbxw11	-0.246395528	8.57744409	-2.99218448	7.04E-03	1.17E-02	-3.953069902
Mapre2	-0.246482293	8.412625988	-3.470428495	2.33E-03	4.26E-03	-2.880794744
Myl12b	-0.24697961	11.09616542	-3.375187474	2.91E-03	5.20E-03	-3.09827346
Slc25a12	-0.247250851	8.382688905	-3.203519748	4.34E-03	7.45E-03	-3.485704688
Mcm7	-0.247441817	6.914199869	-1.500380281	1.49E-01	1.88E-01	-6.726108093
Gnl3	-0.247462055	8.009938235	-2.037242611	5.47E-02	7.59E-02	-5.865231723
Psmd7	-0.248072404	9.638375983	-3.311310448	3.38E-03	5.96E-03	-3.243163009
Psmd11	-0.248612984	9.163201297	-2.79107831	1.11E-02	1.76E-02	-4.385931359
Oaz1	-0.249095497	9.50504057	-1.744257444	9.60E-02	1.26E-01	-6.359039947
Pa2g4	-0.2493137	10.35086929	-2.054958004	5.28E-02	7.34E-02	-5.833695538
Wdr74	-0.250469015	7.6881137	-2.369538418	2.77E-02	4.06E-02	-5.245456219
Cd40	-0.250715098	8.786299272	-2.451978651	2.32E-02	3.45E-02	-5.083198059
Immt	-0.250830239	8.491685294	-2.566328483	1.81E-02	2.75E-02	-4.853177961
Ube2z	-0.25086959	8.545115985	-2.607333449	1.66E-02	2.54E-02	-4.769369279
Atg3	-0.251568453	10.36379329	-4.931783176	7.39E-05	1.79E-04	0.559946566
Brix1	-0.251689512	8.053104098	-2.054378496	5.28E-02	7.34E-02	-5.83473001
Casp2	-0.25263548	7.485189283	-1.010390699	3.24E-01	3.77E-01	-7.322845607
Hsh2d	-0.253335157	8.006630526	-1.791390064	8.79E-02	1.17E-01	-6.283304469
Katna1	-0.253656667	9.570122243	-1.88767789	7.32E-02	9.91E-02	-6.124054009
Rps11	-0.253975406	8.36167238	-1.45375099	1.61E-01	2.02E-01	-6.791304869
Klhdc10	-0.254743042	7.212074353	-1.852335564	7.83E-02	1.05E-01	-6.183200142
Vgll4	-0.255302196	8.185339691	-2.623785439	1.60E-02	2.46E-02	-4.735555204
Prpsap1	-0.255636083	8.071750591	-2.688199793	1.39E-02	2.16E-02	-4.602156769
Pcbp2	-0.256814223	10.97950462	-3.038877206	6.33E-03	1.06E-02	-3.85082728
Prmt7	-0.256989293	6.513351759	-1.688769915	1.06E-01	1.39E-01	-6.44626964
Creb3	-0.257876638	8.945544673	-2.7493653	1.21E-02	1.91E-02	-4.47405621
Atp1b3	-0.257889532	9.196900345	-2.892814538	8.81E-03	1.43E-02	-4.168541985
Larp7	-0.25790658	7.61286599	-2.491483865	2.13E-02	3.19E-02	-5.004364117
Mdh1	-0.257912228	9.74781267	-3.137608042	5.05E-03	8.56E-03	-3.632696416
BC051537	-0.2588413	7.018358814	-2.028056974	5.57E-02	7.72E-02	-5.881512192
Dyrk3	-0.258968285	6.053546435	-1.507050613	1.47E-01	1.86E-01	-6.716646215
LOC102640958	-0.259779691	7.538712674	-1.143951764	2.66E-01	3.16E-01	-7.180289977
Lsm7	-0.260735155	9.223519015	-1.420489466	1.70E-01	2.12E-01	-6.836786136
Naa15	-0.261505158	7.949360251	-2.722554641	1.29E-02	2.02E-02	-4.530373556
Slc20a1	-0.26215673	8.732522574	-2.694695126	1.37E-02	2.13E-02	-4.588618308
Ccdc90b	-0.262202105	7.933726226	-3.920693056	8.07E-04	1.59E-03	-1.834399912
Bag1	-0.26252732	10.25254322	-3.991448397	6.82E-04	1.37E-03	-1.667957983
Nasp	-0.262634525	8.464926462	-2.545777716	1.90E-02	2.87E-02	-4.89492388
Mcur1	-0.262866495	7.874381227	-2.212301236	3.84E-02	5.50E-02	-5.545921531
Glul	-0.26327372	8.690119744	-3.123158666	5.22E-03	8.83E-03	-3.664777937
Trp53	-0.263920318	9.398256355	-3.226961707	4.11E-03	7.10E-03	-3.43317795
Samhd1	-0.264224448	11.46015402	-4.188040903	4.28E-04	8.94E-04	-1.203717724
Kat2a	-0.264270717	7.175666915	-2.837953668	9.96E-03	1.60E-02	-4.286190556
Dcaf12	-0.264477294	8.464310464	-2.263176061	3.45E-02	4.99E-02	-5.450052728
Actr1b	-0.264879867	7.17051086	-1.66443808	1.11E-01	1.45E-01	-6.483848394
C1qbp	-0.265265275	8.622356557	-2.720928727	1.29E-02	2.03E-02	-4.533780569
Idh2	-0.266278482	8.522337486	-2.188568607	4.03E-02	5.75E-02	-5.590184898
Esp6	-0.26636358	2.35624332	-0.770959585	4.49E-01	5.04E-01	-7.537684891
Capn2	-0.267065486	8.962497866	-3.224320103	4.13E-03	7.13E-03	-3.439103345
Ier5	-0.267505711	9.458613193	-2.97349269	7.34E-03	1.21E-02	-3.993825228
Gng10	-0.267567295	9.579819918	-2.183708283	4.07E-02	5.80E-02	-5.599213223
Mvb12b	-0.267691333	6.531700651	-1.660022478	1.12E-01	1.46E-01	-6.490623436
Incenp	-0.267691984	6.942759663	-2.775950708	1.14E-02	1.81E-02	-4.417960557
Wdr36	-0.268005955	7.500522966	-2.246938791	3.57E-02	5.14E-02	-5.480794185
Cdk2ap2	-0.268491702	8.198655346	-2.257196336	3.50E-02	5.04E-02	-5.461389471
Syce2	-0.269004985	7.435500125	-2.116099272	4.67E-02	6.57E-02	-5.723484751
2810428I15Rik	-0.269284411	8.847852183	-3.271883295	3.70E-03	6.46E-03	-3.332174378
Slc7a5	-0.269350112	8.415955372	-2.20888124	3.86E-02	5.53E-02	-5.552318309
Vav3	-0.269898132	7.245556563	-1.64425416	1.15E-01	1.49E-01	-6.514704967
Serpinb9	-0.270061362	8.023507824	-1.387688889	1.80E-01	2.23E-01	-6.880788499
Snord82	-0.270649403	7.530620779	-1.143319354	2.66E-01	3.17E-01	-7.181001931
Tmbim1	-0.270679393	7.518770895	-1.827114806	8.22E-02	1.10E-01	-6.224918923
Kdm4a	-0.270758841	8.217902532	-3.51966127	2.08E-03	3.82E-03	-2.767748758
Rcc2	-0.272281746	7.189731297	-2.138696656	4.46E-02	6.30E-02	-5.682224692
Zdhhc13	-0.272303285	7.863244192	-2.65609239	1.49E-02	2.30E-02	-4.668847269
Grap2	-0.272875935	7.763087432	-1.487771435	1.52E-01	1.92E-01	-6.743901587
Sh3bp2	-0.273095515	7.021325877	-2.423467356	2.47E-02	3.65E-02	-5.13966397
Plcb2	-0.27334373	8.560539739	-1.336067622	1.96E-01	2.41E-01	-6.948305432
Sirt2	-0.273704061	8.828188085	-3.348717638	3.10E-03	5.50E-03	-3.158412954
Eif4g3	-0.273973597	8.471687234	-1.832880545	8.13E-02	1.09E-01	-6.215417789
Ints7	-0.274588413	7.460114385	-2.871385164	9.24E-03	1.49E-02	-4.214611996
Utp14a	-0.274665877	7.538871215	-2.647435582	1.52E-02	2.34E-02	-4.686761467
Atp6v1d	-0.274745607	9.889857474	-4.47119814	2.19E-04	4.85E-04	-0.532301703
Hsd17b10	-0.274753576	8.388166425	-1.795318302	8.73E-02	1.16E-01	-6.276925518

Scai	-0.275507683	7.448338072	-3.297272179	3.49E-03	6.11E-03	-3.274893813
Tcrg-C2	-0.275591756	5.821994021	-0.40895648	6.87E-01	7.31E-01	-7.757215319
Elmo1	-0.275872046	9.561459895	-3.769746469	1.15E-03	2.21E-03	-2.187960115
Rere	-0.276121796	8.18003709	-2.3938179	2.63E-02	3.87E-02	-5.197993404
Nolc1	-0.276139209	8.129528095	-2.262622176	3.46E-02	4.99E-02	-5.451103577
Sppl3	-0.276610057	8.711815843	-3.325847248	3.27E-03	5.78E-03	-3.210262202
Utp20	-0.276631805	6.347473314	-2.678299999	1.42E-02	2.20E-02	-4.62276105
Nfkbib	-0.276879989	9.077501474	-2.765837732	1.17E-02	1.85E-02	-4.439328267
Usp47	-0.277856598	9.226235512	-4.456551825	2.26E-04	5.01E-04	-0.567068835
Psme1	-0.278572457	11.00546229	-3.637957433	1.57E-03	2.95E-03	-2.494564472
Spc25	-0.279767733	6.421271441	-1.816954648	8.38E-02	1.12E-01	-6.241608857
Mtif3	-0.280000206	7.55442292	-2.22082512	3.77E-02	5.41E-02	-5.529951925
Hint1	-0.280473263	10.34433811	-2.996094886	6.97E-03	1.16E-02	-3.944530963
Mlh1	-0.280706002	7.328373391	-1.63751872	1.17E-01	1.51E-01	-6.524937617
Dapk1	-0.281210251	6.611717922	-1.367371077	1.86E-01	2.30E-01	-6.907617518
Aldh2	-0.281316571	6.913184813	-1.73964419	9.68E-02	1.27E-01	-6.366372522
Slc35b1	-0.281568015	8.988351259	-2.262932787	3.46E-02	4.99E-02	-5.450514294
Mrpl37	-0.281682293	7.65355069	-1.942913938	6.58E-02	9.01E-02	-6.030047054
Bnip2	-0.281697825	10.37656684	-3.101300628	5.49E-03	9.25E-03	-3.713207947
Calr	-0.282482607	11.15746469	-2.352200029	2.87E-02	4.19E-02	-5.27918092
Gsdmd	-0.282848059	9.18949689	-3.711327205	1.32E-03	2.51E-03	-2.324138575
Hspd1	-0.283233427	10.00018943	-2.450177682	2.33E-02	3.46E-02	-5.086775587
Scarb2	-0.283358515	8.799482496	-2.796370004	1.09E-02	1.74E-02	-4.374708877
Glrx5	-0.283449127	8.17956533	-2.187345544	4.04E-02	5.76E-02	-5.592457984
Nob1	-0.283472456	7.075969687	-1.668708897	1.10E-01	1.44E-01	-6.477282447
Exosc5	-0.284034328	7.486606062	-2.027680632	5.57E-02	7.72E-02	-5.882178168
Ppp2r4	-0.28445424	7.941475074	-2.103315931	4.79E-02	6.73E-02	-5.746700835
Mtap	-0.284758899	6.490741506	-1.028935518	3.15E-01	3.68E-01	-7.304000709
Sp100	-0.284958089	9.233526579	-2.995353427	6.99E-03	1.16E-02	-3.946150381
Nop16	-0.285302765	7.266064423	-1.351081864	1.91E-01	2.35E-01	-6.928888505
Srp72	-0.285518331	9.516169769	-3.823515206	1.02E-03	1.96E-03	-2.062281037
Sf3b5	-0.285704091	9.730834822	-3.976505566	7.07E-04	1.41E-03	-1.70314189
LOC102639105	-0.286593172	10.94276412	-2.536835639	1.93E-02	2.92E-02	-4.913033874
Lysmd1	-0.286685152	8.534767984	-1.876143324	7.49E-02	1.01E-01	-6.143444455
Prdx3	-0.286765209	8.534698784	-2.488922801	2.14E-02	3.21E-02	-5.009495427
Cst6	-0.286819191	3.923886683	-1.633110002	1.18E-01	1.52E-01	-6.531617948
Gm10052	-0.28704228	11.91551423	-2.912756984	8.42E-03	1.37E-02	-4.125539119
Dnajc3	-0.2872268	10.05251622	-4.966184022	6.82E-05	1.67E-04	0.64129228
Cirh1a	-0.287921166	6.874513629	-1.919470167	6.89E-02	9.39E-02	-6.070177136
Gm6444	-0.288045274	8.746531235	-1.572698005	1.31E-01	1.68E-01	-6.621746848
Mcts2	-0.28812009	8.153632982	-2.61780514	1.62E-02	2.49E-02	-4.747858941
Bzw2	-0.289536067	7.594191642	-1.781112959	8.96E-02	1.19E-01	-6.299944821
Rbl1	-0.289594382	8.340940102	-3.736650222	1.25E-03	2.37E-03	-2.265158907
Prmt1	-0.290118846	8.313782018	-2.320507442	3.07E-02	4.46E-02	-5.340455153
Ccdc102a	-0.290641881	6.843133686	-0.906606644	3.75E-01	4.30E-01	-7.422528893
Mta3	-0.290673417	7.926485239	-4.679565269	1.34E-04	3.09E-04	-0.037700925
Nol9	-0.290939206	7.573180472	-2.605359732	1.67E-02	2.54E-02	-4.773418719
Noc2l	-0.291277943	8.100415156	-2.969138045	7.41E-03	1.22E-02	-4.003305449
Ddx21	-0.291403763	8.681778884	-2.928463812	8.13E-03	1.33E-02	-4.091583276
Tcerg1	-0.293343051	8.936153765	-3.975557986	7.09E-04	1.42E-03	-1.705372457
Eef1b2	-0.29441101	12.08500952	-4.26610006	3.56E-04	7.54E-04	-1.018847431
Nif3l1	-0.294633052	7.22407578	-0.824299669	4.19E-01	4.75E-01	-7.49448006
Ciita	-0.294815921	8.299183292	-1.948456424	6.51E-02	8.92E-02	-6.020510449
Tm4sf5	-0.295093902	7.421446797	-1.429266134	1.68E-01	2.10E-01	-6.82486875
Rab26os	-0.297152243	10.08927169	-1.97105437	6.23E-02	8.57E-02	-5.981434657
Topbp1	-0.297541941	8.513326381	-2.807522995	1.07E-02	1.70E-02	-4.351024714
Rsph3a	-0.297639791	6.627604681	-0.827456163	4.17E-01	4.73E-01	-7.491838411
Nup153	-0.298154362	9.173346848	-4.419567938	2.47E-04	5.41E-04	-0.654851366
Chsy1	-0.299350966	7.506363413	-2.522020214	2.00E-02	3.00E-02	-4.942965306
Rhobtb2	-0.300247034	6.436811013	-1.656266501	1.13E-01	1.46E-01	-6.496375562
Snrpb	-0.301182179	9.35665934	-2.358756055	2.83E-02	4.14E-02	-5.266445587
Sprr4	-0.301503042	5.221341317	-1.344649851	1.93E-01	2.37E-01	-6.937228884
Cyth4	-0.301578999	9.170830209	-1.544451347	1.38E-01	1.75E-01	-6.662973225
Haao	-0.301930942	7.98754067	-2.529308251	1.97E-02	2.96E-02	-4.928252885
Ptplad1	-0.301943282	6.051661526	-1.212805715	2.39E-01	2.88E-01	-7.1007177
Hsph1	-0.302232515	7.823776502	-0.815201764	4.24E-01	4.79E-01	-7.502041125
B230217C12Rik	-0.302372891	7.127403571	-1.243116051	2.28E-01	2.76E-01	-7.064410722
Gramd3	-0.304017023	9.164135914	-3.940004111	7.71E-04	1.53E-03	-1.789013709
Sh3bgrl	-0.304558701	9.107990387	-2.660579058	1.48E-02	2.28E-02	-4.659551399
Atp6v1b2	-0.305117938	9.200218354	-3.015589005	6.67E-03	1.11E-02	-3.901897786
Pdia4	-0.305185974	8.242289352	-2.635608167	1.56E-02	2.40E-02	-4.711190273
Fli1	-0.305326555	8.345223609	-2.709477297	1.33E-02	2.07E-02	-4.557749201
Mov10	-0.305784469	7.662376158	-1.922639916	6.84E-02	9.34E-02	-6.064771066
Ero1lb	-0.306266313	8.924627302	-3.684719605	1.41E-03	2.66E-03	-2.386024604
Zufsp	-0.306516541	8.950817547	-4.260474589	3.60E-04	7.63E-04	-1.032178015
Arl6ip1	-0.307247062	10.71182211	-3.226521738	4.11E-03	7.10E-03	-3.434164957

Cbr3	-0.307485398	6.054239012	-0.98070226	3.38E-01	3.92E-01	-7.35236704
Atxn10	-0.30758041	8.518197488	-3.446219267	2.47E-03	4.48E-03	-2.936231137
Nans	-0.308318979	8.399407472	-2.466599752	2.25E-02	3.35E-02	-5.054100902
Trmt112	-0.30888374	8.077911018	-2.019564106	5.66E-02	7.83E-02	-5.896521123
Kdm7a	-0.309016956	9.319035151	-5.188477758	4.06E-05	1.04E-04	1.165532433
Lamtor2	-0.309405342	9.135664314	-2.282610363	3.32E-02	4.80E-02	-5.413083768
Sh3gl1	-0.309529291	8.374754348	-3.451705443	2.44E-03	4.43E-03	-2.923677429
Cct6a	-0.309681848	8.910795952	-3.633211527	1.59E-03	2.98E-03	-2.505563691
Rragd	-0.309684267	5.30797966	-2.269510286	3.41E-02	4.93E-02	-5.438024234
Zbtb38	-0.309966162	6.478478237	-2.927745753	8.14E-03	1.33E-02	-4.093137258
Gtpbp1	-0.310593994	9.612021093	-2.929583157	8.11E-03	1.33E-02	-4.08916054
Suclg2	-0.310899173	8.116859187	-2.964611052	7.49E-03	1.23E-02	-4.013154982
Prep	-0.311851044	7.648154436	-2.59636857	1.70E-02	2.59E-02	-4.791846132
Tomm5	-0.312376533	7.026994105	-1.54378013	1.38E-01	1.75E-01	-6.66394569
Hist1h2bl	-0.312785702	10.89743868	-3.141311326	5.00E-03	8.50E-03	-3.624465703
Cbfb	-0.313021089	9.44023713	-3.919254671	8.10E-04	1.59E-03	-1.837779234
Tfdp1	-0.313098359	8.605353186	-3.15002847	4.91E-03	8.36E-03	-3.605078019
Cd2ap	-0.313119266	9.396999029	-3.239491784	3.99E-03	6.92E-03	-3.405050095
Nme1	-0.31322179	8.797112597	-2.200590142	3.93E-02	5.62E-02	-5.567800655
1110008L16Rik	-0.313311109	6.859901689	-1.212331755	2.39E-01	2.88E-01	-7.101279272
Kti12	-0.314122743	7.035665745	-2.398869505	2.60E-02	3.83E-02	-5.188083863
Rps11-ps1	-0.314407929	8.616557685	-2.063183298	5.19E-02	7.24E-02	-5.818991977
Gtf2h5	-0.314522375	9.139695933	-2.600083606	1.69E-02	2.57E-02	-4.784236056
Parp4	-0.315508507	8.761551488	-3.587572067	1.77E-03	3.31E-03	-2.611175938
Nudt21	-0.315511748	7.513079966	-1.658255206	1.12E-01	1.46E-01	-6.493331181
Dennd2d	-0.316640148	6.652404639	-1.912462854	6.98E-02	9.49E-02	-6.08210618
Snord100	-0.316707493	10.07975671	-2.054011542	5.29E-02	7.35E-02	-5.835384953
Smc4	-0.317396549	9.806815031	-3.763221499	1.17E-03	2.24E-03	-2.203189955
Itch	-0.318451993	9.243875922	-4.077122287	5.57E-04	1.14E-03	-1.465928309
Bank1	-0.319127935	5.867947872	-1.548586713	1.37E-01	1.74E-01	-6.656974492
LOC102634244	-0.319311346	2.728366817	-0.696841796	4.94E-01	5.47E-01	-7.593176147
Usp45	-0.319966904	6.922613442	-1.738886491	9.70E-02	1.28E-01	-6.367575472
Abce1	-0.319991365	8.300199417	-3.03885614	6.33E-03	1.06E-02	-3.850873544
Slk	-0.320247611	8.967968012	-5.745475713	1.13E-05	3.19E-05	2.464171123
Pxn	-0.320257438	9.06000592	-3.991042381	6.83E-04	1.37E-03	-1.668914198
Ptpn11	-0.320514712	7.607147497	-2.830937909	1.01E-02	1.62E-02	-4.301165317
Tyms	-0.321079803	7.393578347	-1.931285137	6.73E-02	9.19E-02	-6.049994971
Ndufaf2	-0.322409667	7.725851644	-3.689791976	1.39E-03	2.63E-03	-2.374233815
Tbrg4	-0.322608367	7.34630806	-2.463328153	2.27E-02	3.37E-02	-5.060619837
Itgb2	-0.322609655	9.371793047	-2.480549208	2.18E-02	3.26E-02	-5.026252814
Polr1d	-0.323394066	9.831316368	-3.411234234	2.68E-03	4.82E-03	-3.016158154
Rrp15	-0.32449735	6.751742079	-1.856921848	7.77E-02	1.04E-01	-6.175569793
Ostc	-0.32459686	8.612321925	-3.383909353	2.85E-03	5.10E-03	-3.078427469
Hspa8	-0.324710483	11.61875019	-2.627475678	1.59E-02	2.44E-02	-4.727956004
Fes	-0.325205807	7.66689926	-3.294811051	3.51E-03	6.14E-03	-3.28045245
Mthfd1l	-0.325614919	6.283367817	-1.740432591	9.67E-02	1.27E-01	-6.365120413
Snord33	-0.32583643	6.206983508	-0.662936061	5.15E-01	5.67E-01	-7.616779255
Coro1c	-0.325868487	9.102477004	-3.268167472	3.73E-03	6.50E-03	-3.340546103
Ffar2	-0.326564767	6.022483737	-1.56144949	1.34E-01	1.71E-01	-6.638234811
Oaz1-ps	-0.327201885	10.92411842	-3.650624565	1.53E-03	2.87E-03	-2.465191688
Akr1a1	-0.327291757	11.07702221	-5.522649945	1.88E-05	5.13E-05	1.94766169
Fmnl2	-0.328078415	9.424976127	-4.410371133	2.53E-04	5.52E-04	-0.676677748
Arhgef9	-0.328569821	5.903035744	-2.026399366	5.58E-02	7.74E-02	-5.884444886
Samm50	-0.328719338	8.308823774	-2.704590264	1.34E-02	2.09E-02	-4.567963518
Ppat	-0.329067187	8.311824528	-2.998410976	6.94E-03	1.15E-02	-3.939471379
H2-DMb1	-0.329320998	9.550777415	-1.356433358	1.90E-01	2.34E-01	-6.921923845
Sco2	-0.329496357	6.791726913	-1.182240477	2.51E-01	3.01E-01	-7.136542134
Psen2	-0.329979305	6.98588596	-2.816406588	1.05E-02	1.67E-02	-4.33212971
Rad51ap1	-0.330241956	5.652995835	-1.524833884	1.42E-01	1.81E-01	-6.691256583
Gmcl1l	-0.330527461	5.211788081	-1.173925844	2.54E-01	3.04E-01	-7.146149447
Txnl1	-0.330720538	9.386961167	-3.735030512	1.25E-03	2.38E-03	-2.268933679
Pnpt1	-0.332551975	7.344717411	-4.312733704	3.18E-04	6.83E-04	-0.908302148
Fbxo5	-0.332738409	7.738206919	-2.241366665	3.61E-02	5.20E-02	-5.491312806
4933426M11Rik	-0.333561636	7.863967025	-2.220071956	3.78E-02	5.41E-02	-5.531364507
Jarid2	-0.333617101	8.235004869	-4.020604634	6.37E-04	1.29E-03	-1.599260264
Cisd1	-0.333807843	7.144782722	-1.633188692	1.18E-01	1.52E-01	-6.531498834
Gpr132	-0.33450197	9.477460002	-3.128334552	5.16E-03	8.74E-03	-3.653292111
Aco1	-0.334668236	8.073504308	-3.34934714	3.09E-03	5.50E-03	-3.156984329
Strap	-0.334750177	8.470731268	-3.717916667	1.30E-03	2.47E-03	-2.308798618
Bdkrb2	-0.33643079	5.204182098	-1.574943066	1.30E-01	1.67E-01	-6.618444924
Slc46a3	-0.336556348	10.42664661	-4.399944332	2.59E-04	5.64E-04	-0.701421755
Psmg1	-0.337328075	6.642859818	-1.330105408	1.98E-01	2.43E-01	-6.955965396
Magohb	-0.337757963	7.119054119	-2.442828344	2.37E-02	3.51E-02	-5.101359685
Zdhhc14	-0.338346928	7.707144481	-2.523541109	1.99E-02	2.99E-02	-4.93989691
Atic	-0.339612676	8.426220159	-1.855977222	7.78E-02	1.04E-01	-6.177142499
Snhg4	-0.339959055	7.755947546	-2.993479243	7.02E-03	1.16E-02	-3.950243083

Manf	-0.340230342	7.494804591	-3.651952678	1.52E-03	2.86E-03	-2.462110768
Cdc37	-0.341135984	9.97172711	-4.434554599	2.39E-04	5.25E-04	-0.619281823
Sf3b3	-0.343304896	8.965748988	-3.405948117	2.71E-03	4.88E-03	-3.028215358
Ect2	-0.344054896	6.642866292	-2.092457845	4.89E-02	6.87E-02	-5.766348976
Mtpn	-0.345171887	10.54419486	-5.358365021	2.74E-05	7.27E-05	1.564173602
Hist1h2bf	-0.346706986	8.767130309	-1.664226433	1.11E-01	1.45E-01	-6.484173447
Scaper	-0.347522167	8.834122499	-5.104695276	4.93E-05	1.24E-04	0.968259287
Lmtk2	-0.348742393	8.379495969	-4.233742444	3.84E-04	8.09E-04	-1.095509562
Dnajb11	-0.34904224	8.899433147	-4.288634784	3.37E-04	7.20E-04	-0.965436947
Snrpd3	-0.349172765	9.813793417	-3.002587765	6.87E-03	1.14E-02	-3.930343165
Phb2	-0.349311744	8.956383479	-3.957998769	7.39E-04	1.47E-03	-1.746693528
Wdr75	-0.349772296	7.430369093	-2.140250004	4.44E-02	6.28E-02	-5.6793782
Cpsf3	-0.350849226	8.362271124	-4.094153784	5.35E-04	1.10E-03	-1.425709524
Cenpa	-0.351338158	6.942435553	-2.629625841	1.58E-02	2.43E-02	-4.723525785
Timm17a	-0.351512083	9.296017046	-2.821230968	1.03E-02	1.65E-02	-4.321857376
Trim28	-0.351834874	8.175721282	-3.374744866	2.92E-03	5.20E-03	-3.099280192
Exosc8	-0.352222284	8.512593988	-3.493188702	2.21E-03	4.05E-03	-2.828584226
Ap1b1	-0.353382589	8.292083105	-3.841336053	9.74E-04	1.89E-03	-2.020559444
Arf3	-0.353402051	8.322745693	-2.023907116	5.61E-02	7.77E-02	-5.888851241
Ptbp1	-0.353460131	10.03914115	-4.048573947	5.96E-04	1.21E-03	-1.533303364
Nup205	-0.35364714	7.367519451	-3.586762749	1.77E-03	3.31E-03	-2.613046018
Mcm4	-0.35467807	8.473777556	-3.610694335	1.68E-03	3.14E-03	-2.557707052
Mns1	-0.354835463	5.7558641	-1.578078093	1.30E-01	1.66E-01	-6.613827897
Acss1	-0.355859895	7.290714487	-1.920166159	6.88E-02	9.38E-02	-6.06899064
Rdh9	-0.35675455	3.683949123	-1.05754505	3.02E-01	3.55E-01	-7.274322873
Rbm17	-0.357356282	9.425973558	-3.872156977	9.05E-04	1.76E-03	-1.948328157
Tram2	-0.357977514	6.796375749	-2.299160856	3.21E-02	4.65E-02	-5.381452169
Hsd17b12	-0.358105335	7.968276626	-3.033566988	6.40E-03	1.07E-02	-3.862485705
Ahsa1	-0.359127289	9.544638363	-2.711418932	1.32E-02	2.06E-02	-4.553688592
Mat2a	-0.359516	9.848358216	-3.149828637	4.91E-03	8.36E-03	-3.605522674
Prmt5	-0.360146456	6.821657915	-3.418511617	2.63E-03	4.75E-03	-2.99955057
Akr7a5	-0.360483591	6.933373031	-1.383367766	1.81E-01	2.24E-01	-6.886521922
Supt20	-0.361739973	9.797387864	-6.761348287	1.20E-06	4.08E-06	4.753294164
Atp7a	-0.362329815	8.017220413	-3.080364115	5.75E-03	9.66E-03	-3.759480271
Rin3	-0.362705325	9.071171021	-3.977830474	7.05E-04	1.41E-03	-1.700022989
Rps3	-0.364321633	9.897513018	-5.60284422	1.56E-05	4.32E-05	2.134058531
Scarna13	-0.364726067	7.549339429	-1.069429979	2.97E-01	3.49E-01	-7.261779456
Il23r	-0.364803582	5.434826588	-1.059983402	3.01E-01	3.54E-01	-7.271759667
Spata5	-0.367118048	7.444439401	-4.585797057	1.67E-04	3.79E-04	-0.260247872
Exosc1	-0.367418201	7.806353045	-4.791333647	1.03E-04	2.44E-04	0.227368557
Agpat5	-0.368016849	6.877050209	-2.713671115	1.31E-02	2.06E-02	-4.548976796
Yes1	-0.368765942	5.503583187	-1.169172104	2.56E-01	3.06E-01	-7.151615608
AW112010	-0.369177844	10.15438181	-2.224002169	3.75E-02	5.37E-02	-5.523990042
Cd70	-0.369560703	4.73798538	-1.884545917	7.37E-02	9.96E-02	-6.129327384
Rpl27a	-0.370150936	9.431709692	-3.003160533	6.86E-03	1.14E-02	-3.929091015
Prmt3	-0.372476486	6.812243127	-3.898986228	8.49E-04	1.67E-03	-1.885378391
Snora31	-0.372635383	10.00919895	-3.777651585	1.13E-03	2.17E-03	-2.169502466
Psmg4	-0.373069814	8.352136051	-2.933835623	8.03E-03	1.31E-02	-4.079952942
Srsf2	-0.373182855	9.95749728	-4.710253329	1.24E-04	2.90E-04	0.035105664
Gpn1	-0.373569782	7.770859965	-2.323696172	3.05E-02	4.43E-02	-5.334311987
Rnf167	-0.37383019	9.12268073	-4.472811413	2.18E-04	4.84E-04	-0.528472053
AI662270	-0.374023293	9.907195292	-3.952105352	7.49E-04	1.49E-03	-1.760556677
Gpi1	-0.374345762	10.49337066	-3.468686906	2.34E-03	4.27E-03	-2.884786195
Nhp2	-0.374779439	8.414663369	-3.434246203	2.54E-03	4.60E-03	-2.963609813
Sepw1	-0.375104208	10.99348624	-4.063195765	5.76E-04	1.18E-03	-1.498801801
Mamstr	-0.375186932	5.040955657	-1.808977089	8.51E-02	1.13E-01	-6.254666251
2700094K13Rik	-0.375832486	9.542577716	-3.800148058	1.07E-03	2.07E-03	-2.116937685
Zfp385a	-0.376028246	8.666631898	-3.873721633	9.02E-04	1.76E-03	-1.944658823
Snora44	-0.377822019	7.759145923	-2.036031378	5.48E-02	7.60E-02	-5.867381295
Pole4	-0.379548809	7.197415939	-2.652769913	1.50E-02	2.32E-02	-4.675726118
Igsf9	-0.380427206	6.888620389	-2.078203836	5.04E-02	7.05E-02	-5.792041791
Hk2	-0.380713173	7.114385112	-3.536459766	2.00E-03	3.69E-03	-2.729085739
Thg1l	-0.380892786	7.091710635	-1.646591184	1.15E-01	1.49E-01	-6.511146962
Arhgap31	-0.38106221	9.032510211	-3.298068801	3.48E-03	6.11E-03	-3.27309431
Ints4	-0.381383144	8.492489274	-3.790844797	1.10E-03	2.11E-03	-2.138682189
Zfp97	-0.381681339	3.31783815	-0.382061488	7.06E-01	7.48E-01	-7.768267299
Tyk2	-0.381831441	9.144217315	-4.455404404	2.27E-04	5.02E-04	-0.569792486
Bora	-0.382422554	6.362899068	-1.469040173	1.57E-01	1.97E-01	-6.770111179
Qtrtd1	-0.383197955	5.782516216	-1.433818855	1.67E-01	2.08E-01	-6.818663148
Hist2h2bb	-0.384751974	5.166653987	-1.285153475	2.13E-01	2.59E-01	-7.012784197
Casp7	-0.385246549	8.173822232	-4.104717084	5.22E-04	1.07E-03	-1.400756426
Ccnb1	-0.386064128	6.983603521	-1.785671417	8.89E-02	1.18E-01	-6.29257256
H60b	-0.386897898	7.421805945	-1.994309586	5.95E-02	8.20E-02	-5.940901883
Hn1	-0.386953657	9.344102646	-3.957220351	7.40E-04	1.47E-03	-1.74852477
Snora3	-0.387920695	8.690041507	-2.417657593	2.50E-02	3.69E-02	-5.151125149
Polr2g	-0.388072967	10.38476024	-4.274479133	3.49E-04	7.42E-04	-0.998989733

Sssca1	-0.388217885	7.475136068	-3.734851516	1.25E-03	2.38E-03	-2.269350815
Whsc1	-0.389845119	8.342371218	-4.967819982	6.79E-05	1.67E-04	0.645159471
Rnase6	-0.391241368	10.16806111	-3.647921304	1.54E-03	2.89E-03	-2.471461898
Esco2	-0.391769023	5.266789131	-1.770892934	9.14E-02	1.21E-01	-6.316423104
A530032D15Rik	-0.393080314	9.332967056	-3.421125797	2.62E-03	4.73E-03	-2.993582418
Skap2	-0.394076329	9.940936205	-6.467705259	2.26E-06	7.34E-06	4.103794363
Gart	-0.394158409	7.601313276	-3.57875554	1.81E-03	3.37E-03	-2.631542936
Lpcat2	-0.394808256	6.380555954	-1.746369462	9.56E-02	1.26E-01	-6.35567817
Ap1g2	-0.395015499	7.928785695	-4.544493065	1.84E-04	4.14E-04	-0.35830123
Twistnb	-0.396411603	8.895074664	-4.181933079	4.34E-04	9.06E-04	-1.218172589
Asah1	-0.396425853	9.09531145	-5.263392744	3.41E-05	8.85E-05	1.341560796
Mir5107	-0.39814195	10.20298079	-3.549719068	1.94E-03	3.59E-03	-2.698536818
Ccnl1	-0.400591917	10.23467425	-5.499416102	1.98E-05	5.39E-05	1.893557652
Gfer	-0.401187486	7.717832973	-3.228343049	4.09E-03	7.08E-03	-3.430078822
Bcap29	-0.401305643	8.152715637	-3.152058619	4.88E-03	8.33E-03	-3.600560096
Mir5121	-0.401391632	6.695726194	-1.109131139	2.80E-01	3.31E-01	-7.218972458
Snora7a	-0.40146249	7.035007376	-1.245522841	2.27E-01	2.75E-01	-7.061494672
Ube2s	-0.401523402	10.14046106	-3.021399677	6.58E-03	1.10E-02	-3.889169245
Mpc2	-0.402578916	8.38655819	-4.320763764	3.12E-04	6.71E-04	-0.889260547
Alyref	-0.403090679	6.584533898	-1.82581449	8.24E-02	1.10E-01	-6.227058681
Ttc9c	-0.403213458	8.192312387	-3.198910153	4.38E-03	7.52E-03	-3.496018479
Abhd16a	-0.405193522	7.781922627	-3.243374246	3.96E-03	6.86E-03	-3.396327457
Pdlim4	-0.405867022	7.972144293	-3.001770025	6.88E-03	1.14E-02	-3.932130696
Tagap	-0.406120546	8.707594605	-2.832789649	1.01E-02	1.61E-02	-4.297214458
Derl2	-0.40635029	8.907382488	-3.175779622	4.62E-03	7.92E-03	-3.547696427
Prdx2	-0.407296952	8.49374818	-4.152704172	4.65E-04	9.66E-04	-1.28732221
Ruvbl2	-0.408026145	7.877198794	-3.290009888	3.55E-03	6.21E-03	-3.291292533
Pim1	-0.408724358	8.898935446	-3.242414166	3.96E-03	6.88E-03	-3.398484761
Nup85	-0.409189287	8.35414788	-4.036655629	6.13E-04	1.24E-03	-1.561415452
Rpa3	-0.410294672	8.05759867	-2.876126035	9.15E-03	1.48E-02	-4.204432371
Cnih4	-0.410949928	9.986742497	-4.211567144	4.05E-04	8.50E-04	-1.148025024
Bcl2l14	-0.411257652	6.679585554	-2.985691848	7.14E-03	1.18E-02	-3.967237805
Sla	-0.411923963	9.398650904	-3.416717969	2.64E-03	4.77E-03	-3.003644728
Parp8	-0.41248213	10.6745526	-5.559609592	1.72E-05	4.75E-05	2.033635796
Tmem219	-0.412881318	9.290776934	-3.040953334	6.30E-03	1.05E-02	-3.846267094
Dvl1	-0.413082049	7.070664254	-4.026044905	6.29E-04	1.27E-03	-1.586435251
Eprs	-0.413465682	8.440869468	-4.882681975	8.29E-05	2.00E-04	0.443756459
Rrbp1	-0.413546691	8.854156168	-4.894173709	8.07E-05	1.95E-04	0.470958122
Cyba	-0.414131343	10.80025398	-4.730437269	1.19E-04	2.77E-04	0.082981475
Npepl1	-0.417239266	7.925384589	-3.214108301	4.23E-03	7.29E-03	-3.461994459
Txn2	-0.420941771	8.461302864	-3.308151503	3.40E-03	5.99E-03	-3.250306791
2010016I18Rik	-0.421191404	6.393384761	-2.432054719	2.42E-02	3.58E-02	-5.122695319
Usp12	-0.421624293	8.675194572	-4.461045874	2.24E-04	4.96E-04	-0.556401128
Nmral1	-0.422559237	6.393974288	-2.308505362	3.14E-02	4.57E-02	-5.36353309
Dis3	-0.423272826	6.867589886	-2.877165737	9.12E-03	1.47E-02	-4.20219896
Efhd2	-0.42347155	11.07075105	-6.304316967	3.24E-06	1.02E-05	3.737921765
Srebf2	-0.423678021	9.669078356	-6.174729463	4.31E-06	1.33E-05	3.445551083
Timm10	-0.423709426	7.793607181	-2.07556903	5.06E-02	7.08E-02	-5.796778474
Kpna3	-0.424349451	9.388301601	-5.156432638	4.37E-05	1.11E-04	1.090128203
Eif5a	-0.424851914	6.773670585	-4.393595007	2.63E-04	5.71E-04	-0.716488618
Stx16	-0.424953647	8.670860697	-3.988259362	6.87E-04	1.38E-03	-1.675468196
Lsm4	-0.426306864	9.095897235	-3.989481856	6.86E-04	1.38E-03	-1.672589298
Hltf	-0.42806741	8.432396669	-5.2927967	3.19E-05	8.32E-05	1.410550318
Ly6e	-0.430811525	11.03954737	-5.986443712	6.56E-06	1.94E-05	3.017440863
Uhrf1	-0.430934002	8.288915739	-3.517522772	2.09E-03	3.84E-03	-2.772667415
Ran	-0.430941104	9.152434151	-4.199185326	4.17E-04	8.74E-04	-1.177338936
Ldha	-0.431486713	8.904286631	-5.330234762	2.92E-05	7.71E-05	1.498303377
Gsn	-0.431715307	7.769328912	-3.443702789	2.48E-03	4.51E-03	-2.941987667
Fam162a	-0.433172315	7.857495524	-3.401907177	2.74E-03	4.92E-03	-3.037428902
Eif1ax	-0.433658137	10.09669635	-4.209252007	4.07E-04	8.55E-04	-1.153506588
Cxcl16	-0.43369307	10.71335507	-3.513535524	2.11E-03	3.87E-03	-2.781836307
Kif2a	-0.434256399	9.878273478	-5.802640908	9.92E-06	2.83E-05	2.595944244
Atp5g3	-0.434925402	10.21215774	-5.949774136	7.12E-06	2.09E-05	2.933624916
Amz1	-0.435078578	6.691240708	-2.450124282	2.33E-02	3.46E-02	-5.086881641
Ap1ar	-0.435326788	8.580336413	-4.767345256	1.09E-04	2.57E-04	0.170502566
Cacna1e	-0.43546272	6.591674235	-1.916041939	6.93E-02	9.44E-02	-6.076017052
Cd52	-0.435525002	11.91698836	-6.90242051	8.87E-07	3.08E-06	5.061528623
Vamp3	-0.436254795	8.919361864	-4.907610696	7.82E-05	1.89E-04	0.502757994
Nusap1	-0.437147424	7.094866574	-2.079965464	5.02E-02	7.03E-02	-5.788872662
Tas1r3	-0.437681474	5.861095658	-2.805513073	1.07E-02	1.70E-02	-4.35529604
Vav1	-0.439694569	8.620204871	-7.200178309	4.74E-07	1.74E-06	5.703781482
Zfp263	-0.439903551	7.414723533	-3.760939641	1.18E-03	2.25E-03	-2.208514863
Ncapd2	-0.439912085	7.284973666	-2.764571217	1.17E-02	1.85E-02	-4.442001774
Gtf3c6	-0.440405851	9.184720912	-5.86599697	8.60E-06	2.48E-05	2.741616631
Rrad	-0.441292116	7.070788238	-2.95450336	7.66E-03	1.26E-02	-4.035124824
Sec63	-0.441718283	8.714453264	-4.558004984	1.78E-04	4.02E-04	-0.326223954

Lgals9	-0.442580751	7.785499644	-1.944628857	6.56E-02	8.99E-02	-6.027098303
Etf1	-0.445301854	9.315167535	-7.085966926	6.02E-07	2.17E-06	5.458781511
Ankrd12	-0.445302721	8.381190505	-3.775415632	1.14E-03	2.18E-03	-2.1747239
Ywhag	-0.445486044	9.8174236	-4.410173761	2.53E-04	5.52E-04	-0.677146148
Ccnf	-0.445641456	5.685526494	-2.816818723	1.04E-02	1.67E-02	-4.331252474
Pcdhga5	-0.445835403	2.561782888	-0.791281542	4.38E-01	4.92E-01	-7.52154479
Sprr1a	-0.445956264	5.528731847	-2.170044224	4.18E-02	5.95E-02	-5.624527744
Golim4	-0.44630781	7.700473767	-6.004849989	6.29E-06	1.88E-05	3.059459278
Ubl4	-0.446387012	5.299219093	-0.891414642	3.83E-01	4.38E-01	-7.43628724
Trappc1	-0.446452369	9.005984639	-4.328492369	3.07E-04	6.60E-04	-0.870932204
LOC102635191	-0.448098095	7.570490105	-2.666556123	1.46E-02	2.26E-02	-4.647155709
Amd1	-0.44840954	8.885681069	-2.329028213	3.01E-02	4.39E-02	-5.324028675
Npc2	-0.4488836	11.23210372	-6.934306675	8.29E-07	2.89E-06	5.130849468
Inf2	-0.449273955	7.187858161	-3.497284682	2.19E-03	4.02E-03	-2.81917901
Nifk	-0.450032162	7.641157929	-2.986044081	7.13E-03	1.18E-02	-3.96646949
P4hb	-0.450143773	9.255836332	-5.209506556	3.87E-05	9.94E-05	1.214980252
Snord104	-0.451293991	9.806451539	-4.249928997	3.70E-04	7.81E-04	-1.057164802
Aatf	-0.452099792	8.245625066	-5.524311566	1.87E-05	5.11E-05	1.95152935
Lsm2	-0.45266239	8.462852179	-2.719191147	1.30E-02	2.03E-02	-4.537420513
Pml	-0.452662626	8.209215948	-4.076437095	5.58E-04	1.14E-03	-1.467545981
Cs	-0.453417032	9.651756851	-7.247717333	4.30E-07	1.58E-06	5.80526014
Plekho1	-0.453976709	9.786064377	-6.339238023	3.00E-06	9.53E-06	3.816381702
St6galnac2	-0.454121514	6.095586605	-2.167442037	4.21E-02	5.97E-02	-5.629337355
Olfr525	-0.454143906	3.338943877	-0.838427563	4.11E-01	4.67E-01	-7.482583091
Atox1	-0.455035395	11.67071986	-6.955929974	7.92E-07	2.78E-06	5.177785102
Mis18bp1	-0.455652179	5.871107781	-3.941646184	7.68E-04	1.52E-03	-1.78515295
Cmtm7	-0.455800202	9.37910178	-3.997401804	6.73E-04	1.36E-03	-1.65393562
Ubb	-0.456133728	7.450289029	-1.876260194	7.48E-02	1.01E-01	-6.143248409
Vps36	-0.456530228	8.234588843	-5.887987019	8.18E-06	2.36E-05	2.792084347
Fermt3	-0.456707643	7.847342176	-4.365739292	2.81E-04	6.06E-04	-0.782581583
Ano6	-0.458200047	7.218410395	-4.267946462	3.54E-04	7.52E-04	-1.014471812
Gng12	-0.458998758	6.75628452	-2.765103994	1.17E-02	1.85E-02	-4.440877194
Nfatc1	-0.459632492	8.262931748	-6.035022951	5.88E-06	1.77E-05	3.12826169
Mx1	-0.460396282	8.368023461	-2.612125596	1.64E-02	2.51E-02	-4.7595309
Tfg	-0.460606392	8.811251717	-5.240121666	3.60E-05	9.29E-05	1.286919462
Xpr1	-0.460959562	7.8906533	-5.60240133	1.56E-05	4.32E-05	2.133030635
Tmem140	-0.461170675	6.407631326	-2.768683724	1.16E-02	1.84E-02	-4.433318571
Psme2	-0.461508558	7.620591121	-3.838810216	9.79E-04	1.90E-03	-2.026474806
Ncf1	-0.462031622	9.420738111	-7.820421946	1.34E-07	5.42E-07	7.004434177
Dhrs11	-0.462499758	6.790519072	-4.490781704	2.09E-04	4.64E-04	-0.485812411
Lpxn	-0.462593822	6.884859313	-2.865681046	9.36E-03	1.51E-02	-4.22685038
Dbf4	-0.462689567	7.068987923	-4.546344177	1.83E-04	4.12E-04	-0.353906665
Rrp12	-0.46275725	5.841361792	-1.720588406	1.00E-01	1.31E-01	-6.396507443
Fgfr1op	-0.463777035	7.221890774	-2.891965216	8.83E-03	1.43E-02	-4.170370663
Tmem189	-0.464448133	7.167980545	-1.857985541	7.75E-02	1.04E-01	-6.173798168
1500012F01Rik	-0.464531991	9.987463355	-6.202001887	4.06E-06	1.26E-05	3.507239476
Psmb7	-0.465214505	10.36739486	-4.681544733	1.33E-04	3.08E-04	-0.033004194
Cmc1	-0.465732988	8.080299865	-4.80677578	9.91E-05	2.36E-04	0.263966655
Prkch	-0.466213872	8.482975301	-4.400313932	2.59E-04	5.63E-04	-0.700544681
Erh	-0.466235099	4.905403608	-0.672605081	5.09E-01	5.62E-01	-7.610163035
Ctps	-0.467695668	6.459737716	-3.703425007	1.35E-03	2.55E-03	-2.342527429
Defa25	-0.468309837	8.145042056	-1.885356878	7.35E-02	9.95E-02	-6.127962542
Ak6	-0.469780496	8.207344207	-2.711419889	1.32E-02	2.06E-02	-4.553686588
Rrm1	-0.470127918	7.725356115	-4.110358395	5.15E-04	1.06E-03	-1.387427668
Snx3	-0.471210156	9.27158125	-5.298737477	3.14E-05	8.23E-05	1.424481771
Atmin	-0.47148145	8.517875431	-5.260549869	3.43E-05	8.90E-05	1.33488755
Bri3bp	-0.4716938	8.037390425	-4.26684164	3.55E-04	7.53E-04	-1.017090042
Acadl	-0.472278396	9.022550491	-6.282401716	3.40E-06	1.07E-05	3.688611331
Dars	-0.472692219	8.259124011	-4.650402531	1.43E-04	3.29E-04	-0.106903364
Srm	-0.472764186	7.430320503	-2.356177767	2.84E-02	4.16E-02	-5.271456443
Tbc1d10a	-0.473040509	6.491908451	-2.611691865	1.64E-02	2.52E-02	-4.760421732
Mrpl18	-0.473055216	9.000677553	-5.002636069	6.26E-05	1.55E-04	0.727431367
Gm10349	-0.475107964	8.717680521	-2.157266119	4.29E-02	6.09E-02	-5.648110583
Ift43	-0.475662741	6.928648174	-3.48280287	2.27E-03	4.15E-03	-2.852419606
Cand1	-0.475810753	9.000809389	-6.106746932	5.01E-06	1.53E-05	3.291420577
Gmfb	-0.475890923	9.560019035	-5.656599906	1.38E-05	3.85E-05	2.258690185
Rab11a	-0.475961082	9.207101036	-7.015827981	6.98E-07	2.47E-06	5.307488089
Nsmaf	-0.476259327	9.151293901	-5.474342825	2.10E-05	5.67E-05	1.835120598
Snord4a	-0.476262417	5.971007347	-1.510254767	1.46E-01	1.85E-01	-6.712089158
Cebpz	-0.476763532	7.962363838	-4.262391172	3.59E-04	7.60E-04	-1.027636439
Scarna9	-0.477292339	9.502353672	-1.651641226	1.14E-01	1.48E-01	-6.503445285
Rpl6	-0.477299812	5.501378309	-0.828282332	4.17E-01	4.73E-01	-7.491145436
Glipr1	-0.477442511	9.694623992	-5.169645567	4.24E-05	1.08E-04	1.121226601
Sigmar1	-0.478081863	7.008433043	-3.420694151	2.62E-03	4.73E-03	-2.994567949
Acyp1	-0.478984178	7.142637122	-2.440243786	2.38E-02	3.53E-02	-5.106482777
Kif22	-0.479235196	6.124604266	-2.878662897	9.09E-03	1.47E-02	-4.198982268

Dkc1	-0.481292964	6.678650642	-3.083706996	5.71E-03	9.59E-03	-3.752099791
Slc6a12	-0.481632941	6.258640874	-3.540524513	1.98E-03	3.66E-03	-2.719723657
E2f3	-0.481648494	6.281732951	-2.804787217	1.07E-02	1.71E-02	-4.356838235
Sap30	-0.482729781	9.327732592	-3.662579285	1.48E-03	2.80E-03	-2.437450917
Vdac3	-0.48465184	7.494490208	-2.74876379	1.22E-02	1.91E-02	-4.47532253
Cd226	-0.484662626	6.123845494	-1.724253816	9.96E-02	1.31E-01	-6.390730199
Mrps12	-0.484772517	8.937340406	-4.412764615	2.51E-04	5.49E-04	-0.670997512
Snrpd1	-0.484849368	8.995198001	-4.16684101	4.50E-04	9.37E-04	-1.253882391
Cnp	-0.485150772	6.633654953	-3.30687842	3.41E-03	6.00E-03	-3.25318521
Fuca2	-0.48597175	7.502970305	-3.391428125	2.80E-03	5.03E-03	-3.061307368
Tipin	-0.487032279	6.755392379	-2.379978101	2.70E-02	3.97E-02	-5.22508186
Tmem150b	-0.488180256	6.92995243	-3.09628978	5.55E-03	9.34E-03	-3.724292964
Abhd1	-0.490484309	6.92838884	-2.39160413	2.64E-02	3.88E-02	-5.202332356
Snhg6	-0.49048999	10.54493579	-5.082320382	5.20E-05	1.30E-04	0.91550798
Gpr35	-0.490999164	7.329617447	-3.867479264	9.15E-04	1.78E-03	-1.959296655
Ap1s3	-0.493019833	8.991451195	-4.278194903	3.46E-04	7.36E-04	-0.990182946
Mcm3	-0.494072344	7.943897165	-3.93932183	7.72E-04	1.53E-03	-1.790617788
Arhgap19	-0.4944493	5.605952042	-2.362555898	2.81E-02	4.11E-02	-5.259054925
Erp29	-0.4962225	9.155556823	-3.867516148	9.15E-04	1.78E-03	-1.959210177
Dexi	-0.496642973	7.619962511	-3.032334569	6.42E-03	1.07E-02	-3.865190333
Gm3453	-0.496663077	4.477139744	-1.644698644	1.15E-01	1.49E-01	-6.51402856
Spc24	-0.497148756	6.634818845	-2.913167343	8.41E-03	1.37E-02	-4.124652947
1500011K16Rik	-0.497728238	7.720054498	-5.045567382	5.66E-05	1.40E-04	0.828800795
Stx1b	-0.497821603	5.245496532	-2.431577673	2.42E-02	3.59E-02	-5.123638835
Mrpl16	-0.498252454	7.787653503	-5.47524628	2.09E-05	5.66E-05	1.83722712
Asna1	-0.498512086	8.282897091	-2.496104743	2.11E-02	3.16E-02	-4.995098636
Emp2	-0.499190414	5.419228437	-1.789343068	8.83E-02	1.17E-01	-6.286624481
Mak16	-0.499221443	7.980824769	-4.293251438	3.34E-04	7.13E-04	-0.954492878
Aurkb	-0.500805149	6.940124183	-1.956353604	6.41E-02	8.79E-02	-6.006889982
Ighg2c	-0.502402392	3.934996057	-0.592613776	5.60E-01	6.10E-01	-7.662125708
Rap1gds1	-0.502405665	8.047373339	-5.740577128	1.14E-05	3.23E-05	2.452864749
Ipo5	-0.50407301	8.195369606	-4.325915267	3.09E-04	6.63E-04	-0.877043956
Eif5b	-0.505693767	9.004737719	-5.778822671	1.05E-05	2.97E-05	2.541078275
Cmc2	-0.507336688	8.424309382	-4.493548479	2.07E-04	4.62E-04	-0.479244226
Ndufa12	-0.50743204	10.32803259	-5.277780855	3.30E-05	8.59E-05	1.37532649
Tlr13	-0.508359318	8.703241941	-4.71387752	1.23E-04	2.87E-04	0.043702777
Mrps2	-0.508370317	7.897738399	-3.474866148	2.31E-03	4.22E-03	-2.87062197
Arhgap11a	-0.508513302	8.113835898	-4.368323516	2.79E-04	6.03E-04	-0.776450633
Tars	-0.509547903	7.51792463	-4.736970262	1.17E-04	2.74E-04	0.098475698
Vars	-0.509814946	7.312199437	-3.951960987	7.49E-04	1.49E-03	-1.760896234
Arid4a	-0.50999319	9.500167141	-6.589085773	1.74E-06	5.77E-06	4.373547657
Ndufs8	-0.510959575	8.711072413	-4.916072853	7.67E-05	1.86E-04	0.52278088
Snora75	-0.511341721	8.34009513	-2.491239503	2.13E-02	3.19E-02	-5.004853841
Pcbp1	-0.511611687	8.591897761	-3.115251341	5.31E-03	8.97E-03	-3.682311999
Ubac2	-0.512011093	8.634059632	-5.993464067	6.46E-06	1.92E-05	3.033471343
Tln1	-0.512419732	9.251692598	-6.958710127	7.88E-07	2.76E-06	5.183815387
Zfp652	-0.514150074	8.087079624	-4.244763282	3.74E-04	7.90E-04	-1.069403043
Vimp	-0.514874285	9.51375182	-5.279760796	3.28E-05	8.56E-05	1.379971875
Bak1	-0.51603552	10.2180028	-5.581896463	1.64E-05	4.52E-05	2.085422546
Snord16a	-0.516882556	7.103492419	-2.039574455	5.44E-02	7.56E-02	-5.861091019
Mybbp1a	-0.516923362	7.535336552	-4.130804181	4.90E-04	1.01E-03	-1.339105602
LOC102638481	-0.51717359	10.61148712	-6.385367235	2.71E-06	8.67E-06	3.919807015
Arhgef40	-0.517533736	7.680435899	-3.882914498	8.82E-04	1.72E-03	-1.923095651
Tapbpl	-0.517654759	9.474355929	-6.240351721	3.73E-06	1.16E-05	3.59384313
Smc2	-0.518640689	8.104656369	-4.700664749	1.27E-04	2.95E-04	0.012358891
Rad51	-0.5190669	6.443806365	-1.809891844	8.49E-02	1.13E-01	-6.253171126
Mfsd4	-0.519386819	6.851478313	-2.479958747	2.19E-02	3.26E-02	-5.027433305
Soat1	-0.519935785	8.729764219	-4.445415738	2.33E-04	5.13E-04	-0.593502242
Usp32	-0.522306484	8.434299231	-7.77266163	1.47E-07	5.91E-07	6.906086982
Hmgcr	-0.524300172	8.820273889	-5.613389728	1.53E-05	4.23E-05	2.158528319
Mycbp	-0.524741718	7.872536434	-3.89001169	8.68E-04	1.70E-03	-1.906442815
Eml5	-0.524749857	7.86389125	-3.401606966	2.74E-03	4.92E-03	-3.038113275
Tbc1d14	-0.524988399	8.169596041	-6.912143598	8.69E-07	3.02E-06	5.082680409
Gm17767	-0.525327819	6.600401743	-1.869931825	7.58E-02	1.02E-01	-6.153851583
Mthfd1	-0.526030977	6.274285845	-3.071553566	5.87E-03	9.84E-03	-3.778918217
Tmpo	-0.529173407	8.733536691	-7.062304124	6.33E-07	2.27E-06	5.407810378
Alox5ap	-0.529281475	10.32930322	-4.61164432	1.57E-04	3.58E-04	-0.198893082
Lat2	-0.529597317	7.602517868	-2.807653131	1.07E-02	1.70E-02	-4.350748114
Nop2	-0.530549604	7.336362871	-4.54470564	1.84E-04	4.14E-04	-0.357796573
Pik3ap1	-0.530833726	8.427800985	-5.256059985	3.47E-05	8.98E-05	1.324347088
Vta1	-0.531078832	8.707782962	-7.032383781	6.74E-07	2.41E-06	5.343256878
Hipk2	-0.531346873	8.498023457	-5.48502656	2.05E-05	5.56E-05	1.860026891
Nck2	-0.531780731	8.409463978	-3.523262273	2.06E-03	3.79E-03	-2.75946459
Isyna1	-0.532179516	6.910294819	-4.454989631	2.27E-04	5.02E-04	-0.570777037
Lrrc25	-0.533668661	7.484836921	-2.760208461	1.19E-02	1.87E-02	-4.451206889
Aars	-0.533843478	7.447221366	-4.844151129	9.07E-05	2.18E-04	0.352516968

Exoc6	-0.535953503	8.708559095	-6.479014046	2.21E-06	7.20E-06	4.129001495
BC035044	-0.537312249	7.862857555	-2.810068801	1.06E-02	1.69E-02	-4.345612613
Kcnip3	-0.537502248	7.497627392	-3.01839099	6.63E-03	1.10E-02	-3.895761091
Thumpd3	-0.538645075	8.146112104	-3.362116466	3.00E-03	5.35E-03	-3.127987992
Mocos	-0.539671069	5.191339313	-3.569594047	1.85E-03	3.44E-03	-2.6526945
Cpa3	-0.54036173	5.410089988	-1.189371194	2.48E-01	2.98E-01	-7.128255669
Pprc1	-0.540549482	7.39785849	-3.468414566	2.34E-03	4.27E-03	-2.885410309
Lsm6	-0.540603783	8.222976128	-3.261110793	3.80E-03	6.61E-03	-3.356436459
Erlin1	-0.54094641	8.906676362	-7.743246744	1.56E-07	6.24E-07	6.845365888
Slc31a1	-0.541136779	6.533158338	-2.620765555	1.61E-02	2.47E-02	-4.741770001
Cuta	-0.541458865	9.782224325	-5.7209843	1.19E-05	3.36E-05	2.407620149
Cct8	-0.542553433	9.589623708	-7.37475703	3.30E-07	1.24E-06	6.074995177
Prc1	-0.542714865	6.137332334	-3.146898104	4.94E-03	8.41E-03	-3.612042389
Nat10	-0.542730736	6.550623892	-4.563601975	1.76E-04	3.98E-04	-0.312936873
Lima1	-0.545355107	7.392467967	-3.819030757	1.03E-03	1.98E-03	-2.07277477
Vmn2r90	-0.545654569	4.203111535	-1.862477524	7.68E-02	1.03E-01	-6.166308621
Ssr3	-0.545955417	9.385962918	-5.779128805	1.05E-05	2.97E-05	2.54178381
Fkbp4	-0.548275818	8.024314456	-3.539525891	1.98E-03	3.66E-03	-2.722023965
Naa10	-0.548767695	7.4975416	-2.703314213	1.34E-02	2.10E-02	-4.570629131
Chst11	-0.548803698	6.981830444	-3.151754124	4.89E-03	8.33E-03	-3.601237787
Eef1g	-0.54907779	4.687461081	-1.346137067	1.93E-01	2.37E-01	-6.935303379
Snhg12	-0.54926461	10.12606681	-6.812128659	1.08E-06	3.69E-06	4.864534703
Dlgap5	-0.549983703	6.945147684	-5.812992274	9.69E-06	2.77E-05	2.619771745
Trim30d	-0.550443963	9.099273696	-3.926144411	7.97E-04	1.57E-03	-1.821590957
Klf6	-0.550522453	9.597114818	-6.15663074	4.49E-06	1.38E-05	3.404567297
Prim1	-0.550758056	6.635813257	-2.341558808	2.93E-02	4.28E-02	-5.299808353
Mrpl12	-0.550955065	8.740856678	-4.236467508	3.82E-04	8.04E-04	-1.089054764
F2rl2	-0.55233518	6.720930103	-2.790608975	1.11E-02	1.76E-02	-4.386926249
Hap1	-0.552412708	6.347979654	-3.122128442	5.23E-03	8.85E-03	-3.667063303
Snrpa1	-0.552689618	8.645691761	-4.391556643	2.64E-04	5.73E-04	-0.72132549
Srek1	-0.553510756	8.839472597	-6.744746866	1.24E-06	4.22E-06	4.716856824
Map4	-0.553754347	8.686825366	-6.463132529	2.29E-06	7.39E-06	4.093597485
Eps8	-0.554228763	8.0735149	-4.423986796	2.45E-04	5.36E-04	-0.644363876
Larp1	-0.554590724	9.24847536	-7.526913622	2.42E-07	9.28E-07	6.395269701
Cpne2	-0.555245097	9.986521713	-5.504834158	1.95E-05	5.33E-05	1.906178516
Tbc1d10c	-0.558002994	7.803997125	-5.692412609	1.27E-05	3.57E-05	2.341576955
Tmem261	-0.558526005	8.255096062	-5.372225228	2.65E-05	7.06E-05	1.596607608
Pcdhga2	-0.558733028	2.794203224	-1.001308817	3.28E-01	3.82E-01	-7.331961237
Slc31a2	-0.55938639	8.120681415	-5.556288825	1.74E-05	4.78E-05	2.025915878
Hn1l	-0.559686657	7.756053008	-5.354758602	2.76E-05	7.32E-05	1.555731972
Rfx7	-0.560700595	8.683453923	-6.051184642	5.67E-06	1.71E-05	3.165074848
Mcpt4	-0.561374192	4.669062579	-0.718847457	4.80E-01	5.34E-01	-7.57725646
Ust	-0.561426362	5.898659448	-3.15692566	4.83E-03	8.25E-03	-3.589724786
2700060E02Rik	-0.561480562	10.32676512	-6.474238388	2.23E-06	7.25E-06	4.11835848
Serpina3g	-0.561847005	9.750266444	-3.977787982	7.05E-04	1.41E-03	-1.70012302
Gars	-0.562121508	8.946203026	-7.457821994	2.78E-07	1.06E-06	6.25021661
Snord57	-0.562318697	6.079316524	-1.553178365	1.36E-01	1.73E-01	-6.650299005
Ugt1a7c	-0.563100214	8.221771322	-2.459566123	2.28E-02	3.40E-02	-5.068110169
Hlcs	-0.564399692	6.19636401	-2.878307505	9.10E-03	1.47E-02	-4.199745903
Rars	-0.564749288	8.947238163	-6.97959895	7.54E-07	2.65E-06	5.229092692
Ier2	-0.565803415	8.318838128	-3.84155762	9.73E-04	1.89E-03	-2.020040516
Ndufb2	-0.566144463	8.857584385	-4.940679968	7.24E-05	1.76E-04	0.580989107
Cst3	-0.566815427	12.55567727	-9.81228334	3.21E-09	1.82E-08	10.8409561
1110008F13Rik	-0.569020875	9.31961942	-6.002969429	6.32E-06	1.88E-05	3.055167911
Hspa5	-0.569628673	11.09590273	-6.407387832	2.58E-06	8.28E-06	3.969091198
Pid1	-0.570054513	7.734121136	-3.189391463	4.48E-03	7.69E-03	-3.517300387
Asl	-0.570245217	8.374243822	-4.69086441	1.30E-04	3.02E-04	-0.010892029
Enpp4	-0.571392108	7.169675825	-3.338453517	3.17E-03	5.63E-03	-3.181695704
Vwa5a	-0.571486694	9.159014234	-7.805250152	1.38E-07	5.57E-07	6.973225468
Sdf2l1	-0.572001527	8.648768234	-4.399584977	2.59E-04	5.64E-04	-0.702274519
Stom	-0.572196494	9.310647888	-4.038532187	6.10E-04	1.24E-03	-1.556989785
Fam86	-0.572619448	6.753709821	-3.071961207	5.87E-03	9.84E-03	-3.778019329
Dph6	-0.574843714	6.120350995	-2.989798364	7.07E-03	1.17E-02	-3.958278178
Fnbp1l	-0.575801492	8.563502455	-3.976688662	7.07E-04	1.41E-03	-1.70271088
Ppp5c	-0.576082705	7.861211574	-4.760946028	1.10E-04	2.61E-04	0.155330238
Pold4	-0.576604792	9.388089589	-5.667006924	1.35E-05	3.77E-05	2.28278877
Eif3b	-0.577163205	8.430748391	-6.891299008	9.09E-07	3.15E-06	5.037319994
Lrrcc1	-0.577570122	8.145424556	-6.113240279	4.94E-06	1.51E-05	3.306164261
Cd180	-0.57795944	9.615059718	-4.902320367	7.92E-05	1.91E-04	0.490238768
Pdia3	-0.578840238	10.67834968	-6.476827037	2.22E-06	7.22E-06	4.124127872
Sgsh	-0.581579038	6.84311884	-4.040715029	6.07E-04	1.23E-03	-1.551841485
Pdia6	-0.582981481	9.012691465	-3.807889151	1.05E-03	2.03E-03	-2.098837351
Man1a2	-0.583263125	7.865227131	-7.019307911	6.93E-07	2.46E-06	5.315009396
Rnf213	-0.583730416	8.891174481	-4.997240935	6.34E-05	1.57E-04	0.714686089
Ccdc101	-0.587095624	7.013028461	-2.285577895	3.30E-02	4.77E-02	-5.407422162
Map2k3	-0.587138197	7.959959013	-5.074134811	5.30E-05	1.32E-04	0.896202826

Mrps28	-0.588880035	7.870592336	-1.959726117	6.37E-02	8.74E-02	-6.001061796
Zfp710	-0.58989196	7.380354096	-4.823843545	9.52E-05	2.28E-04	0.304409315
Ccdc12	-0.590408327	8.270121849	-4.972506321	6.72E-05	1.65E-04	0.656236691
Supt3	-0.59041825	8.177077852	-5.341253079	2.85E-05	7.55E-05	1.524110853
Efr3a	-0.590907184	8.622091223	-8.744747597	2.22E-08	1.06E-07	8.848417591
Psmb9	-0.592785032	11.91022057	-8.813773517	1.95E-08	9.51E-08	8.981629296
Gm8203	-0.593042289	8.44166168	-3.833237054	9.92E-04	1.92E-03	-2.039524555
Scarb1	-0.59331892	7.98213756	-3.182262165	4.55E-03	7.81E-03	-3.53322604
Tnfaip2	-0.5943573	6.923033421	-3.09843566	5.52E-03	9.31E-03	-3.719546638
Gpsm3	-0.59559784	8.561640213	-6.555122011	1.87E-06	6.19E-06	4.298246448
Ankfy1	-0.596364109	8.621880545	-7.306085158	3.81E-07	1.42E-06	5.929451049
Arl3	-0.596992368	8.93865869	-6.006473369	6.27E-06	1.87E-05	3.063163469
Slc37a3	-0.597042893	6.666831595	-4.540318968	1.86E-04	4.17E-04	-0.368210608
Mcm6	-0.598164631	8.373850819	-4.045517191	6.00E-04	1.22E-03	-1.540514321
Dimt1	-0.599409994	7.279978023	-3.221762675	4.16E-03	7.17E-03	-3.444838406
Serpinb6b	-0.600449226	8.883593062	-3.838354911	9.81E-04	1.90E-03	-2.027541035
Prr11	-0.602366939	7.336073471	-3.438539762	2.51E-03	4.56E-03	-2.95379473
Cdca3	-0.602531242	6.821949373	-2.778671964	1.14E-02	1.80E-02	-4.41220475
Farsb	-0.602548188	7.83198226	-3.744012895	1.23E-03	2.33E-03	-2.247996106
Sqle	-0.60329075	7.309741972	-3.900640256	8.46E-04	1.66E-03	-1.881495375
Tlr6	-0.604338843	7.221508636	-3.502870888	2.16E-03	3.97E-03	-2.806347424
Coro7	-0.604985873	8.811223326	-5.108300371	4.89E-05	1.24E-04	0.976756129
Nars	-0.605220862	9.679719553	-8.78136145	2.07E-08	1.00E-07	8.91915472
Hist1h2af	-0.605359742	7.634328566	-2.834153932	1.00E-02	1.61E-02	-4.294302905
Plbd1	-0.605670125	11.08330523	-4.662892039	1.39E-04	3.20E-04	-0.077264506
Ftsj3	-0.605987286	7.27137769	-4.042649217	6.04E-04	1.23E-03	-1.547279377
Dck	-0.606226969	6.87177999	-3.587411164	1.77E-03	3.31E-03	-2.611547741
Tor1aip2	-0.610549328	8.971165811	-8.499131321	3.54E-08	1.60E-07	8.369384757
Psat1	-0.614807434	5.968781552	-3.336520629	3.19E-03	5.65E-03	-3.186077826
Zfp189	-0.615289138	6.913289242	-3.647867686	1.54E-03	2.89E-03	-2.471586253
Adam19	-0.615905646	8.950181306	-7.735987687	1.58E-07	6.32E-07	6.830363388
Atad2	-0.616182676	7.506059793	-6.771651291	1.17E-06	4.00E-06	4.775890235
Cdca8	-0.61626676	7.372109183	-3.909137536	8.29E-04	1.63E-03	-1.861543212
Vcam1	-0.616936401	7.210051655	-3.776406758	1.14E-03	2.18E-03	-2.172409476
Irf7	-0.617166933	8.206636924	-3.259083204	3.81E-03	6.63E-03	-3.361000201
Spag5	-0.617713037	6.289914974	-3.420117716	2.62E-03	4.74E-03	-2.995884008
Rnf11	-0.617790951	7.894148149	-5.416840079	2.39E-05	6.42E-05	1.700912283
Tcirg1	-0.618317138	8.042913025	-6.742396913	1.25E-06	4.24E-06	4.711696296
Gnaq	-0.618431205	8.267553726	-6.162773073	4.43E-06	1.37E-05	3.418480421
Nop58	-0.619002773	8.659630321	-4.947219491	7.13E-05	1.74E-04	0.596454165
Plat	-0.620583453	5.719035877	-2.531317195	1.96E-02	2.95E-02	-4.924193488
Fchsd2	-0.622858622	9.393192101	-8.113486897	7.47E-08	3.17E-07	7.601286824
Aldh9a1	-0.624407582	7.939045981	-6.119325436	4.87E-06	1.49E-05	3.319976939
Cttnbp2nl	-0.624442023	8.293471147	-3.651350004	1.52E-03	2.87E-03	-2.463508864
Usmg5	-0.624845828	11.07426346	-5.898517481	7.99E-06	2.32E-05	2.816234781
Farsa	-0.624947669	7.647920877	-5.992136873	6.48E-06	1.92E-05	3.030441186
2610524H06Rik	-0.624966001	7.856410321	-2.707051143	1.33E-02	2.08E-02	-4.562821166
Hyou1	-0.625381942	7.762261065	-3.75121198	1.20E-03	2.30E-03	-2.231208473
Cct3	-0.625934586	8.072976821	-7.076214295	6.15E-07	2.21E-06	5.437782488
Noc4l	-0.626096421	6.679838326	-5.596872654	1.58E-05	4.38E-05	2.120197791
LOC102638508	-0.626530104	6.202001187	-2.782558128	1.13E-02	1.79E-02	-4.403980572
Ivns1abp	-0.626647777	10.1256567	-9.859753647	2.95E-09	1.69E-08	10.92627198
Itgb7	-0.627067993	8.179152493	-4.13750329	4.83E-04	9.99E-04	-1.323267907
Foxm1	-0.627764591	6.582058808	-4.290365916	3.36E-04	7.17E-04	-0.961333265
Dusp2	-0.629681785	8.301216617	-4.738515355	1.16E-04	2.73E-04	0.102140035
1700025G04Rik	-0.630474621	8.928562228	-7.249035467	4.28E-07	1.58E-06	5.808069682
Cables2	-0.630778385	7.750463507	-5.469295169	2.12E-05	5.73E-05	1.823350127
Il18	-0.63211878	7.585746911	-4.78085178	1.05E-04	2.50E-04	0.202522536
Ezr	-0.63317024	9.089711644	-8.299307009	5.20E-08	2.29E-07	7.973836084
Gm16617	-0.634237547	7.748048042	-3.321332952	3.30E-03	5.84E-03	-3.220483964
Src	-0.637834669	6.673639372	-4.951173063	7.06E-05	1.72E-04	0.605802931
LOC102639837	-0.638097875	6.959249835	-4.429075848	2.42E-04	5.31E-04	-0.632285481
Tsr1	-0.638649744	7.079606875	-4.340826657	2.98E-04	6.41E-04	-0.841678459
2810004N23Rik	-0.640894504	8.139087994	-6.049964954	5.69E-06	1.71E-05	3.162297611
Asxl2	-0.641150078	8.947993223	-5.631322318	1.46E-05	4.07E-05	2.200116554
Mcm2	-0.642591918	7.157555036	-4.664521855	1.39E-04	3.20E-04	-0.073396955
Oxct1	-0.644820421	8.484570179	-7.571655184	2.21E-07	8.53E-07	6.488865418
Flnb	-0.646167657	8.919780202	-8.399825644	4.28E-08	1.91E-07	8.173466891
Irgm1	-0.647223828	8.067560947	-4.272088319	3.51E-04	7.45E-04	-1.004655994
Mfsd10	-0.648045819	7.410126678	-5.337343771	2.87E-05	7.59E-05	1.514955339
Hcls1	-0.648622427	9.757830788	-4.803659076	9.98E-05	2.38E-04	0.256580574
Arhgap22	-0.649416798	6.822421678	-5.325323398	2.96E-05	7.78E-05	1.486797011
Tiam1	-0.6500059	7.418402708	-5.93913654	7.29E-06	2.13E-05	2.909284395
Prdx1	-0.650869902	8.674054576	-3.775943999	1.14E-03	2.18E-03	-2.1734901
Pcbd2	-0.650985887	9.273771874	-8.352245409	4.69E-08	2.08E-07	8.079137938
Cldn25	-0.651074272	8.85698214	-5.22034329	3.77E-05	9.71E-05	1.240451232

Denr	-0.652292349	9.323021625	-5.221487945	3.76E-05	9.69E-05	1.243141225
Dusp1	-0.652457355	11.40491515	-6.242955494	3.71E-06	1.16E-05	3.599717096
Hist1h2ab	-0.653045455	6.91739219	-1.414390309	1.72E-01	2.14E-01	-6.845032345
Eif3g	-0.65355546	10.57812097	-9.344596258	7.36E-09	3.91E-08	9.985647437
Snord118	-0.653753868	8.47758397	-4.194323045	4.22E-04	8.83E-04	-1.188848592
Dynll2	-0.653965261	7.75610083	-3.794313881	1.09E-03	2.10E-03	-2.130574996
Atp2a2	-0.654926081	9.080327385	-8.531812662	3.33E-08	1.51E-07	8.433578287
Mpp6	-0.65493769	9.533526094	-6.091889349	5.18E-06	1.57E-05	3.257667927
Synrg	-0.655728074	8.385033926	-6.650077858	1.52E-06	5.11E-06	4.508420523
A630072M18Rik	-0.655892037	6.303844935	-3.967188273	7.23E-04	1.44E-03	-1.725071429
Hist1h2an	-0.656101444	8.371550245	-3.145270806	4.96E-03	8.43E-03	-3.615661808
Mrps5	-0.656694592	6.988145611	-3.311478124	3.38E-03	5.96E-03	-3.24278376
Pomp	-0.65731278	10.77164405	-5.399653595	2.49E-05	6.66E-05	1.660750004
Ndc80	-0.657394823	7.265193978	-2.916321227	8.35E-03	1.36E-02	-4.117840388
Dna2	-0.657510853	7.774422339	-3.877442326	8.94E-04	1.74E-03	-1.935932361
Copg2	-0.657910767	8.452742918	-6.135433713	4.70E-06	1.45E-05	3.356521484
Ercc6l	-0.658263375	6.825063585	-3.758532259	1.18E-03	2.26E-03	-2.214132046
Cenpn	-0.660538663	7.179307027	-3.87705597	8.95E-04	1.74E-03	-1.936838576
Snrpf	-0.661859176	6.760220125	-2.933682278	8.03E-03	1.31E-02	-4.080285067
Phyh	-0.661919377	7.340560324	-5.933970241	7.38E-06	2.15E-05	2.897458874
Gdap10	-0.663465021	9.220410884	-6.131140836	4.75E-06	1.46E-05	3.346785098
Ranbp1	-0.664509311	7.393608866	-5.921230292	7.59E-06	2.21E-05	2.868285763
AF251705	-0.665440469	7.799344029	-1.695069384	1.05E-01	1.37E-01	-6.436473239
Fads1	-0.667057344	7.118116719	-5.079608665	5.23E-05	1.31E-04	0.909112961
Cptp	-0.667152243	7.274040361	-6.82824906	1.04E-06	3.57E-06	4.899780743
Tmem243	-0.668625849	9.158332897	-5.555980775	1.74E-05	4.78E-05	2.025199692
Fos	-0.66965805	10.34011871	-8.059206903	8.31E-08	3.49E-07	7.491599631
Clspn	-0.673836722	6.121162963	-3.546403277	1.95E-03	3.61E-03	-2.706178858
Pafah1b3	-0.67694517	9.113398797	-5.111565711	4.85E-05	1.23E-04	0.984451578
Rpsa	-0.677043736	8.719386281	-4.740719853	1.16E-04	2.72E-04	0.107368122
Ccl19	-0.677991802	6.66566471	-2.995663727	6.98E-03	1.16E-02	-3.945472674
Stil	-0.678198799	6.086438878	-5.090290375	5.10E-05	1.28E-04	0.934301254
Tceb3	-0.679418708	9.165184701	-9.550224466	5.09E-09	2.80E-08	10.36502705
Coprs	-0.680310415	6.703758206	-3.688480122	1.40E-03	2.64E-03	-2.377283551
Lars	-0.680396424	8.631011426	-6.982307734	7.49E-07	2.64E-06	5.234959999
Trib1	-0.681130126	8.149807637	-4.662053655	1.39E-04	3.21E-04	-0.079254004
Trim30a	-0.682275764	8.528502272	-3.981397696	6.99E-04	1.40E-03	-1.691624903
Tet3	-0.683095915	8.021395469	-6.519755476	2.02E-06	6.64E-06	4.219686792
Mrps18b	-0.68461647	7.325212275	-3.86384176	9.23E-04	1.79E-03	-1.967824588
Cenpf	-0.684630266	5.955520099	-4.923886291	7.53E-05	1.83E-04	0.541266263
Kntc1	-0.684812121	5.508194284	-4.769213129	1.08E-04	2.56E-04	0.174931024
Tbrg1	-0.685134023	9.726913034	-9.315826484	7.76E-09	4.10E-08	9.932146867
Gins2	-0.686287471	7.687115237	-4.73367488	1.18E-04	2.76E-04	0.090660198
Fgd3	-0.686344859	6.787983517	-3.897912199	8.52E-04	1.67E-03	-1.887899663
Eif1a	-0.687522103	7.732733888	-7.595392769	2.10E-07	8.16E-07	6.538415118
Tmtc4	-0.687992554	6.620130347	-5.296222202	3.16E-05	8.26E-05	1.418583609
Rtcb	-0.689337363	10.39745546	-10.16220814	1.75E-09	1.07E-08	11.46350523
Btk	-0.689473489	7.867364827	-3.919756735	8.09E-04	1.59E-03	-1.836599711
Syvn1	-0.689792633	7.988096402	-5.503130555	1.96E-05	5.35E-05	1.90221039
Mamld1	-0.690107174	5.965600832	-5.259924614	3.44E-05	8.90E-05	1.333419781
Nsun2	-0.691512982	8.014199318	-6.544393115	1.91E-06	6.33E-06	4.274430313
Cep128	-0.692449407	8.110778769	-5.888711277	8.17E-06	2.36E-05	2.79374571
Pld4	-0.693526335	9.764413769	-5.428923756	2.33E-05	6.26E-05	1.729136454
Chaf1a	-0.694249464	6.586308494	-4.020788574	6.36E-04	1.29E-03	-1.598826673
Gm19412	-0.695269555	6.960140369	-4.340017089	2.98E-04	6.42E-04	-0.843598656
Impdh2	-0.696901595	8.782424382	-2.761889705	1.18E-02	1.86E-02	-4.447660371
Wdr43	-0.69718946	9.138296057	-9.707740345	3.85E-09	2.14E-08	10.65210154
Ampd3	-0.702352391	7.23494351	-9.158579071	1.03E-08	5.29E-08	9.637886431
Usp10	-0.703015582	7.521251161	-3.823962808	1.01E-03	1.96E-03	-2.061233523
Tma16	-0.703381027	5.73563547	-2.227285866	3.72E-02	5.34E-02	-5.517822546
Mtmr14	-0.704508164	8.508547318	-8.038495903	8.66E-08	3.63E-07	7.449644698
Sema4c	-0.704826622	6.259394231	-3.818953839	1.03E-03	1.98E-03	-2.072954742
Klhl6	-0.705864678	8.093673804	-4.756275929	1.12E-04	2.64E-04	0.144256961
Pbk	-0.708846654	6.318359666	-3.264473259	3.77E-03	6.56E-03	-3.348866156
Bcl6	-0.709122183	10.3768874	-10.07587038	2.03E-09	1.21E-08	11.31126045
Tcf7l2	-0.709457616	8.288708339	-6.137212498	4.68E-06	1.44E-05	3.360555235
Eef1e1	-0.710526919	7.426649589	-3.895116428	8.57E-04	1.68E-03	-1.894462216
Zdhhc23	-0.71082107	7.24987174	-4.842152237	9.12E-05	2.19E-04	0.347782284
Ptpre	-0.710893322	7.945277217	-5.16311275	4.31E-05	1.10E-04	1.105852047
Slc1a5	-0.711103928	8.40187916	-6.922069709	8.51E-07	2.96E-06	5.104261465
Socs1	-0.712053886	6.977441914	-4.728493695	1.19E-04	2.78E-04	0.078371741
Noa1	-0.712058294	7.191941466	-5.349323115	2.80E-05	7.41E-05	1.543007204
Tiparp	-0.712299931	8.440486327	-6.120002302	4.87E-06	1.49E-05	3.321513103
Nupl1	-0.712532605	8.967656366	-11.72947423	1.38E-10	1.11E-09	14.08052911
Sp140	-0.712636139	7.380392817	-3.469778318	2.34E-03	4.26E-03	-2.882284908
Snord72	-0.715792248	7.373155319	-4.234020426	3.84E-04	8.09E-04	-1.094851127

Eif2s1	-0.716446493	8.71498893	-7.906050878	1.13E-07	4.63E-07	7.180002439
Znfx1	-0.71856004	8.326096426	-8.373387971	4.51E-08	2.00E-07	8.121090332
Rgs2	-0.721030645	10.7464812	-9.316754037	7.74E-09	4.10E-08	9.933873375
Anln	-0.721970971	6.283065888	-5.30582597	3.09E-05	8.10E-05	1.441101474
Snora68	-0.722939549	6.266093098	-3.118433698	5.27E-03	8.92E-03	-3.675257209
Ggta1	-0.724761465	7.996411036	-5.969739958	6.81E-06	2.01E-05	2.979278308
Gnb4	-0.725620296	8.434280596	-3.916289944	8.15E-04	1.60E-03	-1.844743959
Gm5751	-0.727741764	5.260880427	-2.270062749	3.41E-02	4.92E-02	-5.436974168
Aspm	-0.728164673	5.80728042	-4.370566155	2.78E-04	6.00E-04	-0.77112997
A030001D20Rik	-0.728420543	6.185188271	-3.688334453	1.40E-03	2.64E-03	-2.377622183
Irf1	-0.729573952	8.778343769	-7.940244246	1.05E-07	4.34E-07	7.249838744
Ranbp2	-0.7296229	9.416337307	-8.113805558	7.47E-08	3.17E-07	7.601929611
Eif2b2	-0.730235552	7.952030005	-6.213194674	3.96E-06	1.23E-05	3.53253273
Sik1	-0.731190908	8.41514791	-4.548937966	1.82E-04	4.10E-04	-0.34774899
LOC102635268	-0.731416018	5.962212061	-2.269237891	3.41E-02	4.93E-02	-5.43854192
Map3k4	-0.73470749	6.651301177	-4.502770837	2.03E-04	4.52E-04	-0.457350588
Rnpep	-0.735085977	7.311966581	-6.125834574	4.80E-06	1.47E-05	3.334747522
Ddx39	-0.736225362	8.15897623	-4.133468627	4.87E-04	1.01E-03	-1.332806736
Nfatc2	-0.736350302	8.024242093	-7.675197253	1.79E-07	7.07E-07	6.704451982
Ahnak	-0.736975209	8.614059989	-5.087593638	5.13E-05	1.29E-04	0.92794272
Mprip	-0.742526957	8.903848081	-8.268603844	5.52E-08	2.41E-07	7.912593991
Btg2	-0.74400064	10.48640989	-12.28894165	5.93E-11	5.26E-10	14.95172253
Snora70	-0.744032055	6.649577071	-2.990456713	7.06E-03	1.17E-02	-3.956841333
Ept1	-0.744666658	7.671363457	-6.544749893	1.91E-06	6.33E-06	4.275222517
Trerf1	-0.744785817	7.666949103	-7.440004581	2.89E-07	1.09E-06	6.212708134
Eif4ebp1	-0.745363086	6.91635658	-5.298066621	3.15E-05	8.23E-05	1.422908699
Klrb1b	-0.747206578	6.37398408	-4.142448962	4.77E-04	9.88E-04	-1.311574107
Tnni2	-0.747408703	9.291754457	-8.382528778	4.43E-08	1.97E-07	8.139209885
Trpv2	-0.748587392	8.86267612	-10.77516749	6.29E-10	4.33E-09	12.5194523
Fignl1	-0.751549906	5.726990431	-3.252413611	3.87E-03	6.73E-03	-3.376005874
Icos	-0.753597654	8.472618963	-5.967230208	6.85E-06	2.02E-05	2.973541845
Cenph	-0.753616768	6.515609191	-4.398000819	2.60E-04	5.66E-04	-0.706033754
Hist2h2ac	-0.754779802	8.159497296	-1.824836268	8.25E-02	1.10E-01	-6.228667687
Hiat1	-0.756332597	8.398466657	-8.302249388	5.17E-08	2.28E-07	7.979698577
Znrd1	-0.756956045	8.254223157	-6.896563881	8.99E-07	3.12E-06	5.048782214
Dpagt1	-0.757418351	8.444745631	-9.293094483	8.08E-09	4.25E-08	9.889800707
Hspa1b	-0.758129825	7.90752166	-1.132826169	2.70E-01	3.21E-01	-7.192764296
Nudc	-0.758332442	8.840562761	-6.594369733	1.72E-06	5.71E-06	4.385250148
Rnu3a	-0.758637923	8.011259141	-2.731818464	1.26E-02	1.98E-02	-4.510943498
Anxa6	-0.75884564	9.689094499	-4.638950683	1.47E-04	3.37E-04	-0.134081622
Gm8221	-0.760412767	7.407882758	-3.611020749	1.68E-03	3.14E-03	-2.556951684
Prkab2	-0.761451562	7.402176657	-6.392149863	2.67E-06	8.55E-06	3.934993244
Morc2a	-0.76349577	7.940259848	-7.210614499	4.64E-07	1.71E-06	5.7260842
Dhfr	-0.763514156	5.5654861	-4.180071317	4.36E-04	9.09E-04	-1.222578328
Tnfrsf1a	-0.763516033	8.100170495	-3.937275597	7.76E-04	1.53E-03	-1.79542836
2810417H13Rik	-0.763654294	6.605113048	-2.912658651	8.42E-03	1.37E-02	-4.125751462
Tmem50b	-0.764147093	8.526738975	-6.664997853	1.48E-06	4.96E-06	4.541343941
Alg8	-0.764754828	8.296483449	-7.02979156	6.78E-07	2.41E-06	5.337658711
Mapk14	-0.766670749	7.920838312	-4.981987649	6.57E-05	1.62E-04	0.678644941
Mast4	-0.766844046	5.975921329	-3.750795841	1.21E-03	2.30E-03	-2.232179036
Snx2	-0.76704339	9.013788488	-9.092783677	1.16E-08	5.90E-08	9.513833378
Iars	-0.767202831	8.07428721	-9.173390149	1.00E-08	5.17E-08	9.665736008
Amica1	-0.768312559	8.68990629	-4.933502081	7.36E-05	1.79E-04	0.564012347
Tcf19	-0.76844147	6.22572444	-4.472457344	2.18E-04	4.84E-04	-0.529312556
Preb	-0.76980955	8.275887029	-6.321144	3.12E-06	9.89E-06	3.775745985
Wdhd1	-0.77226202	7.084076548	-5.437434461	2.28E-05	6.14E-05	1.74900833
Phf11b	-0.772392827	10.6584096	-7.126571005	5.53E-07	2.01E-06	5.546076739
Man2b1	-0.77272895	10.38462222	-11.52102311	1.91E-10	1.48E-09	13.74781094
Phf11a	-0.77331864	8.857535434	-6.501913829	2.10E-06	6.89E-06	4.179998012
Gmpr2	-0.773787281	8.833886938	-8.499357653	3.54E-08	1.60E-07	8.369829803
Snord15b	-0.774038424	7.552459177	-2.641268621	1.54E-02	2.37E-02	-4.699505686
Krtcap3	-0.774098937	7.046032349	-5.968719953	6.82E-06	2.02E-05	2.97694699
Rbbp7	-0.775245747	9.798248904	-9.82592228	3.13E-09	1.78E-08	10.86549659
Arhgap4	-0.776041448	7.375639023	-6.51957472	2.02E-06	6.64E-06	4.219284892
Tpx2	-0.776814231	7.015767367	-4.786209528	1.04E-04	2.47E-04	0.215222821
Knstrn	-0.777873254	6.958623656	-6.889772225	9.12E-07	3.16E-06	5.033995356
Cpeb2	-0.778165666	7.974718727	-7.139502224	5.38E-07	1.96E-06	5.573832957
Nedd4	-0.77824148	9.783911036	-8.447084548	3.91E-08	1.75E-07	8.266864264
Fastkd1	-0.780308929	5.453159447	-4.068928057	5.68E-04	1.16E-03	-1.485272208
Esyt2	-0.780517909	9.266199166	-9.266270524	8.48E-09	4.44E-08	9.839748281
Ckap2	-0.782215323	5.61373894	-4.655472108	1.42E-04	3.25E-04	-0.094872481
Ptrhd1	-0.782681645	7.532434452	-5.458005908	2.18E-05	5.87E-05	1.797017676
Rpl14	-0.78344339	8.07612916	-3.902786427	8.42E-04	1.65E-03	-1.876456634
Tap1	-0.783625048	10.17986499	-12.55454181	4.01E-11	3.69E-10	15.35455259
Lgals3bp	-0.783731995	8.529724348	-5.967335062	6.85E-06	2.02E-05	2.97378152
Rgs14	-0.786599608	6.240981566	-4.575996573	1.71E-04	3.87E-04	-0.283512959

Asf1b	-0.786708637	6.814742429	-3.972277964	7.14E-04	1.43E-03	-1.713092971
Gm10277	-0.787284626	6.435102868	-4.042070445	6.05E-04	1.23E-03	-1.548644534
Inpp5d	-0.787325395	9.803473566	-9.946804085	2.54E-09	1.48E-08	11.08201639
Mcm5	-0.787600076	7.500254275	-5.147239446	4.47E-05	1.13E-04	1.068484672
Plcg2	-0.788064588	8.459292796	-8.028073981	8.84E-08	3.70E-07	7.428511187
Tbc1d31	-0.789752807	6.270755568	-4.139235588	4.81E-04	9.95E-04	-1.319172117
Bub1b	-0.791770453	6.893234691	-5.318321093	3.00E-05	7.89E-05	1.470389009
Ube2t	-0.792056224	5.422890673	-2.940118767	7.92E-03	1.30E-02	-4.066338433
Mthfd2	-0.795604502	9.437257988	-7.547292264	2.32E-07	8.93E-07	6.437932956
Pank1	-0.796455694	6.629201178	-4.517750294	1.96E-04	4.38E-04	-0.421789237
Shcbp1	-0.796496524	5.881510491	-2.941525181	7.89E-03	1.29E-02	-4.063289342
Clip2	-0.797743575	7.562019024	-7.4076396	3.09E-07	1.17E-06	6.144467839
Ppm1m	-0.799368484	8.006289333	-7.596349596	2.10E-07	8.16E-07	6.540410832
Scimp	-0.799370638	7.886783447	-4.44709558	2.32E-04	5.11E-04	-0.589514925
Atpif1	-0.801104893	11.52985962	-10.18193191	1.70E-09	1.04E-08	11.49816175
Itpripl2	-0.802683663	9.173486037	-11.21739568	3.08E-10	2.28E-09	13.25501924
1190002F15Rik	-0.803002773	6.03606324	-3.327865244	3.25E-03	5.76E-03	-3.205691486
Il27	-0.80329522	7.038506616	-3.324648116	3.28E-03	5.80E-03	-3.212977816
Cep55	-0.804656303	6.104527312	-3.944565025	7.62E-04	1.51E-03	-1.778289769
Prkra	-0.804729152	7.520725724	-7.678894305	1.78E-07	7.02E-07	6.712123435
Rpf2	-0.808174935	8.357511886	-3.493981309	2.21E-03	4.05E-03	-2.826764456
Kifc1	-0.809072148	5.876097953	-2.137735665	4.47E-02	6.31E-02	-5.683985038
Pola1	-0.810044536	6.805403006	-4.669123656	1.37E-04	3.16E-04	-0.06247711
Srgap3	-0.810892875	7.257389319	-3.996662502	6.74E-04	1.36E-03	-1.655677078
Sptbn1	-0.814903713	8.13238509	-9.113650073	1.12E-08	5.71E-08	9.553235221
Hsd3b7	-0.815472203	7.185942374	-4.180353637	4.36E-04	9.09E-04	-1.221910246
Myo1f	-0.817485336	8.709383569	-6.811545699	1.08E-06	3.69E-06	4.863259492
Ncapg	-0.817634042	6.221170747	-4.733381591	1.18E-04	2.76E-04	0.089964606
Hnrnpll	-0.818249962	7.975221809	-9.643192584	4.32E-09	2.39E-08	10.53483118
Smpdl3a	-0.818691219	7.934927589	-7.967743226	9.96E-08	4.14E-07	7.305890006
Hirip3	-0.819806417	6.42377956	-7.908972716	1.12E-07	4.61E-07	7.18597604
Plp2	-0.820034798	10.66565248	-6.327826921	3.07E-06	9.76E-06	3.79075896
Fosb	-0.820207103	10.43459645	-10.1318869	1.85E-09	1.12E-08	11.4101384
Anpep	-0.820504783	9.698173041	-5.405415938	2.46E-05	6.58E-05	1.674218266
Kif14	-0.820704405	5.953497357	-4.692147218	1.30E-04	3.01E-04	-0.007848517
Hps5	-0.823799129	8.54779371	-8.646527629	2.67E-08	1.24E-07	8.657799493
Mki67	-0.824203134	8.862648692	-5.411634402	2.42E-05	6.49E-05	1.688749771
Fbxo4	-0.826148377	6.397679904	-5.05093376	5.59E-05	1.39E-04	0.841465448
Cd9	-0.826491299	9.027930774	-6.461610986	2.29E-06	7.41E-06	4.090203995
Rpa2	-0.827039232	7.274297115	-5.064294321	5.42E-05	1.35E-04	0.87298995
Pstpip1	-0.827532252	8.561788219	-10.02623641	2.21E-09	1.30E-08	11.22333724
Itgal	-0.828390003	8.400563837	-6.686898892	1.41E-06	4.74E-06	4.589622496
Snord38a	-0.830333811	5.351974523	-2.837344627	9.98E-03	1.60E-02	-4.287491166
Cited2	-0.831760828	8.808943968	-8.466423982	3.77E-08	1.70E-07	8.305000089
E2f7	-0.833818702	5.74246035	-5.815701898	9.63E-06	2.75E-05	2.62600722
Vkorc1l1	-0.834493015	8.553847502	-6.199862794	4.08E-06	1.27E-05	3.502403994
Trio	-0.83487308	8.673700567	-10.34729523	1.28E-09	8.09E-09	11.78692516
Trap1	-0.83559405	8.543655506	-10.94297864	4.79E-10	3.40E-09	12.80110834
Notch2	-0.836356484	9.225647123	-12.16231793	7.16E-11	6.25E-10	14.75727069
Tacc3	-0.836455435	7.704170081	-6.075006151	5.38E-06	1.63E-05	3.219284536
Cdca5	-0.836719468	6.465459127	-3.944076309	7.63E-04	1.51E-03	-1.779438954
Gmds	-0.838073787	6.035445431	-3.321209913	3.30E-03	5.84E-03	-3.220762504
H2-Ob	-0.838726587	7.88379445	-4.715856177	1.23E-04	2.86E-04	0.048396331
Prkar2b	-0.839045763	6.926020613	-4.739968254	1.16E-04	2.72E-04	0.105585677
Spop	-0.84097552	10.86286836	-12.17680984	7.01E-11	6.13E-10	14.77960507
Tor1a	-0.84121066	8.438969227	-5.963712562	6.90E-06	2.04E-05	2.965500559
Snora17	-0.841896751	6.176337473	-3.247470705	3.92E-03	6.80E-03	-3.387120378
Usp22	-0.843574972	8.239127113	-7.566221191	2.23E-07	8.62E-07	6.477512057
Tbca	-0.845661682	9.835160253	-8.683643064	2.49E-08	1.17E-07	8.729977784
Atf3	-0.847012399	9.381658997	-6.764132837	1.19E-06	4.06E-06	4.759402418
Kif11	-0.847033098	7.209686623	-7.612101071	2.03E-07	7.95E-07	6.573247251
Sfxn1	-0.848724364	6.838366897	-6.463945415	2.28E-06	7.39E-06	4.095410347
Lpcat1	-0.849197166	8.816506236	-8.135993105	7.15E-08	3.04E-07	7.64665224
Ncaph	-0.849686015	6.332242332	-4.366755086	2.80E-04	6.05E-04	-0.780171675
Ero1l	-0.85350868	8.079968	-9.104868628	1.14E-08	5.78E-08	9.536660042
Rgs10	-0.857187316	7.703669139	-8.632430467	2.75E-08	1.28E-07	8.630337786
Ckap2l	-0.85735132	5.816784486	-4.663216898	1.39E-04	3.20E-04	-0.076493612
Phf10	-0.857426435	8.555098348	-9.53039594	5.28E-09	2.89E-08	10.32867271
Vdac2	-0.858746901	8.867233904	-8.076922197	8.03E-08	3.39E-07	7.527440987
Olfr98	-0.862377856	7.521583878	-4.513438509	1.98E-04	4.42E-04	-0.432025496
Tbc1d9	-0.862759636	9.653421178	-10.5037171	9.85E-10	6.44E-09	12.05714952
Mbp	-0.863114631	7.195870688	-10.51649106	9.65E-10	6.32E-09	12.07909258
LOC102632809	-0.865628403	6.950672693	-3.525806105	2.05E-03	3.77E-03	-2.753611202
Creld2	-0.865645826	8.144414742	-7.069398375	6.24E-07	2.24E-06	5.423099409
Nup210	-0.866955311	9.562463156	-14.37155753	3.24E-12	4.24E-11	17.93966477
Sh2d3c	-0.867092913	7.619064396	-6.973771953	7.63E-07	2.68E-06	5.216468065

Gins1	-0.867853144	6.392274297	-5.821124718	9.51E-06	2.72E-05	2.638484235
Bub1	-0.869123215	5.929172823	-5.604424287	1.56E-05	4.31E-05	2.137725538
Rbpj	-0.869555704	7.094881477	-4.994760666	6.38E-05	1.57E-04	0.708826331
Cdca7l	-0.870128788	6.795128755	-5.682041628	1.30E-05	3.65E-05	2.317585871
Nudt9	-0.872719101	8.026309762	-7.65422288	1.87E-07	7.35E-07	6.660895504
Usp6nl	-0.872818966	8.452063577	-8.842387792	1.85E-08	9.04E-08	9.036670752
Stat1	-0.873787161	10.24231808	-10.14043711	1.82E-09	1.11E-08	11.42519817
Psca	-0.874398349	4.527116622	-3.27902971	3.64E-03	6.35E-03	-3.316065138
Fam188a	-0.876357128	8.711999557	-12.31080119	5.74E-11	5.11E-10	14.98513313
H2afx	-0.876758642	10.35689016	-6.21120905	3.98E-06	1.24E-05	3.528046679
Gabbr1	-0.877787555	6.837708429	-5.22959605	3.69E-05	9.52E-05	1.262193246
Slc12a9	-0.877830768	6.968089334	-6.71787268	1.32E-06	4.45E-06	4.657799758
Rpn1	-0.87810676	7.897951793	-7.813386019	1.35E-07	5.50E-07	6.989964918
Snx10	-0.878362862	8.187516658	-11.36361543	2.44E-10	1.85E-09	13.49355747
Birc5	-0.882199124	6.453424096	-4.709321835	1.25E-04	2.90E-04	0.03289598
Top2a	-0.886306591	8.384721952	-7.43816453	2.90E-07	1.10E-06	6.208832155
Ruvbl1	-0.887794826	8.630840393	-4.729158052	1.19E-04	2.78E-04	0.07994746
Polg2	-0.888317032	7.408760038	-6.177079633	4.29E-06	1.33E-05	3.450870274
Trim7	-0.888732152	7.669770897	-9.677920461	4.06E-09	2.26E-08	10.5979882
Txndc17	-0.890810165	10.44803205	-10.50052992	9.91E-10	6.46E-09	12.05167169
Mylpf	-0.893479971	6.282369728	-4.63158312	1.50E-04	3.42E-04	-0.151567664
Rasgrp4	-0.895154616	8.764754175	-6.948623106	8.05E-07	2.82E-06	5.161931447
Sipa1l3	-0.895237005	8.362664291	-9.434460356	6.26E-09	3.38E-08	10.15209243
Bcl2a1a	-0.896025303	10.52265101	-6.474130686	2.23E-06	7.25E-06	4.118118423
Hsd17b11	-0.899051125	9.553483754	-8.649906434	2.66E-08	1.24E-07	8.664377665
Cyp27a1	-0.90045165	9.232841609	-10.45653837	1.07E-09	6.89E-09	11.97594382
Scgb2b20	-0.900615541	3.033388882	-1.444003871	1.64E-01	2.05E-01	-6.804721998
AB124611	-0.900738271	8.516379559	-4.753383475	1.12E-04	2.65E-04	0.13739839
Aug-02	-0.901344473	7.676843231	-8.290583954	5.29E-08	2.32E-07	7.956449275
1700099I09Rik	-0.902048156	5.016304277	-3.951320143	7.50E-04	1.49E-03	-1.76240352
Myc	-0.903436767	7.67033182	-2.918406673	8.32E-03	1.35E-02	-4.113334033
Hbb-bt	-0.903647593	2.603590351	-1.146865187	2.65E-01	3.15E-01	-7.177005626
Prr5	-0.904964275	6.04749199	-4.505783759	2.02E-04	4.50E-04	-0.450197927
Gm4070	-0.90854902	7.477327306	-2.962427878	7.53E-03	1.24E-02	-4.017902833
Ctse	-0.911010454	6.029768282	-4.296318933	3.31E-04	7.09E-04	-0.947220839
Dtl	-0.912422546	6.323683569	-5.323088021	2.97E-05	7.82E-05	1.481559389
Bin3	-0.912895917	9.202127566	-8.612589297	2.85E-08	1.32E-07	8.591642697
Chd7	-0.914361495	7.977361565	-8.921482991	1.60E-08	7.93E-08	9.188266385
Rcn2	-0.914569823	6.831274168	-8.836831978	1.87E-08	9.12E-08	9.025992046
2610034B18Rik	-0.918086724	8.096127708	-5.199863008	3.95E-05	1.01E-04	1.192307522
Vdr	-0.918417213	5.634524887	-3.326557756	3.26E-03	5.78E-03	-3.208653012
Lrrk2	-0.919596709	8.97033322	-10.37682752	1.22E-09	7.72E-09	11.83815983
Kif15	-0.922028368	6.397428172	-4.777838304	1.06E-04	2.52E-04	0.195378897
Coro2a	-0.923912035	9.248135825	-7.118096797	5.63E-07	2.04E-06	5.527875533
Ptpn7	-0.924918592	6.299322603	-4.662917348	1.39E-04	3.20E-04	-0.077204448
Rraga	-0.925012472	8.50758475	-10.22101697	1.59E-09	9.74E-09	11.56670257
Plk1	-0.925246632	6.298864528	-4.668371848	1.37E-04	3.17E-04	-0.064261091
Camk2d	-0.928208367	7.126427314	-8.015976694	9.05E-08	3.78E-07	7.403962332
Wdr91	-0.928877628	8.174263296	-12.09718205	7.90E-11	6.83E-10	14.65663008
Casc5	-0.933916701	7.128899501	-6.493168806	2.14E-06	7.02E-06	4.160530745
Cdkn3	-0.93488561	6.479366691	-3.772118836	1.15E-03	2.20E-03	-2.182421614
Lig1	-0.938064246	8.180028593	-10.77624359	6.28E-10	4.33E-09	12.52126844
Fasn	-0.938963179	7.242576655	-8.306881787	5.12E-08	2.26E-07	7.988926008
Zbp1	-0.940500289	8.393964007	-6.060437859	5.56E-06	1.68E-05	3.18613928
Slc35c2	-0.940551465	7.588240706	-6.127898044	4.78E-06	1.47E-05	3.339428995
Samd9l	-0.940641009	9.021267477	-11.08549186	3.80E-10	2.75E-09	13.03786237
Entpd1	-0.942091976	7.963696257	-6.246356624	3.68E-06	1.15E-05	3.607388696
Galc	-0.944013593	6.986739478	-7.733815691	1.59E-07	6.34E-07	6.825873102
Ccna2	-0.945177625	7.486485905	-3.75868825	1.18E-03	2.26E-03	-2.213768091
Dhx58	-0.945947264	7.111695806	-9.023538788	1.32E-08	6.64E-08	9.382681285
Aff1	-0.948422547	8.930080265	-11.93638925	1.01E-10	8.38E-10	14.40638624
Gm20559	-0.948629574	8.945558375	-4.942155392	7.21E-05	1.75E-04	0.584478434
Ccnb2	-0.95222807	7.145030868	-3.544721837	1.96E-03	3.62E-03	-2.710053486
Epb4.1l2	-0.957842626	9.68981099	-10.24253015	1.53E-09	9.43E-09	11.6043522
Snord69	-0.960373032	5.527187802	-3.598956304	1.72E-03	3.22E-03	-2.584860293
Ube2c	-0.963092783	8.272575505	-4.576351667	1.71E-04	3.87E-04	-0.282670002
Fkbp1a	-0.966026038	8.348446256	-8.710425452	2.37E-08	1.12E-07	8.781950156
Smyd5	-0.968003502	6.031133565	-5.27288348	3.34E-05	8.67E-05	1.363835011
Rasa3	-0.968263879	7.106868883	-7.161402212	5.14E-07	1.88E-06	5.620790757
Pmepa1	-0.970261821	6.190990757	-7.39342837	3.18E-07	1.20E-06	6.114460492
Cdca7	-0.972710628	5.397573053	-3.201834116	4.35E-03	7.48E-03	-3.4894768
Got2	-0.973966734	10.25026882	-11.70519538	1.43E-10	1.15E-09	14.04200762
Nuf2	-0.979911124	6.831419082	-4.531550821	1.90E-04	4.26E-04	-0.389026413
Kif18b	-0.98124183	6.065445181	-4.32676154	3.08E-04	6.62E-04	-0.875036988
Sh3tc1	-0.981553253	6.872395662	-4.98375584	6.54E-05	1.61E-04	0.68282345
Ppp1r15b	-0.982268907	8.447077901	-12.80446796	2.79E-11	2.68E-10	15.72750043

Slc4a7	-0.982469487	6.924655699	-5.172907503	4.21E-05	1.08E-04	1.128902388
Zfp318	-0.98278246	8.914553586	-11.44562126	2.15E-10	1.65E-09	13.62634361
Cdc45	-0.983252422	6.657804356	-10.49077698	1.01E-09	6.53E-09	12.03490197
Erc1	-0.98356733	6.552512306	-6.616871269	1.64E-06	5.48E-06	4.435046629
Fgd2	-0.983747641	9.020210552	-7.228621631	4.47E-07	1.65E-06	5.764533094
Tmem131	-0.983993154	9.148026874	-12.12168742	7.61E-11	6.61E-10	14.69454218
Gpr65	-0.984508761	9.306427264	-8.673163529	2.54E-08	1.19E-07	8.709616418
Cks1b	-0.986248868	8.850983575	-5.793414513	1.01E-05	2.88E-05	2.574697498
Hps3	-0.988180734	8.345855002	-7.063415659	6.31E-07	2.26E-06	5.410206308
Rnf145	-0.991566152	8.277729139	-10.08948391	1.99E-09	1.19E-08	11.33532469
Txn1	-0.992021285	9.004862461	-10.40878462	1.16E-09	7.41E-09	11.89348736
Gng2	-0.994855836	7.25551001	-8.430959567	4.03E-08	1.80E-07	8.235029563
Naa25	-0.997222815	7.720262714	-8.451329137	3.88E-08	1.74E-07	8.275238457
Gm9257	-0.997816042	4.315911572	-1.911752663	6.99E-02	9.49E-02	-6.08331349
Kif20b	-0.998303463	6.961430944	-7.798711095	1.39E-07	5.63E-07	6.959765071
E2f8	-1.000204408	6.283492015	-5.907579279	7.83E-06	2.28E-05	2.837008008
Pglyrp1	-1.001500769	8.99847216	-11.57921403	1.74E-10	1.36E-09	13.84114571
Taldo1	-1.002569433	8.994982747	-10.00536767	2.29E-09	1.35E-08	11.18628202
Cdc25a	-1.003270803	6.159737809	-5.936867217	7.33E-06	2.14E-05	2.904090313
Ccdc107	-1.005503309	7.889656615	-8.76346133	2.15E-08	1.03E-07	8.884593794
LOC102638398	-1.008537765	5.873146794	-2.59154366	1.72E-02	2.61E-02	-4.801721501
Gm15527	-1.008574814	4.442148937	-1.86944779	7.58E-02	1.02E-01	-6.154661536
Dgkh	-1.009888933	6.178628513	-6.700914423	1.37E-06	4.60E-06	4.620487187
Cd3eap	-1.011962433	6.299352577	-8.530527923	3.33E-08	1.51E-07	8.43105741
Map4k5	-1.015092328	7.759060342	-14.4264562	3.02E-12	3.99E-11	18.01344537
Cyp20a1	-1.017677974	6.227364857	-6.067057524	5.48E-06	1.65E-05	3.201202936
Ndrg1	-1.018563632	7.647247118	-8.843530275	1.85E-08	9.03E-08	9.038866198
Pes1	-1.020520736	8.308229408	-9.492047587	5.65E-09	3.08E-08	10.25822533
Lrba	-1.021759679	8.897247366	-13.65355418	8.48E-12	9.46E-11	16.95218908
Ifi44	-1.025431152	6.777760474	-5.720481993	1.20E-05	3.36E-05	2.406459728
Rpl34	-1.028565014	7.148249015	-2.936452082	7.98E-03	1.31E-02	-4.074284966
Eif2ak4	-1.032010241	7.262947738	-9.143482563	1.06E-08	5.43E-08	9.609471527
Ncoa7	-1.035092455	8.252287709	-10.38241336	1.21E-09	7.67E-09	11.84783916
Phka2	-1.036563638	8.569577961	-14.31057136	3.51E-12	4.54E-11	17.85742219
Mospd1	-1.03677664	8.45151865	-7.337107744	3.57E-07	1.34E-06	5.99527731
Klf5	-1.037117191	6.150367579	-5.092426704	5.08E-05	1.27E-04	0.93933814
Ahr	-1.038730893	7.982569263	-8.153084288	6.91E-08	2.96E-07	7.681057907
Kcnn4	-1.041015406	6.426787987	-4.19904076	4.17E-04	8.74E-04	-1.177681155
Nampt	-1.042217335	8.165847889	-12.0952634	7.92E-11	6.83E-10	14.65365924
Naga	-1.045764835	10.05448765	-10.76963554	6.35E-10	4.36E-09	12.51011397
Isoc1	-1.045853322	8.709494497	-12.2265079	6.51E-11	5.73E-10	14.85604116
Ppp1r11	-1.045897624	9.441709589	-9.295144516	8.05E-09	4.24E-08	9.893622261
Zfp398	-1.047642424	7.123694168	-10.13397769	1.84E-09	1.12E-08	11.41382177
Fbl	-1.049362191	5.574635288	-2.652335136	1.50E-02	2.32E-02	-4.676625966
Cpne3	-1.050559246	8.938890067	-12.69206089	3.28E-11	3.09E-10	15.5604865
Unc93b1	-1.051668783	10.15589925	-10.81443605	5.90E-10	4.09E-09	12.58564201
Cxx1b	-1.05666839	7.029144456	-5.692779024	1.27E-05	3.57E-05	2.342424397
Evi2b	-1.058401386	8.022141535	-5.218451046	3.79E-05	9.75E-05	1.236004186
Mlec	-1.061208573	8.06404517	-7.753031416	1.53E-07	6.14E-07	6.865577099
Chek1	-1.061556571	5.901866106	-8.545391746	3.24E-08	1.48E-07	8.460209573
Itgav	-1.061713094	8.156324863	-11.99148062	9.26E-11	7.76E-10	14.49241654
Egr2	-1.063199385	6.81097633	-4.943216109	7.19E-05	1.75E-04	0.58698694
Gpr157	-1.064236033	8.377502606	-11.51090342	1.94E-10	1.49E-09	13.73154346
Cln3	-1.064675122	8.03540386	-10.64936492	7.74E-10	5.23E-09	12.30623803
Gsap	-1.065139231	9.214472884	-10.58943309	8.54E-10	5.71E-09	12.20403455
Stx17	-1.067404663	8.534968436	-9.9734255	2.43E-09	1.42E-08	11.12946363
Batf3	-1.07069188	10.31572826	-10.09964095	1.95E-09	1.17E-08	11.35326468
Rnf157	-1.072161953	6.973249331	-6.610353032	1.66E-06	5.54E-06	4.420627962
Plekho2	-1.073110856	9.555258859	-10.24779947	1.52E-09	9.37E-09	11.61356563
Sass6	-1.074486236	7.387408582	-8.220025894	6.07E-08	2.62E-07	7.815443873
Ppan	-1.078066652	7.028128217	-5.207436949	3.88E-05	9.98E-05	1.21011494
Il15	-1.078955831	8.38113635	-5.844583519	9.02E-06	2.59E-05	2.692425772
Twsg1	-1.082369703	6.831693132	-7.035068545	6.70E-07	2.39E-06	5.349053991
Pqlc2	-1.083298996	7.040839707	-6.466972462	2.27E-06	7.34E-06	4.102160445
Arhgap15os	-1.086265196	5.954566012	-5.676731815	1.32E-05	3.69E-05	2.305298919
Sfi1	-1.088044792	7.4496034	-10.37872793	1.21E-09	7.71E-09	11.84145333
Gm12185	-1.091716914	5.637369261	-2.951325987	7.72E-03	1.27E-02	-4.042024828
Cenpe	-1.092092764	6.877350117	-9.410282593	6.54E-09	3.51E-08	10.10740992
Tubb2a	-1.093620652	8.342029579	-9.472327464	5.85E-09	3.19E-08	10.22192769
Gm11545	-1.094579972	8.392483298	-5.481931037	2.06E-05	5.59E-05	1.85281145
Dctpp1	-1.096456179	8.693015794	-5.896796917	8.02E-06	2.32E-05	2.812289627
Atp8b4	-1.100158145	7.052260649	-5.104992901	4.93E-05	1.24E-04	0.968960786
Hdac9	-1.107851002	5.968537578	-8.736928035	2.26E-08	1.08E-07	8.83328786
Stard3nl	-1.111330034	8.183465518	-14.34763974	3.35E-12	4.35E-11	17.90744587
Rala	-1.112157104	9.548555386	-14.22006954	3.96E-12	5.01E-11	17.73482816
Lipa	-1.116657997	8.20974465	-10.44862597	1.08E-09	6.97E-09	11.96229964

Trit1	-1.121965533	7.309790003	-5.957496161	7.00E-06	2.06E-05	2.951286822
Cdc20	-1.123669552	6.669799448	-7.865875838	1.22E-07	4.99E-07	7.09775111
Atp8a1	-1.124701399	8.247189182	-12.84712702	2.62E-11	2.54E-10	15.79057639
Zfp367	-1.124980163	7.734106861	-8.801832311	2.00E-08	9.69E-08	8.958628311
Slc25a38	-1.125277983	7.544225489	-14.76717646	1.94E-12	2.68E-11	18.46607662
Nup93	-1.129405418	8.17604901	-15.94916908	4.48E-13	7.44E-12	19.96927537
Bid	-1.132932479	7.344732721	-13.3732391	1.25E-11	1.34E-10	16.5549179
Nccrp1	-1.133664865	6.655291153	-7.265417231	4.14E-07	1.53E-06	5.842967748
Btla	-1.140124651	8.4863276	-7.25448849	4.24E-07	1.57E-06	5.819690145
Acox3	-1.140603613	7.906191119	-10.87416467	5.35E-10	3.78E-09	12.68598872
Bcl9	-1.141027358	8.279668981	-13.55602366	9.69E-12	1.07E-10	16.81473437
Snord92	-1.141927662	6.271224249	-3.352839551	3.07E-03	5.46E-03	-3.149057037
Olfm1	-1.143640115	7.388906806	-12.95501838	2.25E-11	2.21E-10	15.94935856
Klf2	-1.144265658	7.193229671	-8.739659395	2.24E-08	1.07E-07	8.838573551
Cldn1	-1.150973217	8.730410583	-6.935750585	8.27E-07	2.89E-06	5.13398548
Kif4	-1.160173456	6.112230354	-6.160702281	4.45E-06	1.37E-05	3.413790293
F630111L10Rik	-1.161249725	7.034251229	-5.031936105	5.84E-05	1.45E-04	0.796624439
Mgat4b	-1.162349168	7.18216124	-10.65328401	7.69E-10	5.21E-09	12.31290716
Slc9a9	-1.162680334	7.888041247	-10.25652984	1.49E-09	9.27E-09	11.62882354
Ccnyl1	-1.163212889	6.8094208	-4.915297569	7.68E-05	1.86E-04	0.520946544
Pigf	-1.164585382	6.816505188	-7.620828835	2.00E-07	7.81E-07	6.591427514
Praf2	-1.16582158	8.457226877	-10.49499743	1.00E-09	6.51E-09	12.04216019
Mir23a	-1.166604568	6.755818063	-3.893007543	8.62E-04	1.69E-03	-1.899411965
Arhgap15	-1.168496065	8.407067823	-5.311465482	3.05E-05	8.00E-05	1.454321384
Trim30b	-1.172586973	5.477787672	-3.471892238	2.32E-03	4.25E-03	-2.877439667
4632428N05Rik	-1.175353098	8.291762172	-14.80025422	1.86E-12	2.61E-11	18.50954151
Sipa1l1	-1.175371615	7.79716476	-13.14695461	1.71E-11	1.76E-10	16.22921216
Rrm2	-1.175400074	6.449233412	-5.662559121	1.36E-05	3.80E-05	2.272490597
Hes1	-1.177501557	5.937299761	-6.482439478	2.19E-06	7.16E-06	4.136633726
Sdc3	-1.179262488	9.558665753	-12.54385316	4.07E-11	3.74E-10	15.33847163
Itpripl1	-1.179697571	7.287809668	-7.157437022	5.18E-07	1.89E-06	5.612293236
Hmmr	-1.180853173	5.981850155	-9.34985162	7.29E-09	3.88E-08	9.99540915
Pik3cb	-1.184155205	9.726493434	-16.94888077	1.39E-13	2.89E-12	21.16561243
Mir6516	-1.185459565	6.698449891	-3.550328687	1.93E-03	3.59E-03	-2.697131618
Psap	-1.187288153	11.45194617	-17.99790204	4.35E-14	1.11E-12	22.35402193
Nek6	-1.190557397	8.192435407	-13.78345295	7.10E-12	8.14E-11	17.13400525
Trafd1	-1.197793651	9.98069212	-14.15280051	4.33E-12	5.38E-11	17.64327791
Rab43	-1.198128077	9.169934332	-19.11033244	1.35E-14	4.34E-13	23.54611615
Snord14e	-1.20034506	10.5651819	-6.070676011	5.43E-06	1.64E-05	3.209435143
Ppfibp1	-1.202401013	8.098501127	-13.53238597	1.00E-11	1.10E-10	16.78129774
Mrpl42	-1.216519484	9.221308097	-9.860470483	2.95E-09	1.69E-08	10.92755822
P2ry14	-1.219377884	7.11231439	-5.87416684	8.44E-06	2.44E-05	2.760372355
Rinl	-1.222591266	6.922320346	-9.764079856	3.49E-09	1.96E-08	10.75404289
LOC102636093	-1.222954194	6.91202084	-5.320327221	2.99E-05	7.86E-05	1.475090181
Arsb	-1.223002087	9.146303398	-13.89284478	6.13E-12	7.20E-11	17.2860169
Chn2	-1.22921303	7.526115589	-6.122794882	4.84E-06	1.48E-05	3.32785041
Gpd2	-1.234967191	7.988873489	-15.07823631	1.31E-12	1.91E-11	18.87154624
Arhgap5	-1.23683876	7.796780178	-8.597872911	2.93E-08	1.35E-07	8.56290902
Tap2	-1.238006587	9.052288971	-16.69821954	1.86E-13	3.63E-12	20.87173061
Hps4	-1.238824832	7.611785493	-14.6167369	2.36E-12	3.21E-11	18.26733635
Cln8	-1.244202142	5.525438872	-4.642164003	1.46E-04	3.35E-04	-0.126455397
Ebi3	-1.244840162	9.209523945	-13.72894777	7.65E-12	8.63E-11	17.0578895
Stx3	-1.247328599	7.350100803	-9.54294599	5.16E-09	2.83E-08	10.35168808
3110043O21Rik	-1.254889393	8.813981224	-13.73392224	7.60E-12	8.60E-11	17.06484667
Gas2l3	-1.256651405	5.918542315	-9.436180575	6.24E-09	3.38E-08	10.15526877
Impa2	-1.257572175	6.664882595	-7.858136337	1.24E-07	5.05E-07	7.081881251
Rbm47	-1.258034491	7.592938741	-11.35061823	2.49E-10	1.87E-09	13.47244664
Dpep2	-1.262535785	5.969363232	-6.376432325	2.76E-06	8.81E-06	3.899793627
Slfn5	-1.26460591	8.022199732	-12.82979491	2.69E-11	2.59E-10	15.76496929
Snora41	-1.270415876	7.137803672	-6.272339083	3.47E-06	1.09E-05	3.665951473
AW046200	-1.271047311	6.408441906	-7.042117253	6.60E-07	2.36E-06	5.3642696
Sgk1	-1.271978606	7.049701651	-6.946762087	8.08E-07	2.83E-06	5.157892521
Umps	-1.272590059	7.453837299	-8.174219662	6.63E-08	2.85E-07	7.723551372
Trim35	-1.282785366	8.131212981	-18.5701284	2.36E-14	7.07E-13	22.97557808
Abcg3	-1.284148216	7.276252655	-7.435277044	2.92E-07	1.10E-06	6.202748905
Ugt1a6b	-1.284383653	4.519828083	-2.176575762	4.13E-02	5.88E-02	-5.61243959
Lpcat4	-1.284941114	8.087878002	-13.21821864	1.55E-11	1.61E-10	16.33227655
Cyb5a	-1.287575395	9.286090921	-18.20377828	3.48E-14	9.12E-13	22.57973759
Elac2	-1.290069075	6.807338894	-9.871150672	2.90E-09	1.67E-08	10.94671468
Rab11fip1	-1.299521975	8.235173463	-13.18586612	1.62E-11	1.68E-10	16.28554339
S100a1	-1.301598493	7.38739922	-6.453695839	2.33E-06	7.52E-06	4.072546441
Ipcef1	-1.30307536	6.323205744	-4.815219817	9.71E-05	2.32E-04	0.283976198
Sh3bp1	-1.306038064	9.798665569	-12.06913782	8.24E-11	7.06E-10	14.61317
Extl3	-1.310996782	6.628376183	-9.115794692	1.12E-08	5.70E-08	9.557281748
Met	-1.312991467	7.356055875	-7.828398681	1.31E-07	5.34E-07	7.020830253
Ffar4	-1.328004891	8.095909744	-11.75293721	1.33E-10	1.08E-09	14.11769866

Ly75	-1.33095154	8.662952836	-8.431862258	4.03E-08	1.80E-07	8.236812599
Arhgap18	-1.335641055	7.794720247	-12.01706837	8.91E-11	7.50E-10	14.53227079
Fuca1	-1.335916605	10.70535348	-17.60835649	6.65E-14	1.58E-12	21.92032187
P2ry6	-1.348423353	8.522201377	-3.849998246	9.54E-04	1.85E-03	-2.000268272
Hist2h2ab	-1.349433447	7.317596392	-4.950304881	7.07E-05	1.72E-04	0.603750051
Zfp366	-1.354021937	9.313859423	-13.91215713	5.97E-12	7.03E-11	17.31274977
Myo9a	-1.357029285	8.440630997	-14.67883937	2.18E-12	2.99E-11	18.34958903
Ttll4	-1.372960861	7.272958801	-8.789738861	2.04E-08	9.88E-08	8.935315283
Hells	-1.376667261	7.467238717	-11.91767591	1.04E-10	8.60E-10	14.37709425
Mical2	-1.377151005	5.712521307	-7.640250432	1.92E-07	7.54E-07	6.631847265
Rab30	-1.379894646	9.814604645	-11.44899053	2.14E-10	1.64E-09	13.63178404
LOC102636125	-1.379926547	8.998354132	-9.078174519	1.20E-08	6.04E-08	9.486214041
Ndufaf6	-1.388159325	6.619689432	-10.3863894	1.20E-09	7.64E-09	11.85472677
Perm1	-1.390387291	6.543290689	-15.72607478	5.87E-13	9.41E-12	19.69319838
Ptpn22	-1.3907265	8.721069116	-11.17217092	3.31E-10	2.43E-09	13.18077656
Cep70	-1.392861436	7.049002245	-9.627656072	4.44E-09	2.45E-08	10.50652814
Rab19	-1.394760223	6.617738711	-9.750222016	3.58E-09	2.00E-08	10.72900424
4930506M07Rik	-1.398945401	9.555455155	-15.48190974	7.91E-13	1.22E-11	19.3870623
B3gnt5	-1.40671968	5.861956962	-8.586431487	3.00E-08	1.38E-07	8.540550166
Pkib	-1.40727317	6.742597448	-8.41028889	4.20E-08	1.87E-07	8.1941706
Fnbp1	-1.411133916	10.23608777	-15.90309013	4.74E-13	7.80E-12	19.91253423
Mir3074-2	-1.412582003	5.278872336	-2.768941605	1.16E-02	1.84E-02	-4.432773882
Glce	-1.412828021	8.171378452	-13.12490096	1.77E-11	1.80E-10	16.19722551
Gpr124	-1.424615561	7.241624119	-6.492697475	2.14E-06	7.02E-06	4.159481259
LOC102638890	-1.429689611	5.09542707	-4.106639066	5.19E-04	1.07E-03	-1.396215549
Fbxw17	-1.430452414	7.49126675	-13.63457152	8.70E-12	9.68E-11	16.92549959
Pdlim2	-1.435026493	7.51362227	-13.74334403	7.50E-12	8.52E-11	17.07801804
Gm15698	-1.438211705	5.716071614	-5.831432706	9.30E-06	2.66E-05	2.662193263
4933416M07Rik	-1.438315499	7.739805575	-10.2365952	1.54E-09	9.51E-09	11.59397103
Dennd1b	-1.443541033	7.861434765	-13.03516841	2.01E-11	2.00E-10	16.06662655
Mboat1	-1.447589762	5.965190479	-6.813883857	1.07E-06	3.68E-06	4.868373894
Ifi204	-1.448495305	6.472628719	-5.087044028	5.14E-05	1.29E-04	0.926646769
Arhgap21	-1.44856164	7.339976254	-12.88174973	2.50E-11	2.44E-10	15.84164646
Mospd2	-1.450975333	7.597472291	-18.33617846	3.03E-14	8.27E-13	22.7236462
Asap2	-1.453407867	7.998992225	-13.93408873	5.80E-12	6.85E-11	17.34307074
Lpar6	-1.454790667	7.998558088	-13.59610852	9.17E-12	1.01E-10	16.87132655
Havcr2	-1.458250046	7.995881211	-10.74431119	6.62E-10	4.52E-09	12.46732086
Cdk1	-1.458958851	7.777301403	-6.10561005	5.03E-06	1.53E-05	3.288838719
Cdk14	-1.460507786	8.095736195	-10.2643748	1.47E-09	9.17E-09	11.64252643
Ifngr1	-1.462222449	9.772967925	-19.89049342	6.15E-15	2.52E-13	24.34371683
Nr4a2	-1.468220305	7.629327767	-6.379168956	2.75E-06	8.77E-06	3.905924426
Inpp1	-1.469615019	6.784018527	-7.025878641	6.83E-07	2.43E-06	5.329206728
Pi4k2a	-1.470148483	8.386356246	-16.95591656	1.38E-13	2.89E-12	21.17380445
Grk5	-1.472552549	8.194875821	-11.72269585	1.40E-10	1.12E-09	14.06978044
Endod1	-1.476393243	6.955896099	-10.445919	1.09E-09	6.99E-09	11.95763007
Tlr12	-1.484551516	6.995768112	-5.987171856	6.55E-06	1.94E-05	3.019103768
Hspa1a	-1.490032622	9.036124161	-2.374064005	2.74E-02	4.02E-02	-5.236630233
Cd97	-1.500961291	10.31095014	-13.06161015	1.93E-11	1.95E-10	16.10518593
Vrk2	-1.501543541	8.359128097	-12.67076597	3.38E-11	3.17E-10	15.52871384
Irf8	-1.504470799	10.05794574	-11.34523217	2.52E-10	1.88E-09	13.46369302
Nlrp1a	-1.508492763	5.660417997	-8.648998417	2.66E-08	1.24E-07	8.662609998
Gm14005	-1.51884555	5.413820671	-9.351111694	7.28E-09	3.88E-08	9.997749195
Pfkp	-1.519493851	8.905108896	-17.10339637	1.17E-13	2.58E-12	21.3448139
Plk3	-1.526093103	6.219663783	-8.198094387	6.33E-08	2.72E-07	7.771481374
Txndc15	-1.527275699	8.104183704	-13.34428066	1.30E-11	1.38E-10	16.51348833
Gm1965	-1.528004413	7.120679071	-5.992046679	6.48E-06	1.92E-05	3.030235254
2210409E12Rik	-1.533328047	5.310097792	-5.062796662	5.44E-05	1.36E-04	0.869456654
Evl	-1.53498081	8.696234512	-16.28666813	3.00E-13	5.49E-12	20.38048382
Sdad1	-1.535156745	7.884102251	-9.195501339	9.65E-09	4.99E-08	9.707260428
Prelid2	-1.537252401	6.638036666	-9.465262384	5.93E-09	3.22E-08	10.20891167
Ppa1	-1.539888652	7.430259083	-15.24191279	1.07E-12	1.58E-11	19.08200299
Wdfy4	-1.540171563	9.993829889	-11.63119748	1.61E-10	1.27E-09	13.92422602
Mllt3	-1.541175733	6.505850756	-10.52724668	9.48E-10	6.23E-09	12.09755403
LOC102638989	-1.542384046	6.966836302	-4.745522695	1.14E-04	2.69E-04	0.118757935
Sash1	-1.550745521	6.453687552	-16.57295536	2.15E-13	4.06E-12	20.72337189
4930412F12Rik	-1.55207214	6.185930048	-11.74524673	1.35E-10	1.09E-09	14.10552179
Txndc16	-1.552378809	6.876667832	-10.59256437	8.50E-10	5.69E-09	12.20938452
Prkar2a	-1.560923977	7.217814115	-10.32918883	1.32E-09	8.30E-09	11.7554628
Kit	-1.562891613	9.114664469	-9.808842347	3.23E-09	1.83E-08	10.83476118
Irf5	-1.566458124	9.022943291	-18.33285592	3.04E-14	8.27E-13	22.72004672
Tm6sf1	-1.573613426	8.305949424	-10.52905233	9.45E-10	6.22E-09	12.10065203
Ece1	-1.576201972	10.0643056	-18.37893626	2.89E-14	8.19E-13	22.76991378
Tns3	-1.576833849	8.618924147	-14.11897071	4.53E-12	5.56E-11	17.59709842
Cnr2	-1.578678678	7.513759673	-6.847024256	9.99E-07	3.44E-06	4.940790058
Tspan33	-1.58676315	8.268463452	-15.70815877	6.00E-13	9.54E-12	19.67087777
Arhgap8	-1.599224192	5.097424559	-6.039323642	5.83E-06	1.75E-05	3.13806054

Polr1a	-1.601895361	7.155112651	-13.62627919	8.80E-12	9.76E-11	16.91383098
Unc119	-1.607617536	6.520833578	-6.539619185	1.93E-06	6.39E-06	4.263828611
Unc119b	-1.622346285	7.188780671	-7.942540446	1.05E-07	4.33E-07	7.254522934
Adam8	-1.625354772	9.635298195	-14.27512325	3.68E-12	4.74E-11	17.80948236
Ttc39b	-1.629901155	7.850540114	-13.09086898	1.85E-11	1.88E-10	16.14777986
Aif1	-1.630138415	10.93176755	-17.92024854	4.73E-14	1.19E-12	22.26826307
Camk1d	-1.636687698	8.609589749	-16.60924607	2.06E-13	3.92E-12	20.76645703
Dnase1l3	-1.636893151	10.43426646	-14.13966597	4.40E-12	5.44E-11	17.6253596
Snord13	-1.649456564	7.926611735	-5.526767942	1.86E-05	5.09E-05	1.957246494
Pla2g16	-1.654259025	8.650881302	-13.41057528	1.18E-11	1.28E-10	16.60822484
Cxcr3	-1.659140164	7.509113504	-13.0387996	2.00E-11	2.00E-10	16.07192557
Slco3a1	-1.667244994	6.528850375	-9.839730682	3.06E-09	1.74E-08	10.89031902
Fanca	-1.672159648	5.926878605	-12.94782952	2.27E-11	2.23E-10	15.93881194
Fam71a	-1.67234909	5.759725648	-5.152928324	4.41E-05	1.12E-04	1.081878586
Klrk1	-1.675217682	7.905746721	-10.8632354	5.45E-10	3.82E-09	12.66765672
Cd24a	-1.687949395	9.521115482	-9.879561352	2.85E-09	1.64E-08	10.96179076
Kif23	-1.689544266	7.012581323	-11.31069427	2.66E-10	1.97E-09	13.40748733
Atp6v0a2	-1.690065899	8.727068389	-23.067468	3.28E-16	3.02E-14	27.30654539
Parvg	-1.690351094	8.490631171	-10.04373992	2.15E-09	1.27E-08	11.25437701
Glrx	-1.703501022	8.436567128	-14.82770285	1.80E-12	2.54E-11	18.54554627
Slc22a21	-1.718498923	6.600686177	-10.56395186	8.91E-10	5.93E-09	12.16045693
Clec12a	-1.723479003	9.380610779	-14.31465824	3.49E-12	4.53E-11	17.8629428
Myof	-1.726498758	7.327679361	-12.6073031	3.71E-11	3.45E-10	15.43377291
Eefsec	-1.734408689	7.558352341	-12.11727597	7.66E-11	6.64E-10	14.68772164
Ighv10-3	-1.735896268	2.622609056	-1.777428346	9.02E-02	1.19E-01	-6.305893741
Kctd14	-1.738013872	6.241178617	-10.69368814	7.19E-10	4.89E-09	12.38156193
Gna15	-1.741010631	6.265033442	-7.857996262	1.24E-07	5.05E-07	7.081593955
Lrrc1	-1.741578408	6.532885934	-12.3584139	5.35E-11	4.79E-10	15.05774507
2310040G24Rik	-1.741706294	6.392084965	-12.78722434	2.86E-11	2.74E-10	15.70195616
Rab39	-1.744929845	6.064952128	-7.658708202	1.85E-07	7.29E-07	6.670214855
Osbpl3	-1.7680797	8.047636373	-14.78968472	1.89E-12	2.63E-11	18.49566204
BC028528	-1.773824712	8.793804956	-16.93492603	1.41E-13	2.91E-12	21.14935529
Arrb1	-1.791963052	7.778697735	-17.26560331	9.73E-14	2.20E-12	21.53135722
Sult1a1	-1.794940123	6.841788588	-12.6016187	3.74E-11	3.47E-10	15.42525051
P2rx7	-1.799290995	6.555262737	-9.345812876	7.35E-09	3.91E-08	9.987907583
Lap3	-1.802075232	6.909937327	-9.28321545	8.23E-09	4.32E-08	9.871377363
Rab32	-1.807115743	9.838851031	-17.89288908	4.87E-14	1.21E-12	22.23796594
Dnmbp	-1.811746762	6.875853083	-11.61590498	1.65E-10	1.30E-09	13.89981457
1700084J12Rik	-1.821904448	5.211180195	-2.462516242	2.27E-02	3.38E-02	-5.062236906
Cpd	-1.82274241	7.138861168	-21.90737764	9.14E-16	6.05E-14	26.27387402
Heg1	-1.824329177	6.891176371	-14.44726077	2.94E-12	3.91E-11	18.0413432
Ppfibp2	-1.825705031	7.483509246	-14.0596235	4.90E-12	5.92E-11	17.51586103
AI607873	-1.833616327	8.012574171	-17.0549674	1.23E-13	2.67E-12	21.28880661
Egr1	-1.837007757	9.487764641	-10.86792728	5.41E-10	3.81E-09	12.67552819
Ckb	-1.839844189	8.828187285	-8.237892138	5.86E-08	2.54E-07	7.851210449
Arap1	-1.840997883	7.212967377	-18.39296295	2.85E-14	8.13E-13	22.78506999
Itga1	-1.841415387	6.422657691	-12.04061029	8.60E-11	7.27E-10	14.56888082
Cdc14a	-1.848626947	8.86376701	-24.64310571	8.79E-17	1.42E-14	28.62876967
Cxcl10	-1.848979969	6.84682039	-8.905339174	1.65E-08	8.16E-08	9.157390199
Mpeg1	-1.855333804	9.402039646	-14.1475151	4.36E-12	5.40E-11	17.63606917
Ubash3b	-1.860571394	7.660277122	-15.99828679	4.23E-13	7.14E-12	20.02959863
Smagp	-1.870080786	6.77142385	-16.72647582	1.80E-13	3.56E-12	20.90505769
Apol10b	-1.870122753	6.892952355	-7.200458495	4.74E-07	1.74E-06	5.704380442
Sgms1	-1.877476978	7.67971379	-15.69374989	6.10E-13	9.67E-12	19.65291023
Parp12	-1.890454852	8.318187572	-16.68401716	1.89E-13	3.67E-12	20.85496029
Exoc3l4	-1.891930472	6.417277582	-11.90576125	1.06E-10	8.74E-10	14.35842583
Laptm4b	-1.903423492	7.818255141	-13.70585555	7.90E-12	8.86E-11	17.02556586
Plekhn1	-1.905416552	8.385330343	-12.45928654	4.61E-11	4.21E-10	15.21085901
Pdlim7	-1.909166154	6.501607425	-10.86370421	5.44E-10	3.82E-09	12.66844334
Ptplb	-1.912380942	8.2115472	-14.9346993	1.57E-12	2.26E-11	18.68534967
Arid3b	-1.919811046	7.676500925	-14.76796289	1.94E-12	2.68E-11	18.46711099
Pak1	-1.921562241	9.795852347	-27.57359333	9.24E-18	2.49E-15	30.87252259
Ccdc86	-1.924129751	7.024811399	-8.563579278	3.13E-08	1.43E-07	8.495841168
Fnip2	-1.925997963	7.759473744	-16.09593799	3.76E-13	6.47E-12	20.14904082
Nr4a1	-1.934765079	9.232516331	-12.43302946	4.79E-11	4.36E-10	15.17109766
C920021L13Rik	-1.936342123	6.005241161	-16.71896366	1.81E-13	3.58E-12	20.89620238
Cd86	-1.939028142	10.07374991	-21.63739831	1.17E-15	7.34E-14	26.02579449
Tctex1d4	-1.986152457	5.892603968	-9.196065616	9.64E-09	4.99E-08	9.70831932
Gclc	-2.004405353	8.926358168	-20.96575717	2.18E-15	1.14E-13	25.39522958
LOC102640171	-2.006717318	6.118947326	-4.517420035	1.96E-04	4.38E-04	-0.422573278
Gdpd5	-2.00730794	6.811416828	-16.84455047	1.57E-13	3.21E-12	21.04377375
Gpr34	-2.021526827	6.783433189	-13.26281073	1.46E-11	1.53E-10	16.39653736
Lrrc18	-2.031620551	7.888104188	-11.00079792	4.36E-10	3.14E-09	12.89743075
Ccl2	-2.046310077	6.444811623	-6.330489798	3.06E-06	9.71E-06	3.796739591
Plin2	-2.049434121	8.495350645	-14.25185153	3.80E-12	4.86E-11	17.77795506
Hck	-2.064184729	8.988984994	-19.55908579	8.56E-15	3.13E-13	24.00860269

Ptpn18	-2.068800293	7.329852706	-13.30451714	1.37E-11	1.45E-10	16.45648044
Tnf	-2.081775316	8.951195836	-15.96009425	4.42E-13	7.41E-12	19.9827072
Gpr4	-2.087855016	7.131522401	-20.00817458	5.48E-15	2.29E-13	24.46143122
Cxx1c	-2.098594589	8.936893552	-19.73690613	7.16E-15	2.76E-13	24.18907977
Cyb5r1	-2.099112851	7.097028257	-17.2979249	9.39E-14	2.16E-12	21.56833698
Fam46c	-2.126159332	8.055999939	-17.31988684	9.16E-14	2.12E-12	21.59342806
Agpat3	-2.1317983	7.987993737	-19.67539159	7.62E-15	2.90E-13	24.12682219
Mycl	-2.136958764	6.401485559	-9.959186325	2.49E-09	1.45E-08	11.10409577
Rnf180	-2.139428429	6.716873853	-14.21503317	3.99E-12	5.02E-11	17.72798655
Procr	-2.152914746	7.392912417	-13.29788288	1.39E-11	1.46E-10	16.44695553
Slc22a18	-2.163191936	6.684383083	-11.78730101	1.26E-10	1.03E-09	14.17203537
Bst1	-2.176281183	7.161537891	-13.05113702	1.96E-11	1.97E-10	16.08992076
Id2	-2.180503586	11.0367301	-11.86011077	1.13E-10	9.27E-10	14.28676595
Galnt7	-2.183332483	9.069237934	-26.92972197	1.49E-17	3.50E-15	30.40150543
Plekha5	-2.198832566	7.540841404	-21.27578954	1.63E-15	9.24E-14	25.68871671
Olfr433	-2.226741521	5.299696534	-8.115980475	7.44E-08	3.16E-07	7.606316391
Ocstamp	-2.265282017	5.924041495	-10.08324499	2.01E-09	1.20E-08	11.32429907
Pstpip2	-2.280996814	6.13053436	-8.877847077	1.73E-08	8.51E-08	9.104732456
Ppt1	-2.284388002	10.44517456	-17.2630415	9.76E-14	2.20E-12	21.5284235
Pde8a	-2.290902216	6.828592452	-14.02831718	5.11E-12	6.15E-11	17.47289133
Rasgrp3	-2.293008933	7.940664918	-20.6247331	3.01E-15	1.46E-13	25.06749067
Anxa1	-2.294863371	8.531892019	-21.48463391	1.34E-15	8.31E-14	25.88407152
Cpq	-2.330970097	6.957047967	-17.63408077	6.46E-14	1.54E-12	21.94923343
Tgfbi	-2.347539638	9.265835802	-16.64576812	1.97E-13	3.79E-12	20.80973133
LOC102635290	-2.35434058	6.459515137	-10.03283838	2.19E-09	1.29E-08	11.2350491
Fgl2	-2.355739722	9.684735295	-24.21871434	1.24E-16	1.74E-14	28.28123701
Pde1b	-2.364888107	7.49661626	-13.54655662	9.82E-12	1.08E-10	16.80134856
Tubb6	-2.380070831	7.093336546	-18.35465137	2.97E-14	8.27E-13	22.7436478
Ptger2	-2.410373906	4.84588209	-15.86516983	4.96E-13	8.13E-12	19.86573028
Itgae	-2.414847144	5.754123003	-12.21574405	6.61E-11	5.80E-10	14.83950679
Dgkk	-2.417716166	6.404650083	-15.27389949	1.02E-12	1.53E-11	19.12290217
Sema4f	-2.434330607	5.551904192	-15.21493814	1.10E-12	1.63E-11	19.04745427
Ctla2b	-2.436905262	5.45870636	-8.27640122	5.44E-08	2.38E-07	7.928158809
4932438H23Rik	-2.438230378	7.438397143	-15.46657389	8.06E-13	1.23E-11	19.367693
Dock5	-2.455927814	8.47363497	-20.09355733	5.04E-15	2.16E-13	24.54642335
Tlr4	-2.456617676	5.657269317	-10.60690614	8.30E-10	5.58E-09	12.23387398
Serpinf1	-2.466257391	5.958830018	-11.8032621	1.23E-10	1.01E-09	14.19723236
Myo6	-2.494485151	5.736837342	-11.39916637	2.31E-10	1.76E-09	13.55120992
Alms1	-2.503280528	7.382319374	-18.34784649	2.99E-14	8.27E-13	22.73628198
Mctp1	-2.506898891	8.224223245	-19.5935793	8.27E-15	3.05E-13	24.04373348
Pbx1	-2.513524083	8.320159819	-22.94686613	3.64E-16	3.09E-14	27.20162513
LOC102632708	-2.523788412	5.887268374	-12.58323665	3.84E-11	3.55E-10	15.39767013
Cpne8	-2.550623233	4.713023765	-9.118410878	1.11E-08	5.68E-08	9.56221725
Naaa	-2.589724845	9.558811418	-11.97831494	9.45E-11	7.90E-10	14.47188479
Ncf4	-2.608415333	8.177988902	-19.83898543	6.47E-15	2.62E-13	24.29198438
Adrb2	-2.615509977	7.583183595	-20.31920936	4.04E-15	1.81E-13	24.76938384
Ttc39a	-2.626317356	7.940951292	-19.7733072	6.91E-15	2.74E-13	24.22583335
Cenpv	-2.653592745	6.677468049	-16.24368356	3.16E-13	5.64E-12	20.32853571
Rnf144b	-2.66099105	8.56536867	-34.83467793	8.19E-20	7.72E-17	35.48810295
Gusb	-2.701043883	9.733924042	-37.474373	1.86E-20	3.50E-17	36.90603244
Pianp	-2.712325922	6.269934137	-12.98872946	2.14E-11	2.13E-10	15.99875255
Lsr	-2.712818944	7.842359188	-21.28977455	1.61E-15	9.24E-14	25.70185685
Mnda	-2.745376078	9.177238962	-13.83882091	6.59E-12	7.61E-11	17.21107006
Igf2r	-2.750044058	6.252836095	-16.54992724	2.20E-13	4.13E-12	20.69598837
Mab21l3	-2.75098955	7.347695515	-16.06490599	3.90E-13	6.69E-12	20.11115396
Tbxas1	-2.752707969	5.933410115	-17.22234913	1.02E-13	2.29E-12	21.48177025
Clec9a	-2.765045604	8.891333187	-14.89381326	1.65E-12	2.36E-11	18.63202944
Egr3	-2.77256568	6.959687874	-10.86325212	5.45E-10	3.82E-09	12.66768479
Gramd2	-2.826689956	4.99572626	-19.39463927	1.01E-14	3.56E-13	23.84030353
Cd99l2	-2.837149169	5.818387232	-11.02244877	4.21E-10	3.03E-09	12.93340485
Slc7a8	-2.839955557	5.331741849	-12.08043333	8.10E-11	6.97E-10	14.63068401
Apobr	-2.840366647	7.871915234	-23.43464106	2.40E-16	2.47E-14	27.62262723
Slamf8	-2.864054389	8.378085313	-20.12844024	4.87E-15	2.13E-13	24.58104698
Ppt2	-2.865886498	7.402863745	-13.83789317	6.60E-12	7.61E-11	17.20978089
Pdia5	-2.951703451	7.224826738	-18.83266746	1.80E-14	5.55E-13	23.25478187
Sh3pxd2b	-2.983506643	6.489092709	-20.46667067	3.51E-15	1.64E-13	24.91380175
Plce1	-2.985676995	4.893337261	-21.28871932	1.61E-15	9.24E-14	25.70086567
Klra5	-3.039495838	4.718195931	-7.600674073	2.08E-07	8.11E-07	6.549429149
Homer2	-3.071972653	5.125106947	-15.49385328	7.80E-13	1.20E-11	19.40213546
Cd81	-3.083661078	10.64802317	-19.6454646	7.85E-15	2.93E-13	24.09646656
Asb2	-3.086779866	6.804679942	-22.75327975	4.31E-16	3.40E-14	27.0320539
Arhgap42	-3.124697343	5.257296872	-12.05232241	8.45E-11	7.19E-10	14.58707376
Naalad2	-3.124932391	4.553482999	-12.39401351	5.07E-11	4.57E-10	15.11189324
Adcy4	-3.150212753	5.273531497	-14.24739295	3.82E-12	4.86E-11	17.77190984
Gm6377	-3.15564542	8.684248536	-23.27245521	2.75E-16	2.59E-14	27.48362575
Il12b	-3.183495372	7.579085673	-17.08031274	1.20E-13	2.63E-12	21.31813609

Serpinb8	-3.188757912	4.223466469	-17.53131984	7.24E-14	1.71E-12	21.83350746
4930455G09Rik	-3.231796315	6.326760136	-17.04000651	1.26E-13	2.69E-12	21.27147536
Nlrp1b	-3.260837408	6.982256958	-16.77774784	1.69E-13	3.41E-12	20.96540127
Tlr11	-3.263311523	9.432931584	-23.69390069	1.92E-16	2.20E-14	27.84283025
Cadm1	-3.276277975	8.560750181	-22.195427	7.06E-16	4.75E-14	26.53525806
Fgd6	-3.282000918	6.224018831	-23.96945934	1.53E-16	1.86E-14	28.0742304
Snx24	-3.293909335	4.709116672	-13.85759602	6.43E-12	7.48E-11	17.23714423
Clec1a	-3.300527411	5.909141787	-24.15392726	1.31E-16	1.77E-14	28.22763954
Stfa2l1	-3.306439052	3.659557381	-12.15333564	7.26E-11	6.32E-10	14.74341715
Tmem27	-3.338212281	4.681300129	-17.06920409	1.21E-13	2.65E-12	21.3052861
Dbn1	-3.37545982	6.821907159	-19.34249155	1.06E-14	3.69E-13	23.7866509
Gm10677	-3.416906079	6.472742237	-20.4875224	3.44E-15	1.64E-13	24.93414206
St3gal5	-3.424648518	7.549548803	-22.74747878	4.33E-16	3.40E-14	27.02695039
Notch4	-3.437956308	6.469330801	-23.38048911	2.51E-16	2.49E-14	27.57632394
Zdhhc2	-3.507506227	6.117714766	-8.685099989	2.49E-08	1.17E-07	8.732807407
1700009J07Rik	-3.509571172	6.122544269	-18.32810741	3.05E-14	8.27E-13	22.71490134
Cxcl9	-3.550932868	9.502358201	-12.62608315	3.61E-11	3.38E-10	15.46190748
Gm13710	-3.574874796	5.496354147	-8.932107025	1.57E-08	7.79E-08	9.208567322
Treml4	-3.601217961	8.190843141	-19.23449461	1.19E-14	3.97E-13	23.67509974
A530099J19Rik	-3.619626874	9.049173058	-28.80266011	3.84E-18	1.61E-15	31.74027401
Slc8b1	-3.656038922	8.684286706	-38.87022229	8.82E-21	3.32E-17	37.61004866
Gm11837	-3.713664353	5.469303034	-20.80985691	2.53E-15	1.29E-13	25.24604938
Fndc7	-3.74914872	6.347289666	-27.71878266	8.31E-18	2.41E-15	30.9771377
Ppap2a	-3.758941226	6.784354675	-23.52375672	2.22E-16	2.39E-14	27.69859315
Ifi205	-3.834023848	9.057853111	-12.02887753	8.75E-11	7.38E-10	14.55064211
Clnk	-3.838000968	6.504583493	-20.75552735	2.66E-15	1.34E-13	25.19380665
Nlrp1c-ps	-3.882846107	6.660966512	-9.768062422	3.47E-09	1.96E-08	10.76123435
Mpzl2	-3.916224796	6.129227521	-21.78175773	1.02E-15	6.66E-14	26.15882022
Clec4b2	-3.988113943	5.638683402	-18.75452384	1.95E-14	5.92E-13	23.17206193
Itga8	-4.011271039	5.857852783	-21.13785594	1.86E-15	9.99E-14	25.55866235
Gatm	-4.134419578	6.440858061	-23.33520768	2.61E-16	2.52E-14	27.53752275
Fam149a	-4.204317814	6.024618139	-23.79887941	1.76E-16	2.08E-14	27.93130478
Gm3336	-4.205791315	5.6643712	-18.47488078	2.61E-14	7.57E-13	22.87336941
Trpm2	-4.221347463	6.134968766	-21.72766965	1.08E-15	6.88E-14	26.10908038
Snx22	-4.243092789	6.735469022	-24.72496789	8.22E-17	1.42E-14	28.69510507
Gm4524	-4.363335638	5.802997272	-18.39715112	2.84E-14	8.13E-13	22.78959333
Cd207	-4.65890275	6.744875284	-28.35517508	5.26E-18	1.80E-15	31.428961
Ppef2	-4.801127998	6.291472617	-23.99776735	1.49E-16	1.86E-14	28.09784966
Cd36	-4.835242103	7.516305538	-32.74811378	2.87E-19	2.16E-16	34.27844793
Leprel1	-4.907533782	7.262982211	-24.02352637	1.46E-16	1.86E-14	28.11931764
Gpr33	-5.144227499	7.530817407	-35.71348648	4.94E-20	6.21E-17	35.97340009
Gcsam	-5.159051435	6.76258887	-19.19883977	1.23E-14	4.04E-13	23.63814118
Tlr3	-5.22705225	7.531004467	-12.90489894	2.42E-11	2.36E-10	15.87573117
5430435G22Rik	-5.287981045	6.874645864	-28.50035348	4.75E-18	1.79E-15	31.53052974
Hepacam2	-5.707386879	6.008295626	-28.85610696	3.70E-18	1.61E-15	31.77711221
Xcr1	-5.757439844	7.982433709	-31.43675752	6.56E-19	4.12E-16	33.47357993
