# Supplementary material for: CD8+XCR1neg Dendritic Cells Express High Levels of Toll-Like Receptor 5 and a Unique Complement of Endocytic Receptors
Source: Front Immunol. 2019 Jan 16;9:2990. doi: 10.3389/fimmu.2018.02990 (PMC6343586; doi:10.3389/fimmu.2018.02990)
Supplement: Supplementary Table 4 — Genes used in cDC, CD8+ DC and pDC gene signatures. Gene sets with specific expression in cDC, CD8+DC or pDC subsets were compiled using data from Miller et al. (31). Subset specific signature genes were used to create heatmaps of differential gene expression across DC subsets in the microarray. [file Table_4.PDF]

Table S4. Genes used in cDC, CD8+ DC and pDC gene signatures.

Gene sets with specific expression in cDC, CD8+ DC or pDC subsets were compiled using data from Miller et al., 2012.

Subset specific signature genes were used to create heatmaps of differential gene expression across DC subsets in the microarray

**cDC Gene Signature**	**CD8+ DC Gene Signature**	**pDC Gene Signature**
1700025G04Rik	1700009J07Rik	1190002A17Rik
2810417H13Rik	5430435G22Rik	1700024P16Rik
4930506M07Rik	A530099J19Rik	2210020M01Rik
5033414K04Rik	Aif1	2610018G03Rik
Adam23	Alms1	4930572J05Rik
Adam8	Cd36	A130050O07Rik
Aim1	Dbn1	Abca5
Anpep	Fam149a	Ablim1
Anxa1	Fgd6	Angptl7
Arhgap26	Fzd1	Ap1m2
Arrb1	Gpr33	Arhgap24
Asap1	Hepacam2	Asph
Asb2	Htr7	Blnk
Atf3	Ifi205	Cacna1d
Atp8b4	Igf2r	Card6
Batf3	Ipcef1	Cdc14b
Bcl2a1a	Itga1	Cdh1
Bcl2a1b	Itgae	Clec4g
Bcl2a1c	Leprel1	Cmah
Bcl2a1d	Met	Dnajc13
Bub1	Ppap2a	Duxbl
Bub1b	Sh3pxd2b	Ear14
C130092O11Rik	Slc22a21	Efcab4b
Ccna2	Snx22	Erbb3
Ccnb2	Tlr3	Eya1
Ccrl2	Tmeff1	Fads3
Cd80	Ttc39a	Fam169b
Cd81	Xcr1	Fggy
Cd83		Figf
Cd86	**No. Genes: 28**	Gcom1
Cd9		Gimap1
Cdca7		Gimap9
Cep55		Gm10790
Chn2		Gm10838
Cxcl16		Gm12253
Ddef2		Gm14207
Dlgap5		Gm6498
Dna2		Gpr174
Dock5		Gria3
Dock7		Grm8

E2f8		Hs3st1
Ear2		Hsd11b1
Egr1		Igf1r
Eps8		Ikzf2
Errfi1		Ikzf3
Fam38a		Il17rb
Fgl2		Itsn1
Gatm		Klk1
Gm11545		Klk1b21
Gm6377		Klra17
Gpr124		Ldhb
Gpr82		Lynx1
Grk5		Maged1
Havcr2		Mcart6
Hk2		Mctp2
Hnrpll		Mgst2
Id2		Obscn
Ifitm2		Olfr164
Igsf6		Olfr165
Il1b		Olfr166
Kif11		P2ry12
Kif23		Pacsin1
Klri1		Paqr5
Lfng		Pdzd4
Lpcat2		Peli2
Lpcat4		Pkig
Lrrc25		Plod2
Lrrk2		Pnck
Lrrk2		Ppm1e
Ly75		Ppm1l
Lyz1		Prkca
Mctp1		Prrg4
Melk		Rpgrip1
Mki67		Scrn1
Mllt4		Sit1
Myc		Slc39a14
Nav1		Slco4a1
Ncapg		Srl
Nck2		St3gal6
Nck2		Tmem163
Neurl3		Upb1
Nlrp1b		Wfs1
Nlrp1c		Xkrx
Nostrin		Ysk4
Nr4a3		Zc3h12b
Nuf2		Zcchc24
Pak1		Zfp521
Parp12		Zfp658
Pglyrp1		

Pole<br>Prkar2a<br>Pstpip2<br>Ptpn22<br>Pvrl1<br>Rab11fip1<br>Rab31<br>Rab32<br>Ralb<br>Rasgef1b<br>Rasgrp3<br>Rnf144b<br>Rnf150<br>Rrm2<br>Rtn1<br>Sash1<br>Sgk1<br>Slamf8<br>Spint1<br>Spire1<br>Spred1<br>St3gal5<br>Sulf2<br>Tgfbi<br>Tlr11<br>Tlr13<br>Tnfrsf21<br>Top2a<br>Tpx2<br>Tspan33<br>Ttc39b<br>Ttc39b<br>Tubb6<br>Wdfy3<br>Zbtb46<br>Zfp366<br><br>**No. Genes: 125**		**No. Genes: 88**
