# Supplementary material for: CD8+XCR1neg Dendritic Cells Express High Levels of Toll-Like Receptor 5 and a Unique Complement of Endocytic Receptors
Source: Front Immunol. 2019 Jan 16;9:2990. doi: 10.3389/fimmu.2018.02990 (PMC6343586; doi:10.3389/fimmu.2018.02990)
Supplement: Supplementary Table 5 — Gene set enrichment analysis of genes upregulated in CD8+XCR1neg vs. CD8+XCR1+ DC. Gene set erichment analysis was carried out on genes upregulated with a FC >2 and adj. P–value < 0.05 (n = 601) using Enrichr to query. [file Table_5.PDF]

Table S5. Gene set enrichment analysis of genes upregulated in CD8+XCR1neg vs. CD8+XCR1+ DC

Gene set erichment analysis was carried out on genes upregulated with a FC>2 and adj. P-value<0.05 (n=601) using Enrichr to query:

The Mouse Genome Atlas

Term	Overlap	Gene Symbols	Adjusted P-value
dendritic_plasmacytoid_B220+	36/214	L3MBTL3;DCLRE1C;LOC73899;CLEC10A;SLA2;SIGLECH;LRP8;XKRX;CCR9;CTSH;KLK1B27;DIRC2;HAVCR1;LAIR1;CD209D;CD209E;LAG3;CD209C;ST8SIA1;SEMA4B;KLRA17;TRAPPC5;CD209A;RUNX2;FGR;EEPD1;CD4;SNX18;CD7;GM5547;TUBGCP5;TLR7;TCF4;RELL1;MCTP2;CLEC4G	3.65E-11
dendritic_cells_myeloid_CD8a-	31/188	SIRPB1A;DOCK4;CLEC4A3;LOC73899;SLCO5A1;MEFV;RELB;REM1;5430437J10RIK;RAP1A;PTCHD1;MARVELD1;CTSH;ST8SIA6;CD209E;CD209C;ST8SIA1;IL1R1;TRAPPC5;ABCA9;EMR4;CD209A;FGR;CCDC88A;CYP4F16;TBC1D4;AEBP2;GPR183;CD7;TLR5;BEX6	5.73E-07
lymph_nodes	15/90	ITK;TCF7;GIMAP3;TIMD4;TMEM173;SIGLECH;CD3D;CMAH;SLC4A8;CD4;TBC1D4;ABI3;IL2RB;FSCN1;NUDT17	2.53E-03

KEGG Pathways 2016

Term	Overlap	Gene Symbols	Adjusted P-value
Hematopoietic cell lineage	18/88	CSF1R;ITGAM;TFRC;IL1R1;IL1R2;DNTT;CD3G;CD3E;CD3D;CD2;CD4;CD5;IL2RA;CD7;CD14;IL7R;IL9R;CD33	2.29E-08
Cytokine-cytokine receptor interaction	27/265	CX3CR1;CSF1R;CXCL1;TNFRSF11A;IL2RG;CXCL2;IL21R;KDR;CCR9;CCR7;CCL17;IL1R1;IL1R2;TNFRSF9;TNFRSF18;LIFR;TNFRSF1B;ACVR2A;IL17RA;IL2RA;IL2RB;TNFSF9;FAS;LTB;IL6ST;IL7R;IL9R	2.50E-06
NF-kappa B signaling pathway	16/93	TICAM2;GADD45B;IL1R1;DDX58;PRKCB;TNFAIP3;TNFRSF11A;PTGS2;CXCL2;MALT1;RELB;LCK;BLNK;LTB;CD14;LAT	1.75E-06
Primary immunodeficiency	9/37	CD4;DCLRE1C;LCK;AIRE;BLNK;IL7R;IL2RG;CD3E;CD3D	5.96E-05
Measles	16/136	STAT5A;DDX58;TNFAIP3;PIK3R3;CD3G;IL2RG;CD3E;CD3D;CCND2;OAS2;IL2RA;IL2RB;CD28;FAS;TLR7;FYN	1.46E-04
Lysosome_Homo sapiens	15/123	CTSA;CD164;CD63;SORT1;GAA;CTSW;LAMP1;NPC1;CTSL;ACP5;CTSH;TPP1;CTSD;CTSC;LGMN	1.56E-04
T cell receptor signaling pathway	13/104	ITK;PIK3R3;CD3G;CD3E;RASGRP1;CD3D;MALT1;CD4;TEC;LCK;CD28;FYN;LAT	4.41E-04
Osteoclast differentiation	14/132	CSF1R;IL1R1;NCF2;CYBB;PIK3R3;TNFRSF11A;LILRB4;RELB;TEC;LCK;BLNK;SIRPA;ACP5;FYN	1.20E-03
Cell adhesion molecules (CAMs)	14/142	CD274;CADM3;ITGAM;SDC4;PDCD1LG2;L1CAM;PTPRF;CD2;SPN;CD4;SELL;CDH1;PECAM1;CD28	2.37E-03
Choline metabolism in cancer	11/101	SLC44A5;PLA2G4F;DGKD;PCYT1A;CHKA;PRKCB;SLC44A2;RAC2;PIK3R3;PRKCA;GPCPD1	4.49E-03
Leukocyte transendothelial migration	12/118	ITK;RAP1A;ITGAM;PRKCB;NCF2;PECAM1;CTNNA1;RAC2;CYBB;PIK3R3;PRKCA;THY1	4.49E-03
Chemokine signaling pathway	15/187	CX3CR1;ITK;PRKCB;PIK3R3;CXCL1;RASGRP2;CXCL2;ADCY6;FGR;GNGT2;RAP1A;CCR9;RAC2;CCR7;CCL17	9.08E-03
Endocrine and other factor-regulated calcium reabsorption	9/37	KLK1;PRKCB;FXYD2;ATP1A3;PRKCA;ATP1B1;ADCY6	8.51E-03

GO Biological Processes

Term	Overlap	Gene Symbols	Adjusted P-value
regulation of cell activation (GO:0050865)	47/420	CD274;PGLYRP2;TRAC;TNFAIP3;CD3G;THY1;PRDM1;CD3E;SLA2;CD3D;SAMSN1;MALT1;SPN;IL1RL2;LGALS1;TESPA1;LAMP1;HLX;TRBC1;CLPTM1;CCR7;FYN;STAT5A;PRNP;MEF2C;LAG3;FCER1G;CD300A;IL31RA;PRKCA;PDCD1LG2;CD2;FGR;CD4;TEC;ADORA2A;LCK;CD5;IRF4;GPR183;IL2RA;CD28;FAS;TNFSF9;IL6ST;IL7R;LAT	1.16E-11
regulation of leukocyte activation (GO:0002694)	45/390	CD274;PGLYRP2;TRAC;TNFAIP3;CD3G;THY1;PRDM1;CD3E;SLA2;CD3D;SAMSN1;MALT1;SPN;IL1RL2;LGALS1;LAMP1;TESPA1;HLX;TRBC1;CLPTM1;CCR7;FYN;STAT5A;PRNP;MEF2C;LAG3;FCER1G;CD300A;IL31RA;PDCD1LG2;CD2;FGR;CD4;ADORA2A;LCK;CD5;IRF4;GPR183;IL2RA;CD28;FAS;TNFSF9;IL6ST;IL7R;LAT	1.16E-11
regulation of lymphocyte activation (GO:0051249)	42/344	CD274;PGLYRP2;TRAC;TNFAIP3;CD3G;THY1;PRDM1;CD3E;SLA2;SAMSN1;CD3D;MALT1;SPN;IL1RL2;LGALS1;TESPA1;LAMP1;HLX;TRBC1;CLPTM1;FYN;CCR7;STAT5A;PRNP;MEF2C;LAG3;CD300A;PDCD1LG2;CD2;CD4;ADORA2A;IRF4;LCK;CD5;GPR183;IL2RA;CD28;FAS;TNFSF9;IL6ST;IL7R;LAT	1.16E-11
leukocyte activation (GO:0045321)	43/373	PTGER4;CX3CR1;ITK;ITGAM;DCLRE1C;SATB1;LEF1;TCF7;CD3G;CD3E;SLA2;RASGRP1;CLU;CD3D;MALT1;RELB;LGALS1;CLEC7A;IL21R;BLNK;FYN;SOX4;STAT5A;MEF2C;FCER1G;PRKCB;ANXA3;RUNX2;FOXP1;BST2;CD2;CD4;IRF4;LCK;GPR183;CD7;BCL3;LY6D;CD28;TNFSF9;TLR7;IL7R;LAT	3.21E-11
lymphocyte activation (GO:0046649)	36/304	PTGER4;ITK;ITGAM;DCLRE1C;SATB1;LEF1;TCF7;CD3G;CD3E;SLA2;CD3D;MALT1;RELB;LGALS1;CLEC7A;IL21R;BLNK;FYN;SOX4;STAT5A;MEF2C;PRKCB;RUNX2;FOXP1;BST2;CD2;CD4;LCK;IRF4;GPR183;CD7;BCL3;LY6D;CD28;IL7R;LAT	1.45E-09
regulation of T cell activation (GO:0050863)	32/259	CD274;TRAC;CD3G;THY1;CD3E;CD3D;MALT1;SPN;IL1RL2;LGALS1;TESPA1;HLX;TRBC1;CLPTM1;FYN;STAT5A;PRNP;LAG3;CD300A;PDCD1LG2;CD2;CD4;ADORA2A;LCK;CD5;IRF4;IL2RA;CD28;TNFSF9;IL6ST;IL7R;LAT	6.64E-09
positive regulation of leukocyte activation (GO:0002696)	32/262	CD274;TRAC;CD3G;THY1;PRDM1;CD3E;CD3D;MALT1;SPN;IL1RL2;LGALS1;TESPA1;LAMP1;HLX;TRBC1;CCR7;FYN;STAT5A;MEF2C;FCER1G;PDCD1LG2;CD2;FGR;CD4;LCK;CD5;GPR183;IL2RA;CD28;TNFSF9;IL6ST;IL7R	7.74E-09
positive regulation of cell activation (GO:0050867)	32/272	CD274;TRAC;CD3G;THY1;PRDM1;CD3E;CD3D;MALT1;SPN;IL1RL2;LGALS1;TESPA1;LAMP1;HLX;TRBC1;CCR7;FYN;STAT5A;MEF2C;FCER1G;PDCD1LG2;CD2;FGR;CD4;LCK;CD5;GPR183;IL2RA;CD28;TNFSF9;IL6ST;IL7R	1.62E-08
leukocyte differentiation (GO:0002521)	31/251	PTGER4;CSF1R;ITK;L3MBTL3;DCLRE1C;TFRC;SATB1;LEF1;TCF7;TNFRSF11A;CD3D;MALT1;RELB;LGALS1;BLNK;CCR7;SOX4;STAT5A;MEF2C;IL31RA;RUNX2;FOXP1;CD4;LCK;IRF4;GPR183;BCL3;LY6D;CD28;TNFSF9;IL7R	1.06E-08

regulation of cytokine production (GO:0001817)	43/482	PTGER4;CD274;CSF1R;PGLYRP2;LEF1;TNFAIP3;CD3E;PTGS2;MEFV;CLU;MALT1;SPN;PANX1;IL1RL2;ACP5;APOD;CCR7;CD14;STAT5A;PRNP;LAG3;TICAM2;FCER1G;DDX58;IRAK3;KLF4;TMEM173;SULF2;IL17RA;BST2;FGR;CD2;CD4;IRF4;BCL3;CD28;TNFSF9;TLR7;LTB;HPSE;ACKR1;IL6ST;TLR5	6.68E-08
immune response-activating signal transduction (GO:0002757)	36/440	ITK;PGLYRP2;ITGAM;TRAC;TNFAIP3;CD3G;THY1;CD3E;SLA2;CD3D;MALT1;CD79B;IGKC;CTSL;CLEC7A;TRBC1;FYN;CD14;MEF2C;FCER1G;TICAM2;DDX58;PRKCB;RFTN1;IRAK3;FGR;CD4;CACNB3;TEC;LCK;TNIP3;CD28;TLR7;TLR5;LAT;LGMN	9.18E-06
positive regulation of lymphocyte activation (GO:0051251)	29/243	CD274;TRAC;CD3G;THY1;PRDM1;CD3E;CD3D;MALT1;SPN;IL1RL2;LGALS1;TESPA1;LAMP1;HLX;TRBC1;CCR7;FYN;STAT5A;MEF2C;PDCD1LG2;CD4;LCK;CD5;GPR183;IL2RA;CD28;TNFSF9;IL6ST;IL7R	7.39E-08
activation of immune response (GO:0002253)	38/487	ITK;ITGAM;PGLYRP2;TRAC;TNFAIP3;CD3G;THY1;CD3E;SLA2;CD3D;CLU;MALT1;CD79B;IGKC;CTSL;CLEC7A;TRBC1;FYN;CD14;MEF2C;TICAM2;FCER1G;DDX58;PRKCB;RFTN1;IRAK3;TMEM173;FGR;CD4;CACNB3;TEC;LCK;TNIP3;CD28;TLR7;TLR5;LAT;LGMN	1.22E-05
T cell activation (GO:0042110)	26/198	PTGER4;ITK;ITGAM;SATB1;LEF1;TCF7;CD3G;CD3E;SLA2;CD3D;MALT1;RELB;CLEC7A;FYN;SOX4;STAT5A;RUNX2;CD2;CD4;LCK;IRF4;CD7;BCL3;CD28;IL7R;LAT	7.39E-08
positive regulation of defense response (GO:0031349)	30/272	PTGER4;ITGAM;PGLYRP2;TNFAIP3;TNFRSF11A;DDX60;PTGS2;LAMP1;CLEC7A;CTSL;CCR7;CD14;STAT5A;MEF2C;LAG3;FCER1G;TICAM2;DDX58;RFTN1;IRAK3;PRKCA;TMEM173;IL17RA;FABP4;TNIP3;CD28;TLR7;IL6ST;TLR5;LGMN	2.20E-07
antigen receptor-mediated signaling pathway (GO:0050851)	20/127	ITK;MEF2C;PRKCB;TRAC;RFTN1;CD3G;THY1;CD3E;SLA2;CD3D;MALT1;CD79B;CD4;CACNB3;TEC;LCK;TRBC1;CD28;FYN;LAT	2.95E-07
positive regulation of T cell activation (GO:0050870)	24/189	STAT5A;CD274;TRAC;CD3G;THY1;PDCD1LG2;CD3E;CD3D;MALT1;SPN;IL1RL2;CD4;LGALS1;TESPA1;HLX;LCK;CD5;IL2RA;TRBC1;CD28;TNFSF9;FYN;IL6ST;IL7R	5.69E-07
positive regulation of cytokine production (GO:0001819)	31/327	PTGER4;CD274;CSF1R;CD3E;PTGS2;CLU;MALT1;SPN;PANX1;IL1RL2;CCR7;CD14;STAT5A;TICAM2;FCER1G;DDX58;TMEM173;SULF2;IL17RA;CD2;FGR;CD4;IRF4;BCL3;CD28;TNFSF9;TLR7;LTB;HPSE;IL6ST;TLR5	3.20E-06
cytokine-mediated signaling pathway (GO:0019221)	32/342	IFITM3;CX3CR1;CSF1R;IFITM1;EIF4E3;IFITM2;PTAFR;TNFRSF11A;IL2RG;LRP8;IL1RL2;STAT4;IL21R;CCR9;CCR7;STAT5A;IL1R1;IL1R2;TNFRSF18;IL31RA;LIFR;IRAK3;TNFRSF1B;IL17RA;OAS2;IRF4;IL2RA;IL2RB;ACKR1;IL6ST;IL7R;IL9R	2.73E-06
lymphocyte differentiation (GO:0030098)	22/172	STAT5A;PTGER4;ITK;DCLRE1C;SATB1;TCF7;LEF1;CD3D;RUNX2;MALT1;RELB;FOXP1;CD4;LGALS1;LCK;GPR183;BCL3;CD28;LY6D;BLNK;IL7R;SOX4	2.14E-06
negative regulation of immune system process (GO:0002683)	29/311	PTGER4;CD274;PGLYRP2;TNFAIP3;THY1;PRDM1;SLA2;SAMSN1;SPN;HLX;APOD;STAT5A;PRNP;LAG3;TICAM2;FCER1G;CD300A;TMEM176B;TMEM176A;IL31RA;IRAK3;PDCD1LG2;LILRB4;BST2;ADORA2A;IRF4;IL2RA;FAS;IL7R	1.08E-05
regulation of inflammatory response (GO:0050727)	25/247	PTGER4;PGLYRP2;PROS1;TNFAIP3;TNFRSF11A;PTGS2;MEFV;SPN;IL1RL2;ACP5;APOD;CCR7;STAT5A;FCER1G;IL1R1;PRKCA;TNFRSF1B;KLF4;IL17RA;FABP4;ADORA2A;IL2RA;CD28;TLR7;IL6ST	1.65E-05
T cell costimulation (GO:0031295)	14/76	CD274;TRAC;CD3G;PDCD1LG2;CD3E;CD3D;SPN;CD4;LGALS1;CD5;LCK;TRBC1;CD28;FYN	8.53E-06
lymphocyte costimulation (GO:0031294)	14/77	CD274;TRAC;CD3G;PDCD1LG2;CD3E;CD3D;SPN;CD4;LGALS1;CD5;LCK;TRBC1;CD28;FYN	9.18E-06
cellular response to cytokine stimulus (GO:0071345)	36/471	IFITM3;CX3CR1;CSF1R;IFITM1;EIF4E3;IFITM2;NPR2;LEF1;TCF7;PTAFR;TNFRSF11A;IL2RG;LRP8;IL1RL2;IL21R;STAT4;CCR9;CCR7;STAT5A;IL1R1;TNFRSF18;IL1R2;IL31RA;LIFR;IRAK3;TMEM173;TNFRSF1B;IL17RA;IRF4;OAS2;IL2RA;IL2RB;ACKR1;IL6ST;IL7R;IL9R	3.63E-05
regulation of immune effector process (GO:0002697)	25/264	PGLYRP2;PROS1;TNFAIP3;DDX60;MALT1;SPN;LAMP1;HLX;FYN;STAT5A;LAG3;FCER1G;DDX58;CD300A;IRAK3;TMEM173;FOXP1;BST2;FGR;CD4;LCK;IRF4;IL2RA;CD28;IL7R	4.69E-05
T cell differentiation (GO:0030217)	15/97	STAT5A;PTGER4;ITK;SATB1;LEF1;TCF7;CD3D;RUNX2;RELB;CD4;LCK;BCL3;CD28;IL7R;SOX4	2.36E-05
T cell receptor signaling pathway (GO:0050852)	15/99	ITK;TRAC;RFTN1;CD3G;THY1;CD3E;CD3D;MALT1;CD4;CACNB3;LCK;TRBC1;CD28;FYN;LAT	2.99E-05
negative regulation of lymphocyte activation (GO:0051250)	15/102	PRNP;CD274;LAG3;PGLYRP2;CD300A;TNFAIP3;PRDM1;PDCD1LG2;SLA2;SAMSN1;SPN;ADORA2A;HLX;IL2RA;FAS	4.14E-05
cellular response to interleukin-15 (GO:0071350)	6/10	IL2RA;IL2RB;IL21R;IL7R;IL2RG;IL9R	1.70E-05
response to interleukin-15 (GO:0070672)	6/13	IL2RA;IL2RB;IL21R;IL7R;IL2RG;IL9R	1.00E-04
negative regulation of leukocyte activation (GO:0002695)	16/120	PRNP;CD274;LAG3;PGLYRP2;CD300A;IL31RA;TNFAIP3;PRDM1;PDCD1LG2;SLA2;SAMSN1;SPN;ADORA2A;HLX;IL2RA;FAS	6.27E-05
regulation of response to wounding (GO:1903034)	28/347	PTGER4;PGLYRP2;PROS1;TNFAIP3;TNFRSF11A;PTGS2;MEFV;SPN;IL1RL2;ACP5;APOD;CCR7;STAT5A;FCER1G;IL1R1;PLAUR;PRKCA;TNFRSF1B;KLF4;IL17RA;FABP4;TEC;ADORA2A;IL2RA;CD28;TLR7;HPSE;IL6ST	2.23E-04
blood coagulation (GO:0007596)	34/472	CD63;ITGAM;DGKD;PROS1;SLC7A11;RASGRP2;LRRC16A;RASGRP1;CLU;LRP8;FBLN5;PHF21A;PAPSS2;SPN;RAP1A;RAC2;SIRPA;FYN;P2RY12;FCER1G;PRKCB;PLAUR;ATP2B4;PRKCA;L1CAM;ATP1B1;FGR;CD2;ADORA2A;SELL;LCK;PECAM1;LAT;CD244	2.33E-04
coagulation (GO:0050817)	34/472	CD63;ITGAM;DGKD;PROS1;SLC7A11;LRRC16A;RASGRP2;RASGRP1;LRP8;CLU;FBLN5;PHF21A;PAPSS2;SPN;RAP1A;RAC2;SIRPA;FYN;P2RY12;FCER1G;PRKCB;PLAUR;ATP2B4;PRKCA;L1CAM;ATP1B1;FGR;CD2;ADORA2A;SELL;LCK;PECAM1;LAT;CD244	2.33E-04
hemostasis (GO:0007599)	34/478	CD63;ITGAM;DGKD;PROS1;SLC7A11;RASGRP2;LRRC16A;RASGRP1;CLU;LRP8;FBLN5;PHF21A;PAPSS2;SPN;RAP1A;RAC2;SIRPA;FYN;P2RY12;FCER1G;PRKCB;PLAUR;ATP2B4;PRKCA;L1CAM;ATP1B1;FGR;CD2;ADORA2A;SELL;LCK;PECAM1;LAT;CD244	2.91E-04
negative regulation of cell activation (GO:0050866)	16/135	PRNP;CD274;LAG3;PGLYRP2;CD300A;IL31RA;TNFAIP3;PRDM1;PDCD1LG2;SLA2;SAMSN1;SPN;ADORA2A;HLX;IL2RA;FAS	2.65E-04
immune response-activating cell surface receptor signaling pathway (GO:0002429)	23/324	ITK;MEF2C;FCER1G;PRKCB;TRAC;RFTN1;CD3G;THY1;CD3E;SLA2;CD3D;MALT1;FGR;CD79B;CD4;CACNB3;TEC;IGKC;LCK;TRBC1;CD28;FYN;LAT	6.23E-03

positive regulation of innate immune response (GO:0045089)	19/190	STAT5A;MEF2C;LAG3;ITGAM;PGLYRP2;TICAM2;DDX58;RFTN1;TNFAIP3;IRAK3;TMEM173;LAMP1;CLEC7A;CTSL;TNIP3;TLR7;CD14;TLR5;LGMN	3.99E-04
regulation of interleukin-2 biosynthetic process (GO:0045076)	6/19	STAT5A;LAG3;CD4;IRF4;CD28;CD3E	9.66E-04
regulation of interleukin-2 production (GO:0032663)	9/45	STAT5A;PRNP;LAG3;CD4;IRF4;CD28;TNFAIP3;CD3E;MALT1	4.99E-04
regulation of T cell receptor signaling pathway (GO:0050856)	7/25	PRNP;TESPA1;LCK;CD300A;CCR7;THY1;MALT1	4.99E-04
regulation of antigen receptor-mediated signaling pathway (GO:0050854)	8/35	PRNP;TESPA1;PRKCB;LCK;CD300A;CCR7;THY1;MALT1	5.54E-04
T cell selection (GO:0045058)	7/27	SPN;CD4;CD28;FAS;CCR7;CD3E;CD3D	8.34E-04
thymic T cell selection (GO:0045061)	6/20	SPN;CD28;FAS;CCR7;CD3E;CD3D	1.24E-03
positive regulation of inflammatory response (GO:0050729)	12/88	PTGER4;STAT5A;FCER1G;FABP4;CD28;TLR7;CCR7;PRKCA;TNFRSF11A;PTGS2;IL6ST;IL17RA	9.01E-04
activation of innate immune response (GO:0002218)	16/151	MEF2C;ITGAM;TICAM2;PGLYRP2;DDX58;RFTN1;TNFAIP3;IRAK3;TMEM173;CTSL;CLEC7A;TNIP3;TLR7;CD14;TLR5;LGMN	9.24E-04
regulation of leukocyte differentiation (GO:1902105)	19/210	STAT5A;PGLYRP2;TMEM176B;TMEM176A;LEF1;PRKCA;PRDM1;LILRB4;CD2;IL1RL2;TESPA1;IRF4;LCK;HLX;IL2RA;CLPTM1;TNFSF9;FAS;IL7R	1.28E-03
positive regulation of interleukin-2 biosynthetic process (GO:0045086)	5/13	STAT5A;CD4;IRF4;CD28;CD3E	1.46E-03
leukocyte migration (GO:0050900)	20/226	CX3CR1;ITGAM;FCER1G;PROS1;PTPRO;PRKCA;TNFRSF11A;SLC7A11;L1CAM;ATP1B1;SPN;CD2;SELL;LCK;PECAM1;S1PR1;SIRPA;CCR7;FYN;CD244	1.15E-03
regulation of response to biotic stimulus (GO:0002831)	13/107	DDX58;LY86;TNFAIP3;PRKCA;PRDM1;TMEM173;DDX60;SPN;CD4;LCK;IL2RA;CD28;FYN	1.24E-03
pattern recognition receptor signaling pathway (GO:0002221)	15/142	MEF2C;ITGAM;TICAM2;PGLYRP2;DDX58;RFTN1;TNFAIP3;IRAK3;CTSL;CLEC7A;TNIP3;TLR7;CD14;TLR5;LGMN	1.46E-03
regulation of lymphocyte differentiation (GO:0045619)	14/124	STAT5A;PGLYRP2;PRDM1;CD2;IL1RL2;TESPA1;HLX;LCK;IRF4;IL2RA;CLPTM1;TNFSF9;FAS;IL7R	1.34E-03
positive regulation of response to external stimulus (GO:0032103)	18/201	PTGER4;STAT5A;FCER1G;DSCAM;LY86;PRKCA;TNFRSF11A;PTGS2;DDX60;IL17RA;FABP4;CD28;S1PR1;KDR;RAC2;TLR7;CCR7;IL6ST	2.14E-03
negative thymic T cell selection (GO:0045060)	5/12	SPN;CD28;FAS;CCR7;CD3E	1.09E-03
innate immune response-activating signal transduction (GO:0002758)	15/144	MEF2C;ITGAM;TICAM2;PGLYRP2;DDX58;RFTN1;TNFAIP3;IRAK3;CTSL;CLEC7A;TNIP3;TLR7;CD14;TLR5;LGMN	1.68E-03
negative T cell selection (GO:0043383)	5/13	SPN;CD28;FAS;CCR7;CD3E	1.46E-03
negative regulation of locomotion (GO:0040013)	18/204	PTGER4;IFITM3;CX3CR1;IFITM1;IFITM2;LRP1;CD300A;SEMA6D;PTPRO;THY1;KLF4;PODN;SMAD7;BST2;RAP2B;ADORA2A;CTNNA1;APOD	2.56E-03
defense response to other organism (GO:0098542)	24/328	APOBEC1;IFITM3;IFITM1;PGLYRP2;IFITM2;FCER1G;MMP7;ANXA3;DDX60;TMEM173;SPN;BST2;PLAC8;FGR;CD4;CLEC7A;OAS2;IRF4;BCL3;DDIT4;ACP5;TLR7;TLR5;HIST1H2BC	3.18E-03
alpha-beta T cell differentiation (GO:0046632)	7/34	PTGER4;ITK;SATB1;TCF7;LEF1;BCL3;RELB	3.05E-03
response to other organism (GO:0051707)	30/462	IFITM3;IFITM1;PGLYRP2;IFITM2;DDX60;CLU;MALT1;SPN;PLAC8;CLEC7A;ACP5;HAVCR1;APOBEC1;MEF2C;FCER1G;MMP7;ANXA3;DDX58;IRAK3;TMEM173;BST2;FGR;CD4;IRF4;OAS2;DDIT4;BCL3;TLR7;TLR5;HIST1H2BC	3.61E-03
regulation of cell adhesion (GO:0030155)	24/336	DDR1;STAT5A;CD164;DSCAM;SDC4;LEF1;PTPRO;NDNF;PRKCA;LRRC16A;L1CAM;KLF4;SMAD7;SPN;NUAK1;DAB2;LGALS1;CDH1;KDR;S1PR1;RAC2;APOD;CCR7;GPM6B	4.33E-03
inflammatory response (GO:0006954)	26/376	CSF1R;PTAFR;TNFAIP3;CXCL1;PTGS2;MEFV;CXCL2;RASGRP1;NDST1;MMP25;CLEC7A;BLNK;CCR7;CD14;CCL17;TICAM2;LY86;CYBB;TNFRSF1B;ADORA2A;IL2RA;TNIP3;TLR7;ACKR1;TLR5;LAT	3.88E-03
regulation of innate immune response (GO:0045088)	20/254	STAT5A;MEF2C;LAG3;ITGAM;PGLYRP2;TICAM2;DDX58;RFTN1;TNFAIP3;IRAK3;TMEM173;FGR;LAMP1;CTSL;CLEC7A;TNIP3;TLR7;CD14;TLR5;LGMN	4.40E-03
positive regulation of cytokine biosynthetic process (GO:0042108)	9/59	SPN;STAT5A;CD4;IRF4;BCL3;CD28;TLR7;LTB;CD3E	3.21E-03

regulation of cytokine biosynthetic process (GO:0042035)	11/93	SPN;STAT5A;LAG3;CD4;IRF4;BCL3;CD28;TLR7;LTB;CD3E;KLF4	5.09E-03
response to molecule of bacterial origin (GO:0002237)	19/243	PTGER4;CX3CR1;MEF2C;TICAM2;NCF2;PTAFR;TNFAIP3;IRAK3;ADM;TNFRSF11A;TNFRSF1B;PTGS2;CXCL2;MALT1;TNIP3;ACP5;CCR7;CD14;TLR5	6.33E-03
taxis (GO:0042330)	20/263	CX3CR1;DOCK4;NRP2;ITGAM;FCER1G;LEF1;PTAFR;PTPRO;PLAUR;CXCL1;PRKCA;TNFRSF11A;L1CAM;CXCL2;SPN;CCR9;S1PR1;RAC2;CCR7;CCL17	6.33E-03
chemotaxis (GO:0006935)	20/263	CX3CR1;DOCK4;NRP2;ITGAM;FCER1G;LEF1;PTAFR;PTPRO;PLAUR;CXCL1;PRKCA;TNFRSF11A;L1CAM;CXCL2;SPN;CCR9;S1PR1;RAC2;CCR7;CCL17	6.33E-03
positive regulation of interleukin-2 production (GO:0032743)	6/29	STAT5A;CD4;IRF4;CD28;CD3E;MALT1	7.36E-03
negative regulation of B cell activation (GO:0050869)	6/29	CD300A;FAS;TNFAIP3;PRDM1;SLA2;SAMSN1	7.36E-03
regulation of defense response to virus (GO:0050688)	10/77	SPN;CD4;DDX58;LCK;IL2RA;CD28;TNFAIP3;FYN;TMEM173;DDX60	4.79E-03
positive regulation of response to wounding (GO:1903036)	13/130	PTGER4;STAT5A;FCER1G;PRKCA;TNFRSF11A;PTGS2;IL17RA;FABP4;CD28;TLR7;CCR7;HPSE;IL6ST	6.33E-03
alpha-beta T cell activation (GO:0046631)	7/40	PTGER4;ITK;SATB1;TCF7;LEF1;BCL3;RELB	6.80E-03
platelet activation (GO:0030168)	17/205	P2RY12;CD63;FCER1G;DGKD;PRKCB;PROS1;PRKCA;RASGRP2;RASGRP1;CLU;RAP1A;RAP2B;LCK;PECAM1;RAC2;FYN;LAT	6.61E-03
leukocyte activation involved in immune response (GO:0002366)	12/112	PTGER4;CX3CR1;FCER1G;LGALS1;ANXA3;GPR183;LEF1;BCL3;TLR7;RASGRP1;LAT;RELB	6.28E-03
cell activation involved in immune response (GO:0002263)	12/112	PTGER4;CX3CR1;FCER1G;LGALS1;ANXA3;GPR183;LEF1;BCL3;TLR7;RASGRP1;LAT;RELB	6.28E-03
response to interferon-beta (GO:0035456)	5/18	IFITM3;BST2;IFITM1;IFITM2;TMEM173	6.33E-03
regulation of T cell differentiation (GO:0045580)	11/97	STAT5A;CD2;IL1RL2;TESPA1;IRF4;LCK;HLX;IL2RA;CLPTM1;TNFSF9;IL7R	6.56E-03
