# Supplementary material for: CD8+XCR1neg Dendritic Cells Express High Levels of Toll-Like Receptor 5 and a Unique Complement of Endocytic Receptors
Source: Front Immunol. 2019 Jan 16;9:2990. doi: 10.3389/fimmu.2018.02990 (PMC6343586; doi:10.3389/fimmu.2018.02990)
Supplement: Supplementary Table 6 — Ingenuity Pathway Analysis of differentially expressed genes between CD8+XCR1neg and CD8+XCR1+DC. Pathway and network analysis was carried out using Ingenuity pathway analysis with genes up– or down–regulated with a FC >5 and adj. P < 0.05. [file Table_6.PDF]

Table S6. Ingenuity Pathway Analysis of differentially expressed genes between CD8+XCR1neg and CD8+XCR1+ DC

Pathway and network analysis was carried out using Ingenuity pathway Analysis with genes up- or down-regulated with a FC>5 and adj. P-value<0.05

Ingenuity Pathway Analysis

Term	Overlap	Gene Symbols	Adjusted P-value
Communication between Innate and Adaptive Immune Cells	9/63	Tlr11,TLR4,TLR5,IL12B,CD4,TLR7,TLR3,CD8B,Ccl9	3.63E-05
LXR/RXR Activation	10/108	IL1R2,TLR4,IL1RL2,CD36,SERPINF1,IL1R1,PLTP,PTGS2,TLR3,APOD	2.24E-04
Granulocyte Adhesion and Diapedesis	10/151	IL1R2,SELL,IL1RL2,PECAM1,CCL17,IL1R1,SDC4,MMP12,Cxcl9,Ccl9	2.95E-03
Altered T Cell and B Cell Signaling in Rheumatoid Arthritis	7/110	Tlr11,TLR4,CD79B,TLR5,IL12B,TLR7,TLR3	4.68E-03
phagosome formation	8/109	MRC1,Tlr11,TLR4,PLCE1,TLR5,TLR7,TLR3,PRKCA	5.37E-03
Crosstalk between Dendritic Cells and Natural Killer Cells	6/68	TLR4,CD209,KLRD1,IL12B,TLR7,TLR3	1.23E-02
Colorectal Cancer Metastasis Signaling	11/242	Tlr11,TLR4,CDH1,TCF4,TLR5,ADCY4,TLR7,PTGER2,PTGS2,TLR3,M	1.35E-02
NF-kB Signaling	9/168	IL1R2,TLR4,TLR5,TLR7,IL1R1,TLR3,KDR,IGF2R,DDR1	1.35E-02
Role of Macrophages, Fibroblasts and Endothelial Cells in Rheumatoid Arthritis	12/296	IL1R2,Tlr11,TLR4,TCF4,PLCE1,IL1RL2,TLR5,TLR7,DKK2,IL1R1,TLR3,PRKCA	1.78E-02
Hepatic Cholestasis	8/149	IL1R2,TLR4,CYP7B1,IL1RL2,IL12B,ADCY4,IL1R1,PRKCA	2.24E-02
Agranulocyte Adhesion and Diapedesis	8/180	SELL,PECAM1,CCL17,IL1R1,SDC4,MMP12,Cxcl9,Ccl9	3.16E-02
Role of Pattern Recognition Receptors in Recognition of Bacteria and Viruses	7/127	Tlr11,TLR4,TLR5,IL12B,TLR7,TLR3,PRKCA	3.31E-02
TREM1 Signaling	5/68	Tlr11,TLR4,TLR5,TLR7,TLR3	4.17E-02
Toll-like Receptor Signaling	5/70	TLR4,TLR5,IL12B,TLR7,TLR3	4.37E-02
Natural Killer Cell Signaling	6/105	LAIR1,KLRD1,Klra7,SH2D1B,CD300A,PRKCA	4.79E-02
B Cell Development	3/22	IL7R,CD79B,DNTT	4.79E-02
