# Supplementary material for: CD8+XCR1neg Dendritic Cells Express High Levels of Toll-Like Receptor 5 and a Unique Complement of Endocytic Receptors
Source: Front Immunol. 2019 Jan 16;9:2990. doi: 10.3389/fimmu.2018.02990 (PMC6343586; doi:10.3389/fimmu.2018.02990)
Supplement: Supplementary Table 7 — Differentially expressed genes between CD8+XCR1neg and Splenic CD4+ DCs. Differentially expressed genes between CD8+XCR1neg and Splenic CD4+DC subsets. The data are derived from Top Table analysis with FDR < 0.05. [file Table_7.pdf]

Table S7. Differentially expressed genes between CD8+XCR1neg and Splenic CD4+ DCs

Differentially expressed genes between CD8+XCR1neg and Splenic CD4+ DC subsets
The data are derived from Top Table analysis with FDR<0.05

**GeneID**	**logFC**	**AveExpr**	**t**	**P.Value**	**adj.P.Val**
Cd209d	3.198522	0.874796	28.12495	3.59E-20	7.86E-18
Klk1	2.749011	0.752563	26.40055	1.62E-19	2.67E-17
Cd8a	2.733129	1.568052	36.52425	6.87E-23	4.81E-20
Ccl22	2.655159	2.473544	47.98529	9.35E-26	3.31E-22
Ly6d	2.420511	0.637911	25.80377	2.78E-19	3.97E-17
Trac	2.339829	1.709191	13.8633	4.24E-13	9.92E-12
Il7r	2.280299	1.567903	25.37754	4.12E-19	5.57E-17
Apod	2.263802	0.532687	31.07536	3.32E-21	1.24E-18
Siglech	2.254392	0.999409	27.08363	8.81E-20	1.67E-17
Atp1b1	2.130608	0.868026	31.95647	1.70E-21	7.91E-19
Ly6c2	2.125364	1.78417	18.92221	3.90E-16	1.99E-14
Cd209e	2.104469	0.419276	43.31483	1.12E-24	1.39E-21
Cd8b1	2.096533	0.6483	22.98349	4.25E-18	4.00E-16
Olfr164	2.090969	0.451582	19.56227	1.81E-16	1.08E-14
Cyp7b1	2.048505	0.354743	47.28714	1.33E-25	3.31E-22
Mgl2	1.963777	0.319574	32.39771	1.22E-21	6.51E-19
Mctp2	1.950829	0.395683	30.23584	6.39E-21	1.90E-18
Il1r1	1.935485	0.336355	28.20199	3.37E-20	7.59E-18
Cox6a2	1.927485	0.639606	28.8131	2.02E-20	5.06E-18
Sell	1.907878	1.21452	22.89364	4.66E-18	4.23E-16
Sema6d	1.880407	1.496453	22.11658	1.05E-17	8.95E-16
Mmp25	1.84988	1.3793	29.50833	1.14E-20	3.27E-18
Trbc1	1.833011	1.437146	11.06894	5.06E-11	6.94E-10
Dab2	1.813754	0.362542	27.20083	7.95E-20	1.57E-17
Ccl17	1.77943	1.166254	13.8833	4.11E-13	9.64E-12
Klra17	1.769436	0.402446	24.11139	1.38E-18	1.47E-16
Blnk	1.765704	0.830912	28.40466	2.84E-20	6.82E-18
Trbc2	1.760652	2.021712	14.31388	2.10E-13	5.30E-12
Cd3e	1.751317	0.759463	11.313	3.22E-11	4.63E-10
2610528A1	1.712028	0.557984	16.06731	1.61E-14	5.23E-13
Irf8	1.695343	2.354356	26.37301	1.66E-19	2.68E-17
Traj18	1.654162	0.686315	9.398068	1.33E-09	1.35E-08
Traj43	1.646157	0.647585	13.68452	5.62E-13	1.26E-11
Clec10a	1.636494	0.510665	23.72543	2.01E-18	2.05E-16
Chil5	1.614456	0.67689	29.32299	1.33E-20	3.67E-18
Cldn1	1.60599	1.618839	25.49314	3.70E-19	5.19E-17
Trbj2-1	1.602585	0.873685	7.948475	3.04E-08	2.31E-07
Ptprf	1.575538	0.250477	27.63394	5.46E-20	1.16E-17
Cd3g	1.573507	0.673279	12.00576	9.31E-12	1.55E-10
Aff3	1.571776	1.018974	27.06535	8.95E-20	1.67E-17
Ctsl	1.571617	0.858544	17.49262	2.36E-15	9.08E-14
Sep-03	1.561543	0.97753	31.96301	1.69E-21	7.91E-19
Lgals1	1.546848	2.173595	21.48515	2.06E-17	1.60E-15
Gpr171	1.526689	1.54649	21.77054	1.51E-17	1.24E-15
Gfra2	1.523476	0.764849	20.48052	6.27E-17	4.32E-15
Fscn1	1.520049	2.219622	18.46242	6.86E-16	3.17E-14

Sned1	1.509944	0.599359	21.81501	1.44E-17	1.19E-15
Havcr1	1.495398	0.315994	23.67129	2.13E-18	2.11E-16
Cd3d	1.491915	0.719173	10.34028	2.02E-10	2.45E-09
Bst2	1.481741	1.524591	20.86335	4.08E-17	3.00E-15
Slc41a2	1.469769	0.927206	28.28451	3.14E-20	7.30E-18
Clu	1.468049	1.064797	19.86532	1.27E-16	7.88E-15
Myb	1.460971	1.495033	28.80196	2.04E-20	5.06E-18
Il1rl2	1.448615	0.085021	32.63619	1.03E-21	5.88E-19
Ly6a	1.437136	1.544134	12.41881	4.55E-12	8.08E-11
Klk1b27	1.4325	0.323888	13.69969	5.49E-13	1.24E-11
Ccr9	1.429829	0.696088	20.20937	8.54E-17	5.53E-15
Thy1	1.427594	0.603138	12.98713	1.75E-12	3.49E-11
Lag3	1.421293	0.782252	25.00775	5.82E-19	7.28E-17
Fcrla	1.420086	0.718663	25.82641	2.72E-19	3.97E-17
Nudt17	1.415356	1.48101	21.40889	2.24E-17	1.72E-15
Adcy6	1.407711	1.514748	17.21854	3.37E-15	1.26E-13
Cmah	1.407536	0.363771	17.70374	1.79E-15	7.19E-14
Strip2	1.402314	1.719703	17.66692	1.88E-15	7.44E-14
Ctsw	1.393414	0.657375	15.91993	1.98E-14	6.19E-13
Trbj2-7	1.393108	0.661628	8.826259	4.43E-09	4.09E-08
P2ry14	1.391262	1.044111	21.34383	2.40E-17	1.82E-15
Khdc1a	1.377533	0.391244	16.34824	1.09E-14	3.64E-13
Gm5797	1.375807	1.229001	22.17318	9.85E-18	8.53E-16
Igkc	1.374689	1.161602	4.743903	7.58E-05	0.000287
Sema4a	1.370462	1.773268	30.83122	4.01E-21	1.36E-18
Cacnb3	1.364286	1.417898	19.47077	2.02E-16	1.18E-14
Cd2	1.358952	0.683124	12.74876	2.60E-12	5.00E-11
Traj22	1.350147	0.41101	11.73551	1.50E-11	2.37E-10
F630111L1	1.342217	1.04704	18.10294	1.08E-15	4.66E-14
Cd200	1.340153	1.29972	24.64639	8.21E-19	9.70E-17
Kdr	1.339409	0.244754	30.9901	3.55E-21	1.26E-18
Dntt	1.324513	0.198755	18.53415	6.28E-16	2.94E-14
Tcf4	1.318487	1.33336	27.19164	8.02E-20	1.57E-17
A530099J1	1.317653	1.871805	26.06914	2.18E-19	3.38E-17
Ccr5	1.316298	0.885984	15.95846	1.88E-14	5.94E-13
Cd300c	1.307247	0.893046	14.73355	1.11E-13	3.02E-12
Net1	1.297609	1.841656	26.08132	2.15E-19	3.38E-17
Fnbp1l	1.296834	1.679034	19.33663	2.37E-16	1.34E-14
Pi16	1.294297	0.198548	26.04574	2.23E-19	3.38E-17
Cdkn1a	1.285613	2.381641	22.06634	1.10E-17	9.33E-16
Slamf9	1.276171	0.663882	19.20107	2.79E-16	1.49E-14
Adam11	1.272553	1.300232	19.67044	1.60E-16	9.74E-15
Lynx1	1.271674	0.488005	23.43469	2.69E-18	2.60E-16
Xcl1	1.262765	1.240748	6.238581	1.73E-06	9.30E-06
Traj21	1.259862	0.451524	8.593924	7.31E-09	6.38E-08
Lat	1.252271	0.701249	9.066596	2.66E-09	2.56E-08
Srl	1.251139	0.286694	24.32978	1.11E-18	1.24E-16
Atp2b4	1.243981	0.622457	23.51749	2.48E-18	2.43E-16
Traj12	1.23331	0.535083	7.246145	1.53E-07	1.02E-06
Fmnl2	1.232901	2.196545	31.41015	2.57E-21	1.05E-18
Gria3	1.228222	0.54014	18.35841	7.81E-16	3.52E-14
Rftn1	1.227483	1.586358	24.33121	1.11E-18	1.24E-16

Iglv3	1.226114	0.394045	15.6065	3.09E-14	9.16E-13
Pdlim1	1.218824	0.441103	17.67546	1.86E-15	7.40E-14
Cd200r1	1.211777	0.606101	18.92842	3.87E-16	1.99E-14
Cdh1	1.207863	0.232653	20.71229	4.83E-17	3.46E-15
Timd4	1.206073	0.495417	14.72928	1.12E-13	3.03E-12
Nucb2	1.201086	1.420685	19.53297	1.88E-16	1.11E-14
Anxa3	1.199371	2.203711	15.05998	6.84E-14	1.91E-12
Gzmc	1.192454	0.255597	6.998117	2.75E-07	1.74E-06
Igkj1	1.187183	1.006154	5.012694	3.80E-05	0.000155
Cd33	1.184696	0.704596	13.78723	4.78E-13	1.09E-11
Lifr	1.184314	0.514832	18.67073	5.31E-16	2.61E-14
Ptgr1	1.182293	0.307091	16.51019	8.73E-15	2.97E-13
Arhgap31	1.181027	1.808449	19.29135	2.50E-16	1.39E-14
Zmynd15	1.178003	1.575491	17.27631	3.13E-15	1.19E-13
Snrnp25	1.163544	1.630097	16.67287	6.99E-15	2.43E-13
Retnla	1.162879	0.266113	9.811249	5.75E-10	6.30E-09
Efnb2	1.155581	0.405317	15.61424	3.06E-14	9.16E-13
Snx18	1.152078	1.049052	23.88306	1.72E-18	1.78E-16
Crip1	1.149853	3.410685	26.9096	1.03E-19	1.82E-17
Iglv2	1.148616	0.169992	8.63853	6.64E-09	5.88E-08
Cd24a	1.144088	2.049596	13.42411	8.54E-13	1.82E-11
Smim5	1.13718	0.319038	19.26706	2.57E-16	1.41E-14
Ahnak	1.134405	1.584713	19.12177	3.07E-16	1.62E-14
Il9r	1.128325	0.49637	18.72734	4.95E-16	2.49E-14
Tex2	1.126376	0.866691	18.57305	5.99E-16	2.82E-14
Pacsin1	1.120062	0.26195	17.74971	1.69E-15	6.87E-14
Cxcl16	1.115813	2.65295	17.8517	1.48E-15	6.09E-14
Met	1.11493	1.044006	16.11616	1.50E-14	4.91E-13
Trbj1-1	1.112207	0.440487	9.963948	4.24E-10	4.85E-09
Gfpt2	1.11117	0.687002	16.02063	1.72E-14	5.51E-13
Nkg7	1.111011	1.058224	9.608385	8.67E-10	9.17E-09
Fabp5	1.107847	0.655705	14.407	1.82E-13	4.76E-12
Il2ra	1.104362	0.507861	15.29176	4.87E-14	1.41E-12
Nrp2	1.104281	0.612003	20.21648	8.47E-17	5.53E-15
Cnn3	1.101381	0.585994	12.80349	2.37E-12	4.64E-11
Hs3st1	1.100818	0.185596	13.39122	9.01E-13	1.91E-11
Mtss1	1.099203	0.666418	20.23291	8.31E-17	5.52E-15
Podn	1.098451	0.163317	20.21833	8.45E-17	5.53E-15
Ighj4	1.091908	0.569565	5.105031	3.00E-05	0.000124
Rxra	1.085773	0.707588	23.03396	4.04E-18	3.85E-16
Traj23	1.084928	0.455007	11.62087	1.84E-11	2.83E-10
Igkj5	1.08246	0.702769	6.230841	1.76E-06	9.46E-06
Ctnna1	1.076633	2.294226	29.04292	1.67E-20	4.44E-18
Gimap6	1.068442	0.599445	11.50721	2.26E-11	3.38E-10
Cd28	1.053979	0.505585	8.377571	1.17E-08	9.67E-08
Ceacam15	1.052608	0.065143	11.11655	4.63E-11	6.40E-10
Zc3h12c	1.046813	2.106517	18.79688	4.55E-16	2.30E-14
Lef1	1.042832	0.352886	8.439489	1.02E-08	8.57E-08
Lck	1.042708	0.454402	10.0031	3.92E-10	4.54E-09
Cttnbp2nl	1.042086	1.543481	19.2029	2.78E-16	1.49E-14
Gm12253	1.041877	0.059172	13.59658	6.47E-13	1.43E-11
Fas	1.039399	1.080223	12.70365	2.80E-12	5.36E-11

Hpse	1.036227	1.236566	20.69034	4.95E-17	3.47E-15
Sdc4	1.035225	1.097116	15.28601	4.91E-14	1.42E-12
Gm3696	1.032872	0.81788	12.18082	6.86E-12	1.17E-10
Serpinb6b	1.031309	2.039391	15.17482	5.78E-14	1.63E-12
Cd96	1.0291	0.431412	12.019	9.09E-12	1.52E-10
Prkca	1.025658	0.18822	19.6312	1.67E-16	1.01E-14
Poglut1	1.016709	2.298376	19.91189	1.20E-16	7.53E-15
Xkrx	1.007836	0.264441	20.31912	7.53E-17	5.05E-15
Map4k4	1.007065	1.950419	23.70048	2.07E-18	2.08E-16
Ak8	1.003954	0.190068	20.71167	4.83E-17	3.46E-15
Ighj3	0.997798	0.121295	5.640599	7.70E-06	3.62E-05
Cd209c	0.994009	0.280782	17.22405	3.35E-15	1.25E-13
Ifi205	0.988365	1.81775	8.056431	2.39E-08	1.86E-07
Trib1	0.988114	1.366393	14.05123	3.16E-13	7.63E-12
Spib	0.986735	0.870251	19.02406	3.45E-16	1.78E-14
Gatsl2	0.986426	1.020553	16.01118	1.74E-14	5.56E-13
B930041F1	0.98532	0.320575	20.33118	7.43E-17	5.03E-15
Fcgrt	0.980777	1.614566	15.62141	3.03E-14	9.15E-13
Ighj1	0.979194	1.479854	7.513851	8.21E-08	5.77E-07
Trbj2-3	0.973132	0.452293	8.079051	2.27E-08	1.78E-07
Snord35b	0.972318	0.695417	7.455227	9.40E-08	6.50E-07
Sla2	0.972084	0.417409	12.07578	8.23E-12	1.40E-10
Idh1	0.971024	1.752955	18.09801	1.08E-15	4.66E-14
Runx2os2	0.966104	0.644515	11.61135	1.88E-11	2.87E-10
Dirc2	0.964482	0.828744	19.11032	3.11E-16	1.63E-14
Micu1	0.960743	1.261629	17.85736	1.47E-15	6.08E-14
Prkcq	0.955542	0.406738	8.323211	1.32E-08	1.08E-07
Tmem123	0.951692	3.011727	19.60944	1.71E-16	1.03E-14
Olfm1	0.949741	0.973521	24.32535	1.12E-18	1.24E-16
Cd226	0.947527	0.596043	12.39534	4.73E-12	8.37E-11
BC021614	0.945623	0.599744	12.57146	3.50E-12	6.46E-11
Itk	0.944768	0.310684	8.987391	3.15E-09	2.99E-08
Gm1965	0.944402	0.980992	12.73799	2.65E-12	5.07E-11
Trf	0.943942	1.514319	13.12726	1.39E-12	2.84E-11
Fcgr2b	0.941736	0.985726	14.53625	1.50E-13	3.95E-12
P2ry12	0.940003	0.49092	13.11838	1.41E-12	2.87E-11
Pla2g4f	0.934936	0.482836	13.60473	6.39E-13	1.41E-11
Traj19	0.929886	0.15458	8.359289	1.22E-08	1.00E-07
Igkj3	0.925171	0.015723	9.946698	4.39E-10	4.98E-09
Mcpt4	0.923296	0.474176	4.086481	0.000409	0.001315
LOC10263:	0.916682	0.369061	4.784682	6.83E-05	0.000262
Nipal1	0.915968	0.690979	16.43362	9.69E-15	3.28E-13
Gimap4	0.915479	0.663939	8.50803	8.81E-09	7.54E-08
Tnfrsf4	0.914124	1.474343	15.60798	3.08E-14	9.16E-13
Traj38	0.913508	0.183794	6.929513	3.24E-07	2.02E-06
Fads2	0.912253	0.46546	12.03191	8.89E-12	1.50E-10
Glipr2	0.911078	1.040159	17.33674	2.89E-15	1.10E-13
2010016I1	0.910216	0.591546	13.888	4.08E-13	9.60E-12
Tubb2b	0.910049	1.016829	12.25597	6.02E-12	1.03E-10
Gnb4	0.909268	1.582267	15.08944	6.55E-14	1.83E-12
Rabgap1l	0.909141	1.584077	17.70194	1.80E-15	7.19E-14
L1cam	0.905102	0.6415	16.27076	1.21E-14	4.03E-13

Cd200r4	0.904257	0.375087	12.33287	5.27E-12	9.23E-11
Tnip3	0.903398	0.476385	10.43927	1.67E-10	2.05E-09
Serinc5	0.901123	0.922867	16.93207	4.93E-15	1.78E-13
Prnp	0.896883	1.017862	9.396273	1.34E-09	1.36E-08
Mansc1	0.896282	0.665997	18.60831	5.73E-16	2.73E-14
Ptgir	0.894634	0.203218	17.60042	2.05E-15	8.06E-14
Stap2	0.893557	1.393968	13.53628	7.13E-13	1.56E-11
Pnkd	0.891628	0.518113	9.512069	1.06E-09	1.11E-08
S1pr1	0.888183	0.48723	9.093865	2.51E-09	2.43E-08
Slc22a23	0.88701	1.148808	13.89943	4.00E-13	9.49E-12
Traj37	0.885493	0.108602	8.442853	1.02E-08	8.52E-08
Slco4a1	0.87957	0.122766	14.01084	3.36E-13	8.07E-12
Eno2	0.879309	0.873224	11.53218	2.16E-11	3.25E-10
Hsd11b1	0.878607	0.472036	12.92872	1.92E-12	3.80E-11
Mfge8	0.877221	1.425164	13.79074	4.75E-13	1.09E-11
Ccr2	0.877187	1.689933	11.01253	5.62E-11	7.63E-10
Zfp872	0.876031	0.948556	14.5529	1.46E-13	3.87E-12
Padi2	0.87574	0.622603	17.19373	3.48E-15	1.29E-13
Maged1	0.875558	0.513843	14.92179	8.39E-14	2.31E-12
Tnfrsf9	0.875531	0.83036	15.13206	6.15E-14	1.73E-12
Frmd4a	0.871424	1.351713	20.43357	6.61E-17	4.51E-15
Akap2	0.870919	1.204459	11.55143	2.09E-11	3.15E-10
Il13ra1	0.870119	1.913722	13.15165	1.33E-12	2.74E-11
Dapl1	0.869446	0.356956	7.191069	1.74E-07	1.14E-06
Sec24d	0.869052	1.046202	17.95237	1.30E-15	5.48E-14
Il2rb	0.864188	0.703821	8.493952	9.08E-09	7.73E-08
F2r	0.863716	0.420394	11.20071	3.96E-11	5.51E-10
Plxdc1	0.862073	1.375905	18.68794	5.20E-16	2.59E-14
Gimap3	0.862029	0.422758	8.508631	8.80E-09	7.53E-08
Vim	0.856592	3.424248	17.2478	3.24E-15	1.23E-13
Tiam1	0.854517	0.976071	16.04535	1.66E-14	5.35E-13
Bcl2l14	0.852599	0.965507	15.39251	4.21E-14	1.22E-12
Traj17	0.847857	0.21979	6.526097	8.56E-07	4.88E-06
Traj30	0.847199	0.159162	10.68975	1.03E-10	1.32E-09
Trbj2-6	0.846121	0.111463	8.944282	3.45E-09	3.24E-08
Gng12	0.844768	0.817675	12.93971	1.89E-12	3.74E-11
Pfkfb3	0.843549	2.165306	15.94272	1.92E-14	6.05E-13
Ly75	0.84236	1.734175	11.73301	1.51E-11	2.37E-10
Traj27	0.841767	0.202083	5.829672	4.79E-06	2.35E-05
Pdzd4	0.83962	0.326358	13.96154	3.63E-13	8.64E-12
Il18r1	0.839292	0.529405	9.396379	1.34E-09	1.36E-08
Cp	0.838951	0.171494	17.35156	2.83E-15	1.09E-13
Pros1	0.834345	0.174349	16.95454	4.79E-15	1.74E-13
Chchd10	0.829099	0.472253	11.25768	3.57E-11	5.04E-10
Klf12	0.828393	0.128136	15.28197	4.94E-14	1.42E-12
Ets2	0.82674	0.426491	12.64802	3.08E-12	5.74E-11
Sfxn3	0.825193	0.896499	15.42027	4.04E-14	1.18E-12
Gm4759	0.822963	0.391172	9.647652	8.01E-10	8.50E-09
Traj31	0.819355	0.340067	7.019314	2.61E-07	1.66E-06
LOC10263	0.81781	0.712671	11.53777	2.14E-11	3.23E-10
Lad1	0.816017	1.120644	11.47897	2.38E-11	3.55E-10
Cd63	0.813606	1.840561	11.38637	2.82E-11	4.11E-10

Syne2	0.813427	0.684116	18.58865	5.87E-16	2.78E-14
Timp2	0.81229	0.3904	11.45006	2.51E-11	3.71E-10
Serpinb1a	0.810122	1.439544	14.37766	1.91E-13	4.94E-12
Ccr7	0.809234	2.162214	10.34173	2.02E-10	2.44E-09
Enpp5	0.807531	0.484069	11.8481	1.23E-11	1.99E-10
Arsb	0.801319	1.779664	22.29888	8.63E-18	7.56E-16
Cpne2	0.800202	2.395112	14.92554	8.34E-14	2.31E-12
Ndnf	0.800126	0.634088	10.57722	1.28E-10	1.60E-09
Ly6i	0.799404	0.599591	7.960892	2.96E-08	2.25E-07
Atxn1	0.798644	1.100736	14.2738	2.24E-13	5.62E-12
Gbp5	0.7968	1.538966	10.49135	1.51E-10	1.88E-09
Traj11	0.796087	0.164119	7.418533	1.02E-07	7.03E-07
Ctnnd1	0.7956	0.911683	15.89109	2.06E-14	6.42E-13
Mgst2	0.794702	0.523287	9.425367	1.26E-09	1.29E-08
Sidt1	0.7947	0.256861	9.157355	2.20E-09	2.14E-08
Serpinb9	0.793966	1.556679	10.98766	5.88E-11	7.96E-10
Calm4	0.793046	0.904428	11.78285	1.38E-11	2.21E-10
Traj9	0.79231	0.310137	5.756061	5.76E-06	2.78E-05
Trbj1-2	0.79101	0.491907	6.497718	9.17E-07	5.19E-06
Traj34	0.789389	0.082723	7.338064	1.23E-07	8.36E-07
Tmem39a	0.788525	2.292383	15.6116	3.07E-14	9.16E-13
Cdc14b	0.783796	0.520727	13.30204	1.04E-12	2.18E-11
Tcf7	0.781837	1.433591	7.525443	7.99E-08	5.63E-07
Fyn	0.78129	1.451634	17.80211	1.58E-15	6.46E-14
Pvr	0.777353	1.475216	17.17877	3.55E-15	1.31E-13
Sema4b	0.777188	0.474901	12.51991	3.83E-12	6.94E-11
Rasgrp2	0.776458	0.325067	13.06166	1.54E-12	3.11E-11
Cst7	0.775202	0.585761	10.92963	6.56E-11	8.73E-10
Cd6	0.774929	0.262627	11.75004	1.46E-11	2.33E-10
Mt2	0.774224	1.001232	8.690115	5.94E-09	5.34E-08
Synpo2	0.770028	0.676142	14.66249	1.24E-13	3.32E-12
Snn	0.767836	1.064401	13.09912	1.45E-12	2.95E-11
Dap	0.766054	2.267707	16.85757	5.45E-15	1.95E-13
Ptprcap	0.765144	1.240863	8.62973	6.76E-09	5.98E-08
Themis	0.76339	0.22107	6.740688	5.09E-07	3.04E-06
Itga6	0.763363	0.643009	10.61216	1.20E-10	1.51E-09
Ttc3	0.762277	1.52772	18.17435	9.84E-16	4.28E-14
Mrc1	0.762121	0.204576	11.74181	1.48E-11	2.35E-10
Samsn1	0.761728	2.328377	13.82019	4.54E-13	1.05E-11
LOC10263	0.760309	0.800876	13.67265	5.73E-13	1.28E-11
Pnpla8	0.758888	1.339821	12.57775	3.47E-12	6.40E-11
Spata31d1	0.757746	0.620413	8.978997	3.20E-09	3.03E-08
Ephx1	0.757554	0.543151	12.26399	5.93E-12	1.02E-10
Traj15	0.757407	0.205698	6.556946	7.94E-07	4.57E-06
Myzap	0.755667	0.451947	17.99196	1.24E-15	5.24E-14
Ugcg	0.752782	1.067744	10.82209	8.03E-11	1.05E-09
Tagln2	0.751005	2.899857	14.9814	7.68E-14	2.13E-12
Cxcl1	0.74891	0.363808	9.854289	5.28E-10	5.84E-09
Trbv13-2	0.748381	0.162474	7.064278	2.35E-07	1.50E-06
Cacnb2	0.747515	0.385827	13.62003	6.23E-13	1.38E-11
Mir99a	0.746383	0.042656	10.81124	8.20E-11	1.07E-09
Traj40	0.745139	0.062689	5.624543	8.02E-06	3.75E-05

Lima1	0.740585	1.182455	12.68946	2.87E-12	5.42E-11
Traj20	0.739647	0.174957	6.662657	6.15E-07	3.62E-06
Jup	0.737433	1.049732	13.16344	1.31E-12	2.69E-11
Gm3591	0.735217	0.589852	6.473707	9.72E-07	5.47E-06
Cd14	0.732366	0.437253	10.31339	2.13E-10	2.56E-09
Clcn5	0.731953	0.53515	13.47096	7.92E-13	1.71E-11
Insl6	0.731312	1.097504	11.30518	3.27E-11	4.68E-10
Zdhhc15	0.730939	0.462848	12.97566	1.78E-12	3.54E-11
Ldlrad3	0.730671	0.404065	12.66714	2.98E-12	5.60E-11
Ankrd33b	0.730109	0.71096	14.34788	2.00E-13	5.12E-12
Tspan3	0.728138	1.979375	11.90174	1.12E-11	1.83E-10
Naaa	0.727432	1.950058	8.484491	9.27E-09	7.86E-08
Dennd2d	0.726518	0.622161	14.10813	2.89E-13	7.12E-12
Gyg	0.726464	2.430016	11.8635	1.20E-11	1.94E-10
Tspo	0.726192	1.891373	11.41517	2.67E-11	3.92E-10
Trim3	0.725972	0.975166	13.5104	7.43E-13	1.62E-11
Mpeg1	0.721696	2.104636	11.24119	3.68E-11	5.16E-10
P2ry13	0.721345	0.415055	11.91525	1.09E-11	1.79E-10
Sox4	0.719997	0.61224	13.38769	9.06E-13	1.92E-11
Fcor	0.717381	0.771353	8.979261	3.20E-09	3.03E-08
LOC10263!	0.717188	3.113007	15.12276	6.23E-14	1.75E-12
Cxxc5	0.717076	0.443721	10.92855	6.57E-11	8.74E-10
Ybx3	0.71574	1.868722	14.36053	1.96E-13	5.06E-12
L3mbtl3	0.715525	0.604418	11.55825	2.06E-11	3.12E-10
Tmbim1	0.714598	0.950144	10.33173	2.06E-10	2.48E-09
Gne	0.713958	0.785099	11.70582	1.58E-11	2.47E-10
Zhx2	0.713882	1.034835	10.49046	1.51E-10	1.88E-09
Slc15a3	0.713748	0.97895	15.40227	4.15E-14	1.21E-12
Fam129b	0.711155	0.377832	18.66487	5.35E-16	2.61E-14
Galc	0.70913	0.97262	12.55438	3.61E-12	6.61E-11
Traj7	0.708377	0.217304	10.29278	2.22E-10	2.65E-09
Fam3c	0.707688	0.884344	14.1064	2.90E-13	7.12E-12
Il15ra	0.703553	1.665851	10.04699	3.59E-10	4.20E-09
Cx3cr1	0.702705	0.980518	11.42329	2.64E-11	3.88E-10
Apol7c	0.702205	2.514913	7.76907	4.57E-08	3.38E-07
Papss2	0.701864	1.267174	9.433074	1.24E-09	1.28E-08
Slc33a1	0.701295	1.71402	13.98755	3.49E-13	8.34E-12
Trbj1-3	0.700798	0.365209	6.96012	3.01E-07	1.89E-06
Csf2rb2	0.698869	1.901855	11.73423	1.51E-11	2.37E-10
Glycam1	0.695207	0.371191	3.540102	0.001631	0.004498
Trp53i11	0.693522	0.53574	8.690652	5.93E-09	5.34E-08
Ptprs	0.69169	0.854611	14.33724	2.03E-13	5.17E-12
Clec9a	0.690101	1.815695	8.672986	6.16E-09	5.50E-08
Calcoco1	0.688543	0.710509	10.49548	1.50E-10	1.87E-09
Vhl	0.688191	1.343551	11.69526	1.61E-11	2.50E-10
Gm19705	0.687039	0.271227	11.01861	5.55E-11	7.56E-10
Mmp23	0.685247	0.849893	13.32693	9.99E-13	2.10E-11
Bach2	0.683696	0.334588	12.66498	2.99E-12	5.61E-11
Adora2a	0.683295	1.253091	13.69642	5.52E-13	1.24E-11
Plin2	0.680194	1.51704	11.74728	1.47E-11	2.34E-10
2610034B:	0.678748	1.165646	9.946214	4.39E-10	4.98E-09
Gaa	0.678709	1.044178	12.67507	2.94E-12	5.54E-11

Igkv1-110	0.677502	0.214667	2.635483	0.014351	0.030624
Pdia5	0.677179	1.019443	12.36008	5.03E-12	8.87E-11
Slco5a1	0.673122	1.304118	10.45958	1.60E-10	1.98E-09
Aire	0.672467	0.482065	8.396968	1.12E-08	9.34E-08
Ccdc109b	0.671558	0.701134	13.55141	6.96E-13	1.52E-11
Tubgcp5	0.670628	1.044092	10.59469	1.24E-10	1.56E-09
Kdm1b	0.665189	1.275833	15.23378	5.30E-14	1.51E-12
Scin	0.664049	0.472045	10.85302	7.57E-11	9.94E-10
Mtmr4	0.66199	1.934737	17.50638	2.31E-15	8.97E-14
Adm	0.661518	0.704513	11.69913	1.60E-11	2.49E-10
Ccser2	0.661066	2.225995	14.34225	2.01E-13	5.15E-12
Ets1	0.659875	0.556175	9.971986	4.17E-10	4.79E-09
Traj49	0.659306	0.114384	5.793694	5.24E-06	2.54E-05
Cmtm3	0.658897	1.71922	11.94411	1.04E-11	1.72E-10
Ms4a4a	0.655862	0.266435	9.78624	6.05E-10	6.58E-09
Slc2a6	0.655546	1.187186	10.98079	5.96E-11	8.05E-10
Wfdc17	0.654541	1.248477	7.229917	1.59E-07	1.05E-06
Rell1	0.654402	1.057163	11.12838	4.53E-11	6.28E-10
Med10	0.653926	1.954705	13.8436	4.37E-13	1.02E-11
Igkv4-91	0.652807	0.207555	3.039	0.005573	0.013515
Ptpn7	0.648593	0.518692	10.65178	1.11E-10	1.41E-09
Aplp2	0.648543	1.866338	12.77886	2.47E-12	4.79E-11
Cd27	0.647536	0.559992	7.699199	5.36E-08	3.91E-07
Scpep1	0.645873	2.418244	13.55367	6.93E-13	1.52E-11
Dapk1	0.645795	0.833596	12.78169	2.46E-12	4.79E-11
Trav13-1	0.64215	0.388264	7.287298	1.39E-07	9.33E-07
Tnfaip3	0.640368	2.189949	14.61817	1.32E-13	3.52E-12
Tlr12	0.638398	0.935424	10.09102	3.29E-10	3.88E-09
Slamf1	0.637883	0.756894	9.26053	1.77E-09	1.76E-08
Pml	0.637103	1.292573	10.93931	6.44E-11	8.59E-10
Pcgf5	0.635122	1.578814	9.923954	4.59E-10	5.17E-09
Ccng2	0.632745	1.984215	12.77847	2.47E-12	4.79E-11
Bmp2k	0.632653	2.739591	14.089	2.98E-13	7.26E-12
Ift57	0.630718	1.647371	12.25979	5.98E-12	1.03E-10
Gramd4	0.629598	0.942573	14.09248	2.96E-13	7.25E-12
Zfp658	0.628304	0.14636	12.1463	7.28E-12	1.24E-10
Uap1	0.627523	2.022025	12.26792	5.89E-12	1.02E-10
Dkk2	0.626339	0.168272	10.64556	1.12E-10	1.43E-09
Ccl19	0.625308	0.963644	6.758906	4.87E-07	2.92E-06
Lyz1	0.624499	1.216249	4.267103	0.000258	0.00087
Gpr68	0.624117	1.597996	11.26328	3.53E-11	5.01E-10
Ffar4	0.623328	1.405195	12.47334	4.14E-12	7.43E-11
Dync1li2	0.619737	1.139433	11.45293	2.50E-11	3.70E-10
H2-Q7	0.618903	2.158113	10.64025	1.13E-10	1.44E-09
Tm4sf5	0.618835	1.026086	9.46636	1.16E-09	1.20E-08
Izumo4	0.618389	1.091161	11.74597	1.47E-11	2.34E-10
Atp11a	0.617668	1.516976	13.76408	4.96E-13	1.13E-11
Tmed3	0.617333	1.106421	11.68809	1.63E-11	2.53E-10
Nuak1	0.615802	0.745084	10.74816	9.24E-11	1.19E-09
Ltc4s	0.613655	0.67989	8.293627	1.41E-08	1.15E-07
Gm11974	0.612991	1.416174	10.31697	2.12E-10	2.54E-09
Dennd4c	0.61188	1.053955	11.26464	3.52E-11	5.00E-10

Golm1	0.611652	0.578288	9.943046	4.42E-10	5.01E-09
Pdgfa	0.611474	0.816897	16.2203	1.30E-14	4.30E-13
Rnf115	0.607724	2.460229	12.35087	5.11E-12	8.99E-11
Sult1a1	0.606348	0.929848	10.45093	1.63E-10	2.01E-09
Plk2	0.605742	1.026096	12.51729	3.84E-12	6.96E-11
F2rl2	0.602272	0.741202	9.225236	1.91E-09	1.88E-08
Dtx2	0.601902	0.867016	13.0955	1.46E-12	2.96E-11
Spr	0.599033	1.417468	7.828746	3.99E-08	2.98E-07
Adpgk	0.598905	0.757214	11.05274	5.21E-11	7.14E-10
Ppp3cc	0.598019	0.715684	9.437304	1.23E-09	1.27E-08
Vkorc1l1	0.595878	1.70884	8.948898	3.41E-09	3.22E-08
Rnf19a	0.594696	1.551739	9.803529	5.84E-10	6.37E-09
Cxcl2	0.593568	0.533283	4.714308	8.18E-05	0.000308
Oxct1	0.593426	1.607583	13.43647	8.37E-13	1.80E-11
Wls	0.592706	0.313007	8.474375	9.48E-09	8.00E-08
Nek6	0.592494	1.202303	13.82432	4.51E-13	1.05E-11
Dgka	0.59236	1.183805	7.756638	4.70E-08	3.47E-07
Slc52a2	0.591957	0.814819	14.4473	1.71E-13	4.49E-12
Gadd45b	0.590953	2.360934	10.12211	3.10E-10	3.66E-09
Slc25a10	0.589684	1.028518	7.610799	6.56E-08	4.70E-07
Mir1927	0.589537	0.82441	9.058755	2.71E-09	2.60E-08
St3gal1	0.589386	1.98035	15.75602	2.50E-14	7.71E-13
Slc4a8	0.586989	1.266284	8.963915	3.31E-09	3.12E-08
Foxp4	0.586126	1.583935	10.06623	3.46E-10	4.06E-09
Arl5c	0.585592	1.542618	9.40702	1.31E-09	1.34E-08
Tubb3	0.585298	0.488833	6.701879	5.59E-07	3.31E-06
Dkkl1	0.584018	0.453878	10.97562	6.02E-11	8.11E-10
Smim24	0.581053	1.043641	12.83277	2.26E-12	4.43E-11
Fnip2	0.580468	1.326724	10.95657	6.24E-11	8.35E-10
Stat5a	0.580462	1.065727	11.71096	1.57E-11	2.45E-10
Runx2	0.580375	1.132784	12.01881	9.10E-12	1.52E-10
Mex3b	0.580182	0.871668	11.72013	1.54E-11	2.42E-10
LOC10263{	0.580101	0.890156	7.942687	3.08E-08	2.34E-07
Pafah1b3	0.57955	1.653113	7.850291	3.80E-08	2.85E-07
Casp6	0.578959	1.033491	9.572584	9.33E-10	9.81E-09
Gpr52	0.578587	0.740479	7.401575	1.06E-07	7.28E-07
Slc22a15	0.573169	0.686509	9.413326	1.29E-09	1.32E-08
AW549877	0.572998	1.627404	14.75952	1.07E-13	2.91E-12
LOC10264(	0.572175	0.581111	6.314709	1.44E-06	7.84E-06
Ly96	0.571992	1.365903	12.62877	3.18E-12	5.92E-11
Naga	0.570822	2.229451	9.47636	1.14E-09	1.18E-08
Gm20257	0.570143	0.647343	6.618071	6.85E-07	4.00E-06
Tmem163	0.569188	0.322632	11.29348	3.34E-11	4.76E-10
Necap2	0.568183	1.844917	10.21461	2.58E-10	3.09E-09
Cpa3	0.565831	0.42665	3.953119	0.000575	0.001788
Vprbp	0.564406	0.74512	11.35817	2.97E-11	4.29E-10
Thap2	0.563492	1.260976	6.686402	5.80E-07	3.43E-06
Cysltr1	0.563258	0.442909	8.93513	3.52E-09	3.30E-08
Man2a2	0.561096	0.625205	8.68252	6.03E-09	5.41E-08
Gm16525	0.560643	0.342391	3.845995	0.000755	0.00229
Cstb	0.559456	2.037392	11.82822	1.27E-11	2.06E-10
Cox6b2	0.558138	0.699763	8.182678	1.80E-08	1.44E-07

Pgls	0.557145	2.012679	11.51733	2.22E-11	3.33E-10
Kctd12	0.556556	2.545869	6.723195	5.31E-07	3.16E-06
Btla	0.55633	1.534526	11.52616	2.19E-11	3.28E-10
Mrps6	0.555414	1.694079	8.839604	4.31E-09	3.98E-08
Man1c1	0.555087	0.379258	11.79764	1.34E-11	2.16E-10
Sigmar1	0.554747	0.946188	11.69985	1.60E-11	2.49E-10
Itgae	0.554644	0.555616	9.583594	9.12E-10	9.62E-09
Dpp7	0.554456	0.898316	9.165285	2.16E-09	2.11E-08
Gm15128	0.552895	0.498802	3.143139	0.004335	0.010791
Lamp1	0.55238	2.359375	11.5847	1.97E-11	3.00E-10
Ak2	0.552349	1.710511	10.77138	8.84E-11	1.15E-09
Gca	0.551931	1.604885	8.119603	2.07E-08	1.63E-07
Slc25a24	0.548155	0.60553	12.27026	5.87E-12	1.02E-10
Nceh1	0.547418	0.649232	13.16802	1.30E-12	2.68E-11
Casp3	0.547092	1.511185	10.7324	9.52E-11	1.23E-09
Esyt1	0.54663	1.559452	13.4374	8.36E-13	1.80E-11
Rab33b	0.545293	0.817866	10.70213	1.01E-10	1.29E-09
Mvb12a	0.544382	1.477688	8.775352	4.94E-09	4.51E-08
Sbf2	0.544166	1.403187	13.05276	1.57E-12	3.14E-11
Vsig10	0.544064	0.775353	9.905441	4.76E-10	5.34E-09
Laptm4b	0.543537	1.459972	9.438297	1.23E-09	1.27E-08
Crispld2	0.543519	0.497121	9.992922	4.00E-10	4.62E-09
Pdlim4	0.542944	1.120536	9.494834	1.09E-09	1.14E-08
Eya1	0.542712	0.40008	9.884472	4.97E-10	5.53E-09
Phxr4	0.542623	0.993265	7.904121	3.36E-08	2.54E-07
Dse	0.542622	1.012384	9.828275	5.56E-10	6.13E-09
Gm16524	0.541711	0.953109	6.232046	1.76E-06	9.45E-06
Idi2	0.541514	0.565627	8.679267	6.08E-09	5.44E-08
Grap2	0.54028	1.067656	8.087202	2.23E-08	1.75E-07
Lrrtm2	0.539938	0.702249	10.44249	1.66E-10	2.04E-09
Ccdc88c	0.538404	0.747232	8.527737	8.44E-09	7.24E-08
Bst1	0.538154	0.95359	9.518679	1.04E-09	1.09E-08
Cxcr3	0.536947	0.932704	12.53437	3.73E-12	6.83E-11
Npr2	0.535651	0.315165	9.961788	4.26E-10	4.87E-09
Txk	0.534541	0.322974	7.032631	2.53E-07	1.61E-06
Cln5	0.533088	1.458007	9.236324	1.87E-09	1.84E-08
Snap29	0.53212	1.037126	9.65458	7.89E-10	8.39E-09
Olfr109	0.530696	0.790913	7.516209	8.16E-08	5.74E-07
Nlrc5	0.528716	1.63263	9.887743	4.93E-10	5.51E-09
H2-M2	0.528203	2.232969	7.970886	2.89E-08	2.21E-07
Tcf12	0.5279	1.06472	10.5862	1.26E-10	1.58E-09
Mllt3	0.527774	0.788695	13.73416	5.20E-13	1.18E-11
Iqgap2	0.527617	1.315067	7.492555	8.62E-08	6.02E-07
Gcnt4	0.527403	0.63133	11.75055	1.46E-11	2.33E-10
Tnnt2	0.527295	0.72781	7.017233	2.63E-07	1.66E-06
Bcr	0.526793	0.449792	8.012175	2.64E-08	2.04E-07
Aim1	0.525779	1.830856	14.32742	2.06E-13	5.23E-12
Ptger4	0.525216	1.929019	9.823816	5.61E-10	6.18E-09
Cblb	0.524642	2.529368	8.440247	1.02E-08	8.56E-08
Nfil3	0.524196	1.000035	8.780712	4.89E-09	4.46E-08
Stoml1	0.523533	0.897744	11.99849	9.43E-12	1.57E-10
Paqr3	0.522777	0.822545	10.45922	1.61E-10	1.98E-09

Gramd1b	0.522717	1.316558	11.40732	2.71E-11	3.96E-10
Slco3a1	0.521844	0.635701	9.81611	5.70E-10	6.24E-09
Ptpn4	0.521715	1.101778	10.71935	9.75E-11	1.25E-09
Tmcc3	0.520399	0.699388	8.873068	4.01E-09	3.73E-08
Dscaml1	0.520298	0.589032	11.60036	1.91E-11	2.92E-10
Efna2	0.519406	0.586996	7.418703	1.02E-07	7.03E-07
Grn	0.516029	2.646348	9.403298	1.32E-09	1.34E-08
Slc25a35	0.515482	0.998221	8.487336	9.21E-09	7.82E-08
Selm	0.515196	0.984867	6.210657	1.85E-06	9.91E-06
Itgb1	0.514738	2.630394	11.7292	1.52E-11	2.38E-10
Cadm1	0.514071	1.36871	8.010216	2.65E-08	2.05E-07
H2-T22	0.513876	1.965657	5.959397	3.46E-06	1.75E-05
Polr3c	0.512759	1.86713	11.00746	5.67E-11	7.69E-10
Abhd17b	0.51062	0.932062	10.89475	7.00E-11	9.24E-10
Gm13179	0.508322	0.927967	6.080168	2.56E-06	1.32E-05
Lxn	0.50809	0.902985	7.912612	3.30E-08	2.49E-07
Klra13-ps	0.507858	0.529873	3.78364	0.000884	0.002625
Gbp2	0.507731	1.058612	6.18568	1.97E-06	1.05E-05
Cyth1	0.506271	1.339102	9.881217	5.00E-10	5.56E-09
Rexo2	0.505642	1.898359	10.03954	3.65E-10	4.26E-09
Vps39	0.504796	1.794925	12.33654	5.24E-12	9.19E-11
Cdh2	0.504265	0.633672	8.144272	1.96E-08	1.56E-07
Pak4	0.503432	0.70072	8.264328	1.50E-08	1.22E-07
Csad	0.503339	0.89538	7.374462	1.13E-07	7.72E-07
Npc2	0.502139	2.695819	14.11337	2.87E-13	7.09E-12
Abcg1	0.501663	1.929433	9.293906	1.66E-09	1.66E-08
Txndc5	0.49825	0.986781	7.697347	5.38E-08	3.92E-07
Prkx	0.498036	1.593659	11.36275	2.94E-11	4.26E-10
Ube2l6	0.497749	1.402095	8.719409	5.57E-09	5.04E-08
S100a1	0.497726	1.030435	7.579987	7.05E-08	5.02E-07
Sepsecs	0.497562	1.444548	9.221671	1.92E-09	1.89E-08
Mylip	0.497207	1.429808	9.393782	1.35E-09	1.36E-08
Eepd1	0.496276	0.403727	9.591022	8.98E-10	9.48E-09
Itm2c	0.496262	1.889014	10.78906	8.55E-11	1.11E-09
Rhoc	0.494746	1.058177	6.571361	7.67E-07	4.43E-06
Ceacam1	0.494717	0.894472	6.185878	1.97E-06	1.05E-05
Extl1	0.494259	0.619432	8.230337	1.62E-08	1.30E-07
Lgals3	0.491881	1.613083	6.52326	8.62E-07	4.91E-06
Pltp	0.490652	0.655672	7.120621	2.06E-07	1.33E-06
Gm5431	0.489931	0.467445	8.737512	5.36E-09	4.87E-08
Fap	0.488942	0.370228	7.249796	1.52E-07	1.01E-06
Slc30a4	0.488153	1.114431	9.785621	6.06E-10	6.58E-09
Zdhhc14	0.487701	1.133964	7.420346	1.02E-07	7.01E-07
Gal3st2	0.486535	1.056372	7.117933	2.07E-07	1.34E-06
Anxa2	0.485861	2.411732	10.01259	3.85E-10	4.46E-09
Socs2	0.485258	0.73465	7.272957	1.44E-07	9.60E-07
Arhgef9	0.484648	0.580366	9.271279	1.73E-09	1.73E-08
St5	0.484414	0.584711	12.48567	4.06E-12	7.31E-11
Slfn1	0.481092	0.767769	4.909142	4.96E-05	0.000197
Rab9	0.480827	1.561084	4.50275	0.000141	0.000504
Ccdc88a	0.480233	2.503934	13.47171	7.91E-13	1.71E-11
Tnfsf9	0.479234	0.929872	9.186917	2.07E-09	2.02E-08

Tspan17	0.478776	0.937545	7.17469	1.81E-07	1.19E-06
Ankib1	0.4787	1.578164	12.04496	8.69E-12	1.47E-10
Ifnk	0.478583	0.600161	6.441832	1.05E-06	5.88E-06
Atg5	0.47719	1.01917	11.90335	1.11E-11	1.83E-10
Klf8	0.474752	0.742152	8.203981	1.72E-08	1.38E-07
Rasgrp1	0.474358	0.725582	6.187354	1.96E-06	1.04E-05
Pgm2l1	0.474224	0.497541	8.381721	1.16E-08	9.59E-08
Cacnb1	0.473684	0.81461	10.95764	6.22E-11	8.35E-10
Zc2hc1a	0.473595	0.98517	10.58753	1.25E-10	1.58E-09
Ap2a2	0.472913	1.298365	11.24442	3.66E-11	5.14E-10
Gm16364	0.471634	0.460263	8.04889	2.43E-08	1.89E-07
Pgap2	0.471503	1.517038	11.09863	4.79E-11	6.58E-10
Cd44	0.471326	1.774418	8.860126	4.12E-09	3.83E-08
Sav1	0.471027	1.589284	11.56801	2.03E-11	3.08E-10
Orai3	0.470928	1.073469	6.762149	4.83E-07	2.90E-06
Daam1	0.47076	1.226687	9.275999	1.72E-09	1.71E-08
Tmem63b	0.468788	1.029485	6.852966	3.89E-07	2.38E-06
Aldoc	0.468731	0.432731	8.177089	1.82E-08	1.45E-07
Birc2	0.466738	1.929324	10.04778	3.59E-10	4.20E-09
Mir3109	0.466515	0.600569	5.070366	3.28E-05	0.000135
Ttc39a	0.466457	1.375083	7.667782	5.76E-08	4.18E-07
Klhl6	0.466036	1.159268	9.442183	1.22E-09	1.26E-08
Sgcb	0.464711	0.540859	6.16518	2.08E-06	1.09E-05
Cx3cl1	0.463595	0.450788	9.748974	6.52E-10	7.01E-09
Il15	0.463442	1.738874	5.520881	1.04E-05	4.76E-05
Dnase1l1	0.463377	1.297388	9.829467	5.55E-10	6.12E-09
Rcsd1	0.46281	1.315821	9.924319	4.59E-10	5.17E-09
Map3k4	0.462654	0.690271	10.41063	1.76E-10	2.16E-09
Plekhb2	0.462443	1.449872	9.991785	4.01E-10	4.63E-09
Cmtm7	0.462364	1.787841	7.510537	8.27E-08	5.80E-07
Mxd1	0.462164	2.042452	7.695227	5.41E-08	3.94E-07
Tnfrsf18	0.460503	1.451163	8.70638	5.73E-09	5.17E-08
Stxbp1	0.460482	0.689244	8.693221	5.90E-09	5.31E-08
Oprd1	0.459904	0.863086	8.140054	1.98E-08	1.57E-07
Auh	0.458994	0.786652	6.709961	5.48E-07	3.26E-06
Sh3bp4	0.458249	0.96333	7.930525	3.17E-08	2.40E-07
Rab27a	0.458032	1.132136	8.145847	1.96E-08	1.55E-07
Vps37b	0.45777	0.606575	7.328868	1.26E-07	8.52E-07
Lrp8	0.456892	0.788292	8.147404	1.95E-08	1.55E-07
Gch1	0.456874	0.95672	7.039009	2.50E-07	1.59E-06
Epcam	0.456481	0.71296	8.531955	8.36E-09	7.20E-08
Cd38	0.456457	0.847444	9.804638	5.83E-10	6.36E-09
Nenf	0.455892	1.394453	8.138714	1.99E-08	1.57E-07
Prkra	0.455853	1.156232	9.478126	1.13E-09	1.17E-08
Hmga2-ps1	0.454326	1.027864	6.133745	2.24E-06	1.17E-05
Basp1	0.453865	2.068723	8.684677	6.01E-09	5.39E-08
E030011O(	0.453781	0.60989	7.614986	6.50E-08	4.67E-07
Rgag4	0.453557	0.639918	7.752114	4.75E-08	3.50E-07
Pdcd1lg2	0.45258	0.657869	7.75479	4.72E-08	3.48E-07
Stambpl1	0.452467	0.691753	6.248396	1.69E-06	9.11E-06
Ndrg1	0.452128	1.177873	10.02005	3.79E-10	4.40E-09
Gpr137b	0.450832	1.176924	8.01998	2.59E-08	2.01E-07

Gm8221	0.44956	1.224465	7.131936	2.00E-07	1.30E-06
Dstn	0.449045	2.018849	8.288473	1.43E-08	1.16E-07
Gba	0.448472	2.124688	10.95233	6.29E-11	8.40E-10
Hmgn3	0.448288	1.603101	7.717487	5.14E-08	3.76E-07
Trip10	0.446931	0.77334	7.495668	8.56E-08	6.00E-07
Spsb1	0.445223	0.993467	7.279735	1.41E-07	9.48E-07
Mospd1	0.443093	1.340258	8.034849	2.51E-08	1.95E-07
Ctns	0.442796	0.827448	8.891486	3.86E-09	3.59E-08
Slc23a2	0.441636	0.879101	10.75221	9.16E-11	1.19E-09
Hadh	0.441545	0.751349	9.768512	6.27E-10	6.79E-09
Rasa2	0.4408	1.437794	7.142656	1.95E-07	1.27E-06
Arc	0.440644	0.848977	8.388344	1.14E-08	9.49E-08
Slc48a1	0.43922	0.897197	9.42796	1.25E-09	1.29E-08
Slpi	0.438084	0.250358	4.58229	0.000115	0.000418
Prdx2	0.437755	1.59149	9.062274	2.69E-09	2.58E-08
BE692007	0.437384	0.490025	5.822953	4.87E-06	2.39E-05
Got1	0.43677	0.835094	8.438638	1.02E-08	8.57E-08
Mmp7	0.43627	0.418815	7.737389	4.91E-08	3.61E-07
Gm5424	0.435972	1.228761	8.706769	5.73E-09	5.17E-08
N28178	0.435449	0.66764	7.374362	1.13E-07	7.72E-07
Fam129a	0.433369	1.881646	10.83324	7.86E-11	1.03E-09
Spn	0.432551	0.722005	6.570827	7.68E-07	4.43E-06
Slc5a3	0.431493	0.450118	5.197029	2.37E-05	0.000101
Nfe2l1	0.431319	1.698598	9.142152	2.27E-09	2.20E-08
Smurf1	0.429853	1.145056	8.913136	3.68E-09	3.44E-08
Tmem154	0.429641	0.592244	6.167121	2.07E-06	1.09E-05
2200002D(	0.429064	1.411575	6.612505	6.94E-07	4.04E-06
Tmem19	0.428614	1.490247	8.386468	1.15E-08	9.50E-08
Reep5	0.427498	1.840707	7.931549	3.16E-08	2.39E-07
Rhbdf2	0.426993	0.727885	8.787279	4.82E-09	4.41E-08
Rem1	0.42692	0.413874	7.857045	3.74E-08	2.81E-07
Psd3	0.426752	0.420679	6.666732	6.09E-07	3.58E-06
Enpp4	0.42654	0.811897	7.018024	2.62E-07	1.66E-06
2610018G(	0.426071	0.330204	8.214959	1.68E-08	1.35E-07
Lyrm4	0.423998	1.148113	6.807564	4.33E-07	2.63E-06
Soga1	0.423921	0.457018	7.658243	5.89E-08	4.26E-07
Prelid2	0.423838	0.810188	8.127546	2.04E-08	1.61E-07
Zdhhc23	0.422998	0.873227	7.068715	2.33E-07	1.49E-06
Ppfibp1	0.422239	1.119693	11.26195	3.54E-11	5.01E-10
Usp11	0.422175	0.896793	8.55841	7.89E-09	6.86E-08
Rap2b	0.421146	1.397802	7.171528	1.82E-07	1.19E-06
Evi5	0.421005	0.690077	7.981132	2.83E-08	2.17E-07
Suox	0.420755	1.140429	7.469711	9.09E-08	6.31E-07
Trio	0.418	1.51169	10.20589	2.63E-10	3.13E-09
Gltp	0.416731	2.460712	7.803604	4.23E-08	3.14E-07
Iigp1	0.415466	0.53071	7.654712	5.94E-08	4.29E-07
AI839979	0.415409	0.442864	5.643847	7.64E-06	3.60E-05
Gem	0.415216	0.666936	5.4041	1.40E-05	6.27E-05
Glcci1	0.415188	1.007436	6.875426	3.68E-07	2.26E-06
Tubb2a	0.414732	2.160085	7.577797	7.08E-08	5.04E-07
Ift43	0.414512	0.841741	6.977328	2.89E-07	1.82E-06
Ptafr	0.414364	1.20924	7.510686	8.27E-08	5.80E-07

Tmed10	0.41426	1.994892	6.466871	9.89E-07	5.56E-06
Slc31a2	0.414205	1.112059	9.884529	4.97E-10	5.53E-09
Txndc17	0.413906	2.564344	8.482809	9.31E-09	7.88E-08
Mir677	0.413709	0.693991	4.157761	0.000341	0.001115
Swap70	0.413543	2.18651	7.146512	1.94E-07	1.26E-06
Dtx3	0.41343	0.787295	7.429305	9.98E-08	6.87E-07
Lztfl1	0.413367	1.06235	6.013853	3.02E-06	1.54E-05
Mpnd	0.413292	1.897572	8.603674	7.16E-09	6.27E-08
Gm21188	0.412636	1.08182	4.071837	0.000425	0.001357
Mtss1l	0.412402	0.731631	7.147362	1.93E-07	1.26E-06
Fam53b	0.412337	1.362338	8.478901	9.39E-09	7.94E-08
Anxa7	0.412035	1.528564	10.3217	2.10E-10	2.52E-09
Clec12a	0.411175	2.060457	6.289248	1.53E-06	8.31E-06
Nrp1	0.410867	0.574057	10.35208	1.98E-10	2.40E-09
Catsperg1	0.410165	0.561485	4.083121	0.000413	0.001324
Sptbn1	0.409666	1.302916	9.987245	4.05E-10	4.66E-09
Gtf2i	0.40924	1.016727	8.810686	4.58E-09	4.22E-08
Tbl1x	0.40905	1.393624	8.619906	6.91E-09	6.07E-08
Plcg1	0.408753	0.932072	8.288913	1.42E-08	1.16E-07
Raph1	0.405646	0.83175	9.869803	5.12E-10	5.68E-09
Gm9994	0.405622	0.668054	6.322703	1.41E-06	7.73E-06
Traf6	0.404738	1.592552	9.939729	4.45E-10	5.03E-09
Arl5a	0.404253	2.074379	8.109971	2.12E-08	1.67E-07
Ptpn1	0.404126	0.957977	9.636168	8.20E-10	8.69E-09
Slc7a11	0.403933	0.325417	4.190097	0.000314	0.001037
Pfkp	0.402909	1.636393	6.050425	2.76E-06	1.42E-05
Lamp2	0.4028	1.77404	10.74419	9.30E-11	1.20E-09
Svil	0.402116	0.57603	9.422409	1.27E-09	1.30E-08
Ikzf4	0.401685	0.391826	6.639811	6.49E-07	3.80E-06
Zbtb10	0.401591	1.470934	5.466915	1.19E-05	5.42E-05
Amica1	0.401151	1.636441	5.666008	7.22E-06	3.42E-05
Capn2	0.400807	1.728566	9.610402	8.64E-10	9.14E-09
Gna15	0.400672	0.605968	7.281378	1.41E-07	9.45E-07
Mir1949	0.400585	0.649517	4.937729	4.61E-05	0.000184
Aif1	0.400302	2.618544	7.597894	6.76E-08	4.83E-07
Cnpy2	0.400074	1.754315	9.412235	1.30E-09	1.32E-08
Srd5a3	0.398832	0.864027	5.204214	2.33E-05	9.94E-05
As3mt	0.397938	0.869522	8.462718	9.72E-09	8.17E-08
Pepd	0.397234	1.010036	8.041478	2.47E-08	1.92E-07
Gpr146	0.396443	0.775832	8.982373	3.18E-09	3.02E-08
Ldhb	0.394913	0.448264	7.962477	2.95E-08	2.25E-07
Hcn3	0.394312	0.459492	5.746936	5.89E-06	2.84E-05
Agpat5	0.393701	1.019324	9.360558	1.44E-09	1.45E-08
Pias3	0.391115	1.207122	8.556754	7.92E-09	6.87E-08
Cnih1	0.390889	1.133074	7.738053	4.91E-08	3.60E-07
Maml2	0.389995	0.789006	6.313891	1.44E-06	7.85E-06
Vkorc1	0.388978	0.846346	5.319411	1.74E-05	7.61E-05
Fah	0.388775	0.580066	6.928744	3.24E-07	2.02E-06
Bcl2l11	0.388391	1.002162	6.745493	5.03E-07	3.01E-06
Tuba1a	0.388363	2.831032	5.212434	2.28E-05	9.75E-05
Mpp1	0.388144	1.058163	8.93262	3.53E-09	3.31E-08
Uck2	0.386643	0.933115	5.94429	3.59E-06	1.82E-05

Nedd4l	0.385926	1.305598	8.666992	6.24E-09	5.55E-08
Mmgt2	0.385716	0.556889	6.499642	9.13E-07	5.17E-06
Cpq	0.384344	1.0081	6.990339	2.80E-07	1.77E-06
Kif1b	0.382856	0.75289	8.291292	1.42E-08	1.15E-07
Acvr2a	0.38138	1.165717	8.955248	3.37E-09	3.18E-08
Fads1	0.38031	0.763973	8.316162	1.34E-08	1.10E-07
Nr4a3	0.379934	1.680369	3.772472	0.000909	0.002689
Psen2	0.379831	0.767109	9.35809	1.45E-09	1.46E-08
Ndnl2	0.379822	1.432504	6.412582	1.13E-06	6.29E-06
Man1b1	0.376596	0.963703	8.910657	3.70E-09	3.46E-08
Gypc	0.376554	0.885462	7.341677	1.22E-07	8.30E-07
Mtch1	0.376504	1.453558	8.237642	1.60E-08	1.28E-07
Pik3r3	0.376046	0.75918	7.222876	1.62E-07	1.07E-06
Sil1	0.375764	0.862356	7.732105	4.97E-08	3.65E-07
Rpain	0.375298	1.009718	6.556278	7.95E-07	4.58E-06
Tmem176a	0.374795	1.838152	6.600183	7.15E-07	4.16E-06
Loxl3	0.374357	0.86943	7.582251	7.01E-08	5.00E-07
Ergic3	0.37411	1.713544	8.185502	1.79E-08	1.43E-07
Renbp	0.373899	0.764277	6.223235	1.80E-06	9.63E-06
Igsf9	0.371782	0.665113	6.33634	1.36E-06	7.50E-06
Ict1	0.370882	1.252323	7.2255	1.61E-07	1.06E-06
Pitrm1	0.370874	0.991435	8.329497	1.30E-08	1.07E-07
Ifi44	0.370775	0.713141	6.554417	7.99E-07	4.60E-06
Gm8995	0.370663	1.579334	5.938726	3.64E-06	1.84E-05
Alkbh6	0.370178	1.098113	6.692597	5.72E-07	3.38E-06
Lmf1	0.370146	0.704204	6.257352	1.65E-06	8.92E-06
Mpp5	0.369762	1.289586	8.902012	3.77E-09	3.52E-08
Tapbpl	0.36923	1.939757	8.139645	1.98E-08	1.57E-07
Zeb2	0.368995	0.871679	5.769929	5.56E-06	2.69E-05
Fut8	0.368672	1.406355	9.948742	4.37E-10	4.97E-09
Zfp260	0.368408	0.900772	7.198983	1.71E-07	1.12E-06
1700021K1	0.367785	1.166199	8.303499	1.38E-08	1.13E-07
Ighm	0.36742	2.324287	6.057487	2.71E-06	1.40E-05
Ptpn21	0.366977	0.990903	7.602561	6.69E-08	4.79E-07
Flna	0.36672	1.976391	7.615938	6.49E-08	4.66E-07
Ppm1h	0.366158	1.39444	6.387202	1.20E-06	6.69E-06
Tmem18	0.364737	0.607469	8.841673	4.29E-09	3.98E-08
S100a13	0.364419	1.855669	7.06125	2.37E-07	1.51E-06
Asprv1	0.364283	1.083596	5.638154	7.75E-06	3.64E-05
Ntng2	0.363829	1.496819	4.92325	4.78E-05	0.00019
Rbms2	0.363748	0.565662	6.592852	7.28E-07	4.22E-06
Atrnl1	0.363476	1.177925	7.065337	2.34E-07	1.50E-06
Atp6ap2	0.362377	1.535063	8.967697	3.28E-09	3.10E-08
Anxa4	0.361619	1.820443	7.61449	6.51E-08	4.67E-07
Zyx	0.361513	1.932643	5.744098	5.93E-06	2.86E-05
Htr7	0.361477	0.428286	8.06189	2.36E-08	1.84E-07
Brk1	0.361422	2.372041	5.221225	2.23E-05	9.56E-05
Gramd3	0.361123	1.645431	9.714599	6.99E-10	7.49E-09
Etfb	0.360953	1.918287	6.231729	1.76E-06	9.45E-06
Acadl	0.360418	1.894119	8.672221	6.17E-09	5.50E-08
Mrpl14	0.360191	1.368869	5.080201	3.20E-05	0.000132
Ell2	0.359701	1.666238	8.505574	8.86E-09	7.56E-08

Uap1l1	0.359431	0.978943	7.232138	1.58E-07	1.05E-06
Atic	0.359374	1.32378	4.987439	4.06E-05	0.000164
Gm16617	0.358971	1.031325	5.795359	5.22E-06	2.54E-05
Hook3	0.358964	1.915339	10.92617	6.60E-11	8.76E-10
LOC10264(	0.358617	1.140068	3.769828	0.000915	0.002704
Eps15	0.358595	2.092115	9.165889	2.16E-09	2.11E-08
Cflar	0.357848	1.813794	5.797608	5.19E-06	2.52E-05
Fam110a	0.356557	0.958471	6.70978	5.48E-07	3.26E-06
Dyrk3	0.356038	0.509822	6.922085	3.30E-07	2.05E-06
Clic4	0.355246	2.599169	8.337338	1.28E-08	1.05E-07
Ahcyl2	0.355044	1.12977	8.793366	4.76E-09	4.37E-08
Hsd3b7	0.353587	0.857799	5.372651	1.52E-05	6.74E-05
Elp3	0.353174	0.725352	6.145121	2.18E-06	1.14E-05
Atraid	0.352935	1.534503	8.812138	4.57E-09	4.21E-08
C3	0.352805	0.66318	3.663861	0.001196	0.003426
Arhgap28	0.352583	0.906456	5.482937	1.15E-05	5.21E-05
Gsr	0.352349	1.219569	7.059029	2.38E-07	1.52E-06
Pam	0.35228	0.552285	5.345587	1.63E-05	7.17E-05
Arhgef40	0.350555	1.085607	5.447868	1.25E-05	5.66E-05
Ctsb	0.349517	2.424652	6.932609	3.21E-07	2.01E-06
4930562F(	0.349263	1.692376	5.837622	4.69E-06	2.31E-05
Nipsnap3b	0.349065	1.088974	6.196228	1.92E-06	1.02E-05
Trim24	0.348814	1.480763	7.455699	9.39E-08	6.50E-07
Dpysl2	0.348791	1.402826	6.145215	2.18E-06	1.14E-05
Lpcat3	0.348696	1.015745	6.458756	1.01E-06	5.67E-06
4930523C(	0.348687	1.654016	7.575907	7.11E-08	5.06E-07
Il23r	0.348523	0.56828	4.133269	0.000363	0.001182
Nucb1	0.348259	1.102762	8.729416	5.45E-09	4.95E-08
Scarb2	0.34806	1.72486	6.422053	1.10E-06	6.16E-06
Hivep1	0.348057	2.34146	6.317328	1.43E-06	7.82E-06
E130311K1	0.34773	0.803226	5.552124	9.63E-06	4.44E-05
Iglv1	0.347172	0.063157	2.746077	0.011127	0.024655
Cdc7	0.346989	0.558198	6.345407	1.33E-06	7.36E-06
Slc9a1	0.346378	1.51975	7.393896	1.08E-07	7.41E-07
Rnh1	0.345485	1.603914	6.519061	8.71E-07	4.95E-06
Ypel2	0.345414	0.730603	4.199849	0.000306	0.001015
Itgb8	0.345182	0.662504	5.536084	1.00E-05	4.60E-05
Gnai3	0.345166	1.845011	7.033959	2.53E-07	1.60E-06
Adsl	0.344695	0.847999	7.699861	5.35E-08	3.91E-07
Bcl7a	0.34391	0.773637	4.265225	0.000259	0.000874
Scn4b	0.34365	0.47157	7.646896	6.04E-08	4.35E-07
D730005E1	0.343557	1.002183	7.154024	1.90E-07	1.24E-06
Serpinc1	0.343374	0.448395	8.505703	8.85E-09	7.56E-08
Cd84	0.343198	0.871161	4.63321	0.000101	0.000372
Cln6	0.342716	1.162835	4.767364	7.14E-05	0.000272
Apc	0.342538	1.192116	8.205529	1.71E-08	1.37E-07
Me2	0.342456	1.745593	8.05265	2.41E-08	1.88E-07
Msmp	0.341996	1.09546	7.108288	2.12E-07	1.37E-06
Fmn1	0.341411	0.736684	6.343132	1.34E-06	7.39E-06
Rbpms	0.340113	1.163332	6.87461	3.69E-07	2.26E-06
Gpcpd1	0.339654	2.093368	7.720058	5.11E-08	3.74E-07
Csrp1	0.33962	2.126871	4.854015	5.71E-05	0.000224

Caprin2	0.339543	0.796245	5.093707	3.09E-05	0.000128
Rps6ka5	0.339508	1.158086	7.486533	8.74E-08	6.09E-07
Mcemp1	0.338223	1.841413	6.918114	3.33E-07	2.07E-06
2610524H(	0.337569	1.489232	3.062761	0.005263	0.012845
Erp44	0.337455	1.960866	9.1025	2.47E-09	2.39E-08
Tom1l2	0.336947	0.662701	6.9029	3.45E-07	2.13E-06
Tulp4	0.336843	0.986553	5.987021	3.23E-06	1.64E-05
Olfr110	0.336783	0.316335	3.797024	0.000855	0.002554
Tnni3	0.336241	1.231613	6.795017	4.47E-07	2.70E-06
Ube2r2	0.336149	1.658237	6.174695	2.03E-06	1.07E-05
Tmem120b	0.335878	0.891588	5.133527	2.79E-05	0.000116
Cbr3	0.33584	0.600865	4.518651	0.000135	0.000485
Flt3	0.335391	2.772474	8.636433	6.67E-09	5.90E-08
Lactb2	0.33509	0.866791	8.54531	8.12E-09	7.03E-08
Tnni2	0.334989	1.708359	8.017759	2.60E-08	2.02E-07
Gm3086	0.334415	1.05655	5.453055	1.24E-05	5.60E-05
Uggt1	0.3344	1.692917	9.264731	1.76E-09	1.75E-08
Slamf6	0.333422	0.957383	4.799502	6.57E-05	0.000254
Bcl2	0.332284	0.787208	4.985809	4.07E-05	0.000164
Syngr2	0.33199	3.36621	7.297381	1.36E-07	9.13E-07
Dnajb9	0.331585	2.317131	5.729356	6.16E-06	2.96E-05
Krtcap2	0.330715	1.803487	6.158779	2.11E-06	1.11E-05
Glmp	0.330618	1.788863	5.136354	2.77E-05	0.000116
Kcnn4	0.330419	0.605814	6.330353	1.38E-06	7.60E-06
Il6st	0.330223	2.136732	5.524685	1.03E-05	4.72E-05
Grap	0.330051	0.893598	5.541589	9.89E-06	4.55E-05
Lrrc42	0.329883	1.108289	5.664118	7.26E-06	3.43E-05
Cd79b	0.329332	0.739773	5.016564	3.77E-05	0.000153
Zfp608	0.327508	0.555017	7.331618	1.25E-07	8.48E-07
Zdhhc3	0.327361	1.242655	6.581146	7.49E-07	4.34E-06
Vcam1	0.326865	1.010882	4.388554	0.000189	0.000655
Tnfrsf1b	0.32665	1.924226	5.279658	1.92E-05	8.33E-05
Itpripl1	0.326368	1.067469	5.452497	1.24E-05	5.61E-05
LOC10263:	0.326296	0.71979	4.173123	0.000328	0.001078
Lrp6	0.326208	1.445656	6.844437	3.97E-07	2.42E-06
Zfp472	0.325781	0.971577	5.894212	4.07E-06	2.04E-05
Nostrin	0.325684	1.505547	5.040006	3.55E-05	0.000145
Spin1	0.324239	1.66425	7.082723	2.25E-07	1.45E-06
Appl1	0.323816	1.805382	7.70565	5.28E-08	3.86E-07
Ctse	0.323695	0.507667	5.318739	1.74E-05	7.62E-05
LOC10263(	0.322849	0.100219	5.619146	8.13E-06	3.80E-05
Irf4	0.322073	1.032214	5.647588	7.56E-06	3.57E-05
Rab6b	0.321633	0.480141	3.861025	0.000727	0.002217
S100a10	0.321408	2.370493	4.377105	0.000194	0.000671
Dctn6	0.32118	1.926199	7.563399	7.32E-08	5.19E-07
Atxn1l	0.321042	1.082762	4.688921	8.73E-05	0.000327
Mesdc2	0.321029	0.715824	4.069353	0.000428	0.001365
Ikbkb	0.320421	1.880655	8.334022	1.29E-08	1.06E-07
Cpt1a	0.320359	1.037599	5.131825	2.80E-05	0.000117
Tbc1d15	0.320343	1.744822	7.878695	3.56E-08	2.68E-07
Pianp	0.32033	0.494234	5.012556	3.80E-05	0.000155
Pdia4	0.320069	1.425271	5.451525	1.24E-05	5.62E-05

Katnbl1	0.319861	1.265737	5.306438	1.80E-05	7.84E-05
Stx3	0.318921	1.015655	5.447229	1.26E-05	5.66E-05
Atp1b3	0.318867	2.092769	6.321641	1.41E-06	7.75E-06
Pigyl	0.318355	1.09501	5.659495	7.34E-06	3.47E-05
Dpagt1	0.318087	1.388521	7.540051	7.73E-08	5.46E-07
Naip2	0.31776	1.103403	5.976001	3.32E-06	1.69E-05
Sptan1	0.317721	1.761933	9.809227	5.78E-10	6.31E-09
Lrrc8c	0.317417	2.495332	7.204509	1.69E-07	1.11E-06
Adk	0.317393	0.521732	4.921614	4.80E-05	0.000191
AI504432	0.31722	0.924024	4.439063	0.000166	0.000583
Olfr111	0.31661	0.509215	2.448242	0.021873	0.044283
Erlec1	0.3166	1.495002	7.208856	1.67E-07	1.10E-06
Tram2	0.316234	0.891011	6.249016	1.69E-06	9.10E-06
Traj5	0.316175	0.418678	3.188027	0.003887	0.009788
Zfc3h1	0.316016	2.489737	7.315426	1.30E-07	8.78E-07
Zdhhc20	0.315692	1.410651	5.548021	9.73E-06	4.48E-05
Klhl9	0.315687	1.317316	7.15653	1.89E-07	1.23E-06
Klrb1b	0.314918	0.628762	4.66645	9.25E-05	0.000345
Tyk2	0.314811	1.750003	7.560262	7.37E-08	5.22E-07
Mllt11	0.314324	1.012897	6.29463	1.51E-06	8.21E-06
Unc119b	0.314267	0.826681	4.828345	6.10E-05	0.000238
Rassf2	0.313767	1.58418	5.700925	6.61E-06	3.17E-05
Acot9	0.313737	1.185064	4.670624	9.15E-05	0.000342
Ccdc90b	0.313648	1.423929	8.412289	1.09E-08	9.06E-08
1110001JC	0.313446	1.349997	4.632481	0.000101	0.000373
Sp3os	0.313295	0.735816	4.979253	4.14E-05	0.000167
Tmem55a	0.313072	0.722991	5.558008	9.48E-06	4.38E-05
Abhd2	0.312803	1.591629	8.931305	3.54E-09	3.32E-08
Fkbp2	0.312586	1.992986	4.892155	5.18E-05	0.000205
Arf3	0.312456	1.4237	3.607165	0.001379	0.003866
Mnda	0.312114	1.8001	4.932305	4.67E-05	0.000186
Rad54l2	0.310474	0.866138	5.492243	1.12E-05	5.10E-05
Gadd45a	0.310472	0.81667	5.44915	1.25E-05	5.64E-05
Gpr126	0.310464	1.041799	4.742652	7.60E-05	0.000288
Gucd1	0.310362	0.829658	5.889619	4.12E-06	2.05E-05
Sucla2	0.310258	1.488653	7.592104	6.85E-08	4.89E-07
Runx1	0.309699	1.377924	5.930326	3.72E-06	1.87E-05
Icosl	0.309661	2.392715	7.741733	4.86E-08	3.58E-07
Zfp358	0.309585	1.096192	4.918336	4.84E-05	0.000192
Anxa5	0.30925	2.454581	7.373652	1.14E-07	7.72E-07
Ankrd12	0.308363	1.56527	5.583935	8.88E-06	4.13E-05
Olfr108	0.308305	0.497425	4.81031	6.39E-05	0.000247
Aldh2	0.308261	0.895295	5.393486	1.44E-05	6.42E-05
Fabp4	0.308018	0.370506	2.654647	0.013736	0.029454
Asl	0.307813	1.447106	5.239066	2.13E-05	9.17E-05
Plin3	0.307614	1.017952	6.312986	1.44E-06	7.87E-06
Dnpep	0.30737	1.139406	5.438477	1.28E-05	5.77E-05
Car13	0.307191	0.44195	5.812945	4.99E-06	2.44E-05
Kctd6	0.307062	0.882286	4.702533	8.43E-05	0.000317
Ech1	0.306999	1.467095	4.811753	6.37E-05	0.000247
LOC10263:	0.306836	0.771278	5.870078	4.32E-06	2.15E-05
Lman1	0.306538	1.268083	4.992789	4.00E-05	0.000162

Tapbp	0.305839	2.301303	6.479236	9.59E-07	5.41E-06
Gm5617	0.305736	1.379358	4.978337	4.15E-05	0.000167
Stx11	0.305433	0.888302	5.679881	6.97E-06	3.31E-05
H1f0	0.30481	1.88557	5.18418	2.45E-05	0.000104
Ctla2b	0.304183	0.453506	4.21998	0.000291	0.000969
Gatad1	0.303557	1.723956	7.464288	9.21E-08	6.39E-07
1600002K(	0.303509	0.941923	5.267948	1.98E-05	8.57E-05
Myo1c	0.303344	1.0744	9.018025	2.95E-09	2.81E-08
5430435G:	0.302889	1.001676	5.591122	8.72E-06	4.06E-05
Pbx1	0.302844	1.328937	6.673584	5.99E-07	3.53E-06
Rgp1	0.302825	1.204256	6.635615	6.56E-07	3.84E-06
Ago1	0.302579	0.996859	5.94903	3.55E-06	1.80E-05
Nme3	0.302495	0.952602	7.389958	1.09E-07	7.47E-07
Prr5	0.302203	0.591599	3.835866	0.000775	0.002339
Cd209a	0.302176	0.963598	4.488605	0.000146	0.00052
Gm21540	0.301266	1.836958	6.735169	5.16E-07	3.08E-06
Tdrd3	0.300779	1.072999	7.959279	2.97E-08	2.26E-07
Desi1	0.300313	1.094542	4.78735	6.78E-05	0.00026
E430025E2	0.300188	1.535233	6.97417	2.91E-07	1.83E-06
Serp1	0.299288	1.756898	4.472705	0.000152	0.000539
Bcl3	0.299179	1.818395	4.619893	0.000104	0.000383
Ginm1	0.298774	1.236206	5.686555	6.86E-06	3.27E-05
Tmem223	0.298358	1.425112	5.085277	3.16E-05	0.00013
Slc7a6os	0.298325	1.301983	5.166284	2.57E-05	0.000108
Rassf3	0.298215	1.375983	5.125101	2.85E-05	0.000119
LOC10263ℰ	0.298184	0.517664	4.865786	5.54E-05	0.000218
Lpar3	0.298046	0.785904	5.800121	5.15E-06	2.51E-05
Man1a2	0.297808	1.316269	6.964169	2.98E-07	1.87E-06
Fxyd5	0.297623	2.735818	6.52582	8.56E-07	4.88E-06
Gpr155	0.297581	0.509473	6.171882	2.04E-06	1.08E-05
Hspb6	0.29744	0.948678	4.18642	0.000317	0.001046
Gtf2e2	0.297174	1.579675	6.443402	1.05E-06	5.86E-06
Tcp11l2	0.297003	0.485157	6.095558	2.47E-06	1.28E-05
Hsdl2	0.296006	1.102231	6.121574	2.31E-06	1.20E-05
Cd164	0.295881	2.159162	5.121604	2.88E-05	0.00012
Qser1	0.295529	0.866165	6.504361	9.02E-07	5.12E-06
Hipk2	0.295414	1.539964	6.138005	2.22E-06	1.16E-05
Zfp46	0.295224	0.87079	8.603096	7.16E-09	6.27E-08
Galnt12	0.294471	1.224008	5.397449	1.42E-05	6.36E-05
Ssr2	0.294437	1.953202	7.233461	1.58E-07	1.05E-06
Mob3b	0.294343	1.012009	4.395099	0.000186	0.000646
Ipcef1	0.293658	0.750139	3.598007	0.001411	0.003949
Spint2	0.293531	1.590263	5.850769	4.54E-06	2.25E-05
Prkrir	0.293494	1.858425	5.403066	1.40E-05	6.28E-05
Polr1d	0.293331	2.16135	6.494019	9.25E-07	5.23E-06
Mpi	0.293123	0.82562	4.823168	6.18E-05	0.000241
LOC10263⁄	0.292736	1.251305	4.379628	0.000193	0.000668
Ccl9	0.292709	0.88528	4.61868	0.000105	0.000384
Dock9	0.29218	0.413064	5.600513	8.52E-06	3.97E-05
Rptor	0.292038	1.347983	7.898389	3.41E-08	2.57E-07
Sidt2	0.292012	1.442783	5.084305	3.17E-05	0.00013
Rnf19b	0.292006	2.506641	6.592847	7.28E-07	4.22E-06

Tmem63a	0.291631	1.059029	7.65008	6.00E-08	4.33E-07
Smg8	0.291515	0.893795	4.717881	8.10E-05	0.000306
Sep-09	0.291216	1.365596	7.493231	8.61E-08	6.02E-07
Sh3kbp1	0.291043	1.787636	8.113107	2.10E-08	1.66E-07
Rspry1	0.290898	1.377421	6.724872	5.29E-07	3.15E-06
Ubtd2	0.290687	0.641043	3.663248	0.001198	0.00343
LOC10263(	0.29061	0.902834	3.57855	0.001481	0.004123
Zfp296	0.290318	1.215574	4.368931	0.000199	0.000684
Slc7a6	0.290044	0.961719	5.858738	4.45E-06	2.21E-05
Tmem176b	0.28982	2.226335	5.203804	2.33E-05	9.95E-05
Arhgef6	0.289248	2.05332	6.044614	2.80E-06	1.44E-05
Pmvk	0.288939	1.517941	4.117636	0.000378	0.001225
Gna11	0.288791	0.948643	3.883723	0.000686	0.002106
Trp53inp1	0.288685	1.36797	5.011532	3.81E-05	0.000155
Cyfip2	0.288026	2.04408	6.419043	1.11E-06	6.21E-06
Cfdp1	0.287834	2.040858	7.062744	2.36E-07	1.51E-06
Gm11545	0.287605	1.170084	4.042611	0.000458	0.001453
Rcn3	0.287218	0.586735	5.058743	3.38E-05	0.000138
Lnx2	0.286869	0.850314	5.309747	1.78E-05	7.78E-05
Hsf2	0.286836	1.23655	5.286325	1.89E-05	8.20E-05
Arhgap5	0.286808	1.496199	5.107216	2.99E-05	0.000124
D17Wsu10	0.286472	1.0524	6.194284	1.93E-06	1.03E-05
Pisd-ps1	0.286236	0.873125	2.943124	0.007006	0.016481
Acer3	0.28562	1.118582	4.391855	0.000187	0.00065
Dnajc10	0.285536	1.680811	5.313612	1.76E-05	7.71E-05
Rab26	0.285436	0.354606	4.095465	0.0004	0.001291
Sdf2l1	0.285178	1.633524	4.677665	8.98E-05	0.000336
B630005N:	0.285176	2.39077	6.031011	2.89E-06	1.48E-05
Atmin	0.28513	1.466215	5.787216	5.32E-06	2.58E-05
Prkag2	0.28477	0.960719	5.225829	2.21E-05	9.47E-05
Fibp	0.284514	1.348139	6.95	3.08E-07	1.93E-06
Arfgap3	0.284388	0.662978	5.344685	1.63E-05	7.18E-05
Olfr749	0.284212	0.629506	6.698809	5.63E-07	3.33E-06
D17Wsu92	0.283719	1.599748	5.468858	1.19E-05	5.39E-05
Gstp1	0.28366	1.920017	4.724344	7.97E-05	0.000301
Igf2r	0.283624	0.628175	5.318494	1.74E-05	7.62E-05
Snhg4	0.283614	1.09726	5.848321	4.57E-06	2.26E-05
Clec2d	0.283611	2.613639	5.631398	7.88E-06	3.70E-05
Creg1	0.283597	1.979443	5.150137	2.68E-05	0.000112
Rabac1	0.283556	1.864861	4.911383	4.93E-05	0.000196
Trim35	0.283334	1.218466	6.570944	7.67E-07	4.43E-06
Slc2a1	0.283213	1.133546	4.927729	4.73E-05	0.000188
Clnk	0.283093	0.727566	5.822114	4.88E-06	2.39E-05
Etv3	0.282992	2.063636	5.273421	1.95E-05	8.46E-05
Capn1	0.282463	1.345968	8.535006	8.31E-09	7.17E-08
Acaa2	0.281674	0.904178	5.534111	1.01E-05	4.62E-05
Slc25a29	0.28148	0.517846	4.736201	7.73E-05	0.000293
Kdelr1	0.281256	1.661366	4.701478	8.45E-05	0.000318
Carhsp1	0.281109	0.800547	5.326161	1.71E-05	7.50E-05
Yif1b	0.280932	1.109595	6.117116	2.34E-06	1.21E-05
Pcyox1	0.280205	0.784148	5.672411	7.11E-06	3.37E-05
Sdc3	0.280158	1.770428	5.091652	3.11E-05	0.000128

Snx29	0.280065	0.611086	5.211621	2.29E-05	9.76E-05
Copz1	0.27982	1.710786	5.322492	1.72E-05	7.56E-05
Zfp677	0.279331	0.833022	4.002925	0.000507	0.00159
Tpst2	0.279225	0.739604	5.420465	1.34E-05	6.02E-05
Gpr157	0.278926	1.367459	5.222827	2.22E-05	9.53E-05
Cux1	0.27823	1.41858	6.692362	5.72E-07	3.38E-06
Chst12	0.278037	0.907151	5.197207	2.37E-05	0.000101
R3hdm4	0.277915	1.275328	5.624118	8.03E-06	3.75E-05
Prr14	0.27749	1.670714	6.79225	4.50E-07	2.72E-06
Srgap2	0.277256	1.480294	5.753974	5.79E-06	2.79E-05
Txndc12	0.277216	1.207451	4.643897	9.80E-05	0.000364
Tmem9b	0.277129	1.36645	5.042837	3.52E-05	0.000144
Bet1	0.277024	0.866985	4.653373	9.56E-05	0.000356
Cep170	0.276932	1.787903	5.19301	2.40E-05	0.000102
Sep-11	0.276817	1.499039	4.671303	9.13E-05	0.000341
Klrk1	0.276704	1.41559	4.116567	0.000379	0.001228
Atl3	0.275998	1.569304	6.81455	4.26E-07	2.59E-06
Mien1	0.275738	1.899544	6.381892	1.22E-06	6.78E-06
Pde6d	0.275525	1.358609	4.398038	0.000184	0.000641
Sco2	0.275391	0.827728	3.356793	0.00257	0.006772
Tram1	0.274339	2.23856	5.865921	4.37E-06	2.17E-05
Prkab2	0.274291	1.077872	5.3346	1.67E-05	7.35E-05
Clybl	0.273965	0.547357	5.885582	4.16E-06	2.07E-05
Glt25d1	0.273789	1.46418	6.79563	4.46E-07	2.70E-06
Cdc42ep3	0.273401	1.921111	5.924286	3.78E-06	1.90E-05
Arl8b	0.273024	1.948094	6.548425	8.11E-07	4.66E-06
Rab2b	0.272448	0.736335	3.709656	0.001066	0.003084
Gm10277	0.272429	0.507032	3.801506	0.000845	0.002532
Hibadh	0.272156	1.11688	4.381906	0.000192	0.000665
Grina	0.271916	1.567498	4.860828	5.61E-05	0.000221
Nudt14	0.271799	1.28364	4.272617	0.000254	0.000859
Glod4	0.271597	1.493381	5.889679	4.12E-06	2.05E-05
AW11201C	0.271321	2.408805	3.795777	0.000857	0.002559
Txndc11	0.270773	1.284923	5.909683	3.92E-06	1.97E-05
Cbx4	0.270634	0.813624	4.459968	0.000157	0.000555
Cdkn2b	0.270563	0.604223	3.54979	0.001592	0.0044
Gramd1a	0.27029	1.317706	6.072066	2.61E-06	1.35E-05
Mapk1ip1l	0.269391	1.976415	4.802056	6.53E-05	0.000252
1300002E1	0.269343	0.726054	4.810869	6.38E-05	0.000247
Crat	0.269043	0.795442	4.131644	0.000365	0.001186
Ndufa7	0.268764	1.353529	5.594854	8.64E-06	4.02E-05
Mcu	0.268754	0.689851	3.89843	0.000661	0.002034
Ms4a6c	0.268739	1.522832	3.459762	0.001992	0.005385
Arhgef11	0.26832	1.076134	5.643486	7.64E-06	3.60E-05
Bag3	0.268246	0.857719	4.23652	0.000279	0.000933
Zfp935	0.267854	0.977571	2.715564	0.011941	0.026201
Zcchc24	0.267465	0.483006	5.842321	4.64E-06	2.29E-05
Ercc6l	0.267344	0.87784	5.258098	2.03E-05	8.77E-05
Iscu	0.266971	2.287795	5.107503	2.98E-05	0.000124
Bnip3l	0.266393	1.247282	5.78213	5.39E-06	2.62E-05
Lrrcc1	0.266221	1.416922	6.811006	4.30E-07	2.61E-06
Jmjd7	0.265929	1.004262	2.905422	0.007661	0.017819

Wdtc1	0.265555	0.615668	5.66998	7.15E-06	3.39E-05
Lsm10	0.26542	0.92291	5.146785	2.70E-05	0.000113
Polr2e	0.265198	1.688693	4.157934	0.000341	0.001115
Psmg4	0.264375	1.436456	4.373119	0.000196	0.000677
Ap3m2	0.264009	0.968108	5.366095	1.54E-05	6.84E-05
Rasa3	0.263917	0.853016	4.430243	0.00017	0.000595
Add1	0.263874	1.611381	5.583416	8.89E-06	4.13E-05
Snapin	0.26372	1.499026	4.315869	0.000228	0.000776
Nptn	0.262643	1.930863	5.161738	2.60E-05	0.000109
Atp6v0a1	0.262597	1.397497	5.356246	1.58E-05	7.00E-05
Kif2a	0.262451	1.961305	7.269357	1.45E-07	9.67E-07
Krcc1	0.262375	2.091349	6.708756	5.50E-07	3.27E-06
Lrrc1	0.262311	0.67994	5.399412	1.42E-05	6.33E-05
Lix1l	0.2623	0.824227	4.533034	0.00013	0.000469
Tmbim4	0.261883	2.418022	5.659833	7.33E-06	3.47E-05
Smc6	0.261788	1.836323	5.852526	4.52E-06	2.24E-05
Gm10865	0.261758	1.353832	5.001429	3.91E-05	0.000159
Trbj1-6	0.261715	1.296555	2.395438	0.024576	0.049158
Mettl4	0.261673	1.044135	4.735121	7.75E-05	0.000293
Coa5	0.261116	1.567591	5.824089	4.85E-06	2.38E-05
Gemin7	0.260998	1.542064	4.413476	0.000177	0.000619
Emc10	0.260794	0.833513	4.668255	9.20E-05	0.000344
Tpm1	0.260739	1.19498	6.316438	1.43E-06	7.83E-06
Golph3l	0.260414	1.796087	4.499955	0.000142	0.000506
Zeb2os	0.259834	0.347249	2.679058	0.012988	0.028134
Trappc3	0.259493	2.043771	4.589551	0.000113	0.000411
Tmem181a	0.259346	0.867974	3.094964	0.004871	0.012
Gtpbp8	0.259263	0.897922	5.540707	9.91E-06	4.56E-05
1700020I1	0.259263	1.607517	4.942159	4.56E-05	0.000182
Fam57a	0.259188	0.794406	3.708346	0.001069	0.00309
Nup62	0.259041	2.655219	4.604266	0.000108	0.000397
Atp6v1b2	0.258868	1.881978	6.338128	1.36E-06	7.47E-06
Klrd1	0.258419	1.534682	3.832037	0.000782	0.00236
Ramp1	0.258373	0.848351	4.96561	4.29E-05	0.000172
Emc3	0.257918	1.692176	6.98135	2.86E-07	1.80E-06
Itga1	0.257286	0.745357	5.424552	1.33E-05	5.97E-05
Il3ra	0.257103	0.877743	4.141407	0.000356	0.00116
Mlf2	0.257024	1.41807	4.027822	0.000475	0.001505
8430419L(	0.257004	0.689511	4.178467	0.000324	0.001065
Tnks	0.256997	1.402637	4.985529	4.08E-05	0.000164
Npc1	0.256878	1.152322	5.108251	2.98E-05	0.000124
Rps27l	0.256455	1.649675	5.353958	1.59E-05	7.03E-05
Sft2d2	0.256434	1.841966	3.850487	0.000746	0.002267
Vac14	0.256106	1.556124	6.812715	4.28E-07	2.60E-06
Slc38a2	0.25606	2.384187	5.635307	7.80E-06	3.66E-05
2310047M	0.255927	1.057899	3.569917	0.001513	0.004206
1110038B:	0.255622	1.427961	4.484606	0.000148	0.000525
Ptpra	0.255404	1.471081	6.274041	1.59E-06	8.60E-06
Tmem256	0.255068	1.352897	2.849608	0.008738	0.019956
D16Ertd47	0.254908	0.855161	5.201281	2.35E-05	1.00E-04
Pcgf3	0.254683	0.709243	4.429159	0.00017	0.000597
AU040320	0.254218	0.917334	5.528094	1.02E-05	4.68E-05

Zc3h7b	0.253826	0.965271	5.243865	2.11E-05	9.08E-05
Cdk6	0.253678	1.098477	3.721287	0.001035	0.00301
Oaz2	0.253394	1.569715	3.634164	0.001288	0.003645
Lrpap1	0.253201	1.332878	6.032719	2.88E-06	1.48E-05
Mrpl9	0.252724	1.109264	4.17183	0.000329	0.001079
Uchl3	0.252448	1.664702	3.715141	0.001051	0.003049
Snx4	0.25229	1.243367	3.843815	0.000759	0.002298
Bad	0.252103	0.98362	5.472322	1.18E-05	5.35E-05
Ccs	0.252093	0.731857	3.144844	0.004317	0.010754
Sf3b4	0.251772	0.788659	2.779759	0.010289	0.023011
1700021F(	0.251604	1.29056	3.809332	0.000828	0.002488
Zscan29	0.251313	1.30652	5.087046	3.14E-05	0.00013
Dnajc13	0.250851	1.366867	6.532398	8.43E-07	4.82E-06
Tmem261	0.250577	1.292521	4.874335	5.42E-05	0.000214
Fbxl4	0.250364	0.780311	3.49231	0.001837	0.004988
Src	0.250233	0.720017	5.874156	4.28E-06	2.13E-05
Ddost	0.250146	1.826661	4.667138	9.23E-05	0.000344
Frmd4b	0.249944	0.895505	6.352917	1.31E-06	7.23E-06
Cdc42se1	0.249825	2.250093	4.594566	0.000111	0.000407
Ndufa1	0.249757	2.556978	3.32195	0.002801	0.007312
Zdhhc9	0.249558	1.055676	3.28345	0.003079	0.007954
Gabarapl1	0.249459	0.857415	5.042527	3.52E-05	0.000144
BC005537	0.249407	2.499855	7.209783	1.67E-07	1.10E-06
Plekha2	0.249247	1.144004	4.615017	0.000106	0.000387
Serhl	0.249169	1.224453	5.024702	3.69E-05	0.00015
Eif2b2	0.248883	1.332492	4.214841	0.000295	0.00098
Nek7	0.248722	1.766263	6.329649	1.38E-06	7.61E-06
Smim15	0.248439	1.523423	5.444468	1.26E-05	5.69E-05
Sdhaf1	0.248416	1.424027	5.197712	2.37E-05	0.000101
Ssr4	0.248185	1.67405	4.576884	0.000116	0.000423
3110009E1	0.247979	0.704798	3.137982	0.004389	0.010912
Khk	0.247837	0.899358	2.903866	0.007689	0.017873
Ube2k	0.247644	2.345013	4.793042	6.68E-05	0.000257
Polr2i	0.247598	1.403283	4.394391	0.000186	0.000646
Slc25a12	0.247397	1.392016	6.199287	1.91E-06	1.01E-05
Lpar5	0.24725	0.946913	3.779114	0.000894	0.002648
C1qbp	0.247237	1.519618	5.174701	2.51E-05	0.000106
Ahcyl1	0.247181	1.923259	6.759363	4.87E-07	2.92E-06
Rfwd2	0.246952	2.618054	4.89293	5.17E-05	0.000205
Ddhd2	0.246586	1.085133	6.022971	2.95E-06	1.51E-05
Nsmce2	0.246516	1.33912	5.295994	1.84E-05	8.03E-05
Insr	0.246505	1.179864	5.728142	6.17E-06	2.97E-05
Lancl1	0.245548	1.067336	4.996126	3.97E-05	0.00016
Trappc11	0.245305	1.533764	5.460596	1.21E-05	5.50E-05
Kiz	0.24516	0.73898	4.394268	0.000186	0.000646
Ap3d1	0.24505	1.839831	5.533781	1.01E-05	4.62E-05
Med9	0.244957	1.323653	5.19406	2.39E-05	0.000101
Rtn4	0.244725	1.55496	5.227381	2.20E-05	9.44E-05
Cd69	0.244628	0.986414	3.900507	0.000657	0.002025
Mknk2	0.244533	1.517086	4.886085	5.26E-05	0.000208
Gm6625	0.24436	1.030089	2.567962	0.01673	0.035028
Fars2	0.244322	0.92921	4.880305	5.34E-05	0.000211

Eea1	0.243867	1.369937	5.812091	5.00E-06	2.44E-05
Slc10a3	0.243791	1.083027	3.442325	0.00208	0.005592
Myo18a	0.243647	1.133557	6.168833	2.06E-06	1.08E-05
Dynlt3	0.243424	1.512474	5.080732	3.20E-05	0.000131
Gm14586	0.243271	2.027504	3.184469	0.003921	0.009866
Fchsd2	0.242835	2.189032	5.83496	4.72E-06	2.32E-05
Golgb1	0.242723	1.094015	5.293056	1.86E-05	8.08E-05
Nsdhl	0.242222	1.187879	4.797399	6.61E-05	0.000255
Grk6	0.241882	0.657171	4.651723	9.60E-05	0.000357
Elmod2	0.24107	0.956578	4.548853	0.000125	0.000452
Tollip	0.240962	1.063781	5.248769	2.08E-05	8.98E-05
Sdf2	0.240531	1.552135	4.899725	5.08E-05	0.000201
Bmyc	0.240175	1.936736	2.937449	0.007101	0.016657
Ipmk	0.239829	1.319198	3.676889	0.001157	0.003324
Tapt1	0.239777	0.670857	3.736572	0.000996	0.002909
Mea1	0.239137	1.528687	5.106576	2.99E-05	0.000124
Eif3c	0.239088	2.597373	6.842946	3.98E-07	2.43E-06
Ankrd28	0.238526	0.466443	4.381619	0.000192	0.000665
Rps11	0.238424	1.620222	2.645732	0.014019	0.030035
1110032A(	0.238404	0.838763	3.82174	0.000803	0.00242
Itpripl2	0.238235	1.661602	5.539608	9.94E-06	4.57E-05
Eif3i	0.238027	2.392066	4.80686	6.45E-05	0.000249
Exoc6b	0.238023	1.098383	5.796541	5.20E-06	2.53E-05
Gpr183	0.237965	1.532051	4.006824	0.000502	0.001576
Hmg20b	0.237944	1.105956	4.787992	6.77E-05	0.00026
P2rx4	0.237914	1.589219	5.727233	6.19E-06	2.97E-05
5430437J1	0.237895	1.219056	2.752539	0.010961	0.024353
Ifi30	0.237759	3.065279	6.642279	6.46E-07	3.78E-06
Tbc1d16	0.237727	0.356773	5.357861	1.58E-05	6.97E-05
Gm10677	0.23751	0.709796	3.560526	0.001549	0.004301
Arl13b	0.237221	0.757632	4.486322	0.000147	0.000523
Slc27a3	0.236943	0.402496	3.231876	0.003493	0.008894
Gm19723	0.236604	0.915973	3.631962	0.001296	0.003658
Pet100	0.236499	1.656051	4.360959	0.000203	0.000696
Atp1a1	0.236379	2.284942	5.554636	9.57E-06	4.41E-05
Hspb11	0.23636	1.527609	3.714107	0.001054	0.003054
BC022960	0.235908	0.280048	2.910632	0.007567	0.017634
Ube2h	0.235765	0.704891	2.673014	0.01317	0.028461
Manf	0.235374	1.209556	4.68102	8.91E-05	0.000334
Dnm1l	0.235151	2.248798	5.907674	3.94E-06	1.97E-05
Cyp51	0.234903	1.198268	3.03488	0.005628	0.013618
P4hb	0.234678	1.950019	5.332715	1.68E-05	7.38E-05
Prkcd	0.234518	2.402512	5.91102	3.90E-06	1.96E-05
Gm2614	0.234464	1.500679	2.908408	0.007607	0.017704
Gbe1	0.234378	0.596497	5.021251	3.72E-05	0.000152
Ctsd	0.234363	1.341932	4.091464	0.000404	0.0013
Trappc6a	0.233972	1.181671	3.779838	0.000893	0.002645
Csf1	0.233972	0.664694	3.60617	0.001382	0.003875
Dip2b	0.23378	1.520626	5.240427	2.12E-05	9.15E-05
Ppapdc2	0.233598	0.972172	4.174912	0.000326	0.001073
Pip5k1c	0.233421	1.178214	4.426388	0.000171	0.0006
Mvp	0.232944	1.759409	5.32825	1.70E-05	7.46E-05

Lrrc59	0.232921	1.287572	5.688895	6.82E-06	3.25E-05
Ptcd2	0.232904	1.878451	6.519617	8.70E-07	4.95E-06
Dscr3	0.232129	0.933394	3.716305	0.001048	0.003043
Pea15a	0.231811	1.527559	4.105421	0.00039	0.001261
Tsta3	0.231334	1.035591	3.758252	0.000943	0.002768
Glb1	0.231266	0.953225	4.932422	4.67E-05	0.000186
LOC10263:	0.231261	0.670078	3.695445	0.001104	0.003185
Ssfa2	0.230814	1.745181	4.15993	0.000339	0.00111
Arg2	0.230372	0.607451	3.732708	0.001005	0.002933
Ccdc32	0.230183	0.83877	3.412363	0.00224	0.005977
Rgmb	0.229859	0.857312	4.973863	4.20E-05	0.000168
Arap1	0.229767	0.928221	6.795363	4.46E-07	2.70E-06
Mrps27	0.229743	0.570842	4.818386	6.26E-05	0.000243
2310045N(	0.229573	2.246201	5.270411	1.97E-05	8.52E-05
Nupr1l	0.22956	0.404079	2.909032	0.007596	0.017689
Bcl2l1	0.2294	0.787541	4.711136	8.24E-05	0.00031
Cdc37	0.229379	2.218127	5.393242	1.44E-05	6.42E-05
Tbcb	0.229341	2.228092	5.583726	8.89E-06	4.13E-05
Map1lc3b	0.22882	2.16521	5.236078	2.15E-05	9.24E-05
Dok1	0.228764	1.104665	3.914998	0.000634	0.001958
4833439L1	0.228762	1.532398	3.831326	0.000783	0.002363
Fbxl5	0.228572	1.707002	4.927082	4.74E-05	0.000189
Mpg	0.2285	0.756907	5.00174	3.91E-05	0.000158
Hmgcs1	0.228359	1.425415	3.926446	0.000615	0.001907
Spcs3	0.22833	2.10214	4.406883	0.00018	0.000628
Zfp760	0.228247	0.662411	3.6498	0.001239	0.00353
Slc25a4	0.228244	1.839789	3.658645	0.001212	0.003466
Tmem183a	0.228105	1.203456	4.751117	7.44E-05	0.000283
Shmt2	0.227969	0.811186	4.168123	0.000332	0.001088
LOC10263!	0.227766	0.789489	3.403066	0.002293	0.006107
Ak3	0.227632	1.06882	4.637441	9.96E-05	0.000369
Smarcd1	0.227582	0.829298	4.017858	0.000488	0.001537
Ostm1	0.22741	1.162236	3.672162	0.001171	0.003361
Jmjd8	0.227391	0.754339	3.043038	0.005519	0.013407
Mx1	0.227289	1.565562	2.55096	0.017385	0.036215
Prkcsh	0.227191	1.226794	5.597261	8.59E-06	4.00E-05
Prrc2b	0.226956	1.526479	6.0891	2.51E-06	1.30E-05
Tnfrsf21	0.226692	0.730056	4.994179	3.99E-05	0.000161
Ecsit	0.226507	0.683674	3.572295	0.001504	0.004183
Iqsec1	0.226246	1.22179	5.391236	1.45E-05	6.45E-05
Dhrs7	0.226011	0.824691	4.829928	6.08E-05	0.000237
Endod1	0.225862	0.898423	3.769627	0.000916	0.002704
Cacul1	0.225854	1.7363	5.778687	5.44E-06	2.64E-05
Zfp280c	0.225546	0.797522	4.23923	0.000277	0.000928
Zfp809	0.225511	1.577807	5.369907	1.53E-05	6.78E-05
Kcna3	0.225378	0.911147	4.341047	0.000213	0.00073
Rptoros	0.22534	0.832133	2.902852	0.007708	0.017905
Atp2b1	0.225305	2.297442	5.373028	1.52E-05	6.74E-05
Pdlim5	0.225207	1.736778	6.012121	3.03E-06	1.55E-05
Slc29a3	0.225047	1.797098	4.09264	0.000403	0.001298
Polr2j	0.225037	1.56625	5.108837	2.97E-05	0.000124
Psma7	0.224987	2.923277	6.19939	1.91E-06	1.01E-05

Htra2	0.224705	2.357047	5.445912	1.26E-05	5.68E-05
Parp1	0.224567	1.273033	4.83304	6.03E-05	0.000235
H2-Q5	0.22443	2.115101	4.474605	0.000151	0.000538
BC004004	0.224303	1.449595	6.100316	2.44E-06	1.26E-05
Ift22	0.223612	1.011312	4.031065	0.000472	0.001495
Pja2	0.223525	1.454523	4.508221	0.000139	0.000498
Yes1	0.223406	0.61453	3.215206	0.003638	0.009235
Nmrk1	0.223366	0.597904	4.622712	0.000103	0.000381
Rmnd5a	0.223363	1.293141	3.502865	0.001789	0.004876
Klf10	0.223101	0.662359	4.22542	0.000287	0.000958
A530064D(	0.222955	0.38739	3.088737	0.004944	0.012157
Fuca1	0.222866	2.620497	5.44687	1.26E-05	5.67E-05
Myo1e	0.222792	0.490999	4.133455	0.000363	0.001182
Zmpste24	0.222652	1.135589	4.247257	0.000271	0.000911
Os9	0.222246	1.839704	4.793315	6.68E-05	0.000257
Zfp369	0.222228	0.580286	2.822827	0.009305	0.021043
Dbi	0.222228	1.728493	4.527912	0.000132	0.000475
Commd2	0.222224	1.301365	3.149001	0.004274	0.010656
Tspan31	0.221867	1.340308	5.244369	2.10E-05	9.08E-05
Mpv17	0.221719	0.945184	4.184501	0.000319	0.001051
Mab21l3	0.221518	1.026433	3.695907	0.001103	0.003182
Ssr3	0.221295	2.044105	4.355458	0.000206	0.000705
2700060E(	0.221124	2.396936	2.770756	0.010507	0.023435
Tnfrsf11a	0.220739	1.08543	3.300059	0.002956	0.007679
Ghitm	0.220582	2.165654	5.680078	6.97E-06	3.31E-05
Tcf3	0.22022	1.224685	5.449985	1.25E-05	5.63E-05
Parp3	0.219891	0.990905	5.295742	1.85E-05	8.03E-05
Sc5d	0.219613	1.07531	3.856842	0.000734	0.002238
Mllt1	0.219337	1.030288	4.057538	0.000441	0.001402
Wdr3	0.21931	1.01356	5.442008	1.27E-05	5.73E-05
BC033916	0.21918	0.511197	2.817832	0.009414	0.021259
Sec11c	0.219122	2.620493	4.613307	0.000106	0.000388
Galk2	0.219014	0.900932	4.986108	4.07E-05	0.000164
Ctdnep1	0.218955	1.311353	3.275966	0.003136	0.008078
Nabp1	0.218948	2.790677	4.50213	0.000141	0.000504
Gab2	0.218897	1.539709	4.30401	0.000235	0.000797
Sec14l1	0.218462	0.712202	4.642372	9.84E-05	0.000365
Zfp524	0.218424	1.119765	4.66014	9.40E-05	0.00035
Mgat4b	0.218375	0.825432	3.998978	0.000512	0.001606
Pex19	0.217922	1.251685	5.213334	2.28E-05	9.73E-05
Glce	0.217729	1.486496	4.425027	0.000172	0.000602
Bhlhb9	0.216742	0.461353	5.584038	8.88E-06	4.13E-05
Pelo	0.216258	0.949086	3.151881	0.004244	0.010586
Rab11a	0.21618	1.909781	5.891712	4.10E-06	2.05E-05
C1galt1c1	0.216176	1.04184	3.454764	0.002017	0.005447
Slc25a1	0.215785	1.216083	3.972741	0.000547	0.001705
Acot13	0.215016	1.272536	3.201931	0.003758	0.009507
Tmem150c	0.214939	0.870861	3.002787	0.006078	0.014583
Dusp19	0.214777	0.879995	3.117682	0.004611	0.011408
Dpy19l4	0.214666	1.010341	4.773795	7.02E-05	0.000269
Tm7sf3	0.214562	0.491391	4.382705	0.000192	0.000664
Mknk1	0.214311	1.040658	3.993263	0.000519	0.001626

Scamp2	0.214054	2.185504	5.628588	7.94E-06	3.72E-05
Tmem50a	0.214019	1.661952	4.077597	0.000419	0.00134
Zfp568	0.213888	0.581829	3.550331	0.001589	0.004396
Ehd4	0.213864	0.981001	4.851293	5.75E-05	0.000225
Hyou1	0.213651	1.236729	3.410741	0.002249	0.005997
Ap1b1	0.213585	1.395117	3.746856	0.00097	0.002839
Cdc42se2	0.213582	1.863851	4.777258	6.96E-05	0.000267
Adnp2	0.213087	0.914598	2.962281	0.006694	0.015857
Man2b2	0.212991	1.374383	4.965626	4.29E-05	0.000172
Naip6	0.212989	1.28962	3.502178	0.001792	0.004883
Kctd10	0.21295	0.970014	4.374091	0.000196	0.000676
Txn1	0.21284	1.869155	3.844621	0.000758	0.002297
Rabgef1	0.212711	1.237373	3.537995	0.001639	0.004519
A730063M	0.21243	0.64322	3.046009	0.00548	0.013329
Alg9	0.212257	0.83374	5.294276	1.85E-05	8.06E-05
Cmtm6	0.211982	2.152147	5.026394	3.67E-05	0.00015
Spata6	0.21193	1.289805	3.582392	0.001467	0.004086
Lamtor2	0.211753	1.681011	2.869779	0.008333	0.019143
Nudt21	0.21153	1.11921	2.771552	0.010487	0.023406
Arfgef2	0.211388	0.927505	4.611286	0.000107	0.00039
Prmt1	0.211384	1.20024	3.558459	0.001557	0.004317
Psmd10	0.211258	1.859601	4.914386	4.89E-05	0.000194
Eml6	0.21092	1.032499	4.757291	7.32E-05	0.000278
Cib1	0.210884	1.011435	4.775963	6.98E-05	0.000268
Nsf	0.210871	1.234184	4.641668	9.85E-05	0.000365
Gtf3c6	0.210757	1.956061	3.862182	0.000725	0.002213
Hbs1l	0.210581	1.533171	4.773247	7.03E-05	0.000269
Fdft1	0.210549	1.419344	3.943825	0.000589	0.001828
Ndufa12	0.210539	2.060957	5.222724	2.22E-05	9.53E-05
Rfc1	0.210316	1.297362	4.02112	0.000484	0.001527
Mreg	0.210281	1.971536	3.483483	0.001878	0.005092
Sdad1	0.210202	1.372037	4.016836	0.000489	0.00154
Tmtc3	0.210189	0.898991	4.358327	0.000204	0.0007
Ormdl1	0.209735	1.02299	3.800822	0.000846	0.002534
Eid1	0.209696	1.482477	4.063469	0.000434	0.001384
Vps37a	0.209648	1.235255	4.238013	0.000278	0.00093
Arhgap21	0.209608	1.052036	4.816983	6.28E-05	0.000244
Cep57	0.209604	1.129592	5.009276	3.84E-05	0.000156
Srp54c	0.209422	1.319314	3.093465	0.004888	0.012035
Smim4	0.209134	1.140499	3.06551	0.005229	0.012773
Arl4c	0.208959	1.298164	4.641978	9.85E-05	0.000365
Bsg	0.208867	1.609705	3.978168	0.00054	0.001684
Sh3gl1	0.20868	1.495796	3.716753	0.001047	0.003041
Siah1a	0.208636	1.744566	3.500196	0.001801	0.004904
Edem2	0.208592	1.331608	4.054299	0.000444	0.001413
Orai1	0.208542	0.831009	4.024941	0.000479	0.001514
Rwdd4a	0.208497	1.071799	4.546964	0.000126	0.000454
Ccni	0.208481	2.12373	3.839349	0.000768	0.00232
Pi4k2b	0.208192	0.673468	3.618398	0.00134	0.003773
Ttc13	0.208048	0.801962	4.828398	6.10E-05	0.000238
Anapc2	0.20744	1.065811	5.358125	1.57E-05	6.97E-05
Setd7	0.207264	1.354162	4.865929	5.54E-05	0.000218

Ccdc186	0.207214	1.199172	4.693809	8.62E-05	0.000324
Tmed9	0.20717	0.671767	2.901413	0.007734	0.017955
Eif1a	0.206708	1.151629	4.470885	0.000153	0.000542
Mrps33	0.206686	1.23518	2.978903	0.006434	0.015335
Ostc	0.206584	1.514777	4.059944	0.000438	0.001395
Znrf1	0.2065	1.014356	4.216837	0.000293	0.000976
Tpra1	0.206287	0.882931	4.095032	0.0004	0.001291
Psmd3	0.206225	1.264047	3.666826	0.001187	0.003404
Gorasp2	0.206162	1.651729	4.2813	0.000249	0.000841
Naglu	0.206089	0.612515	2.826312	0.009229	0.020898
Myo1g	0.206076	2.356248	4.742358	7.61E-05	0.000288
Aaed1	0.206028	1.037395	3.723704	0.001028	0.002994
Mtif3	0.205859	1.069587	4.031992	0.00047	0.001492
Nudt1	0.205834	0.828606	3.607819	0.001376	0.003862
Ubald1	0.205559	1.079973	3.799651	0.000849	0.00254
Calr	0.205495	2.843114	3.38071	0.002423	0.006418
Cope	0.20534	1.995728	3.548905	0.001595	0.004409
Fam173a	0.204679	1.518238	4.841182	5.90E-05	0.000231
Ndufb9	0.20467	2.490018	4.500452	0.000142	0.000506
Jtb	0.204413	1.416877	3.843943	0.000759	0.002298
Kif16b	0.204345	1.039743	4.351043	0.000208	0.000713
Zbtb20	0.204228	1.167422	4.674484	9.06E-05	0.000339
Timm13	0.204222	1.37527	4.170199	0.00033	0.001084
Timm10b	0.203962	1.282418	4.017673	0.000488	0.001537
Vcl	0.203567	1.229866	4.6158	0.000105	0.000387
Cuta	0.203337	1.880337	4.010274	0.000497	0.001563
Nf2	0.203276	0.740075	4.415589	0.000176	0.000616
Socs7	0.203078	0.913098	4.106523	0.000389	0.001258
Appl2	0.203046	1.167624	4.374404	0.000196	0.000676
Dzip3	0.202906	1.047885	4.304677	0.000234	0.000796
Gm4024	0.202831	1.148339	3.655239	0.001222	0.00349
Slc35b4	0.202805	0.731916	3.772411	0.000909	0.002689
Nras	0.202634	1.908998	4.573903	0.000117	0.000426
Ldlr	0.202391	0.897639	3.595543	0.001419	0.003972
Hsd17b10	0.202306	1.365274	3.336265	0.002704	0.007083
Gm15698	0.202274	0.950787	2.813636	0.009507	0.021436
Slc41a1	0.202236	0.987662	4.927325	4.73E-05	0.000188
Rab11b	0.202225	0.963033	3.797618	0.000853	0.002552
Ergic1	0.20206	1.537056	3.321825	0.002802	0.007312
Nfkbia	0.202001	2.527387	5.137389	2.76E-05	0.000115
Ubxn4	0.20196	2.036651	4.700447	8.47E-05	0.000318
Ttc19	0.201936	0.684811	3.57719	0.001486	0.004135
Ankrd27	0.201893	1.101154	3.804934	0.000838	0.002514
Zdhhc18	0.201787	1.114434	4.177403	0.000324	0.001067
Sap30bp	0.201537	1.066417	3.745428	0.000974	0.002848
Snx1	0.201207	1.714738	5.367081	1.54E-05	6.83E-05
Rnpepl1	0.201043	1.20566	3.616707	0.001346	0.003786
Rnasek	0.200681	2.822802	3.788125	0.000874	0.002601
Stx4a	0.200546	2.266693	5.147565	2.69E-05	0.000113
Gcsam	0.200541	0.876156	2.786776	0.010122	0.022676
Fgfr1op2	0.200461	1.973207	4.364742	0.000201	0.00069
Tbc1d9b	0.200459	0.809979	4.789631	6.74E-05	0.000259

Plekhg1	0.200403	0.504491	3.275856	0.003137	0.008078
Zfyve1	0.200391	1.011164	5.021558	3.72E-05	0.000152
Bcap31	0.200374	1.983719	3.844063	0.000759	0.002298
Stx8	0.200243	1.304902	3.786916	0.000877	0.002605
Irak3	0.199978	0.421681	2.602713	0.015463	0.032651
Kctd9	0.199967	0.929731	4.567343	0.000119	0.000433
Kctd14	0.199956	0.639263	3.548567	0.001596	0.004409
Rtcb	0.199864	2.264264	4.929387	4.71E-05	0.000188
Jkamp	0.19962	0.749943	5.005228	3.88E-05	0.000157
Vps28	0.199506	1.928037	4.442846	0.000164	0.000579
Lpin1	0.199348	0.652223	3.614952	0.001352	0.0038
LOC10263(	0.199304	1.383705	3.131195	0.004462	0.011074
Hdac3	0.199041	1.051535	4.229346	0.000284	0.000949
Fam160b1	0.198948	1.094627	4.851558	5.75E-05	0.000225
Trim30a	0.198865	1.772435	2.960646	0.00672	0.015904
2210016L2	0.198798	1.156005	3.979902	0.000537	0.001677
Vmn1r127	0.198759	1.214408	2.789406	0.01006	0.022554
Bsdc1	0.198704	1.86322	4.331423	0.000219	0.000747
N6amt2	0.198701	0.95961	2.841808	0.0089	0.020294
Rab6a	0.198632	1.334065	3.554685	0.001572	0.004355
Commd3	0.198604	1.688549	4.078081	0.000418	0.001339
Sdhd	0.198568	1.178238	3.912704	0.000637	0.001969
Ninj1	0.198391	0.740954	2.729302	0.011567	0.025495
Ctr9	0.19836	0.805434	4.05731	0.000441	0.001403
Tmem214	0.198311	1.406682	5.636388	7.78E-06	3.66E-05
Kynu	0.198048	2.009968	4.690946	8.68E-05	0.000326
LOC10263:	0.198037	0.960416	2.407843	0.023915	0.048016
H2-Q4	0.197842	1.351461	3.758547	0.000942	0.002767
Sos2	0.196932	1.263366	5.531323	1.01E-05	4.65E-05
Srgap3	0.196798	0.829035	3.251502	0.003329	0.008518
Dr1	0.196773	1.780069	3.17936	0.00397	0.009983
Papd7	0.196759	0.744868	4.238588	0.000277	0.00093
Pla2g16	0.196722	1.713377	4.287911	0.000244	0.000828
Ext1	0.19671	1.154603	4.206628	0.000301	0.000998
Tango2	0.19657	0.88062	3.060029	0.005298	0.012917
Sec61a1	0.196546	2.068174	4.168603	0.000332	0.001087
Rela	0.196538	2.097425	4.190896	0.000313	0.001035
Elp5	0.195877	1.41031	3.861306	0.000726	0.002216
Ube2j1	0.195687	2.315401	4.683112	8.86E-05	0.000332
Zfp189	0.195644	0.829818	3.072433	0.005142	0.012578
Stx6	0.195462	1.159792	3.73401	0.001002	0.002926
Gm561	0.195235	1.100348	2.839981	0.008938	0.020358
Slc44a1	0.195205	1.788574	4.133715	0.000363	0.001182
Cd40	0.194969	1.621733	3.994955	0.000517	0.001621
Ddb1	0.194779	1.727257	4.731882	7.82E-05	0.000295
Iqcb1	0.194513	1.024727	4.441762	0.000165	0.000579
Pus10	0.19431	1.491546	3.75584	0.000948	0.00278
Hrsp12	0.194296	0.648892	2.975588	0.006485	0.015436
Skp1a	0.194288	2.008595	4.253956	0.000267	0.000896
Hbp1	0.194264	1.595705	4.664111	9.30E-05	0.000347
Arrdc3	0.194252	1.41729	3.338202	0.002691	0.007052
Txndc15	0.194105	1.3684	4.213186	0.000296	0.000984

Gpr89	0.194102	0.721555	3.794132	0.000861	0.002567
Dusp5	0.193944	1.093083	2.647888	0.01395	0.029905
Fto	0.193603	0.874683	3.288755	0.003039	0.007862
Gid4	0.193552	0.907606	3.407473	0.002268	0.006043
Otud1	0.193497	1.03646	2.96978	0.006576	0.015607
Inpp4a	0.193408	1.238269	4.852163	5.74E-05	0.000225
Gspt1	0.193279	1.299546	2.553725	0.017277	0.03605
Cnn2	0.193225	2.35484	3.206728	0.003714	0.009406
Sec11a	0.193008	1.957451	3.443518	0.002074	0.005579
Zfp318	0.192814	1.53851	4.565798	0.00012	0.000435
Uqcrc1	0.192615	1.550448	3.905071	0.00065	0.002005
Rprd1a	0.192558	0.783487	3.793388	0.000862	0.00257
Ssbp4	0.192557	0.648394	2.549957	0.017424	0.036266
Enox2	0.192525	1.420114	2.529993	0.018225	0.037785
Ept1	0.192452	1.196212	3.372569	0.002472	0.006544
Atxn7l3b	0.192417	1.739633	3.246392	0.003371	0.008614
Pqlc3	0.192349	1.490609	4.07246	0.000424	0.001356
Ly86	0.192312	2.247931	4.641978	9.85E-05	0.000365
Mllt6	0.192287	1.088413	3.44855	0.002048	0.005519
Dctn2	0.192105	1.55831	4.124763	0.000371	0.001205
Itfg1	0.191945	1.894229	4.617351	0.000105	0.000385
Wdr53	0.19192	1.17602	2.85334	0.008662	0.019825
Ing4	0.191684	1.429719	4.068033	0.000429	0.001369
Hagh	0.191626	0.908819	3.451529	0.002033	0.005487
Dnajc15	0.191584	2.324815	2.957996	0.006763	0.015974
Gapt	0.191153	0.406991	3.177072	0.003992	0.010032
Decr2	0.191129	1.001691	3.080814	0.00504	0.012355
1700008J0	0.190989	0.752487	3.365758	0.002514	0.006647
H2-Q6	0.190945	2.882492	4.155757	0.000343	0.001119
Orai2	0.190793	1.01413	3.805892	0.000836	0.002509
Ric1	0.190614	1.666464	3.348953	0.002621	0.00689
Gm13157	0.190148	1.474766	2.633365	0.014421	0.030745
Praf2	0.18981	1.357037	3.529291	0.001675	0.004605
Rcor3	0.189551	0.88826	3.654259	0.001225	0.003497
Hlx	0.189221	1.237065	2.710729	0.012075	0.026411
Gmppa	0.18901	1.270203	4.554181	0.000123	0.000447
Lepre1	0.18901	0.615955	3.062421	0.005268	0.012851
Clip2	0.188962	0.962247	4.997165	3.96E-05	0.00016
Eif4e3	0.188954	1.124648	3.329571	0.002749	0.007189
Ubxn6	0.188902	0.987285	4.159083	0.00034	0.001112
Csnk1g1	0.188898	1.388946	3.685043	0.001134	0.003263
Phf2	0.188664	1.284478	5.105785	3.00E-05	0.000124
Fam117b	0.188507	1.223291	4.574481	0.000117	0.000426
Chd4	0.188468	2.07411	5.344278	1.63E-05	7.18E-05
Galnt7	0.188275	1.877543	4.529026	0.000132	0.000474
Nck1	0.188274	1.187858	3.714058	0.001054	0.003054
Mrpl45	0.188179	0.778031	3.980905	0.000536	0.001674
Suds3	0.187937	1.755399	3.632371	0.001294	0.003657
Mkl1	0.187599	1.846086	5.291713	1.86E-05	8.10E-05
Mrfap1	0.187588	1.452575	3.031468	0.005674	0.013712
Prr12	0.18737	1.187074	3.611725	0.001363	0.003827
Prcp	0.187336	2.48134	4.123492	0.000372	0.001209

Slc50a1	0.187085	1.137021	3.766483	0.000923	0.002721
Rap2c	0.186916	2.17949	5.166365	2.57E-05	0.000108
Jmy	0.186681	0.919283	4.003428	0.000506	0.001589
Mtx1	0.186622	1.083634	4.012075	0.000495	0.001558
Tmc6	0.18645	0.593222	3.986001	0.000529	0.001655
Sik2	0.186446	1.47415	3.500298	0.001801	0.004904
Kdelr2	0.186391	2.250027	4.477389	0.00015	0.000534
Cnppd1	0.186374	1.135005	4.123237	0.000373	0.001209
Pi4ka	0.186248	1.518486	5.148875	2.68E-05	0.000113
Ints4	0.186068	1.390114	3.619059	0.001338	0.003768
Jak2	0.185928	2.89942	3.17485	0.004014	0.010079
Ubxn8	0.185888	0.898698	3.763002	0.000931	0.00274
2610021A(	0.185871	0.644561	4.236219	0.000279	0.000933
Socs4	0.185865	1.386771	4.331654	0.000218	0.000747
Gm10051	0.185847	2.970564	3.463362	0.001974	0.005341
Foxn2	0.185682	1.724581	4.0579	0.00044	0.001402
Ypel5	0.185644	1.73461	4.10912	0.000386	0.001251
Sike1	0.185451	1.008407	3.492952	0.001834	0.004982
Zfp131	0.185347	1.444349	4.57825	0.000116	0.000422
Prorsd1	0.185301	1.010272	3.090198	0.004927	0.012119
Snora78	0.185137	0.836762	2.974959	0.006495	0.01545
Arl3	0.18513	1.838734	3.519985	0.001715	0.004695
Sys1	0.185085	1.271518	4.399312	0.000184	0.000639
Sdhc	0.185017	1.551118	3.681783	0.001143	0.003287
Nfkb2	0.184805	1.948734	4.197132	0.000308	0.00102
Cd48	0.184799	2.044309	3.760491	0.000937	0.002756
Slk	0.184615	1.820036	4.931324	4.68E-05	0.000187
1810058I2	0.184492	1.317167	3.185541	0.003911	0.009844
Nlk	0.184457	1.577387	3.347219	0.002632	0.006912
Ero1lb	0.184368	1.709246	3.588782	0.001444	0.00403
Cptp	0.184329	0.947525	4.252274	0.000268	0.0009
Atp6v1g1	0.184312	2.41364	3.646495	0.001249	0.003552
Dync1li1	0.184125	1.731299	5.423477	1.33E-05	5.98E-05
Eif2b1	0.183918	0.738538	3.331644	0.002735	0.007157
Tbc1d1	0.183884	1.553891	4.365548	0.0002	0.000689
Mcee	0.183784	0.813123	3.035694	0.005617	0.013603
Aff1	0.183501	1.677292	4.59103	0.000112	0.00041
Trappc5	0.183467	1.025703	2.737046	0.011362	0.025102
Rtca	0.18329	1.412787	3.87827	0.000696	0.002133
4921524J1	0.183215	0.988225	4.274231	0.000253	0.000856
Edem1	0.183147	1.603708	4.426411	0.000171	0.0006
Etfdh	0.182627	1.482105	3.523379	0.0017	0.004664
Cdk2ap2	0.182474	1.134133	3.58981	0.00144	0.004023
Bloc1s1	0.182451	2.349461	3.270195	0.00318	0.008177
Ecd	0.182437	0.931152	4.782521	6.86E-05	0.000263
Cyfip1	0.182284	1.636505	4.379552	0.000193	0.000668
Sec24c	0.182098	1.649369	4.516913	0.000136	0.000487
Zmym6	0.182065	0.936867	4.208582	0.0003	0.000994
Mrpl34	0.181922	1.349414	2.999305	0.006128	0.014681
Ap1g2	0.181755	1.27049	3.697697	0.001098	0.00317
Gclc	0.181652	1.660919	3.796732	0.000855	0.002555
1500011K:	0.181259	1.377629	4.09089	0.000405	0.001302

Pdia6	0.180859	1.810147	2.67477	0.013117	0.02838
Mfn1	0.180695	1.287418	4.021025	0.000484	0.001527
Vps13a	0.180554	1.233192	4.133973	0.000363	0.001181
Higd2a	0.180137	2.101164	4.159336	0.00034	0.001112
Tlr5	0.180136	0.612007	3.189326	0.003875	0.009767
Stxbp5	0.180111	0.971646	3.370119	0.002487	0.006581
Usp18	0.18006	1.643399	3.690383	0.001119	0.003223
Ubn2	0.179836	1.516932	3.583678	0.001462	0.004075
Cnp	0.179655	0.868961	3.651129	0.001235	0.003521
Ift46	0.179425	0.962883	3.372611	0.002472	0.006544
Uqcc3	0.179259	1.33242	3.335848	0.002707	0.007086
Emc4	0.179204	1.49494	3.352978	0.002595	0.006824
Atp6v0e	0.179191	2.628121	3.425829	0.002167	0.005806
Ndufa6	0.179179	2.345699	4.554597	0.000123	0.000446
Tlr9	0.179155	1.914879	3.621255	0.001331	0.003752
E2f5	0.179127	0.833071	4.491634	0.000145	0.000517
Ift27	0.179102	0.57942	2.429124	0.022818	0.045963
Rnf38	0.17882	1.763469	4.306466	0.000233	0.000794
Blmh	0.178588	1.740046	4.422046	0.000173	0.000606
Mbd1	0.178581	0.958986	3.747135	0.000969	0.002838
Dhcr24	0.178126	0.791376	2.72086	0.011795	0.025921
Calcrl	0.178027	1.259034	3.315643	0.002845	0.007421
Bet1l	0.177965	1.028811	3.42061	0.002195	0.005873
Pdcl	0.177769	0.790871	3.662077	0.001201	0.003438
Dennd4a	0.177392	2.213657	4.100068	0.000395	0.001277
Mef2d	0.177244	1.607372	4.01065	0.000497	0.001563
Znf512b	0.176703	0.79602	2.734047	0.011441	0.025246
Plekha5	0.176488	1.149599	3.522989	0.001702	0.004665
Gm3837	0.176401	1.69173	3.581264	0.001471	0.004096
Tprkb	0.176251	0.84128	3.039647	0.005564	0.013498
Fam206a	0.176187	0.8338	2.698271	0.012426	0.027052
Hps4	0.176001	1.068204	4.552697	0.000124	0.000448
Akr1a1	0.175878	2.838463	4.53993	0.000128	0.000462
Serinc1	0.175767	1.625612	4.853714	5.72E-05	0.000225
Gskip	0.175406	1.001486	3.853601	0.00074	0.002253
Ndfip2	0.175187	1.071498	3.811285	0.000824	0.002479
Nt5c3	0.175162	0.925196	3.651163	0.001235	0.003521
Obfc1	0.175139	0.63573	3.962883	0.000561	0.001746
Mar-02	0.174964	1.406814	3.861442	0.000726	0.002216
G6pdx	0.174794	1.038022	2.742167	0.011228	0.024865
Uso1	0.174712	1.763474	3.87567	0.0007	0.002145
Eif2a	0.174523	1.780433	3.646544	0.001249	0.003552
Map2k2	0.174441	0.999404	3.529207	0.001676	0.004605
Rab35	0.174284	0.911463	2.975732	0.006483	0.015436
Fam179b	0.174156	1.250586	3.270991	0.003174	0.008164
Cdk17	0.173852	1.499639	2.539484	0.01784	0.0371
1700025G(	0.173549	1.645081	3.8418	0.000763	0.002308
2510039O:	0.173515	1.342787	3.771282	0.000912	0.002696
Dvl3	0.173277	0.965631	2.980114	0.006416	0.015295
Arhgap12	0.173138	1.176176	3.800574	0.000847	0.002535
Sppl3	0.173033	1.666954	3.510235	0.001757	0.0048
Marcks	0.172896	1.865841	3.400662	0.002306	0.006135

Brox	0.172843	1.079658	2.805445	0.009691	0.021811
Zfp182	0.17284	0.682706	3.197313	0.0038	0.009601
Kansl1l	0.172563	0.840191	3.993611	0.000519	0.001626
Rfk	0.172561	1.231734	3.508061	0.001766	0.004824
Fcho1	0.172345	0.86787	2.854217	0.008644	0.019797
Plekhn1	0.172262	1.496964	2.642563	0.014121	0.030236
Ubash3b	0.171959	1.104299	3.312079	0.00287	0.007479
Reps1	0.171911	1.582069	4.112192	0.000383	0.001241
Mrps15	0.171891	1.605981	4.171979	0.000329	0.001079
Dhx57	0.171825	1.69982	3.188651	0.003881	0.009776
Sqstm1	0.17174	1.996117	3.763231	0.000931	0.002739
Slc35a5	0.171396	1.135022	3.50573	0.001777	0.004847
Ndufc1	0.171344	2.054345	2.462498	0.021191	0.043072
Prdx3	0.171208	1.523226	3.39261	0.002353	0.006245
Leprotl1	0.171185	1.886966	3.045459	0.005487	0.013342
Scarna3a	0.171167	1.231046	2.856971	0.008588	0.01968
Tmem173	0.170919	1.656335	3.402409	0.002296	0.006112
Mta1	0.17078	1.596989	3.58505	0.001457	0.004062
Hint2	0.170725	1.847965	2.552601	0.017321	0.036121
Arhgap1	0.170708	1.564321	4.359006	0.000204	0.0007
Nbeal1	0.170673	1.162839	3.759086	0.000941	0.002764
Prdm2	0.170635	1.178534	3.639023	0.001273	0.003613
Mtmr12	0.17063	1.489804	3.664135	0.001195	0.003425
Lsm4	0.170428	1.438217	3.044286	0.005502	0.013371
Eif4e2	0.170009	1.377457	3.11858	0.004601	0.011391
Ndst2	0.169995	1.25811	3.473412	0.001925	0.005215
Ptk2	0.169928	0.824799	3.647562	0.001246	0.003546
Ncdn	0.169888	1.070191	3.757748	0.000944	0.002769
Cd37	0.169817	1.613486	2.605352	0.015371	0.032501
Vdac1	0.169795	2.042824	3.615928	0.001349	0.003792
Bbip1	0.169793	0.990201	3.707114	0.001072	0.003099
Amz2	0.169739	1.007435	3.801401	0.000845	0.002532
Ap2b1	0.169659	1.981388	3.543377	0.001617	0.004463
Mfsd1	0.169541	1.930953	3.681037	0.001145	0.003292
Kdm5c	0.169522	1.523639	4.470039	0.000153	0.000542
Trak2	0.169398	0.834799	4.317644	0.000226	0.000773
Mrpl43	0.169262	1.030479	3.264967	0.003221	0.008268
Snora33	0.169125	1.143877	2.56487	0.016848	0.035245
Camsap2	0.169034	1.072053	3.231359	0.003497	0.008903
Ppa2	0.168953	1.175362	3.454976	0.002016	0.005446
Zfp518a	0.168929	1.357955	3.360737	0.002545	0.006721
Fam92a	0.168913	0.726833	2.502176	0.019397	0.039806
Mrpl28	0.168839	0.85013	2.934498	0.007151	0.016764
Rufy1	0.168366	1.36349	3.121187	0.004572	0.011327
Slc22a21	0.168125	0.577002	3.289424	0.003034	0.007852
Samd4b	0.168065	1.252021	2.669533	0.013275	0.028681
Med11	0.168048	1.527652	3.458036	0.002	0.005406
Pigt	0.167739	1.38247	2.894806	0.007856	0.018182
Rnf141	0.167699	1.065889	2.732228	0.011489	0.025345
Pccb	0.167698	0.643793	2.401192	0.024267	0.048645
Anapc5	0.167647	2.031001	3.426038	0.002166	0.005805
Uba5	0.16762	1.485547	4.447956	0.000162	0.000571

Tmem234	0.167602	2.164765	3.421568	0.00219	0.005861
Zfand6	0.167429	2.132813	3.585492	0.001456	0.004061
Krit1	0.167412	1.376624	4.288801	0.000244	0.000827
Cebpg	0.16741	1.528869	3.324993	0.00278	0.00726
Fam96a	0.167362	2.006972	3.711025	0.001062	0.003075
Icam1	0.167348	2.031543	2.967167	0.006617	0.015699
Nol6	0.167078	0.711298	2.894778	0.007856	0.018182
Stk25	0.166893	1.381618	3.065811	0.005225	0.012767
AW04620C	0.166804	0.613627	2.453292	0.021629	0.043841
Eif2b5	0.16663	0.902115	3.832959	0.00078	0.002355
Ptms	0.166426	2.085799	3.60502	0.001386	0.003884
Prr5l	0.166407	0.956745	3.787186	0.000876	0.002604
Akap9	0.166332	1.772348	3.970762	0.00055	0.001712
Crcp	0.166331	1.296473	3.791399	0.000867	0.002581
Scd2	0.16627	1.209152	2.389934	0.024875	0.049609
Ufc1	0.16612	1.717302	3.526889	0.001685	0.00463
Coprs	0.165926	1.040445	2.60917	0.015238	0.032257
Chmp1b	0.165875	2.056571	3.242002	0.003408	0.008695
Apip	0.165822	1.355565	3.448592	0.002048	0.005519
Tmem165	0.165494	1.440508	3.534077	0.001655	0.004558
Tada1	0.165313	1.290106	2.965895	0.006637	0.015737
Derl2	0.165293	1.739996	2.700244	0.01237	0.026945
Sep-02	0.165192	1.653581	3.646269	0.00125	0.003553
Acsl4	0.16507	1.538383	3.887261	0.00068	0.002089
Ppp2r1b	0.164859	1.147105	3.742502	0.000981	0.002868
Pvrl1	0.164771	1.426106	3.51066	0.001755	0.004797
Aga	0.164762	1.444325	3.032356	0.005662	0.013688
Nat6	0.164742	1.121386	3.281816	0.003091	0.00798
Paf1	0.164592	1.601178	3.911662	0.000639	0.001974
Slc7a5	0.164487	1.364209	2.663151	0.013471	0.028995
Fndc3a	0.164475	1.557041	3.666235	0.001189	0.003408
Vimp	0.164472	1.880484	3.361007	0.002544	0.006719
Ccdc6	0.164466	1.243468	3.984838	0.00053	0.001659
Phf10	0.164385	1.649918	3.082175	0.005023	0.012323
Acd	0.164324	1.037731	2.48128	0.020324	0.041465
Bcas3	0.164319	0.897836	3.726805	0.00102	0.002973
9130023H:	0.164306	0.699316	2.942446	0.007017	0.016497
Zfp456	0.164161	0.676373	2.828613	0.009179	0.020798
Polr1c	0.164096	1.716141	3.35454	0.002585	0.0068
Tmem57	0.164044	1.125988	3.768372	0.000919	0.00271
Hcst	0.163818	1.512635	2.897069	0.007814	0.018117
Nup98	0.163511	1.99735	4.127231	0.000369	0.001198
Capns1	0.163309	2.417333	3.02318	0.005788	0.013965
Vps8	0.16283	0.696106	4.367553	0.000199	0.000686
Abcc1	0.162604	1.429619	3.72426	0.001027	0.002991
Cyc1	0.162567	1.442638	3.759119	0.000941	0.002764
Tmem251	0.16234	1.103616	2.930663	0.007216	0.016896
Mogs	0.162253	0.597429	2.673839	0.013145	0.028424
Nploc4	0.162098	1.354834	3.205782	0.003723	0.009424
Mtmr1	0.162046	1.733604	4.536963	0.000129	0.000465
Tmem128	0.162024	1.484492	2.958562	0.006754	0.015958
Cd47	0.161933	2.840168	5.28857	1.88E-05	8.16E-05

Plbd2	0.16178	1.021208	2.829896	0.009152	0.020748
Pcmtd1	0.161713	1.377963	4.530502	0.000131	0.000472
Snord95	0.16165	0.777348	2.462743	0.02118	0.04306
Erlin2	0.161473	1.058279	3.259923	0.003261	0.008362
LOC10263(	0.161351	1.163472	2.521843	0.018561	0.038289
Zc3hc1	0.161321	0.691405	3.595348	0.00142	0.003972
Mrpl39	0.161306	1.566467	3.47306	0.001927	0.005218
Msmo1	0.161157	1.188796	3.113011	0.004663	0.01153
Tmem106a	0.161115	1.394302	2.769641	0.010534	0.023484
Wrn	0.161066	1.585454	4.463752	0.000156	0.000551
Cfap20	0.160785	0.915729	2.87484	0.008234	0.018928
Psmd7	0.160667	1.973425	3.167248	0.004089	0.010239
Mrpl52	0.160394	2.344441	3.042244	0.005529	0.013428
Ipo9	0.160323	0.93703	2.770925	0.010503	0.023433
Nsfl1c	0.160061	1.921882	3.916832	0.000631	0.00195
Emp3	0.159804	2.181627	2.626181	0.014659	0.031182
Git1	0.159675	1.154516	3.226858	0.003536	0.008998
Mrps35	0.159627	1.241349	3.225463	0.003548	0.009022
Arl2bp	0.159456	1.136968	2.710162	0.01209	0.026436
Rragd	0.159208	0.263365	3.013385	0.005925	0.014265
Crkl	0.159084	1.194067	2.961783	0.006702	0.015866
Rgs10	0.159066	1.462876	3.521815	0.001707	0.004677
Sdhaf2	0.158965	1.240313	2.975073	0.006493	0.01545
Ndufb6	0.158861	2.119645	2.668103	0.013319	0.028734
Egln1	0.15884	1.233132	2.99233	0.006231	0.014899
Rps26	0.158654	1.929174	2.95926	0.006742	0.015936
Chp1	0.15863	1.063108	2.949219	0.006905	0.016265
Fastk	0.158609	1.0433	2.925801	0.0073	0.01707
Apool	0.158539	1.549605	2.679097	0.012987	0.028134
Rfxank	0.158493	1.076717	3.035354	0.005622	0.013607
Zfp609	0.158425	0.975146	2.97088	0.006558	0.015576
Zfp141	0.158416	0.991959	2.437737	0.022388	0.045219
Rabgap1	0.158273	1.547315	4.212489	0.000297	0.000985
Churc1	0.158166	1.677011	2.657671	0.013641	0.029276
Sec63	0.158151	1.645685	3.769	0.000917	0.002707
A930002C(	0.158132	0.474416	3.652943	0.001229	0.003507
Aco1	0.158127	1.296552	3.18989	0.00387	0.009757
Ptpn2	0.158033	1.689747	3.637344	0.001278	0.003625
1110059G:	0.158014	1.076402	2.686114	0.012779	0.027746
Sdf4	0.157977	1.678577	3.013284	0.005927	0.014265
Pank1	0.157786	0.644621	3.17885	0.003975	0.009992
Dtnbp1	0.157525	1.135281	2.947716	0.00693	0.016318
Creld2	0.157424	1.271825	3.105285	0.004751	0.011724
Lyrm2	0.157307	1.263077	2.904405	0.007679	0.017856
Hn1l	0.157236	1.24775	3.193782	0.003833	0.009678
Csrnp1	0.157213	1.463444	3.136791	0.004402	0.01094
Nomo1	0.157018	0.828328	2.951255	0.006872	0.016207
Cnot4	0.156944	1.682986	4.08366	0.000412	0.001323
Reep4	0.156478	1.169174	3.516723	0.001729	0.004732
1110059E:	0.156401	1.546158	2.963886	0.006669	0.015802
Snx8	0.156306	1.1514	3.05975	0.005302	0.012921
Msantd4	0.156212	1.149551	3.00114	0.006102	0.014631

Pdcd2	0.156125	1.430061	3.310216	0.002883	0.007505
Vti1b	0.155917	2.042189	4.377758	0.000194	0.00067
Tmem222	0.15587	1.254632	2.713981	0.011984	0.026275
Mbtps2	0.155778	0.967025	3.348822	0.002621	0.00689
Zfp36l1	0.155693	3.071432	3.777174	0.000899	0.00266
Rnf187	0.155661	1.576662	3.687584	0.001126	0.003245
Cpeb4	0.155601	1.571828	3.766421	0.000923	0.002721
Zfyve16	0.155434	0.9253	3.905408	0.000649	0.002004
Apex1	0.155413	1.12994	3.781863	0.000888	0.002634
Tex261	0.155373	1.420344	3.052475	0.005395	0.013132
Chtf8	0.155269	2.091374	3.234624	0.00347	0.008838
Rpl36al	0.155247	1.970707	2.718952	0.011848	0.026012
Mib1	0.155153	1.199051	4.091863	0.000404	0.0013
Wbp1	0.155125	1.16557	2.669009	0.013291	0.028691
Mrpl54	0.154523	1.743277	2.725792	0.011662	0.025672
Naa38	0.15437	1.829625	3.021365	0.005813	0.014016
Afmid	0.15421	0.81034	2.528953	0.018267	0.037841
Eefsec	0.154152	1.034222	2.669021	0.013291	0.028691
Carm1	0.154115	0.936673	2.961799	0.006702	0.015866
Ddx1	0.154048	1.228089	2.817608	0.009419	0.021263
Vps26a	0.154042	1.730236	3.087851	0.004955	0.012179
Klf4	0.154032	1.274395	3.429652	0.002146	0.005759
Wars	0.153791	1.463046	3.162684	0.004134	0.01034
Ncstn	0.153739	1.682203	3.667097	0.001186	0.003403
Snf8	0.153648	1.679775	3.619536	0.001337	0.003767
Ice1	0.153596	1.14142	3.174004	0.004022	0.01009
Gtf3a	0.153513	1.465944	3.239833	0.003426	0.008735
Ankrd13a	0.153453	2.140389	3.462274	0.001979	0.005354
Tmem126b	0.153412	1.103948	2.6207	0.014843	0.031547
Cmc1	0.153406	1.320524	2.749447	0.01104	0.024492
Ehmt1	0.153399	1.503373	3.7334	0.001004	0.002929
Zmym2	0.153338	2.047958	4.147856	0.00035	0.001142
Smarcb1	0.15304	1.433463	3.21714	0.003621	0.009195
Cox6b1	0.152702	2.281618	2.6233	0.014756	0.031379
Dstyk	0.152527	0.817984	3.181664	0.003948	0.00993
Rab31	0.152487	0.885197	2.457692	0.021419	0.043475
Stk4	0.152277	2.572667	3.69403	0.001108	0.003195
Fyttd1	0.152235	1.609279	3.417679	0.002211	0.005907
Smdt1	0.152185	2.531679	3.45414	0.00202	0.005453
Usp40	0.151892	1.117125	3.796178	0.000856	0.002558
Rab8b	0.151831	2.622985	3.986828	0.000528	0.001652
Mtmr14	0.151699	1.627155	2.984658	0.006347	0.01514
2810428I1	0.151597	1.577994	3.213995	0.003649	0.009253
Ncor2	0.151573	1.409051	3.816392	0.000814	0.00245
Manbal	0.151499	0.927827	2.757159	0.010844	0.02413
Timm8b	0.151487	2.125251	3.990779	0.000523	0.001636
Ppp2r1a	0.151414	1.759129	3.141818	0.004349	0.010822
Upf1	0.15131	0.80059	3.537776	0.00164	0.004519
Hsp90b1	0.151191	3.248005	2.986551	0.006318	0.015086
Mut	0.151183	0.837076	3.360248	0.002549	0.006727
Dync1i2	0.150932	1.595303	3.737855	0.000992	0.002901
Pex13	0.150864	1.138727	2.87501	0.008231	0.018926

Ap5m1	0.150809	0.82898	3.256871	0.003286	0.008422
Clcn7	0.150268	0.717553	2.661427	0.013524	0.029084
Txnl4b	0.150155	1.20969	2.61022	0.015202	0.032201
Sik3	0.150155	1.059388	2.830343	0.009142	0.020733
Rpn2	0.150065	1.583641	3.710158	0.001064	0.003081
Map4	0.15002	1.780555	3.633745	0.00129	0.003646
Map4k2	0.149913	0.950577	2.99412	0.006205	0.01484
Pdcl3	0.149669	1.835745	2.950773	0.00688	0.01622
Aebp2	0.149597	2.215095	3.199822	0.003777	0.009546
Cep192	0.149416	1.450125	3.780641	0.000891	0.00264
Nup93	0.149376	1.421509	4.256059	0.000265	0.000893
Rpn1	0.149367	1.226979	3.521717	0.001707	0.004677
Ube2d1	0.148868	2.065064	3.296833	0.002979	0.007723
Hadha	0.14879	1.855081	4.027481	0.000476	0.001506
Rnf11	0.148663	1.237916	2.641949	0.014141	0.030266
Rpp25l	0.148642	0.855844	2.713837	0.011988	0.026275
Magt1	0.148637	2.040492	3.632044	0.001295	0.003658
Bscl2	0.148575	1.126266	3.013983	0.005917	0.014253
Ticam1	0.148465	1.103194	2.666949	0.013354	0.028793
Purg	0.148428	1.002506	3.26484	0.003222	0.008268
Ubl7	0.148324	1.559895	3.284777	0.003069	0.007931
Zfp598	0.148278	1.020489	3.028843	0.00571	0.01379
Gars	0.148217	1.861321	3.442228	0.002081	0.005592
Specc1l	0.148145	1.15863	4.127288	0.000369	0.001198
Ndufa9	0.147961	1.766979	4.261534	0.000262	0.000882
Ralbp1	0.147928	1.220626	3.271472	0.00317	0.00816
Stxbp3a	0.147903	2.16013	2.706995	0.012179	0.026599
Tsc22d1	0.147584	1.208698	3.412977	0.002237	0.00597
Cpsf3l	0.147481	0.98488	3.329855	0.002747	0.007186
Pank3	0.147469	1.048863	2.698579	0.012418	0.027041
Eif2ak1	0.14736	1.345932	2.86016	0.008524	0.019545
Otud5	0.147149	1.838766	3.219324	0.003602	0.009152
Usp24	0.147064	1.417341	3.787272	0.000876	0.002604
Gtf2a1	0.146865	2.22037	3.884947	0.000684	0.002101
Ctsh	0.146823	2.693153	3.720658	0.001036	0.003013
Lrrc41	0.146688	1.393465	2.611848	0.015145	0.032098
Inafm2	0.146578	1.053445	2.89355	0.007879	0.018229
Ddx10	0.14653	0.979687	2.750945	0.011002	0.024429
Foxp1	0.146492	1.362213	2.456883	0.021457	0.043541
Snrk	0.146489	1.051159	3.113345	0.004659	0.011525
Gnptg	0.14646	1.116396	2.914572	0.007497	0.017486
Lrrc58	0.146332	1.410933	2.966245	0.006631	0.015729
Fbxl20	0.146279	1.361735	2.524895	0.018435	0.038113
Arl6	0.146148	0.718275	2.739988	0.011285	0.024968
Cuedc2	0.145902	0.938148	2.941675	0.00703	0.016517
Snhg7	0.14586	0.508071	2.735196	0.011411	0.025202
Ddx17	0.145856	2.441807	4.101006	0.000394	0.001275
Acadsb	0.145723	0.9163	2.978375	0.006442	0.015349
Tpd52l2	0.145719	1.487425	2.951676	0.006865	0.016196
Ube2z	0.145671	1.558324	3.139343	0.004375	0.010883
Degs1	0.145526	1.947351	2.431396	0.022704	0.045783
Taf11	0.145479	0.803313	2.786315	0.010133	0.02269

Nsmce1	0.145425	1.464951	2.737047	0.011362	0.025102
Dis3l2	0.145386	0.890129	2.476208	0.020555	0.041904
Tnpo1	0.145066	1.905764	3.126954	0.004508	0.011184
Kcnk6	0.144843	1.567368	3.062899	0.005262	0.012845
Mrpl37	0.144329	1.078264	2.663862	0.013449	0.028956
Rp9	0.143886	1.316586	3.15524	0.00421	0.010511
Cdkal1	0.143791	0.848408	2.500919	0.019452	0.039874
Sri	0.143684	2.812082	3.552803	0.00158	0.004371
Bcat2	0.143613	1.068262	3.023793	0.00578	0.013949
Spcs1	0.143601	1.666906	2.6653	0.013405	0.028877
Golga3	0.143484	1.032541	2.685478	0.012798	0.027773
Gmps	0.143159	1.98359	3.676407	0.001159	0.003327
Tspyl2	0.143156	1.197384	3.281392	0.003094	0.007986
Acbd6	0.142869	1.342808	3.067988	0.005198	0.012705
Zc3h14	0.142595	1.579718	2.933736	0.007164	0.016789
Pecam1	0.142463	0.798732	2.531236	0.018174	0.03769
Pcmtd2	0.14246	0.906732	3.388126	0.002379	0.00631
Fam162a	0.142398	1.329644	2.395464	0.024575	0.049158
Ppm1m	0.142319	1.405831	2.887449	0.007993	0.018465
Maf1	0.142238	0.9742	2.490863	0.019894	0.040701
Zfp644	0.14167	1.194087	3.297485	0.002974	0.007717
Crbn	0.141411	1.30267	3.395937	0.002333	0.006196
Clns1a	0.141409	1.533526	2.389047	0.024923	0.049676
Pdpr	0.141399	0.931871	3.402852	0.002294	0.006108
Vps26b	0.141366	1.050419	3.000444	0.006112	0.01465
Iah1	0.141316	1.68969	3.037863	0.005588	0.013542
Hars	0.141197	1.382085	3.131696	0.004457	0.011068
Tbcel	0.141032	0.686837	2.823962	0.00928	0.020994
Zhx1	0.140964	1.542087	2.584851	0.016103	0.033886
Mapkap1	0.140851	1.732805	3.504975	0.00178	0.004854
Surf1	0.140678	1.06709	2.610167	0.015203	0.032201
Scamp3	0.140397	1.144729	2.994177	0.006204	0.01484
Polr3b	0.140343	1.111546	3.00413	0.006058	0.01455
Sin3b	0.140301	1.324854	3.035626	0.005618	0.013603
Trnau1ap	0.140199	1.033313	2.524953	0.018432	0.038113
Tecr	0.140113	2.229805	3.145348	0.004312	0.010744
Gm13363	0.139941	0.911438	2.491064	0.019885	0.040694
Arl4a	0.139561	1.508986	2.669126	0.013288	0.028691
Prpf19	0.139474	0.826833	3.013205	0.005928	0.014265
B3gnt5	0.139472	0.616968	2.416181	0.023479	0.047193
Mdm2	0.139403	1.643071	3.560416	0.00155	0.004301
Atf7ip	0.139379	1.760896	3.587708	0.001448	0.00404
Stat5b	0.13935	1.244905	2.728539	0.011588	0.025517
Pigk	0.13933	0.929509	2.734309	0.011434	0.025239
Ccdc25	0.13931	1.436549	2.632159	0.01446	0.030804
Ctdsp2	0.139043	1.00613	2.553272	0.017294	0.036077
Smim14	0.138801	1.352687	2.712692	0.01202	0.026329
Stat3	0.138789	2.406704	3.449489	0.002043	0.00551
Dicer1	0.138735	1.337239	2.704439	0.012251	0.026719
Polr2m	0.138688	1.702938	3.86324	0.000723	0.002208
Ndufv3	0.138625	1.628214	3.211748	0.003669	0.009298
Bicd2	0.138442	0.745251	2.531711	0.018154	0.03767

Tmed8	0.138325	0.814377	2.390889	0.024823	0.049532
Tbc1d14	0.138078	1.261285	2.951666	0.006865	0.016196
Sgta	0.137946	0.797471	2.896692	0.007821	0.018128
Manea	0.137911	0.779832	2.481178	0.020329	0.041465
Mettl14	0.137907	1.289246	3.174425	0.004018	0.010083
Fnta	0.137822	1.279333	3.328164	0.002758	0.007209
Vps4a	0.137816	1.182253	2.850322	0.008723	0.019935
Smim20	0.137783	1.027414	2.619253	0.014892	0.031624
Prdx4	0.137562	0.642371	2.438954	0.022327	0.045109
Phka2	0.137528	1.411037	3.341436	0.00267	0.007001
Rnaseh2b	0.137485	1.440431	2.719863	0.011823	0.025973
Sugt1	0.137383	1.551715	2.524008	0.018471	0.038158
Elf2	0.13731	1.364065	3.398317	0.00232	0.006164
Ptdss2	0.137297	0.675592	2.854203	0.008644	0.019797
Sdhb	0.137229	1.68828	3.219161	0.003603	0.009153
Ece1	0.13711	2.122102	2.795264	0.009924	0.022288
Ube2w	0.137071	0.946114	2.801175	0.009788	0.021996
Gmfb	0.136999	1.885713	3.054939	0.005363	0.013059
Ercc5	0.136725	1.04274	2.85817	0.008564	0.019631
Dusp11	0.13664	2.096247	2.722554	0.011749	0.02585
Prr33	0.136625	0.969998	2.9068	0.007636	0.017766
Amfr	0.136547	2.076259	3.287433	0.003049	0.007885
Npepps	0.136398	1.773204	3.276264	0.003133	0.008075
Ywhae	0.136188	2.939879	3.414693	0.002228	0.005947
Pacs2	0.136171	1.401255	2.91563	0.007478	0.017448
Phpt1	0.136156	1.543775	2.636298	0.014324	0.030576
Leprel1	0.136144	1.084241	2.926985	0.00728	0.017028
Arl6ip4	0.136141	1.783672	2.937494	0.0071	0.016657
Elf1	0.136107	2.23704	3.599244	0.001406	0.003938
Tmem30a	0.136022	1.641914	2.505001	0.019275	0.039594
Slc25a38	0.135929	0.915765	3.0964	0.004854	0.011966
0610007P:	0.135555	1.799114	2.939278	0.00707	0.016601
Scyl2	0.135506	1.421489	3.47851	0.001901	0.005153
Jak3	0.135329	0.682433	2.666068	0.013381	0.028843
Sec23b	0.135031	1.147085	3.076028	0.005098	0.012474
Fbxl3	0.134956	1.29406	2.900045	0.007759	0.017996
Med16	0.134939	1.001987	2.985011	0.006341	0.015134
Ccdc53	0.134924	1.347308	2.877234	0.008188	0.018845
Grcc10	0.134871	2.599212	3.491855	0.001839	0.004992
Setdb1	0.134804	1.2851	3.058365	0.005319	0.012956
Ktn1	0.134573	1.864168	3.098668	0.004827	0.011905
Exosc3	0.134314	1.9686	2.479163	0.02042	0.04164
Psmd2	0.134308	1.800098	2.737092	0.011361	0.025102
AI662270	0.134189	2.076863	2.636538	0.014317	0.030568
Acat1	0.134183	1.021413	2.39051	0.024843	0.04956
Strn3	0.134014	1.272829	3.435479	0.002116	0.005681
Extl3	0.133847	0.947564	2.790723	0.010029	0.022505
Hp1bp3	0.133812	1.496934	3.633723	0.00129	0.003646
Myh9	0.133749	2.417206	3.386693	0.002387	0.00633
Prmt7	0.133453	0.666286	2.895455	0.007843	0.018164
Xpa	0.133369	1.586339	2.511449	0.018999	0.039096
Sp3	0.133145	1.123224	3.246921	0.003367	0.008609

Ndufs2	0.133063	2.045838	3.497133	0.001815	0.004934
Yy1	0.133054	1.705175	3.343405	0.002657	0.00697
1110004F1	0.132977	1.330763	2.550407	0.017406	0.03625
Fkbp8	0.132957	1.155323	2.883377	0.00807	0.01862
Nmt1	0.132931	2.150104	2.886715	0.008007	0.018491
Noc3l	0.132912	0.760017	3.126626	0.004512	0.011186
Fgd6	0.132795	0.570062	3.297462	0.002975	0.007717
Ndufb5	0.132743	2.123099	3.041529	0.005539	0.013446
Dnajc1	0.132492	1.536435	3.018895	0.005848	0.014095
Ddx49	0.13245	1.086586	3.103869	0.004767	0.01176
Cers5	0.132072	1.217681	2.508955	0.019105	0.039282
Slc9a7	0.131702	1.729369	2.933355	0.00717	0.016799
Chchd3	0.131664	1.562091	2.994549	0.006198	0.014834
Eaf1	0.13164	1.140278	2.485138	0.02015	0.041179
Zmym5	0.131179	1.707504	3.038741	0.005576	0.013519
Stt3a	0.130915	2.150918	3.427961	0.002155	0.005781
Fam188a	0.130891	1.61374	3.302545	0.002938	0.00764
Abi2	0.130886	0.943682	3.311384	0.002875	0.007489
Exo5	0.130725	1.004212	2.394461	0.024629	0.049238
Tdh	0.13071	0.353938	2.676154	0.013075	0.028298
Psmb8	0.130603	2.744153	2.701168	0.012344	0.026903
Proser1	0.130519	1.430889	2.953316	0.006838	0.016143
Tyw5	0.130496	0.827603	2.79036	0.010038	0.022517
Ddx23	0.130448	1.546645	3.523206	0.001701	0.004664
Phb2	0.130317	1.944377	2.728553	0.011587	0.025517
Ncbp2	0.13027	1.121596	3.190864	0.00386	0.009737
Atad2b	0.130108	1.795471	3.91872	0.000628	0.001942
Asun	0.130049	1.102353	2.61838	0.014922	0.031678
Adnp	0.130035	1.892344	2.613752	0.01508	0.031968
Isyna1	0.129473	0.7925	2.598801	0.015601	0.032905
Idua	0.129439	0.870721	2.581029	0.016243	0.034142
Selplg	0.129145	3.167586	3.005268	0.006042	0.014515
Vps52	0.128818	1.15531	2.973888	0.006512	0.015484
Adam10	0.128433	1.644628	2.57397	0.016504	0.034643
Drg2	0.12829	0.910235	2.554189	0.017259	0.036033
Lypla1	0.128229	1.707199	2.430846	0.022731	0.045826
Cog6	0.128169	0.713944	2.811915	0.009545	0.021516
Csnk1d	0.128118	1.824753	2.406596	0.02398	0.048135
Adipor2	0.128118	0.978689	2.436819	0.022433	0.045286
Bcl2l12	0.127825	0.851265	2.432571	0.022645	0.045676
Eps15l1	0.127618	1.636003	2.776738	0.010362	0.02316
Trak1	0.127466	0.976213	3.079473	0.005056	0.012387
Hdlbp	0.127453	2.19799	3.364873	0.00252	0.006657
Dnajc5	0.127361	1.81357	3.030375	0.005689	0.013744
Uhrf1bp1l	0.127301	1.379119	2.489748	0.019944	0.040791
Sar1a	0.12699	2.132934	3.383904	0.002404	0.00637
Ndufc2	0.126865	2.081198	2.593246	0.015799	0.033303
Cpne3	0.126656	1.903939	3.19199	0.00385	0.009713
Myo9a	0.126451	1.572624	2.816063	0.009453	0.021334
Cct4	0.126406	2.133703	3.138714	0.004382	0.010896
Zbed6	0.126385	1.755854	3.037768	0.005589	0.013542
Tubgcp2	0.126278	1.347443	2.562978	0.01692	0.035384

Nup88	0.126248	2.025428	2.441103	0.022221	0.044932
Rheb	0.12607	1.790776	2.660914	0.01354	0.02911
Tmem127	0.126063	1.390305	2.570196	0.016646	0.03489
Hdac5	0.126039	0.912024	2.552125	0.017339	0.036131
Nr3c1	0.126013	1.62188	2.973292	0.006521	0.015501
Dctn4	0.125772	1.986029	2.746274	0.011122	0.024651
Cog5	0.125747	1.487794	2.839234	0.008954	0.020368
Snd1	0.125507	1.611977	3.245232	0.003381	0.008635
Aars	0.125456	1.09452	2.515434	0.01883	0.03878
Ssu72	0.125392	2.216129	2.673333	0.01316	0.028449
Ofd1	0.125294	1.040393	2.581787	0.016215	0.034093
Sepw1	0.125241	2.652515	2.775832	0.010384	0.023195
Inf2	0.125223	0.932313	2.520421	0.018621	0.038391
Ubxn2a	0.125209	1.134539	2.557363	0.017136	0.035806
Pfdn6	0.125111	1.491201	2.784315	0.01018	0.022782
Ctsc	0.125067	1.729381	2.729599	0.011559	0.025485
Zdhhc13	0.125037	1.263015	2.697338	0.012453	0.027094
Atpif1	0.124961	2.555554	3.078007	0.005074	0.012423
Sgpl1	0.124724	1.817374	3.512852	0.001745	0.004772
Dnajc3	0.124313	2.243787	3.090439	0.004924	0.012116
Racgap1	0.123713	0.702344	2.39954	0.024355	0.048809
Mios	0.123656	1.062449	3.25198	0.003325	0.008511
Acbd3	0.123578	1.487146	2.608089	0.015275	0.032327
Mrpl57	0.123512	0.837909	2.629259	0.014556	0.030987
Ints3	0.123472	1.585119	3.41704	0.002215	0.005914
Cdk13	0.123344	1.926936	2.838298	0.008973	0.020399
5330426P1	0.123283	0.769978	2.534249	0.018051	0.037477
Micall1	0.123263	0.799704	2.640382	0.014191	0.030335
Trrap	0.122955	1.386934	3.554191	0.001574	0.004359
0610010F0	0.122931	0.871552	2.501159	0.019442	0.039872
Nfya	0.122791	1.655865	2.793798	0.009958	0.022358
Taz	0.122575	1.460834	2.696692	0.012472	0.027127
Stambp	0.122303	0.936284	2.882985	0.008078	0.018625
Aco2	0.12224	1.458904	3.419129	0.002203	0.00589
Apopt1	0.122099	1.294896	2.556432	0.017172	0.035861
Twsg1	0.122094	0.707834	2.387018	0.025034	0.049861
Rnf138	0.122034	0.985169	2.568576	0.016707	0.034989
Tcf25	0.121817	2.125152	3.034091	0.005639	0.01364
Shoc2	0.121592	1.573634	3.146737	0.004297	0.010712
Mapk3	0.120813	2.069072	3.214388	0.003645	0.009249
Setx	0.120413	1.398757	2.706274	0.012199	0.026628
Sec31a	0.120336	1.809989	2.8786	0.008162	0.018802
Bzw1	0.120232	2.206261	2.919983	0.007401	0.017281
Tbcd	0.120062	1.057332	3.397836	0.002322	0.006169
Gdap2	0.11972	1.129927	2.606358	0.015336	0.032436
Mef2a	0.119551	2.333056	3.687015	0.001128	0.003248
Sod2	0.119268	1.259052	2.755269	0.010892	0.024214
Rnf31	0.119078	1.375914	2.487519	0.020043	0.040984
Bmpr2	0.118957	2.171815	2.51355	0.01891	0.038933
Pi4k2a	0.118955	1.445173	2.424736	0.02304	0.046386
Srp68	0.118844	1.448566	2.38896	0.024928	0.049676
Vamp3	0.118641	1.535799	2.94484	0.006978	0.016419

Rtfdc1	0.118344	1.841835	3.012859	0.005933	0.014272
Canx	0.11801	2.713657	2.841669	0.008902	0.020294
9130401M	0.117845	1.319408	2.578675	0.01633	0.034314
H13	0.117762	1.792609	2.839624	0.008945	0.020367
Pno1	0.117498	0.869892	2.486143	0.020105	0.041098
Tsfm	0.117467	0.391451	2.582544	0.016187	0.034054
Mettl9	0.117437	1.11062	2.852599	0.008677	0.019853
Ylpm1	0.117424	1.436551	3.007	0.006017	0.014465
Gpbp1l1	0.117355	1.998893	2.721633	0.011774	0.025894
Hexb	0.117244	1.847617	2.832486	0.009096	0.020635
Ap2a1	0.117122	0.77921	2.705551	0.01222	0.026664
Tmod2	0.117085	0.351482	2.391501	0.024789	0.049505
Yipf6	0.116914	1.057151	2.853612	0.008656	0.019818
Fam193b	0.116742	1.090563	2.841967	0.008896	0.020293
Crebrf	0.116647	1.407576	2.56739	0.016752	0.035063
Eif3d	0.116604	2.0408	2.629188	0.014559	0.030987
Gfm1	0.116507	1.151048	2.851646	0.008696	0.019885
Snx11	0.116091	1.021728	2.526687	0.018361	0.038003
Trmt2b	0.115979	0.906204	2.399366	0.024365	0.048812
Ndufv1	0.115826	1.151384	2.709613	0.012106	0.026454
Eif3g	0.115815	2.369119	2.618016	0.014934	0.031695
Prmt5	0.115801	0.77932	2.547047	0.017539	0.036484
Rab3gap1	0.115775	0.892564	2.614978	0.015038	0.031888
Ndufaf6	0.11574	0.803392	2.64068	0.014182	0.030323
Commd6	0.115658	1.705717	2.685441	0.012799	0.027773
Ppp6r1	0.115622	1.361619	3.293208	0.003006	0.007787
Scyl1	0.11562	0.975716	2.901476	0.007733	0.017955
Sin3a	0.11539	1.826741	2.892824	0.007892	0.018255
Actr10	0.115266	1.295137	2.775081	0.010402	0.023221
Prex1	0.115076	1.884713	3.021998	0.005805	0.014
Phkb	0.115042	1.478981	2.553861	0.017271	0.036049
Psmd1	0.114708	1.937786	3.265422	0.003218	0.008261
Vgll4	0.114644	1.173927	2.403453	0.024147	0.04843
Mtf1	0.114552	1.335697	3.292451	0.003011	0.007799
Tprgl	0.114451	1.317113	2.677113	0.013046	0.028252
Atp5d	0.114327	1.921949	2.398273	0.024423	0.048906
Gpalpp1	0.114152	1.168105	2.68825	0.012716	0.027618
Pafah1b2	0.113863	1.392545	2.447183	0.021924	0.044367
Stxbp2	0.113858	1.881295	2.84431	0.008847	0.020194
1500012F(	0.113841	2.087017	2.703683	0.012272	0.026756
Sars	0.113736	1.257503	2.458883	0.021362	0.043383
Mdm4	0.11356	2.019115	2.665449	0.0134	0.028876
Tusc3	0.113515	0.667992	2.73943	0.011299	0.024993
Ube4b	0.113432	1.804871	2.971113	0.006555	0.015572
Cops2	0.113362	2.057673	2.590527	0.015897	0.033481
Braf	0.113278	1.56066	2.64188	0.014143	0.030266
Mnat1	0.113276	1.356041	2.81972	0.009372	0.021171
Anapc4	0.113139	1.174322	3.152409	0.004239	0.010579
Mier3	0.11312	1.338917	2.833525	0.009074	0.020591
Ctnnbl1	0.112898	1.124315	2.51064	0.019033	0.039156
Ints7	0.112825	1.010616	2.464692	0.021088	0.042909
Man2a1	0.112416	1.560192	2.784957	0.010165	0.022755

Herpud1	0.112278	2.41554	2.674273	0.013132	0.028404
Surf2	0.111493	1.164273	2.754929	0.0109	0.024226
Trp53bp1	0.111382	0.854533	2.417195	0.023427	0.047101
Luc7l2	0.11126	1.920144	2.984966	0.006342	0.015134
Gm15466	0.111127	0.840831	2.422687	0.023145	0.046584
Slc38a10	0.110691	0.820302	2.570514	0.016634	0.034875
Kmt2e	0.110613	2.373598	2.959771	0.006734	0.015932
Lonp2	0.11043	1.312023	2.620898	0.014837	0.031542
Arrdc1	0.110295	1.611359	2.655903	0.013696	0.029387
Dpp8	0.110088	1.852843	2.473969	0.020658	0.04209
Wdr91	0.109981	1.45577	2.454701	0.021562	0.043716
Synrg	0.109735	1.428944	2.561087	0.016992	0.035526
Tlk1	0.109623	1.76609	2.869595	0.008337	0.019146
Lsm14a	0.109433	1.913677	2.994617	0.006197	0.014834
Atp6v1a	0.109418	1.603811	2.483434	0.020227	0.041302
Ptrhd1	0.109355	1.019496	2.602704	0.015464	0.032651
Saraf	0.108888	1.264638	2.877959	0.008174	0.018821
Fem1b	0.108385	1.11606	2.433187	0.022614	0.045627
Brwd1	0.108287	1.086011	2.43907	0.022322	0.045109
Srek1	0.10806	1.611061	2.552106	0.01734	0.036131
Ier3ip1	0.107807	1.65486	2.61761	0.014948	0.031716
Opa1	0.10771	1.377996	2.998983	0.006133	0.014687
Wdr37	0.107311	1.545405	2.570014	0.016653	0.034895
Rhot2	0.107304	1.062224	2.600637	0.015536	0.032786
Tsc2	0.107167	1.115846	2.91995	0.007402	0.017281
Itm2b	0.107155	3.018469	2.866717	0.008393	0.019264
Golga4	0.10706	1.544381	2.607527	0.015295	0.032359
Cers2	0.106972	1.520636	2.442247	0.022165	0.04483
Sf3b6	0.106785	1.860865	3.072099	0.005147	0.012584
Lsp1	0.106286	2.83572	3.192691	0.003843	0.0097
Gabarap	0.105957	2.656419	2.700961	0.01235	0.026908
Copa	0.10588	2.057471	2.929888	0.00723	0.016921
Atp5b	0.105822	2.738846	2.601016	0.015523	0.032767
Rbm25	0.105714	2.32932	2.820103	0.009364	0.021161
Apobec3	0.105232	2.513644	2.805488	0.00969	0.021811
Rnf4	0.104898	1.843892	2.634343	0.014389	0.030686
Yeats4	0.104197	1.959734	2.445999	0.021982	0.044471
Ace2	0.104192	0.450814	2.418824	0.023343	0.04697
Rab14	0.104145	2.154422	2.395174	0.02459	0.049174
Ttyh3	0.103454	1.988091	2.640923	0.014174	0.030315
Dpp9	0.103439	0.887246	2.435891	0.022479	0.045367
Tmx3	0.102991	1.982227	2.604321	0.015407	0.03255
Wdr1	0.102194	2.356107	2.404482	0.024092	0.048347
Vps25	0.102061	1.644752	2.685282	0.012804	0.027775
Nisch	0.102039	1.638519	2.571668	0.016591	0.034804
Capn7	0.10195	1.798927	2.776101	0.010377	0.023187
Nxf1	0.101846	2.703738	2.425333	0.02301	0.046337
Osbpl8	0.101735	1.88916	2.482798	0.020255	0.041349
Flii	0.101682	1.844019	2.757029	0.010847	0.02413
Tmem59	0.101425	2.089188	2.822091	0.009321	0.021073
Tm9sf4	0.101286	1.122411	2.619447	0.014886	0.031624
Gphn	0.101275	1.354896	2.464046	0.021119	0.042947

Inpp5e	0.100959	0.907796	3.27859	0.003116	0.008038
Csnk1a1	0.10006	2.01292	2.835273	0.009037	0.02052
Heatr5b	0.099801	0.9375	2.576917	0.016395	0.034442
Uqcrc2	0.099668	2.174751	2.862077	0.008486	0.019463
Ralgapa1	0.099352	1.917467	2.391372	0.024796	0.049506
Psmb2	0.099001	2.655856	2.740533	0.011271	0.024944
Calm1	0.098881	2.971189	2.467214	0.02097	0.042692
Tbce	0.098311	1.468194	2.449952	0.02179	0.044131
Phf20l1	0.097016	1.487298	2.560456	0.017016	0.035566
Mbd2	0.096875	1.66839	2.706426	0.012195	0.026626
Crybg3	0.096857	0.949188	2.495121	0.019706	0.040349
Marf1	0.096282	2.02364	2.456233	0.021488	0.043592
Vcpip1	0.095082	1.511422	2.450052	0.021785	0.044131
Arf4	0.093903	2.252125	2.549992	0.017423	0.036266
Srpk1	0.093132	1.836819	2.513076	0.01893	0.038964
Csde1	0.092365	2.841831	2.518811	0.018688	0.03852
Fam120a	0.091891	2.167225	2.525563	0.018407	0.038086
Atrx	0.089856	2.203393	2.709958	0.012096	0.026441
Dap3	0.08982	0.862886	2.401537	0.024249	0.048622
Taok1	0.088575	1.95834	2.527861	0.018312	0.037913
Atp6v1d	0.088235	2.381799	2.517455	0.018745	0.038618
Slu7	0.087498	1.572442	2.790195	0.010042	0.022519
Kidins220	0.087228	1.757377	2.497589	0.019597	0.040161
Kdm1a	0.086799	1.832672	2.694563	0.012533	0.027252
Dync1h1	0.086603	2.064547	2.524755	0.01844	0.038115
Psmc3	0.085064	2.119381	2.523793	0.01848	0.038165
Whsc1l1	-0.08532	1.414714	-2.46614	0.02102	0.042782
Psme4	-0.08774	1.583152	-2.39121	0.024805	0.049511
Cnot1	-0.08891	2.4221	-2.68124	0.012923	0.028026
Sf3b1	-0.08912	2.826474	-2.53789	0.017904	0.037213
Scaper	-0.09033	1.738942	-2.45868	0.021372	0.043391
Uba7	-0.09042	1.860525	-2.47448	0.020634	0.042054
Eef1b2	-0.09089	2.651996	-2.64289	0.01411	0.030222
Dock7	-0.09098	1.317252	-2.52547	0.018411	0.038086
Akap13	-0.09119	2.091855	-2.38855	0.02495	0.049707
Ncoa7	-0.09343	1.339989	-2.52457	0.018448	0.03812
Hira	-0.09453	0.905066	-2.40366	0.024136	0.048421
Tor1aip1	-0.09509	1.907859	-2.41722	0.023426	0.047101
Tnks2	-0.09573	1.931878	-2.66183	0.013512	0.029066
Kdm3b	-0.09765	2.254082	-2.76959	0.010535	0.023484
Brd4	-0.09797	1.656214	-2.70441	0.012252	0.026719
Cops3	-0.09833	1.431336	-2.39635	0.024527	0.049086
Rab3gap2	-0.09869	1.194747	-2.92051	0.007392	0.017269
Arid1a	-0.09903	1.948016	-2.82006	0.009365	0.021161
B2m	-0.09924	3.991378	-2.58711	0.016021	0.033732
Secisbp2	-0.0994	1.065523	-2.50219	0.019397	0.039806
Azi2	-0.09986	1.14787	-2.48233	0.020276	0.041381
Rnf44	-0.10052	1.280467	-2.55682	0.017157	0.03584
Rc3h2	-0.10116	1.920523	-3.07652	0.005092	0.012463
Pabpc1	-0.10134	3.506719	-2.4974	0.019605	0.040166
Tes	-0.10233	1.980563	-2.41573	0.023503	0.047227
Map3k7	-0.10302	1.459891	-2.71366	0.011993	0.026278

Exoc1	-0.10315	1.419971	-2.52184	0.018561	0.038289
B4galnt1	-0.10403	2.126008	-2.71899	0.011846	0.026012
Melk	-0.10433	0.819452	-2.7623	0.010716	0.02385
Ppp1r21	-0.10508	1.374603	-2.51033	0.019046	0.039172
Rraga	-0.10559	1.505848	-2.50576	0.019243	0.039542
Nin	-0.106	1.227728	-2.61006	0.015207	0.032201
Baz1b	-0.10605	1.998735	-2.88113	0.008113	0.018701
Sec13	-0.10619	1.780822	-2.66827	0.013314	0.028731
Agps	-0.1067	1.390176	-2.65493	0.013727	0.029444
Cd86	-0.10697	2.339723	-2.58194	0.01621	0.034091
Ngly1	-0.10749	1.587107	-2.72143	0.01178	0.025894
Mre11a	-0.10752	0.938313	-3.16421	0.004119	0.010308
Atp2c1	-0.10762	1.346344	-2.66044	0.013555	0.029133
Parp2	-0.10785	1.575489	-2.7867	0.010124	0.022676
Slc35e1	-0.10816	1.261318	-2.66282	0.013481	0.029008
Ubp1	-0.1084	1.555856	-3.42202	0.002187	0.005857
Clock	-0.10858	1.383242	-2.41791	0.02339	0.047051
Arpc2	-0.10912	3.153921	-2.68942	0.012682	0.027553
Usp47	-0.10929	2.073428	-2.658	0.013631	0.029263
Son	-0.10991	2.284427	-3.08748	0.004959	0.012186
Ddhd1	-0.11005	2.291334	-2.4373	0.022409	0.045251
Arhgdia	-0.1101	2.119788	-2.60482	0.015389	0.032522
2410004B1	-0.11051	1.360355	-2.49688	0.019628	0.040202
Pan2	-0.11054	1.176089	-2.54919	0.017454	0.036319
Hnrnpa2b1	-0.11173	2.704738	-2.66513	0.01341	0.02888
Wdfy3	-0.11174	0.915581	-2.83994	0.008939	0.020358
Eci1	-0.11285	0.738287	-2.81172	0.00955	0.021519
Ate1	-0.11291	1.432449	-2.62927	0.014556	0.030987
Erap1	-0.1132	1.887065	-2.94965	0.006898	0.016253
Inpp5d	-0.11377	2.085178	-2.59111	0.015876	0.03345
Btaf1	-0.11417	1.501952	-2.76552	0.010635	0.023693
Nupl1	-0.11427	1.63031	-2.87676	0.008197	0.01886
Srrt	-0.11498	1.884976	-2.81473	0.009482	0.021387
2700029M	-0.11539	1.851309	-2.82903	0.00917	0.020784
Fbxo42	-0.11568	0.989407	-2.97006	0.006571	0.015601
Sash3	-0.11579	1.15541	-3.1184	0.004602	0.011392
Nars	-0.11613	2.122896	-3.08592	0.004978	0.012228
Prpf18	-0.11645	0.9543	-2.41479	0.023551	0.047312
B4galt6	-0.11739	1.184828	-2.43998	0.022277	0.045031
Nbeal2	-0.11766	0.732923	-3.0844	0.004996	0.012265
Camk1d	-0.11784	1.425102	-2.8685	0.008358	0.019189
Pygb	-0.11851	1.296611	-2.41388	0.023599	0.047395
Col4a3bp	-0.11865	1.718147	-3.00288	0.006076	0.014583
Rin2	-0.11872	0.437287	-2.83855	0.008968	0.020394
Cep162	-0.11874	1.500192	-2.75603	0.010873	0.024178
Akap8	-0.11905	1.836574	-2.73172	0.011503	0.025368
Csnk1g3	-0.11919	1.585139	-2.75035	0.011017	0.024453
Mtmr3	-0.11928	1.357384	-3.63615	0.001282	0.003634
Trim7	-0.11951	1.126066	-2.88316	0.008074	0.018623
Gsk3a	-0.11979	1.543323	-2.57607	0.016426	0.034498
Rnf216	-0.11987	1.624965	-3.20132	0.003763	0.009518
Trp53bp2	-0.12022	1.137358	-2.98872	0.006285	0.015018

Taf8	-0.12033	1.106069	-2.73503	0.011415	0.025204
Abhd12	-0.1205	1.424465	-2.50106	0.019446	0.039872
Hnrnpd	-0.12081	2.595458	-2.57466	0.016479	0.034598
Cdc25a	-0.12101	0.669507	-2.88566	0.008027	0.018525
Psap	-0.12112	3.039553	-2.72151	0.011778	0.025894
Lmnb1	-0.12166	1.335377	-2.42934	0.022807	0.045954
Tmem245	-0.12166	0.770899	-2.39369	0.024671	0.049281
Anxa1	-0.12168	1.703753	-2.45472	0.021561	0.043716
Stag2	-0.12222	1.830343	-3.2612	0.003251	0.008339
Rps6kc1	-0.12261	0.806086	-2.71221	0.012034	0.026351
LOC102638	-0.12278	2.423007	-2.56485	0.016848	0.035245
Ptcd3	-0.12279	1.03066	-2.61688	0.014973	0.031759
Hat1	-0.12352	2.134483	-2.78035	0.010275	0.022986
Mob1b	-0.12358	1.384507	-2.88085	0.008119	0.018708
Letm2	-0.12361	0.829719	-2.67946	0.012976	0.028125
Fnbp4	-0.12378	1.688385	-3.13128	0.004461	0.011074
Prrc1	-0.12379	0.84355	-3.26594	0.003214	0.008255
Rnf219	-0.12404	1.021448	-3.52568	0.00169	0.004641
Hnrnpab	-0.12413	2.761977	-3.28241	0.003087	0.007971
Trim25	-0.1242	1.472152	-2.78748	0.010105	0.022648
Sf1	-0.12429	2.225194	-3.63427	0.001288	0.003645
Spg11	-0.12448	1.407849	-2.8012	0.009787	0.021996
Ndufa8	-0.12472	1.798648	-2.83628	0.009016	0.020477
Smchd1	-0.12475	1.918152	-3.28997	0.00303	0.007844
Snrnp70	-0.12534	2.443824	-2.45926	0.021345	0.043359
Med14	-0.12567	1.30117	-3.11248	0.004669	0.011541
Vmp1	-0.12595	1.166318	-2.90885	0.007599	0.017692
Ifngr2	-0.12639	2.366963	-2.57172	0.016589	0.034804
Zdhhc21	-0.12685	1.185082	-2.51742	0.018746	0.038618
Atxn2	-0.12699	1.340346	-3.27126	0.003172	0.008161
Rtn3	-0.12703	2.2484	-2.71071	0.012075	0.026411
Anp32a	-0.12713	2.108901	-2.52202	0.018554	0.038289
Pacsin2	-0.12721	1.277942	-3.13549	0.004416	0.010971
Camta2	-0.12731	1.751765	-3.16625	0.004099	0.010261
Psmb9	-0.12734	3.207505	-2.91167	0.007548	0.017596
Cnbp	-0.12785	2.580341	-3.34567	0.002642	0.006936
LOC102631	-0.12815	2.95454	-3.16208	0.00414	0.010351
Rab1	-0.12889	2.802466	-2.52934	0.018252	0.03783
Hnrnph3	-0.12933	2.236352	-2.85143	0.008701	0.019889
Upf2	-0.12988	1.489017	-2.71386	0.011988	0.026275
R3hcc1l	-0.1299	1.510733	-2.55231	0.017332	0.036131
Zfhx3	-0.13059	0.632436	-3.15994	0.004162	0.010397
Bnip2	-0.13092	2.160413	-2.69799	0.012434	0.027061
Fancl	-0.13093	1.538037	-2.83811	0.008977	0.020402
B4galt1	-0.13098	1.06416	-3.49727	0.001814	0.004934
Foxk2	-0.13099	1.232064	-2.80315	0.009743	0.021908
Smg1	-0.13102	2.08824	-3.34401	0.002653	0.006962
Srsf7	-0.13115	2.53225	-2.67667	0.01306	0.028273
Mms22l	-0.13145	0.718184	-3.19497	0.003822	0.009653
Clstn1	-0.13154	0.595641	-2.76407	0.010672	0.023767
Fam222b	-0.13169	0.937136	-2.46743	0.02096	0.042683
Eif1	-0.13184	3.42475	-3.00307	0.006073	0.014582

Usp32	-0.13207	1.477967	-3.62723	0.001311	0.003699
Rbm5	-0.1323	2.433644	-2.96509	0.006649	0.015762
Fam45a	-0.13234	1.302014	-3.38544	0.002395	0.006348
Traf1	-0.1324	2.419762	-2.74763	0.011087	0.024588
Coro1a	-0.13251	2.068546	-2.88606	0.008019	0.018514
Fam196b	-0.13281	1.126676	-2.8523	0.008683	0.019861
Safb2	-0.13283	1.872167	-2.50492	0.019279	0.039594
Pcnt	-0.13286	0.979159	-3.05884	0.005313	0.012945
Nop58	-0.13293	1.668051	-2.98071	0.006407	0.015278
Sh3bgrl3	-0.13331	3.429952	-3.02809	0.00572	0.01381
Mark3	-0.13349	1.666827	-2.65868	0.01361	0.029234
Lysmd1	-0.13357	1.611756	-2.44816	0.021877	0.044283
Irf1	-0.13374	1.669819	-2.86656	0.008397	0.019265
Tbc1d8	-0.13375	2.573459	-3.07904	0.005061	0.012396
Fuca2	-0.13379	1.303198	-2.39774	0.024452	0.04895
Mtmr6	-0.1349	1.453289	-3.05083	0.005417	0.01318
Nfkb1	-0.13508	2.052196	-3.24647	0.003371	0.008614
Tnrc6a	-0.13526	1.580605	-2.89607	0.007832	0.018143
Ppp1r15a	-0.13543	2.338194	-2.56909	0.016688	0.034958
Pank2	-0.13548	1.613863	-3.78121	0.000889	0.002638
Ptch1	-0.13555	0.858316	-2.53293	0.018105	0.037578
Cd2ap	-0.13564	1.850715	-2.50113	0.019443	0.039872
Lsm3	-0.13565	2.117031	-2.752	0.010975	0.024376
Dcp2	-0.13582	1.734813	-3.1744	0.004018	0.010083
Lyar	-0.13587	0.950624	-2.74672	0.01111	0.024633
Satb1	-0.13603	0.882579	-2.5349	0.018025	0.037432
Strada	-0.1362	1.256151	-3.14444	0.004321	0.01076
Tm9sf2	-0.13654	2.038246	-3.55342	0.001577	0.004366
5031414D	-0.13688	1.246494	-2.85008	0.008728	0.01994
Rgs19	-0.13703	1.503003	-2.8055	0.009689	0.021811
Trim36	-0.13729	0.753622	-2.87567	0.008218	0.018903
Cdc42	-0.1374	3.138675	-3.1053	0.004751	0.011724
Stat1	-0.13742	2.13145	-3.00605	0.00603	0.014493
Rfc2	-0.13752	1.085991	-2.50466	0.01929	0.039607
Mir467c	-0.13772	1.861454	-3.34848	0.002624	0.006893
Dpcd	-0.13793	1.231023	-2.389	0.024926	0.049676
Tbc1d5	-0.13801	1.503052	-3.32554	0.002776	0.007253
Cpsf2	-0.13802	1.469826	-3.10528	0.004751	0.011724
Rpa1	-0.13807	1.084546	-2.94213	0.007023	0.016504
Ehd1	-0.13861	0.963382	-2.52329	0.018501	0.038198
Ubap2	-0.13913	1.545244	-3.76337	0.00093	0.002739
Dscc1	-0.13924	0.524266	-2.53653	0.017959	0.037317
Bcl10	-0.13932	1.424279	-3.11034	0.004693	0.011597
Lcp1	-0.13939	2.512655	-3.62751	0.00131	0.003698
Rfx7	-0.13965	1.708401	-2.76673	0.010606	0.023634
Ccnt1	-0.14005	1.571821	-3.06136	0.005281	0.012879
Ap4b1	-0.14007	0.670758	-2.71089	0.01207	0.026411
Hnrnpdl	-0.14009	2.328363	-3.17341	0.004028	0.010098
Clip1	-0.14015	1.563637	-2.90111	0.007739	0.017962
Shisa5	-0.14053	1.454891	-2.52914	0.01826	0.037836
Pold4	-0.1406	1.741584	-2.66018	0.013563	0.029142
Baz2a	-0.14066	1.706795	-3.22652	0.003539	0.009002

Fbrsl1	-0.14075	1.730538	-3.35533	0.00258	0.006789
Incenp	-0.14082	0.82358	-3.41979	0.0022	0.005883
Gltscr1	-0.14083	0.988417	-3.21434	0.003646	0.009249
Senp1	-0.14116	1.377717	-2.95928	0.006742	0.015936
Srrm2	-0.14121	2.911686	-4.4235	0.000173	0.000604
Zfp277	-0.14131	1.362672	-3.04042	0.005554	0.013478
Gm20199	-0.14138	1.755651	-2.97667	0.006469	0.015407
Rnf167	-0.14143	1.731374	-2.95664	0.006785	0.016021
Arhgap25	-0.14145	1.25869	-2.63312	0.014429	0.030745
Casp1	-0.14161	1.682559	-2.83922	0.008954	0.020368
Lrch3	-0.14197	1.96636	-3.54876	0.001596	0.004409
Abce1	-0.14207	1.555483	-3.17265	0.004035	0.010113
Nufip1	-0.1423	1.042974	-3.24279	0.003401	0.008681
Rbm39	-0.14249	2.961769	-3.95667	0.00057	0.001773
Tra2a	-0.14273	1.910154	-3.95224	0.000576	0.001791
Eif5b	-0.14302	1.866643	-3.60779	0.001377	0.003862
Dlst	-0.14303	1.567568	-3.94889	0.000581	0.001806
Fam168a	-0.1431	1.551785	-2.90928	0.007591	0.017684
Nampt	-0.14324	1.658972	-2.74188	0.011235	0.024874
Tcerg1	-0.14339	1.838602	-3.76741	0.000921	0.002716
Herc3	-0.14359	1.06192	-3.27187	0.003167	0.008155
Adrbk1	-0.14386	2.023639	-2.88757	0.007991	0.018465
Srsf11	-0.14421	2.160582	-3.4459	0.002062	0.005548
Diablo	-0.14433	1.52689	-2.50797	0.019147	0.039358
Usp25	-0.14468	2.011629	-3.63466	0.001287	0.003643
4930503L1	-0.14491	0.806911	-2.49285	0.019806	0.040543
Cyp4v3	-0.14496	1.310784	-2.66758	0.013335	0.02876
Nup153	-0.14515	1.91263	-4.74849	7.49E-05	0.000284
H3f3b	-0.14524	1.633576	-2.4613	0.021248	0.043174
Slc25a40	-0.14573	0.575392	-2.71105	0.012066	0.026411
Swt1	-0.14581	1.034414	-3.36501	0.002519	0.006657
Tmem135	-0.14581	0.970449	-3.2386	0.003436	0.008759
Zfp592	-0.1459	1.347266	-3.40234	0.002297	0.006112
Piezo1	-0.14644	1.349117	-3.87326	0.000704	0.002157
Dusp22	-0.14653	1.516927	-2.57143	0.0166	0.034813
Ikzf1	-0.14701	1.740074	-2.84332	0.008868	0.020234
Phf11a	-0.14721	1.632533	-2.53791	0.017903	0.037213
Dhrs3	-0.1474	0.939825	-2.9292	0.007241	0.016944
Srpk2	-0.14754	1.057848	-3.17378	0.004024	0.010092
Elk1	-0.14779	0.870824	-2.83929	0.008952	0.020368
G2e3	-0.1479	0.938569	-3.81318	0.00082	0.002468
Atf6b	-0.14847	1.376521	-3.119	0.004596	0.011383
Zufsp	-0.14873	1.794875	-3.80946	0.000828	0.002488
Agap1	-0.14884	0.929739	-3.41104	0.002248	0.005994
6820431F2	-0.14913	1.913989	-2.79654	0.009894	0.022228
Hnrnpm	-0.14917	2.866667	-3.31105	0.002877	0.007492
Htt	-0.14968	1.433137	-4.17624	0.000325	0.00107
Pten	-0.15024	2.527088	-3.65713	0.001216	0.003476
Jmjd1c	-0.15042	2.264735	-3.33616	0.002704	0.007083
Aste1	-0.15044	0.952968	-3.78332	0.000885	0.002626
Ccdc163	-0.15067	0.811376	-2.6932	0.012573	0.02733
Hspa14	-0.15071	1.262142	-3.95469	0.000573	0.001782

Akt2	-0.15077	1.131253	-3.25317	0.003316	0.00849
Tk2	-0.15098	0.92809	-2.59106	0.015878	0.03345
Atg4c	-0.15126	0.721667	-2.8048	0.009705	0.02183
Cyb5r3	-0.15127	2.019527	-2.87034	0.008322	0.019124
Nap1l4	-0.15138	1.476416	-3.42255	0.002185	0.005851
Dapp1	-0.15156	2.26016	-4.038	0.000463	0.00147
Bak1	-0.15156	2.320296	-2.94294	0.007009	0.016483
Alpk1	-0.15167	0.94795	-3.56704	0.001524	0.004235
Fam35a	-0.15171	0.907546	-2.83466	0.00905	0.020543
Btbd9	-0.15192	1.040347	-3.53085	0.001669	0.004591
Wnk1	-0.15241	2.749908	-4.21183	0.000297	0.000987
D5Ertd579	-0.15247	0.872171	-2.63891	0.014239	0.030429
H2-Eb1	-0.15311	3.016444	-4.20705	0.000301	0.000998
Kif18a	-0.1533	0.58468	-3.09138	0.004913	0.012092
Ammecr1	-0.15354	0.853479	-2.59898	0.015595	0.0329
4930594C1	-0.1537	0.348568	-2.5351	0.018017	0.037426
Galnt1	-0.15374	2.054432	-3.71602	0.001049	0.003044
Sfi1	-0.15394	1.124402	-3.63523	0.001285	0.003641
Acsl1	-0.15434	0.799118	-2.91776	0.00744	0.017365
Tlr11	-0.15441	1.768509	-2.39421	0.024642	0.049252
Pom121	-0.15447	1.356139	-2.98894	0.006282	0.015015
Zcchc7	-0.15474	1.874917	-4.45656	0.000159	0.00056
Mto1	-0.15492	0.841962	-4.08603	0.00041	0.001316
Ndel1	-0.15526	0.931703	-3.83766	0.000771	0.002329
Ppp1r12a	-0.15577	2.227101	-3.77029	0.000914	0.002702
Thap6	-0.15629	1.050827	-2.89151	0.007917	0.0183
Rif1	-0.1566	1.093775	-3.79345	0.000862	0.00257
Narf	-0.15684	0.67588	-2.71563	0.011939	0.026201
Fam214a	-0.15733	0.833277	-3.09461	0.004875	0.012006
Mpzl2	-0.15761	0.628416	-2.4644	0.021102	0.042925
Rnf41	-0.15769	0.999068	-3.09501	0.00487	0.012
Alkbh1	-0.15771	1.320352	-3.002	0.006089	0.014605
Fgfr1op	-0.15787	1.025583	-2.45286	0.02165	0.043871
Dhx40	-0.15791	1.21905	-2.9456	0.006965	0.016395
Ccnl1	-0.15829	2.582968	-4.28929	0.000244	0.000826
Mif4gd	-0.15889	1.076255	-2.90023	0.007756	0.017994
Depdc5	-0.15896	1.065604	-2.8409	0.008918	0.020325
Phactr2	-0.15896	1.084837	-3.302	0.002942	0.007647
Fam122a	-0.15912	1.236987	-2.82584	0.009239	0.020914
Cenpi	-0.15913	0.404528	-3.48422	0.001874	0.005084
Sfmbt1	-0.15916	1.167087	-3.2118	0.003668	0.009298
Far1	-0.15928	2.275113	-4.454	0.00016	0.000563
Arid4a	-0.1593	2.017235	-4.03031	0.000472	0.001496
Diap2	-0.15939	1.284058	-3.98113	0.000535	0.001673
Gdi2	-0.15957	2.82254	-3.92329	0.00062	0.001921
Supt3	-0.16002	1.162331	-3.53757	0.001641	0.00452
Lasp1	-0.16018	1.205906	-2.80767	0.00964	0.021717
Actr2	-0.16028	2.57821	-3.85083	0.000746	0.002266
Ccdc97	-0.16037	1.367774	-3.68377	0.001137	0.003272
Dtd1	-0.16053	0.65376	-3.55906	0.001555	0.004312
Trip13	-0.1607	0.503033	-2.53123	0.018174	0.03769
8030462N1	-0.1608	0.771739	-2.42968	0.02279	0.045932

Lnpep	-0.16083	2.068358	-4.19245	0.000312	0.001032
Ulbp1	-0.16111	0.875773	-3.4474	0.002054	0.005533
Ccdc34	-0.16136	0.785501	-3.15217	0.004241	0.010582
Marveld1	-0.16149	1.380722	-3.06486	0.005237	0.012788
Ccl5	-0.1615	3.336834	-2.47283	0.02071	0.042185
Rnf13	-0.16155	1.542152	-3.9041	0.000651	0.002009
Rn7sk	-0.16185	3.936097	-2.48375	0.020212	0.041284
Mrps18b	-0.16189	0.953496	-2.52809	0.018303	0.037904
Pkn1	-0.16189	1.37455	-2.68091	0.012933	0.02804
Kmt2b	-0.16202	1.155023	-3.924	0.000619	0.001918
Mgat4a	-0.16211	0.935359	-3.61693	0.001345	0.003786
Tbk1	-0.16213	1.966961	-3.81049	0.000826	0.002483
Spi1	-0.16226	2.747997	-3.89933	0.000659	0.00203
Srrm1	-0.16229	2.461497	-4.10165	0.000394	0.001273
Sfpq	-0.16239	2.402852	-3.88893	0.000677	0.002082
Irf9	-0.1624	1.326227	-3.70064	0.00109	0.003148
Cpsf3	-0.16248	1.437885	-3.31504	0.002849	0.00743
Mapre1	-0.16252	1.957259	-3.75153	0.000959	0.002809
Hccs	-0.1626	1.113608	-3.65923	0.00121	0.003462
Ckap5	-0.16293	1.445058	-4.82218	6.20E-05	0.000241
Tcf20	-0.16294	1.879379	-3.35823	0.002561	0.006753
Coro1c	-0.16294	1.715197	-3.29774	0.002973	0.007717
Pnpla7	-0.1631	0.635752	-3.93516	0.000602	0.001867
Pola2	-0.1632	0.847416	-3.01397	0.005917	0.014253
Mbtd1	-0.16335	1.495895	-3.08471	0.004993	0.01226
Nadk2	-0.16369	0.920568	-2.76332	0.01069	0.023801
Tns1	-0.16381	0.833085	-2.98763	0.006302	0.015052
Naa16	-0.16404	1.06612	-3.63653	0.001281	0.003631
Ubap1	-0.16411	1.430093	-4.25714	0.000264	0.000891
Nup85	-0.16468	1.426979	-3.30932	0.002889	0.007519
Asap1	-0.16478	2.462279	-3.59359	0.001426	0.003987
Tmem29	-0.16512	0.892522	-3.08167	0.005029	0.012334
Unc119	-0.16516	0.601624	-2.64118	0.014166	0.030306
Gng2	-0.16519	0.798491	-3.3404	0.002676	0.007017
Irak1	-0.1653	1.341535	-3.77616	0.000901	0.002666
Rbm33	-0.16531	1.305245	-4.12085	0.000375	0.001216
Uqcc2	-0.16534	1.406068	-2.63315	0.014428	0.030745
4930599N:	-0.1655	0.897837	-3.15985	0.004163	0.010397
Chek2	-0.16573	0.700666	-2.52173	0.018566	0.038289
Efcab14	-0.16573	1.850127	-4.13241	0.000364	0.001184
Smc1a	-0.166	1.973234	-3.64805	0.001244	0.003544
Gtf2a2	-0.1662	1.442646	-4.04392	0.000456	0.001449
Ilk	-0.16626	1.469399	-3.51495	0.001736	0.004751
Plxnb2	-0.16639	0.998631	-3.73165	0.001008	0.00294
Pip4k2b	-0.16645	1.074096	-3.08361	0.005006	0.012284
Rprd1b	-0.16674	0.867049	-3.30517	0.002919	0.007593
Zmiz1	-0.16688	1.867586	-3.87062	0.000709	0.002169
Srsf10	-0.16695	1.543806	-2.73824	0.011331	0.025054
Kras	-0.16737	1.549534	-4.05236	0.000447	0.001419
Cenpj	-0.1674	0.963394	-4.256	0.000265	0.000893
Sirt7	-0.1677	1.327557	-3.31487	0.00285	0.00743
Eya3	-0.16802	1.873239	-4.30102	0.000236	0.000803

Ank	-0.16808	0.895074	-2.6049	0.015387	0.032522
Mical1	-0.16812	1.274073	-2.97135	0.006551	0.015568
Cxcr4	-0.16853	1.77634	-3.16352	0.004126	0.010322
Myl12b	-0.16891	2.870282	-3.80108	0.000846	0.002533
Ppfibp2	-0.16938	1.065372	-3.4981	0.001811	0.004928
Slc15a4	-0.16944	1.501298	-2.84851	0.008761	0.020002
Adap2	-0.16965	1.124237	-3.52498	0.001693	0.004647
Psip1	-0.16975	1.862365	-3.87209	0.000707	0.002162
Calhm2	-0.16975	1.277094	-3.48922	0.001851	0.005023
Pds5a	-0.16975	2.096765	-3.53861	0.001637	0.004513
Lpcat4	-0.16991	1.365867	-4.22386	0.000288	0.000961
Dennd1b	-0.16999	1.314208	-3.44695	0.002056	0.005537
Kmt2c	-0.17016	1.825112	-4.76689	7.14E-05	0.000272
Fbxo45	-0.1703	1.084783	-3.88365	0.000686	0.002106
Ints10	-0.17104	0.683671	-3.64393	0.001257	0.003573
Acsl5	-0.17116	1.94874	-4.22246	0.000289	0.000964
Slc9a3r1	-0.1716	1.372582	-3.35567	0.002578	0.006786
Pcf11	-0.17161	1.824475	-3.63482	0.001286	0.003643
Cep120	-0.17165	1.218063	-4.49271	0.000144	0.000515
Laptm5	-0.17197	3.54638	-4.20107	0.000305	0.001012
Hnrnpll	-0.17226	1.304081	-4.23767	0.000278	0.000931
Tmem231	-0.17241	0.51496	-4.08669	0.000409	0.001315
Mcm2	-0.17282	0.860483	-3.03328	0.00565	0.013662
Vps37c	-0.17285	1.134044	-3.04524	0.00549	0.013345
Pik3c2a	-0.17295	0.919313	-3.7215	0.001034	0.003009
Adam8	-0.17306	2.05023	-3.39103	0.002362	0.006267
Prkd2	-0.17334	0.831915	-3.88868	0.000677	0.002083
Pttg1	-0.17334	1.45258	-3.61276	0.001359	0.00382
Knstrn	-0.17336	0.840076	-3.8573	0.000734	0.002236
Rangap1	-0.17382	0.840656	-3.7577	0.000944	0.002769
Zc3h12d	-0.17412	1.239377	-2.48489	0.020161	0.04119
Clec2i	-0.17458	1.728251	-2.63785	0.014274	0.030485
Spryd7	-0.17507	0.845007	-2.93954	0.007066	0.016596
Tatdn2	-0.17572	1.043886	-4.45449	0.000159	0.000563
Gm10052	-0.17635	3.30828	-2.58552	0.016078	0.033844
Mta2	-0.17644	1.781806	-4.78786	6.77E-05	0.00026
Fosl2	-0.17659	0.558078	-3.23733	0.003447	0.008783
Rnf111	-0.17663	1.62101	-5.17193	2.53E-05	0.000107
Mcm3	-0.17678	1.269073	-2.99992	0.006119	0.014664
Hmgb2	-0.17697	1.280703	-2.39406	0.024651	0.049255
Fmnl1	-0.17712	1.81422	-3.9737	0.000546	0.001702
Nav1	-0.17718	1.876021	-4.4863	0.000147	0.000523
Mfng	-0.17724	0.685373	-4.08049	0.000416	0.001332
Orc6	-0.17739	1.032904	-3.54752	0.001601	0.004419
2700094K1	-0.17762	1.911851	-3.71341	0.001055	0.003058
Neat1	-0.17799	2.406198	-3.50382	0.001785	0.004867
Rps5	-0.17817	3.860492	-3.84838	0.00075	0.002279
Ppp1r12c	-0.1789	0.892092	-3.85378	0.00074	0.002253
Setd2	-0.17912	2.195387	-4.76731	7.14E-05	0.000272
Pik3r5	-0.17921	1.861304	-4.52264	0.000134	0.00048
Nmi	-0.17921	1.661898	-3.29359	0.003003	0.007783
Atp10d	-0.17981	1.618175	-3.64974	0.001239	0.00353

Ncf2	-0.18047	1.087492	-2.87789	0.008175	0.018821
Usp45	-0.18071	0.946209	-3.43033	0.002143	0.005752
Igtp	-0.18093	1.175152	-2.89263	0.007896	0.018257
Hjurp	-0.18143	1.229669	-4.54773	0.000125	0.000453
Trit1	-0.18144	1.071676	-3.08054	0.005043	0.012359
Sbf1	-0.18163	1.34666	-4.44233	0.000164	0.000579
Stk24	-0.18174	1.678784	-3.57393	0.001498	0.004167
Gripap1	-0.18179	1.069424	-4.55151	0.000124	0.000449
Paxip1	-0.18209	1.081477	-3.93627	0.0006	0.001862
Umps	-0.18222	1.065568	-2.69009	0.012663	0.027518
Zfp36	-0.18237	3.523327	-3.99779	0.000513	0.00161
Ran	-0.18243	1.930818	-2.91297	0.007525	0.017547
Krtcap3	-0.18273	0.873267	-2.39927	0.02437	0.048812
Mbnl1	-0.18284	2.777546	-4.98727	4.06E-05	0.000164
Anpep	-0.18319	2.217021	-2.93721	0.007105	0.016662
Clk1	-0.18385	3.009858	-4.80745	6.44E-05	0.000249
Kif3b	-0.18426	1.068286	-3.98464	0.000531	0.001659
Sec24b	-0.18447	1.750333	-4.63359	0.000101	0.000372
Rbks	-0.18455	0.865007	-3.30074	0.002951	0.007668
Dlg1	-0.18465	1.583104	-4.63258	0.000101	0.000373
Sgsh	-0.18511	0.679846	-3.87397	0.000703	0.002154
Rnpep	-0.18521	1.142445	-3.00708	0.006016	0.014465
Mbip	-0.18521	1.242626	-4.24241	0.000275	0.000921
Eif4g3	-0.18584	1.469983	-3.22209	0.003577	0.009094
Tbrg1	-0.18598	2.153279	-4.72424	7.97E-05	0.000301
Dhx9	-0.18633	2.181312	-5.05747	3.39E-05	0.000139
Cited2	-0.18642	1.956798	-3.35914	0.002556	0.006743
Wdr44	-0.18655	0.904557	-3.16992	0.004062	0.010177
Dpy19l1	-0.1869	1.957706	-3.5262	0.001688	0.004637
Trim56	-0.18696	1.175273	-2.77516	0.0104	0.023221
Tpp2	-0.1871	1.787358	-4.77477	7.00E-05	0.000268
Fermt3	-0.18738	1.185015	-4.17266	0.000328	0.001078
Rgs14	-0.18783	0.50262	-4.76227	7.23E-05	0.000275
5830418K(	-0.18783	1.194607	-3.71422	0.001053	0.003054
Zbtb11	-0.18787	1.277858	-4.17971	0.000323	0.001062
Ankfy1	-0.18878	1.560865	-4.09713	0.000398	0.001286
Plekhm3	-0.1889	1.290867	-4.04884	0.000451	0.001431
Hibch	-0.18891	0.789106	-3.4956	0.001822	0.004951
Tbc1d31	-0.18903	0.648664	-4.32222	0.000224	0.000764
Hnrnph1	-0.18904	2.947994	-4.41331	0.000177	0.000619
Gpatch2l	-0.18918	1.363964	-3.4977	0.001812	0.004931
Nckap1l	-0.1894	2.606776	-5.30062	1.82E-05	7.95E-05
Hspd1	-0.1898	2.440421	-2.80508	0.009699	0.021823
Grb2	-0.19052	1.464704	-3.12684	0.004509	0.011184
Nup155	-0.19068	1.239789	-4.59971	0.00011	0.000402
Alox5ap	-0.19074	2.261172	-3.20277	0.00375	0.009491
Topbp1	-0.19092	1.618493	-4.54068	0.000128	0.000461
Ythdf3	-0.19095	1.680745	-3.65796	0.001214	0.00347
Mllt4	-0.19124	1.289232	-5.15515	2.64E-05	0.000111
Epn1	-0.19125	1.177669	-3.52941	0.001675	0.004605
Mcm7	-0.19126	0.790503	-3.97161	0.000549	0.001709
Nedd4	-0.19152	2.204427	-3.84634	0.000754	0.002289

Ndc1	-0.19172	0.631614	-3.35704	0.002569	0.00677
Ctso	-0.19211	1.160424	-4.06964	0.000427	0.001364
Cenpa	-0.19222	0.804425	-4.25557	0.000266	0.000893
Hells	-0.19241	1.143537	-3.79414	0.000861	0.002567
Lrch1	-0.19307	2.167385	-4.0784	0.000418	0.001339
LOC10263[illegible]	-0.19313	0.684433	-3.29698	0.002978	0.007723
Ccz1	-0.19342	1.669589	-5.79993	5.16E-06	2.51E-05
Ap1s3	-0.19371	1.983492	-3.25495	0.003301	0.008456
Ufsp2	-0.194	1.46089	-4.74258	7.60E-05	0.000288
Gm19710	-0.19417	0.981202	-3.24051	0.00342	0.008724
Morc3	-0.19494	1.516031	-4.37804	0.000194	0.00067
Depdc7	-0.195	1.010153	-2.92222	0.007362	0.017205
Sesn2	-0.19506	0.807577	-3.28649	0.003056	0.0079
Atox1	-0.19588	3.259707	-5.09919	3.05E-05	0.000126
Rufy3	-0.19611	1.138772	-4.63518	0.0001	0.000371
Rmdn1	-0.19666	0.66582	-4.23715	0.000278	0.000932
Inpp1	-0.19673	0.777203	-2.8265	0.009225	0.020895
Arpc5l	-0.19673	2.160004	-4.09327	0.000402	0.001296
Mark2	-0.19686	1.12627	-3.26703	0.003205	0.008238
Hspa5	-0.19716	2.727822	-3.84373	0.000759	0.002298
Snord14a	-0.19729	1.422328	-3.44678	0.002057	0.005538
M6pr	-0.19729	2.701022	-5.24057	2.12E-05	9.15E-05
Ttc37	-0.19783	0.813833	-5.18261	2.46E-05	0.000104
Strbp	-0.19809	1.704816	-3.6119	0.001362	0.003827
Plk4	-0.19835	1.160945	-4.0654	0.000432	0.001377
LOC10264[illegible]	-0.19891	0.689042	-2.715	0.011956	0.026228
BC035044	-0.19912	1.475157	-3.27659	0.003131	0.008072
Mtdh	-0.19926	2.197541	-4.80966	6.40E-05	0.000248
Pole4	-0.2008	0.70272	-3.40021	0.002309	0.00614
Aurkb	-0.20141	0.709217	-2.93859	0.007082	0.016623
Tmem50b	-0.20153	1.674087	-3.7309	0.00101	0.002944
Tespa1	-0.20202	0.560346	-3.64759	0.001246	0.003546
Dusp2	-0.20238	1.319841	-3.36586	0.002513	0.006647
Rb1	-0.20251	2.004595	-4.84135	5.90E-05	0.000231
Snord47	-0.20262	1.821415	-4.07605	0.00042	0.001345
Rogdi	-0.20349	2.974476	-4.33095	0.000219	0.000748
Fam107b	-0.20352	1.35075	-3.8414	0.000764	0.00231
Dcaf12	-0.20354	1.513082	-3.81578	0.000815	0.002453
Stx17	-0.2039	1.441965	-3.58506	0.001457	0.004062
Ncoa3	-0.20393	2.116309	-5.96167	3.44E-06	1.74E-05
Ncapd3	-0.20466	1.073615	-5.28785	1.88E-05	8.17E-05
Gpn1	-0.20498	1.154041	-3.41886	0.002205	0.005892
Heca	-0.2053	1.456823	-4.65308	9.57E-05	0.000356
Rap1b	-0.20531	2.888256	-5.13079	2.81E-05	0.000117
Clec7a	-0.20553	0.992222	-4.50208	0.000141	0.000504
Bcl6	-0.20582	2.462372	-5.36325	1.55E-05	6.89E-05
Snx22	-0.20624	0.707416	-3.7363	0.000996	0.00291
Klf6	-0.20629	2.312402	-4.58402	0.000114	0.000417
Mad2l1	-0.20655	1.069127	-3.27779	0.003122	0.008051
Capza2	-0.20696	2.601376	-3.76462	0.000928	0.002732
LOC10263[illegible]	-0.20705	0.670373	-4.33251	0.000218	0.000746
Midn	-0.20776	1.266575	-4.46062	0.000157	0.000555

Snord69	-0.20783	0.816812	-2.62772	0.014608	0.031082
P2ry6	-0.208	1.644373	-2.64626	0.014002	0.030008
5430427O:	-0.20855	0.757408	-3.32882	0.002754	0.007199
Gas5	-0.20861	2.583922	-5.06914	3.29E-05	0.000135
Lrr1	-0.20898	0.378434	-4.00798	0.0005	0.001572
Il10rb	-0.20901	1.603575	-4.63217	0.000101	0.000373
Hirip3	-0.20908	0.68701	-5.44006	1.28E-05	5.75E-05
Nfatc3	-0.20914	1.743693	-5.34256	1.64E-05	7.21E-05
Pxn	-0.20923	1.719416	-4.47739	0.00015	0.000534
Akirin1	-0.20926	1.473148	-2.63862	0.014249	0.03044
Pigf	-0.20995	0.989664	-3.65681	0.001217	0.003478
Eed	-0.21002	1.80643	-4.3115	0.00023	0.000784
Cnpy3	-0.21022	1.574625	-4.15603	0.000343	0.001119
Herc1	-0.21023	1.527036	-6.09579	2.46E-06	1.28E-05
Parp9	-0.21043	1.36094	-5.06392	3.34E-05	0.000137
Pgap1	-0.21043	0.701592	-4.59903	0.00011	0.000402
Snora44	-0.21044	1.251443	-2.61934	0.014889	0.031624
Nfam1	-0.21063	1.143954	-3.24907	0.003349	0.008566
Cmip	-0.21067	1.427009	-4.55066	0.000125	0.00045
Mcm6	-0.21072	1.38022	-4.01915	0.000486	0.001533
Eogt	-0.21078	0.744212	-4.58113	0.000115	0.000419
Was	-0.21108	1.140979	-3.90047	0.000657	0.002025
Tatdn3	-0.21138	0.779929	-3.47652	0.001911	0.005177
Rp2h	-0.2115	2.093464	-4.44942	0.000161	0.000569
BC017643	-0.21155	1.018499	-2.77673	0.010362	0.02316
Ifrd1	-0.21169	1.800685	-3.43641	0.002111	0.00567
Dleu2	-0.21209	1.295201	-4.52072	0.000134	0.000483
Ildr1	-0.21227	0.386304	-4.03058	0.000472	0.001496
Mospd2	-0.21247	1.222044	-6.03328	2.88E-06	1.48E-05
Ctc1	-0.21256	0.757372	-3.63766	0.001277	0.003624
Rac2	-0.21299	1.816724	-2.75027	0.011019	0.024453
Smpdl3a	-0.21304	1.245724	-4.22296	0.000289	0.000963
Lat2	-0.21317	1.108742	-3.42668	0.002162	0.005798
Snora3	-0.21318	1.622295	-2.93069	0.007216	0.016896
Lrrfip1	-0.21339	1.435042	-6.41775	1.11E-06	6.22E-06
Adrbk2	-0.21347	1.979823	-5.18181	2.47E-05	0.000104
Anapc16	-0.21349	1.420542	-4.35279	0.000207	0.00071
Arfgef1	-0.21395	1.9483	-6.13401	2.24E-06	1.17E-05
2310010J1	-0.21454	0.713265	-3.51346	0.001743	0.004767
Itgal	-0.21458	1.415282	-4.07316	0.000423	0.001354
Fam72a	-0.21476	0.280268	-3.74772	0.000968	0.002835
Irak4	-0.2148	1.769534	-4.34668	0.00021	0.00072
Sipa1	-0.215	1.125533	-6.05433	2.73E-06	1.41E-05
Atp8b4	-0.21507	1.043946	-3.78782	0.000875	0.002602
Mcur1	-0.21511	1.357922	-3.80157	0.000845	0.002532
Ctsa	-0.21514	1.673967	-2.74351	0.011193	0.024795
Mitd1	-0.21563	1.025117	-3.18871	0.003881	0.009776
Tspan13	-0.21591	2.172165	-4.30396	0.000235	0.000797
Blm	-0.21601	0.544036	-3.4501	0.00204	0.005504
Nsmaf	-0.21668	1.966874	-4.39153	0.000187	0.00065
Whsc1	-0.21673	1.385659	-5.55814	9.48E-06	4.38E-05
Azin1	-0.21721	1.66307	-5.35367	1.59E-05	7.03E-05

Elf4	-0.21753	2.083786	-5.11781	2.91E-05	0.000121
Sass6	-0.21769	1.02954	-5.47673	1.17E-05	5.29E-05
Ube2s	-0.21785	2.388934	-2.72353	0.011723	0.025799
Haao	-0.21805	1.339545	-3.91983	0.000626	0.001937
Sumf1	-0.21838	0.907106	-4.5077	0.000139	0.000498
Ttyh2	-0.21861	0.534377	-4.18018	0.000322	0.001062
Map3k3	-0.21875	1.538622	-4.2807	0.000249	0.000842
Spc24	-0.21914	0.709408	-4.06158	0.000436	0.00139
Prdx6	-0.21927	1.087368	-3.24278	0.003401	0.008681
Cntln	-0.21964	0.90376	-4.25998	0.000263	0.000885
Malat1	-0.21983	3.620507	-5.17725	2.50E-05	0.000105
C330027C(	-0.22003	0.890358	-3.79546	0.000858	0.00256
Impa2	-0.2204	0.691886	-3.84031	0.000766	0.002315
Mrpl13	-0.22064	1.802707	-4.41153	0.000178	0.000621
Otulin	-0.22076	2.01294	-6.34565	1.33E-06	7.36E-06
LOC10263	-0.2208	0.986582	-3.80398	0.00084	0.002519
Lyst	-0.22096	2.338217	-4.67524	9.04E-05	0.000338
Tkt	-0.22102	1.421062	-4.19902	0.000307	0.001016
Fkbp15	-0.22135	1.637856	-4.67905	8.95E-05	0.000335
Cd53	-0.22145	2.297325	-4.02112	0.000484	0.001527
Rpl15	-0.22156	1.314345	-3.35897	0.002557	0.006743
Sipa1l3	-0.22195	1.460726	-4.80107	6.54E-05	0.000253
Celf2	-0.22208	1.451398	-4.82639	6.13E-05	0.000239
5830428M	-0.22213	0.868806	-3.75666	0.000946	0.002775
C920009B:	-0.22217	0.779982	-3.85091	0.000746	0.002266
Prpf38b	-0.22265	2.285567	-3.55694	0.001563	0.004332
Ankrd50	-0.22282	0.872335	-4.79135	6.71E-05	0.000258
Ltbr	-0.22303	0.994265	-3.72909	0.001015	0.002957
Rnf144b	-0.22359	1.433468	-5.18743	2.43E-05	0.000103
Gdpd5	-0.22366	0.615744	-5.20355	2.33E-05	9.95E-05
Tab2	-0.22384	2.551284	-6.15846	2.11E-06	1.11E-05
Ifnar2	-0.22384	2.444731	-4.70836	8.30E-05	0.000312
Ddi2	-0.22457	1.509861	-4.76228	7.23E-05	0.000275
Cdca2	-0.22485	0.619909	-4.4112	0.000178	0.000621
Casp7	-0.22497	1.155755	-4.84144	5.90E-05	0.000231
Haus4	-0.22517	0.897459	-5.76419	5.64E-06	2.73E-05
Orm2	-0.22532	1.330564	-2.95934	0.006741	0.015936
LOC10263	-0.22539	0.579654	-2.63473	0.014376	0.030667
Macf1	-0.22581	1.710409	-4.47282	0.000152	0.000539
Cytip	-0.22586	2.015973	-4.50246	0.000141	0.000504
9030617O	-0.22594	1.438417	-5.66693	7.20E-06	3.42E-05
Nfkbiz	-0.22607	1.834445	-3.85464	0.000739	0.002249
Rel	-0.2272	2.775448	-4.40255	0.000182	0.000634
Cdkn3	-0.22792	0.679547	-3.6397	0.001271	0.003608
Gbp9	-0.22794	2.436449	-3.88124	0.00069	0.002118
Mapk8	-0.22815	1.37362	-5.10958	2.97E-05	0.000123
Ifi203	-0.22822	1.682782	-4.41551	0.000176	0.000616
Herc6	-0.22823	1.647049	-5.6312	7.88E-06	3.70E-05
Ddb2	-0.22841	1.055326	-4.2055	0.000302	0.001001
Zfp961	-0.22888	0.94574	-4.91683	4.86E-05	0.000193
Lpar6	-0.22891	1.255075	-4.09774	0.000398	0.001284
Snord22	-0.22906	1.468679	-3.26589	0.003214	0.008255

Tmem206	-0.22906	1.120086	-4.22031	0.000291	0.000968
Snord65	-0.22919	2.136066	-2.8961	0.007832	0.018143
Cdc45	-0.22924	0.695005	-5.84796	4.57E-06	2.26E-05
Pvt1	-0.22947	0.365691	-2.95047	0.006885	0.016227
Cd68	-0.22974	2.269694	-4.50324	0.000141	0.000504
D8Ertd738	-0.22979	2.507223	-4.08628	0.00041	0.001316
Slfn5	-0.22983	1.223403	-4.62904	0.000102	0.000375
Dut	-0.23011	0.907184	-3.53304	0.00166	0.004568
Dpp4	-0.23071	2.394617	-4.56696	0.000119	0.000434
Skp2	-0.23081	0.701166	-4.47037	0.000153	0.000542
Zfand5	-0.23123	2.069575	-5.69001	6.80E-06	3.25E-05
Chd9	-0.23279	1.322979	-5.09267	3.10E-05	0.000128
Donson	-0.23289	1.326401	-4.28476	0.000246	0.000835
Rab43	-0.23317	1.754489	-5.14417	2.72E-05	0.000114
Dhfr	-0.23339	0.401236	-4.266	0.000259	0.000872
Dbf4	-0.23365	1.107312	-4.97956	4.14E-05	0.000166
Slfn2	-0.23385	2.157585	-4.9105	4.94E-05	0.000196
Cul3	-0.23402	2.375341	-6.44529	1.04E-06	5.84E-06
Fli1	-0.23432	1.518236	-4.28388	0.000247	0.000836
Cd52	-0.2346	3.484752	-5.59283	8.69E-06	4.04E-05
Arhgap11a	-0.23467	1.361092	-4.31235	0.00023	0.000782
Map3k14	-0.23504	1.449577	-4.55983	0.000122	0.000441
Heatr5a	-0.23598	0.973612	-5.68865	6.82E-06	3.25E-05
Slc7a7	-0.23618	1.216928	-4.22803	0.000285	0.000952
Plxnc1	-0.23659	2.997345	-5.71849	6.33E-06	3.04E-05
Dock4	-0.2367	0.691479	-4.43322	0.000168	0.000591
Arhgef2	-0.23675	1.57723	-6.02875	2.91E-06	1.49E-05
Chaf1a	-0.23701	0.654714	-4.98493	4.08E-05	0.000164
Cd82	-0.23729	1.299074	-5.15745	2.63E-05	0.00011
Stat2	-0.23766	1.549203	-4.97513	4.19E-05	0.000168
Uhrf1	-0.2377	1.279851	-3.87078	0.000709	0.002169
Ranbp2	-0.2378	1.984303	-5.19421	2.39E-05	0.000101
Ampd3	-0.23829	0.837839	-6.59918	7.17E-07	4.17E-06
Sp140	-0.23852	1.247514	-3.25632	0.00329	0.00843
Exosc5	-0.23892	1.017956	-4.09453	0.000401	0.001293
Atp1a3	-0.23899	0.370536	-3.69686	0.0011	0.003176
Atad5	-0.23944	0.707366	-3.81916	0.000808	0.002434
Ppdpf	-0.2398	0.724175	-2.7933	0.009969	0.022377
Setbp1	-0.23997	0.416247	-3.97464	0.000544	0.001698
Rere	-0.23999	1.281516	-3.87004	0.00071	0.002171
Ep300	-0.2405	2.337509	-6.76947	4.75E-07	2.86E-06
Evi2a	-0.24061	2.086899	-4.48834	0.000146	0.00052
Atp13a3	-0.24082	1.427391	-5.01852	3.75E-05	0.000153
B9d2	-0.24144	0.869715	-4.29197	0.000242	0.000821
Rbbp7	-0.24153	2.201163	-5.923	3.79E-06	1.91E-05
Lpcat1	-0.24163	1.915761	-4.61376	0.000106	0.000388
Cdc6	-0.24165	0.502644	-5.60063	8.52E-06	3.97E-05
Nup160	-0.24206	1.195155	-5.62984	7.91E-06	3.71E-05
Osgin2	-0.24206	1.02239	-3.70836	0.001069	0.00309
Cenph	-0.24209	0.618943	-4.2217	0.00029	0.000965
Clasp2	-0.24214	1.415905	-5.8905	4.11E-06	2.05E-05
Acap2	-0.24267	1.93669	-4.78078	6.89E-05	0.000264

Mink1	-0.24274	1.670921	-5.64383	7.64E-06	3.60E-05
Bora	-0.24323	0.569428	-4.18554	0.000318	0.001048
Wdhd1	-0.24375	0.943438	-4.34634	0.00021	0.000721
Usp1	-0.24399	1.194483	-3.84637	0.000754	0.002289
Usp12	-0.2442	1.684619	-5.54656	9.76E-06	4.49E-05
Zfp770	-0.24435	1.425641	-3.56004	0.001551	0.004304
Wdr76	-0.24466	0.760263	-5.17815	2.49E-05	0.000105
Helz2	-0.24477	0.840931	-4.26848	0.000257	0.000868
A930012O	-0.24493	0.66517	-3.46807	0.001951	0.005281
Sh3pxd2a	-0.24494	1.110482	-5.16424	2.58E-05	0.000109
Lims1	-0.24503	1.439233	-7.14478	1.94E-07	1.26E-06
Sgms1	-0.24543	1.056124	-4.84083	5.91E-05	0.000231
Akap10	-0.24546	1.176662	-5.40673	1.39E-05	6.23E-05
Usp48	-0.24549	1.594335	-6.17451	2.03E-06	1.07E-05
Samd9l	-0.24562	1.561774	-4.36926	0.000198	0.000683
Gabbr1	-0.24562	0.780723	-4.07696	0.000419	0.001342
Bin1	-0.24567	1.119111	-5.00255	3.90E-05	0.000158
Trim37	-0.2458	0.933811	-4.37052	0.000198	0.000681
Gm20324	-0.24606	0.713672	-4.17274	0.000328	0.001078
Tgs1	-0.24672	1.720561	-4.85128	5.75E-05	0.000225
Spred2	-0.24673	0.985174	-4.77538	6.99E-05	0.000268
Lig1	-0.24674	1.265425	-5.9913	3.20E-06	1.63E-05
Pyhin1	-0.24683	2.143197	-4.58192	0.000115	0.000419
Exosc8	-0.24701	1.498463	-5.23228	2.17E-05	9.32E-05
Lpin2	-0.24729	1.160238	-5.40297	1.40E-05	6.28E-05
Mir1198	-0.24738	1.171213	-3.39882	0.002317	0.006159
Ccne2	-0.24753	0.76901	-5.56515	9.31E-06	4.32E-05
Napg	-0.24757	1.21818	-5.00907	3.84E-05	0.000156
Chuk	-0.2478	1.67441	-6.49259	9.29E-07	5.24E-06
Cdca7l	-0.24794	0.764773	-5.92276	3.79E-06	1.91E-05
Per1	-0.24849	1.267463	-3.71856	0.001042	0.003028
Samd1	-0.24876	1.272672	-3.93326	0.000605	0.001875
Nup210	-0.24934	2.005642	-6.83775	4.03E-07	2.46E-06
Gla	-0.24961	0.825675	-3.78542	0.00088	0.002614
Slc3a2	-0.24989	1.855103	-5.73844	6.02E-06	2.90E-05
LOC10263	-0.25069	0.88909	-2.70806	0.012149	0.026541
Rev1	-0.25116	0.957858	-5.21491	2.27E-05	9.71E-05
Tbxas1	-0.25131	0.461582	-5.5132	1.06E-05	4.84E-05
Snx30	-0.25174	0.958526	-4.76907	7.10E-05	0.000271
Tyms	-0.25275	0.991166	-3.35582	0.002577	0.006786
Slc38a1	-0.25289	1.394767	-3.16031	0.004158	0.010392
Cyba	-0.25369	2.625777	-5.20019	2.35E-05	0.0001
Ahr	-0.25407	1.302945	-3.90277	0.000654	0.002015
Man2b1	-0.25409	2.335373	-6.56557	7.78E-07	4.49E-06
Chm	-0.2543	1.544542	-5.1504	2.67E-05	0.000112
Gmnn	-0.25439	1.239902	-3.81953	0.000807	0.002433
Trafd1	-0.25514	2.265383	-6.42951	1.08E-06	6.06E-06
Xpr1	-0.2556	1.31486	-6.13796	2.22E-06	1.16E-05
Cdk2	-0.2558	0.886074	-4.52487	0.000133	0.000478
Snord17	-0.25583	1.312233	-3.44139	0.002085	0.005602
Notch2	-0.25665	1.868851	-6.67457	5.97E-07	3.52E-06
Arl6ip5	-0.25674	1.651687	-5.39081	1.45E-05	6.45E-05

Wdr11	-0.25778	0.936611	-6.91435	3.36E-07	2.08E-06
Lrp1	-0.25821	0.396657	-4.86192	5.60E-05	0.00022
Rbl1	-0.2584	1.45174	-6.38046	1.22E-06	6.79E-06
Myadm	-0.25897	1.928806	-5.68816	6.83E-06	3.26E-05
Alg8	-0.25942	1.454375	-4.81918	6.25E-05	0.000243
Pik3cg	-0.25983	1.445862	-4.19895	0.000307	0.001016
Parp12	-0.25992	1.454616	-4.77041	7.08E-05	0.00027
Rasgrp4	-0.25995	1.537478	-4.44145	0.000165	0.00058
Cap1	-0.26052	2.824796	-6.22142	1.81E-06	9.67E-06
Slc11a1	-0.26062	0.391975	-4.08191	0.000414	0.001328
Lrp5	-0.26081	0.671187	-6.88765	3.58E-07	2.20E-06
H2-Ke6	-0.26171	1.559191	-5.70085	6.61E-06	3.17E-05
Smarca2	-0.26177	1.66338	-6.1427	2.19E-06	1.15E-05
Tti1	-0.26245	1.018883	-4.89463	5.15E-05	0.000204
Gngt2	-0.26253	1.215863	-5.06392	3.34E-05	0.000137
Oma1	-0.26276	1.35704	-6.60493	7.07E-07	4.12E-06
Spag5	-0.26285	0.505531	-6.54639	8.15E-07	4.67E-06
Mtr	-0.26365	0.761303	-4.21513	0.000295	0.00098
Pbk	-0.26371	0.631709	-4.71108	8.25E-05	0.00031
Nadk	-0.26392	1.346755	-6.0291	2.91E-06	1.49E-05
Hist1h4i	-0.26395	0.844297	-2.92326	0.007344	0.017168
Ticam2	-0.26428	0.656967	-4.77356	7.02E-05	0.000269
Srebf2	-0.26444	2.024944	-6.99763	2.75E-07	1.74E-06
Skap2	-0.26482	2.476913	-7.94837	3.04E-08	2.31E-07
Rabggta	-0.26483	0.707394	-5.81601	4.95E-06	2.42E-05
Ube2n	-0.26485	0.733005	-3.58967	0.00144	0.004023
Med30	-0.26518	1.292314	-4.99685	3.96E-05	0.00016
Cbfb	-0.26525	2.007915	-6.31555	1.43E-06	7.84E-06
Clec4b1	-0.26534	0.418858	-4.94275	4.55E-05	0.000182
Nif3l1	-0.26693	0.849401	-3.29745	0.002975	0.007717
BC055324	-0.26728	0.598624	-4.47375	0.000152	0.000538
Rab19	-0.26765	0.620263	-4.38781	0.000189	0.000656
Zfp263	-0.26819	1.027178	-5.21449	2.27E-05	9.71E-05
Cdon	-0.26866	1.014964	-4.17976	0.000322	0.001062
A530032D	-0.26878	1.624945	-4.02696	0.000476	0.001507
Olfr98	-0.26906	1.313534	-3.1756	0.004006	0.010064
Lcor	-0.26912	1.56795	-7.17218	1.82E-07	1.19E-06
Ivns1abp	-0.26936	2.560541	-7.36497	1.16E-07	7.87E-07
Fam49b	-0.26965	2.201287	-5.89559	4.06E-06	2.03E-05
Tet2	-0.27025	1.676556	-4.66259	9.34E-05	0.000348
Abhd17c	-0.27039	1.03215	-4.77155	7.06E-05	0.00027
Kdm7a	-0.27045	1.988986	-7.44177	9.70E-08	6.68E-07
Ccser1	-0.27062	0.636853	-5.14882	2.68E-05	0.000113
Pole	-0.27082	0.557462	-5.85741	4.46E-06	2.22E-05
Il18	-0.27082	1.138907	-4.43268	0.000169	0.000592
Zcchc2	-0.27089	0.909346	-6.27151	1.60E-06	8.64E-06
Jund	-0.27112	1.151228	-3.79135	0.000867	0.002581
Dock11	-0.27113	2.09338	-6.99909	2.74E-07	1.73E-06
Ncapg2	-0.27209	0.988798	-6.29982	1.49E-06	8.12E-06
Rmrp	-0.27277	2.356404	-2.81515	0.009473	0.021373
Ncf1	-0.27277	1.769724	-7.35969	1.17E-07	7.96E-07
LOC10263	-0.27294	0.872941	-2.82515	0.009254	0.020942

Gatm	-0.27359	0.753514	-5.56375	9.35E-06	4.33E-05
Dtl	-0.27374	0.687152	-5.241	2.12E-05	9.14E-05
Fignl1	-0.27412	0.482261	-4.38307	0.000191	0.000664
Atf6	-0.27417	1.713433	-5.96547	3.41E-06	1.73E-05
Gpsm3	-0.27427	1.456723	-5.63936	7.72E-06	3.63E-05
Rnf130	-0.27427	1.904161	-5.68248	6.93E-06	3.30E-05
Phtf2	-0.2744	0.859277	-5.30059	1.82E-05	7.95E-05
Trim12a	-0.27495	0.893848	-3.10808	0.004719	0.011657
Ifitm1	-0.27516	0.335675	-4.32188	0.000224	0.000765
4632427E1	-0.27518	1.790716	-5.21985	2.24E-05	9.59E-05
Fam69a	-0.27522	1.820513	-5.5113	1.07E-05	4.87E-05
Irak2	-0.27588	1.168286	-5.75251	5.81E-06	2.80E-05
Plekho2	-0.27607	1.895865	-6.69041	5.75E-07	3.39E-06
Abca3	-0.27613	1.054417	-6.90446	3.44E-07	2.12E-06
Ska3	-0.27641	0.451577	-4.58946	0.000113	0.000411
Cep83	-0.27643	1.635408	-5.77414	5.50E-06	2.66E-05
Rab8a	-0.27646	2.123487	-6.20628	1.87E-06	1.00E-05
LOC10263	-0.27656	0.752597	-3.64132	0.001265	0.003595
Cdca7	-0.2766	0.406049	-4.01956	0.000486	0.001532
Cdc20	-0.27679	0.758277	-5.55748	9.50E-06	4.39E-05
Foxm1	-0.27796	0.670599	-5.69643	6.69E-06	3.20E-05
Notch4	-0.27798	0.670845	-6.79442	4.47E-07	2.70E-06
LOC10263	-0.27888	0.692305	-3.20758	0.003706	0.00939
Ms4a4c	-0.27963	0.985629	-3.506	0.001775	0.004846
Ksr1	-0.27994	0.535524	-4.71107	8.25E-05	0.00031
Mir3064	-0.2801	3.855283	-4.66555	9.27E-05	0.000345
Setd1b	-0.28107	1.130201	-5.82258	4.87E-06	2.39E-05
Hist1h1a	-0.28111	0.595814	-5.32092	1.73E-05	7.58E-05
Baz1a	-0.28159	1.76977	-7.07281	2.30E-07	1.48E-06
Tmem141	-0.2834	0.807949	-4.01631	0.00049	0.001541
Traf3ip3	-0.28386	1.157213	-4.3052	0.000234	0.000796
Acsf2	-0.28399	0.577109	-5.82118	4.89E-06	2.39E-05
Tcf7l2	-0.28444	1.232292	-4.73617	7.73E-05	0.000293
Lbr	-0.28484	0.849181	-5.21457	2.27E-05	9.71E-05
Mis18bp1	-0.28528	0.638149	-6.64964	6.34E-07	3.72E-06
Rnd3	-0.28545	2.006204	-3.6807	0.001146	0.003294
Atad2	-0.28606	1.065821	-6.66191	6.16E-07	3.62E-06
Cyp27a1	-0.28617	1.930102	-6.47569	9.68E-07	5.45E-06
Wipf1	-0.28639	1.422388	-6.88023	3.64E-07	2.24E-06
4933426M	-0.2866	1.061389	-5.38211	1.48E-05	6.59E-05
Rbm38	-0.28815	1.172523	-5.33021	1.69E-05	7.43E-05
Arhgap4	-0.28872	0.997303	-6.74677	5.02E-07	3.00E-06
Hist1h1c	-0.28874	1.688628	-2.83748	0.00899	0.020426
Acap1	-0.28895	0.86453	-4.2845	0.000247	0.000835
Plcl2	-0.28905	1.538315	-5.50116	1.10E-05	4.99E-05
Nelfe	-0.28913	1.279728	-6.76225	4.83E-07	2.90E-06
Lrmp	-0.28919	1.697221	-4.82546	6.15E-05	0.000239
Ttk	-0.29006	0.501124	-6.36013	1.28E-06	7.12E-06
Errfi1	-0.29011	0.68448	-6.15442	2.13E-06	1.12E-05
Crlf3	-0.29049	1.649143	-6.28446	1.55E-06	8.39E-06
Spire1	-0.29061	1.141672	-7.65071	5.99E-08	4.32E-07
Junb	-0.29092	2.533521	-5.84189	4.64E-06	2.29E-05

Sh3bgrl	-0.29127	1.70579	-4.94105	4.57E-05	0.000183
Dexi	-0.29131	0.961507	-4.32451	0.000223	0.00076
Tmem156	-0.29152	0.769538	-4.44197	0.000165	0.000579
Gba2	-0.29235	0.940582	-6.15418	2.13E-06	1.12E-05
Baz2b	-0.29241	1.987453	-6.77975	4.63E-07	2.79E-06
Csk	-0.2929	1.477364	-5.84686	4.58E-06	2.27E-05
Trps1	-0.29292	1.297047	-6.18347	1.98E-06	1.05E-05
Zfp36l2	-0.29302	1.863903	-4.86759	5.52E-05	0.000217
Ganc	-0.29317	0.943962	-6.66152	6.16E-07	3.62E-06
Camkmt	-0.29358	0.628598	-5.68218	6.93E-06	3.30E-05
Slc43a2	-0.2937	0.626503	-6.27171	1.60E-06	8.64E-06
Dclre1b	-0.29481	0.665467	-5.64941	7.53E-06	3.56E-05
Spc25	-0.29498	0.698726	-5.17935	2.48E-05	0.000105
St6gal1	-0.29627	1.62526	-6.20478	1.88E-06	1.00E-05
Polr1a	-0.29631	0.995348	-8.05953	2.37E-08	1.85E-07
Rnu11	-0.29674	2.031396	-5.16723	2.56E-05	0.000108
Fnbp1	-0.29691	2.462156	-7.37896	1.12E-07	7.66E-07
Sort1	-0.29704	1.493485	-6.50608	8.99E-07	5.10E-06
Ccnb1	-0.29739	0.923789	-4.16797	0.000332	0.001088
Bcl2a1a	-0.2974	2.804415	-5.34945	1.61E-05	7.10E-05
5530601H(	-0.29741	0.852476	-6.95831	3.02E-07	1.90E-06
Sik1	-0.2982	1.441512	-4.40749	0.00018	0.000627
Tmem68	-0.29842	1.340972	-6.62743	6.69E-07	3.91E-06
Ciita	-0.29953	1.492966	-6.2862	1.54E-06	8.36E-06
Bin3	-0.29996	1.947304	-5.43427	1.30E-05	5.83E-05
Polg2	-0.30001	1.136507	-4.86641	5.53E-05	0.000218
Ptpn6	-0.30015	2.419572	-6.38058	1.22E-06	6.79E-06
Ppp1r12b	-0.30028	0.647711	-7.82729	4.00E-08	2.99E-07
Tipin	-0.30032	0.997619	-4.63555	0.0001	0.000371
Mcm4	-0.30095	1.520033	-5.80136	5.14E-06	2.51E-05
Arid5b	-0.3016	0.713427	-4.7152	8.16E-05	0.000308
Cd300a	-0.30184	0.643598	-4.84306	5.88E-05	0.00023
LOC10263(	-0.30226	2.782429	-4.97646	4.17E-05	0.000168
4933416M	-0.30287	0.999873	-4.45202	0.00016	0.000566
Ptpn3	-0.30301	0.924304	-6.17134	2.04E-06	1.08E-05
Prim1	-0.30331	0.74921	-4.99748	3.95E-05	0.00016
Dnmt3a	-0.30338	1.0203	-5.27053	1.97E-05	8.52E-05
Stx16	-0.30385	1.641194	-6.00869	3.06E-06	1.56E-05
Tlr3	-0.30478	1.184724	-3.56199	0.001544	0.004287
Fnip1	-0.30479	1.070632	-6.14989	2.16E-06	1.13E-05
Psd4	-0.30495	1.448369	-6.20308	1.89E-06	1.01E-05
Trim21	-0.30514	1.282345	-6.92058	3.31E-07	2.06E-06
Agpat3	-0.30526	1.136542	-5.82789	4.81E-06	2.36E-05
Fos	-0.30569	2.31755	-6.5862	7.40E-07	4.29E-06
Gnaq	-0.30574	1.473857	-5.69742	6.67E-06	3.19E-05
Lfng	-0.30575	1.509584	-6.09429	2.47E-06	1.28E-05
Kif14	-0.30616	0.43722	-7.20575	1.68E-07	1.11E-06
Mov10	-0.30619	0.950056	-5.14589	2.70E-05	0.000113
Pdlim2	-0.30664	0.952476	-7.27087	1.44E-07	9.64E-07
Meis1	-0.30727	0.506363	-5.66926	7.16E-06	3.40E-05
Prkar2a	-0.30747	1.112532	-4.5923	0.000112	0.000409
Cks1b	-0.30807	1.744917	-3.85251	0.000743	0.002259

Zfp366	-0.30882	2.087226	-6.53524	8.37E-07	4.79E-06
Slc4a7	-0.30907	0.935907	-4.24711	0.000271	0.000911
Snord49b	-0.30908	1.358134	-4.14687	0.000351	0.001144
Cass4	-0.30955	0.495844	-5.29953	1.83E-05	7.97E-05
Milr1	-0.30991	0.735056	-4.63168	0.000101	0.000373
Trim12c	-0.30995	1.455616	-5.82615	4.83E-06	2.37E-05
Mir23a	-0.31047	0.805735	-2.65847	0.013616	0.02924
Plxnd1	-0.31076	1.270424	-6.06522	2.66E-06	1.37E-05
Tgfb1	-0.31132	1.841659	-5.9739	3.34E-06	1.69E-05
Cdca3	-0.3115	0.730367	-4.76238	7.23E-05	0.000275
Gpr132	-0.312	2.088612	-5.98968	3.21E-06	1.63E-05
Glipr1	-0.31208	2.260803	-5.60242	8.48E-06	3.96E-05
Plk1	-0.31217	0.688891	-6.27129	1.60E-06	8.64E-06
Il10ra	-0.31405	2.041226	-6.89595	3.51E-07	2.16E-06
Wdr41	-0.31427	1.029149	-5.9387	3.64E-06	1.84E-05
Snora31	-0.31472	2.738419	-3.85794	0.000732	0.002233
Chek1	-0.31494	0.511354	-6.03968	2.83E-06	1.46E-05
Ptprj	-0.31586	2.000709	-7.65699	5.90E-08	4.27E-07
Zcchc11	-0.31618	1.590163	-7.48565	8.76E-08	6.09E-07
Gpr65	-0.3162	2.160709	-6.90451	3.44E-07	2.12E-06
Plcg2	-0.31647	1.441904	-6.9181	3.33E-07	2.07E-06
Abr	-0.31669	1.395417	-8.61988	6.91E-09	6.07E-08
Hist2h2ac	-0.31697	1.269482	-3.12387	0.004542	0.011258
Morc2a	-0.31757	1.292821	-8.39297	1.13E-08	9.41E-08
Hsh2d	-0.31809	1.299955	-5.57553	9.07E-06	4.21E-05
Tubb6	-0.31907	0.992119	-5.48337	1.15E-05	5.21E-05
Hist1h2bl	-0.31969	2.592843	-5.05004	3.46E-05	0.000141
Scarna6	-0.31978	0.748275	-2.72855	0.011588	0.025517
Il1r2	-0.31981	0.471648	-4.65486	9.53E-05	0.000354
Prr11	-0.31994	0.988459	-4.68442	8.83E-05	0.000331
Mcm5	-0.32085	0.891809	-5.8019	5.13E-06	2.50E-05
Adcy7	-0.32087	0.892781	-5.84658	4.59E-06	2.27E-05
Scarna3b	-0.32092	1.351495	-3.19983	0.003777	0.009546
Tpx2	-0.32107	0.817847	-5.32336	1.72E-05	7.54E-05
Smc4	-0.32109	2.190796	-7.66039	5.86E-08	4.25E-07
4632428N(	-0.32125	1.173515	-8.37717	1.17E-08	9.67E-08
LOC10263:	-0.32152	0.620892	-4.55857	0.000122	0.000442
Slc35c2	-0.32155	1.100968	-6.2458	1.70E-06	9.16E-06
Klra5	-0.32333	0.24574	-4.87271	5.44E-05	0.000215
Hist1h2af	-0.32418	1.020574	-5.52701	1.03E-05	4.69E-05
Adap1	-0.32437	1.227568	-5.85958	4.44E-06	2.21E-05
Stk10	-0.3245	1.2646	-6.18325	1.98E-06	1.05E-05
Cerk	-0.32517	0.787671	-7.19042	1.74E-07	1.15E-06
Jag1	-0.32594	1.165815	-5.52442	1.03E-05	4.72E-05
Sh3bp2	-0.32605	0.923184	-7.55774	7.42E-08	5.25E-07
Foxn3	-0.32664	1.110039	-7.00835	2.68E-07	1.70E-06
Rab11fip1	-0.32664	1.269126	-7.30553	1.33E-07	8.96E-07
Zfp831	-0.32683	0.386168	-4.81971	6.24E-05	0.000242
Dram2	-0.32687	1.287604	-4.63159	0.000101	0.000373
Supt20	-0.32769	2.383818	-9.22203	1.92E-09	1.89E-08
4930592I0	-0.32819	0.572107	-5.08375	3.17E-05	0.000131
Rrm1	-0.32891	1.256896	-5.86429	4.39E-06	2.18E-05

Phf11b	-0.32914	2.788582	-5.07204	3.27E-05	0.000134
Wdr86	-0.32988	0.759106	-5.55012	9.68E-06	4.46E-05
Gm2a	-0.3299	2.528363	-6.77516	4.69E-07	2.82E-06
4921511I1	-0.3318	1.352743	-4.62067	0.000104	0.000383
Bin2	-0.33188	1.257703	-5.06227	3.35E-05	0.000137
Gpd2	-0.3324	1.368757	-6.9165	3.34E-07	2.07E-06
Rnf145	-0.33241	1.368295	-7.27627	1.43E-07	9.55E-07
Lsm6	-0.333	1.175203	-5.41713	1.36E-05	6.07E-05
Taf7	-0.33308	1.149969	-7.57369	7.15E-08	5.08E-07
Nusap1	-0.33418	0.866568	-5.14306	2.72E-05	0.000114
Tex9	-0.33464	0.785497	-3.60216	0.001396	0.003911
Phc1	-0.33488	1.00449	-5.77528	5.49E-06	2.66E-05
Tln1	-0.33497	1.871453	-9.41136	1.30E-09	1.33E-08
Dusp16	-0.33508	1.202608	-7.59564	6.80E-08	4.86E-07
Ect2	-0.33606	0.622411	-6.75969	4.86E-07	2.92E-06
Nfatc2	-0.33761	1.231246	-7.54923	7.56E-08	5.35E-07
Tmem170b	-0.33936	0.797293	-6.26668	1.62E-06	8.73E-06
H2afx	-0.33999	1.973976	-5.17702	2.50E-05	0.000105
Cenpk	-0.34024	0.658656	-5.34931	1.61E-05	7.10E-05
Syk	-0.34026	1.097711	-7.84343	3.86E-08	2.89E-07
Aspm	-0.34026	0.518803	-6.37113	1.25E-06	6.94E-06
Asrgl1	-0.34036	0.821571	-5.94668	3.57E-06	1.81E-05
Ccdc102a	-0.34101	0.566627	-6.31566	1.43E-06	7.84E-06
Dlgap5	-0.34157	0.803115	-8.65173	6.45E-09	5.72E-08
Csf2ra	-0.34236	1.256976	-7.09283	2.20E-07	1.41E-06
Plau	-0.34291	0.484667	-7.46218	9.25E-08	6.41E-07
Ptpn12	-0.34293	1.61028	-7.95799	2.98E-08	2.26E-07
App	-0.34341	1.76831	-7.56193	7.35E-08	5.21E-07
Btg1	-0.34348	2.414338	-6.7064	5.53E-07	3.28E-06
Prkcb	-0.34379	1.329836	-6.02396	2.95E-06	1.51E-05
Kpna4	-0.34419	2.094995	-9.81648	5.69E-10	6.24E-09
Tob2	-0.34555	1.084378	-5.85265	4.52E-06	2.24E-05
Esco1	-0.34677	1.585224	-7.76227	4.64E-08	3.43E-07
Snx13	-0.34698	1.621017	-8.5683	7.73E-09	6.73E-08
Tmpo	-0.34794	1.482743	-8.52832	8.43E-09	7.24E-08
P2rx7	-0.34837	0.718273	-8.01848	2.60E-08	2.02E-07
Glud1	-0.34904	2.624196	-6.15333	2.14E-06	1.12E-05
AB124611	-0.34926	1.384052	-4.81057	6.39E-05	0.000247
Hipk1	-0.34941	1.823989	-10.9689	6.09E-11	8.20E-10
Dopey1	-0.3495	1.104365	-7.3116	1.31E-07	8.85E-07
Aph1c	-0.34963	1.005055	-3.62631	0.001314	0.003706
Nrm	-0.35053	1.316306	-6.55995	7.88E-07	4.54E-06
Ipo7	-0.35105	1.820366	-7.97503	2.87E-08	2.19E-07
Samhd1	-0.35159	3.157179	-10.1824	2.75E-10	3.27E-09
Napsa	-0.35193	2.232639	-5.08967	3.12E-05	0.000129
Ppt2	-0.35216	1.095567	-4.84259	5.88E-05	0.00023
Ttc39b	-0.35225	1.112645	-6.18732	1.96E-06	1.04E-05
Herc4	-0.35251	1.88017	-8.66666	6.24E-09	5.55E-08
Cdkn1b	-0.35283	1.993111	-7.10359	2.14E-07	1.38E-06
Mef2c	-0.35363	1.00109	-5.88991	4.12E-06	2.05E-05
Ifit1	-0.35426	0.551614	-6.20962	1.86E-06	9.93E-06
Ago2	-0.35486	1.177912	-6.29214	1.52E-06	8.25E-06

Ms4a6b	-0.35498	1.929333	-3.6192	0.001338	0.003768
Gbp8	-0.35504	2.001022	-4.24727	0.000271	0.000911
Vrk2	-0.35522	1.689568	-6.21746	1.82E-06	9.76E-06
Pim1	-0.35568	1.705183	-5.37812	1.50E-05	6.65E-05
Kntc1	-0.35607	0.483043	-7.53681	7.78E-08	5.49E-07
Tbrg3	-0.35656	1.129748	-5.61256	8.26E-06	3.86E-05
Ezr	-0.35659	1.748407	-7.98748	2.79E-08	2.14E-07
Tcp11l1	-0.35707	0.719509	-7.03945	2.49E-07	1.59E-06
Cebpz	-0.3576	1.196478	-7.41692	1.03E-07	7.05E-07
Daglb	-0.35938	0.680472	-6.37733	1.23E-06	6.84E-06
Brip1	-0.35986	0.583658	-6.50117	9.09E-07	5.16E-06
Gm4070	-0.35998	1.39982	-3.87644	0.000699	0.002142
Ndufaf7	-0.36049	0.973896	-6.83584	4.05E-07	2.47E-06
Dck	-0.36061	0.871745	-7.85781	3.74E-08	2.81E-07
Cyld	-0.361	1.913636	-8.39126	1.14E-08	9.44E-08
Itpr3	-0.36283	0.497921	-8.65843	6.36E-09	5.65E-08
Tacc3	-0.3632	1.349475	-5.95313	3.51E-06	1.78E-05
Cenpf	-0.36389	0.548514	-9.15304	2.22E-09	2.15E-08
Dna2	-0.36439	1.167067	-6.78275	4.60E-07	2.77E-06
Rad51	-0.36479	0.550043	-4.61792	0.000105	0.000385
Stom	-0.36492	1.709681	-5.70792	6.50E-06	3.12E-05
Ier5	-0.36516	1.908189	-7.07584	2.29E-07	1.47E-06
Rilpl2	-0.36519	0.840323	-4.79297	6.68E-05	0.000257
Defa25	-0.36611	1.20212	-3.59523	0.001421	0.003972
Cd180	-0.3671	2.135866	-6.25963	1.64E-06	8.88E-06
Mir5107	-0.36735	2.2995	-7.32933	1.26E-07	8.52E-07
Kif15	-0.36765	0.756493	-8.2026	1.72E-08	1.38E-07
Atp8a1	-0.36775	1.451197	-8.32982	1.30E-08	1.07E-07
Snord118	-0.36784	1.567138	-4.50269	0.000141	0.000504
Sap30	-0.36805	1.819717	-6.16028	2.10E-06	1.10E-05
Pygl	-0.36805	1.302325	-5.14564	2.71E-05	0.000113
Ace	-0.36811	0.544477	-3.94324	0.00059	0.00183
Rhof	-0.36813	0.784609	-6.31537	1.43E-06	7.84E-06
Lyn	-0.3682	2.231165	-8.5145	8.68E-09	7.45E-08
Kbtbd7	-0.36854	1.396207	-5.94372	3.60E-06	1.82E-05
Ncapd2	-0.37017	0.929588	-6.33429	1.37E-06	7.53E-06
Plek	-0.37038	2.125675	-6.6522	6.30E-07	3.70E-06
Fndc7	-0.37058	0.742303	-7.80572	4.20E-08	3.13E-07
Pydc4	-0.37058	0.422595	-3.70872	0.001068	0.00309
Sema4d	-0.37189	0.714832	-7.53214	7.87E-08	5.55E-07
Fam114a1	-0.37339	0.809586	-6.79206	4.50E-07	2.72E-06
Clcf1	-0.37358	0.509168	-4.85259	5.73E-05	0.000225
Fyb	-0.37373	2.549645	-8.47246	9.52E-09	8.01E-08
Arf2	-0.3739	1.061852	-4.74753	7.51E-05	0.000285
Eml3	-0.37416	1.1565	-8.57988	7.53E-09	6.57E-08
Tlr13	-0.37434	1.694154	-8.00564	2.68E-08	2.06E-07
Plekha1	-0.37501	1.780948	-11.0344	5.39E-11	7.36E-10
Cdca8	-0.37559	1.027954	-6.24223	1.72E-06	9.23E-06
Ptp4a2	-0.3756	2.32036	-9.74229	6.61E-10	7.10E-09
E2f8	-0.37632	0.737054	-7.77451	4.51E-08	3.35E-07
Aurka	-0.37712	0.77243	-6.81881	4.22E-07	2.57E-06
Ncapg	-0.37726	0.71704	-7.44919	9.53E-08	6.59E-07

Gpr18	-0.37726	1.625974	-6.21336	1.84E-06	9.85E-06
Birc5	-0.37849	0.70376	-6.54166	8.24E-07	4.72E-06
Rap1gds1	-0.37893	1.409293	-8.27824	1.46E-08	1.18E-07
Sorl1	-0.37941	1.196642	-10.4565	1.61E-10	1.99E-09
Hist2h2ab	-0.38105	1.063186	-4.55896	0.000122	0.000442
Sgk1	-0.38111	0.855525	-5.56438	9.33E-06	4.32E-05
Trim5	-0.38183	1.65781	-6.08096	2.56E-06	1.32E-05
Prc1	-0.38256	0.687076	-7.48579	8.76E-08	6.09E-07
Preb	-0.38322	1.395914	-6.79962	4.42E-07	2.68E-06
Ap1s2	-0.38341	0.93708	-8.40544	1.10E-08	9.18E-08
Clspn	-0.38374	0.556292	-6.27546	1.58E-06	8.58E-06
Batf3	-0.38488	2.429385	-5.84362	4.62E-06	2.28E-05
BC037034	-0.38527	0.804191	-8.28814	1.43E-08	1.16E-07
Phf11c	-0.38538	1.04378	-4.46545	0.000155	0.000548
Rras2	-0.38556	1.499594	-7.9782	2.85E-08	2.18E-07
Hist1h1e	-0.38659	0.662451	-5.26062	2.02E-05	8.72E-05
Stim2	-0.38739	1.405946	-10.7951	8.45E-11	1.10E-09
Pcna	-0.38757	2.963121	-7.74693	4.81E-08	3.54E-07
Tbc1d10c	-0.38778	1.057804	-8.94178	3.47E-09	3.26E-08
Nap1l1	-0.38804	2.120218	-9.76746	6.28E-10	6.79E-09
BC064078	-0.38825	1.240741	-6.8484	3.93E-07	2.40E-06
Snx10	-0.39014	1.461679	-7.09834	2.17E-07	1.40E-06
Epb4.1l2	-0.39268	2.10014	-8.40532	1.10E-08	9.18E-08
Filip1l	-0.39285	1.659562	-8.68116	6.05E-09	5.42E-08
Cd4	-0.39385	0.938623	-4.53703	0.000129	0.000465
Cdca5	-0.39386	0.552421	-5.94137	3.62E-06	1.83E-05
Gmip	-0.39391	1.258463	-7.48837	8.71E-08	6.07E-07
Slc9a9	-0.39395	1.416931	-9.341	1.50E-09	1.51E-08
Pik3ap1	-0.39413	1.530576	-9.20825	1.98E-09	1.94E-08
Mir142	-0.39575	1.458118	-4.4137	0.000177	0.000619
Cep55	-0.39618	0.642276	-8.47824	9.40E-09	7.94E-08
Soat1	-0.39665	1.591586	-6.49848	9.15E-07	5.18E-06
Cd244	-0.3967	1.332112	-7.23651	1.57E-07	1.04E-06
Bub1b	-0.39689	0.870165	-7.44871	9.54E-08	6.59E-07
Trim34a	-0.3985	1.552252	-6.9229	3.29E-07	2.05E-06
Mfsd7b	-0.39891	1.266566	-8.00174	2.70E-08	2.08E-07
Clec4n	-0.39961	0.434518	-6.3569	1.29E-06	7.17E-06
Parp14	-0.39985	1.535094	-9.38406	1.37E-09	1.38E-08
Xaf1	-0.40021	1.223884	-7.48322	8.81E-08	6.12E-07
Irs2	-0.40067	1.476624	-6.35525	1.30E-06	7.20E-06
Stil	-0.40075	0.562593	-7.78827	4.38E-08	3.25E-07
4933431G	-0.40103	0.541988	-6.55255	8.03E-07	4.61E-06
Fes	-0.40183	1.199925	-8.54286	8.17E-09	7.06E-08
Lrba	-0.40183	1.756884	-10.3105	2.14E-10	2.57E-09
Arhgap9	-0.4022	1.217904	-8.55984	7.87E-09	6.84E-08
Lta4h	-0.40244	1.293562	-8.00734	2.67E-08	2.06E-07
Sh3bp1	-0.4029	2.087541	-7.14415	1.95E-07	1.26E-06
Ccnd2	-0.4034	0.944936	-4.75532	7.36E-05	0.00028
Cyth4	-0.40378	1.791501	-5.75675	5.75E-06	2.78E-05
Klri2	-0.40477	0.516044	-6.18495	1.98E-06	1.05E-05
Pip4k2a	-0.40525	2.895294	-8.38778	1.15E-08	9.49E-08
Trim59	-0.40532	1.112821	-5.51856	1.05E-05	4.78E-05

Rnu3a	-0.40596	1.335524	-2.90345	0.007697	0.017885
H60b	-0.40677	1.25215	-6.88931	3.56E-07	2.20E-06
Gins1	-0.40706	0.664209	-6.93928	3.16E-07	1.98E-06
Ripk2	-0.40828	0.958862	-6.88314	3.62E-07	2.22E-06
Slc8b1	-0.40872	1.609775	-8.01156	2.64E-08	2.04E-07
Scarna2	-0.40877	0.63935	-2.59633	0.015689	0.033081
Sla	-0.40907	1.823967	-8.293	1.41E-08	1.15E-07
Eps8	-0.41001	1.12667	-8.84265	4.28E-09	3.97E-08
Snora70	-0.41006	0.899345	-5.81906	4.91E-06	2.40E-05
Fbxo5	-0.41015	1.037386	-5.9812	3.28E-06	1.67E-05
Hdac4	-0.41031	0.729156	-11.2536	3.59E-11	5.07E-10
Aim2	-0.41031	1.881963	-8.19125	1.77E-08	1.41E-07
Gdap10	-0.41154	1.918231	-8.08323	2.25E-08	1.76E-07
Nxpe3	-0.41171	0.472477	-8.76622	5.04E-09	4.59E-08
Anxa6	-0.4131	1.983798	-6.24456	1.71E-06	9.18E-06
Rpa2	-0.41359	0.865274	-7.86913	3.64E-08	2.74E-07
Smc2	-0.41381	1.391701	-8.50066	8.95E-09	7.63E-08
Pkmyt1	-0.41401	1.05652	-7.70979	5.23E-08	3.83E-07
Apoe	-0.41404	1.100298	-4.52548	0.000133	0.000477
Ccdc88b	-0.41477	0.808413	-9.01074	3.00E-09	2.85E-08
Tnfsf8	-0.41502	0.218023	-6.70602	5.53E-07	3.28E-06
Anln	-0.41528	0.601484	-9.29292	1.66E-09	1.66E-08
Mir340	-0.41661	0.676684	-4.87913	5.36E-05	0.000211
Ube2c	-0.41702	1.318962	-5.89419	4.07E-06	2.04E-05
Hsd17b4	-0.41715	1.398825	-6.87527	3.69E-07	2.26E-06
Kif20b	-0.41902	0.831983	-9.13525	2.30E-09	2.23E-08
Ccl2	-0.4191	0.618064	-3.50745	0.001769	0.00483
Entpd1	-0.41937	1.11142	-8.61977	6.91E-09	6.07E-08
Hcls1	-0.41954	2.001344	-7.04676	2.45E-07	1.56E-06
Fam111a	-0.4204	1.608522	-7.97801	2.85E-08	2.18E-07
Ptprc	-0.42056	3.046979	-9.67066	7.64E-10	8.14E-09
Malt1	-0.42255	2.189063	-8.62481	6.84E-09	6.04E-08
Eif2ak4	-0.4231	1.019657	-10.7024	1.01E-10	1.29E-09
Lmtk2	-0.42445	1.368463	-9.50389	1.07E-09	1.12E-08
Pla2g7	-0.42477	0.43986	-7.08637	2.23E-07	1.43E-06
Arhgap39	-0.42492	0.958283	-6.93953	3.16E-07	1.98E-06
Arhgap42	-0.42543	0.285677	-6.12175	2.31E-06	1.20E-05
Lysmd2	-0.42642	0.421966	-8.17429	1.84E-08	1.46E-07
Kcnip3	-0.42702	0.887971	-8.27016	1.48E-08	1.20E-07
Itga4	-0.4281	2.559187	-11.4194	2.65E-11	3.90E-10
Abhd1	-0.42818	0.797604	-5.53289	1.01E-05	4.63E-05
Cdk1	-0.42922	1.035717	-5.45826	1.22E-05	5.53E-05
Fam105a	-0.42981	1.655142	-7.22433	1.61E-07	1.07E-06
Limd2	-0.43009	1.485609	-7.04876	2.44E-07	1.55E-06
2810417H	-0.43067	0.988057	-5.01612	3.77E-05	0.000153
Hltf	-0.43197	1.720013	-9.2125	1.96E-09	1.93E-08
Shcbp1	-0.43404	0.596147	-6.5736	7.63E-07	4.41E-06
H2-Oa	-0.43533	2.064039	-9.40449	1.32E-09	1.34E-08
Dennd1c	-0.43636	1.475641	-9.76905	6.26E-10	6.79E-09
Dock10	-0.43644	2.866812	-12.4825	4.08E-12	7.33E-11
Mir680-1	-0.4368	0.739537	-3.89277	0.00067	0.002063
Snord14e	-0.43848	2.442369	-4.19347	0.000311	0.001029

Hmmr	-0.43873	0.649509	-9.26254	1.77E-09	1.75E-08
Rinl	-0.43962	0.73821	-7.44429	9.64E-08	6.65E-07
Cotl1	-0.44122	1.670071	-4.96952	4.25E-05	0.00017
Cd80	-0.44367	1.58506	-6.54367	8.20E-07	4.70E-06
Rnf213	-0.44672	1.777574	-7.49285	8.62E-08	6.02E-07
Stk17b	-0.44705	2.397082	-12.5646	3.55E-12	6.52E-11
Ddx39	-0.44798	1.280814	-8.06801	2.33E-08	1.82E-07
Tmem150b	-0.44806	0.728063	-8.47337	9.50E-09	8.00E-08
Asf1b	-0.44935	0.782031	-7.20349	1.69E-07	1.11E-06
LOC10263	-0.4494	0.937181	-6.90411	3.44E-07	2.12E-06
Rgs12	-0.45003	0.955225	-7.67252	5.70E-08	4.14E-07
Rrad	-0.45023	0.854	-7.27475	1.43E-07	9.57E-07
Slc46a3	-0.45152	2.5596	-10.537	1.38E-10	1.73E-09
Rnf149	-0.45176	0.854351	-6.59615	7.22E-07	4.19E-06
Taldo1	-0.45211	1.906562	-9.86127	5.20E-10	5.77E-09
Cenpe	-0.45249	0.820361	-10.8777	7.23E-11	9.53E-10
Dennd3	-0.45785	1.162657	-7.84457	3.85E-08	2.88E-07
Mns1	-0.45805	0.609569	-8.2992	1.39E-08	1.14E-07
1190002F1	-0.45877	0.610926	-6.48753	9.40E-07	5.31E-06
Tlr2	-0.45926	1.157976	-8.78894	4.80E-09	4.40E-08
Myo9b	-0.4616	1.918588	-9.98975	4.03E-10	4.64E-09
Lpcat2	-0.46193	0.673314	-8.76707	5.03E-09	4.58E-08
Sirpa	-0.46242	1.1075	-5.55705	9.51E-06	4.39E-05
Bub1	-0.46384	0.629195	-8.23965	1.59E-08	1.28E-07
Hlcs	-0.46389	0.615123	-7.83905	3.90E-08	2.91E-07
S100pbp	-0.46425	1.41065	-9.82082	5.64E-10	6.20E-09
Irf5	-0.46433	1.626301	-10.1325	3.04E-10	3.60E-09
Hist1h1d	-0.46461	0.592774	-5.66535	7.23E-06	3.42E-05
Ncaph	-0.46863	0.708232	-7.81553	4.11E-08	3.06E-07
Ebi3	-0.46903	1.906923	-9.48035	1.13E-09	1.17E-08
Fam49a	-0.47007	2.823519	-9.95107	4.35E-10	4.96E-09
Ggta1	-0.4702	1.161546	-8.7895	4.80E-09	4.40E-08
Dusp1	-0.47064	3.075808	-9.07427	2.62E-09	2.53E-08
Kif4	-0.47085	0.542975	-9.27235	1.73E-09	1.72E-08
6330407A(	-0.47094	1.541303	-7.97167	2.89E-08	2.21E-07
Slc14a1	-0.47161	0.831941	-5.42887	1.32E-05	5.91E-05
Neil3	-0.47205	0.520909	-8.73289	5.41E-09	4.91E-08
Klhl18	-0.47319	0.952306	-8.83993	4.30E-09	3.98E-08
LOC10263	-0.47388	0.639143	-6.13863	2.22E-06	1.16E-05
Csgalnact2	-0.47516	1.187519	-9.91296	4.69E-10	5.27E-09
Abca9	-0.47527	0.33869	-7.15088	1.92E-07	1.25E-06
Gpr35	-0.47762	0.916683	-8.81715	4.52E-09	4.17E-08
Siglecg	-0.47924	1.206511	-8.53189	8.36E-09	7.20E-08
Lpxn	-0.47926	0.857559	-9.07319	2.63E-09	2.53E-08
Ggct	-0.48111	0.80329	-6.5184	8.72E-07	4.95E-06
Emb	-0.48131	1.332429	-6.01238	3.03E-06	1.55E-05
Actr1b	-0.48401	0.833585	-8.43185	1.04E-08	8.69E-08
Ccnb2	-0.48405	1.072059	-6.44588	1.04E-06	5.84E-06
Itgb2	-0.4843	1.899868	-8.5342	8.32E-09	7.18E-08
Pik3cb	-0.48551	2.285354	-12.4959	3.99E-12	7.20E-11
Nuf2	-0.48653	0.811411	-8.61827	6.93E-09	6.09E-08
Ms4a7	-0.48682	0.240227	-9.23232	1.88E-09	1.86E-08

Rapgef1	-0.48884	1.360828	-11.0181	5.56E-11	7.56E-10
Spint1	-0.49135	0.849368	-6.90602	3.42E-07	2.12E-06
Ear2	-0.49186	1.125269	-4.39429	0.000186	0.000646
Arhgap15	-0.49412	1.544258	-6.52687	8.54E-07	4.88E-06
Nfatc1	-0.49415	1.296915	-11.9006	1.12E-11	1.83E-10
Sh2d3c	-0.49496	0.955074	-9.03406	2.85E-09	2.72E-08
Tnfrsf1a	-0.495	1.324205	-7.23223	1.58E-07	1.05E-06
Pmaip1	-0.4953	1.57188	-7.41569	1.03E-07	7.06E-07
Slfn8	-0.49629	1.033904	-4.61921	0.000104	0.000384
Vav2	-0.49674	1.066242	-11.3258	3.15E-11	4.53E-10
Hist1h2an	-0.49743	1.407834	-6.11863	2.33E-06	1.21E-05
Gm15133	-0.49781	0.689118	-6.61273	6.94E-07	4.04E-06
Bcl2a1b	-0.49828	2.780506	-8.13534	2.00E-08	1.58E-07
Hist1h2bn	-0.49914	2.675931	-6.34236	1.34E-06	7.40E-06
Sat1	-0.49945	2.903653	-6.29717	1.50E-06	8.17E-06
Bcl9	-0.50077	1.530195	-9.97144	4.18E-10	4.79E-09
Gm1966	-0.50154	1.332272	-9.89044	4.91E-10	5.49E-09
Prkch	-0.50223	1.345616	-10.4048	1.78E-10	2.18E-09
Ier2	-0.50408	1.434107	-7.63407	6.22E-08	4.48E-07
Mtpn	-0.50582	2.575468	-13.799	4.69E-13	1.08E-11
Immp2l	-0.50794	0.523137	-9.1835	2.08E-09	2.03E-08
Hist1h2bf	-0.50841	1.835364	-5.98516	3.24E-06	1.65E-05
Camk2d	-0.50916	0.916307	-9.71232	7.02E-10	7.51E-09
Fosb	-0.50952	2.281704	-11.5636	2.04E-11	3.10E-10
Nedd9	-0.50966	1.516199	-13.3399	9.79E-13	2.06E-11
Oasl2	-0.50985	0.6553	-7.17223	1.82E-07	1.19E-06
Gsn	-0.51002	1.210764	-10.1969	2.67E-10	3.18E-09
Cdk14	-0.51062	1.330038	-9.05313	2.74E-09	2.62E-08
Klri1	-0.51108	0.987371	-8.19509	1.75E-08	1.40E-07
Lbh	-0.51251	2.280251	-11.4097	2.70E-11	3.95E-10
Grasp	-0.51251	0.926609	-6.47459	9.70E-07	5.46E-06
Snx20	-0.51428	1.278938	-7.37708	1.13E-07	7.68E-07
Mctp1	-0.51431	1.357636	-10.4878	1.52E-10	1.88E-09
Gsap	-0.51432	1.929529	-10.9074	6.84E-11	9.04E-10
LOC10263:	-0.51505	1.062834	-8.48827	9.20E-09	7.81E-08
Ehf	-0.51875	0.774066	-7.1784	1.79E-07	1.18E-06
Abi3	-0.51883	0.908018	-10.0273	3.74E-10	4.35E-09
Rrm2	-0.52098	0.641332	-9.76791	6.28E-10	6.79E-09
Il12rb2	-0.52103	1.577837	-12.2776	5.80E-12	1.01E-10
Stra6l	-0.52323	0.791214	-11.9459	1.03E-11	1.71E-10
Spic	-0.52335	0.812757	-5.69955	6.64E-06	3.18E-05
Pld4	-0.52414	2.171622	-10.3364	2.04E-10	2.46E-09
Map3k5	-0.52477	1.277746	-9.7658	6.30E-10	6.80E-09
Wdfy4	-0.52487	2.134006	-8.49315	9.10E-09	7.74E-08
Ccna2	-0.52513	1.243657	-7.66559	5.79E-08	4.20E-07
Slc12a6	-0.52539	0.932441	-9.93159	4.52E-10	5.11E-09
Casc5	-0.52542	0.964887	-10.8759	7.26E-11	9.54E-10
Dhx58	-0.52821	0.901528	-11.9259	1.07E-11	1.76E-10
H2-Ob	-0.53152	1.273583	-10.3612	1.94E-10	2.36E-09
Lrrc25	-0.53778	1.053569	-9.3867	1.37E-09	1.38E-08
Man1a	-0.53966	1.613316	-9.03608	2.84E-09	2.72E-08
Pla2g4a	-0.54139	0.592069	-9.23876	1.86E-09	1.84E-08

Chd7	-0.54161	1.321256	-10.5851	1.26E-10	1.58E-09
Fgd3	-0.54369	0.723748	-9.6739	7.59E-10	8.09E-09
Snora23	-0.54457	1.822742	-7.32127	1.28E-07	8.66E-07
Tcirg1	-0.54799	1.217138	-12.7595	2.55E-12	4.92E-11
Ctnnd2	-0.54818	0.561702	-12.3037	5.54E-12	9.68E-11
Mki67	-0.54994	1.565418	-10.0896	3.30E-10	3.88E-09
Tlr7	-0.55046	0.699443	-6.6212	6.79E-07	3.97E-06
Fem1c	-0.55158	1.562868	-12.272	5.85E-12	1.02E-10
Zbp1	-0.55263	1.549797	-7.15455	1.90E-07	1.24E-06
Hbb-bt	-0.55338	0.015461	-4.30211	0.000236	0.000801
Mta3	-0.55603	1.273066	-12.0736	8.26E-12	1.40E-10
Ifih1	-0.5564	1.452318	-11.4249	2.63E-11	3.88E-10
Ndc80	-0.5567	0.987613	-8.4738	9.49E-09	8.00E-08
Lrrk2	-0.55739	1.872552	-13.6262	6.17E-13	1.38E-11
Gm5086	-0.55817	0.564633	-8.24273	1.58E-08	1.27E-07
Ptchd1	-0.55834	0.414611	-9.9212	4.62E-10	5.19E-09
Ccnd1	-0.55981	1.986862	-11.6537	1.74E-11	2.68E-10
Clec4a3	-0.56172	1.035071	-4.02465	0.000479	0.001515
H2-Eb2	-0.56188	1.486942	-6.45136	1.03E-06	5.76E-06
Gm2848	-0.5626	0.458886	-8.62109	6.89E-09	6.07E-08
Mir3097	-0.56349	1.228646	-6.51883	8.71E-07	4.95E-06
Pilrb1	-0.56381	0.173698	-7.28747	1.39E-07	9.33E-07
Hps3	-0.56407	1.512233	-11.1127	4.66E-11	6.44E-10
Card11	-0.56415	1.124766	-9.94963	4.36E-10	4.97E-09
Ptpn18	-0.5651	1.060443	-10.4197	1.73E-10	2.12E-09
Cyp4f16	-0.56689	1.005464	-8.60585	7.12E-09	6.24E-08
Irgm2	-0.56932	1.208046	-7.79689	4.29E-08	3.19E-07
Smagp	-0.56948	0.709255	-12.095	7.96E-12	1.35E-10
Klf2	-0.56964	1.363651	-7.96781	2.91E-08	2.22E-07
Tiparp	-0.57039	1.398995	-9.39292	1.35E-09	1.36E-08
Apobec1	-0.57177	1.307174	-11.3104	3.24E-11	4.64E-10
Igsf6	-0.5753	1.860812	-12.5282	3.77E-12	6.87E-11
Top2a	-0.57568	1.585443	-11.3015	3.29E-11	4.70E-10
Jun	-0.57578	2.1115	-10.3602	1.94E-10	2.36E-09
Tbc1d9	-0.57806	2.095113	-13.7934	4.73E-13	1.09E-11
Rbm47	-0.57927	1.080027	-10.1136	3.15E-10	3.72E-09
Chst15	-0.58004	1.213893	-11.2189	3.83E-11	5.35E-10
Glrx	-0.58139	1.356765	-11.1041	4.74E-11	6.53E-10
Gm13807	-0.58183	0.682276	-6.91036	3.39E-07	2.10E-06
Exoc6	-0.58272	1.696862	-15.1972	5.59E-14	1.59E-12
Rgs1	-0.58373	2.52309	-10.4487	1.64E-10	2.02E-09
B230217C:	-0.58415	0.921103	-7.63693	6.18E-08	4.45E-07
Gm13420	-0.58458	0.59573	-5.70708	6.51E-06	3.12E-05
Gpr4	-0.58496	1.12371	-12.6981	2.83E-12	5.38E-11
Pik3cd	-0.58625	1.199323	-8.67461	6.14E-09	5.48E-08
Hmox1	-0.5866	0.543346	-6.8873	3.58E-07	2.20E-06
Cln3	-0.58781	1.256949	-13.2669	1.10E-12	2.30E-11
Tlr6	-0.58854	1.137245	-9.49293	1.10E-09	1.14E-08
Kif9	-0.58904	0.561346	-10.0788	3.37E-10	3.96E-09
Trim14	-0.5957	1.067998	-12.8909	2.05E-12	4.03E-11
Ubac2	-0.59984	1.609296	-14.0822	3.01E-13	7.32E-12
Itgav	-0.60178	1.328175	-12.4651	4.20E-12	7.52E-11

Ddx60	-0.60181	0.447152	-9.50227	1.08E-09	1.12E-08
Scimp	-0.6024	1.308994	-12.0208	9.06E-12	1.52E-10
Cdc14a	-0.6034	1.877045	-16.5292	8.50E-15	2.90E-13
Ptpre	-0.60432	1.28736	-10.4909	1.51E-10	1.88E-09
D930015E(	-0.60533	2.550422	-16.5435	8.34E-15	2.86E-13
Themis2	-0.61089	1.984586	-12.2388	6.20E-12	1.06E-10
LOC10263ε	-0.61519	1.779176	-6.73525	5.16E-07	3.08E-06
Mir1941	-0.61638	0.185529	-7.49326	8.61E-08	6.02E-07
Nxpe4	-0.61868	0.500084	-11.2752	3.46E-11	4.92E-10
Sucnr1	-0.6248	0.158672	-19.0491	3.35E-16	1.74E-14
Gm19327	-0.62541	0.961339	-8.24959	1.55E-08	1.25E-07
Itch	-0.62622	1.827294	-15.6463	2.92E-14	8.94E-13
Unc93b1	-0.62896	2.346754	-11.586	1.96E-11	2.99E-10
Scn3a	-0.63056	0.392322	-11.8013	1.34E-11	2.15E-10
Evi2b	-0.63332	1.264664	-7.76388	4.62E-08	3.42E-07
Il21r	-0.63347	1.598872	-10.1232	3.09E-10	3.66E-09
Kif11	-0.63384	0.994538	-13.1686	1.29E-12	2.68E-11
Gpr82	-0.63434	0.584406	-11.3274	3.14E-11	4.53E-10
Rab30	-0.63624	2.188504	-9.84312	5.40E-10	5.97E-09
Evl	-0.63631	1.568295	-15.64	2.95E-14	8.99E-13
Lipa	-0.63697	1.388302	-12.6986	2.83E-12	5.38E-11
Isg20	-0.6373	0.644014	-8.71908	5.58E-09	5.04E-08
Pglyrp2	-0.63767	0.557169	-11.0428	5.31E-11	7.26E-10
Tctex1d2	-0.63893	2.036257	-14.9199	8.41E-14	2.31E-12
Ltb	-0.64017	0.827588	-10.1776	2.78E-10	3.30E-09
Btg2	-0.64033	2.651412	-16.7961	5.92E-15	2.08E-13
9530059O:	-0.64364	0.449137	-12.6942	2.85E-12	5.41E-11
Slc44a5	-0.6441	0.218964	-12.4616	4.23E-12	7.52E-11
Cep70	-0.64477	1.089268	-11.4706	2.42E-11	3.59E-10
Pola1	-0.64684	0.874577	-14.2717	2.24E-13	5.62E-12
Fam46c	-0.6485	1.294003	-11.4982	2.30E-11	3.43E-10
Fcgr4	-0.6492	0.323658	-11.3661	2.93E-11	4.24E-10
Eef2k	-0.64936	0.432338	-13.8958	4.03E-13	9.51E-12
AI607873	-0.64997	1.702851	-5.08082	3.19E-05	0.000131
5830416P:	-0.65074	0.464934	-9.69183	7.32E-10	7.82E-09
Nrros	-0.65255	1.675092	-18.0868	1.10E-15	4.70E-14
Nr4a2	-0.65615	1.157286	-7.13686	1.98E-07	1.28E-06
Cfp	-0.65625	1.256875	-13.5654	6.80E-13	1.50E-11
Runx3	-0.6567	1.367346	-9.16765	2.15E-09	2.10E-08
Pak1	-0.6673	1.916905	-14.1742	2.61E-13	6.51E-12
Itgax	-0.66801	1.883534	-10.8443	7.70E-11	1.01E-09
I830077J0:	-0.67111	1.691977	-13.4228	8.56E-13	1.82E-11
Frmd5	-0.67256	0.570679	-11.2246	3.79E-11	5.31E-10
Nlrc4	-0.67965	0.396097	-15.5354	3.42E-14	1.01E-12
Ckb	-0.68098	1.607934	-11.8061	1.32E-11	2.13E-10
Lcp2	-0.68659	1.928275	-8.54767	8.08E-09	7.00E-08
Mycbp2	-0.68877	2.110172	-18.6466	5.47E-16	2.64E-14
Elmo1	-0.68917	2.069216	-19.3548	2.32E-16	1.32E-14
Lrrc18	-0.69052	1.228534	-10.6075	1.21E-10	1.53E-09
Flnb	-0.6919	1.811986	-17.8616	1.46E-15	6.08E-14
Fgl2	-0.69571	1.848233	-13.9655	3.61E-13	8.61E-12
Ube2e2	-0.69995	0.85919	-11.9338	1.06E-11	1.74E-10

Klrb1f	-0.70568	0.524359	-9.3352	1.52E-09	1.52E-08
Arap2	-0.70631	1.230333	-15.6288	2.99E-14	9.09E-13
Coro2a	-0.70829	1.690508	-9.55203	9.73E-10	1.02E-08
Nfkbid	-0.71303	1.42604	-9.19795	2.02E-09	1.98E-08
Hist1h2ab	-0.71389	1.46166	-7.07845	2.27E-07	1.46E-06
Pou2f2	-0.71402	0.303796	-14.0266	3.28E-13	7.90E-12
Gpr114	-0.71413	1.119862	-9.76071	6.37E-10	6.86E-09
Serpinb10	-0.71601	0.511069	-11.2124	3.88E-11	5.41E-10
Fam134b	-0.71957	1.001637	-11.8394	1.25E-11	2.02E-10
Havcr2	-0.7283	1.558458	-12.6593	3.02E-12	5.65E-11
LOC73899	-0.72854	1.568077	-12.9792	1.77E-12	3.53E-11
Gm8817	-0.72937	0.343361	-11.6678	1.70E-11	2.62E-10
Rasgrp3	-0.73593	1.401037	-15.9385	1.93E-14	6.06E-13
Gpr124	-0.73957	0.90031	-15.6131	3.06E-14	9.16E-13
Myo1f	-0.74404	1.53964	-12.4101	4.62E-12	8.18E-11
Tspan2	-0.74509	0.604995	-12.691	2.86E-12	5.42E-11
Ier3	-0.74554	1.16167	-6.71904	5.36E-07	3.19E-06
Spns3	-0.74718	0.934194	-14.6386	1.28E-13	3.43E-12
St8sia6	-0.74996	1.152652	-10.0362	3.67E-10	4.28E-09
Ifi27	-0.75468	1.419099	-13.5016	7.54E-13	1.64E-11
Rundc3b	-0.76209	0.2708	-10.9624	6.17E-11	8.29E-10
Gm16894	-0.76383	1.026306	-9.73588	6.70E-10	7.18E-09
St3gal5	-0.76605	0.979247	-14.0759	3.04E-13	7.36E-12
Ddit4	-0.77045	0.43851	-7.9768	2.86E-08	2.19E-07
4930469K1	-0.77087	0.581902	-11.8921	1.14E-11	1.85E-10
Adrb2	-0.77191	1.141825	-12.9636	1.81E-12	3.60E-11
Gm15232	-0.77309	0.800394	-10.9198	6.68E-11	8.85E-10
Ifngr1	-0.77371	2.117265	-17.2395	3.28E-15	1.23E-13
1600010M	-0.77661	0.934366	-11.4204	2.65E-11	3.90E-10
Fam71a	-0.78012	0.404537	-8.59434	7.30E-09	6.38E-08
Rgs2	-0.78063	2.485835	-17.5513	2.18E-15	8.53E-14
Mefv	-0.7844	0.646981	-13.2972	1.05E-12	2.19E-11
Slamf8	-0.78603	1.349638	-13.8152	4.57E-13	1.06E-11
Ddx58	-0.79081	1.015201	-11.9574	1.01E-11	1.68E-10
LOC102638	-0.79603	1.407432	-9.57321	9.32E-10	9.81E-09
Dusp6	-0.80204	1.046475	-14.3819	1.89E-13	4.93E-12
Stap1	-0.80742	1.479073	-17.1071	3.91E-15	1.43E-13
Dscam	-0.80774	0.620645	-15.2357	5.28E-14	1.51E-12
Snord89	-0.81523	0.830759	-7.41483	1.03E-07	7.07E-07
Parvg	-0.81774	1.374577	-14.7909	1.02E-13	2.79E-12
Kif23	-0.81824	1.207015	-16.6352	7.36E-15	2.55E-13
Pglyrp1	-0.83104	1.617536	-16.1915	1.35E-14	4.46E-13
Plk3	-0.83232	0.515934	-14.3584	1.96E-13	5.06E-12
Cd72	-0.83334	0.831036	-13.0547	1.56E-12	3.14E-11
Tns3	-0.83884	1.533104	-14.6812	1.20E-13	3.24E-12
Rtn1	-0.84412	0.897752	-20.7214	4.78E-17	3.46E-15
Mapk14	-0.8506	1.363359	-19.427	2.13E-16	1.23E-14
Kit	-0.85486	1.794821	-11.6289	1.82E-11	2.79E-10
Art2a-ps	-0.86269	1.589085	-12.5975	3.35E-12	6.22E-11
Acp5	-0.87171	0.636363	-13.3895	9.03E-13	1.91E-11
Ptgs2	-0.87528	1.272912	-10.6854	1.04E-10	1.33E-09
Gm19345	-0.88436	0.20565	-13.2658	1.10E-12	2.30E-11

Gm14446	-0.88839	0.655058	-10.6465	1.12E-10	1.43E-09
Tm6sf1	-0.88999	1.357527	-17.7018	1.80E-15	7.19E-14
Mir1962	-0.8927	0.239111	-14.3184	2.09E-13	5.29E-12
Prss30	-0.89804	0.553466	-15.988	1.80E-14	5.72E-13
AF251705	-0.89832	1.222578	-8.9862	3.15E-09	3.00E-08
Ccl4	-0.90055	0.832529	-8.78869	4.80E-09	4.40E-08
Il1b	-0.90687	1.522383	-12.5907	3.39E-12	6.28E-11
Hap1	-0.90702	0.483336	-16.9016	5.14E-15	1.85E-13
Tmem131	-0.91544	1.934896	-20.6952	4.92E-17	3.47E-15
Serpinb2	-0.91694	0.121819	-17.5492	2.19E-15	8.53E-14
Rtp4	-0.92473	0.660758	-11.3664	2.92E-11	4.24E-10
Haus8	-0.93096	1.489092	-15.5749	3.23E-14	9.55E-13
Dnase1l3	-0.93382	2.665598	-16.8485	5.52E-15	1.96E-13
Hist1h2bg	-0.93544	1.664056	-6.54661	8.14E-07	4.67E-06
Gpr34	-0.93604	0.907579	-16.8524	5.49E-15	1.96E-13
Nod1	-0.94805	0.910597	-19.3904	2.22E-16	1.27E-14
Nlrp3	-0.95278	0.670383	-16.7421	6.37E-15	2.23E-13
Fcrl1	-0.96265	0.865597	-18.449	6.98E-16	3.21E-14
Nlrp1b	-0.96619	0.942504	-16.1695	1.40E-14	4.58E-13
Ifitm3	-0.97116	2.143782	-11.2385	3.70E-11	5.18E-10
Npl	-0.97191	0.319562	-19.1747	2.88E-16	1.53E-14
St14	-0.97871	0.475177	-19.2804	2.53E-16	1.40E-14
Usp6nl	-0.98503	1.448143	-19.9691	1.13E-16	7.17E-15
Hpgd	-0.9857	0.820516	-12.2728	5.85E-12	1.02E-10
Apobr	-0.98658	1.189331	-18.2227	9.26E-16	4.10E-14
Cd101	-0.98758	0.256842	-20.6062	5.44E-17	3.78E-15
Rasgef1b	-0.99843	1.356865	-13.0826	1.49E-12	3.01E-11
Ggt5	-0.99909	0.229851	-24.4189	1.02E-18	1.19E-16
Emr1	-1.0001	0.649904	-24.2768	1.17E-18	1.28E-16
Ptger3	-1.00044	0.091749	-22.9126	4.57E-18	4.20E-16
Lst1	-1.00339	2.113629	-14.6215	1.32E-13	3.51E-12
Egr2	-1.00713	0.961982	-12.7682	2.51E-12	4.86E-11
Ptgs2os2	-1.00936	0.208066	-22.8587	4.83E-18	4.33E-16
Slc6a12	-1.01488	0.442864	-25.277	4.52E-19	6.00E-17
Ptpn22	-1.01549	1.732828	-17.1041	3.92E-15	1.43E-13
Atf3	-1.01781	1.782712	-17.905	1.38E-15	5.79E-14
Cd81	-1.01985	2.650918	-13.1019	1.44E-12	2.94E-11
LOC10263[illegible]	-1.02137	0.817779	-15.7109	2.66E-14	8.19E-13
2900052N[illegible]	-1.02175	0.24753	-16.3927	1.03E-14	3.45E-13
Ceacam16	-1.02929	0.210603	-16.8021	5.87E-15	2.07E-13
Bank1	-1.04103	0.423383	-19.9176	1.20E-16	7.53E-15
Mdh2	-1.04197	2.50832	-24.1142	1.37E-18	1.47E-16
LOC10263[illegible]	-1.04267	0.417276	-18.2013	9.51E-16	4.19E-14
Gm12159	-1.05135	0.331989	-18.2728	8.70E-16	3.90E-14
Cd300lf	-1.05704	0.54617	-14.1426	2.74E-13	6.82E-12
Ncf4	-1.07161	1.273893	-19.4572	2.05E-16	1.19E-14
Hpgds	-1.07496	0.389801	-21.5953	1.83E-17	1.46E-15
Sh2d1b1	-1.08175	0.608391	-18.3619	7.78E-16	3.52E-14
Nlrp1c-ps	-1.08413	0.991494	-13.7174	5.34E-13	1.21E-11
Trpm2	-1.08429	0.616235	-21.8164	1.44E-17	1.19E-15
Tagap	-1.08468	1.552896	-18.4724	6.78E-16	3.15E-14
Cd300ld	-1.08612	0.451175	-21.719	1.60E-17	1.29E-15

Trim30b	-1.08732	0.847152	-12.5279	3.77E-12	6.87E-11
Treml4	-1.09158	1.297102	-14.524	1.53E-13	4.01E-12
Itgam	-1.09305	0.550066	-21.5422	1.93E-17	1.52E-15
Hes1	-1.11114	0.350273	-22.916	4.55E-18	4.20E-16
Gm5150	-1.12378	0.415872	-12.7912	2.42E-12	4.73E-11
Myo1b	-1.12787	0.249314	-24.6873	7.90E-19	9.64E-17
Egr1	-1.13176	1.966952	-18.6283	5.59E-16	2.69E-14
Tlr1	-1.14048	1.209603	-14.1404	2.75E-13	6.82E-12
Ndst1	-1.14605	0.440279	-25.0478	5.61E-19	7.20E-17
Pram1	-1.1518	0.505199	-20.1786	8.85E-17	5.68E-15
Krt80	-1.1605	0.615467	-15.7657	2.46E-14	7.64E-13
Pstpip2	-1.1624	0.577597	-19.2599	2.60E-16	1.41E-14
Scel	-1.17239	0.082234	-33.6427	4.96E-22	3.07E-19
Dock5	-1.18459	1.459792	-23.954	1.61E-18	1.69E-16
Cd97	-1.19223	2.392013	-19.7497	1.45E-16	8.95E-15
Chn2	-1.20647	1.069902	-21.1295	3.04E-17	2.28E-15
Arhgap6	-1.21634	0.402041	-22.6448	6.02E-18	5.33E-16
Ccrl2	-1.21756	1.584654	-18.1936	9.61E-16	4.21E-14
Tgfbi	-1.221	1.823261	-18.6855	5.21E-16	2.59E-14
Oas2	-1.22925	0.429983	-16.0528	1.64E-14	5.32E-13
LOC10264(	-1.25701	0.699833	-18.4383	7.07E-16	3.23E-14
Egr3	-1.29185	0.842125	-16.3587	1.07E-14	3.60E-13
Hck	-1.29584	1.743014	-21.1007	3.13E-17	2.33E-15
Bex6	-1.32348	0.458124	-25.0004	5.87E-19	7.28E-17
Cyp4f37	-1.33087	0.157285	-30.7211	4.37E-21	1.41E-18
Snord13	-1.34316	1.138722	-11.1973	3.99E-11	5.54E-10
Ffar2	-1.34563	0.526936	-27.4552	6.37E-20	1.32E-17
Clec4a1	-1.34641	0.911104	-16.6021	7.70E-15	2.65E-13
Sirpb1b	-1.37931	0.645278	-18.2323	9.15E-16	4.08E-14
Siglece	-1.39215	0.307618	-26.6406	1.30E-19	2.20E-17
Abcb1a	-1.42083	0.423594	-25.2596	4.60E-19	6.00E-17
Neurl3	-1.46859	1.19601	-19.3093	2.45E-16	1.37E-14
Gpr141	-1.48242	0.880491	-26.9946	9.53E-20	1.73E-17
Abcg3	-1.48876	1.239577	-30.4004	5.62E-21	1.74E-18
Mmp12	-1.50548	0.741985	-18.6611	5.37E-16	2.61E-14
Ppp1r14a	-1.52147	0.55481	-25.9296	2.47E-19	3.68E-17
Tnf	-1.52437	1.69192	-21.5601	1.90E-17	1.50E-15
Dtx1	-1.55182	0.564379	-25.409	4.00E-19	5.51E-17
Gm9733	-1.56643	0.457963	-18.0578	1.14E-15	4.85E-14
Sash1	-1.57216	0.628783	-38.6673	1.74E-23	1.62E-20
Sirpb1a	-1.61045	1.091921	-24.652	8.17E-19	9.70E-17
Cd22	-1.6213	0.648885	-31.5195	2.37E-21	1.04E-18
Gm6377	-1.70042	1.587298	-31.3532	2.68E-21	1.05E-18
Ccl3	-1.72086	1.028989	-12.4621	4.22E-12	7.52E-11
Slc12a2	-1.81059	0.897124	-41.4755	3.19E-24	3.40E-21
Nr4a1	-1.86361	1.815156	-26.6994	1.24E-19	2.14E-17
Tgm2	-1.90494	0.462198	-37.92	2.78E-23	2.30E-20
Ifitm6	-2.03339	0.579692	-36.4704	7.12E-23	4.81E-20
Lphn3	-2.07399	0.318566	-45.3836	3.61E-25	6.72E-22
Asb2	-2.20194	0.701125	-49.2127	5.06E-26	3.31E-22
Clec4a4	-2.24254	0.459241	-43.6413	9.32E-25	1.39E-21
